# How to Get the Most out of Your Curation Effort

**DOI:** 10.1371/journal.pcbi.1000391

**Published:** 2009-05-22

**Authors:** Andrey Rzhetsky, Hagit Shatkay, W. John Wilbur

**Affiliations:** 1 Department of Medicine, University of Chicago, Chicago, Illinois, United States of America; 2 Department of Human Genetics, University of Chicago, Chicago, Illinois, United States of America; 3 Computation Institute, University of Chicago, Chicago, Illinois, United States of America; 4 Institute for Genomics and Systems Biology, University of Chicago, Chicago, Illinois, United States of America; 5 The Computational Biology and Machine Learning Lab, School of Computing, Queen's University, Kingston, Ontario, Canada; 6 National Center for Biotechnology Information, National Library of Medicine, National Institutes of Health, Bethesda, Maryland, United States of America; University of California San Diego, United States of America

## Abstract

Large-scale annotation efforts typically involve several experts who may disagree with each other. We propose an approach for modeling disagreements among experts that allows providing each annotation with a confidence value (i.e., the posterior probability that it is correct). Our approach allows computing certainty-level for individual annotations, given annotator-specific parameters estimated from data. We developed two probabilistic models for performing this analysis, compared these models using computer simulation, and tested each model's actual performance, based on a large data set generated by human annotators specifically for this study. We show that even in the worst-case scenario, when all annotators disagree, our approach allows us to significantly increase the probability of choosing the correct annotation. Along with this publication we make publicly available a corpus of 10,000 sentences annotated according to several cardinal dimensions that we have introduced in earlier work. The 10,000 sentences were all 3-fold annotated by a group of eight experts, while a 1,000-sentence subset was further 5-fold annotated by five new experts. While the presented data represent a specialized curation task, our modeling approach is general; most data annotation studies could benefit from our methodology.

## Author Summary

Data annotation (manual data curation) tasks are at the very heart of modern biology. Experts performing curation obviously differ in their efficiency, attitude, and precision, but directly measuring their performance is not easy. We propose an experimental design schema and associated mathematical models with which to estimate annotator-specific correctness in large multi-annotator efforts. With these, we can compute confidence in every annotation, facilitating the effective use of all annotated data, even when annotations are conflicting. Our approach retains all annotations with computed confidence values, and provides more comprehensive training data for machine learning algorithms than approaches where only perfect-agreement annotations are used. We provide results of independent testing that demonstrate that our methodology works. We believe these models can be applied to and improve upon a wide variety of annotation tasks that involve multiple annotators.

## Introduction

Virtually every large-scale biological project today, ranging from creation of sequence repositories, collections of three-dimensional structures, annotated experiments, controlled vocabularies and ontologies, or providing evidence from the literature in organism-specific genome databases, utilizes manual curation.

A typical curation task in biology and medicine involves a group of experts assigning discrete codes to a datum, an experimental observation, or a text fragment. For example, curators of the PubMed database assign topics to each article that is registered in the database. These topics are encoded in a hierarchical MESH terminology [1] to ensure that curators have a consistent way to define an article's content. Other curation examples include annotation of function of genes and proteins, description of genetic variation in genomes, and cataloguing human phenotypes. A standard approach to assessing quality of curation involves computation of inter-annotator agreement [2], such as a kappa-measure [3].

Manual curation is tedious, difficult, and expensive. It typically requires annotation by multiple people with variable attitudes, productivity, stamina, experience, tendency to err, and personal bias. Despite its difficulties and the imprecision in outcome, curation is critical. Existing curation approaches can be improved and enhanced with careful experimental design and appropriate modeling. This study aims to address the following questions:

- How can we account for, and possibly utilize, annotator heterogeneity?
- What should we do with several conflicting annotations? (They are often wastefully discarded.)
- How can we quantify confidence in the quality of any particular annotation?

In this study we propose a holistic *approach to quantify our certainty in individual annotations* for a group of several annotators, which allows to retain the complete dataset as a basis for training and testing machine learning methods.

Specifically, we suggest an internally consistent way to design annotation experiments and analyze curation data. We created two alternative probabilistic models for such analysis, tested these models with computer simulations, and then applied them to the analysis of a newly annotated corpus of roughly 10,000 sentences. Each sentence in this corpus was annotated by three experts. To test the utility of our computational predictions, we randomly sampled a subset of 1,000 sentences (out of the original 10,000) to reannotate by five new experts. Using these two rounds of annotation, we evaluated the models' predictions by comparing the three-experts-per-sentence results against the “gold standard” eight-experts-per-sentence analysis.

## Methods

### Corpus: Two cycles of annotations

First, to generate the corpus, our homemade scripts extracted 10,000 full sentences randomly from diverse scientific texts, making sure that all sentences are distinct and that section-specific and topic-specific constraints are met. Specifically, we randomly selected 1,000 sentences from the PubMed database, which at the time of our analysis stored 8,039,972 article abstracts (note that not every PubMed entry comes with an abstract). We also sampled 9,000 sentences from the GeneWays corpus (368,331 full-text research articles from 100 high-impact biomedical journals). We put the following constraints on these 9,000 sentences: 2,100 sentences were sampled from articles related to *WNT pathways*, *apoptosis*, or *schizophrenia research* (700 sentences per topic, with random sampling within each pool of topic-specific articles). The remaining 6,900 sentences were sampled with restriction on article section: 20% of the sentences came from abstracts, 10% from introductions, 20% from methods, 25% from results, and 25% from article discussion sections. We did not process sentences in any way before the annotation. Because the current study is not concerned with automatic annotation of sentence fragments per se, we do not elaborate on machine-learning features that we described in our earlier study [4].

**Figure 1:**
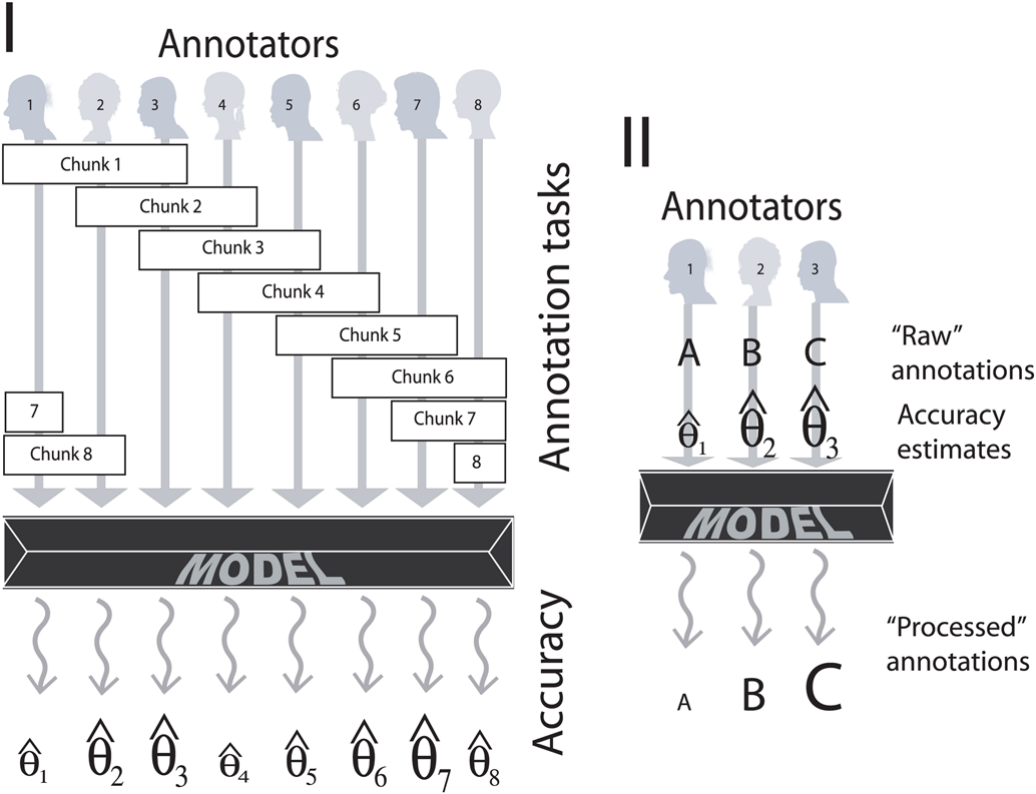
Two stages of our analysis: annotation (I) and inference (II). First, we used a loop design of experiments to generate annotation data and to estimate the annotator-specific correctness parameters (I). Second, we used the correctness parameter estimates obtained to resolve annotation conflicts and estimate the posterior probability associated with each alternative annotation (II). The probabilistic model is depicted as a dark prism. We had eight annotators grouped into three-annotator groups in such a way that each annotator participated in exactly three groups and all groups were different. This ensured that we could recover correctness estimates for all eight annotators even though some of them (for example, annotators 2 and 7) never annotated the same fragment of text. (Size of symbols representing hypothetical correctness parameter estimates is intended to indicate the magnitude of the corresponding value.)

Second, we randomly reordered the 10,000 sentences and partitioned them into eight equal-size sets. We arranged eight annotators recruited for the first cycle of analysis into eight 3-annotator groups, assigning to each group a unique sentence set. This way each annotator analyzed three sets of sentences, and utilizing the loop-design of the analysis (see Figure 1A) we were able to computationally compare annotators' performances with each other. This concluded the first cycle of annotation. The part of this corpus on which all three annotators perfectly agreed, as well as the part on which at least two out of the three agreed, were used for training, testing and analyzing supervised machine learning methods for automatic annotation assignment, in a recent study, reported elsewhere [4].

As the models for annotation reliability introduced here are based on the above corpus, to reliably validate the models, we performed a second cycle of annotation. To do this, we recruited five additional annotators, sampled a subset of 1,000 random sentences out of the original 10,000, and asked the new annotators to annotate the 1,000-sentence subset. The result of the second cycle of annotation was a 1,000-sentence set that was annotated by five annotators per sentence in the second cycle and by three annotators per sentence in the first cycle.

The whole annotated corpus is publicly available along with this manuscript (see *Dataset S*1).

### Rationale for producing the corpus

When defining guidelines for our present annotation effort [5] we aimed at distinguishing among several types of scientific statements, varying across multiple dimensions. Specifically, we tried to distinguish commonplace knowledge from original conclusions, high certainty statements from uncertain ones, experimentally supported evidence from speculations, and scientific statements from methodological or meta-statements. The goal of this effort was to generate a manually annotated corpus that can be further used to train computers to automatically perform well-defined annotation tasks at a large scale.

In the long run, we hoped to learn to automatically highlight portions of research articles that fit a particular search goal. Such a goal can be, for example, to identify all original conclusions supported by experiments. Another plausible goal (out of many imaginable) is to find the scientific statements made with high certainty, with or without experimental support. A tool of this kind would be a useful addition to the armamentarium of a biomedical text-miner.

### Annotations

We asked experts to annotate sentences along the following six dimensions (with two of them, polarity and certainty, combined), described in great detail in an earlier article [5] :

- *Focus* allowed values G, M, and S for *generic* (“Financial support was provided by X agency”), *methodology* (“In this application we used an RT-PCR technique.”), and *science* (“Our experiments support the former of the two hypotheses.”), respectively; Combinations such as GM, GS, MS, and GMS are allowed when necessary.
- *Evidence* allowed codes E0, E1, E2, and E3, where E0 is the complete lack of evidence and E3 is direct evidence present in the sentence. E1 and E2 are somewhat in between: E1 corresponds to a claim of evidence that is implied but is not supported by a reference to other publications or by original data, while E2 represents the case of an explicit reference within the sentence to other publications.
- *Polarity* (P and N for *positive* and *negative*) and *certainty* (0, 1, 2, 3) are combined such that 0 is completely uncertain and 3 is absolutely certain, in positive and negative directions. As such, the seven possible codes (N3, N2, N1, N0, P0, P1, P2, and P3) correspond to increasing positive certainty.
- *Trend* or *direction* captures changes in a quantity or a process described in the sentence, such as the increase or decrease of a particular property. Only a minority of the sentences was annotated with trend/direction codes and for this reason we do not analyze them here.
- For *number of fragments in the sentence*, we asked annotators to break the sentence into fragments each time one of the above properties changed, see Table 1. (The number of sentence fragments does not formally belong to the list of annotation types that we defined for this study. Nevertheless, this property of annotations follows directly from fragmentation of sentences according to our guidelines and therefore can serve as a legitimate annotation dimension.)

**Table 1:** Example of a sentence from the dataset, annotated by 5 independent annotators (sentence 10835394_70).

Annotations	3 fragments (A, B, C)	5 annotators (A1–A5)
*Number of sentence fragments*		A1: **1**
		A2: **2**
		A3: **1**
		A4: **2**
		A5: **2**
*Evidence*	**A** |A1:E3|A2:E3|A3:E3|A4:E3|A5:E3	A1: **A**|E3 **B**|E3 **C**|E3
	**B** |A1:E3|A2:E1|A3:E3|A4:E3|A5:E3	A2: **A**|E3 **B**|E1 **C**|E1
	**C** |A1:E3|A2:E1|A3:E0|A4:E0|A5:E3	A3: **A**|E3 **B**|E3 **C**|E0
		A4: **A**|E3 **B**|E3 **C**|E0
		A5: **A**|E3 **B**|E3 **C**|E3
*Focus*	**A** |A1:S|A2:S|A3:S|A4:S|A5:G	A1: **A**|S **B**|S **C**|S
	**B** |A1:S|A2:S|A3:S|A4:S|A5:G	A2: **A**|S **B**|S **C**|S
	**C** |A1:S|A2:S|A3:S|A4:S|A5:G	A3: **A**|S **B**|S **C**|S
		A4: **A**|S **B**|S **C**|S
		A5: **A**|G **B**|G **C**|G
*Polarity-Certainty*	**A** |A1:P3|A2:P3|A3:P2|A4:P2|A5:P3	A1: **A**|P3 **B**|P3 **C**|P3
	**B** |A1:P3|A2:P3|A3:P2|A4:P2|A5:P3	A2: **A**|P3 **B**|P3 **C**|P3
	**C** |A1:P3|A2:P3|A3:P2|A4:P3|A5:P3	A3: **A**|P2 **B**|P2 **C**|P2
		A4: **A**|P2 **B**|P2 **C**|P3
		A5: **A**|P3 **B**|P3 **C**|P3

Annotations in the context of the real sentence are as follows:

**The phenotypes of mxp19 (** **Fig 1B** **)** |A2:**1SP3E3| **and mxp170 (data not shown) homozygotes and hemizygotes (data not shown) are identical**, |A3:**1SP3E3| |A4:**1SP3E3| |A5:**1GP3E3| **suggesting that mxp19 and mxp170 are null alleles**. |A1:**1SP3E3| |A2:**2SP3E1| |A3:**1SP2E0| |A4:**2SP2E0| |A5:**2GP2E3|

The minimum number of sentence fragments required to represent these annotations is three:

**A** = “The phenotypes of mxp19 (Fig 1B)”

**B** = “and mxp170 (data not shown) homozygotes and hemizygotes (data not shown) are identical,”

**C** = “suggesting that mxp19 and mxp170 are null alleles.”

Annotators' identities are concealed with codes A1, A2, A3, A4, and A5.

In addition, each annotation of a dimension is allowed to have code *Error*, indicating erroneously extracted or jumbled sentence.

As the focus of this work is the construction of models for annotation correctness, we next describe these models.

### Models

#### Folk wisdom of modeling

Time and energy permitting, we could design an infinite number of mathematical models to compete in describing the same real-life process. Every model with a circumspect number of parameters and computable probabilities must unavoidably incorporate simplifying assumptions. Nevertheless, some models portray reality better than their rivals, and some models are efficient enough to become practically useful. Our goal was to develop a model that is sufficiently realistic, demonstrably useful and easy to implement. We believe that we succeeded in this paper in achieving this goal.

#### Grouping annotators

The experimental design incorporated in our present analysis is a special case of *incomplete block design* suggested by Frank Yates [6]; the specific *loop design* version is due to Kerr and Churchill, 2001 [7].

Our task in this study was to infer correct annotation for each multi-annotator experiment by estimating annotator-specific error rates. It is important to note that the correctness of an annotation is not an observable or known attribute of the data, as we have no direct way to identify which annotation is correct. We thus must view the underlying correctness (or incorrectness) of each annotation as a hidden or, as often referred to in the Bayesian framework, *latent* variable.

While we focus here on text data, sampled from research articles, and annotated by eight experts along certain pre-defined dimensions, the ideas presented are not specific to this task. The same experimental approach is applicable to cases where the number of annotators as well as the type of data and nature of the annotation task are different.

As described earlier, to obtain expert annotations, we used 10,000 randomly ordered sentences, partitioned into eight equal-size sets. Each set was annotated by three of the annotators. Annotators were grouped following a *loop design* (see Figure 1I): That is, the first group included annotators 1, 2, and 3, the second 2, 3, and 4, and so on, with the eighth group consisting of annotators 8, 1, and 2.

**Figure 2:**
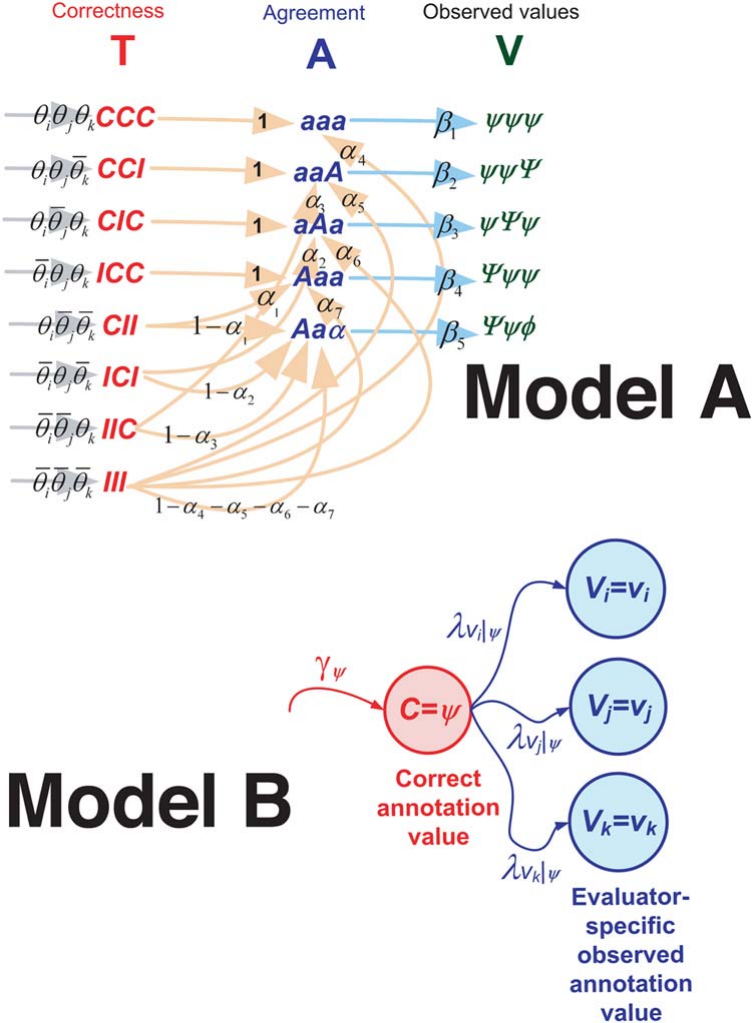
Graphic outline of the two generative models of text annotations introduced in this study (A and B).

To estimate annotator-specific accuracies and to use these correctness estimates to infer the correct annotation (see Figure 1, I and II), we developed probabilistic models, symbolized by a prism in Figure 1. We designed and tested two quite different probabilistic models based on the same data (see Figure 2). Both models are *generative*, which means they can be used to generate artificial data. Both models represent the task of annotating a single-sentence by a triplet of annotators, and both allow us to express annotator-specific correctness. However, the two models differ in their complexity (number of independent parameters), and in the underlying generative process that they represent.

Importantly, in the computation of the likelihood value for both models, we used the informative prior distribution for the model parameters, 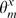, namely, the beta-distribution with parameters *a* = 2 and *b* = 1, (see [8], p. 37 in v. 2):
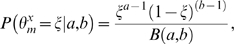
(1)that roughly corresponds to the assumption that an average annotator tends to be correct in more than 50% of her annotations. (This assumption is used to decrease the number of equal-height modes of the posterior distribution under each model. If we drop the assumption, situation where all annotators are incorrect – given the observed perfect agreement in a triplet of annotators – will be as likely as situation where all annotations are correct.) The detailed equations for both models are given in the *Text S1*. The equations, while somewhat cumbersome, are straightforward to derive and easy to implement and compute.

#### The two models: Rationale

Clearly, annotation of data by experts is not really a stochastic process. However, formally modeling the annotation generation process using a probabilistic generative model can account for disagreements and uncertainty inherent to this task, which involves subjective judgment by multiple individuals. A generative model is tightly coupled with a putative probabilistic process that is assumed to generate the data (in this case, the annotated corpus). Different assumptions about the generative process, may give rise to different probabilistic generative models. In particular, we considered two scenarios. One (Model A) is parsimonious in the number of parameters, but more cumbersome and less intuitive in terms of the underlying generation process. The other (Model B) is simpler and more intuitive, but involves a larger number of parameters. We thus focus first on Model B.

Model B, assumes that for each datum to be annotated, the correct value of annotation is defined first – by sampling from a distribution of correct annotations. The observed individual annotations are then generated by sampling their values from another distribution that differs for different annotators, and for the distinct correct annotation values.

An alternative scenario, corresponding to Model A, is more artificial and a bit more cumbersome. In this case, the first step of the data generation is to decide for each annotator whether or not she is correct. Next, given the annotator's correctness indicators, an inter-annotator agreement pattern is decided probabilistically. (Note that at this stage all that is determined is an agreement pattern, i.e. which annotators must agree and which must disagree, but the precise annotation values are unknown.) Finally, another probabilistic process is used to generate the actual annotation values given the agreement patterns. Model A does not require estimating the distribution of correct annotations.

We considered other data-generation scenarios but will not discuss them here.

Both models have their pros and cons. Ultimately, a model is validated by its practical utility; it is likely that each of our models can show superior performance over the other given a favorable configuration of a specific data set. We thus implemented and thoroughly tested both models. We focus here on Model B and only briefly describe Model A. For details regarding Model A the reader is referred to the *Text S1*.

#### Model A

The idea behind Model A is that for each sentence and each triplet of annotators, the generation of annotations follows a three-stage stochastic process, see Figure 2, Model A. Given three annotators (*i*, *j*, and *k*), **the first stage** assigns each of the three annotators his/her probability of being correct, giving rise to a three-dimensional annotator-correctness vector, **T** *_ijk_*.

**The second stage** of the annotation-generating process involves producing an agreement pattern, **A** *_ijk_*, conditioned on the state of **T** *_ijk_* (see *Text S1*).

**The third stage** of data generation involves producing a triplet of observed annotations, **V** *_ijk_*, given the agreement pattern, **A** *_ijk_*.

#### Model B

Model B is based on a simpler underlying generation process (see Figure 2 (model B)). Rather than focusing on the annotators probability to be correct and on their probability to agree with each other, the model directly accounts for the probability that annotations are correct. A set of parameters, denoted by *γ_j_*, (for each annotation value *j*), represent the probability that each annotation value is correct. The same set of parameters is assumed to apply to the entire collection of sentences. For example, if the allowed annotation values are *1, 2*, and *3*, the probabilities that the corresponding annotations are correct could be, for example, *γ*_1_ = 0.4, *γ*_2_ = 0.35, and *γ*_3_ = 0.25, which sum to 1, thus forming a probability distribution, as required.

Further, for the *i* ^th^ annotator (*i* = 1, 2, …, 8), we introduce a matrix of probabilities, denoted *λ* ^(*i*)^ *_x|y_*, that defines the probability that this annotator assigns annotation value *x* to a text fragment with correct annotation *y*. A hypothetical perfect annotator would have the matrix *λ* ^(*i*)^ *_x|y_* equal to the identity matrix, with ones on the diagonal and zeros elsewhere.

Generating mock annotations is a simple two-stage process. In **the first stage**, we sample the correct value of the annotation for each sentence fragment using parameters *γ_z_*. In **the second stage**, using the known values of *λ* ^(*i*)^ *_x|y_*, *λ* ^(*j*)^ *_x|y_*, and *λ* ^(*k*)^ *_x|y_*, we sample the observed annotation values for a triplet of annotators, *i*, *j*, and *k*.

To speed up computation and to ease direct comparison with Model A, we implemented both complete and simplified versions of Model B. In the simplified version, we kept the *γ*-parameters unchanged but postulated that *λ* ^(*k*)^ *_y|y_* = *θ_k_* and *λ* ^(*k*)^ *_x|y_* = (1−*θ_k_*)/(*m*−1) for all *x≠y*, where *m* is the total number of distinct annotation values, and *θ_k_* is the expected correctness of the *k* ^th^ annotator. We refer to this simplified version of our B-model as *B-with-thetas*. While these *θ*-parameters have the same meaning as *θ*-parameters in Model A, the two models have quite different properties as is evidenced by out experiments, shown in the following sections.

Formally stated, the joint probability that the annotations provided by the three evaluators are correct (under Model B) is given by:
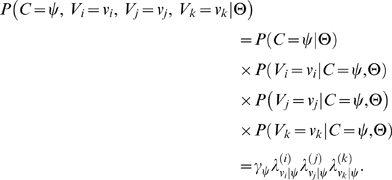
(2)


Here *C* denotes the random variable that represents the correct value for the given instance of annotation, *V_i_*, *V_j_*, and *V_k_* are random variables representing the annotation values assigned by the three annotators, and Θ are the parameters of Model B. So long as the correct value is unknown, the likelihood of the three annotations given the model parameters is obtained by integration over all possible correctness values:
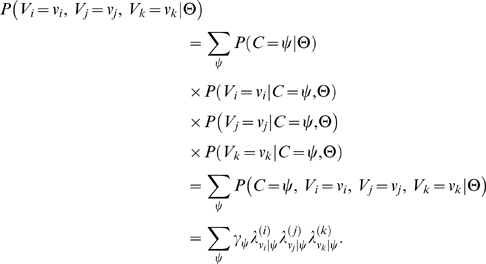
(3)


When the parameter values are known (estimated), we can compute the posterior distribution of correct values given the observed annotations and parameter values:
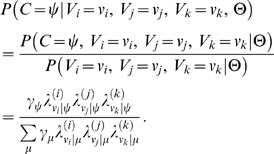
(4)


Finally, we can directly compare annotator-specific correctness under Model A (*θ*'s) with analogous values computed under Model B using the following relation under Model B:
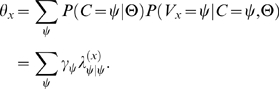
(5)


## Results

### Comparing the two models

Despite the apparent complexity of the generative process under Model A, in its simplest form the model requires only one parameter per annotator for any number of allowed annotation values. In contrast, for Model B, given *n* permissible annotation values, there are *n*−1 independent values of *γ*'s and *one* independent value of *λ* ^(*i*)^ *_x|x_* for *each* annotator. As a result, for the number of fragments in a sentence that allows 9 values, Model B requires optimization of a likelihood function depending on 16 free parameters (584 for the full model), whereas the likelihood for Model A depends only on 8 (11 for the full model).

It is well known that if we estimate parameters using numerical function optimization over a fixed-sized dataset, it is much easier and quicker to obtain the maximum-likelihood estimates when the number of model parameters is small. As the number of parameters increases, the data is typically insufficient to uniquely determine the parameter values, and parameter estimates may widely vary. As our experiments with simulated data illustrate (see below), the number of local optima grew exponentially with the number of permitted annotation values for Model B. While Model A also had multiple optima, their number was smaller, and only one optimum occurred within the parameter area where all annotators performed with correctness >0.5.

The multimodal shape of our likelihood functions is a direct consequence of the inability to directly observe or determine the correctness of annotation values. Multimodal likelihood surfaces are a common feature of models involving latent variables (e.g., see [9] ), suggesting that multiple explanations are possible for the same data and each corresponds to a mode on the likelihood surface. Moreover, the larger the number of parameters, the larger the number of possible configurations explaining the same dataset.

One additional advantage of Model A is, when we annotate the same fragment of text along multiple dimensions, Model A can easily be altered to allow for non-independence among distinct types of annotations. (See *Text S1* for details.)

### Simulating data from either model; estimating parameters under both

**Figure 3:**
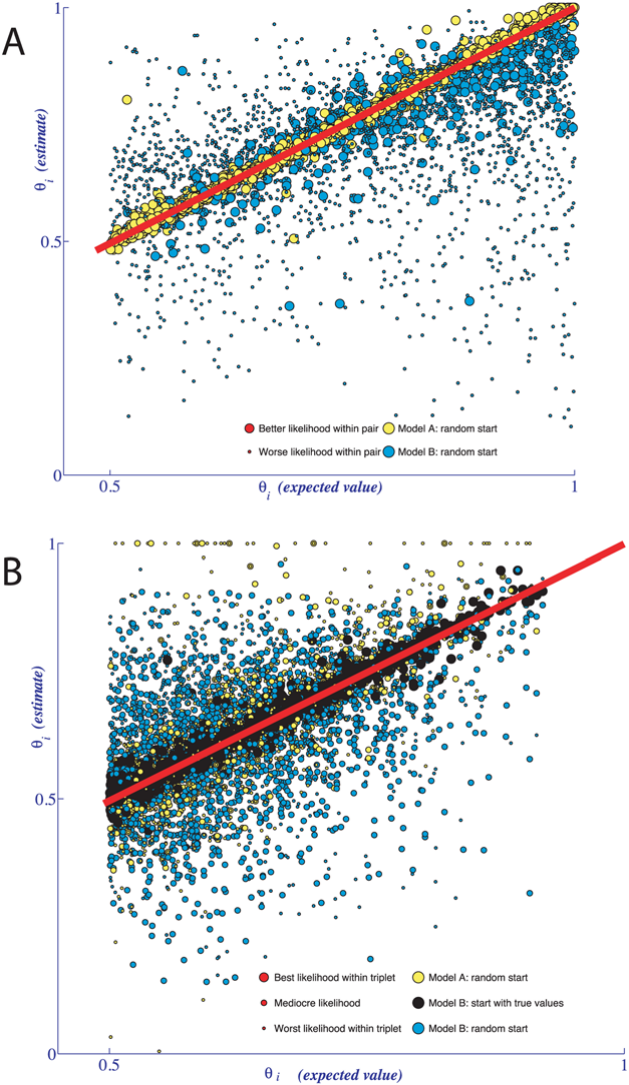
Simulation-estimation experiments assuming Model A (A) and Model B (B). We performed 1,000×2 computational experiments to generate annotation data under Model A (plot A) and under Model B (plot B), *estimating parameters under both models* in each case. Each of the 1,000 iterations per plot involved sampling a new set of the expected parameter values, generating artificial annotations using these expected values, and then estimating parameters from these artificial data. In each simulation iteration we generated 10,000 sets of artificial annotations imitating work of three annotators. Note that although Model B was not defined in terms of annotator-specific correctness parameters, these parameters can be expressed easily as a function of the native parameters of Model B. (A) Simulations under Model A: For each simulated data set we produced two different estimates, one with Model A and one with Model B. Model A estimation, starting with a random set of initial values with correctness parameter values>0.5 each, reliably recovered the correctness parameter values (yellow dots). Estimation under Model B (blue dots) yielded a significantly wider scatter of estimates, most likely because the hill-climbing algorithm used in this estimation got stuck in one of the numerous local optima on the surface of posterior probability under Model B. Each round of parameter estimation produced two sets of three-annotator-specific estimates, resulting in 6 plot data points. (B) Simulations under Model B: For each of the 1,000 simulated data sets we produced a triplet of estimates (random starts under Models A and B, and start under Model B at the expected values of parameters). When started in the global-optimum mode (black dots), estimation of Model B reliably resulted in near-perfect estimates of the correctness parameters, outperforming the estimated parameters for Model A (yellow dots). However, when started with random parameter values for estimating under Model B, the estimates were widely scattered (blue dots), corresponding to the numerous local optima associated with Model B. Each estimation round resulted in three sets of three-annotator-specific estimates, represented as 9 separate data points in the plot.

To test our methodology, before applying it to real annotator data, we conducted a study in which data were simulated from one of the models and then parameters were estimated under both (see Figure 3). When we obtained simulated data from Model A, the parameters estimated for Model A clustered nicely along a perfect diagonal (given that both true values and the initial optimization values of the correctness parameters were >0.5) (yellow circles in Figure 3A). The parameters for Model B produced a much greater scatter of likelihood values, with better likelihood estimates closer to the expected values (blue circles in Figure 3A). This result (along with additional repeated-estimation analysis) indicates that poorer likelihood values for Model B correspond to convergence to the numerous local optima on the B-model likelihood surface. In one example (detailed in the *Text S1*), we made 300 estimates under Model B for the same simulated dataset (3 allowed annotation values). The estimation search ended in the same local optimum only 2 times out of 300; 298 sets of estimates were all distinct from each other.

When simulation was performed by generating data under Model B, the parameters estimated for Model A tended to be more widely scattered than when the data was simulated under Model A (yellow circles in Figure 3B). Nevertheless, Model A estimates still tend to follow the diagonal of the plot. As expected, when estimation for Model B was initialized at the true parameter values, the resulting estimates grouped tightly around the perfect-estimate diagonal (black circles in Figure 3B). However, when estimation under Model B was initialized at a random point in the parameter space, (blue circles in Figure 3B), estimation scatter became extreme due to convergence on local optima.

The simulations indicate that we can indeed obtain reasonable estimates of the annotator correctness parameters, and this was practically easier with Model A. Estimating parameters for Model B was computationally more expensive, requiring the estimation of many more parameters, while frequently settling into local optima. As such, we use Model A and the simplified B-with-thetas to analyze our real annotator data.

### Analysis of real data

**Figure 4:**
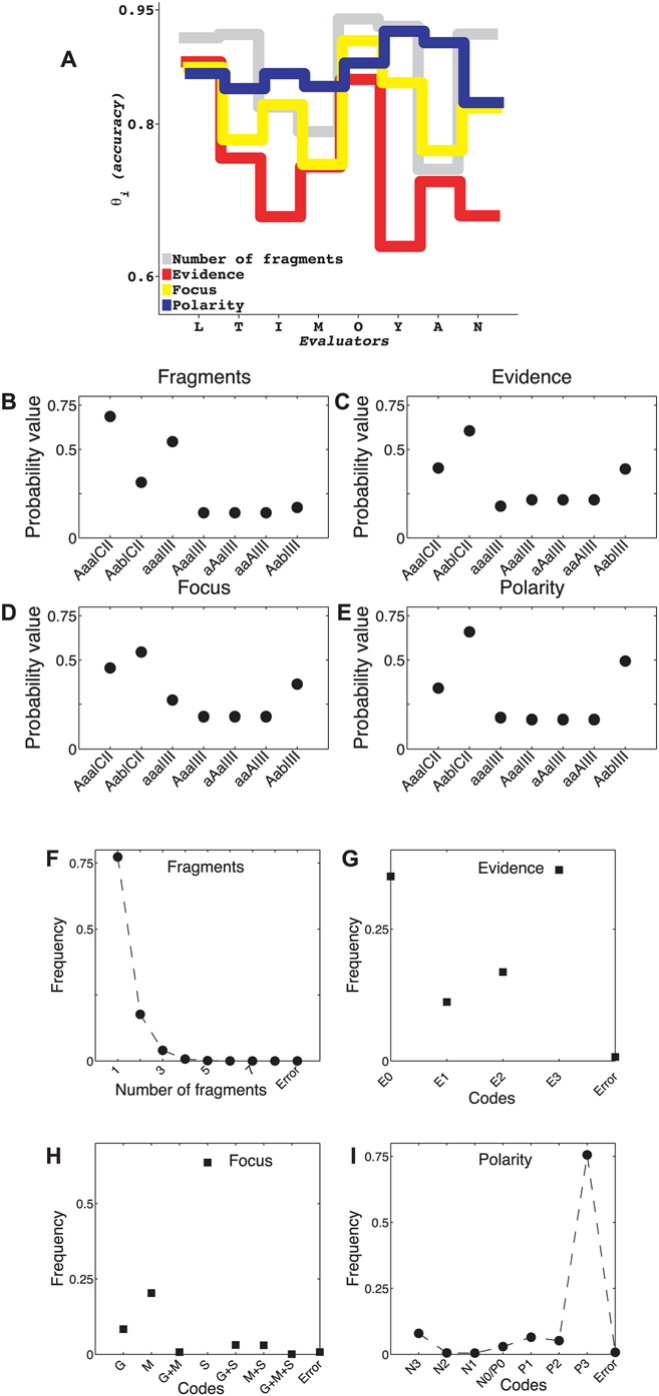
Estimates of parameters defined under Model A from real data. (A) Estimates of correctness parameter values for eight annotators across multiple annotation types. (B–E) Estimates of *α*-parameters (conditional probabilities of agreement patterns given the correctness pattern). (F–I) Estimates of *ω*-values (frequencies of the annotation codes).

Figure 4 shows estimates of key parameters for Model A using approximately 10,000 sentences, each annotated by three annotators. Figure 4A shows maximum likelihood estimates of the correctness parameters for the eight annotators (the first round of evaluation) and four dimensions of annotation.

**Figure 5:**
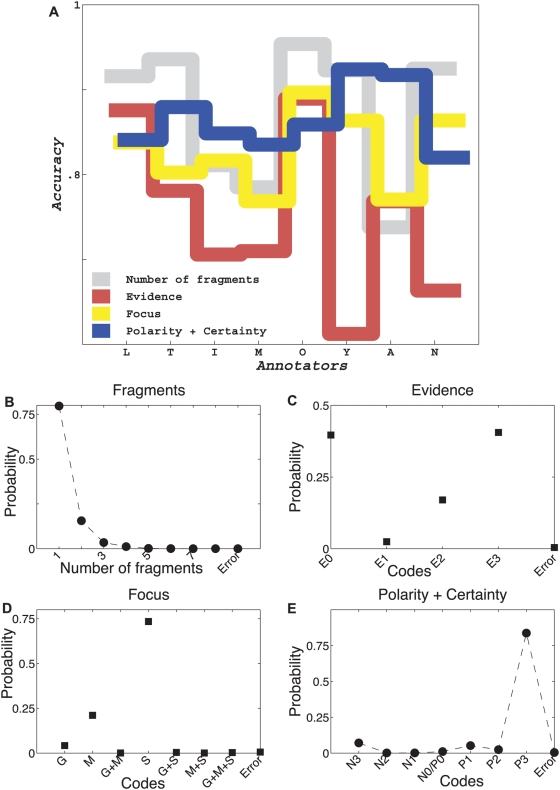
Estimates of parameters defined under Model B-with-thetas from real data. (A) Estimates of correctness parameter values for eight annotators across multiple annotation types. While these values are different from those estimated under Model A, (Figure 4 A), the estimates are clearly consistent across the two models. (B–E) Estimates of *γ*-distributions, where *γ_i_* is the probability that the *i^th^* annotation code is correct. Note that *γ*-distributions are similar but not identical to distributions of *ω*-values shown in Figures 4 (F–I).

Surprisingly, not only did the value of correctness vary significantly *among* annotators, but the same annotator's correctness fluctuated widely across the annotation tasks. The same annotator could perform extremely well at one annotation task and terribly at another (see Figures 4A and 5A for results of analysis under models A and B-with-thetas, respectively). We observed very similar absolute values of correctness and nearly identical patterns of annotator-specific correctness across dimensions under the two models. Thus, it is more likely that the features of our annotator correctness estimates reflect properties of annotator performance rather than being artifacts of model design.

Estimates of conditional probabilities of agreement patterns given correctness status (denoted by *α'*s, see Figure 4B–E) and estimates of code frequencies (denoted by *ω'*s, see Figure 4F–I) also tell an interesting story. As we have noted previously [4], frequencies of annotation values for each dimension were far from uniform: The probability was almost 0.75 that a sentence would be annotated as having a single fragment (Figure 4F). Similarly, there was a greater than 0.60 chance that a fragment would contain either no reference to experimental evidence at all (E0) or direct evidence (E3), but not a value in between (E1 and E2, see Figure 4G); a 0.55 chance that the sentence would be annotated as having scientific focus (Figure 4H); and a greater than 0.75 chance that the fragment would contain the most certain positive statement (Figure 4I). Distributions of the code-frequency values, *ω'*s, were mirrored fairly closely by the annotation correctness distributions (*γ*-distributions), estimated for Model B-with-thetas, Figure 5 (B–E).

The direct consequence of the skewed distribution of annotation codes is that under Model A the probability of random convergence to incorrect annotation values was high. Consider the conditional probabilities of agreement patterns given correctness states for the number of fragments in the sentence (Figure 4B). When all three annotators had incorrect annotations (*III*), the most likely observed agreement pattern was a perfect consensus (*aaa*, Figure 4B). Other dimensions of annotation showed a similar trend (Figure 4C–E). Why are these observations important? Because, depending on the annotation task, relying on annotator consensus annotations can lead to accepting erroneous annotations, while a proper stochastic modeling can rectify the problem.

The online *Text S1* provides all equations required to identify the annotation with the highest posterior probability for each annotated fragment of text.

While there are numerous approaches for comparison of models in terms of their goodness-of-fit to data (e.g. [10] ), we do not apply them in our comparison of models A and B, because comparison of the raw log-likelihood values makes application of more sophisticated approaches unnecessary. Indeed, when we apply both models to our real annotator data, the most complicated version of Model A (namely, *A-with-alphas*) has 11 parameters to resolve the number of sentence fragments while the simplest version of Model B has 16 parameters. The best log-likelihood values we achieved after performing hundreds of independent runs of our random-start likelihood-maximization processes with *A-with-alphas* and *B-with-thetas* were −19,215.544 and −22,897.744, respectively. It is clear, even without any more sophisticated model-selection approaches, that the simple Model A fits the data orders of magnitude better than the more complicated Model B. The simplified Model A (−19,289.269), as expected, does not fit the data as well as its parameter-enriched version, but still significantly better than Model B. Curiously, estimates of annotator-specific accuracies (*θ*-values) are virtually identical under both versions of Model A (data not shown).

That said, it is important to note that comparison of models is never absolute but is always relative to the data on which the models are being compared. In other words, despite our observations on the particular dataset, it is likely that there are datasets on which the performance of the models is reversed.

### Does it work?

The critical question regarding a study like this is whether the suggested approach is actually useful. To compare model-based predictions with external evaluations, we selected a random subset of 1,000 sentences (out of the original 10,000) and recruited five additional independent annotators to provide 5-fold re-annotation of these 1,000 sentences.

The most obvious way to demonstrate the utility of our models would be to re-evaluate predictions for cases where the three original annotators provided three different annotations for the same fragment of text, and compare these annotations to those produced by the additional five independent annotators.

To understand details of the underlying computation, consider the specific task of annotating the number of fragments in a sentence. For example, the original three annotators had estimated accuracies under the simplified Model A (*θ*-values) of 0.91776, 0.91335, and 0.82234, and detected 2, 1, and 4 fragments in the sentence, respectively (3-way disagreement). The correctness values alone suggest that we should trust annotator 1 most and annotator 3 least. We can further quantify our trust by computing the posterior probabilities that each annotator is correct given this particular triplet of annotators and annotation values.

The posterior probabilities (again, under simplified Model A) that the correct number of sentence fragments is 2, 1, and 4 are 0.4223, 0.3989, and 0.1752, respectively. That is, we have more than twice as much confidence that annotator 1 is right than that annotator 3 is right. Furthermore, we have more than four times the confidence that the correct number of fragments is either 1 or 2 as opposed to 4. To check the validity of our prediction, we looked at five additional (independent) annotations for the same sentence: 2, 2, 2, 2, and 1. Combining the original three annotations with the five new ones, we obtained an 8-way annotator vote value: 2. In this case, clearly, the 8-way vote coincided with our maximum *a posteriori* probability (MAP) prediction.

Due to the nature of our annotation protocol, where annotations are assigned to fragments rather than to complete sentences, the validity of *polarity*, *focus*, and *evidence* annotations was confounded by the validity of sentence segmentation (see Table 1 for an example). When annotators disagreed on the number of fragments and, especially on fragment boundaries, our analysis had to deal with small spurious fragments. To clarify, consider the case in which three annotators annotate a hypothetical sentence consisting of just three one-letter words: “*A B C.*” The annotators are allowed to break the sentence into fragments and annotate fragments with one of two codes: 1 or 2. Suppose that annotator 1 broke the sentence into fragments “*A*” and “*BC*”, annotating the first fragment with code 1 and the second with code 2 – for brevity we write this as *A1|BC2*. Similarly, evaluator 2 produced annotation *AB1|C2* – breaking the sentence also into a pair of fragments, but, unlike annotator 1, grouping A and B. The third evaluator did not break the sentence at all, assigning annotation 2 to the whole sentence: *ABC2*. In combining these annotations, in order to enable analysis of the results, we first find the minimal fragmentation that incorporates all breakpoints –in our case, *A*, *B*, and *C*. Then we re-write the original annotations by transferring codes from larger fragments to smaller ones: A1B1C2, A1B2C2, and A2B2C2, for annotators 1, 2, and 3, respectively. As such, we pooled all breakpoints from the annotators to determine the final fragmentation and each of the original annotated fragments propagates its annotation down to all the final fragments composing it. We recognize that in future studies the segmentation and annotation should be performed in two stages. The first stage should focus on annotating boundaries of the fragments and finding the maximum *a posteriori* boundary. The second stage should involve annotation of fragment codes given the MAP sentence fragmentation. Despite this additional noise, our analysis below demonstrates that MAP predictions were significantly enriched with correct answers.

The main difficulty with the practical application of Model B is that even after a large number of numerical optimization runs, starting with different initial values, we had no confidence that we had identified the global optimum of the posterior probability. Nevertheless, we used the set of parameter estimates marked with the best likelihood value and the highest prior probability observed in a set of about 100 independent runs. Our results show that even these imperfect parameter estimates provide surprisingly robust prediction results (see Table 2).

**Table 2:** Comparison of two models, *A* and *B-with-thetas*, in terms of their efficiency of resolving three-way ties among three annotators.

	MAP coincides with the 3+5 majority vote (expected by chance) [*p*-value]		Two highest *a posteriori* estimates coincide with the 3+5 majority vote (expected by chance) [*p*-value]		Total
	*Model A*	*Model B-with-thetas*	*Model A*	*Model B-with-thetas*	
Number of fragments	19 ^***^	22^****^	30^***^	29^**^	31
	(31/3)	(31/3)	(62/3)	(62/3)	
	[0.00096]	[8.8×10^−6^]	[0.00038]	[0.0015]	
Evidence	62	70^**^	114	135^****^	157
	(157/3)	(157/3)	(314/3)	(314/3)	
	[0.1]	[0.0028]	[0.1]	[2.8×10^−7^]	
Focus	56^****^	85^****^	76	99^****^	108
	(108/3)	(108/3)	(216/3)	(216/3)	
	[4.5×10^−5^]	[0]	[0.4]	[3.6×10^−8^]	
Polarity+Certainty	54^****^	52^****^	**56**	52	87
	(29)	(29)	(58)	(58)	
	[1.3×10^−8^]	[1.7×10^−7^]	[0.6]	[0.2]	

To test the models, we compare posterior distributions of correct annotations computed under each model with the majority vote obtained by combining the original 3 annotations with 5 additional annotations. The first pair of columns with numbers indicate matches of the maximum *a posteriori* (MAP) estimate of correct annotation with the 8-evaluator majority vote. The second pair of columns with numbers indicate matches between the two best MAP predictions and the majority vote. Numbers in parentheses indicate the number of matches expected if MAP predictions perform no better than random.

**Note:** * p<0.05; ** p<0.01; *** p<0.001; **** p<0.0001.

For evaluating the quality of our model-specific predictions we need to establish a baseline corresponding to a naïve random-predictor method. If we consider only three-way annotation disagreements, a naïve random-predictor method would work by sampling an annotation out of three choices with a uniform probability (1/3). Similarly, the probability that two annotations out of three include the correct answer (given that one of the three answers is correct) is 2/3. Examining Table 1, we can immediately see that the number of correct MAP predictions under both models was almost invariably greater than the randomly expected number (with the one exception of the two-best-predictions analysis of Polarity—Certainty annotations).

Both models appear to do their prediction job extremely well, with Model B-with-thetas performing marginally better. Despite the relatively small numbers of test cases for each type of annotation (31, 157, 108, and 87 three-way disagreements for distinct annotation types, see Table 2), we observe highly significant deviation from random prediction for each annotation type. The majority of our model-specific *p*-values, computed with Pearson's chi-squared test, are smaller than 10^−3^ and a few are smaller than 10^−7^ and 10^−10^ (see Table 2), indicating the extreme improbability that our prediction success is accidental.

While Model A fits the data better, Model A assumes that annotations are dependent only on the agreement pattern of judges and, given agreement pattern, are conditionally independent of their correctness. We suspect this independence assumption is violated to some extent and this explains Model B's slight advantage in predicting the eight judge results based on the three judge data.

In summary, our method picks the correct prediction (as determined by a larger panel of new additional independent experts) much more frequently than random, proving that our approach offers a practical aid to annotation tasks.

### (Some) implementation details

We performed our probabilistic analysis using programs written in MatLab (MathWorks); all corresponding scripts are available to anyone interested.

For our numerical analysis of posterior probability distributions, we used our own implementation of a simulated annealing algorithm [11], the MatLab implementation of the multidimensional simplex method, and common sense, see *Dataset S*2.

## Discussion

Our analysis above, demonstrates the advantages of careful experimental design, hopefully sufficiently so to convince the biological data curation community regarding the value of an experimental methodology in implementing and analyzing data curation results. It appears that a comparison of curator performance already justifies the effort, but the benefits go well beyond quality control. Our analysis offers the possibility of probabilistic data annotation, where alternative annotations are presented with appropriate degrees of certainty. This represents the plurality of opinions and disagreements among human experts in a much more organic way than does exclusive, deterministic (“crisp”) annotation. Our probabilistic, Bayesian approach to data annotation allows preservation and use of all annotation data, rather than the discarding conflicting parts. Furthermore, probabilistic machine learning methods, such as the maximum entropy and conditional random fields approaches, are well suited for imitating human curators and learning from such annotations.

As is further exemplified in the following section, the methodology described in this paper is directly applicable to a wide spectrum of annotation tasks, such as annotation of large fragments of text (articles, paragraphs, books), nucleotide sequences, phenotypes, three-dimensional models, and raw experiments. One could even use it to compare computational methods, for example, in the computational annotation of genomic regions, or in the detection of copy number variation using expression array data. In these applications, computation-generated predictions take the role of annotators (with unknown accuracies) annotating the same piece of data.

In the spirit of exploring mathematical symmetries [12], we notice that extrema in likelihood optimization under Model B form a permutation group that has *n!* group members for annotation with *n* admissible values. We can show (see *Text S1*) that every mode (solution) that belongs to the same permutation group has exactly the same height (the maximum likelihood value). We exploited this property in our implementation of the Expectation-Maximization algorithm, as explained in the *Text S1*. While each optimum has a corresponding permutation group of equivalent solutions yielding the same probability for the data, the likelihood surface is replete with local optima which are not equivalent and which we cannot currently count or characterize.

Both of the proposed models give rise to multiple solutions for the same data, although Model B is especially rich in alternative modes at the likelihood surface. At first we viewed this property disparagingly. Later, however, we realized a positive aspect of this multiplicity. It is true that a practical minded researcher looks for a *unique* solution to a mathematical problem. However, reality *can* often be explained in multiple ways. We can think of the multiple solutions to a set of equations as merely an invitation to consider alternative logically consistent ways to interpret data. This is not an unprecedented situation: the famous field equations formulated by Albert Einstein [13] allow for numerous solutions; each consistent solution, discovered by different thinkers during the last century, suggested a unique view of the physical world with profound and distinct philosophical implications.

### Applying our modeling to problems of the real world

How can the real-world data curation efforts, such as *Arabidopsis thaliana* annotation [14], Mouse Genome Database [15], UniProt and Swiss-Prot [16], GenBank [17] and numerous other repositories heavily used by bench biologists, benefit from our methodology?

It would be naïve on our part to expect that every curation team in the world will immediately switch to annotating each piece of data three times, using a loop design for multiple annotators (it would be nice, though). However, it is likely to benefit the curation teams to conduct small-scale annotation experiments, estimating error rates specific to the task at hand and to the group of annotator experts. Such estimates can be immediately used to assign confidence to data annotated by a single expert with a known correctness rate. Furthermore, estimates of annotator correctness are useful in conducting randomized quality control checks, where a randomly chosen datum is re-annotated by a group of three annotators with known performance metrics. We further illustrate the applicability of the method in the following example.

### Example of a realistic application

Consider a team of curators at the Jackson Laboratory in Bar Harbor, Maine, working on curating mouse phenotypes for mouse strains with genetic differences within corresponding genomes.

A genome of a given mouse strain can harbor a spectrum of variations relative to the genome of another mouse strain. Mouse phenotypes are arranged into a hierarchical terminology [15],[18] where each term is assigned to a unique code. While in some cases assignment of genetic variation to a phenotype is clear and unambiguous, in others the curators have to resolve some degree of ambiguity of assignment of rearrangement to a specific gene or genes (e.g., when multiple genes are affected) or of genetic variation to a phenotype (e.g., when pleiotropic variation is considered).

We can directly relate such an annotation task to our modeling framework. Suppose that eight curators (1, 2, …, 8) are arranged into eight groups of three experts each: (1, 2, 3), (2, 3, 4), (3, 4, 5), …(7, 8, 1). We ask curators within the same group to assign *discrete phenotypic codes* to the same subset of genetic variations. From the annotated data we can estimate model parameters for Models A and B as described in the paper, and estimate curator-specific error rates.

Such error-rates are immediately useful in order to:

1. provide feedback to the experts;
2. assign confidence values to annotations produced by any subset of the curators, and
3. find the most likely correct annotation in cases of disagreement.

The above example illustrates the applicability and potential utility of the models within the setting of a current and ongoing curation effort.

## Supporting Information

Text S1 Detailed description of our modeling approaches.
(2.01 MB PDF)

Dataset S1 Full dataset produced by the two-round annotation effort described in this study.
(1.79 MB ZIP)

Dataset S2 Complete set of MatLab programs required to estimate model parameters (under both models described here) and to compute the posterior distribution of correct annotation values.
(0.62 MB ZIP)
