# Supplementary material for: How to Get the Most out of Your Curation Effort
Source: PLoS Comput Biol. 2009 May 22;5(5):e1000391. doi: 10.1371/journal.pcbi.1000391 (PMC2678295; doi:10.1371/journal.pcbi.1000391)
Supplement: Text S1 — Detailed description of our modeling approaches. (2.01 MB PDF) [file pcbi.1000391.s001.pdf]

Supplement: In-depth description of models and simulation experiments.

December 24, 2008

Contents

1 The idea of a loop design experiment **4**

2 Grouping evaluators **4**

3 Two generative models: A and B **4**

4 Model A: General framework of our analysis **5**

5 Model A: Parameters **7**

6 Model A: Annotating a single dimension **8**

7 Model A: Annotating multiple dimensions, introducing dependence among dimensions **12**

8 Model A: Numerical data simulation **16**

9 Model B: Description **18**

10 Model B: Counting parameters and observations **21**

11 Model B: Numerical data simulation **21**

12 Model B and the Expectation-Maximization algorithm **25**

13 Symmetries within solutions for model B **29**

List of Tables

1 Model A: Parameters and notations. 6
2 Model A: Probabilities of all possible triplets of correctness values. 10
3 Model A: Conditional probabilities of three-evaluator-agreement patterns, given correctness values. 10
4 Model A: Defining α-parameters: evaluators are assumed identical in terms of frequency of annotation values. Note that these equations are applicable for any number of admissible values for annotations. For example, for only two admissible values we have $[\omega_1, \omega_2, \omega_3, \omega_4, \ldots] = [\omega_1, 1 - \omega_1, 0, 0, \ldots]$, while for only three admissible values we have $[\omega_1, \omega_2, \omega_3, \omega_4, \ldots] = [\omega_1, \omega_2, 1 - \omega_1 - \omega_2, 0, \ldots]$. 10
5 Model A: Defining β's. 11
6 Model A: Joint probabilities of three-evaluator correctness values *and* the observed values of evaluations. 11
7 Model A: $P(\mathbf{V}_{ijk}|\Theta)$. 11
8 Model A: $P(\mathbf{T}_{ijk}|\mathbf{V}_{ijk}, \Theta)$. 12
9 Model A simulation: $M = 3$: expected frequencies of three-evaluator patterns. $\omega_1 = 0.1$, $\omega_2 = 0.3$, $\omega_3 = 0.6$, $\theta_1 = 0.6$, $\theta_2 = 0.8$, and $\theta_3 = 0.5$. Estimates: $\hat{\omega}_1 = 0.0792$, $\hat{\omega}_2 = 0.2966$, and $\hat{\omega}_3 = 0.6242$. 16
10 Model A simulation / model A estimation: Maximum likelihood parameter estimates for simulated data with "true" parameter values $\theta_i = 0.6$, $\theta_j = 0.8$, and $\theta_k = 0.5$, obtained in $1,000$ independent runs of simulated annealing. . 17
11 Model A simulation / model B estimation: Maximum likelihood parameter estimates (under model B) for simulated data with "true" parameter values (generated under model A) $\theta_i = 0.6$, $\theta_j = 0.8$, and $\theta_k = 0.5$, obtained in 300 independent runs of simulated annealing. We show here only three results because only two estimates out of 300 ended in the same local optimum. It appears that model B generates a large number of local extrema. 17
11 Model A simulation / model B estimation: Maximum likelihood parameter estimates (under model B) for simulated data with "true" parameter values (generated under model A) $\theta_i = 0.6$, $\theta_j = 0.8$, and $\theta_k = 0.5$, obtained in 300 independent runs of simulated annealing. We show here only three results because only two estimates out of 300 ended in the same local optimum. It appears that model B generates a large number of local extrema. 18
12 Model B: Parameters and notations. 18
13 Model B simulation: $M = 2$: expected frequencies of three-evaluator patterns. 21
14 Model B simulation / model B estimation: Example 1, $M = 2$, 40 independent runs of simulated annealing estimation (same data, different starting values of parameters). The "true" value for each parameter is shown in brackets. To perform the estimation, we first generated 10,000 "data points": $N =$[1882, 443, 1863, 506, 1376, 1152, 1412, 1366] for annotation patterns [111, 112, 121, 122, 211, 212, 221, 222], respectively. We can clearly see that the likelihood surface has two modes of exactly the same height (the less frequent mode is shown in bold). 22

15 Model B simulation / model B estimation: Example 2, $M = 2$, 20 independent runs of simulated annealing estimation (same data, different starting values of parameters). The "true" value for each parameter is shown in brackets. To perform the estimation, we first generated 10,000 "data points": $N =$[564, 338, 1752, 867, 681, 342, 3730, 1726] for annotation patterns [111, 112, 121, 122, 211, 212, 221, 222], respectively. We can clearly see that the likelihood surface has two modes of exactly the same height (the less frequent mode is shown in bold). $[\theta_i, \theta_j, \theta_k] = [0.68, 0.83, 0.36]$, where $\theta_x = \sum_\psi \gamma_\psi \lambda_{x,\psi|\psi}$. . . . 23

16 Model B simulation / model A estimation: Example 2, $M = 2$, 50 independent runs of simulated annealing estimation (same data, different starting values of parameters). The "true" value for each parameter is shown in brackets. To perform the estimation, we first generated 10,000 "data points": $N =$[564, 338, 1752, 867, 681, 342, 3730, 1726] for annotation patterns [111, 112, 121, 122, 211, 212, 221, 222], respectively. We can clearly see that the likelihood surface has two modes. $[\theta_i, \theta_j, \theta_k] = [0.680.830.36]$, where $\theta_x = \sum_\psi \gamma_\psi \lambda_{x,\psi|\psi}$. . 24

16 Model B simulation / model A estimation: Example 2, $M = 2$, 50 independent runs of simulated annealing estimation (same data, different starting values of parameters). The "true" value for each parameter is shown in brackets. To perform the estimation, we first generated 10,000 "data points": $N =$[564, 338, 1752, 867, 681, 342, 3730, 1726] for annotation patterns [111, 112, 121, 122, 211, 212, 221, 222], respectively. We can clearly see that the likelihood surface has two modes. $[\theta_i, \theta_j, \theta_k] = [0.680.830.36]$, where $\theta_x = \sum_\psi \gamma_\psi \lambda_{x,\psi|\psi}$. . 25

1 The idea of a loop design experiment

The idea of a *loop design* experiment can be traced to Sir Ronald Aylmer Fisher [1]—it is exposed in detail in numerous recent books, for example, [2, 3].

2 Grouping evaluators

A *loop design* involves partitioning evaluators into several groups (in our case, with three people each) and assigning them approximately equal numbers of sentences. In addition, attributes of the sentences (as described below) should be randomly distributed across all groups.

Eight different evaluators (numbered 1 to 8)
Three evaluators work on each sentence

$$\text{Groups} \quad \left\{ \begin{array}{ccc} \multicolumn{3}{c}{\overbrace{\hphantom{1 \quad 2 \quad 3}}^{\textit{Evaluators in the same group}}} \\ 1 & 2 & 3 \\ 2 & 3 & 4 \\ 3 & 4 & 5 \\ 4 & 5 & 6 \\ 5 & 6 & 7 \\ 6 & 7 & 8 \\ 7 & 8 & 1 \\ 8 & 1 & 2 \end{array} \right. \tag{1}$$

3 Two generative models: A and B

For historical reasons, we explored in depth two probabilistic models which describe a single annotation dimension.

Model A is slightly more complicated to describe, but has a smaller number of parameters (one parameter per annotator per dimension) and allows for easy extension to joint analysis of multiple dimensions.

Model B is simpler to describe and is somewhat more intuitive, but the number of parameters associated with it grows quadratically with the number of admissible annotation values. For example, with combined Polarity–Certainty dimensions (admissible values N3, N2, N1, N0 or P0, P1, P2, and P3), we need to estimate 8 parameters under model A and 342 parameters under model B.

Furthermore, both models generate multiple solutions (modes along the likelihood surface). With appropriately chosen priors, model A has two modes, only one of which is the global

maximum. Model B has multiple modes—the number of modes is growing with the number of admissible values for annotation.

Below we describe in detail both models and illustrate them with numerical examples.

4 Model A: General framework of our analysis

While we develop a framework for describing multiple annotation dimensions jointly, for compactness of presentation, we describe equations for a single dimension and a single annotation instance (the same equations would work for a group of annotations after each triplet of variables is substituted with the corresponding matrix).

To estimate parameters of the model with the maximum likelihood (ML) or the maximum *a posteriori* probability (MAP) methods, we need to maximize the appropriate probability functions. For example, the likelihood function in our case is defined in the following way:

$$P(\mathbf{V}_{ijk}|\Theta) = \sum_{\mathbf{T}_{ijk}} \sum_{\mathbf{A}_{ijk}} P(\mathbf{V}_{ijk}|\mathbf{A}_{ijk}, \Theta) P(\mathbf{A}_{ijk}|\mathbf{T}_{ijk}, \Theta) P(\mathbf{T}_{ijk}|\Theta), \tag{2}$$

Then we can compute

$$\hat{\Theta}^{ML} = \arg\max_{\Theta} P(\mathbf{V}_{ijk}|\Theta), \tag{3}$$

or

$$\hat{\Theta}^{MAP} = \arg\max_{\Theta} P(\Theta|\mathbf{V}_{ijk}) = \arg\max_{\Theta} \frac{P(\mathbf{V}_{ijk}|\Theta)P(\Theta)}{P(\mathbf{V}_{ijk})} = \arg\max_{\Theta} P(\mathbf{V}_{ijk}|\Theta)P(\Theta). \tag{4}$$

The advantage of using Equation 4 instead of Equation 3 is that we can specify a non-uniform prior distribution for Θ. For example, we can assume that our accuracy parameters are more likely to have high values (greater than 0.5) than smaller values. This can be expressed with a beta-distribution:

$$P(\theta_m^x = \xi|a, b) = \frac{\xi^{a-1}(1-\xi)^{(b-1)}}{B(a, b)}, \tag{5}$$

where m refers to the mth annotator, x refers to the xth dimension of annotation, $a = 2$, $b = 1$, and $B(a, b)$ is a beta-function with parameters a and b. This way we incorporate an assumption that human evaluators are more frequently right than wrong in their assessments.

The main reason we want to estimate parameter values, $\hat{\Theta}$, is to find the most likely assignment of correctness labels to observed annotations:

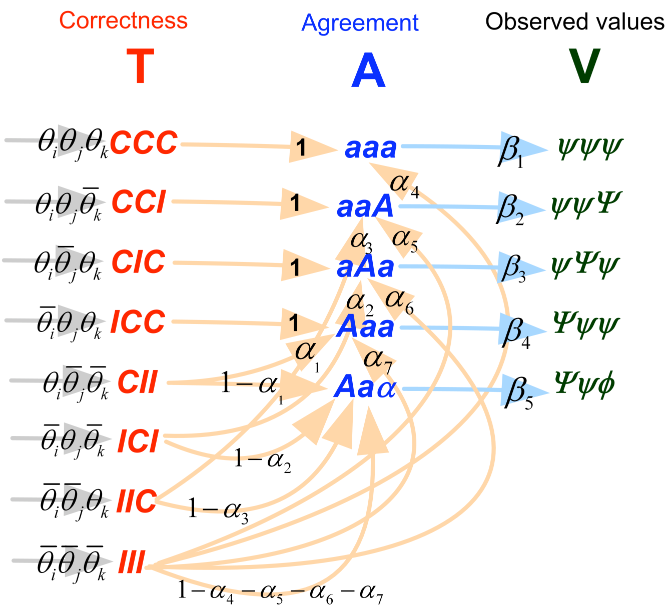

Figure 1: Model A: Graphical-model-style outline of the correct-value-specific annotation error model (one annotation dimension, three evaluators are annotating the same fragment of text).

$$\hat{\mathbf{T}}_{ijk}^{MAP} = \arg\max_{\mathbf{T}_{ijk}} P(\mathbf{T}_{ijk}|\mathbf{V}_{ijk}, \Theta). \tag{6}$$

The value of $P(\mathbf{T}_{ijk}|\mathbf{V}_{ijk}, \Theta)$ is computed in the following way

$$P(\mathbf{T}_{ijk}|\mathbf{V}_{ijk}, \Theta) = \frac{P(\mathbf{V}_{ijk}, \mathbf{T}_{ijk}|\Theta)}{P(\mathbf{V}_{ijk}|\Theta)} \tag{7}$$

where

$$P(\mathbf{V}_{ijk}, \mathbf{T}_{ijk}|\Theta) = \sum_{\mathbf{A}_{ijk}} P(\mathbf{V}_{ijk}|\mathbf{A}_{ijk}, \Theta) P(\mathbf{A}_{ijk}|\mathbf{T}_{ijk}, \Theta) P(\mathbf{T}_{ijk}|\Theta). \tag{8}$$

In equations 2 and 8 we implicitly assume that $\mathbf{V}_{ijk}$ and $\mathbf{T}_{ijk}$ are conditionally independent, given the known value of $\mathbf{A}_{ijk}$.

Table 1: Model A: Parameters and notations.

Notation		*Explanation*
T_i^z	$\longrightarrow$	Correctness value (hidden variable) of annotation provided by the ith evaluator for z^{th} dimension for a given text fragment.
$T_i^z = C$	$\longrightarrow$	Correct annotation.
$T_i^z = I$	$\longrightarrow$	Incorrect annotation.
θ_i^z	$\longrightarrow$	$P(T_i^z = C|\Theta)$
$\bar{\theta}_i^z$	$\longrightarrow$	$1 - P(T_i^z = C|\Theta) = P(T_i^z = I|\Theta)$

ω_ψ	$\longrightarrow$	Probability to encounter annotation value ψ (assumed to be the same for all evaluators).
$\beta_x^{(v_i,v_j,v_k)}$	$\longrightarrow$	Probability of a given triplet of annotations for three evaluators ($[v_i, v_j, v_k]$)), given one of the agreement patterns. A more precise definition of these parameters is given in Table 5.
α_y	$\longrightarrow$	Probability of an agreement pattern, given the correctness pattern value. A more precise definition of these parameters is given in Table 4.
$\mathbf{T}^z_{ijk} = (T^z_i, T^z_j, T^z_k)$	$\longrightarrow$	State of correctness values for annotations for the zth dimension and the same text fragment as provided by annotators i, j, and k.
$\mathbf{A}^z_{ijk} = (A^z_i, A^z_j, A^z_k)$	$\longrightarrow$	A three-annotator agreement pattern for the zth dimension.
$\mathbf{A}^z_{ijk} = (aaa)$	$\longrightarrow$	The three annotators agree on annotation (regardless the annotation value).
$\mathbf{A}^z_{ijk} = (aaA)$	$\longrightarrow$	Annotator k disagrees with other two annotators.
$\mathbf{A}^z_{ijk} = (aAa)$	$\longrightarrow$	Annotator j disagrees with other two annotators.
$\mathbf{A}^z_{ijk} = (Aaa)$	$\longrightarrow$	Annotator i disagrees with other two annotators.
$\mathbf{A}^z_{ijk} = (Aa\alpha)$	$\longrightarrow$	All three annotation values are different (regardless of the values).
$\mathbf{V}^z_{ijk} = (v^z_i, v^z_j, v^z_k)$	$\longrightarrow$	The actual annotation values for the zth dimension of a text fragment assigned by evaluators i, j, and k.
ρ^{xy}	$\longrightarrow$	Correlation between annotations for dimensions x and y.
Θ	$\longrightarrow$	A shorthand for all model parameters.

5 Model A: Parameters

Evaluator- and dimension-specific success [$= 1 - error$] rates.

$$Evaluators \left\{ \overbrace{\begin{matrix} \theta_1^1 & \theta_1^2 & \dots & \theta_1^n \\ \dots & \dots & \dots & \dots \\ \theta_8^1 & \theta_8^2 & \dots & \theta_8^n \end{matrix}}^{Annotation\ dimensions} \right. \tag{9}$$

The second-order correlation matrix:

$$Annotation\ dimensions \left\{ \overbrace{\begin{matrix} 1 & \rho_{1,2} & \dots & \rho_{1,n} \\ \rho_{1,2} & 1 & \dots & \rho_{2,n} \\ \dots & \dots & \dots & \dots \\ \rho_{1,n} & \rho_{2,n} & \dots & 1 \end{matrix}}^{Annotation\ dimensions} \right. \tag{10}$$

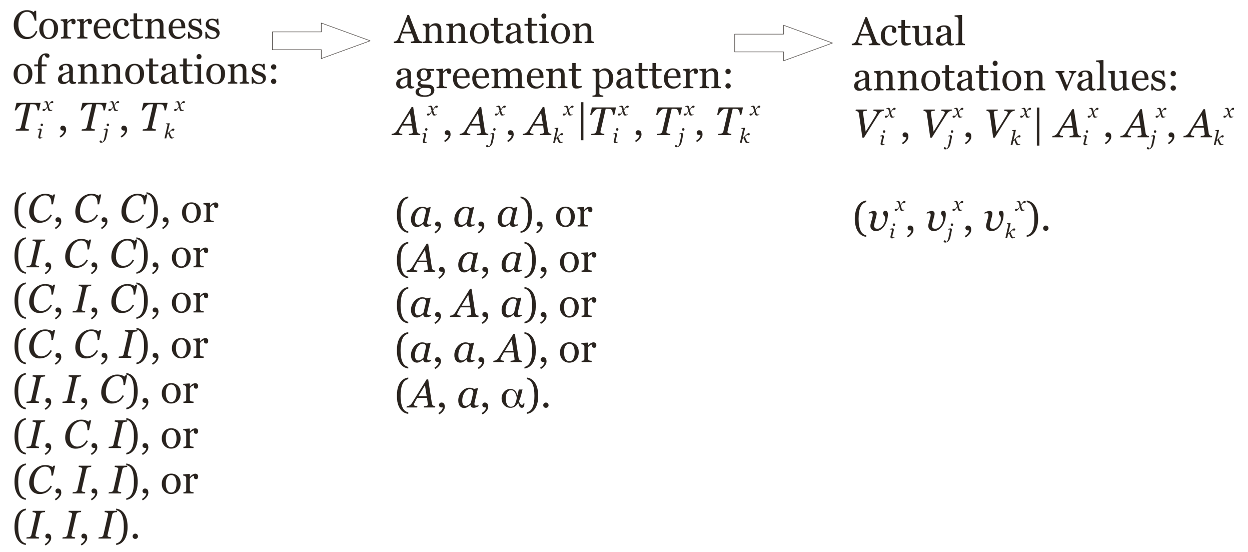

Figure 2: Model A: Outline of the generative model for one dimension of annotation: three evaluators are annotating the same fragment of text (evaluators are indicated by subscripts i, j, and k).

Additional set of parameters (ω_ψ^x) is associated with the expected frequencies of the observed annotation values:

$$P(V_x = \psi | \Theta) = \omega_\psi^x. \tag{11}$$

(We consider here two flavors of the model. In one of them $\omega_\psi^x = \omega_\psi$, that is, the expected frequencies are the same for all evaluators. In the more complicated model each evaluator is provided with an individual set of ω_ψ^x values, where x refers to the evaluator.)

6 Model A: Annotating a single dimension

Let us start with the simplest case: three evaluators (i, j, and k) are annotating a single property (such as *focus*) of a sentence fragment (the dimension has more than three admissible values).

$$P(\mathbf{T}_{ijk} = \{C, C, C\} | \Theta) = \theta_i \theta_j \theta_k. \tag{12}$$

$$P(\mathbf{T}_{ijk} = \{I, I, I\} | \Theta) = \bar{\theta}_i \bar{\theta}_j \bar{\theta}_k. \tag{13}$$

The complete listing of values for $P(\mathbf{T}_{ijk} | \Theta)$ is shown in Table 2.

$$P(\mathbf{A}_{ijk} = \{a,a,a\} | \mathbf{T}_{ijk} = \{T_i, T_j, T_k\}, \Theta) = \begin{cases} 1 & \text{if } T_i = T_j = T_k = C, \\ \alpha_4 & \text{if } T_i = T_j = T_k = I, \\ 0 & \text{otherwise.} \end{cases} \quad (14)$$

We provide the complete listing of non-zero values of $P(\mathbf{A}_{ijk}|\mathbf{T}_{ijk}, \Theta)$ in Table 3; two alternative ways of defining parameters α_ms are shown in Table 4.

$$P(\mathbf{V}_{ijk} = \{v_i, v_j, v_k\} | \mathbf{A}_{ijk} = \{a,a,a\}, \Theta) = \begin{cases} \beta_1^{(\psi)} & \text{if } v_i = v_j = v_k = \psi, \\ 0 & \text{otherwise.} \end{cases} \quad (15)$$

Our notations for $P(\mathbf{V}_{ijk}|\mathbf{A}_{ijk}, \Theta)$'s are shown in Figure 1 (they have values β_1, β_2, β_3, β_4, and β_5); two alternative ways of defining parameters β_ms are shown in Table 5.

Therefore,

$$P(\mathbf{V}_{ijk} = \{\psi, \psi, \psi\} | \Theta) = [\theta_i \theta_j \theta_k + \bar{\theta}_i \bar{\theta}_j \bar{\theta}_k \alpha_4] \beta_1^{(\psi)}. \quad (16)$$

Similarly,

$$P(\mathbf{V}_{ijk} = \{\psi, \psi, \Psi\} | \Theta) = [\theta_i \theta_j \bar{\theta}_k + \bar{\theta}_i \bar{\theta}_j \theta_k \alpha_3 + \bar{\theta}_i \bar{\theta}_j \bar{\theta}_k \alpha_5] \beta_2^{(\Psi,\psi)}, \quad (17)$$

$$P(\mathbf{V}_{ijk} = \{\psi, \Psi, \psi\} | \Theta) = [\theta_i \bar{\theta}_j \theta_k + \bar{\theta}_i \theta_j \bar{\theta}_k \alpha_2 + \bar{\theta}_i \bar{\theta}_j \bar{\theta}_k \alpha_6] \beta_3^{(\Psi,\psi)}, \quad (18)$$

$$P(\mathbf{V}_{ijk} = \{\Psi, \psi, \psi\} | \Theta) = [\bar{\theta}_i \theta_j \theta_k + \theta_i \bar{\theta}_j \bar{\theta}_k \alpha_1 + \bar{\theta}_i \bar{\theta}_j \bar{\theta}_k \alpha_7] \beta_4^{(\Psi,\psi)}, \quad (19)$$

and

$$\begin{aligned} P(\mathbf{V}_{ijk} = \{\Psi, \psi, \phi\} | \Theta) &= [\bar{\theta}_i \bar{\theta}_j \bar{\theta}_k (1 - \sum_{i=4}^{7} \alpha_i) + \theta_i \bar{\theta}_j \bar{\theta}_k (1 - \alpha_1) \\ &+ \bar{\theta}_i \theta_j \bar{\theta}_k (1 - \alpha_2) + \bar{\theta}_i \bar{\theta}_j \theta_k (1 - \alpha_3)] \beta_5^{(\Psi,\psi,\phi)}. \end{aligned} \quad (20)$$

A full listing of non-zero values for $P(\mathbf{V}_{ijk}|\Theta)$ is shown in Table 7 for annotation dimensions with more than three, exactly three, and exactly two admissible values.

Finally, we show values of $P(\mathbf{T}_{ijk}|\mathbf{V}_{ijk}, \Theta)$ in Table 8. Curiously, $P(\mathbf{T}_{ijk}|\mathbf{V}_{ijk}, \Theta)$ does not depend on β_m's.

Table 2: Model A: Probabilities of all possible triplets of correctness values.

	$\mathbf{T}_{ijk}$							
	CCC	CCI	CIC	ICC	CII	ICI	IIC	III
$P(\mathbf{T}_{ijk}\|\Theta)$	$\theta_i\theta_j\theta_k$	$\theta_i\theta_j\bar{\theta}_k$	$\theta_i\bar{\theta}_j\theta_k$	$\bar{\theta}_i\theta_j\theta_k$	$\theta_i\bar{\theta}_j\bar{\theta}_k$	$\bar{\theta}_i\theta_j\bar{\theta}_k$	$\bar{\theta}_i\bar{\theta}_j\theta_k$	$\bar{\theta}_i\bar{\theta}_j\bar{\theta}_k$

Table 3: Model A: Conditional probabilities of three-evaluator-agreement patterns, given correctness values.

$P(\mathbf{A}_{ijk}\|\mathbf{T}_{ijk},\Theta)$		$\mathbf{A}_{ijk}$				
		aaa	aaA	aAa	Aaa	$Aa\alpha$
$\mathbf{T}_{ijk}$	CCC	1	0	0	0	0
	CCI	0	1	0	0	0
	CIC	0	0	1	0	0
	ICC	0	0	0	1	0
	CII	0	0	0	α_1	$1-\alpha_1$
	ICI	0	0	α_2	0	$1-\alpha_2$
	IIC	0	α_3	0	0	$1-\alpha_3$
	III	α_4	α_5	α_6	α_7	$1-\sum_{i=4}^{7}\alpha_i$

Table 4: Model A: Defining α-parameters: evaluators are assumed identical in terms of frequency of annotation values. Note that these equations are applicable for any number of admissible values for annotations. For example, for only two admissible values we have $[\omega_1,\omega_2,\omega_3,\omega_4,\ldots]=[\omega_1,1-\omega_1,0,0,\ldots]$, while for only three admissible values we have $[\omega_1,\omega_2,\omega_3,\omega_4,\ldots]=[\omega_1,\omega_2,1-\omega_1-\omega_2,0,\ldots]$.

α_m	$\mathbf{A}_{ijk}\|\mathbf{T}_{ijk}$	$P(\mathbf{A}_{ijk}\|\mathbf{T}_{ijk},\Theta)$
$\alpha_1=\alpha_2=\alpha_3$	$Aaa\|CII$<br>$aAa\|ICI$<br>$aaA\|IIC$	$\sum_\Psi P(\Psi\ \texttt{is correct})P(A_j=A_k\neq A_i, A_i=\Psi\|\Psi\ \texttt{is correct})$<br>$=\sum_\Psi \omega_\Psi \frac{\sum_{z\neq\Psi}\omega_z^2}{\sum_{x\neq\Psi}\sum_{y\neq\Psi}\omega_x\omega_y}$
α_4	$aaa\|III$	$\sum_\Psi P(\Psi\ \texttt{is correct})P(A_i=A_j=A_k\neq\Psi\|\Psi\ \texttt{is correct})$<br>$=\sum_\Psi \omega_\Psi \frac{\sum_{r\neq\Psi}\omega_r^3}{\sum_{x\neq\Psi}\sum_{y\neq\Psi}\sum_{z\neq\Psi}\omega_x\omega_y\omega_z}$
$\alpha_5=\alpha_6=\alpha_7$	$Aaa\|III$<br>$aAa\|III$<br>$aaA\|III$	$\sum_\Psi P(\Psi\ \texttt{is correct})P(A_i=A_j\neq A_k, A_i\neq\Psi, A_k\neq\Psi\|\Psi\ \texttt{is correct})$<br>$=\sum_\Psi \omega_\Psi \frac{\sum_{\psi\neq\phi,\psi\neq\Psi,\phi\neq\Psi}\omega_\psi\omega_\phi^2}{\sum_{x\neq\Psi}\sum_{y\neq\Psi}\sum_{z\neq\Psi}\omega_x\omega_y\omega_z}$

Table 5: Model A: Defining β's.

$\beta_m^{(v_i,v_j,v_k)}$	$\mathbf{V}_{ijk} \vert \mathbf{A}_{ijk}$	$P(\mathbf{V}_{ijk} \vert \mathbf{A}_{ijk}, \Theta)$
$\beta_1^{(\psi)}$	$\psi\psi\psi \vert aaa$	$\frac{\omega_\psi^3}{\sum_\xi \omega_\xi^3}$
$\beta_2^{(\psi,\Psi)}, \psi \neq \Psi$	$\psi\psi\Psi \vert aaA$	$\frac{\omega_\psi^2 \omega_\Psi}{\sum_{\xi\neq\tau} \omega_\xi^2 \omega_\tau}$
$\beta_3^{(\psi,\Psi)}, \psi \neq \Psi$	$\psi\Psi\psi \vert aAa$	$\frac{\omega_\psi^2 \omega_\Psi}{\sum_{\xi\neq\tau} \omega_\xi^2 \omega_\tau}$
$\beta_4^{(\psi,\Psi)}, \psi \neq \Psi$	$\Psi\psi\psi \vert Aaa$	$\frac{\omega_\psi^2 \omega_\Psi}{\sum_{\xi\neq\tau} \omega_\xi^2 \omega_\tau}$
$\beta_5^{(\psi,\Psi,\phi)}, \psi \neq \Psi, \psi \neq \phi, \phi \neq \Psi$	$\Psi\psi\phi \vert Aa\alpha$	$\frac{\omega_\psi \omega_\Psi \omega_\phi}{\sum_{\xi\neq\tau\neq\mu} \omega_\xi \omega_\tau \omega_\mu}$

Table 6: Model A: Joint probabilities of three-evaluator correctness values *and* the observed values of evaluations.

$P(\mathbf{V}_{ijk}, \mathbf{T}_{ijk} \vert \Theta)$		$\mathbf{V}_{ijk}$				
		$\psi\psi\psi$	$\psi\psi\Psi$	$\psi\Psi\psi$	$\Psi\psi\psi$	$\psi\Psi\phi$
	CCC	$\beta_1^{(\psi)}\theta_i\theta_j\theta_k$	0	0	0	0
	CCI	0	$\beta_2^{(\psi,\Psi)}\theta_i\theta_j\bar{\theta}_k$	0	0	0
	CIC	0	0	$\beta_3^{(\psi,\Psi)}\theta_i\bar{\theta}_j\theta_k$	0	0
	ICC	0	0	0	$\beta_4^{(\psi,\Psi)}\bar{\theta}_i\theta_j\theta_k$	0
$\mathbf{T}_{ijk}$	CII	0	0	0	$\alpha_1\beta_4^{(\psi,\Psi)}\theta_i\bar{\theta}_j\bar{\theta}_k$	$(1-\alpha_1)\beta_5^{(\psi,\Psi,\phi)}\theta_i\bar{\theta}_j\bar{\theta}_k$
	ICI	0	0	$\alpha_2\beta_3^{(\psi,\Psi)}\bar{\theta}_i\theta_j\bar{\theta}_k$	0	$(1-\alpha_2)\beta_5^{(\psi,\Psi,\phi)}\bar{\theta}_i\theta_j\bar{\theta}_k$
	IIC	0	$\alpha_3\beta_2^{(\psi,\Psi)}\bar{\theta}_i\bar{\theta}_j\theta_k$	0	0	$(1-\alpha_3)\beta_5^{(\psi,\Psi,\phi)}\bar{\theta}_i\bar{\theta}_j\theta_k$
	III	$\alpha_4\beta_1^{(\psi)}\bar{\theta}_i\bar{\theta}_j\bar{\theta}_k$	$\alpha_5\beta_2^{(\psi,\Psi)}\bar{\theta}_i\bar{\theta}_j\bar{\theta}_k$	$\alpha_6\beta_3^{(\psi,\Psi)}\bar{\theta}_i\bar{\theta}_j\bar{\theta}_k$	$\alpha_7\beta_4^{(\psi,\Psi)}\bar{\theta}_i\bar{\theta}_j\bar{\theta}_k$	$(1-\sum_{i=4}^7 \alpha_i)\beta_5^{(\psi,\Psi,\phi)}\bar{\theta}_i\bar{\theta}_j\bar{\theta}_k$

Table 7: Model A: $P(\mathbf{V}_{ijk} \vert \Theta)$.

$\mathbf{V}_{ijk}$	$P(\mathbf{V}_{ijk} \vert \Theta)$
$\psi\psi\psi$	$[\theta_i\theta_j\theta_k + \bar{\theta}_i\bar{\theta}_j\bar{\theta}_k\alpha_4]\beta_1^{(\psi)}$
$\psi\psi\Psi$	$[\theta_i\theta_j\bar{\theta}_k + \bar{\theta}_i\bar{\theta}_j\theta_k\alpha_3 + \bar{\theta}_i\bar{\theta}_j\bar{\theta}_k\alpha_5]\beta_2^{(\psi,\Psi)}$
$\psi\Psi\psi$	$[\theta_i\bar{\theta}_j\theta_k + \bar{\theta}_i\theta_j\bar{\theta}_k\alpha_2 + \bar{\theta}_i\bar{\theta}_j\bar{\theta}_k\alpha_6]\beta_3^{(\psi,\Psi)}$
$\Psi\psi\psi$	$[\bar{\theta}_i\theta_j\theta_k + \theta_i\bar{\theta}_j\bar{\theta}_k\alpha_1 + \bar{\theta}_i\bar{\theta}_j\bar{\theta}_k\alpha_7]\beta_4^{(\psi,\Psi)}$
$\Psi\psi\phi$	$[\bar{\theta}_i\bar{\theta}_j\bar{\theta}_k(1-\sum_{i=4}^7 \alpha_i) + \theta_i\bar{\theta}_j\bar{\theta}_k(1-\alpha_1) + \bar{\theta}_i\theta_j\bar{\theta}_k(1-\alpha_2) + \bar{\theta}_i\bar{\theta}_j\theta_k(1-\alpha_3)]\beta_5^{(\psi,\Psi,\phi)}$

Table 8: Model A: $P(\mathbf{T}_{ijk}|\mathbf{V}_{ijk},\Theta)$.

$\mathbf{T}_{ijk}\vert\mathbf{V}_{ijk}$	$P(\mathbf{T}_{ijk}\vert\mathbf{V}_{ijk},\Theta)$
$CCC\vert\psi\psi\psi$	$\frac{\theta_i\theta_j\theta_k}{\theta_i\theta_j\theta_k+\bar{\theta}_i\bar{\theta}_j\bar{\theta}_k\alpha_4}$
$III\vert\psi\psi\psi$	$\frac{\alpha_4\bar{\theta}_i\bar{\theta}_j\bar{\theta}_k}{\theta_i\theta_j\theta_k+\bar{\theta}_i\bar{\theta}_j\bar{\theta}_k\alpha_4}$
$CCI\vert\psi\psi\Psi$	$\frac{\theta_i\theta_j\bar{\theta}_k}{\theta_i\theta_j\bar{\theta}_k+\bar{\theta}_i\bar{\theta}_j\theta_k\alpha_3+\bar{\theta}_i\bar{\theta}_j\bar{\theta}_k\alpha_5}$
$IIC\vert\psi\psi\Psi$	$\frac{\alpha_3\bar{\theta}_i\bar{\theta}_j\theta_k}{\theta_i\theta_j\bar{\theta}_k+\bar{\theta}_i\bar{\theta}_j\theta_k\alpha_3+\bar{\theta}_i\bar{\theta}_j\bar{\theta}_k\alpha_5}$
$III\vert\psi\psi\Psi$	$\frac{\alpha_5\bar{\theta}_i\bar{\theta}_j\bar{\theta}_k}{\theta_i\theta_j\bar{\theta}_k+\bar{\theta}_i\bar{\theta}_j\theta_k\alpha_3+\bar{\theta}_i\bar{\theta}_j\bar{\theta}_k\alpha_5}$
$CIC\vert\psi\Psi\psi$	$\frac{\theta_i\bar{\theta}_j\theta_k}{\theta_i\bar{\theta}_j\theta_k+\bar{\theta}_i\theta_j\bar{\theta}_k\alpha_2+\bar{\theta}_i\bar{\theta}_j\bar{\theta}_k\alpha_6}$
$ICI\vert\psi\Psi\psi$	$\frac{\alpha_2\bar{\theta}_i\theta_j\bar{\theta}_k}{\theta_i\bar{\theta}_j\theta_k+\bar{\theta}_i\theta_j\bar{\theta}_k\alpha_2+\bar{\theta}_i\bar{\theta}_j\bar{\theta}_k\alpha_6}$
$III\vert\psi\Psi\psi$	$\frac{\alpha_6\bar{\theta}_i\bar{\theta}_j\bar{\theta}_k}{\theta_i\bar{\theta}_j\theta_k+\bar{\theta}_i\theta_j\bar{\theta}_k\alpha_2+\bar{\theta}_i\bar{\theta}_j\bar{\theta}_k\alpha_6}$
$ICC\vert\Psi\psi\psi$	$\frac{\bar{\theta}_i\theta_j\theta_k}{\bar{\theta}_i\theta_j\theta_k+\theta_i\bar{\theta}_j\bar{\theta}_k\alpha_1+\bar{\theta}_i\bar{\theta}_j\bar{\theta}_k\alpha_7}$
$CII\vert\Psi\psi\psi$	$\frac{\alpha_1\theta_i\bar{\theta}_j\bar{\theta}_k}{\bar{\theta}_i\theta_j\theta_k+\theta_i\bar{\theta}_j\bar{\theta}_k\alpha_1+\bar{\theta}_i\bar{\theta}_j\bar{\theta}_k\alpha_7}$
$III\vert\Psi\psi\psi$	$\frac{\alpha_7\bar{\theta}_i\bar{\theta}_j\bar{\theta}_k}{\bar{\theta}_i\theta_j\theta_k+\theta_i\bar{\theta}_j\bar{\theta}_k\alpha_1+\bar{\theta}_i\bar{\theta}_j\bar{\theta}_k\alpha_7}$
$CII\vert\Psi\psi\phi$	$\frac{(1-\alpha_1)\theta_i\bar{\theta}_j\bar{\theta}_k}{\bar{\theta}_i\bar{\theta}_j\bar{\theta}_k(1-\sum_{i=4}^7\alpha_i)+\theta_i\bar{\theta}_j\bar{\theta}_k(1-\alpha_1)+\bar{\theta}_i\theta_j\bar{\theta}_k(1-\alpha_2)+\bar{\theta}_i\bar{\theta}_j\theta_k(1-\alpha_3)}$
$ICI\vert\Psi\psi\phi$	$\frac{(1-\alpha_2)\bar{\theta}_i\theta_j\bar{\theta}_k}{\bar{\theta}_i\bar{\theta}_j\bar{\theta}_k(1-\sum_{i=4}^7\alpha_i)+\theta_i\bar{\theta}_j\bar{\theta}_k(1-\alpha_1)+\bar{\theta}_i\theta_j\bar{\theta}_k(1-\alpha_2)+\bar{\theta}_i\bar{\theta}_j\theta_k(1-\alpha_3)}$
$IIC\vert\Psi\psi\phi$	$\frac{(1-\alpha_3)\bar{\theta}_i\bar{\theta}_j\theta_k}{\bar{\theta}_i\bar{\theta}_j\bar{\theta}_k(1-\sum_{i=4}^7\alpha_i)+\theta_i\bar{\theta}_j\bar{\theta}_k(1-\alpha_1)+\bar{\theta}_i\theta_j\bar{\theta}_k(1-\alpha_2)+\bar{\theta}_i\bar{\theta}_j\theta_k(1-\alpha_3)}$
$III\vert\Psi\psi\phi$	$\frac{(1-\alpha_4-\alpha_5-\alpha_6-\alpha_7)\bar{\theta}_i\bar{\theta}_j\bar{\theta}_k}{\bar{\theta}_i\bar{\theta}_j\bar{\theta}_k(1-\sum_{i=4}^7\alpha_i)+\theta_i\bar{\theta}_j\bar{\theta}_k(1-\alpha_1)+\bar{\theta}_i\theta_j\bar{\theta}_k(1-\alpha_2)+\bar{\theta}_i\bar{\theta}_j\theta_k(1-\alpha_3)}$
All other values	0

7 Model A: Annotating multiple dimensions, introducing dependence among dimensions

Using the p-dimensional multivariate binary distribution form described by Cox [4]:

$$P(\mathbf{Y}=\mathbf{y}|\Theta) \;=\; \prod_{i=1}^{p} P(Y_i=y_i|\Theta)\left\{1+\sum_{i>j}\rho^{ij}u_iu_j+\sum_{i>j>k}\rho^{ijk}u_iu_ju_k+\ldots\right\}, \tag{21}$$

we will model joint distribution of non-independent annotations for multiple dimensions.

$$U_i \stackrel{def}{=} \frac{Y_i-P(Y_i=1)}{\sqrt{P(Y_i=1))[1-P(Y_i=1)]}} = \frac{Y_i-\theta_i}{\left(\theta_i\bar{\theta}_i\right)^{\frac{1}{2}}}. \tag{22}$$

In this formulation, Y_i is a binary (0 or 1 valued) variable for all $i = 1, 2, \ldots, p$; and ρ_{ij}, ρ_{ijk}, and ρ_{ijkl} are the second-, third-, and fourth-way correlations

$$\rho^{ijk\ldots} \stackrel{def}{=} E(U_i U_j U_k \ldots), \tag{23}$$

correspondingly. If we map the correct (C) and incorrect (I) values of our "correctness" variables to integers 1 and 0, respectively, we can write a joint likelihood function for truth values of multiple annotations by the same triplet of evaluators.

In our modeling, we define the following shorthand notation (a variance-normalized variable representing annotations for dimensions x and y for the same sentence as provided by the lth evaluator) by analogy with Equation 22:

$$\xi_l^{xy} \stackrel{def}{=} \xi_l^{xy}(\mathbf{T}_{ijk}^x, \mathbf{T}_{ijk}^y) = \begin{cases} \left[\frac{\bar{\theta}_l^x \bar{\theta}_l^y}{\theta_l^x \theta_l^y}\right]^{\frac{1}{2}}, \text{ iff } t_l^x = C \wedge t_l^y = C, \\ -\left[\frac{\bar{\theta}_l^x \theta_l^y}{\theta_l^x \bar{\theta}_l^y}\right]^{\frac{1}{2}}, \text{ iff } t_l^x = C \wedge t_l^y = I, \\ -\left[\frac{\theta_l^x \bar{\theta}_l^y}{\bar{\theta}_l^x \theta_l^y}\right]^{\frac{1}{2}}, \text{ iff } t_l^x = I \wedge t_l^y = C, \\ \left[\frac{\theta_l^x \theta_l^y}{\bar{\theta}_l^x \bar{\theta}_l^y}\right]^{\frac{1}{2}}, \text{ iff } t_l^x = I \wedge t_l^y = I, \end{cases} \tag{24}$$

where $l \in \{i, j, k\}$.

Given a set of three annotators (i, j, and k) and N distinct annotation dimensions provided for the same sentence by these annotators, we can write the likelihood of a complex annotation set in the following way (by analogy with Equation 21):

$$
\begin{aligned}
P(\mathbf{A}_{ijk} = \{a^1, a^2, \dots, a^N\}|\Theta) &= \sum_{\mathbf{T}_{ijk}} P(\mathbf{A}_{ijk}|\mathbf{T}_{ijk}, \Theta) \times P(\mathbf{T}_{ijk}|\Theta) && (25) \\
&= \sum_{\mathbf{T}_{ijk}} [\prod_{x}^{N} \{P(\mathbf{A}_{ijk}^{x} = a^x | \mathbf{T}_{ijk}^{x} = t^x, \Theta)\} \\
&\times \; P(\mathbf{T}_{ijk} = \{\mathbf{T}_{ijk}^{1}, \dots, \mathbf{T}_{ijk}^{N}\}|\Theta)] && (26) \\
&= \sum_{\mathbf{T}_{ijk}} [\prod_{x}^{N} \{P(\mathbf{A}_{ijk}^{x} = a^x | \mathbf{T}_{ijk}^{x} = t^x, \Theta) P(\mathbf{T}_{ijk}^{x} = t^x|\Theta)\} \\
&\times \; \Xi(\mathbf{T}_{ijk})], && (27)
\end{aligned}
$$

where

$$
\Xi(\mathbf{T}_{ijk}) = \left(1 + \sum_{x>y} \sum_{l \in \{i,j,k\}} \rho_{xy} \, \xi_l^{xy}\right), \tag{28}
$$

Here, departing from our earlier definition of $\mathbf{A}_{ijk}$ as a 1×3 vector, $\mathbf{A}_{ijk}$ is an $N \times 3$ matrix.

If all ρ_{xy} are zero, $\mathbf{\Lambda}(\mathbf{T}_{ijk}) = 1$; this case corresponds to the assumption of complete independence between annotations of different dimensions.
Put differently, if

$$
\mathbf{R}^{x} \stackrel{def}{=} P(\mathbf{A}_{ijk}^{x}|\mathbf{T}_{ijk}^{x}, \Theta), \tag{29}
$$

$$
\mathbf{R}^{y} \stackrel{def}{=} P(\mathbf{A}_{ijk}^{y}|\mathbf{T}_{ijk}^{y}, \Theta), \tag{30}
$$

and

$$
\mathbf{R}^{xy} \stackrel{def}{=} P(\mathbf{A}_{ijk}^{x}, \mathbf{A}_{ijk}^{y}|\mathbf{T}_{ijk}^{x}, \mathbf{T}_{ijk}^{y} \Theta), \tag{31}
$$

we have

$$\mathbf{R}^{xy} = \mathbf{R}^{x} \otimes \mathbf{R}^{y}, \tag{32}$$

where $\otimes$ is the Kronecker matrix product ($\mathbf{A} \otimes \mathbf{B} \stackrel{def}{=} (a_{ij}\mathbf{B})$; see Figure 3 for a visual explanation and p. 29 in [5]).

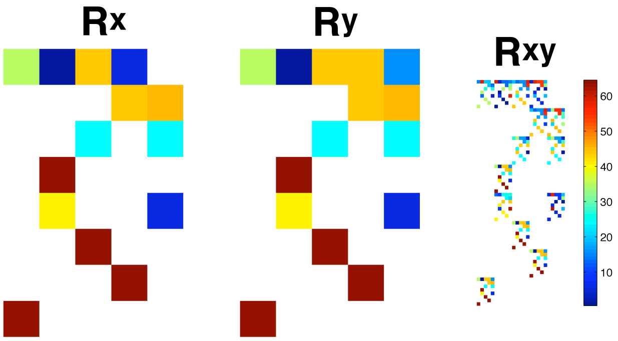

Figure 3: Model A: Matrices, dimension x has three annotation values, while dimension y has four. All probability values are scaled by 100. In this example $\theta_i^x = 0.95, \theta_j^x = 0.4, \theta_k^x = 0.6, \theta_i^y = 0.6, \theta_j^y = 0.9, \theta_k^y = 0.7, \rho_{xy} = 0.1, \alpha_1 = 0.3, \alpha_2 = 0.5, \alpha_3 = 0.6, \alpha_4 = 0.1, \alpha_5 = 0.2, \alpha_6 = 0.3$, and $\alpha_7 = 0.4$.

8 Model A: Numerical data simulation

Let us consider annotations for a single dimension with just three annotation values ($M = 3$: expected frequencies of three-evaluator patterns. $\omega_1 = 0.1$, $\omega_2 = 0.3$, $\omega_3 = 0.6$, $\theta_1 = 0.6$, $\theta_2 = 0.8$, and $\theta_3 = 0.5$. Estimates: $\hat{\omega}_1 = 0.0792$, $\hat{\omega}_2 = 0.2966$, and $\hat{\omega}_3 = 0.6242$.). Here are the data:

Table 9: Model A simulation: $M = 3$: expected frequencies of three-evaluator patterns. $\omega_1 = 0.1$, $\omega_2 = 0.3$, $\omega_3 = 0.6$, $\theta_1 = 0.6$, $\theta_2 = 0.8$, and $\theta_3 = 0.5$. Estimates: $\hat{\omega}_1 = 0.0792$, $\hat{\omega}_2 = 0.2966$, and $\hat{\omega}_3 = 0.6242$.

Pattern: $[v_i, v_j, v_k]$	*Probability*	*Simulated data: counts*
111	0.0011298	4
112	0.0038489	32
113	0.0076978	83
121	0.0028356	26
122	0.0089567	99
123	0.00468	48
131	0.0056711	51
132	0.00468	38
133	0.035827	375
211	0.0029856	37
212	0.0085067	80
213	0.00468	57
221	0.011547	143
222	0.030506	309
223	0.06928	690
231	0.00468	62
232	0.05104	521
233	0.10748	1,090
311	0.0059711	52
312	0.00468	53
313	0.034027	353
321	0.00468	42
322	0.05374	530
323	0.10208	1,035
331	0.046187	453
332	0.13856	1,325
333	0.24404	2,412
Total	1	10,000

Table 10: Model A simulation / model A estimation: Maximum likelihood parameter estimates for simulated data with "true" parameter values $\theta_i = 0.6$, $\theta_j = 0.8$, and $\theta_k = 0.5$, obtained in $1,000$ independent runs of simulated annealing.

$\log L_{max}$	$\hat{\theta}_i$ [0.6]	$\hat{\theta}_j$ [0.8]	$\hat{\theta}_k$ [0.5]	*Times visited*	*Comment*
−25527.871689	0.668050	0.728766	0.564728	396	← **Global**
−25879.609684	0.157385	0.132595	0.239020	562	← Local
−25536.806999	0.545150	1.000000	0.490790	20	← Local
−25536.828815	0.545262	0.999999	0.489812	4	← Local
−25571.781813	1.000000	0.545119	0.479177	9	← Local
−25536.842161	0.546401	0.999999	0.490438	1	← Local
−25537.202882	0.545510	1.000000	0.495285	1	← Local
−25571.793123	1.000000	0.545764	0.478727	2	← Local
−25571.860053	0.998901	0.545367	0.479291	2	← Local
−25805.836830	0.479160	0.490854	0.999999	2	← Local
−25537.521125	0.547845	0.999995	0.485529	1	← Local
−25536.997176	0.547409	0.999999	0.488768	1	← Local
−25805.885787	0.477935	0.491827	0.999995	1	← Local

Apparently, like in our earlier analysis [6], the likelihood surface has multiple modes.

Now let us use the same "data" for estimating parameters with the second model.

Table 11: Model A simulation / model B estimation: Maximum likelihood parameter estimates (under model B) for simulated data with "true" parameter values (generated under model A) $\theta_i = 0.6$, $\theta_j = 0.8$, and $\theta_k = 0.5$, obtained in 300 independent runs of simulated annealing. We show here only three results because only two estimates out of 300 ended in the same local optimum. It appears that model B generates a large number of local extrema.

#	*Parameters*	*Estimates*					
1:	$\log L_{max} = -25510.168554$	**(Mode #1)**					
	$[\hat{\gamma}_1, \hat{\gamma}_2]$	0.072429	0.091682				
	$[\hat{\lambda}^{(i)}_{2\|1}, \hat{\lambda}^{(i)}_{3\|1}; \hat{\lambda}^{(i)}_{1\|2}, \hat{\lambda}^{(i)}_{3\|2}; \hat{\lambda}^{(i)}_{1\|3}, \hat{\lambda}^{(i)}_{2\|3}]$	0.643967	0.181024	0.069346	0.860127	0.070614	0.291446
	$[\hat{\lambda}^{(j)}_{2\|1}, \hat{\lambda}^{(j)}_{3\|1}; \hat{\lambda}^{(j)}_{1\|2}, \hat{\lambda}^{(j)}_{3\|2}; \hat{\lambda}^{(j)}_{1\|3}, \hat{\lambda}^{(j)}_{2\|3}]$	0.563529	0.228430	0.037449	0.928269	0.066827	0.296033
	$[\hat{\lambda}^{(k)}_{2\|1}, \hat{\lambda}^{(k)}_{3\|1}; \hat{\lambda}^{(k)}_{1\|2}, \hat{\lambda}^{(k)}_{3\|2}; \hat{\lambda}^{(k)}_{1\|3}, \hat{\lambda}^{(k)}_{2\|3}]$	0.317300	0.254958	0.543633	0.000000	0.006615	0.279374
2:	$\log L_{max} = -25513.788112$	**(Mode #2)**					
	$[\hat{\gamma}_1, \hat{\gamma}_2]$	0.052294	0.048655				

Table 11: Model A simulation / model B estimation: Maximum likelihood parameter estimates (under model B) for simulated data with "true" parameter values (generated under model A) $\theta_i = 0.6$, $\theta_j = 0.8$, and $\theta_k = 0.5$, obtained in 300 independent runs of simulated annealing. We show here only three results because only two estimates out of 300 ended in the same local optimum. It appears that model B generates a large number of local extrema.

#	*Parameters*	*Estimates*					
	$[\hat{\lambda}^{(i)}_{2\vert 1}, \hat{\lambda}^{(i)}_{3\vert 1}; \hat{\lambda}^{(i)}_{1\vert 2}, \hat{\lambda}^{(i)}_{3\vert 2}; \hat{\lambda}^{(i)}_{1\vert 3}, \hat{\lambda}^{(i)}_{2\vert 3}]$	0.000012	0.136700	0.227834	0.068975	0.022146	0.294202
	$[\hat{\lambda}^{(j)}_{2\vert 1}, \hat{\lambda}^{(j)}_{3\vert 1}; \hat{\lambda}^{(j)}_{1\vert 2}, \hat{\lambda}^{(j)}_{3\vert 2}; \hat{\lambda}^{(j)}_{1\vert 3}, \hat{\lambda}^{(j)}_{2\vert 3}]$	0.058033	0.613684	0.097179	0.044550	0.062505	0.277868
	$[\hat{\lambda}^{(k)}_{2\vert 1}, \hat{\lambda}^{(k)}_{3\vert 1}; \hat{\lambda}^{(k)}_{1\vert 2}, \hat{\lambda}^{(k)}_{3\vert 2}; \hat{\lambda}^{(k)}_{1\vert 3}, \hat{\lambda}^{(k)}_{2\vert 3}]$	0.041889	0.848665	0.333664	0.131729	0.073904	0.299874
3:	$\log L_{max} = -25733.589116$	**(Mode #3)**					
	$[\hat{\gamma}_1, \hat{\gamma}_2]$	0.017506	0.000000				
	$[\hat{\lambda}^{(i)}_{2\vert 1}, \hat{\lambda}^{(i)}_{3\vert 1}; \hat{\lambda}^{(i)}_{1\vert 2}, \hat{\lambda}^{(i)}_{3\vert 2}; \hat{\lambda}^{(i)}_{1\vert 3}, \hat{\lambda}^{(i)}_{2\vert 3}]$	0.217812	0.062223	0.334380	0.009519	0.067055	0.295998
	$[\hat{\lambda}^{(j)}_{2\vert 1}, \hat{\lambda}^{(j)}_{3\vert 1}; \hat{\lambda}^{(j)}_{1\vert 2}, \hat{\lambda}^{(j)}_{3\vert 2}; \hat{\lambda}^{(j)}_{1\vert 3}, \hat{\lambda}^{(j)}_{2\vert 3}]$	0.000000	0.000012	0.132163	0.279946	0.034066	0.303387
	$[\hat{\lambda}^{(k)}_{2\vert 1}, \hat{\lambda}^{(k)}_{3\vert 1}; \hat{\lambda}^{(k)}_{1\vert 2}, \hat{\lambda}^{(k)}_{3\vert 2}; \hat{\lambda}^{(k)}_{1\vert 3}, \hat{\lambda}^{(k)}_{2\vert 3}]$	0.239152	0.559573	0.114760	0.028080	0.086721	0.291228

9 Model B: Description

Let us introduce a random variable, C^{ξ}, to define the correct value of an instance of annotation for the ξth dimension. In this section we will be considering only one dimension at a time, so we will drop the superscript ξ in the equations to follow. We also introduce random variables V_i, V_j, and V_k to denote annotation values provided for the same fragment of text by evaluators i, j, and k, respectively.

Table 12: Model B: Parameters and notations.

Notation		*Explanation*
C	$\longrightarrow$	A random variable representing the correct value of annotation for the zth dimension for a given text fragment.
$C = \psi$	$\longrightarrow$	A specific value (ψ) of the correct annotation variable.
γ^z_ψ	$\longrightarrow$	$P(C = \psi \vert \Theta)$
$\lambda_{v_x \vert \psi}$	$\longrightarrow$	$P(V_x = v_x \vert C = \psi, \Theta)$.
Θ	$\longrightarrow$	A shorthand for all model parameters.

To make the resulting equations more compact, we introduce the following model parameters. Let γ_ψ be the probability that the *correct* annotation for a fragment has value ψ. Further, let $\lambda^x_{v_x|\psi}$ be the probability that evaluator x assigns annotation value v_x to a text fragment, given that the correct annotation for this text fragment is ψ.

The joint distribution of the correct annotation value (C) and three annotations provided by evaluators i, j, and k are defined as follows—we explicitly assume that, given the true value of annotation, evaluators are independent in their choice of annotations.

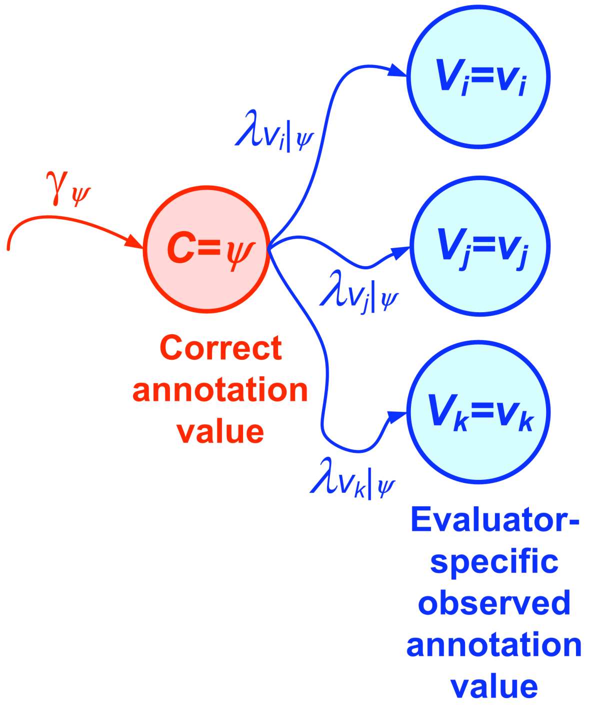

Figure 4: Model B: Graphical-model-style outline (one annotation dimension, three evaluators are annotating the same fragment of text).

$$
\begin{aligned}
P(C=\psi, V_i=v_i, V_j=v_j, V_k=v_k|\Theta) &= P(C=\psi|\Theta) \\
&\times P(V_i=v_i|C=\psi,\Theta) \\
&\times P(V_j=v_j|C=\psi,\Theta) \\
&\times P(V_k=v_k|C=\psi,\Theta) \\
&= \gamma_\psi \lambda^{(i)}_{v_i|\psi} \lambda^{(j)}_{v_j|\psi} \lambda^{(k)}_{v_k|\psi}. \qquad (33)
\end{aligned}
$$

The likelihood of a triplet of annotations for evaluators i, j and k is as follows:

$$
\begin{aligned}
P(V_i = v_i, V_j = v_j, V_k = v_k|\Theta) &= \sum_{\psi} P(C=\psi|\Theta) \\
&\times \; P(V_i = v_i|C=\psi,\Theta) \\
&\times \; P(V_j = v_j|C=\psi,\Theta) \\
&\times \; P(V_k = v_k|C=\psi,\Theta) \\
&= \sum_{\psi} P(C=\psi, V_i = v_i, V_j = v_j, V_k = v_k|\Theta) \quad (34) \\
&= \sum_{\psi} \gamma_\psi \lambda^{(i)}_{v_i|\psi} \lambda^{(j)}_{v_j|\psi} \lambda^{(k)}_{v_k|\psi}. \quad (35)
\end{aligned}
$$

To analyze the real data, we will also need to be able to compute the posterior probability that annotation value ψ is correct, given the observed annotation values for three annotators and known values of model parameters.

$$
\begin{aligned}
P(C=\psi|V_i = v_i, V_j = v_j, V_k = v_k, \Theta) &= \frac{P(C=\psi, V_i = v_i, V_j = v_j, V_k = v_k|\Theta)}{P(V_i = v_i, V_j = v_j, V_k = v_k|\Theta)} \quad (36) \\
&= \frac{\gamma_\psi \lambda^{(i)}_{v_i|\psi} \lambda^{(j)}_{v_j|\psi} \lambda^{(k)}_{v_k|\psi}}{\sum_\mu \gamma_\mu \lambda^{(i)}_{v_i|\mu} \lambda^{(j)}_{v_j|\mu} \lambda^{(k)}_{v_k|\mu}}. \quad (37)
\end{aligned}
$$

To make a comparison with model A easier, we can also define the probability of success (correct annotation) for the xth evaluator:

$$
\begin{aligned}
\theta_x &= \sum_\psi P(C=\psi|\Theta) P(V_x = \psi|C=\psi,\Theta) \quad (38) \\
&= \sum_\psi \gamma_\psi \lambda^{(x)}_{\psi|\psi}. \quad (39)
\end{aligned}
$$

If we have N distinct annotations that were generated by the triplet of evaluators (i, j, and k), we can compute the joint likelihood for all these data points:

$$
P(\{\mathbf{V}^{(n)}_{ijk}\}_{n=1,\dots,N}|\Theta) = \prod_{n=1}^{N} \left(\sum_\psi \gamma_\psi \lambda^{(i)}_{v^{(n)}_i|\psi} \lambda^{(j)}_{v^{(n)}_j|\psi} \lambda^{(k)}_{v^{(n)}_k|\psi} \right), \quad (40)
$$

and estimate the model parameters by maximizing the likelihood function with respect to parameter values.

10 Model B: Counting parameters and observations

For a dimension with M admissible values, we can observe M^3 distinct three-evaluator annotations and therefore we can have up to $M^3 - 1$ independent observation classes. For the same dimension (with M admissible values) we have to estimate $M - 1$ parameters related to $P(C = \psi|\Theta)$, and $3 \times [M \times (M-1)]$ parameters related to $P(V_x = v_x|C = \psi, \Theta)$.

This brings us to 7 observation classes and 7 parameters for a dimension with exactly 2 admissible values, 26 observation classes vs. 18 parameters for a dimension with three admissible values, and 63 vs. 36 for a dimension with four admissible values.

Therefore, in this model, the observation-to-parameter ratio grows with dimensions and observation classes enabling estimation for a sufficiently large data set.

11 Model B: Numerical data simulation

In our numerical example, the "true" parameter values are as follows (where M is the number the admissible annotation values, and $\mathbf{\Gamma}$ and $\mathbf{\Lambda}_x$ represent a vector of γ_ψ's and a matrix of $\lambda^{(x)}_{v_i|\psi}$'s, respectively).

$M = 2$:

$\mathbf{\Gamma} = [0.7, 0.3]$.

$$\mathbf{\Lambda}_i = \begin{bmatrix} 0.6 & 0.4 \\ 0.2 & 0.8 \end{bmatrix}, \mathbf{\Lambda}_j = \begin{bmatrix} 0.5 & 0.5 \\ 0.45 & 0.55 \end{bmatrix}, \mathbf{\Lambda}_k = \begin{bmatrix} 0.9 & 0.1 \\ 0.1 & 0.9 \end{bmatrix}.$$

The expected frequencies of all possible annotation patterns are shown in Table 13.

Table 13: Model B simulation: $M = 2$: expected frequencies of three-evaluator patterns.

Pattern	*Probability*
111	0.1917
112	0.0453
121	0.1923
122	0.0507
211	0.1368
212	0.1112
221	0.1392
222	0.1328
Total	1

10,000 "data points":

$N = [1882, 443, 1863, 506, 1376, 1152, 1412, 1366]$,

Table 14: Model B simulation / model B estimation: Example 1, $M = 2$, 40 independent runs of simulated annealing estimation (same data, different starting values of parameters). The "true" value for each parameter is shown in brackets. To perform the estimation, we first generated 10,000 "data points": N =[1882, 443, 1863, 506, 1376, 1152, 1412, 1366] for annotation patterns [111, 112, 121, 122, 211, 212, 221, 222], respectively. We can clearly see that the likelihood surface has two modes of exactly the same height (the less frequent mode is shown in bold).

$\log L_{max}$	$\hat{\gamma}_1$ [0.7]	$\hat{\lambda}_{i,2\|1}$ [0.4]	$\hat{\lambda}_{i,1\|2}$ [0.2]	$\hat{\lambda}_{j,2\|1}$ [0.5]	$\hat{\lambda}_{j,1\|2}$ [0.45]	$\hat{\lambda}_{k,2\|1}$ [0.1]	$\hat{\lambda}_{k,1\|2}$ [0.1]
-19866.653175	0.605930	0.376678	0.232727	0.494839	0.454762	0.060055	0.212549
-19866.653175	0.605901	0.376667	0.232741	0.494837	0.454763	0.060038	0.212577
-19866.653175	0.605933	0.376683	0.232732	0.494840	0.454763	0.060049	0.212535
**-19866.653175**	**0.394075**	**0.767266**	**0.623320**	**0.545237**	**0.505161**	**0.787455**	**0.939954**
-19866.653175	0.605942	0.376688	0.232728	0.494840	0.454764	0.060054	0.212525
-19866.653175	0.605930	0.376682	0.232732	0.494839	0.454763	0.060050	0.212539
-19866.653175	0.605935	0.376684	0.232731	0.494840	0.454762	0.060051	0.212535
-19866.653175	0.605934	0.376685	0.232733	0.494840	0.454763	0.060049	0.212532
-19866.653176	0.605929	0.376678	0.232733	0.494838	0.454760	0.060045	0.212541
**-19866.653175**	**0.394075**	**0.767261**	**0.623317**	**0.545237**	**0.505160**	**0.787462**	**0.939958**
-19866.653175	0.605966	0.376699	0.232723	0.494842	0.454762	0.060061	0.212490
-19866.653175	0.605923	0.376680	0.232739	0.494840	0.454764	0.060042	0.212545
-19866.653175	0.605931	0.376684	0.232734	0.494841	0.454763	0.060047	0.212535
-19866.653175	0.605933	0.376685	0.232733	0.494840	0.454763	0.060048	0.212532
-19866.653175	0.605935	0.376684	0.232731	0.494840	0.454763	0.060050	0.212533
-19866.653175	0.605941	0.376687	0.232731	0.494840	0.454762	0.060052	0.212525
-19866.653175	0.605911	0.376670	0.232733	0.494839	0.454763	0.060049	0.212575
-19866.653175	0.605931	0.376683	0.232734	0.494840	0.454763	0.060048	0.212535
-19866.653175	0.605929	0.376682	0.232734	0.494840	0.454763	0.060047	0.212539
-19866.653175	0.605932	0.376683	0.232732	0.494840	0.454763	0.060049	0.212535
-19866.653175	0.605934	0.376684	0.232732	0.494840	0.454763	0.060050	0.212534
-19866.653175	0.605936	0.376685	0.232731	0.494840	0.454763	0.060051	0.212532
-19866.653175	0.605934	0.376684	0.232732	0.494840	0.454763	0.060050	0.212534
-19866.653175	0.605934	0.376684	0.232732	0.494840	0.454763	0.060050	0.212534
-19866.653175	0.605933	0.376684	0.232732	0.494840	0.454763	0.060050	0.212535
-19866.653175	0.605934	0.376684	0.232731	0.494840	0.454763	0.060050	0.212533

-19866.653175	0.605934	0.376684	0.232731	0.494840	0.454763	0.060050	0.212533
**-19866.653175**	**0.394066**	**0.767269**	**0.623316**	**0.545237**	**0.505160**	**0.787466**	**0.939950**
-19866.653175	0.605934	0.376684	0.232732	0.494840	0.454763	0.060050	0.212534
-19866.653175	0.605934	0.376684	0.232731	0.494840	0.454763	0.060050	0.212534
-19866.653175	0.605934	0.376684	0.232731	0.494840	0.454763	0.060050	0.212534
-19866.653175	0.605934	0.376684	0.232732	0.494840	0.454763	0.060050	0.212534
-19866.653175	0.605934	0.376684	0.232732	0.494840	0.454763	0.060050	0.212534
-19866.653175	0.605936	0.376685	0.232731	0.494840	0.454763	0.060051	0.212532
-19866.653175	0.605934	0.376684	0.232731	0.494840	0.454763	0.060050	0.212534
-19866.653175	0.605935	0.376684	0.232731	0.494840	0.454763	0.060050	0.212532
-19866.653175	0.605935	0.376684	0.232731	0.494840	0.454763	0.060051	0.212534
-19866.653175	0.605934	0.376684	0.232731	0.494840	0.454763	0.060050	0.212534
-19866.653175	0.605934	0.376684	0.232732	0.494840	0.454763	0.060050	0.212534
-19866.653175	0.605934	0.376684	0.232732	0.494840	0.454763	0.060050	0.212535

10,000 “data points”:

$N = [564, 338, 1752, 867, 681, 342, 3730, 1726]$,

Table 15: Model B simulation / model B estimation: Example 2, $M = 2$, 20 independent runs of simulated annealing estimation (same data, different starting values of parameters). The “true” value for each parameter is shown in brackets. To perform the estimation, we first generated 10,000 “data points”: N =[564, 338, 1752, 867, 681, 342, 3730, 1726] for annotation patterns [111, 112, 121, 122, 211, 212, 221, 222], respectively. We can clearly see that the likelihood surface has two modes of exactly the same height (the less frequent mode is shown in bold). $[\theta_i, \theta_j, \theta_k] = [0.68, 0.83, 0.36]$, where $\theta_x = \sum_\psi \gamma_\psi \lambda_{x,\psi|\psi}$.

$\log L_{max}$	$\hat{\gamma}_1$ [0.2]	$\hat{\lambda}_{i,2\|1}$ [0.4]	$\hat{\lambda}_{i,1\|2}$ [0.3]	$\hat{\lambda}_{j,2\|1}$ [0.45]	$\hat{\lambda}_{j,1\|2}$ [0.45]	$\hat{\lambda}_{k,2\|1}$ [0.1]	$\hat{\lambda}_{k,1\|2}$ [0.7]
**-17633.090670**	**0.850300**	**0.712360**	**0.718234**	**0.868736**	**0.540325**	**0.312940**	**0.591135**
**-17633.090670**	**0.850300**	**0.712360**	**0.718234**	**0.868736**	**0.540325**	**0.312940**	**0.591135**
−17633.090670	0.149700	0.281766	0.287640	0.459675	0.131264	0.408865	0.687060
**-17633.090670**	**0.850300**	**0.712360**	**0.718234**	**0.868736**	**0.540325**	**0.312940**	**0.591135**
−17633.090670	0.149700	0.281766	0.287640	0.459675	0.131264	0.408865	0.687060
−17633.090670	0.149700	0.281766	0.287640	0.459675	0.131264	0.408865	0.687060
−17633.090670	0.149700	0.281766	0.287640	0.459675	0.131263	0.408865	0.687060
−17633.090670	0.149700	0.281766	0.287640	0.459675	0.131264	0.408865	0.687060
−17633.090670	0.149700	0.281766	0.287640	0.459676	0.131264	0.408865	0.687060
−17633.090670	0.149700	0.281766	0.287640	0.459675	0.131264	0.408865	0.687060
−17633.090670	0.149700	0.281766	0.287640	0.459676	0.131264	0.408865	0.687060

−17633.090670	0.149700	0.281766	0.287640	0.459676	0.131264	0.408865	0.687060
−17633.090670	0.149700	0.281766	0.287640	0.459675	0.131264	0.408865	0.687060
−17633.090670	0.149700	0.281766	0.287640	0.459675	0.131264	0.408865	0.687060
−17633.090670	0.149700	0.281766	0.287640	0.459675	0.131264	0.408865	0.687060
−17633.090670	0.149700	0.281766	0.287640	0.459675	0.131264	0.408865	0.687060
−17633.090670	0.149700	0.281766	0.287640	0.459676	0.131263	0.408865	0.687060
−17633.090670	0.149700	0.281766	0.287640	0.459676	0.131263	0.408865	0.687060
−17633.090670	0.149700	0.281766	0.287640	0.459675	0.131264	0.408865	0.687060
−17633.090670	0.149700	0.281766	0.287640	0.459675	0.131264	0.408865	0.687060

Table 16: Model B simulation / model A estimation: Example 2, $M = 2$, 50 independent runs of simulated annealing estimation (same data, different starting values of parameters). The "true" value for each parameter is shown in brackets. To perform the estimation, we first generated 10,000 "data points": N =[564, 338, 1752, 867, 681, 342, 3730, 1726] for annotation patterns [111, 112, 121, 122, 211, 212, 221, 222], respectively. We can clearly see that the likelihood surface has two modes. $[\theta_i, \theta_j, \theta_k] = [0.680.830.36]$, where $\theta_x = \sum_\psi \gamma_\psi \lambda_{x,\psi|\psi}$. .

Simulated Annealing run	$\log L_{max}$	$\hat{\theta}_i$ [0.68]	$\hat{\theta}_j$ [0.83]	$\hat{\theta}_k$ [0.36]	*Comment about the extremum*
	−13199.377582	0.309318	0.144775	0.663192	← Local
	−13194.999964	0.689629	0.858267	0.337781	
	−13194.999964	0.689629	0.858267	0.337781	
	−13199.377582	0.309318	0.144775	0.663192	← Local
	−13199.377582	0.309318	0.144775	0.663192	← Local
	−13194.999964	0.689629	0.858267	0.337781	
	−13194.999964	0.689629	0.858267	0.337781	
	−13194.999964	0.689629	0.858267	0.337781	
	−13194.999964	0.689629	0.858267	0.337781	
	−13194.999964	0.689629	0.858267	0.337781	
	−13194.999964	0.689629	0.858267	0.337781	
	−13194.999964	0.689629	0.858267	0.337781	
	−13199.377582	0.309318	0.144775	0.663192	← Local
	−13194.999964	0.689629	0.858267	0.337781	
	−13194.999964	0.689629	0.858267	0.337781	
	−13194.999964	0.689629	0.858267	0.337781	
	−13194.999964	0.689629	0.858267	0.337781	
	−13194.999964	0.689629	0.858267	0.337781	
	−13199.377582	0.309318	0.144775	0.663192	← Local
	−13194.999964	0.689629	0.858267	0.337781	
	−13194.999964	0.689629	0.858267	0.337781	

Table 16: Model B simulation / model A estimation: Example 2, $M = 2$, 50 independent runs of simulated annealing estimation (same data, different starting values of parameters). The "true" value for each parameter is shown in brackets. To perform the estimation, we first generated 10,000 "data points": N =[564, 338, 1752, 867, 681, 342, 3730, 1726] for annotation patterns [111, 112, 121, 122, 211, 212, 221, 222], respectively. We can clearly see that the likelihood surface has two modes. $[\theta_i, \theta_j, \theta_k] = [0.680.830.36]$, where $\theta_x = \sum_\psi \gamma_\psi \lambda_{x,\psi|\psi}$. .

Simulated Annealing run	$\log L_{max}$	$\hat{\theta}_i$ [0.68]	$\hat{\theta}_j$ [0.83]	$\hat{\theta}_k$ [0.36]	*Comment about the extremum*
	−13194.999964	0.689629	0.858267	0.337781	
	−13194.999964	0.689629	0.858267	0.337781	
	−13194.999964	0.689629	0.858267	0.337781	
	−13194.999964	0.689629	0.858267	0.337781	
	−13194.999964	0.689629	0.858267	0.337781	

12 Model B and the Expectation-Maximization algorithm

We explain below how to implement the Expectation-Maximization algorithm for the simplest case in which a group of 3 evaluators annotates a text fragment with 2 admissible values of annotation. It is straightforward to generalize this approach to a larger number of evaluators and allowed annotations.

Let us denote all model parameters of model B with vector Θ.

We assume there are three annotators working independently, numbered 1, 2, and 3 and that each fragment was annotated with one of two possible annotations, which again we denote by 1 and 2.

Let there be N data points represented by $\{(a_{i1}, a_{i2}, a_{i3})\}_{i=1}^N$ where a_{i1} represents annotator 1's annotation of the ith fragment, etc.

We have missing data in this problem which is the correct annotation for each data point. We will use a double to represent the missing data for each data point: $\{(x_{i1}, x_{i2})\}_{i=1}^N$. Here the x_{ik} are each either zero or one and the constraint $x_{i1} + x_{i2} = 1$ is obeyed for each data point i. If $x_{ik} = 1$ that means that value k is the correct annotation for the ith data point and all other values are incorrect. Using the variables representing the missing data we can write an expression for the probability of seeing the complete data for a data point.

$$
\begin{aligned}
P([a_{i1}, a_{i2}, a_{i3}], [x_{i1}, x_{i2}]|\Theta) &= \left(\lambda^{(1)}_{a_{i1}|1}\lambda^{(2)}_{a_{i2}|1}\lambda^{(3)}_{a_{i3}|1}\gamma_1\right)^{x_{i1}} \\
&\times \left(\lambda^{(1)}_{a_{i1}|2}\lambda^{(2)}_{a_{i2}|2}\lambda^{(3)}_{a_{i3}|2}\gamma_2\right)^{x_{i2}} . \qquad (41)
\end{aligned}
$$

Based on this the log probability of the complete data for the point can be written as

$$
\begin{aligned}
\log P([a_{i1}, a_{i2}, a_{i3}], [x_{i1}, x_{i2}]|\Theta) &= x_{i1} \log \left(\lambda^{(1)}_{a_{i1}|1}\lambda^{(2)}_{a_{i2}|1}\lambda^{(3)}_{a_{i3}|1}\gamma_1\right) \\
&+ x_{i2} \log \left(\lambda^{(1)}_{a_{i1}|2}\lambda^{(2)}_{a_{i2}|2}\lambda^{(3)}_{a_{i3}|2}\gamma_2\right) . \qquad (42)
\end{aligned}
$$

We assume that data for different data points are independent of each other. Then based on equation 42 we can write the log probability of the complete data (missing included) as

$$
\begin{aligned}
&\log P(complete\ data|\Theta) = \\
&\sum_{i=1}^{N} \left[x_{i1} \log \left(\lambda^{(1)}_{a_{i1}|1}\lambda^{(2)}_{a_{i2}|1}\lambda^{(3)}_{a_{i3}|1}\gamma_1\right) + x_{i2} \log \left(\lambda^{(1)}_{a_{i1}|2}\lambda^{(2)}_{a_{i2}|2}\lambda^{(3)}_{a_{i3}|2}\gamma_2\right)\right] . \qquad (43)
\end{aligned}
$$

Now the expectation step in the EM algorithm requires that we compute the expectation of the log likelihood of the data. To do this we note that the expectation of a variable that is either zero or one is just the probability that the variable takes the value one.

$$
E(x_{ij}) = P(x_{ij} = 1|\Theta). \qquad (44)
$$

For convenience we define the following notation

$$
\tilde{x}_{ij} \overset{\text{def}}{=} P(x_{ij} = 1|\Theta). \qquad (45)
$$

Then we have

$$
\begin{aligned}
&E\left[\log P(complete\ data|\Theta)\right] \\
= &\sum_{i=1}^{N} \left[\tilde{x}_{i1} \log \left(\lambda^{(1)}_{a_{i1}|1}\lambda^{(2)}_{a_{i2}|1}\lambda^{(3)}_{a_{i3}|1}\gamma_1\right) + \tilde{x}_{i2} \log \left(\lambda^{(1)}_{a_{i1}|2}\lambda^{(2)}_{a_{i2}|2}\lambda^{(3)}_{a_{i3}|2}\gamma_2\right)\right] \\
= &\sum_{i=1}^{N} \left[\tilde{x}_{i1} \left\{\sum_{l=1}^{3} \log \lambda^{(l)}_{a_{il}|1} + \log \gamma_1\right\} + \tilde{x}_{i2} \left\{\sum_{m=1}^{3} \log \lambda^{(m)}_{a_{im}|2} + \log \gamma_2\right\}\right] . \qquad (46)
\end{aligned}
$$

Initially we begin with a guess for the numbers $\tilde{x}_{ij}$. Shortly we will show how they are estimated. The next step in the EM algorithm is the M step. Here we need to maximize equation 46 based on the choice of the $\lambda^{(j)}_{n|k}$'s. Note that the lambdas are probabilities whose only constraint is that for each k and j

$$\sum_{n=1}^{2} \lambda^{(j)}_{n|k} = 1. \tag{47}$$

Using Lagrange multipliers we can estimate $\hat{\lambda}^{(j)}_{k|k}$ in terms of a_{ij}'s and $\tilde{x}_{il}$'s.

$$\hat{\lambda}^{(j)}_{m|k} = \frac{\sum_{i:a_{ij}=m} \tilde{x}_{ik}}{\sum_{i:a_{ij}=1} \tilde{x}_{i1} + \sum_{i:a_{ij}=2} \tilde{x}_{i2}}. \tag{48}$$

Similarly, for γ_k's we have the restriction

$$\sum_{n=1}^{2} \gamma_k = 1. \tag{49}$$

Again Lagrange multipliers can be used to show that

$$\hat{\gamma}_k = \frac{\sum_i \tilde{x}_{ik}}{\sum_{j=1}^{2} \sum_i \tilde{x}_{ij}}. \tag{50}$$

This completes the maximization step of the EM algorithm and defines a set of parameters Θ from the set of $\tilde{x}_{ij}$.

Once Θ is obtained from the maximization, new probabilities are defined by

$$\begin{aligned} \tilde{x}'_{ij} &= P(x_{ij} = 1 | [a_{i1}, a_{i2}, a_{i3}], \Theta) \\ &= \frac{P([a_{i1}, a_{i2}, a_{i3}] | x_{ij} = 1, \Theta)\gamma_j}{P([a_{i1}, a_{i2}, a_{i3}] | \Theta)} \\ &= \frac{\lambda^{(1)}_{a_{i1}|j} \lambda^{(2)}_{a_{i2}|j} \lambda^{(3)}_{a_{i3}|j} \gamma_j}{\sum_{n=1}^{2} \lambda^{(1)}_{a_{i1}|n} \lambda^{(2)}_{a_{i2}|n} \lambda^{(3)}_{a_{i3}|n} \gamma_n} \end{aligned} \tag{51}$$

using Bayes Theorem. The resulting $\tilde{x}'_{ij}$ are used to seed the next round of expectation—maximization. This process is repeated until some convergence criterion is met.

Effective Optimization. The EM algorithm as described works well to achieve a local maximum of the objective function (log likelihood of the data). There are many local maxima for this function, however, and so the results are not very satisfactory. To overcome this problem in part we take the following approach. We introduce a smoothing factor into equation 51. The result can be written

$$\tilde{x}'_{ij} = \frac{\lambda^{(1)}_{a_{i1}|j}\lambda^{(2)}_{a_{i2}|j}\lambda^{(3)}_{a_{i3}|j}\gamma_j + \epsilon C_{ij}}{\left[\sum_{n=1}^{2}\lambda^{(1)}_{a_{i1}|n}\lambda^{(2)}_{a_{i2}|n}\lambda^{(3)}_{a_{i3}|n}\gamma_n\right] + \epsilon N_i} \qquad (52)$$

Here for each example the number N_i is the number of annotators and C_{ij} is the number of annotators that annotated with the jth annotation. Thus the smoothing is a bias that tends to move the $\tilde{x}'_{ij}$ toward higher probabilities for what the annotators actually annotated. If we can move all of these probabilities toward agreement with what the actual annotations then the model naturally predicts a much higher probability for the data. This roughly corresponds to how one may believe a certain thing is true because a number of others state that it is true. The bias in equation 52 has the result that the system of equations does not exactly fulfill the conditions for the EM algorithm. Thus we are not guaranteed that the likelihood will be nondecreasing as we iterate, but in the cases we have investigated this is more than compensated for by the effect of shifting the $\tilde{x}'_{ij}$ toward agreement with the annotators. The strategy we have found to work best is to begin with a fairly large ϵ, say 0.1, and then run till convergence. Subsequently, set $\epsilon = 0$ and run to convergence again. In this way, the final run is a pure EM implementation and produces a local optimum, but we have moved things into a region so that this tends to be a much better optimum than it would have been had we chosen a random starting point and run EM exclusively. We compared this approach with running exclusively EM with 1 million distinct random starts and saving the best. Actually the strictly EM approach generally gives a slightly better result, but it may not correspond with what we think is the most reasonable solution. (One way to define the most reasonable solution as the one with the highest sum of θ's if the likelihoods are about the same.)

Figure 5 shows data from our calculations for a case of 3-evaluator annotations of a dimension with 3 admissible values using the "exclusive" EM algorithm; our efficient implementation of EM using equation 52 would generate values close to the global optimum.

13 Symmetries within solutions for model B

Symmetries. Let us suppose that the number of possible annotations for a fragment is n. Let S_n denote the symmetric group also known as the group of permutations on n objects. Let $\pi \in S_n$ be a permutation belonging to the group. Let $\left\{\lambda_{i|j}^{(k)}\right\}_{j=1}^{n}$ and $\{\gamma_j\}_{j=1}^{n}$ constitute a solution to the EM problem which maximizes the probability of the data given model parameters (likelihood) and also satisfies constraints that we impose on parameters (equations 49 and 47). Then using π we may define another solution:

$$\begin{aligned} \lambda_{ij}^{(k)\prime} &= \lambda_{i\pi(j)}^{(k)}, \\ \gamma_j' &= \gamma_{\pi(j)}. \end{aligned} \tag{53}$$

It is trivial to check that the new parameter set satisfies equations 47 and 49 and one can readily see that by applying the new parameter set in equations 51 one obtains a new set

$$\tilde{x}_{ij}' = \tilde{x}_{i\pi(j)}, \tag{54}$$

and with this new set $\left\{\tilde{x}_{i\pi(j)}\right\}$ and new parameters expression 46 achieves the same maximum. This new solution is of interest because the expression

$$\theta_k = \sum_{i=1}^{n} \gamma_i \lambda_{ii}^{(k)}, \tag{55}$$

is generally not invariant under the permutation π. Thus for a given π we can define

$$F(\pi) = \sum_{k=1}^{m} \sum_{i=1}^{n} \lambda_{i\pi(i)}^{(k)}, \tag{56}$$

Then we take that π which maximizes $F(\pi)$ as our preferred π and denote it with π^*. It is natural to choose the solution corresponding to π^* if we use correctness as our best predictive estimate for annotations. In the case where n is 2, there are two members of S_n, for 3 it is 6, for 4 it is 24, for 5 it is 120, and for 9 it is $362,880$. In general S_n has $n!$ elements.

References

[1] Ronald Aylmer Fisher. *The design of experiments.* Oliver and Boyde, Edinburgh, London, 1935.

[2] D. R. Cox and N. Reid. *The theory of the design of experiments*. Monographs on statistics and applied probability ; 86. Chapman & Hall/CRC, Boca Raton, 2000.

[3] Jiju Antony. *Design of experiments for engineers and scientists*. Butterworth-Heinemann, Oxford ; Burlinton, MA, 2003.

[4] D. R. Cox. Analysis of multivariate binary data. *Journal of the Royal Statistical Society Series C-Applied Statistics*, 21(2):113–120, 1972.

[5] C. Radhakrishna Rao. *Linear statistical inference and its applications*. Wiley series in probability and mathematical statistics. Wiley, New York, 2d edition, 1973.

[6] A. Rzhetsky, I. Iossifov, J. M. Loh, and K. P. White. Microparadigms: chains of collective reasoning in publications about molecular interactions. *Proc. Natl. Acad. Sci. U S A*, 103(13):4940–4945, 2006.

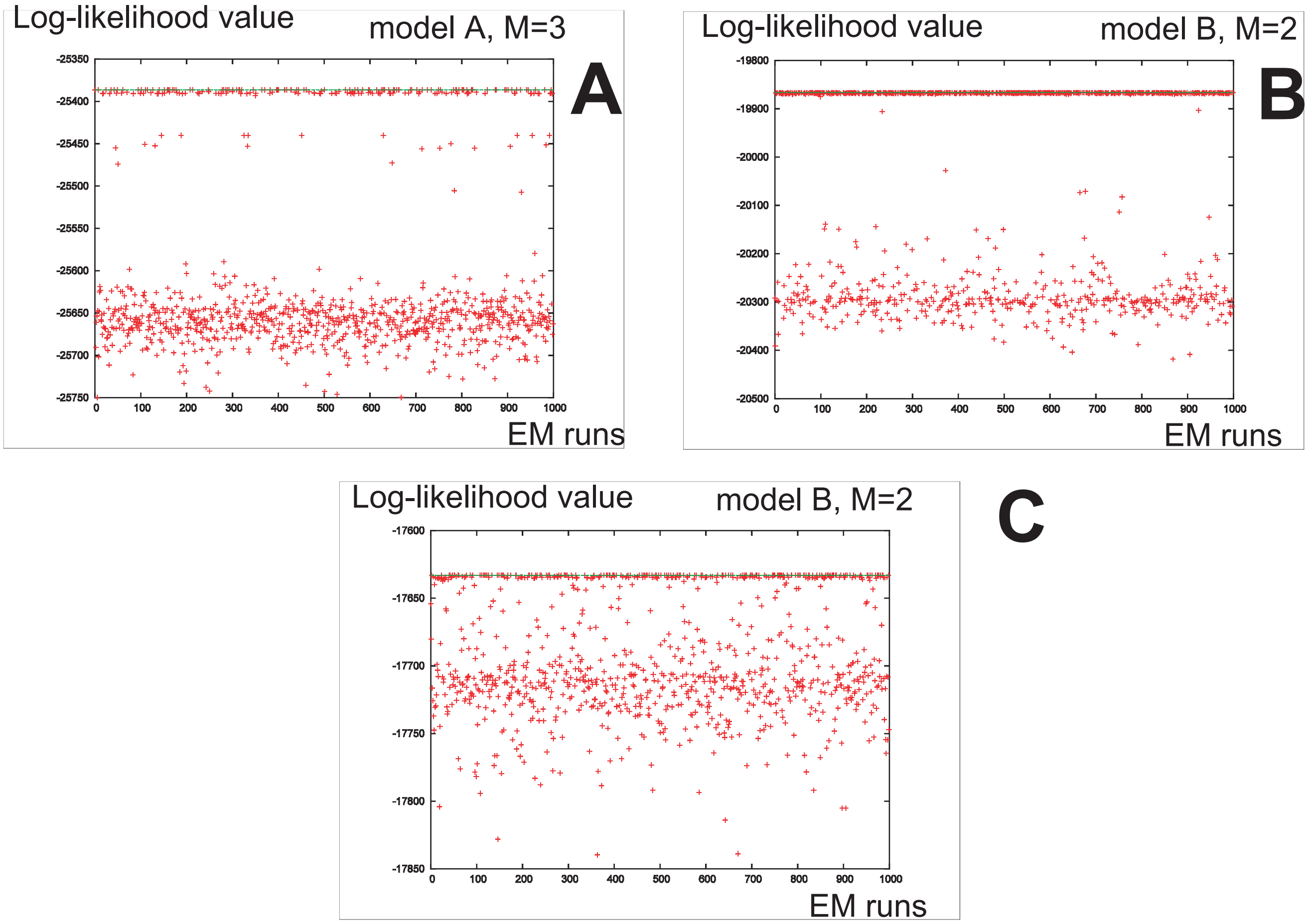

Figure 5: Testing implementation of our EM algorithm. Figures A, B, and C reproduce our earlier results represented in tables 9, 14, and 15, respectively. Note that likelihood values listed in tables 14 and 15 were obtained with Simulated Annealing (SA) algorithm that can jump among local optima, so that, in most SA runs, only the highest likelihood modes are visible. The EM algorithm, in contrast, is a strict hill-climbing algorithm that cannot escape from local optima even if there are other optima with better likelihood values. This explains the abundance of recovered lower likelihood optima with EM compared to SA.
