# Supplementary material for: How to Get the Most out of Your Curation Effort
Source: PLoS Comput Biol. 2009 May 22;5(5):e1000391. doi: 10.1371/journal.pcbi.1000391 (PMC2678295; doi:10.1371/journal.pcbi.1000391)
Supplement: Dataset S1 — Full dataset produced by the two-round annotation effort described in this study. (1.79 MB ZIP) [file pcbi.1000391.s002.zip › annotation-data/additional5.html]

|  |  |  |  |  |  |  |  |  |  |
| --- | --- | --- | --- | --- | --- | --- | --- | --- | --- |
| **#** | **No. in file** | **Id** | **Sentence** | **Columns** | **A1** | **A2** | **A3** | **A4** | **A5** |
| 1 | 5345 | 7559588\_100 | Figure 5:TrkA autophosphorylation in KCNR cells treated with IFN- . |A1:\*\*1SP3E3| |A2:\*\*1SP3E3| |A3:\*\*1SP3E3| |A4:\*\*1SP3E3| |A5:\*\*1GP3E3| |  |  |  |  |  |  |
|  |  |  |  | Annotation | 1SP3E3 | 1SP3E3 | 1SP3E3 | 1SP3E3 | 1GP3E3 |
|  |  |  |  | Evidence | 4 | 4 | 4 | 4 | 4 |
|  |  |  |  | Focus | 4 | 4 | 4 | 4 | 1 |
|  |  |  |  | Polarity | 7 | 7 | 7 | 7 | 7 |
| 2 | 6504 | 12433372\_265 | First, of the various mast cell markers examined, the IL-3-dependent PU.1 / progenitors express detectable transcripts of only one, mMC CPA. |A1:\*\*1SP3E1+| |A2:\*\*1SP3E0| |A3:\*\*1SP3E0| |A4:\*\*1SP3E0| |A5:\*\*1GP3E3| |  |  |  |  |  |  |
|  |  |  |  | Annotation | 1SP3E1+ | 1SP3E0 | 1SP3E0 | 1SP3E0 | 1GP3E3 |
|  |  |  |  | Evidence | 2 | 1 | 1 | 1 | 4 |
|  |  |  |  | Focus | 4 | 4 | 4 | 4 | 1 |
|  |  |  |  | Polarity | 7 | 7 | 7 | 7 | 7 |
| 3 | 6712 | 9620677\_97 | Some recent data may suggest that this is also true in the human disease (Conrad et al. 1997), |A1:\*\*1GP1E3| |A2:\*\*1SP1E3| |A3:\*\*1GP2E2| |A4:\*\*1GP2E2| |A5:\*\*1GP2E2| although of course in this case it is much more difficult to demonstrate that a certain antigen and certain T cells are indeed the cause of the disease. |A1:\*\*2SP0E0| |A2:\*\*2SP0E0| |A3:\*\*2SP0E0| |A4:\*\*2SP0E0| |A5:\*\*2GP1E0| |  |  |  |  |  |  |
|  |  |  |  | Annotation | 1GP1E3 2SP0E0 | 1SP1E3 2SP0E0 | 1GP2E2 2SP0E0 | 1GP2E2 2SP0E0 | 1GP2E2 2GP1E0 |
|  |  |  |  | Evidence | 4 1 | 4 1 | 3 1 | 3 1 | 3 1 |
|  |  |  |  | Focus | 1 4 | 4 4 | 1 4 | 1 4 | 1 1 |
|  |  |  |  | Polarity | 5 4 | 5 4 | 6 4 | 6 4 | 6 5 |
| 4 | 6794 | 9862746\_178 | However, it seems unlikely that the protection is due to inhibition of prolyl endopeptidase activity, |A1:\*\*1SN1E0-| |A2:\*\*1SN1E0-| |A3:\*\*1SN1E0-| |A4:\*\*1SN1E0-| |A5:\*\*1SN1E0-| because this enzymatic activity is unaltered during age induced age-induced apoptosis (data not shown). |A1:\*\*2SP3E1| |A2:\*\*2SP3E3| |A3:\*\*2SP3E3| |A4:\*\*2SP3E3| |A5:\*\*2SP2E3| |  |  |  |  |  |  |
|  |  |  |  | Annotation | 1SN1E0- 2SP3E1 | 1SN1E0- 2SP3E3 | 1SN1E0- 2SP3E3 | 1SN1E0- 2SP3E3 | 1SN1E0- 2SP2E3 |
|  |  |  |  | Evidence | 1 2 | 1 4 | 1 4 | 1 4 | 1 4 |
|  |  |  |  | Focus | 4 4 | 4 4 | 4 4 | 4 4 | 4 4 |
|  |  |  |  | Polarity | 3 7 | 3 7 | 3 7 | 3 7 | 3 6 |
| 5 | 8040 | 10903313\_53 | The protection of labeled pAPI from proteinase K digestion in the absence or presence of detergent was analyzed in osmotically lysed spheroplasts by autoradiography after immunoprecipitation (see "Experimental Procedures" and the legend to Fig. 5). |A1:\*\*1MSP3E3| |A2:\*\*1MSP3E3| |A3:\*\*1MP3E3| |A4:\*\*1MP3E3| |A5:\*\*1MP3E3| |  |  |  |  |  |  |
|  |  |  |  | Annotation | 1MSP3E3 | 1MSP3E3 | 1MP3E3 | 1MP3E3 | 1MP3E3 |
|  |  |  |  | Evidence | 4 | 4 | 4 | 4 | 4 |
|  |  |  |  | Focus | 6 | 6 | 2 | 2 | 2 |
|  |  |  |  | Polarity | 7 | 7 | 7 | 7 | 7 |
| 6 | 1563 | 11700328\_19 | Putative transmembrane domains ( ) are highlighted with gray. |A1:\*\*1GP3E2| |A2:\*\*1GP3E0| |A3:\*\*1GP3E0| |A4:\*\*1GP3E0| |A5:\*\*1GP2E3| |  |  |  |  |  |  |
|  |  |  |  | Annotation | 1GP3E2 | 1GP3E0 | 1GP3E0 | 1GP3E0 | 1GP2E3 |
|  |  |  |  | Evidence | 3 | 1 | 1 | 1 | 4 |
|  |  |  |  | Focus | 1 | 1 | 1 | 1 | 1 |
|  |  |  |  | Polarity | 7 | 7 | 7 | 7 | 6 |
| 7 | 2261 | 12086874\_206 | Western blotting analysis of primary astrocytes ( K) also demonstrated that P-Akt Ser473 levels were increased in tumor cells (T 1-2) relative to normal cells ( C), |A1:\*\*1SP3E3+| |A2:\*\*1MSP3E3+| |A3:\*\*1MP3E3+| |A4:\*\*1MP3E3+| |A5:\*\*1MP3E3+| while the level of total Akt and p-Erk1,2Thy183/Tyr185 were unchanged. |A1:\*\*2SP3E3| |A2:\*\*2SP3E3| |A3:\*\*2SP3E0| |A4:\*\*2SP3E0| |A5:\*\*2SP3E3| |  |  |  |  |  |  |
|  |  |  |  | Annotation | 1SP3E3+ 2SP3E3 | 1MSP3E3+ 2SP3E3 | 1MP3E3+ 2SP3E0 | 1MP3E3+ 2SP3E0 | 1MP3E3+ 2SP3E3 |
|  |  |  |  | Evidence | 4 4 | 4 4 | 4 1 | 4 1 | 4 4 |
|  |  |  |  | Focus | 4 4 | 6 4 | 2 4 | 2 4 | 2 4 |
|  |  |  |  | Polarity | 7 7 | 7 7 | 7 7 | 7 7 | 7 7 |
| 8 | 1628 | 11278665\_37 | It has also been suggested that TRAIL-R could, with the intermediary of FADD, recruit TRADD and activate NF-kappaB in this way (17). |A1:\*\*1SP2E2| |A2:\*\*1SP3E2| |A3:\*\*1SP2E2| |A4:\*\*1SP2E2| |A5:\*\*1SP2E2+| |  |  |  |  |  |  |
|  |  |  |  | Annotation | 1SP2E2 | 1SP3E2 | 1SP2E2 | 1SP2E2 | 1SP2E2+ |
|  |  |  |  | Evidence | 3 | 3 | 3 | 3 | 3 |
|  |  |  |  | Focus | 4 | 4 | 4 | 4 | 4 |
|  |  |  |  | Polarity | 6 | 7 | 6 | 6 | 6 |
| 9 | 3216 | 12453426\_73 | Note that samples in lanes 5/6 and 7/8 were reconstituted with the after-displacement BER substrate and the 5 nt noncomplementary flap substrate, respectively. |A1:\*\*1MP3E3| |A2:\*\*1MP3E3| |A3:\*\*1MP3E3| |A4:\*\*1MP3E3| |A5:\*\*1MP3E3| |  |  |  |  |  |  |
|  |  |  |  | Annotation | 1MP3E3 | 1MP3E3 | 1MP3E3 | 1MP3E3 | 1MP3E3 |
|  |  |  |  | Evidence | 4 | 4 | 4 | 4 | 4 |
|  |  |  |  | Focus | 2 | 2 | 2 | 2 | 2 |
|  |  |  |  | Polarity | 7 | 7 | 7 | 7 | 7 |
| 10 | 9456 | 9039267\_319 | This work was supported by the Dutch Cancer Society. |A1:\*\*1GP3E0| |A2:\*\*1GP3E0| |A3:\*\*1GP3E3| |A4:\*\*1GP3E3| |A5:\*\*1GP3E0| |  |  |  |  |  |  |
|  |  |  |  | Annotation | 1GP3E0 | 1GP3E0 | 1GP3E3 | 1GP3E3 | 1GP3E0 |
|  |  |  |  | Evidence | 1 | 1 | 4 | 4 | 1 |
|  |  |  |  | Focus | 1 | 1 | 1 | 1 | 1 |
|  |  |  |  | Polarity | 7 | 7 | 7 | 7 | 7 |
| 11 | 3680 | 11841944\_564 | IC50 measurements were performed in an analogous fashion to that described for the 4-epimerase; compound 1-143 was dispensed to the microplate, a solution containing the dehydrogenase was added, and the solution was incubated at room temperature until the absorbance achieved a stable baseline, and finally the reaction was initiated by the addition of UDP-Glc. |A1:\*\*1MP3E3| |A2:\*\*1MP3E3| |A3:\*\*1MP3E3| |A4:\*\*1MP3E3| |A5:\*\*1MP3E3| |  |  |  |  |  |  |
|  |  |  |  | Annotation | 1MP3E3 | 1MP3E3 | 1MP3E3 | 1MP3E3 | 1MP3E3 |
|  |  |  |  | Evidence | 4 | 4 | 4 | 4 | 4 |
|  |  |  |  | Focus | 2 | 2 | 2 | 2 | 2 |
|  |  |  |  | Polarity | 7 | 7 | 7 | 7 | 7 |
| 12 | 3038 | 12190174\_12 | Evaluation of antiplatelet antibodies, using an antigen-specific assay, was useful in supporting this diagnosis. |A1:\*\*1MSP3E3| |A2:\*\*1MP3E3| |A3:\*\*1MP3E3| |A4:\*\*1MP3E3| |A5:\*\*1MP3E3| |  |  |  |  |  |  |
|  |  |  |  | Annotation | 1MSP3E3 | 1MP3E3 | 1MP3E3 | 1MP3E3 | 1MP3E3 |
|  |  |  |  | Evidence | 4 | 4 | 4 | 4 | 4 |
|  |  |  |  | Focus | 6 | 2 | 2 | 2 | 2 |
|  |  |  |  | Polarity | 7 | 7 | 7 | 7 | 7 |
| 13 | 5793 | 10428033\_224 | Since the affinity of cytochrome bd oxidase for oxygen is very high (Km 20 nM - 1 muM), |A1:\*\*1SP3E0+| |A2:\*\*1SP3E0+| |A3:\*\*1SP3E0+| |A4:\*\*1SP3E0+| we carried out our experiments in strains containing null mutations in both cytochrome bd and bo oxidases to avoid trace quantities of oxygen that would allow the flow of electrons through cytochrome bd oxidase. |A1:\*\*2MSP3E3| |A2:\*\*2MSP3E3| |A3:\*\*2MP3E3| |A4:\*\*2MP3E3| |A5:\*\*1MP3E3| |  |  |  |  |  |  |
|  |  |  |  | Annotation | 1SP3E0+ 2MSP3E3 | 1SP3E0+ 2MSP3E3 | 1SP3E0+ 2MP3E3 | 1SP3E0+ 2MP3E3 | 1MP3E3 1MP3E3 |
|  |  |  |  | Evidence | 1 4 | 1 4 | 1 4 | 1 4 | 4 4 |
|  |  |  |  | Focus | 4 6 | 4 6 | 4 2 | 4 2 | 2 2 |
|  |  |  |  | Polarity | 7 7 | 7 7 | 7 7 | 7 7 | 7 7 |
| 14 | 30 | 10721990\_248 | Here we show that WNT-7a, secreted by GCs, plays a role in this process. |A1:\*\*1SP3E3| |A2:\*\*1SP3E3| |A3:\*\*1SP3E3| |A4:\*\*1SP3E3| |A5:\*\*1GP3E3| |  |  |  |  |  |  |
|  |  |  |  | Annotation | 1SP3E3 | 1SP3E3 | 1SP3E3 | 1SP3E3 | 1GP3E3 |
|  |  |  |  | Evidence | 4 | 4 | 4 | 4 | 4 |
|  |  |  |  | Focus | 4 | 4 | 4 | 4 | 1 |
|  |  |  |  | Polarity | 7 | 7 | 7 | 7 | 7 |
| 15 | 9117 | 12193063\_5 | No interconversion of the two forms was detected after purification. |A1:\*\*1GN3E3| |A2:\*\*1MN3E3| |A3:\*\*1GN3E3| |A4:\*\*1GN3E3| |A5:\*\*1GN3E3| |  |  |  |  |  |  |
|  |  |  |  | Annotation | 1GN3E3 | 1MN3E3 | 1GN3E3 | 1GN3E3 | 1GN3E3 |
|  |  |  |  | Evidence | 4 | 4 | 4 | 4 | 4 |
|  |  |  |  | Focus | 1 | 2 | 1 | 1 | 1 |
|  |  |  |  | Polarity | 1 | 1 | 1 | 1 | 1 |
| 16 | 8304 | 9882705\_153 | As shown in Fig. 6, 1-h pretreatment of cells with 25 muM PhoCho or DiC8 inhibited by 30% DNA fragmentation |A1:\*\*1MSN3E3| |A2:\*\*1SN3E3-| |A4:\*\*1SP3E3-| induced by 1 muM DNR and MXT. |A1:\*\*2SP3E3| |A2:\*\*2SP3E3| |A3:\*\*1MP3E3| |A4:\*\*2SP3E3| |A5:\*\*1MP3E3-| |  |  |  |  |  |  |
|  |  |  |  | Annotation | 1MSN3E3 2SP3E3 | 1SN3E3- 2SP3E3 | 1MP3E3 1MP3E3 | 1SP3E3- 2SP3E3 | 1MP3E3- 1MP3E3- |
|  |  |  |  | Evidence | 4 4 | 4 4 | 4 4 | 4 4 | 4 4 |
|  |  |  |  | Focus | 6 4 | 4 4 | 2 2 | 4 4 | 2 2 |
|  |  |  |  | Polarity | 1 7 | 1 7 | 7 7 | 7 7 | 7 7 |
| 17 | 7814 | 11274124\_37 | The root and the stem were checked for the efficacy of sterilization by rolling them on 0.1% tryptic soy agar (TSA) plates. |A1:\*\*1MSP3E3| |A2:\*\*1MP3E3| |A3:\*\*1MP3E3| |A4:\*\*1MP3E3| |A5:\*\*1MP3E3| |  |  |  |  |  |  |
|  |  |  |  | Annotation | 1MSP3E3 | 1MP3E3 | 1MP3E3 | 1MP3E3 | 1MP3E3 |
|  |  |  |  | Evidence | 4 | 4 | 4 | 4 | 4 |
|  |  |  |  | Focus | 6 | 2 | 2 | 2 | 2 |
|  |  |  |  | Polarity | 7 | 7 | 7 | 7 | 7 |
| 18 | 9033 | 11861801\_19 | At least 14 members of the caspase family have been identified (Van de Craen et al., 1998). |A1:\*\*1SP3E2| |A2:\*\*1SP3E2| |A3:\*\*1SP3E2| |A4:\*\*1SP3E2| |A5:\*\*1SGP3E2| |  |  |  |  |  |  |
|  |  |  |  | Annotation | 1SP3E2 | 1SP3E2 | 1SP3E2 | 1SP3E2 | 1SGP3E2 |
|  |  |  |  | Evidence | 3 | 3 | 3 | 3 | 3 |
|  |  |  |  | Focus | 4 | 4 | 4 | 4 | 5 |
|  |  |  |  | Polarity | 7 | 7 | 7 | 7 | 7 |
| 19 | 4167 | 11114897\_101 | Other data obtained in the course of characterizing this mutant's biochemical activity in crude extracts |A5:\*\*1GP3E3| suggested that the T124I mutant may also accumulate cleaved substrate molecules even under recombination conditions (data not shown). |A5:\*\*2SP2E3| |A1:\*\*1SP2E1| |A2:\*\*1SP2E3| |A3:\*\*1SP3E3| |A4:\*\*1SP3E3| |  |  |  |  |  |  |
|  |  |  |  | Annotation | 1SP2E1 1SP2E1 | 1SP2E3 1SP2E3 | 1SP3E3 1SP3E3 | 1SP3E3 1SP3E3 | 1GP3E3 2SP2E3 |
|  |  |  |  | Evidence | 2 2 | 4 4 | 4 4 | 4 4 | 4 4 |
|  |  |  |  | Focus | 4 4 | 4 4 | 4 4 | 4 4 | 1 4 |
|  |  |  |  | Polarity | 6 6 | 6 6 | 7 7 | 7 7 | 7 6 |
| 20 | 2656 | 10877790\_113 | The analysis indicated that the sandy loam rhizosphere (C1) and interspace (C0) soil communities at Cosnino were the most similar in composition, |A1:\*\*1SP3E3| whereas the cinders interspace (S0) community was the most different. |A1:\*\*2SP3E3| |A2:\*\*1SP3E3| |A3:\*\*1SP3E3| |A4:\*\*1SP3E3| |A5:\*\*1GP3E3| |  |  |  |  |  |  |
|  |  |  |  | Annotation | 1SP3E3 2SP3E3 | 1SP3E3 1SP3E3 | 1SP3E3 1SP3E3 | 1SP3E3 1SP3E3 | 1GP3E3 1GP3E3 |
|  |  |  |  | Evidence | 4 4 | 4 4 | 4 4 | 4 4 | 4 4 |
|  |  |  |  | Focus | 4 4 | 4 4 | 4 4 | 4 4 | 1 1 |
|  |  |  |  | Polarity | 7 7 | 7 7 | 7 7 | 7 7 | 7 7 |
| 21 | 7526 | 9843507\_145 | This gap is lost in dCtBP mutants (Figure 3D), |A4:\*\*1SP2E3-| |A5:\*\*1SP3E3-| similar to the situation observed in knirps- embryos (Arnosti et al., 1996). |A4:\*\*2SP3E2| |A1:\*\*1SP3E23| |A2:\*\*1SP3E23| |A3:\*\*1SP3E3| |A5:\*\*1SP3E2| |  |  |  |  |  |  |
|  |  |  |  | Annotation | 1SP3E23 1SP3E23 | 1SP3E23 1SP3E23 | 1SP3E3 1SP3E3 | 1SP2E3- 2SP3E2 | 1SP3E3- 1SP3E2 |
|  |  |  |  | Evidence | 3 3 | 3 3 | 4 4 | 4 3 | 4 3 |
|  |  |  |  | Focus | 4 4 | 4 4 | 4 4 | 4 4 | 4 4 |
|  |  |  |  | Polarity | 7 7 | 7 7 | 7 7 | 6 7 | 7 7 |
| 22 | 990 | 10956650\_12 | It is likely that this difference reflects the amount of annexin molecules bound per unit amount of phospholipid surface area or curvature. |A1:\*\*1GP2E1| |A2:\*\*1SP2E1| |A3:\*\*1SP2E0| |A4:\*\*1SP2E0| |A5:\*\*1GP1E0| |  |  |  |  |  |  |
|  |  |  |  | Annotation | 1GP2E1 | 1SP2E1 | 1SP2E0 | 1SP2E0 | 1GP1E0 |
|  |  |  |  | Evidence | 2 | 2 | 1 | 1 | 1 |
|  |  |  |  | Focus | 1 | 4 | 4 | 4 | 1 |
|  |  |  |  | Polarity | 6 | 6 | 6 | 6 | 5 |
| 23 | 3596 | 10805799\_15 | Presumably through interactions with surface membrane-associated VH3-encoded B-cell antigen receptors (11), |A2:\*\*1SP3E2| |A5:\*\*1SP2E2| in vitro stimulation with SpA can contribute to selection of these B cells |A1:\*\*1SP3E2| and promote their production of antibodies that may include rheumatoid factor autoantibodies ( 12, 13). |A1:\*\*2SP1E2+| |A2:\*\*2SP2E2+| |A3:\*\*1SP2E2| |A4:\*\*1SP2E2| |A5:\*\*2SP3E2+| |  |  |  |  |  |  |
|  |  |  |  | Annotation | 1SP3E2 1SP3E2 2SP1E2+ | 1SP3E2 2SP2E2+ 2SP2E2+ | 1SP2E2 1SP2E2 1SP2E2 | 1SP2E2 1SP2E2 1SP2E2 | 1SP2E2 2SP3E2+ 2SP3E2+ |
|  |  |  |  | Evidence | 3 3 3 | 3 3 3 | 3 3 3 | 3 3 3 | 3 3 3 |
|  |  |  |  | Focus | 4 4 4 | 4 4 4 | 4 4 4 | 4 4 4 | 4 4 4 |
|  |  |  |  | Polarity | 7 7 5 | 7 6 6 | 6 6 6 | 6 6 6 | 6 7 7 |
| 24 | 3511 | 9390512\_37 | As it happens, prevalence of a disease is also affected by its mode of inheritance, as is nicely demonstrated in the case of maturity onset-type diabetes of youth (MODY) and insulin-dependent diabetes mellitus (IDDM). |A1:\*\*1GP3E3| |A2:\*\*1SP3E1| |A3:\*\*1SP3E3| |A4:\*\*1SP3E3| |A5:\*\*1GP3E3| |  |  |  |  |  |  |
|  |  |  |  | Annotation | 1GP3E3 | 1SP3E1 | 1SP3E3 | 1SP3E3 | 1GP3E3 |
|  |  |  |  | Evidence | 4 | 2 | 4 | 4 | 4 |
|  |  |  |  | Focus | 1 | 4 | 4 | 4 | 1 |
|  |  |  |  | Polarity | 7 | 7 | 7 | 7 | 7 |
| 25 | 3772 | 12887906\_155 | Although rad55 strains did allow delayed but extensive Rad51p association with MAT, |A1:\*\*1SP3E3| |A2:\*\*1SP3E3| |A3:\*\*1SP3E0| |A4:\*\*1SP3E0| |A5:\*\*1SP3E3+| they failed to exhibit an association of Rad51p with the HML donor (Figures 6A and 6C) |A1:\*\*2SN3E3| |A2:\*\*2SN3E3| |A3:\*\*2SP3E3| |A4:\*\*2SP3E3| |A5:\*\*2SP3E3-| |  |  |  |  |  |  |
|  |  |  |  | Annotation | 1SP3E3 2SN3E3 | 1SP3E3 2SN3E3 | 1SP3E0 2SP3E3 | 1SP3E0 2SP3E3 | 1SP3E3+ 2SP3E3- |
|  |  |  |  | Evidence | 4 4 | 4 4 | 1 4 | 1 4 | 4 4 |
|  |  |  |  | Focus | 4 4 | 4 4 | 4 4 | 4 4 | 4 4 |
|  |  |  |  | Polarity | 7 1 | 7 1 | 7 7 | 7 7 | 7 7 |
| 26 | 2746 | 11606579\_43 | We would like to propose that the negative charge introduced by the BH group at the Pro- Rp position (Fig. 1) becomes the driving force for PPi release. |A1:\*\*1SP3E3| |A2:\*\*1SP3E3| |A3:\*\*1SP2E3| |A4:\*\*1SP2E3| |A5:\*\*1SP3E3| |  |  |  |  |  |  |
|  |  |  |  | Annotation | 1SP3E3 | 1SP3E3 | 1SP2E3 | 1SP2E3 | 1SP3E3 |
|  |  |  |  | Evidence | 4 | 4 | 4 | 4 | 4 |
|  |  |  |  | Focus | 4 | 4 | 4 | 4 | 4 |
|  |  |  |  | Polarity | 7 | 7 | 6 | 6 | 7 |
| 27 | 6641 | 11340075\_128 | Because most wild type IC expressing wild-type IC-expressing cells displayed a partial phenotype, |A1:\*\*1GP3E0| we also examined the effects of S84A and S84D mutant expression on late endosome-lysosome distribution. |A1:\*\*2SP3E3| |A2:\*\*1SP3E3| |A3:\*\*1SP3E3| |A4:\*\*1SP3E3| |A5:\*\*1SP3E3| |  |  |  |  |  |  |
|  |  |  |  | Annotation | 1GP3E0 2SP3E3 | 1SP3E3 1SP3E3 | 1SP3E3 1SP3E3 | 1SP3E3 1SP3E3 | 1SP3E3 1SP3E3 |
|  |  |  |  | Evidence | 1 4 | 4 4 | 4 4 | 4 4 | 4 4 |
|  |  |  |  | Focus | 1 4 | 4 4 | 4 4 | 4 4 | 4 4 |
|  |  |  |  | Polarity | 7 7 | 7 7 | 7 7 | 7 7 | 7 7 |
| 28 | 6215 | 10671457\_11 | Removal of the 40 C-terminal amino acids, residues 247 to 286, of FAEXynZ resulted in protein without activity. |A1:\*\*1SP3E3| |A2:\*\*1SP3E3| |A3:\*\*1SP3E3| |A4:\*\*1SP3E3| |A5:\*\*1GN3E3| |  |  |  |  |  |  |
|  |  |  |  | Annotation | 1SP3E3 | 1SP3E3 | 1SP3E3 | 1SP3E3 | 1GN3E3 |
|  |  |  |  | Evidence | 4 | 4 | 4 | 4 | 4 |
|  |  |  |  | Focus | 4 | 4 | 4 | 4 | 1 |
|  |  |  |  | Polarity | 7 | 7 | 7 | 7 | 1 |
| 29 | 4905 | 9573199\_70 | Recombination substrates were prepared by linearizing cosmid pYUB325 with PacI (New England Biolabs), |A5:\*\*1MP3E3| which releases the chromosomal sequences as a single fragment. |A5:\*\*2GP3E0| |A1:\*\*1SP3E3| |A2:\*\*1SP3E3| |A3:\*\*1MP3E3| |A4:\*\*1MP3E3| |  |  |  |  |  |  |
|  |  |  |  | Annotation | 1SP3E3 1SP3E3 | 1SP3E3 1SP3E3 | 1MP3E3 1MP3E3 | 1MP3E3 1MP3E3 | 1MP3E3 2GP3E0 |
|  |  |  |  | Evidence | 4 4 | 4 4 | 4 4 | 4 4 | 4 1 |
|  |  |  |  | Focus | 4 4 | 4 4 | 2 2 | 2 2 | 2 1 |
|  |  |  |  | Polarity | 7 7 | 7 7 | 7 7 | 7 7 | 7 7 |
| 30 | 1555 | 10581284\_244 | Graphs corresponding to Figure 3 for WL are not shown, |A3:\*\*1SN3E3| |A4:\*\*1SN3E3| |A5:\*\*1GN3E3| but the chi2 values for each of the models are shown in Table 4. |A3:\*\*2SP3E3| |A1:\*\*1GP3E3| |A2:\*\*1GP3E3| |A4:\*\*2SP3E3| |A5:\*\*2GP3E3| |  |  |  |  |  |  |
|  |  |  |  | Annotation | 1GP3E3 1GP3E3 | 1GP3E3 1GP3E3 | 1SN3E3 2SP3E3 | 1SN3E3 2SP3E3 | 1GN3E3 2GP3E3 |
|  |  |  |  | Evidence | 4 4 | 4 4 | 4 4 | 4 4 | 4 4 |
|  |  |  |  | Focus | 1 1 | 1 1 | 4 4 | 4 4 | 1 1 |
|  |  |  |  | Polarity | 7 7 | 7 7 | 1 7 | 1 7 | 1 7 |
| 31 | 6095 | 10523308\_185 | It is possible that the motif1-hairpin and flanking sequences cannot function as an independent promoter |A5:\*\*1SN2E0| because it lacks sequences capable of directing de novo initiation of RNA synthesis. |A5:\*\*2SN3E0| |A1:\*\*1SN3E0| |A2:\*\*1SN2E0| |A3:\*\*1SN3E0| |A4:\*\*1SN3E0| |  |  |  |  |  |  |
|  |  |  |  | Annotation | 1SN3E0 1SN3E0 | 1SN2E0 1SN2E0 | 1SN3E0 1SN3E0 | 1SN3E0 1SN3E0 | 1SN2E0 2SN3E0 |
|  |  |  |  | Evidence | 1 1 | 1 1 | 1 1 | 1 1 | 1 1 |
|  |  |  |  | Focus | 4 4 | 4 4 | 4 4 | 4 4 | 4 4 |
|  |  |  |  | Polarity | 1 1 | 2 2 | 1 1 | 1 1 | 2 1 |
| 32 | 5441 | 9892664\_13 | Among these, ZO-1, ZO-2, and ZO-3 are related to each other and belong to the membrane-associated guanylate kinase homologues gene family, which contains PDZ, SH3, and GUK (guanylate kinase-like) domains (17-19). |A1:\*\*1SP3E2| |A2:\*\*1SP3E2| |A3:\*\*1SP3E2| |A4:\*\*1SP3E2| |A5:\*\*1SP3E2| |  |  |  |  |  |  |
|  |  |  |  | Annotation | 1SP3E2 | 1SP3E2 | 1SP3E2 | 1SP3E2 | 1SP3E2 |
|  |  |  |  | Evidence | 3 | 3 | 3 | 3 | 3 |
|  |  |  |  | Focus | 4 | 4 | 4 | 4 | 4 |
|  |  |  |  | Polarity | 7 | 7 | 7 | 7 | 7 |
| 33 | 3806 | 11287622\_79 | Primers 2571 (5''-CGCTCTCGGCCGAAGGACATC) and 2572 (5''-TCAACGATGTAAAAGAAGTCACCG) were used in a PCR reaction with genomic DNA from the serotype A strain H99 as template. |A1:\*\*1MP3E3| |A2:\*\*1MP3E3| |A3:\*\*1MP3E3| |A4:\*\*1MP3E3| |A5:\*\*1MP3E3| |  |  |  |  |  |  |
|  |  |  |  | Annotation | 1MP3E3 | 1MP3E3 | 1MP3E3 | 1MP3E3 | 1MP3E3 |
|  |  |  |  | Evidence | 4 | 4 | 4 | 4 | 4 |
|  |  |  |  | Focus | 2 | 2 | 2 | 2 | 2 |
|  |  |  |  | Polarity | 7 | 7 | 7 | 7 | 7 |
| 34 | 8470 | 12125819\_4 | The expression of lipA and nadAB, which are involved in lipoic acid synthesis and NAD synthesis, respectively, |A5:\*\*1GP3E1| was found to be reduced in the cells overproducing Era-dE. |A5:\*\*2GP3E3-| |A1:\*\*1SP3E3| |A2:\*\*1SP3E3-| |A3:\*\*1SP3E3+| |A4:\*\*1SP3E3+| |  |  |  |  |  |  |
|  |  |  |  | Annotation | 1SP3E3 1SP3E3 | 1SP3E3- 1SP3E3- | 1SP3E3+ 1SP3E3+ | 1SP3E3+ 1SP3E3+ | 1GP3E1 2GP3E3- |
|  |  |  |  | Evidence | 4 4 | 4 4 | 4 4 | 4 4 | 2 4 |
|  |  |  |  | Focus | 4 4 | 4 4 | 4 4 | 4 4 | 1 1 |
|  |  |  |  | Polarity | 7 7 | 7 7 | 7 7 | 7 7 | 7 7 |
| 35 | 6993 | 10698942\_456 | Laurent, M.N. , M.N., Blitz, I.L. , I.L., Hashimoto, C. , C., Rothbacher, U. and Cho, K.W. (1997) |A3:\*\*1GP3E3| |A4:\*\*1GP3E2| |A5:\*\*1GP3E2| The Xenopus homeobox gene twin mediates Wnt induction of goosecoid in establishment of Spemann's organizer. |A3:\*\*2SP3E0| |A1:\*\*1SP3E3| |A2:\*\*1SP3E2| |A4:\*\*2SP3E0| |A5:\*\*2SP3E0+| |  |  |  |  |  |  |
|  |  |  |  | Annotation | 1SP3E3 1SP3E3 | 1SP3E2 1SP3E2 | 1GP3E3 2SP3E0 | 1GP3E2 2SP3E0 | 1GP3E2 2SP3E0+ |
|  |  |  |  | Evidence | 4 4 | 3 3 | 4 1 | 3 1 | 3 1 |
|  |  |  |  | Focus | 4 4 | 4 4 | 1 4 | 1 4 | 1 4 |
|  |  |  |  | Polarity | 7 7 | 7 7 | 7 7 | 7 7 | 7 7 |
| 36 | 7587 | 10480939\_34 | 0.5 mug of GST54, 0.5 mug of cpSRP43, or 3.4 muCi of cpSRP54his (~0.2 mug) were mixed as indicated, purified on glutathione-Sepharose, subjected to SDS-PAGE, electrophoretically transferred to nitrocellulose, and analyzed by radioimaging ( lower panel) and immunoblotting ( upper panel) with antibodies against cpSRP54 and cpSRP43 as described under "Experimental Procedures." |A1:\*\*1MP3E3| |A2:\*\*1MP3E3| |A3:\*\*1MP3E3| |A4:\*\*1MP3E3| |A5:\*\*1MP3E3| Arabidopsis Contains a Chloroplast Homologue of Bacterial FtsY-- An alignment of the Arabidopsis FtsY homologue with related sequences is shown in Fig. 3. |A1:\*\*2MP3E3| |A2:\*\*2SP3E3| |A3:\*\*2SP3E3| |A4:\*\*2SP3E3| |A5:\*\*2SP3E3| High similarity among all sequences is observed in the C terminus containing the GTP-binding and hydrolysis domains. |A1:\*\*3SP3E3| |A2:\*\*3SP3E3| |A3:\*\*3SP3E0| |A4:\*\*3SP3E0| |A5:\*\*3GP3E1| |  |  |  |  |  |  |
|  |  |  |  | Annotation | 1MP3E3 2MP3E3 3SP3E3 | 1MP3E3 2SP3E3 3SP3E3 | 1MP3E3 2SP3E3 3SP3E0 | 1MP3E3 2SP3E3 3SP3E0 | 1MP3E3 2SP3E3 3GP3E1 |
|  |  |  |  | Evidence | 4 4 4 | 4 4 4 | 4 4 1 | 4 4 1 | 4 4 2 |
|  |  |  |  | Focus | 2 2 4 | 2 4 4 | 2 4 4 | 2 4 4 | 2 4 1 |
|  |  |  |  | Polarity | 7 7 7 | 7 7 7 | 7 7 7 | 7 7 7 | 7 7 7 |
| 37 | 4337 | 11159990\_229 | The number of histidine and tyrosine residues in these proteins, including those contained within the histidine triad motif, suggests that they may be involved in metal or nucleoside binding. |A1:\*\*1SP1E1| |A2:\*\*1SP2E1| |A3:\*\*1SP2E0| |A4:\*\*1SP2E0| |A5:\*\*1SP2E1| |  |  |  |  |  |  |
|  |  |  |  | Annotation | 1SP1E1 | 1SP2E1 | 1SP2E0 | 1SP2E0 | 1SP2E1 |
|  |  |  |  | Evidence | 2 | 2 | 1 | 1 | 2 |
|  |  |  |  | Focus | 4 | 4 | 4 | 4 | 4 |
|  |  |  |  | Polarity | 5 | 6 | 6 | 6 | 6 |
| 38 | 6786 | 12414801\_118 | As shown in Fig. 6 A, mutation of the MIP 3alpha MIP-3alpha promoter at either position reduced IL-1beta-mediated luciferase activity by ~75% ( p < 0.01) compared with the wild-type control. |A1:\*\*1SP3E3-| |A2:\*\*1SP3E3-| |A3:\*\*1SP3E3| |A4:\*\*1SP3E3| |A5:\*\*1SP3E3-| |  |  |  |  |  |  |
|  |  |  |  | Annotation | 1SP3E3- | 1SP3E3- | 1SP3E3 | 1SP3E3 | 1SP3E3- |
|  |  |  |  | Evidence | 4 | 4 | 4 | 4 | 4 |
|  |  |  |  | Focus | 4 | 4 | 4 | 4 | 4 |
|  |  |  |  | Polarity | 7 | 7 | 7 | 7 | 7 |
| 39 | 4159 | 9797273\_61 | Protein concentrations (15 mug) were estimated by the method of Lowry et al. (16), |A1:\*\*1MP3E2| |A2:\*\*1SP3E23| |A3:\*\*1MP3E2| |A4:\*\*1MP3E2| |A5:\*\*1MP3E2| and proteins were separated through a sodium dodecyl sulfate (SDS)-15% polyacrylamide minigel at 100 V with the Mini-Protean II apparatus (Bio-Rad Laboratories, Mississauga, Ontario, Canada). |A1:\*\*2MP3E2| |A2:\*\*1MP3E3| |A3:\*\*2MP3E3| |A4:\*\*2MP3E3| |A5:\*\*2SP3E3| |  |  |  |  |  |  |
|  |  |  |  | Annotation | 1MP3E2 2MP3E2 | 1SP3E23 1MP3E3 | 1MP3E2 2MP3E3 | 1MP3E2 2MP3E3 | 1MP3E2 2SP3E3 |
|  |  |  |  | Evidence | 3 3 | 3 4 | 3 4 | 3 4 | 3 4 |
|  |  |  |  | Focus | 2 2 | 4 2 | 2 2 | 2 2 | 2 4 |
|  |  |  |  | Polarity | 7 7 | 7 7 | 7 7 | 7 7 | 7 7 |
| 40 | 2872 | 10330411\_74 | The reaction mixture containing the [35] methionine- or [35]cysteine- (in vitro translation grade; ICN) labeled proteins was clarified by centrifugation before incubation with GST fusion proteins in fusion protein binding buffer for 120 min at 4 degrees C as described above. |A2:\*\*1MP3E3| |A1:\*\*1MP3E3| |A3:\*\*1MP3E3| |A4:\*\*1MP3E3| |A5:\*\*1MP3E2| |  |  |  |  |  |  |
|  |  |  |  | Annotation | 1MP3E3 | 1MP3E3 | 1MP3E3 | 1MP3E3 | 1MP3E2 |
|  |  |  |  | Evidence | 4 | 4 | 4 | 4 | 3 |
|  |  |  |  | Focus | 2 | 2 | 2 | 2 | 2 |
|  |  |  |  | Polarity | 7 | 7 | 7 | 7 | 7 |
| 41 | 889 | 9658201\_148 | In the more recent model (Haskell-Luevano et al., 1996), MTII, |A3:\*\*1SP3E2| |A4:\*\*1SP3E2| a cyclic lactam analogue, and the core tripeptide (D-Phe)-Arg-Trp were docked, and amino acids in all TM regions were identified as possible contact points. |A3:\*\*2SP2E3| |A1:\*\*1SP3E2| |A2:\*\*1SP3E2| |A4:\*\*2SP2E3| |A5:\*\*1SP3E2| |  |  |  |  |  |  |
|  |  |  |  | Annotation | 1SP3E2 1SP3E2 | 1SP3E2 1SP3E2 | 1SP3E2 2SP2E3 | 1SP3E2 2SP2E3 | 1SP3E2 1SP3E2 |
|  |  |  |  | Evidence | 3 3 | 3 3 | 3 4 | 3 4 | 3 3 |
|  |  |  |  | Focus | 4 4 | 4 4 | 4 4 | 4 4 | 4 4 |
|  |  |  |  | Polarity | 7 7 | 7 7 | 7 6 | 7 6 | 7 7 |
| 42 | 5516 | 9889191\_237 | In order to investigate further the role of porin in Neisseria-induced apoptosis, |A5:\*\*1GP3E0| we tested several isogenic gonococcal strains producing recombinant porins, |A4:\*\*1SP3E0| i.e. Ngo MS11 synthesizing PorBIA of strain VP1 (MS11 PorBIA), MS11 PorBIB synthesizing the authentic PorBIB, MS11 PorB Nla synthesizing a Neisseria lactamica PorB, and MS11 PorBdeltaloop1 synthesizing a mutant MS11 PorB with a deletion in loop 1 (Bauer et al., 1999). 1999). |A4:\*\*2SP3E2| |A1:\*\*1SP3E23| |A2:\*\*1SP3E23| |A3:\*\*1SP3E2| |A5:\*\*2SP3E23| |  |  |  |  |  |  |
|  |  |  |  | Annotation | 1SP3E23 1SP3E23 1SP3E23 | 1SP3E23 1SP3E23 1SP3E23 | 1SP3E2 1SP3E2 1SP3E2 | 1SP3E0 1SP3E0 2SP3E2 | 1GP3E0 2SP3E23 2SP3E23 |
|  |  |  |  | Evidence | 3 3 3 | 3 3 3 | 3 3 3 | 1 1 3 | 1 3 3 |
|  |  |  |  | Focus | 4 4 4 | 4 4 4 | 4 4 4 | 4 4 4 | 1 4 4 |
|  |  |  |  | Polarity | 7 7 7 | 7 7 7 | 7 7 7 | 7 7 7 | 7 7 7 |
| 43 | 3208 | 12052837\_1 | Glycosylphosphatidylinositol (GPI)-anchored proteins are synthesized on membrane-bound ribosomes, |A1:\*\*1SP3E3| translocated across the endoplasmic reticulum membrane, and GPI-anchored by GPI transamidase (GPIT). |A1:\*\*2SP3E3| |A2:\*\*1SP3E1| |A3:\*\*1SP3E0| |A4:\*\*1SP3E0| |A5:\*\*1SP3E0| |  |  |  |  |  |  |
|  |  |  |  | Annotation | 1SP3E3 2SP3E3 | 1SP3E1 1SP3E1 | 1SP3E0 1SP3E0 | 1SP3E0 1SP3E0 | 1SP3E0 1SP3E0 |
|  |  |  |  | Evidence | 4 4 | 2 2 | 1 1 | 1 1 | 1 1 |
|  |  |  |  | Focus | 4 4 | 4 4 | 4 4 | 4 4 | 4 4 |
|  |  |  |  | Polarity | 7 7 | 7 7 | 7 7 | 7 7 | 7 7 |
| 44 | 9502 | 12707304\_268 | Nuclear PKP3 could not be detected in contrast to PKP1 and PKP2 ( Mertens et al., 1996; Schmidt et al., 1997). |A1:\*\*1SN3E2| |A2:\*\*1SN3E2| |A3:\*\*1SN3E2| |A4:\*\*1SN3E2| |A5:\*\*1GN3E2| |  |  |  |  |  |  |
|  |  |  |  | Annotation | 1SN3E2 | 1SN3E2 | 1SN3E2 | 1SN3E2 | 1GN3E2 |
|  |  |  |  | Evidence | 3 | 3 | 3 | 3 | 3 |
|  |  |  |  | Focus | 4 | 4 | 4 | 4 | 1 |
|  |  |  |  | Polarity | 1 | 1 | 1 | 1 | 1 |
| 45 | 2983 | 11357142\_70 | Most sporadic colorectal tumours also involve constitutive activation of the Tcf-mediated Wnt transcriptional response, |A5:\*\*1SP2E0+| |A2:\*\*1SP2E1+| |A4:\*\*1SP3E0| due either to loss of APC |A5:\*\*2SP3E0-| or to stabilizing oncogenic mutations in beta-catenin 3, 28. |A5:\*\*3SP3E2| |A1:\*\*1SP3E2-| |A2:\*\*1SP2E2-| |A3:\*\*1SP2E0| |A4:\*\*2SP3E2| |  |  |  |  |  |  |
|  |  |  |  | Annotation | 1SP3E2- 1SP3E2- 1SP3E2- | 1SP2E1+ 1SP2E2- 1SP2E2- | 1SP2E0 1SP2E0 1SP2E0 | 1SP3E0 2SP3E2 2SP3E2 | 1SP2E0+ 2SP3E0- 3SP3E2 |
|  |  |  |  | Evidence | 3 3 3 | 2 3 3 | 1 1 1 | 1 3 3 | 1 1 3 |
|  |  |  |  | Focus | 4 4 4 | 4 4 4 | 4 4 4 | 4 4 4 | 4 4 4 |
|  |  |  |  | Polarity | 7 7 7 | 6 6 6 | 6 6 6 | 7 7 7 | 6 7 7 |
| 46 | 8243 | 9603979\_37 | Gal4-RAP46 fusion protein-encoding plasmid was generated by cloning the RAP46 sequence in frame into pSG424 (the Gal4-DBD vector) (23). |A1:\*\*1SP3E23| |A2:\*\*1SP3E23| |A3:\*\*1MP3E2| |A4:\*\*1MP3E2| |A5:\*\*1SP3E2| |  |  |  |  |  |  |
|  |  |  |  | Annotation | 1SP3E23 | 1SP3E23 | 1MP3E2 | 1MP3E2 | 1SP3E2 |
|  |  |  |  | Evidence | 3 | 3 | 3 | 3 | 3 |
|  |  |  |  | Focus | 4 | 4 | 2 | 2 | 4 |
|  |  |  |  | Polarity | 7 | 7 | 7 | 7 | 7 |
| 47 | 8078 | 8653784\_219 | Similar to our results with the Xmads, mRNA injection of some components of the Wnt signal transduction pathway, such as glycogen synthase kinase-3 or dishevelled, leads to activation of the Wnt signal |A1:\*\*1SP3E3+| |A2:\*\*1SP3E3+| |A3:\*\*1SP3E3| |A4:\*\*1SP3E3| |A5:\*\*1SP3E3+| |  |  |  |  |  |  |
|  |  |  |  | Annotation | 1SP3E3+ | 1SP3E3+ | 1SP3E3 | 1SP3E3 | 1SP3E3+ |
|  |  |  |  | Evidence | 4 | 4 | 4 | 4 | 4 |
|  |  |  |  | Focus | 4 | 4 | 4 | 4 | 4 |
|  |  |  |  | Polarity | 7 | 7 | 7 | 7 | 7 |
| 48 | 6528 | 9501094\_219 | Interestingly, the mutation of the DNA-binding sites (Figure 7) and the transactivation domain deletion mutants (Figure 8) |A3:\*\*1MP3E3| |A4:\*\*1MP3E3| have reciprocal effects on enhancer function: |A1:\*\*1SP3E3+| |A2:\*\*1SP3E3| |A5:\*\*1SP3E3| whereas a mutation of the E-box alone almost completely abolishes the activity of the enhancer element, |A1:\*\*2SN3E3-| |A2:\*\*1SN3E3-| |A5:\*\*2SP3E3-| USFdeltaTA has no effect. |A1:\*\*3SN3E3| |A2:\*\*3SN3E3| |A3:\*\*1SN3E0-| |A4:\*\*2SN3E0-| |A5:\*\*3SP3E3| |  |  |  |  |  |  |
|  |  |  |  | Annotation | 1SP3E3+ 1SP3E3+ 2SN3E3- 3SN3E3 | 1SP3E3 1SP3E3 1SN3E3- 3SN3E3 | 1MP3E3 1SN3E0- 1SN3E0- 1SN3E0- | 1MP3E3 2SN3E0- 2SN3E0- 2SN3E0- | 1SP3E3 1SP3E3 2SP3E3- 3SP3E3 |
|  |  |  |  | Evidence | 4 4 4 4 | 4 4 4 4 | 4 1 1 1 | 4 1 1 1 | 4 4 4 4 |
|  |  |  |  | Focus | 4 4 4 4 | 4 4 4 4 | 2 4 4 4 | 2 4 4 4 | 4 4 4 4 |
|  |  |  |  | Polarity | 7 7 1 1 | 7 7 1 1 | 7 1 1 1 | 7 1 1 1 | 7 7 7 7 |
| 49 | 1957 | 11408527\_15 | Adenosine exerts multiple actions throughout the body and modifies various cardiovascular functions (Berne, 1980). |A1:\*\*1GP3E2| |A2:\*\*1SP3E2| |A3:\*\*1SP3E2| |A4:\*\*1SP3E2| |A5:\*\*1GP3E2| |  |  |  |  |  |  |
|  |  |  |  | Annotation | 1GP3E2 | 1SP3E2 | 1SP3E2 | 1SP3E2 | 1GP3E2 |
|  |  |  |  | Evidence | 3 | 3 | 3 | 3 | 3 |
|  |  |  |  | Focus | 1 | 4 | 4 | 4 | 1 |
|  |  |  |  | Polarity | 7 | 7 | 7 | 7 | 7 |
| 50 | 9832 | 10490647\_749 | The Xenopus homeobox gene Twin mediates Wnt induction of Goosecoid in establishment of Spemann's organizer. |A1:\*\*1SP3E0| |A2:\*\*1SP3E0| |A3:\*\*1SP3E0| |A4:\*\*1SP3E0| |A5:\*\*1SP3E0+| |  |  |  |  |  |  |
|  |  |  |  | Annotation | 1SP3E0 | 1SP3E0 | 1SP3E0 | 1SP3E0 | 1SP3E0+ |
|  |  |  |  | Evidence | 1 | 1 | 1 | 1 | 1 |
|  |  |  |  | Focus | 4 | 4 | 4 | 4 | 4 |
|  |  |  |  | Polarity | 7 | 7 | 7 | 7 | 7 |
| 51 | 9464 | 9665968\_166 | Further confirmation was obtained with slide agglutination antiserum, which is an option for excluding possible false-positive strains. |A1:\*\*1SP3E3| |A2:\*\*1SP3E3| |A3:\*\*1SP3E0| |A4:\*\*1MP3E3| |A5:\*\*1MP3E1| |  |  |  |  |  |  |
|  |  |  |  | Annotation | 1SP3E3 | 1SP3E3 | 1SP3E0 | 1MP3E3 | 1MP3E1 |
|  |  |  |  | Evidence | 4 | 4 | 1 | 4 | 2 |
|  |  |  |  | Focus | 4 | 4 | 4 | 2 | 2 |
|  |  |  |  | Polarity | 7 | 7 | 7 | 7 | 7 |
| 52 | 4966 | 12173058\_16 | Our results further demonstrate that the faster and simple registration method allows us to use the system more frequently, |A5:\*\*1GP3E3| thereby increasing the number of navigated cases. |A5:\*\*2GP3E1+| |A1:\*\*1GP3E3+| |A2:\*\*1GP3E3+| |A3:\*\*1GP2E3| |A4:\*\*1SP3E3+| |  |  |  |  |  |  |
|  |  |  |  | Annotation | 1GP3E3+ 1GP3E3+ | 1GP3E3+ 1GP3E3+ | 1GP2E3 1GP2E3 | 1SP3E3+ 1SP3E3+ | 1GP3E3 2GP3E1+ |
|  |  |  |  | Evidence | 4 4 | 4 4 | 4 4 | 4 4 | 4 2 |
|  |  |  |  | Focus | 1 1 | 1 1 | 1 1 | 4 4 | 1 1 |
|  |  |  |  | Polarity | 7 7 | 7 7 | 6 6 | 7 7 | 7 7 |
| 53 | 5759 | 9632612\_169 | We previously reported that the NCHi-PRT 1 isolate in question had the same P2 sequence as an NCHi-PRT 25 isolate; |A3:\*\*1SP3E1| |A4:\*\*1SP3E3| |A5:\*\*1SP3E13| this was most likely a result of horizontal gene transfer (31). |A3:\*\*2SP2E2| |A1:\*\*1SP3E23| |A2:\*\*1SP3E23| |A4:\*\*2SP3E2| |A5:\*\*2SP3E2| |  |  |  |  |  |  |
|  |  |  |  | Annotation | 1SP3E23 1SP3E23 | 1SP3E23 1SP3E23 | 1SP3E1 2SP2E2 | 1SP3E3 2SP3E2 | 1SP3E13 2SP3E2 |
|  |  |  |  | Evidence | 3 3 | 3 3 | 2 3 | 4 3 | 2 3 |
|  |  |  |  | Focus | 4 4 | 4 4 | 4 4 | 4 4 | 4 4 |
|  |  |  |  | Polarity | 7 7 | 7 7 | 7 6 | 7 7 | 7 7 |
| 54 | 784 | 10747890\_60 | Termination probability was calculated as outlined under "Experimental Procedures." |A1:\*\*1GP3E3| |A2:\*\*1GP3E3| |A3:\*\*1GP3E3| |A4:\*\*1GP3E3| |A5:\*\*1GP3E3| |  |  |  |  |  |  |
|  |  |  |  | Annotation | 1GP3E3 | 1GP3E3 | 1GP3E3 | 1GP3E3 | 1GP3E3 |
|  |  |  |  | Evidence | 4 | 4 | 4 | 4 | 4 |
|  |  |  |  | Focus | 1 | 1 | 1 | 1 | 1 |
|  |  |  |  | Polarity | 7 | 7 | 7 | 7 | 7 |
| 55 | 3665 | 9419341\_90 | After pelleting, beads were washed in extract buffer, and the pellets and supernatant were analyzed by SDS-PAGE and Western blotting with anti-histone H3 antibody. |A1:\*\*1MP3E3| |A2:\*\*1MP3E3| |A3:\*\*1MP3E3| |A4:\*\*1MP3E3| |A5:\*\*1MP3E3| |  |  |  |  |  |  |
|  |  |  |  | Annotation | 1MP3E3 | 1MP3E3 | 1MP3E3 | 1MP3E3 | 1MP3E3 |
|  |  |  |  | Evidence | 4 | 4 | 4 | 4 | 4 |
|  |  |  |  | Focus | 2 | 2 | 2 | 2 | 2 |
|  |  |  |  | Polarity | 7 | 7 | 7 | 7 | 7 |
| 56 | 2529 | 10567588\_236 | Ubiquitination of the ankyrin repeats of p105 is also observed |A4:\*\*1SP3E3| and may be required for proteolytic processing of this protein (58, 60, 61). |A4:\*\*2SP3E2| |A1:\*\*1SP1E2| |A2:\*\*1SP1E2| |A3:\*\*1SP2E2| |A5:\*\*1SP2E2-| |  |  |  |  |  |  |
|  |  |  |  | Annotation | 1SP1E2 1SP1E2 | 1SP1E2 1SP1E2 | 1SP2E2 1SP2E2 | 1SP3E3 2SP3E2 | 1SP2E2- 1SP2E2- |
|  |  |  |  | Evidence | 3 3 | 3 3 | 3 3 | 4 3 | 3 3 |
|  |  |  |  | Focus | 4 4 | 4 4 | 4 4 | 4 4 | 4 4 |
|  |  |  |  | Polarity | 5 5 | 5 5 | 6 6 | 7 7 | 6 6 |
| 57 | 6443 | 9721280\_142 | Fluorescent bands corresponding to Chit108 and Chit67 appeared as a result of the release of 4 MU 4-MU from trimeric [4 MU (GlcNAc)2 [4-MU-(GlcNAc)2 and tetrameric [4 MU (GlcNAc)3 [4-MU-(GlcNAc)3 chitin analogs, |A1:\*\*1SP3E0| |A2:\*\*1MP3E3| |A3:\*\*1MSP3E3| |A4:\*\*1MP3E3| |A5:\*\*1SP3E3+| but not from the dimeric analog 4 MU GlcNAc. 4-MU-GlcNAc. |A1:\*\*2SN3E0| |A2:\*\*1MN3E3| |A3:\*\*2SN3E3| |A4:\*\*2MN3E0| |A5:\*\*2GN3E0| |  |  |  |  |  |  |
|  |  |  |  | Annotation | 1SP3E0 2SN3E0 | 1MP3E3 1MN3E3 | 1MSP3E3 2SN3E3 | 1MP3E3 2MN3E0 | 1SP3E3+ 2GN3E0 |
|  |  |  |  | Evidence | 1 1 | 4 4 | 4 4 | 4 1 | 4 1 |
|  |  |  |  | Focus | 4 4 | 2 2 | 6 4 | 2 2 | 4 1 |
|  |  |  |  | Polarity | 7 1 | 7 1 | 7 1 | 7 1 | 7 1 |
| 58 | 9044 | 11961915\_6 | The process of experiential avoidance mediated the bivariate correlation between anxiety sensitivity and coping-motivated drinking to a greater extent than did the process of alexithymic coping. |A1:\*\*1GP3E0| |A2:\*\*1GP3E0| |A3:\*\*1GP3E3| |A4:\*\*1SP3E3| |A5:\*\*1GP3E0| |  |  |  |  |  |  |
|  |  |  |  | Annotation | 1GP3E0 | 1GP3E0 | 1GP3E3 | 1SP3E3 | 1GP3E0 |
|  |  |  |  | Evidence | 1 | 1 | 4 | 4 | 1 |
|  |  |  |  | Focus | 1 | 1 | 1 | 4 | 1 |
|  |  |  |  | Polarity | 7 | 7 | 7 | 7 | 7 |
| 59 | 528 | 11906793\_4 | GDNF immunoreactivity was localized to RGCs, photoreceptors, and retinal pigment epithelial cells. |A1:\*\*1SP3E3| |A2:\*\*1SP3E3| |A3:\*\*1SP3E3| |A4:\*\*1MP3E3| |A5:\*\*1GP3E1| |  |  |  |  |  |  |
|  |  |  |  | Annotation | 1SP3E3 | 1SP3E3 | 1SP3E3 | 1MP3E3 | 1GP3E1 |
|  |  |  |  | Evidence | 4 | 4 | 4 | 4 | 2 |
|  |  |  |  | Focus | 4 | 4 | 4 | 2 | 1 |
|  |  |  |  | Polarity | 7 | 7 | 7 | 7 | 7 |
| 60 | 262 | 10749878\_244 | Furthermore, both calphostin C and the dominant negative PKC inhibit partially the Wnt-mediated Tcf/ef reporter activation. |A1:\*\*1SN3E3-| |A2:\*\*1SN3E1-| |A3:\*\*1SP3E0| |A4:\*\*1SN2E0-| |A5:\*\*1SP3E1-| |  |  |  |  |  |  |
|  |  |  |  | Annotation | 1SN3E3- | 1SN3E1- | 1SP3E0 | 1SN2E0- | 1SP3E1- |
|  |  |  |  | Evidence | 4 | 2 | 1 | 1 | 2 |
|  |  |  |  | Focus | 4 | 4 | 4 | 4 | 4 |
|  |  |  |  | Polarity | 1 | 1 | 7 | 2 | 7 |
| 61 | 1255 | 12569130\_289 | Either these molecules have different signaling activities or some mechanism may be required to prevent Wnt signaling outside the AER (see below). |A1:\*\*1GP1E3| |A2:\*\*1SP1E1| |A3:\*\*1SP2E3| |A4:\*\*1SP2E3| |A5:\*\*1GP1E3| |  |  |  |  |  |  |
|  |  |  |  | Annotation | 1GP1E3 | 1SP1E1 | 1SP2E3 | 1SP2E3 | 1GP1E3 |
|  |  |  |  | Evidence | 4 | 2 | 4 | 4 | 4 |
|  |  |  |  | Focus | 1 | 4 | 4 | 4 | 1 |
|  |  |  |  | Polarity | 5 | 5 | 6 | 6 | 5 |
| 62 | 4981 | 9802909\_436 | Our data support the view that there are at least three distinct GDI isoforms (Janoueix-Lerosey et al., 1995; Pfeffer et al., 1995). |A1:\*\*1SP3E23| |A2:\*\*1SP3E23| |A3:\*\*1SP3E2| |A4:\*\*1SP3E2| |A5:\*\*1GP3E23| |  |  |  |  |  |  |
|  |  |  |  | Annotation | 1SP3E23 | 1SP3E23 | 1SP3E2 | 1SP3E2 | 1GP3E23 |
|  |  |  |  | Evidence | 3 | 3 | 3 | 3 | 3 |
|  |  |  |  | Focus | 4 | 4 | 4 | 4 | 1 |
|  |  |  |  | Polarity | 7 | 7 | 7 | 7 | 7 |
| 63 | 9760 | 11279051\_1 | The cornified envelope (CE) has a vital role in the barrier function of stratified squamous epithelia. |A1:\*\*1GP3E1| |A2:\*\*1SP3E0| |A3:\*\*1SP3E0| |A4:\*\*1SN3E0| |A5:\*\*1SP3E0| |  |  |  |  |  |  |
|  |  |  |  | Annotation | 1GP3E1 | 1SP3E0 | 1SP3E0 | 1SN3E0 | 1SP3E0 |
|  |  |  |  | Evidence | 2 | 1 | 1 | 1 | 1 |
|  |  |  |  | Focus | 1 | 4 | 4 | 4 | 4 |
|  |  |  |  | Polarity | 7 | 7 | 7 | 1 | 7 |
| 64 | 5896 | 9700168\_45 | Full-length cDNAs coding for chicken cRac1A, cRac1B, cRhoA, and cRhoB polypeptides were obtained by PCR from the corresponding clones in pBluescript KS vector (Malosio et al., 1997), |A4:\*\*1MP3E2| |A5:\*\*1SP3E2| and subcloned into the pGEX-4T-1 vector (Pharmacia Biotech, Uppsala, Sweden). |A4:\*\*2MP3E0| |A1:\*\*1MP3E23| |A2:\*\*1MP3E23| |A3:\*\*1MSP3E3| |A5:\*\*2SP3E3| |  |  |  |  |  |  |
|  |  |  |  | Annotation | 1MP3E23 1MP3E23 | 1MP3E23 1MP3E23 | 1MSP3E3 1MSP3E3 | 1MP3E2 2MP3E0 | 1SP3E2 2SP3E3 |
|  |  |  |  | Evidence | 3 3 | 3 3 | 4 4 | 3 1 | 3 4 |
|  |  |  |  | Focus | 2 2 | 2 2 | 6 6 | 2 2 | 4 4 |
|  |  |  |  | Polarity | 7 7 | 7 7 | 7 7 | 7 7 | 7 7 |
| 65 | 3293 | 10319820\_357 | Such animals derived from a delII germ cell. |A1:\*\*1SP3E3| |A2:\*\*1SP3E0| |A3:\*\*1SP3E0| |A4:\*\*1GP3E0| |A5:\*\*1GP3E1| |  |  |  |  |  |  |
|  |  |  |  | Annotation | 1SP3E3 | 1SP3E0 | 1SP3E0 | 1GP3E0 | 1GP3E1 |
|  |  |  |  | Evidence | 4 | 1 | 1 | 1 | 2 |
|  |  |  |  | Focus | 4 | 4 | 4 | 1 | 1 |
|  |  |  |  | Polarity | 7 | 7 | 7 | 7 | 7 |
| 66 | 1382 | 11889220\_1 | The melanocortin 3 receptor (MC3R) plays a critical role in weight regulation as demonstrated in mouse models. |A1:\*\*1GP3E3| |A2:\*\*1SP3E1| |A3:\*\*1SP3E3| |A4:\*\*1SP3E3| |A5:\*\*1SP3E1| |  |  |  |  |  |  |
|  |  |  |  | Annotation | 1GP3E3 | 1SP3E1 | 1SP3E3 | 1SP3E3 | 1SP3E1 |
|  |  |  |  | Evidence | 4 | 2 | 4 | 4 | 2 |
|  |  |  |  | Focus | 1 | 4 | 4 | 4 | 4 |
|  |  |  |  | Polarity | 7 | 7 | 7 | 7 | 7 |
| 67 | 2929 | 11585828\_20 | These observations, when considered together, are consistent with B56alpha binding to the APC ARM domain and carrying APC into the nucleus. |A1:\*\*1SP3E3| |A2:\*\*1SP3E3| |A3:\*\*1SP3E0| |A4:\*\*1SP3E3| |A5:\*\*1SP2E1| |  |  |  |  |  |  |
|  |  |  |  | Annotation | 1SP3E3 | 1SP3E3 | 1SP3E0 | 1SP3E3 | 1SP2E1 |
|  |  |  |  | Evidence | 4 | 4 | 1 | 4 | 2 |
|  |  |  |  | Focus | 4 | 4 | 4 | 4 | 4 |
|  |  |  |  | Polarity | 7 | 7 | 7 | 7 | 6 |
| 68 | 7764 | 11083814\_29 | These findings suggest that neutrophil-derived cytokines and chemokines might represent an early event during the course of meningitis. |A1:\*\*1SP1E3| |A2:\*\*1SP2E3| |A3:\*\*1SP2E0| |A4:\*\*1SP2E1| |A5:\*\*1SP1E1| |  |  |  |  |  |  |
|  |  |  |  | Annotation | 1SP1E3 | 1SP2E3 | 1SP2E0 | 1SP2E1 | 1SP1E1 |
|  |  |  |  | Evidence | 4 | 4 | 1 | 2 | 2 |
|  |  |  |  | Focus | 4 | 4 | 4 | 4 | 4 |
|  |  |  |  | Polarity | 5 | 6 | 6 | 6 | 5 |
| 69 | 3556 | 10618246\_208 | In our study we performed correspondence analyses, which took into account both the presence of a band and its relative staining intensity. |A1:\*\*1MP3E3| |A2:\*\*1SP3E3| |A3:\*\*1MSP3E3| |A4:\*\*1SP3E3| |A5:\*\*1GP3E3| |  |  |  |  |  |  |
|  |  |  |  | Annotation | 1MP3E3 | 1SP3E3 | 1MSP3E3 | 1SP3E3 | 1GP3E3 |
|  |  |  |  | Evidence | 4 | 4 | 4 | 4 | 4 |
|  |  |  |  | Focus | 2 | 4 | 6 | 4 | 1 |
|  |  |  |  | Polarity | 7 | 7 | 7 | 7 | 7 |
| 70 | 8105 | 11916423\_1 | Semiclassical molecular dynamics simulations have been combined with quantum chemistry calculations |A5:\*\*1MP3E1| to provide detailed modeling of the methane and ethane hydroxylation reactions catalyzed by the hydroxylase enzymes of the soluble methane monooxygenase system. |A5:\*\*2GP3E0| |A1:\*\*1GP3E0| |A2:\*\*1GP3E0| |A3:\*\*1SP3E0| |A4:\*\*1MP3E3| |  |  |  |  |  |  |
|  |  |  |  | Annotation | 1GP3E0 1GP3E0 | 1GP3E0 1GP3E0 | 1SP3E0 1SP3E0 | 1MP3E3 1MP3E3 | 1MP3E1 2GP3E0 |
|  |  |  |  | Evidence | 1 1 | 1 1 | 1 1 | 4 4 | 2 1 |
|  |  |  |  | Focus | 1 1 | 1 1 | 4 4 | 2 2 | 2 1 |
|  |  |  |  | Polarity | 7 7 | 7 7 | 7 7 | 7 7 | 7 7 |
| 71 | 1068 | 12069952\_5 | In primate fat cells, NP-induced lipolysis involved a cGMP-dependent pathway. |A1:\*\*1SP3E0| |A2:\*\*1SP3E0| |A3:\*\*1SP3E0| |A4:\*\*1SP3E0| |A5:\*\*1SP3E0| |  |  |  |  |  |  |
|  |  |  |  | Annotation | 1SP3E0 | 1SP3E0 | 1SP3E0 | 1SP3E0 | 1SP3E0 |
|  |  |  |  | Evidence | 1 | 1 | 1 | 1 | 1 |
|  |  |  |  | Focus | 4 | 4 | 4 | 4 | 4 |
|  |  |  |  | Polarity | 7 | 7 | 7 | 7 | 7 |
| 72 | 7723 | 9548972\_97 | At each downward step in stringency, the increase in sensitivity is primarily the result of inclusion of new ESTs. |A1:\*\*1GP3E0+| |A2:\*\*1GP3E0+| |A3:\*\*1SP3E0+| |A4:\*\*1SP3E3+| |A5:\*\*1GP3E0| |  |  |  |  |  |  |
|  |  |  |  | Annotation | 1GP3E0+ | 1GP3E0+ | 1SP3E0+ | 1SP3E3+ | 1GP3E0 |
|  |  |  |  | Evidence | 1 | 1 | 1 | 4 | 1 |
|  |  |  |  | Focus | 1 | 1 | 4 | 4 | 1 |
|  |  |  |  | Polarity | 7 | 7 | 7 | 7 | 7 |
| 73 | 6488 | 11149951\_26 | Several studies have demonstrated the increased binding of dopamine antagonists in lymphocytes of schizophrenic patients as compared with healthy individuals (11, 12). |A1:\*\*1SP3E2+| |A2:\*\*1SP3E2+| |A3:\*\*1SP3E2| |A4:\*\*1SP3E2+| |A5:\*\*1SP3E2+| |  |  |  |  |  |  |
|  |  |  |  | Annotation | 1SP3E2+ | 1SP3E2+ | 1SP3E2 | 1SP3E2+ | 1SP3E2+ |
|  |  |  |  | Evidence | 3 | 3 | 3 | 3 | 3 |
|  |  |  |  | Focus | 4 | 4 | 4 | 4 | 4 |
|  |  |  |  | Polarity | 7 | 7 | 7 | 7 | 7 |
| 74 | 5290 | 9827803\_244 | Nearly all tissues and organs can develop normally or near normally in the complete absence of the entire family of v integrins, comprising five different adhesion receptors with a multiplicity of ECM ligands. |A1:\*\*1GP2E0| |A2:\*\*1SP1E0| |A3:\*\*1SP1E0| |A4:\*\*1SP2E3| |A5:\*\*1GP3E0| |  |  |  |  |  |  |
|  |  |  |  | Annotation | 1GP2E0 | 1SP1E0 | 1SP1E0 | 1SP2E3 | 1GP3E0 |
|  |  |  |  | Evidence | 1 | 1 | 1 | 4 | 1 |
|  |  |  |  | Focus | 1 | 4 | 4 | 4 | 1 |
|  |  |  |  | Polarity | 6 | 5 | 5 | 6 | 7 |
| 75 | 4468 | 7814421\_42 | In other tumor types, such as renal cell carcinoma, significant fibrin was observed in tumor stroma despite the absence of demonstrable tissue factor antigen on these tumor cells ( ). |A1:\*\*1GP3E23| |A2:\*\*1SP3E2| |A3:\*\*1SP1E3| |A4:\*\*1SP2E3| |A5:\*\*1SP2E2| |  |  |  |  |  |  |
|  |  |  |  | Annotation | 1GP3E23 | 1SP3E2 | 1SP1E3 | 1SP2E3 | 1SP2E2 |
|  |  |  |  | Evidence | 3 | 3 | 4 | 4 | 3 |
|  |  |  |  | Focus | 1 | 4 | 4 | 4 | 4 |
|  |  |  |  | Polarity | 7 | 7 | 5 | 6 | 6 |
| 76 | 8205 | 10421629\_4 | Wnt signaling stabilizes beta-catenin, |A3:\*\*1SP3E0| |A5:\*\*1GP3EO| but it was not clear whether and how Wnt signaling regulates the beta-catenin complex. |A3:\*\*2SN0E0| |A1:\*\*1SP0E3| |A2:\*\*1SN0E0| |A4:\*\*1SN2E0| |A5:\*\*2SN0E0| |  |  |  |  |  |  |
|  |  |  |  | Annotation | 1SP0E3 1SP0E3 | 1SN0E0 1SN0E0 | 1SP3E0 2SN0E0 | 1SN2E0 1SN2E0 | 2SN0E0 2SN0E0 |
|  |  |  |  | Evidence | 4 4 | 1 1 | 1 1 | 1 1 | 1 1 |
|  |  |  |  | Focus | 4 4 | 4 4 | 4 4 | 4 4 | 4 4 |
|  |  |  |  | Polarity | 4 4 | 4 4 | 7 4 | 2 2 | 4 4 |
| 77 | 593 | 9797283\_40 | The clinical strains Enterobacter cloacae RYC70770 and Escherichia hermannii RYC78330 were provided by the Hospital Ramon y Cajal, Madrid, Spain. |A1:\*\*1GP3E3| |A2:\*\*1GP3E3| |A3:\*\*1SP3E3| |A4:\*\*1SP3E3| |A5:\*\*1GP3E0| |  |  |  |  |  |  |
|  |  |  |  | Annotation | 1GP3E3 | 1GP3E3 | 1SP3E3 | 1SP3E3 | 1GP3E0 |
|  |  |  |  | Evidence | 4 | 4 | 4 | 4 | 1 |
|  |  |  |  | Focus | 1 | 1 | 4 | 4 | 1 |
|  |  |  |  | Polarity | 7 | 7 | 7 | 7 | 7 |
| 78 | 3048 | 12702652\_170 | As a result, Sfrps can be used to inhibit a broad range of Wnt signals, |A4:\*\*1SP3E3| including Wnt7a (Hall et al., 2000 ; Yoshino et al., 2001 ; Bergwitz et al., 2001 ). |A1:\*\*1SN3E2| |A2:\*\*1SP3E2-| |A3:\*\*1SP2E2-| |A4:\*\*2SP3E2| |A5:\*\*1SP3E2| |  |  |  |  |  |  |
|  |  |  |  | Annotation | 1SN3E2 1SN3E2 | 1SP3E2- 1SP3E2- | 1SP2E2- 1SP2E2- | 1SP3E3 2SP3E2 | 1SP3E2 1SP3E2 |
|  |  |  |  | Evidence | 3 3 | 3 3 | 3 3 | 4 3 | 3 3 |
|  |  |  |  | Focus | 4 4 | 4 4 | 4 4 | 4 4 | 4 4 |
|  |  |  |  | Polarity | 1 1 | 7 7 | 6 6 | 7 7 | 7 7 |
| 79 | 2485 | 9777950\_1 | Occult aspects of tumor proliferation are likely recorded genetically as their microsatellite (MS) loci become polymorphic. |A1:\*\*1GP3E1| |A2:\*\*1SP1E0| |A3:\*\*1SP2E0| |A4:\*\*1SP2E0| |A5:\*\*1GP2E0| |  |  |  |  |  |  |
|  |  |  |  | Annotation | 1GP3E1 | 1SP1E0 | 1SP2E0 | 1SP2E0 | 1GP2E0 |
|  |  |  |  | Evidence | 2 | 1 | 1 | 1 | 1 |
|  |  |  |  | Focus | 1 | 4 | 4 | 4 | 1 |
|  |  |  |  | Polarity | 7 | 5 | 6 | 6 | 6 |
| 80 | 5690 | 11867725\_20 | Sulcal/gyral anatomy shows marked variation between individuals |A4:\*\*1SP2E3| and is dissimilar even within pairs of healthy identical or monozygotic (MZ) co-twins (20, 21). |A4:\*\*2SP3E2| |A1:\*\*1GP3E2| |A2:\*\*1SN3E23| |A3:\*\*1SP3E2| |A5:\*\*1GP3E2| |  |  |  |  |  |  |
|  |  |  |  | Annotation | 1GP3E2 1GP3E2 | 1SN3E23 1SN3E23 | 1SP3E2 1SP3E2 | 1SP2E3 2SP3E2 | 1GP3E2 1GP3E2 |
|  |  |  |  | Evidence | 3 3 | 3 3 | 3 3 | 4 3 | 3 3 |
|  |  |  |  | Focus | 1 1 | 4 4 | 4 4 | 4 4 | 1 1 |
|  |  |  |  | Polarity | 7 7 | 1 1 | 7 7 | 6 7 | 7 7 |
| 81 | 4013 | 11133465\_64 | One unit of the PLA-degrading activity was defined as a 1-U decrease in absorbance at 630 nm per min under the assay conditions described. |A1:\*\*1SP3E3-| |A2:\*\*1SP3E3| |A3:\*\*1SP3E3| |A4:\*\*1SP3E3| |A5:\*\*1MP3E1-| |  |  |  |  |  |  |
|  |  |  |  | Annotation | 1SP3E3- | 1SP3E3 | 1SP3E3 | 1SP3E3 | 1MP3E1- |
|  |  |  |  | Evidence | 4 | 4 | 4 | 4 | 2 |
|  |  |  |  | Focus | 4 | 4 | 4 | 4 | 2 |
|  |  |  |  | Polarity | 7 | 7 | 7 | 7 | 7 |
| 82 | 2229 | 10380924\_217 | We have shown that rather than binding stably with POP-1, WRM-1 is required for POP-1 phosphorylation. |A1:\*\*1SP3E3| |A2:\*\*1SP3E3| |A3:\*\*1SP3E3| |A4:\*\*1SP3E3| |A5:\*\*1GP3E3| |  |  |  |  |  |  |
|  |  |  |  | Annotation | 1SP3E3 | 1SP3E3 | 1SP3E3 | 1SP3E3 | 1GP3E3 |
|  |  |  |  | Evidence | 4 | 4 | 4 | 4 | 4 |
|  |  |  |  | Focus | 4 | 4 | 4 | 4 | 1 |
|  |  |  |  | Polarity | 7 | 7 | 7 | 7 | 7 |
| 83 | 3232 | 9844016\_10 | In the dorsolateral prefrontal cortex, functional hypoactivity (3) in schizophrenia is associated with defects in a number of neurotransmitter systems ( 4-11), |A4:\*\*1SP3E2| including the inhibitory gamma-amino butyrate (GABA)ergic system. |A4:\*\*2SP3E0| |A1:\*\*1GP3E2| |A2:\*\*1SP3E2| |A3:\*\*1SP3E2| |A5:\*\*1GP3E2| |  |  |  |  |  |  |
|  |  |  |  | Annotation | 1GP3E2 1GP3E2 | 1SP3E2 1SP3E2 | 1SP3E2 1SP3E2 | 1SP3E2 2SP3E0 | 1GP3E2 1GP3E2 |
|  |  |  |  | Evidence | 3 3 | 3 3 | 3 3 | 3 1 | 3 3 |
|  |  |  |  | Focus | 1 1 | 4 4 | 4 4 | 4 4 | 1 1 |
|  |  |  |  | Polarity | 7 7 | 7 7 | 7 7 | 7 7 | 7 7 |
| 84 | 6292 | 12651889\_284 | Wnt signaling polarizes an early C. elegans blastomere to distinguish endoderm and mesoderm. |A1:\*\*1GP3E0| |A2:\*\*1SP3E0| |A3:\*\*1SP3E0| |A4:\*\*1SP3E0| |A5:\*\*1GP3E0| |  |  |  |  |  |  |
|  |  |  |  | Annotation | 1GP3E0 | 1SP3E0 | 1SP3E0 | 1SP3E0 | 1GP3E0 |
|  |  |  |  | Evidence | 1 | 1 | 1 | 1 | 1 |
|  |  |  |  | Focus | 1 | 4 | 4 | 4 | 1 |
|  |  |  |  | Polarity | 7 | 7 | 7 | 7 | 7 |
| 85 | 9395 | 9508000\_76 | Alternatively, proteins were electroblotted onto nitrocellulose for immunoblot analysis with IgG-C7 and IgG-HL1 or 45Ca blot analysis. |A1:\*\*1MP3E3| |A2:\*\*1MP3E3| |A3:\*\*1MP3E3| |A4:\*\*1MP3E3| |A5:\*\*1MP3E3| |  |  |  |  |  |  |
|  |  |  |  | Annotation | 1MP3E3 | 1MP3E3 | 1MP3E3 | 1MP3E3 | 1MP3E3 |
|  |  |  |  | Evidence | 4 | 4 | 4 | 4 | 4 |
|  |  |  |  | Focus | 2 | 2 | 2 | 2 | 2 |
|  |  |  |  | Polarity | 7 | 7 | 7 | 7 | 7 |
| 86 | 678 | 12079534\_1 | PURPOSE: To characterize the role of various cellular damagesensing, processing and survival genes in the in-vitro radiosensitivity of haemopoietic colony-forming cells. |A1:\*\*1GP2E0| |A2:\*\*1SP2E0| |A3:\*\*1SP3E0| |A4:\*\*1MP3E3| |A5:\*\*1GP0E0| |  |  |  |  |  |  |
|  |  |  |  | Annotation | 1GP2E0 | 1SP2E0 | 1SP3E0 | 1MP3E3 | 1GP0E0 |
|  |  |  |  | Evidence | 1 | 1 | 1 | 4 | 1 |
|  |  |  |  | Focus | 1 | 4 | 4 | 2 | 1 |
|  |  |  |  | Polarity | 6 | 6 | 7 | 7 | 4 |
| 87 | 5294 | 9464989\_145 | We have examined the structure of the paranode and the node of Ranvier in mice incapable of synthesizing the myelin galactolipids GalC and sulfatide. |A1:\*\*1GN3E1| |A2:\*\*1SN3E1| |A3:\*\*1SP3E3| |A4:\*\*1SP3E3| |A5:\*\*1GP3E3| |  |  |  |  |  |  |
|  |  |  |  | Annotation | 1GN3E1 | 1SN3E1 | 1SP3E3 | 1SP3E3 | 1GP3E3 |
|  |  |  |  | Evidence | 2 | 2 | 4 | 4 | 4 |
|  |  |  |  | Focus | 1 | 4 | 4 | 4 | 1 |
|  |  |  |  | Polarity | 1 | 1 | 7 | 7 | 7 |
| 88 | 278 | 11976332\_58 | All these peptides were found to serve as substrates, their K m values ranging from 10 to 40 muM (Table III). |A1:\*\*1SP3E3| |A2:\*\*1SP3E3| |A3:\*\*1SP3E3| |A4:\*\*1SP3E3| |A5:\*\*1GP3E3| |  |  |  |  |  |  |
|  |  |  |  | Annotation | 1SP3E3 | 1SP3E3 | 1SP3E3 | 1SP3E3 | 1GP3E3 |
|  |  |  |  | Evidence | 4 | 4 | 4 | 4 | 4 |
|  |  |  |  | Focus | 4 | 4 | 4 | 4 | 1 |
|  |  |  |  | Polarity | 7 | 7 | 7 | 7 | 7 |
| 89 | 2041 | 9230313\_36 | Thus, analysis of the Fu locus has identified a novel inhibitor of the Wnt signaling pathway |A1:\*\*1SN3E1| |A2:\*\*1SN3E1| |A3:\*\*1SP3E3| |A4:\*\*1SP3E3| |A5:\*\*1GP3E1| and suggests that the same pathway regulates an early step in embryonic axis formation in mammals and amphibians. |A1:\*\*2SP3E1| |A2:\*\*2SP3E1| |A3:\*\*2SP2E0| |A4:\*\*2SP3E1| |A5:\*\*2GP2E0| |  |  |  |  |  |  |
|  |  |  |  | Annotation | 1SN3E1 2SP3E1 | 1SN3E1 2SP3E1 | 1SP3E3 2SP2E0 | 1SP3E3 2SP3E1 | 1GP3E1 2GP2E0 |
|  |  |  |  | Evidence | 2 2 | 2 2 | 4 1 | 4 2 | 2 1 |
|  |  |  |  | Focus | 4 4 | 4 4 | 4 4 | 4 4 | 1 1 |
|  |  |  |  | Polarity | 1 7 | 1 7 | 7 6 | 7 7 | 7 6 |
| 90 | 9828 | 11679314\_117 | The methyl ester in the organic layer was analyzed by using GC. |A1:\*\*1MP3E3| |A2:\*\*1SP3E3| |A3:\*\*1MP3E3| |A4:\*\*1SP3E3| |A5:\*\*1GP3E1| |  |  |  |  |  |  |
|  |  |  |  | Annotation | 1MP3E3 | 1SP3E3 | 1MP3E3 | 1SP3E3 | 1GP3E1 |
|  |  |  |  | Evidence | 4 | 4 | 4 | 4 | 2 |
|  |  |  |  | Focus | 2 | 4 | 2 | 4 | 1 |
|  |  |  |  | Polarity | 7 | 7 | 7 | 7 | 7 |
| 91 | 6672 | 10801471\_232 | In general, anti idiotype anti-idiotype RNA aptamers isolated with an anti ligand anti-ligand antibody |A4:\*\*1SP3E0| can apparently mimic faithfully some of the structural and functional properties of a protein domain in vivo and in vitro [23,30,34]. |A4:\*\*2MP2E2| |A1:\*\*1GP2E2| |A2:\*\*1SP2E2| |A3:\*\*1MSP3E2| |A5:\*\*1SP3E2| |  |  |  |  |  |  |
|  |  |  |  | Annotation | 1GP2E2 1GP2E2 | 1SP2E2 1SP2E2 | 1MSP3E2 1MSP3E2 | 1SP3E0 2MP2E2 | 1SP3E2 1SP3E2 |
|  |  |  |  | Evidence | 3 3 | 3 3 | 3 3 | 1 3 | 3 3 |
|  |  |  |  | Focus | 1 1 | 4 4 | 6 6 | 4 2 | 4 4 |
|  |  |  |  | Polarity | 6 6 | 6 6 | 7 7 | 7 6 | 7 7 |
| 92 | 9825 | 11333250\_111 | No significant homology to GenBank sequences was observed for the first 1.5 kb. |A1:\*\*1SN3E3| |A2:\*\*1SN3E3| |A3:\*\*1SN3E3| |A4:\*\*1GN2E0| |A5:\*\*1GN3E1| |  |  |  |  |  |  |
|  |  |  |  | Annotation | 1SN3E3 | 1SN3E3 | 1SN3E3 | 1GN2E0 | 1GN3E1 |
|  |  |  |  | Evidence | 4 | 4 | 4 | 1 | 2 |
|  |  |  |  | Focus | 4 | 4 | 4 | 1 | 1 |
|  |  |  |  | Polarity | 1 | 1 | 1 | 2 | 1 |
| 93 | 9094 | 11349131\_16 | The interaction-trap assay was performed as described previously ( ). |A1:\*\*1MP3E2| |A2:\*\*1MP3E2| |A3:\*\*1MP3E3| |A4:\*\*1MP3E3| |A5:\*\*1GMP3E2| Numbers in parentheses are the amino acid residues of p45SKP2 ( A) or CDK2 ( B) fused to the LexA DNA-binding domain. |A1:\*\*2SP3E3| |A2:\*\*2SP3E3| |A3:\*\*1SP3E0| |A4:\*\*2SP3E0| |A5:\*\*2GP3E0| |  |  |  |  |  |  |
|  |  |  |  | Annotation | 1MP3E2 2SP3E3 | 1MP3E2 2SP3E3 | 1MP3E3 1SP3E0 | 1MP3E3 2SP3E0 | 1GMP3E2 2GP3E0 |
|  |  |  |  | Evidence | 3 4 | 3 4 | 4 1 | 4 1 | 3 1 |
|  |  |  |  | Focus | 2 4 | 2 4 | 2 4 | 2 4 | 3 1 |
|  |  |  |  | Polarity | 7 7 | 7 7 | 7 7 | 7 7 | 7 7 |
| 94 | 3139 | 10496918\_96 | Among pregnant women, primiparae with noninfected placentas have the higher likelihood not to have been infected with placental parasites. |A1:\*\*1GP1E1| |A2:\*\*1SP2E0| |A3:\*\*1SN2E0| |A4:\*\*1SN2E3+| |A5:\*\*1GN2E0| |  |  |  |  |  |  |
|  |  |  |  | Annotation | 1GP1E1 | 1SP2E0 | 1SN2E0 | 1SN2E3+ | 1GN2E0 |
|  |  |  |  | Evidence | 2 | 1 | 1 | 4 | 1 |
|  |  |  |  | Focus | 1 | 4 | 4 | 4 | 1 |
|  |  |  |  | Polarity | 5 | 6 | 2 | 2 | 2 |
| 95 | 8093 | 11533249\_31 | A genomic DNA fragment of the murine stat3 gene was isolated from a lambda fix II 129/sv mouse genomic library (Stratagene) |A3:\*\*1SP3E3| |A4:\*\*1SP3E3| using a 2.2-kb genomic fragment of the stat3 promoter as a probe (16). |A3:\*\*1SP3E2| |A1:\*\*1SP3E23| |A2:\*\*1SP3E23| |A4:\*\*2SP3E2| |A5:\*\*1MP3E2| |  |  |  |  |  |  |
|  |  |  |  | Annotation | 1SP3E23 1SP3E23 | 1SP3E23 1SP3E23 | 1SP3E3 1SP3E2 | 1SP3E3 2SP3E2 | 1MP3E2 1MP3E2 |
|  |  |  |  | Evidence | 3 3 | 3 3 | 4 3 | 4 3 | 3 3 |
|  |  |  |  | Focus | 4 4 | 4 4 | 4 4 | 4 4 | 2 2 |
|  |  |  |  | Polarity | 7 7 | 7 7 | 7 7 | 7 7 | 7 7 |
| 96 | 6419 | 10893270\_256 | Wnt proteins increased beta catenin beta-catenin levels in cardiac myocytes and fibroblasts. |A1:\*\*1SP3E3+| |A2:\*\*1SP3E0+| |A3:\*\*1SP3E0+| |A4:\*\*1SP3E0+| |A5:\*\*1GP3E1+| |  |  |  |  |  |  |
|  |  |  |  | Annotation | 1SP3E3+ | 1SP3E0+ | 1SP3E0+ | 1SP3E0+ | 1GP3E1+ |
|  |  |  |  | Evidence | 4 | 1 | 1 | 1 | 2 |
|  |  |  |  | Focus | 4 | 4 | 4 | 4 | 1 |
|  |  |  |  | Polarity | 7 | 7 | 7 | 7 | 7 |
| 97 | 9253 | 9107167\_6 | Lymphocyte activation was studied at day 1 of hospital admission and after 7, 15, 30, 60, and 90 days. |A1:\*\*1SP3E3| |A2:\*\*1SP3E3| |A3:\*\*1SP3E3+| |A4:\*\*1SP3E3| |A5:\*\*1MP3E1| |  |  |  |  |  |  |
|  |  |  |  | Annotation | 1SP3E3 | 1SP3E3 | 1SP3E3+ | 1SP3E3 | 1MP3E1 |
|  |  |  |  | Evidence | 4 | 4 | 4 | 4 | 2 |
|  |  |  |  | Focus | 4 | 4 | 4 | 4 | 2 |
|  |  |  |  | Polarity | 7 | 7 | 7 | 7 | 7 |
| 98 | 6308 | 12361574\_5 | The relative position of the chromosomes involved in these translocations is conserved in normal splenocytes. |A1:\*\*1SP3E3| |A2:\*\*1SP3E0| |A3:\*\*1SP3E0| |A4:\*\*1SP2E0| |A5:\*\*1GP3E1| |  |  |  |  |  |  |
|  |  |  |  | Annotation | 1SP3E3 | 1SP3E0 | 1SP3E0 | 1SP2E0 | 1GP3E1 |
|  |  |  |  | Evidence | 4 | 1 | 1 | 1 | 2 |
|  |  |  |  | Focus | 4 | 4 | 4 | 4 | 1 |
|  |  |  |  | Polarity | 7 | 7 | 7 | 6 | 7 |
| 99 | 4508 | 9547360\_141 | Before proceeding with a detailed examination of the PMA effect, |A5:\*\*1GP3E0| it was necessary to confirm that the effect was not a clonal artifact unique to the P5A11 cell line. |A5:\*\*2GN1E0| |A1:\*\*1SP3E3| |A2:\*\*1SP3E3| |A3:\*\*1SN2E0| |A4:\*\*1SN3E3| |  |  |  |  |  |  |
|  |  |  |  | Annotation | 1SP3E3 1SP3E3 | 1SP3E3 1SP3E3 | 1SN2E0 1SN2E0 | 1SN3E3 1SN3E3 | 1GP3E0 2GN1E0 |
|  |  |  |  | Evidence | 4 4 | 4 4 | 1 1 | 4 4 | 1 1 |
|  |  |  |  | Focus | 4 4 | 4 4 | 4 4 | 4 4 | 1 1 |
|  |  |  |  | Polarity | 7 7 | 7 7 | 2 2 | 1 1 | 7 3 |
| 100 | 79 | 9407023\_209 | The genetic evidence that Wnt proteins require Fz proteins for signaling, |A5:\*\*1GP3E1| although lacking in flies, |A5:\*\*2GP3E1-| is accumulating in C. elegans (Table 3), |A5:\*\*3GP3E3+| |A3:\*\*1SPE2| |A4:\*\*1SP3E3| though the story is a little complicated. |A5:\*\*4GP1E0| |A1:\*\*1SP2E3| |A2:\*\*1SP2E3| |A3:\*\*1GP0E0| |A4:\*\*2GP3E0| |  |  |  |  |  |  |
|  |  |  |  | Annotation | 1SP2E3 1SP2E3 1SP2E3 1SP2E3 | 1SP2E3 1SP2E3 1SP2E3 1SP2E3 | 1GP0E0 1GP0E0 1GP0E0 1GP0E0 | 1SP3E3 1SP3E3 1SP3E3 2GP3E0 | 1GP3E1 2GP3E1- 3GP3E3+ 4GP1E0 |
|  |  |  |  | Evidence | 4 4 4 4 | 4 4 4 4 | 1 1 1 1 | 4 4 4 1 | 2 2 4 1 |
|  |  |  |  | Focus | 4 4 4 4 | 4 4 4 4 | 1 1 1 1 | 4 4 4 1 | 1 1 1 1 |
|  |  |  |  | Polarity | 6 6 6 6 | 6 6 6 6 | 4 4 4 4 | 7 7 7 7 | 7 7 7 5 |
| 101 | 4024 | 9502810\_68 | The lesion was defined as the area that included axons that were completely and partially demyelinated |A4:\*\*1SP3E0| |A5:\*\*1GP3E3-| as well as completely and partially remyelinated (Fig. 1). |A4:\*\*2SP3E3| |A1:\*\*1SP3E3| |A2:\*\*1SP3E3| |A3:\*\*1SP2E3| |A5:\*\*2GP3E3+| |  |  |  |  |  |  |
|  |  |  |  | Annotation | 1SP3E3 1SP3E3 | 1SP3E3 1SP3E3 | 1SP2E3 1SP2E3 | 1SP3E0 2SP3E3 | 1GP3E3- 2GP3E3+ |
|  |  |  |  | Evidence | 4 4 | 4 4 | 4 4 | 1 4 | 4 4 |
|  |  |  |  | Focus | 4 4 | 4 4 | 4 4 | 4 4 | 1 1 |
|  |  |  |  | Polarity | 7 7 | 7 7 | 6 6 | 7 7 | 7 7 |
| 102 | 7073 | 11136978\_12 | Class I enzymes are acutely activated by a variety of cell-surface receptors, |A1:\*\*1SP3E3+| |A2:\*\*1SP3E3+| and are responsible for synthesis of intracellular phosphatidylinositol (3, 4, 5)-trisphosphate (PtdIns (3, 4, 5) P3). |A1:\*\*2SP3E3| |A2:\*\*2SP3E3| |A3:\*\*1SP2E0+| |A4:\*\*1SP3E3| |A5:\*\*1SP3E0| |  |  |  |  |  |  |
|  |  |  |  | Annotation | 1SP3E3+ 2SP3E3 | 1SP3E3+ 2SP3E3 | 1SP2E0+ 1SP2E0+ | 1SP3E3 1SP3E3 | 1SP3E0 1SP3E0 |
|  |  |  |  | Evidence | 4 4 | 4 4 | 1 1 | 4 4 | 1 1 |
|  |  |  |  | Focus | 4 4 | 4 4 | 4 4 | 4 4 | 4 4 |
|  |  |  |  | Polarity | 7 7 | 7 7 | 6 6 | 7 7 | 7 7 |
| 103 | 6613 | 11679341\_106 | If one assumes that viral OB in only the top 0.2 cm of soil are subject to possible transport by precipitation, |A5:\*\*1GP1E0| then the proportion of OB transported to foliage of cotton plants still ranged only from 6.2 x 10 6 to 2.5 x 10 5. |A5:\*\*2GP3E3| |A1:\*\*1GP1E0| |A2:\*\*1SP1E0| |A3:\*\*1SP2E0| |A4:\*\*1SP2E0| |  |  |  |  |  |  |
|  |  |  |  | Annotation | 1GP1E0 1GP1E0 | 1SP1E0 1SP1E0 | 1SP2E0 1SP2E0 | 1SP2E0 1SP2E0 | 1GP1E0 2GP3E3 |
|  |  |  |  | Evidence | 1 1 | 1 1 | 1 1 | 1 1 | 1 4 |
|  |  |  |  | Focus | 1 1 | 4 4 | 4 4 | 4 4 | 1 1 |
|  |  |  |  | Polarity | 5 5 | 5 5 | 6 6 | 6 6 | 5 7 |
| 104 | 8268 | 10330411\_361 | These motifs are implicated in the binding of paxillin to vinculin, FAK, and the E6 oncoprotein from papillomavirus (Brown et al., 1996; Tong et al., 1997 ; Vande Pol et al., 1998). |A3:\*\*1SP3E2| |A1:\*\*1SP3E2| |A2:\*\*1SP3E2| |A4:\*\*1SP3E2| |A5:\*\*1GP3E2| |  |  |  |  |  |  |
|  |  |  |  | Annotation | 1SP3E2 | 1SP3E2 | 1SP3E2 | 1SP3E2 | 1GP3E2 |
|  |  |  |  | Evidence | 3 | 3 | 3 | 3 | 3 |
|  |  |  |  | Focus | 4 | 4 | 4 | 4 | 1 |
|  |  |  |  | Polarity | 7 | 7 | 7 | 7 | 7 |
| 105 | 4504 | 12185709\_1 | Current views on efficacy and safety of magnesium sulfate treatment in obstetrics are presented. |A1:\*\*1GP3E3| |A2:\*\*1SP3E1| |A3:\*\*1SP3E3| |A4:\*\*1GP3E0| |A5:\*\*1GP3E3| |  |  |  |  |  |  |
|  |  |  |  | Annotation | 1GP3E3 | 1SP3E1 | 1SP3E3 | 1GP3E0 | 1GP3E3 |
|  |  |  |  | Evidence | 4 | 2 | 4 | 1 | 4 |
|  |  |  |  | Focus | 1 | 4 | 4 | 1 | 1 |
|  |  |  |  | Polarity | 7 | 7 | 7 | 7 | 7 |
| 106 | 9155 | 11555643\_1 | GROs are a new type of antiproliferative oligonucleotide with considerable potential as therapeutic agents for cancer. |A1:\*\*1GP3E0| |A2:\*\*1SP3E0| |A3:\*\*1SP2E0| |A4:\*\*1SP3E0| |A5:\*\*1GP2E0| |  |  |  |  |  |  |
|  |  |  |  | Annotation | 1GP3E0 | 1SP3E0 | 1SP2E0 | 1SP3E0 | 1GP2E0 |
|  |  |  |  | Evidence | 1 | 1 | 1 | 1 | 1 |
|  |  |  |  | Focus | 1 | 4 | 4 | 4 | 1 |
|  |  |  |  | Polarity | 7 | 7 | 6 | 7 | 6 |
| 107 | 5888 | 11279203\_93 | Analysis of these data revealed three major differences in the translocation profiles (Fig. 8 B). |A1:\*\*1SP3E3| |A2:\*\*1SP3E3| |A3:\*\*1SP3E3| |A4:\*\*1GP3E3| |A5:\*\*1GP3E3| |  |  |  |  |  |  |
|  |  |  |  | Annotation | 1SP3E3 | 1SP3E3 | 1SP3E3 | 1GP3E3 | 1GP3E3 |
|  |  |  |  | Evidence | 4 | 4 | 4 | 4 | 4 |
|  |  |  |  | Focus | 4 | 4 | 4 | 1 | 1 |
|  |  |  |  | Polarity | 7 | 7 | 7 | 7 | 7 |
| 108 | 9847 | 10026183\_146 | Thus, increases of nuclear cytochrome c-GFP reflected increases of cytosolic cytochrome c-GFP. |A1:\*\*1SP3E3+| |A2:\*\*1SP3E1+| |A3:\*\*1SP2E0| |A4:\*\*1SP3E0+| |A5:\*\*1SP3E1+| |  |  |  |  |  |  |
|  |  |  |  | Annotation | 1SP3E3+ | 1SP3E1+ | 1SP2E0 | 1SP3E0+ | 1SP3E1+ |
|  |  |  |  | Evidence | 4 | 2 | 1 | 1 | 2 |
|  |  |  |  | Focus | 4 | 4 | 4 | 4 | 4 |
|  |  |  |  | Polarity | 7 | 7 | 6 | 7 | 7 |
| 109 | 4884 | 10985388\_13 | Thus, MEI-S332 function is necessary for normal minichromosome transmission. |A1:\*\*1GP3E3| |A2:\*\*1GP3E0| |A3:\*\*1SP3E0| |A4:\*\*1SP3E0| |A5:\*\*1GP3E0| |  |  |  |  |  |  |
|  |  |  |  | Annotation | 1GP3E3 | 1GP3E0 | 1SP3E0 | 1SP3E0 | 1GP3E0 |
|  |  |  |  | Evidence | 4 | 1 | 1 | 1 | 1 |
|  |  |  |  | Focus | 1 | 1 | 4 | 4 | 1 |
|  |  |  |  | Polarity | 7 | 7 | 7 | 7 | 7 |
| 110 | 1601 | 9472043\_229 | Received for publication 22 August 1997 and in revised form 16 November 1997. |A1:\*\*1GP3E3| |A2:\*\*1GP3E3| |A3:\*\*1GP3E0| |A4:\*\*1GP3E3| |A5:\*\*1GP3E0| |  |  |  |  |  |  |
|  |  |  |  | Annotation | 1GP3E3 | 1GP3E3 | 1GP3E0 | 1GP3E3 | 1GP3E0 |
|  |  |  |  | Evidence | 4 | 4 | 1 | 4 | 1 |
|  |  |  |  | Focus | 1 | 1 | 1 | 1 | 1 |
|  |  |  |  | Polarity | 7 | 7 | 7 | 7 | 7 |
| 111 | 3358 | 9351973\_90 | This large shift on incubation with AP was inhibited by the presence of NaF (Fig. 1A). |A1:\*\*1SN3E3| |A2:\*\*1SP3E3-| |A3:\*\*1SP3E3-| |A4:\*\*1SN3E3| |A5:\*\*1GP3E3-| |  |  |  |  |  |  |
|  |  |  |  | Annotation | 1SN3E3 | 1SP3E3- | 1SP3E3- | 1SN3E3 | 1GP3E3- |
|  |  |  |  | Evidence | 4 | 4 | 4 | 4 | 4 |
|  |  |  |  | Focus | 4 | 4 | 4 | 4 | 1 |
|  |  |  |  | Polarity | 1 | 7 | 7 | 1 | 7 |
| 112 | 4472 | 12116612\_2 | This internationally best selling book, written for lay readers, described several recorded sessions of alleged time-regression to a prior life nearly two centuries before 1956. |A1:\*\*1GP3E3| |A2:\*\*1GP3E3| |A3:\*\*1GP3E3| |A4:\*\*1GP3E3| |A5:\*\*1GP3E1| |  |  |  |  |  |  |
|  |  |  |  | Annotation | 1GP3E3 | 1GP3E3 | 1GP3E3 | 1GP3E3 | 1GP3E1 |
|  |  |  |  | Evidence | 4 | 4 | 4 | 4 | 2 |
|  |  |  |  | Focus | 1 | 1 | 1 | 1 | 1 |
|  |  |  |  | Polarity | 7 | 7 | 7 | 7 | 7 |
| 113 | 9658 | 12530981\_184 | Precipitates were resolved by SDS-PAGE |A1:\*\*1MP3E3| and proteins were visualized by autoradiography. |A1:\*\*2MP3E3| |A2:\*\*1MP3E3| |A3:\*\*1MP3E3| |A4:\*\*1SP3E3| |A5:\*\*1MP3E3| |  |  |  |  |  |  |
|  |  |  |  | Annotation | 1MP3E3 2MP3E3 | 1MP3E3 1MP3E3 | 1MP3E3 1MP3E3 | 1SP3E3 1SP3E3 | 1MP3E3 1MP3E3 |
|  |  |  |  | Evidence | 4 4 | 4 4 | 4 4 | 4 4 | 4 4 |
|  |  |  |  | Focus | 2 2 | 2 2 | 2 2 | 4 4 | 2 2 |
|  |  |  |  | Polarity | 7 7 | 7 7 | 7 7 | 7 7 | 7 7 |
| 114 | 3563 | 11416148\_141 | To achieve levels of apoptosis which were comparable to the death seen in HOMyc3 cells, |A5:\*\*1SP3E1| we assayed for apoptosis in HO15.19 cells over an extended time course. |A5:\*\*2SP3E3| |A1:\*\*1MSP2E3| |A2:\*\*1MP3E1| |A3:\*\*1MP3E3| |A4:\*\*1MP3E3| |  |  |  |  |  |  |
|  |  |  |  | Annotation | 1MSP2E3 1MSP2E3 | 1MP3E1 1MP3E1 | 1MP3E3 1MP3E3 | 1MP3E3 1MP3E3 | 1SP3E1 2SP3E3 |
|  |  |  |  | Evidence | 4 4 | 2 2 | 4 4 | 4 4 | 2 4 |
|  |  |  |  | Focus | 6 6 | 2 2 | 2 2 | 2 2 | 4 4 |
|  |  |  |  | Polarity | 6 6 | 7 7 | 7 7 | 7 7 | 7 7 |
| 115 | 3499 | 9312148\_21 | The function of this gene, necessary for surfactin production, |A5:\*\*1GP3E1| is still unclear. |A5:\*\*2GP0E0| |A1:\*\*1GP0E0| |A2:\*\*1SN0E0| |A3:\*\*1SP0E0| |A4:\*\*1SP0E0| |  |  |  |  |  |  |
|  |  |  |  | Annotation | 1GP0E0 1GP0E0 | 1SN0E0 1SN0E0 | 1SP0E0 1SP0E0 | 1SP0E0 1SP0E0 | 1GP3E1 2GP0E0 |
|  |  |  |  | Evidence | 1 1 | 1 1 | 1 1 | 1 1 | 2 1 |
|  |  |  |  | Focus | 1 1 | 4 4 | 4 4 | 4 4 | 1 1 |
|  |  |  |  | Polarity | 4 4 | 4 4 | 4 4 | 4 4 | 7 4 |
| 116 | 2688 | 11139192\_96 | Restriction enzyme ( EcoRI) digests of the phage DNA were subjected to gel electrophoresis on a 0.8% agarose gel at 40 V for 3 h. |A1:\*\*1MP3E3| |A2:\*\*1MP3E3| |A3:\*\*1MP3E3| |A4:\*\*1MP3E3| |A5:\*\*1MP3E3| |  |  |  |  |  |  |
|  |  |  |  | Annotation | 1MP3E3 | 1MP3E3 | 1MP3E3 | 1MP3E3 | 1MP3E3 |
|  |  |  |  | Evidence | 4 | 4 | 4 | 4 | 4 |
|  |  |  |  | Focus | 2 | 2 | 2 | 2 | 2 |
|  |  |  |  | Polarity | 7 | 7 | 7 | 7 | 7 |
| 117 | 2796 | 9727068\_235 | p21 mRNA induction occurred earlier and was more sustained in C/ EBPbeta / livers at several time points posthepatectomy (Fig. 7 A). |A1:\*\*1SP3E3| |A2:\*\*1SP3E3| |A3:\*\*1SP3E3| |A4:\*\*1MP3E3| |A5:\*\*1SP3E3+| |  |  |  |  |  |  |
|  |  |  |  | Annotation | 1SP3E3 | 1SP3E3 | 1SP3E3 | 1MP3E3 | 1SP3E3+ |
|  |  |  |  | Evidence | 4 | 4 | 4 | 4 | 4 |
|  |  |  |  | Focus | 4 | 4 | 4 | 2 | 4 |
|  |  |  |  | Polarity | 7 | 7 | 7 | 7 | 7 |
| 118 | 4447 | 12479820\_317 | A tumor fragment inoculum or 5 x 10^6^ of the corresponding cancer cell suspension was pelleted by centrifugation. |A1:\*\*1MP3E3| |A2:\*\*1MP3E3| |A3:\*\*1MP3E3| |A4:\*\*1SP3E3| |A5:\*\*1MP3E3| |  |  |  |  |  |  |
|  |  |  |  | Annotation | 1MP3E3 | 1MP3E3 | 1MP3E3 | 1SP3E3 | 1MP3E3 |
|  |  |  |  | Evidence | 4 | 4 | 4 | 4 | 4 |
|  |  |  |  | Focus | 2 | 2 | 2 | 4 | 2 |
|  |  |  |  | Polarity | 7 | 7 | 7 | 7 | 7 |
| 119 | 2946 | 9463377\_336 | Plasmids pEG1-KK and pEG1-QK, encoding Inv.-Wbp1p fusion proteins with, or without, a functional retrieval motif were used (Gaynor et al., 1994). |A1:\*\*1SP3E23| |A2:\*\*1SP3E23| |A3:\*\*1SP1E2| |A4:\*\*1SP3E2| |A5:\*\*1SP3E2| |  |  |  |  |  |  |
|  |  |  |  | Annotation | 1SP3E23 | 1SP3E23 | 1SP1E2 | 1SP3E2 | 1SP3E2 |
|  |  |  |  | Evidence | 3 | 3 | 3 | 3 | 3 |
|  |  |  |  | Focus | 4 | 4 | 4 | 4 | 4 |
|  |  |  |  | Polarity | 7 | 7 | 5 | 7 | 7 |
| 120 | 5985 | 11207362\_384 | To prepare osteoclasts from progenitor cells, spleen cells from 4-week-old mice were cocultured for 14 days on biotinylated ivory with ST2 stromal cells in the presence of 10 nM vitamin D3 and 100 nM dexamethasone (Udagawa et al., 1989). |A1:\*\*1SP2E23| |A2:\*\*1MP3E23| |A3:\*\*1MSP3E2| |A4:\*\*1SP3E2| |A5:\*\*1MP3E23| |  |  |  |  |  |  |
|  |  |  |  | Annotation | 1SP2E23 | 1MP3E23 | 1MSP3E2 | 1SP3E2 | 1MP3E23 |
|  |  |  |  | Evidence | 3 | 3 | 3 | 3 | 3 |
|  |  |  |  | Focus | 4 | 2 | 6 | 4 | 2 |
|  |  |  |  | Polarity | 6 | 7 | 7 | 7 | 7 |
| 121 | 6838 | 11014836\_6 | Our analysis suggests genetic variance for Drosophila mortality declines with age, |A2:\*\*1SP3E3-| |A5:\*\*1SP1E3-| while genetic variance is constant at all ages for reproductive output. |A2:\*\*1SP3E3| |A1:\*\*1GP3E3| |A3:\*\*1SP3E3| |A4:\*\*1SP3E3| |A5:\*\*2SP2E3| |  |  |  |  |  |  |
|  |  |  |  | Annotation | 1GP3E3 1GP3E3 | 1SP3E3- 1SP3E3 | 1SP3E3 1SP3E3 | 1SP3E3 1SP3E3 | 1SP1E3- 2SP2E3 |
|  |  |  |  | Evidence | 4 4 | 4 4 | 4 4 | 4 4 | 4 4 |
|  |  |  |  | Focus | 1 1 | 4 4 | 4 4 | 4 4 | 4 4 |
|  |  |  |  | Polarity | 7 7 | 7 7 | 7 7 | 7 7 | 5 6 |
| 122 | 4786 | 12575957\_106 | Thus, our results confirm that the currents underlying action potential generation can largely account for the results described above and in Figure 2. |A1:\*\*1GP3E3| |A2:\*\*1GP3E3| |A3:\*\*1SP3E3| |A4:\*\*1SP3E3| |A5:\*\*1SP3E3| |  |  |  |  |  |  |
|  |  |  |  | Annotation | 1GP3E3 | 1GP3E3 | 1SP3E3 | 1SP3E3 | 1SP3E3 |
|  |  |  |  | Evidence | 4 | 4 | 4 | 4 | 4 |
|  |  |  |  | Focus | 1 | 1 | 4 | 4 | 4 |
|  |  |  |  | Polarity | 7 | 7 | 7 | 7 | 7 |
| 123 | 1471 | 11571181\_1 | The diversity of French fungus-ripened cheeses |A5:\*\*1GP3E0| is due partly to the succession of fungi that colonize the cheese during ripening. |A5:\*\*1GP2E0+| |A1:\*\*1GP3E0| |A2:\*\*1GP3E0| |A3:\*\*1SP1E0| |A4:\*\*1GP2E0| |  |  |  |  |  |  |
|  |  |  |  | Annotation | 1GP3E0 1GP3E0 | 1GP3E0 1GP3E0 | 1SP1E0 1SP1E0 | 1GP2E0 1GP2E0 | 1GP3E0 1GP2E0+ |
|  |  |  |  | Evidence | 1 1 | 1 1 | 1 1 | 1 1 | 1 1 |
|  |  |  |  | Focus | 1 1 | 1 1 | 4 4 | 1 1 | 1 1 |
|  |  |  |  | Polarity | 7 7 | 7 7 | 5 5 | 6 6 | 7 6 |
| 124 | 7519 | 10391866\_56 | These were analyzed by approved strategies by EIA and Western blot analysis (Cambridge Biotech Corp., Worcester, Mass.). |A1:\*\*1MP1E3| |A2:\*\*1SP3E3| |A3:\*\*1MP3E3| |A4:\*\*1MP3E3| |A5:\*\*1MP3E1| |  |  |  |  |  |  |
|  |  |  |  | Annotation | 1MP1E3 | 1SP3E3 | 1MP3E3 | 1MP3E3 | 1MP3E1 |
|  |  |  |  | Evidence | 4 | 4 | 4 | 4 | 2 |
|  |  |  |  | Focus | 2 | 4 | 2 | 2 | 2 |
|  |  |  |  | Polarity | 5 | 7 | 7 | 7 | 7 |
| 125 | 8527 | 9788248\_165 | After a 2-h chase at 37 degrees C, the quantity of intracellular PLTP was clearly reduced. |A1:\*\*1SP3E3-| |A2:\*\*1SP3E3-| |A3:\*\*1MP3E3-| |A4:\*\*1SP3E3| |A5:\*\*1MP3E3-| |  |  |  |  |  |  |
|  |  |  |  | Annotation | 1SP3E3- | 1SP3E3- | 1MP3E3- | 1SP3E3 | 1MP3E3- |
|  |  |  |  | Evidence | 4 | 4 | 4 | 4 | 4 |
|  |  |  |  | Focus | 4 | 4 | 2 | 4 | 2 |
|  |  |  |  | Polarity | 7 | 7 | 7 | 7 | 7 |
| 126 | 748 | 11381111\_9 | These data suggest that the COMT Val allele, |A5:\*\*1GP2E1| because it increases prefrontal dopamine catabolism, |A2:\*\*1SP3E3+| |A1:\*\*1SP3E3+| |A3:\*\*1SP3E3+| |A4:\*\*1SP3E3+| |A5:\*\*2SP3E0+| impairs prefrontal cognition and physiology, |A2:\*\*2SP3E3| |A1:\*\*2SN3E3| |A3:\*\*2SP3E0| |A4:\*\*2SP3E0| |A5:\*\*3SP3E0-| and by this mechanism slightly increases risk for schizophrenia. |A2:\*\*3SP3E3+| |A1:\*\*3SP3E3+| |A3:\*\*3SP2E3+| |A4:\*\*3SP2E3+| |A5:\*\*4GP3E0+| |  |  |  |  |  |  |
|  |  |  |  | Annotation | 1SP3E3+ 1SP3E3+ 2SN3E3 3SP3E3+ | 1SP3E3+ 1SP3E3+ 2SP3E3 3SP3E3+ | 1SP3E3+ 1SP3E3+ 2SP3E0 3SP2E3+ | 1SP3E3+ 1SP3E3+ 2SP3E0 3SP2E3+ | 1GP2E1 2SP3E0+ 3SP3E0- 4GP3E0+ |
|  |  |  |  | Evidence | 4 4 4 4 | 4 4 4 4 | 4 4 1 4 | 4 4 1 4 | 2 1 1 1 |
|  |  |  |  | Focus | 4 4 4 4 | 4 4 4 4 | 4 4 4 4 | 4 4 4 4 | 1 4 4 1 |
|  |  |  |  | Polarity | 7 7 1 7 | 7 7 7 7 | 7 7 7 6 | 7 7 7 6 | 6 7 7 7 |
| 127 | 380 | 12408808\_140 | (C) In wild-type muscle-tendon cell junctions, actin filaments terminate at the submembranous density of the muscle hemiadherens junctions (arrowhead), |A1:\*\*1SN3E0| |A2:\*\*1SN3E0| while, in the tendon cells, microtubules (arrow) run from tonofibrils embedded in the cuticle (c) to the basal hemiadherens junctions. |A1:\*\*2SP3E0| |A2:\*\*2SP3E0| |A3:\*\*1SP3E0| |A4:\*\*1SP3E0| |A5:\*\*1SP3E3| |  |  |  |  |  |  |
|  |  |  |  | Annotation | 1SN3E0 2SP3E0 | 1SN3E0 2SP3E0 | 1SP3E0 1SP3E0 | 1SP3E0 1SP3E0 | 1SP3E3 1SP3E3 |
|  |  |  |  | Evidence | 1 1 | 1 1 | 1 1 | 1 1 | 4 4 |
|  |  |  |  | Focus | 4 4 | 4 4 | 4 4 | 4 4 | 4 4 |
|  |  |  |  | Polarity | 1 7 | 1 7 | 7 7 | 7 7 | 7 7 |
| 128 | 2940 | 9971740\_43 | For experiments on axons in the presence and absence of metabolic substrates, the above medium was rinsed and replaced with Dulbecco's PBS ( ) supplemented with 0.5% MethocelTM and 50 ng/ml 2.5 nerve growth factor, with or without 0.6% glucose ( ) and 0.055% sodium pyruvate ( ). |A1:\*\*1SP3E23| |A2:\*\*1SP3E23| |A3:\*\*1MP3E3| |A4:\*\*1MP3E3| |A5:\*\*1MP3E3| |  |  |  |  |  |  |
|  |  |  |  | Annotation | 1SP3E23 | 1SP3E23 | 1MP3E3 | 1MP3E3 | 1MP3E3 |
|  |  |  |  | Evidence | 3 | 3 | 4 | 4 | 4 |
|  |  |  |  | Focus | 4 | 4 | 2 | 2 | 2 |
|  |  |  |  | Polarity | 7 | 7 | 7 | 7 | 7 |
| 129 | 315 | 11333250\_583 | TRAUT, T. W., 1994 The functions and consensus motifs of nine types of peptide segments that form different types of nucleotide-binding sites. |A1:\*\*1SP2E3| |A2:\*\*1SP2E2| |A3:\*\*1SP3E2| |A4:\*\*1SP3E2| |A5:\*\*1SP3E2| |  |  |  |  |  |  |
|  |  |  |  | Annotation | 1SP2E3 | 1SP2E2 | 1SP3E2 | 1SP3E2 | 1SP3E2 |
|  |  |  |  | Evidence | 4 | 3 | 3 | 3 | 3 |
|  |  |  |  | Focus | 4 | 4 | 4 | 4 | 4 |
|  |  |  |  | Polarity | 6 | 6 | 7 | 7 | 7 |
| 130 | 3116 | 10862619\_43 | In S. pombe, deletion of rqh1 only partially suppressed the defect observed in top3 cells ( ). |A1:\*\*1SN3E2-| |A2:\*\*1SN3E2-| |A3:\*\*1SN1E0| |A4:\*\*1SN2E0| |A5:\*\*1SP3E2-| |  |  |  |  |  |  |
|  |  |  |  | Annotation | 1SN3E2- | 1SN3E2- | 1SN1E0 | 1SN2E0 | 1SP3E2- |
|  |  |  |  | Evidence | 3 | 3 | 1 | 1 | 3 |
|  |  |  |  | Focus | 4 | 4 | 4 | 4 | 4 |
|  |  |  |  | Polarity | 1 | 1 | 3 | 2 | 7 |
| 131 | 5920 | 10531359\_36 | The results are expressed as the mean plus-or-minus S.E. of three independent experiments performed in triplicate. |A1:\*\*1GP3E3| |A2:\*\*1GP3E3| |A3:\*\*1SP3E3| |A4:\*\*1GP3E3| |A5:\*\*1GP3E3| |  |  |  |  |  |  |
|  |  |  |  | Annotation | 1GP3E3 | 1GP3E3 | 1SP3E3 | 1GP3E3 | 1GP3E3 |
|  |  |  |  | Evidence | 4 | 4 | 4 | 4 | 4 |
|  |  |  |  | Focus | 1 | 1 | 4 | 1 | 1 |
|  |  |  |  | Polarity | 7 | 7 | 7 | 7 | 7 |
| 132 | 7338 | 9367983\_275 | The amplified product was inserted into plasmid II digested with SacI and HindIII. |A1:\*\*1SP3E3| |A2:\*\*1MP3E3| |A3:\*\*1MP3E3| |A4:\*\*1MP3E0| |A5:\*\*1GP3E3| |  |  |  |  |  |  |
|  |  |  |  | Annotation | 1SP3E3 | 1MP3E3 | 1MP3E3 | 1MP3E0 | 1GP3E3 |
|  |  |  |  | Evidence | 4 | 4 | 4 | 1 | 4 |
|  |  |  |  | Focus | 4 | 2 | 2 | 2 | 1 |
|  |  |  |  | Polarity | 7 | 7 | 7 | 7 | 7 |
| 133 | 3254 | 12111470\_7 | In Experiment 2, one of the de-novo patients was normosmic, 3 hyposmic, and 1 anosmic. |A1:\*\*1SP3E3| |A2:\*\*1SP3E3| |A3:\*\*1SP3E3| |A4:\*\*1MP3E3| |A5:\*\*1GP3E3| |  |  |  |  |  |  |
|  |  |  |  | Annotation | 1SP3E3 | 1SP3E3 | 1SP3E3 | 1MP3E3 | 1GP3E3 |
|  |  |  |  | Evidence | 4 | 4 | 4 | 4 | 4 |
|  |  |  |  | Focus | 4 | 4 | 4 | 2 | 1 |
|  |  |  |  | Polarity | 7 | 7 | 7 | 7 | 7 |
| 134 | 963 | 11292744\_99 | They were submitted from different areas of the United States. |A1:\*\*1GP3E3| |A2:\*\*1GP3E3| |A3:\*\*1GP3E3| |A4:\*\*1GP3E0| |A5:\*\*1GP3E1| |  |  |  |  |  |  |
|  |  |  |  | Annotation | 1GP3E3 | 1GP3E3 | 1GP3E3 | 1GP3E0 | 1GP3E1 |
|  |  |  |  | Evidence | 4 | 4 | 4 | 1 | 2 |
|  |  |  |  | Focus | 1 | 1 | 1 | 1 | 1 |
|  |  |  |  | Polarity | 7 | 7 | 7 | 7 | 7 |
| 135 | 4424 | 10496918\_128 | Indeed, exogenous CSA almost totally inhibited this binding, |A1:\*\*1SN3E3| |A2:\*\*1SP3E3-| |A3:\*\*1SP2E0| |A4:\*\*1SN3E0-| |A5:\*\*1SP3E3-| while anti-ICAM-1 monoclonal antibody did not affect cytoadherence. |A1:\*\*2SN3E3| |A2:\*\*2SN3E3| |A3:\*\*2SN3E0| |A4:\*\*2SN3E0| |A5:\*\*2SN3E1| |  |  |  |  |  |  |
|  |  |  |  | Annotation | 1SN3E3 2SN3E3 | 1SP3E3- 2SN3E3 | 1SP2E0 2SN3E0 | 1SN3E0- 2SN3E0 | 1SP3E3- 2SN3E1 |
|  |  |  |  | Evidence | 4 4 | 4 4 | 1 1 | 1 1 | 4 2 |
|  |  |  |  | Focus | 4 4 | 4 4 | 4 4 | 4 4 | 4 4 |
|  |  |  |  | Polarity | 1 1 | 7 1 | 6 1 | 1 1 | 7 1 |
| 136 | 7403 | 11514458\_115 | Effect of mating system on polymorphism: |A3:\*\*1SP3E0| |A5:\*\*1GP3E0| The effect of mating system and species on silent polymorphism is highly significant (Mann-Whitney U test, P < 0.001). |A3:\*\*1SP3E2| |A1:\*\*1GP3E3| |A2:\*\*1SP3E3| |A4:\*\*1SP3E0| |A5:\*\*2GP3E3| |  |  |  |  |  |  |
|  |  |  |  | Annotation | 1GP3E3 1GP3E3 | 1SP3E3 1SP3E3 | 1SP3E0 1SP3E2 | 1SP3E0 1SP3E0 | 1GP3E0 2GP3E3 |
|  |  |  |  | Evidence | 4 4 | 4 4 | 1 3 | 1 1 | 1 4 |
|  |  |  |  | Focus | 1 1 | 4 4 | 4 4 | 4 4 | 1 1 |
|  |  |  |  | Polarity | 7 7 | 7 7 | 7 7 | 7 7 | 7 7 |
| 137 | 5765 | 8702751\_29 | Fig. 3. Fig.3. BoNT E preferentially destabilizes docking protein complexes following ATP-dependent priming. |A1:\*\*1SP3E3| |A2:\*\*1SP3E3-| |A3:\*\*1SP2E3| |A4:\*\*1SP3E3| |A5:\*\*1SP3E3-| |  |  |  |  |  |  |
|  |  |  |  | Annotation | 1SP3E3 | 1SP3E3- | 1SP2E3 | 1SP3E3 | 1SP3E3- |
|  |  |  |  | Evidence | 4 | 4 | 4 | 4 | 4 |
|  |  |  |  | Focus | 4 | 4 | 4 | 4 | 4 |
|  |  |  |  | Polarity | 7 | 7 | 6 | 7 | 7 |
| 138 | 2216 | 10455125\_26 | In separate experiments, it was however noted that in the presence of Mn2+-activated integrins the contribution of L1-L1 binding of B16F10 cells was marginal (data not shown; also see Fig. |A1:\*\*1SP3E1| |A2:\*\*1SP3E1| |A3:\*\*1SP3E3| |A4:\*\*1MP3E3+| |A5:\*\*1SP3E3| |  |  |  |  |  |  |
|  |  |  |  | Annotation | 1SP3E1 | 1SP3E1 | 1SP3E3 | 1MP3E3+ | 1SP3E3 |
|  |  |  |  | Evidence | 2 | 2 | 4 | 4 | 4 |
|  |  |  |  | Focus | 4 | 4 | 4 | 2 | 4 |
|  |  |  |  | Polarity | 7 | 7 | 7 | 7 | 7 |
| 139 | 3944 | 9658196\_62 | The H1 receptor antagonists were dissolved in dimethylsulfoxide at concentrations between 5 and 50 mM, and stock solutions were kept at 20 degrees. |A1:\*\*1SP3E3| |A2:\*\*1SP3E3| |A3:\*\*1MP3E3| |A4:\*\*1MP3E3| |A5:\*\*1MP3E3| |  |  |  |  |  |  |
|  |  |  |  | Annotation | 1SP3E3 | 1SP3E3 | 1MP3E3 | 1MP3E3 | 1MP3E3 |
|  |  |  |  | Evidence | 4 | 4 | 4 | 4 | 4 |
|  |  |  |  | Focus | 4 | 4 | 2 | 2 | 2 |
|  |  |  |  | Polarity | 7 | 7 | 7 | 7 | 7 |
| 140 | 8442 | 12117676\_11 | Moreover, a synergistic induction of these MMPs was seen when IL17 was combined with other proinflammatory cytokines. |A1:\*\*1SP3E3| |A2:\*\*1SP3E3| |A3:\*\*1SP3E3| |A4:\*\*1SP3E0| |A5:\*\*1SP2E1+| |  |  |  |  |  |  |
|  |  |  |  | Annotation | 1SP3E3 | 1SP3E3 | 1SP3E3 | 1SP3E0 | 1SP2E1+ |
|  |  |  |  | Evidence | 4 | 4 | 4 | 1 | 2 |
|  |  |  |  | Focus | 4 | 4 | 4 | 4 | 4 |
|  |  |  |  | Polarity | 7 | 7 | 7 | 7 | 6 |
| 141 | 4305 | 10318810\_2 | Inhibitors of the peptidyltransferase reaction ( e.g. anisomycin) can trigger a ribotoxic stress response |A3:\*\*1SP2E0| |A4:\*\*1SP2E0| that activates c-Jun N-terminal kinase (JNK)/p38 mitogen-activated protein kinases, |A1:\*\*1SP2E0| |A2:\*\*1SP2E0+| |A4:\*\*2SP3E0+| |A5:\*\*1SP3E0+| components of a signaling cascade that regulates cell survival in response to stress. |A1:\*\*2SP3E0| |A2:\*\*2SP3E0| |A3:\*\*2SP3E0| |A4:\*\*3SP3E0| |A5:\*\*2SP3E0| |  |  |  |  |  |  |
|  |  |  |  | Annotation | 1SP2E0 1SP2E0 2SP3E0 | 1SP2E0+ 1SP2E0+ 2SP3E0 | 1SP2E0 2SP3E0 2SP3E0 | 1SP2E0 2SP3E0+ 3SP3E0 | 1SP3E0+ 1SP3E0+ 2SP3E0 |
|  |  |  |  | Evidence | 1 1 1 | 1 1 1 | 1 1 1 | 1 1 1 | 1 1 1 |
|  |  |  |  | Focus | 4 4 4 | 4 4 4 | 4 4 4 | 4 4 4 | 4 4 4 |
|  |  |  |  | Polarity | 6 6 7 | 6 6 7 | 6 7 7 | 6 7 7 | 7 7 7 |
| 142 | 8373 | 12399371\_227 | The substantial (11%) and significant gain in the average fitness of the mutator subpopulation in population Ara+3 between generations 3000 and 3500 was consistent with hitchhiking. |A1:\*\*1GP3E3| |A2:\*\*1GP3E3| |A3:\*\*1SP3E3+| |A4:\*\*1SP3E3+| |A5:\*\*1GP3E0| |  |  |  |  |  |  |
|  |  |  |  | Annotation | 1GP3E3 | 1GP3E3 | 1SP3E3+ | 1SP3E3+ | 1GP3E0 |
|  |  |  |  | Evidence | 4 | 4 | 4 | 4 | 1 |
|  |  |  |  | Focus | 1 | 1 | 4 | 4 | 1 |
|  |  |  |  | Polarity | 7 | 7 | 7 | 7 | 7 |
| 143 | 7331 | 11430806\_151 | This increase was evident both in cell body number and the number of terminals in the IPL (Figure 5B). |A1:\*\*1GP3E3+| |A2:\*\*1SP3E3+| |A3:\*\*1SP3E3| |A4:\*\*1SP3E3+| |A5:\*\*1GP3E3| |  |  |  |  |  |  |
|  |  |  |  | Annotation | 1GP3E3+ | 1SP3E3+ | 1SP3E3 | 1SP3E3+ | 1GP3E3 |
|  |  |  |  | Evidence | 4 | 4 | 4 | 4 | 4 |
|  |  |  |  | Focus | 1 | 4 | 4 | 4 | 1 |
|  |  |  |  | Polarity | 7 | 7 | 7 | 7 | 7 |
| 144 | 8498 | 12189148\_21 | D, recombinant PGAM-B protein was affinity-purified and subjected to in vitro kinase reaction with purified, constitutively active Pak1. |A1:\*\*1SP3E3| |A2:\*\*1SP3E3| |A3:\*\*1SP3E3| |A4:\*\*1MP3E3| |A5:\*\*1MP3E3| |  |  |  |  |  |  |
|  |  |  |  | Annotation | 1SP3E3 | 1SP3E3 | 1SP3E3 | 1MP3E3 | 1MP3E3 |
|  |  |  |  | Evidence | 4 | 4 | 4 | 4 | 4 |
|  |  |  |  | Focus | 4 | 4 | 4 | 2 | 2 |
|  |  |  |  | Polarity | 7 | 7 | 7 | 7 | 7 |
| 145 | 3901 | 9390512\_272 | Both linkage and association methods have been developed to map QTLs (Risch and Zhang 1995 ), |A1:\*\*1MP3E2| |A2:\*\*1MP3E2| and successes have been reported for reading disability ( Cardon et al. 1994 ). |A3:\*\*1SP3E2| |A1:\*\*2GP3E2| |A2:\*\*2GP3E2| |A4:\*\*1SP3E2| |A5:\*\*1GP3E2| |  |  |  |  |  |  |
|  |  |  |  | Annotation | 1MP3E2 2GP3E2 | 1MP3E2 2GP3E2 | 1SP3E2 1SP3E2 | 1SP3E2 1SP3E2 | 1GP3E2 1GP3E2 |
|  |  |  |  | Evidence | 3 3 | 3 3 | 3 3 | 3 3 | 3 3 |
|  |  |  |  | Focus | 2 1 | 2 1 | 4 4 | 4 4 | 1 1 |
|  |  |  |  | Polarity | 7 7 | 7 7 | 7 7 | 7 7 | 7 7 |
| 146 | 2727 | 11583618\_98 | Furthermore, coexpression of a dominant-negative HPC2 (described below) reversed this Rb- and HPC2-dependent repression of the SV40 promoter/enhancer (Figure 1A). |A1:\*\*1SP3E3| |A2:\*\*1SP3E3| |A3:\*\*1SP3E3| |A4:\*\*1SP3E3-| |A5:\*\*1SP3E3| |  |  |  |  |  |  |
|  |  |  |  | Annotation | 1SP3E3 | 1SP3E3 | 1SP3E3 | 1SP3E3- | 1SP3E3 |
|  |  |  |  | Evidence | 4 | 4 | 4 | 4 | 4 |
|  |  |  |  | Focus | 4 | 4 | 4 | 4 | 4 |
|  |  |  |  | Polarity | 7 | 7 | 7 | 7 | 7 |
| 147 | 3990 | 12108620\_6 | Results suggest that individuals with AS/HFA have difficulty extracting mental state information from vocalizations. |A1:\*\*1GP3E3| |A2:\*\*1GP3E3| |A3:\*\*1SP2E3| |A4:\*\*1SP3E1| |A5:\*\*1SP2E1| |  |  |  |  |  |  |
|  |  |  |  | Annotation | 1GP3E3 | 1GP3E3 | 1SP2E3 | 1SP3E1 | 1SP2E1 |
|  |  |  |  | Evidence | 4 | 4 | 4 | 2 | 2 |
|  |  |  |  | Focus | 1 | 1 | 4 | 4 | 4 |
|  |  |  |  | Polarity | 7 | 7 | 6 | 7 | 6 |
| 148 | 6965 | 12686140\_55 | Moreover, it is known that drosophila dishevelled, regulator of Wnt signaling, |A5:\*\*1GP3E1| is able to up-regulate -catenin levels and activate c-jun through the JNKs. [11 and 13]. |A1:\*\*1SP3E2+| |A2:\*\*1SP3E2+| |A3:\*\*1SP2E2| |A4:\*\*1SP3E2+| |A5:\*\*2SP3E2+| |  |  |  |  |  |  |
|  |  |  |  | Annotation | 1SP3E2+ 1SP3E2+ | 1SP3E2+ 1SP3E2+ | 1SP2E2 1SP2E2 | 1SP3E2+ 1SP3E2+ | 1GP3E1 2SP3E2+ |
|  |  |  |  | Evidence | 3 3 | 3 3 | 3 3 | 3 3 | 2 3 |
|  |  |  |  | Focus | 4 4 | 4 4 | 4 4 | 4 4 | 1 4 |
|  |  |  |  | Polarity | 7 7 | 7 7 | 6 6 | 7 7 | 7 7 |
| 149 | 1774 | 12967567\_9 | In metazoan cells, the nuclear envelope becomes reorganized at mitosis, |A1:\*\*1SP3E0| and ultimately its disassembly is key to accurate inheritance of both genomic DNA and nuclear envelope components. |A1:\*\*2GP3E0| |A2:\*\*1SP3E0| |A3:\*\*1SP3E0| |A4:\*\*1SP3E0| |A5:\*\*1SP3E0| |  |  |  |  |  |  |
|  |  |  |  | Annotation | 1SP3E0 2GP3E0 | 1SP3E0 1SP3E0 | 1SP3E0 1SP3E0 | 1SP3E0 1SP3E0 | 1SP3E0 1SP3E0 |
|  |  |  |  | Evidence | 1 1 | 1 1 | 1 1 | 1 1 | 1 1 |
|  |  |  |  | Focus | 4 1 | 4 4 | 4 4 | 4 4 | 4 4 |
|  |  |  |  | Polarity | 7 7 | 7 7 | 7 7 | 7 7 | 7 7 |
| 150 | 2628 | 10383421\_17 | It is particularly interesting that the domains labeled with the Bpa22- and Bpa6-containing secretin analogues were both near the end of the amino terminus of the secretin receptor. |A1:\*\*1SP3E3| |A2:\*\*1SP3E1| |A3:\*\*1SP3E0| |A4:\*\*1SP3E0| |A5:\*\*1SP3E1| |  |  |  |  |  |  |
|  |  |  |  | Annotation | 1SP3E3 | 1SP3E1 | 1SP3E0 | 1SP3E0 | 1SP3E1 |
|  |  |  |  | Evidence | 4 | 2 | 1 | 1 | 2 |
|  |  |  |  | Focus | 4 | 4 | 4 | 4 | 4 |
|  |  |  |  | Polarity | 7 | 7 | 7 | 7 | 7 |
| 151 | 519 | 12033758\_2 | It is seen in patients with healthy joints as well as those with somatic dysfunction. |A1:\*\*1GP3E0| |A2:\*\*1SP3E0| |A3:\*\*1SP3E0| |A4:\*\*1SP3E0| |A5:\*\*1SP3E1| |  |  |  |  |  |  |
|  |  |  |  | Annotation | 1GP3E0 | 1SP3E0 | 1SP3E0 | 1SP3E0 | 1SP3E1 |
|  |  |  |  | Evidence | 1 | 1 | 1 | 1 | 2 |
|  |  |  |  | Focus | 1 | 4 | 4 | 4 | 4 |
|  |  |  |  | Polarity | 7 | 7 | 7 | 7 | 7 |
| 152 | 7991 | 10733592\_145 | To identify cis-acting elements involved in hormone control of bcl-2 expression, we have made a reporter construct in which the bcl-2 P1 promoter, from 1,623 to 1,390 bp upstream of the translation start site, was cloned 5' to the CAT gene (P1 construct). |A1:\*\*1SP3E3| |A2:\*\*1SP3E3| |A3:\*\*1SP3E0| |A4:\*\*1SP3E0| |A5:\*\*1SMP3E3| |  |  |  |  |  |  |
|  |  |  |  | Annotation | 1SP3E3 | 1SP3E3 | 1SP3E0 | 1SP3E0 | 1SMP3E3 |
|  |  |  |  | Evidence | 4 | 4 | 1 | 1 | 4 |
|  |  |  |  | Focus | 4 | 4 | 4 | 4 | 6 |
|  |  |  |  | Polarity | 7 | 7 | 7 | 7 | 7 |
| 153 | 6762 | 9262333\_183 | More recently, it has been demonstrated that the suppressant effects of 8-OHDPAT on the basal firing rate of spontaneously active cells in the mPFCx is potentiated by concurrent iontophoretic administration of either of the 5-HT2A antagonists ritanserin and MDL 100,907 (Ashby et al., 1994). |A1:\*\*1SP3E2| |A2:\*\*1SP3E2| |A3:\*\*1SP3E2| |A4:\*\*1SP3E2| |A5:\*\*1MP3E12| |  |  |  |  |  |  |
|  |  |  |  | Annotation | 1SP3E2 | 1SP3E2 | 1SP3E2 | 1SP3E2 | 1MP3E12 |
|  |  |  |  | Evidence | 3 | 3 | 3 | 3 | 2 |
|  |  |  |  | Focus | 4 | 4 | 4 | 4 | 2 |
|  |  |  |  | Polarity | 7 | 7 | 7 | 7 | 7 |
| 154 | 7018 | 12871700\_429 | S. Ikeda, S. Kishida, H. Yamamoto, H. Murai, S. Koyama and A. Kikuchi, |A3:\*\*1GP3E0| Axin, a negative regulator of the Wnt signaling pathway, forms a complex with GSK-beta and beta-catenin |A1:\*\*1SP3E3| |A2:\*\*1SP3E3| |A5:\*\*1SP3E2| and |A4:\*\*1SP3E0| promotes GSK-3beta-dependent phosphorylation and beta-catenin. |A1:\*\*2SP3E3+| |A2:\*\*2SP3E3+| |A3:\*\*1SP3E0| |A4:\*\*2SP3E0+| |A5:\*\*2SP3E2+| |  |  |  |  |  |  |
|  |  |  |  | Annotation | 1SP3E3 1SP3E3 2SP3E3+ 2SP3E3+ | 1SP3E3 1SP3E3 2SP3E3+ 2SP3E3+ | 1GP3E0 1SP3E0 1SP3E0 1SP3E0 | 1SP3E0 1SP3E0 1SP3E0 2SP3E0+ | 1SP3E2 1SP3E2 2SP3E2+ 2SP3E2+ |
|  |  |  |  | Evidence | 4 4 4 4 | 4 4 4 4 | 1 1 1 1 | 1 1 1 1 | 3 3 3 3 |
|  |  |  |  | Focus | 4 4 4 4 | 4 4 4 4 | 1 4 4 4 | 4 4 4 4 | 4 4 4 4 |
|  |  |  |  | Polarity | 7 7 7 7 | 7 7 7 7 | 7 7 7 7 | 7 7 7 7 | 7 7 7 7 |
| 155 | 2723 | 12359842\_24 | During the whole experiment, the position of the fly in the chamber is monitored, and the fraction of time the flies spent on the "`unpunished"` side is calculated. |A1:\*\*1MP3E3| |A2:\*\*1MP3E3| |A3:\*\*1SP3E0| |A4:\*\*1SP3E3| |A5:\*\*1MP2E3| |  |  |  |  |  |  |
|  |  |  |  | Annotation | 1MP3E3 | 1MP3E3 | 1SP3E0 | 1SP3E3 | 1MP2E3 |
|  |  |  |  | Evidence | 4 | 4 | 1 | 4 | 4 |
|  |  |  |  | Focus | 2 | 2 | 4 | 4 | 2 |
|  |  |  |  | Polarity | 7 | 7 | 7 | 7 | 6 |
| 156 | 2861 | 9512517\_281 | By analogy to the role that vertebrate myb genes are thought to play in cell cycle regulation, |A1:\*\*1SP1E0| |A3:\*\*1SP1E1| |A5:\*\*1GP3E0| Dm myb may also participate in regulation of the G1/S transition, |A1:\*\*2SP1E0| |A2:\*\*1SP1E0| |A3:\*\*2SP2E0| |A5:\*\*2GP1E0| but our studies indicate that it is not required for DNA synthesis in endocycling cells. |A1:\*\*3SN3E3| |A2:\*\*2SN3E3| |A3:\*\*3SN3E3| |A4:\*\*1SP3E3| |A5:\*\*3GN3E3| |  |  |  |  |  |  |
|  |  |  |  | Annotation | 1SP1E0 2SP1E0 3SN3E3 | 1SP1E0 1SP1E0 2SN3E3 | 1SP1E1 2SP2E0 3SN3E3 | 1SP3E3 1SP3E3 1SP3E3 | 1GP3E0 2GP1E0 3GN3E3 |
|  |  |  |  | Evidence | 1 1 4 | 1 1 4 | 2 1 4 | 4 4 4 | 1 1 4 |
|  |  |  |  | Focus | 4 4 4 | 4 4 4 | 4 4 4 | 4 4 4 | 1 1 1 |
|  |  |  |  | Polarity | 5 5 1 | 5 5 1 | 5 6 1 | 7 7 7 | 7 5 1 |
| 157 | 8510 | 11980919\_239 | When overexpressed, many of these "`BH3-only"` proteins (e.g., BIM and HRK), like their multidomain cousins, localize to mitochondria, |A4:\*\*1SP3E3+| cause cyt c release, |A1:\*\*1SP3E2| |A3:\*\*1SP3E3+| |A4:\*\*2SP3E0| and induce cell death |A1:\*\*2SP3E2| |A2:\*\*1SP3E2| |A5:\*\*1SP3E2+| that can be attenuated by caspase inhibition |A5:\*\*2SP3E2-| or cooverexpression of antiapoptotic BCL-2 proteins ( Imaizumi et al., 1997; Inohara et al., 1997; O'Connor et al., 1998). |A1:\*\*3SN1E2| |A2:\*\*2SP1E2| |A3:\*\*2SP3E2+| |A4:\*\*3SP3E2+| |A5:\*\*3SP3E2+| |  |  |  |  |  |  |
|  |  |  |  | Annotation | 1SP3E2 1SP3E2 2SP3E2 3SN1E2 3SN1E2 | 1SP3E2 1SP3E2 1SP3E2 2SP1E2 2SP1E2 | 1SP3E3+ 1SP3E3+ 2SP3E2+ 2SP3E2+ 2SP3E2+ | 1SP3E3+ 2SP3E0 3SP3E2+ 3SP3E2+ 3SP3E2+ | 1SP3E2+ 1SP3E2+ 1SP3E2+ 2SP3E2- 3SP3E2+ |
|  |  |  |  | Evidence | 3 3 3 3 3 | 3 3 3 3 3 | 4 4 3 3 3 | 4 1 3 3 3 | 3 3 3 3 3 |
|  |  |  |  | Focus | 4 4 4 4 4 | 4 4 4 4 4 | 4 4 4 4 4 | 4 4 4 4 4 | 4 4 4 4 4 |
|  |  |  |  | Polarity | 7 7 7 3 3 | 7 7 7 5 5 | 7 7 7 7 7 | 7 7 7 7 7 | 7 7 7 7 7 |
| 158 | 896 | 12726860\_121 | To more directly assess whether loss of E2F3 can lead to aneuploidy, |A5:\*\*1SP1E0| chromosome numbers were counted in cells from early and late passage populations. |A5:\*\*2SP3E3| |A1:\*\*1SP1E3| |A2:\*\*1SP1E3| |A3:\*\*1SP3E3| |A4:\*\*1MP3E3| |  |  |  |  |  |  |
|  |  |  |  | Annotation | 1SP1E3 1SP1E3 | 1SP1E3 1SP1E3 | 1SP3E3 1SP3E3 | 1MP3E3 1MP3E3 | 1SP1E0 2SP3E3 |
|  |  |  |  | Evidence | 4 4 | 4 4 | 4 4 | 4 4 | 1 4 |
|  |  |  |  | Focus | 4 4 | 4 4 | 4 4 | 2 2 | 4 4 |
|  |  |  |  | Polarity | 5 5 | 5 5 | 7 7 | 7 7 | 5 7 |
| 159 | 7256 | 9736749\_103 | These deformations, shown as either simple surface displacements or z-scores, were similar on each side and involved specific subregions of the head and body of the hippocampus. |A1:\*\*1GP3E3| |A2:\*\*1GP3E3| |A3:\*\*1SP3E3| |A4:\*\*1SP3E0| |A5:\*\*1SP3E1| |  |  |  |  |  |  |
|  |  |  |  | Annotation | 1GP3E3 | 1GP3E3 | 1SP3E3 | 1SP3E0 | 1SP3E1 |
|  |  |  |  | Evidence | 4 | 4 | 4 | 1 | 2 |
|  |  |  |  | Focus | 1 | 1 | 4 | 4 | 4 |
|  |  |  |  | Polarity | 7 | 7 | 7 | 7 | 7 |
| 160 | 237 | 12810961\_7 | Cells expressing TGase2 antisense RNA are less sensitive to apoptosis induced by various agents, |A1:\*\*1SP3E0-| |A2:\*\*1SP3E0-| |A5:\*\*1SN3E1| whereas cells overexpressing TGase2 have increased sensitivity ( 13, 14). |A1:\*\*2SP3E2+| |A2:\*\*2SP3E2+| |A3:\*\*1SP3E2| |A4:\*\*1SP3E3+| |A5:\*\*2SP3E2+| |  |  |  |  |  |  |
|  |  |  |  | Annotation | 1SP3E0- 2SP3E2+ | 1SP3E0- 2SP3E2+ | 1SP3E2 1SP3E2 | 1SP3E3+ 1SP3E3+ | 1SN3E1 2SP3E2+ |
|  |  |  |  | Evidence | 1 3 | 1 3 | 3 3 | 4 4 | 2 3 |
|  |  |  |  | Focus | 4 4 | 4 4 | 4 4 | 4 4 | 4 4 |
|  |  |  |  | Polarity | 7 7 | 7 7 | 7 7 | 7 7 | 1 7 |
| 161 | 9478 | 10790415\_521 | In using models M9 - M11 as well as other continuous distribution models not based on the beta distribution (M5, M6, M12, and M13), |A1:\*\*1GN0E0| |A3:\*\*1SN3E0| |A4:\*\*1SN3E0| |A5:\*\*1GN3E0| it is important to examine the discrete distributions to see whether there is any category with omega > 1. |A1:\*\*2GP0E0| |A2:\*\*1SN3E0| |A3:\*\*2SP3E0| |A4:\*\*2SP3E0| |A5:\*\*2GP3E0| |  |  |  |  |  |  |
|  |  |  |  | Annotation | 1GN0E0 2GP0E0 | 1SN3E0 1SN3E0 | 1SN3E0 2SP3E0 | 1SN3E0 2SP3E0 | 1GN3E0 2GP3E0 |
|  |  |  |  | Evidence | 1 1 | 1 1 | 1 1 | 1 1 | 1 1 |
|  |  |  |  | Focus | 1 1 | 4 4 | 4 4 | 4 4 | 1 1 |
|  |  |  |  | Polarity | 4 4 | 1 1 | 1 7 | 1 7 | 1 7 |
| 162 | 5589 | 9560377\_181 | One important qualification to our conclusions relates to the possibility that |A5:\*\*1SP2E3| the dnaE antimutator alleles not only reduce normal DNA replication errors |A3:\*\*1SP1E3| but also reduce mutations at DNA lesions. |A3:\*\*2SN3E0| |A1:\*\*1GP3E1-| |A2:\*\*1SP3E1-| |A4:\*\*1SP2E3-| |A5:\*\*2SP3E0-| |  |  |  |  |  |  |
|  |  |  |  | Annotation | 1GP3E1- 1GP3E1- 1GP3E1- | 1SP3E1- 1SP3E1- 1SP3E1- | 1SP1E3 1SP1E3 2SN3E0 | 1SP2E3- 1SP2E3- 1SP2E3- | 1SP2E3 2SP3E0- 2SP3E0- |
|  |  |  |  | Evidence | 2 2 2 | 2 2 2 | 4 4 1 | 4 4 4 | 4 1 1 |
|  |  |  |  | Focus | 1 1 1 | 4 4 4 | 4 4 4 | 4 4 4 | 4 4 4 |
|  |  |  |  | Polarity | 7 7 7 | 7 7 7 | 5 5 1 | 6 6 6 | 6 7 7 |
| 163 | 3519 | 9039265\_27 | Affected mice that survive past two months of age recover much of their immune function and body weight and live normal lifespans, |A1:\*\*1GP3E2| |A2:\*\*1SP3E0| |A4:\*\*1SP3E0| |A5:\*\*1SP3E2+| but exhibit reduced fertility (Dung and Swigart, 1971 Dung and Swigart, 1972 ; Dung, 1977). |A1:\*\*2GP3E2-| |A2:\*\*1SP3E2-| |A3:\*\*1SP3E2| |A4:\*\*2SP3E2-| |A5:\*\*2SP3E2-| |  |  |  |  |  |  |
|  |  |  |  | Annotation | 1GP3E2 2GP3E2- | 1SP3E0 1SP3E2- | 1SP3E2 1SP3E2 | 1SP3E0 2SP3E2- | 1SP3E2+ 2SP3E2- |
|  |  |  |  | Evidence | 3 3 | 1 3 | 3 3 | 1 3 | 3 3 |
|  |  |  |  | Focus | 1 1 | 4 4 | 4 4 | 4 4 | 4 4 |
|  |  |  |  | Polarity | 7 7 | 7 7 | 7 7 | 7 7 | 7 7 |
| 164 | 4092 | 9430651\_126 | Introduction of a DSB would result in recruitment of a Ku heterodimer to each end. |A1:\*\*1GP2E0| |A2:\*\*1SP2E0| |A3:\*\*1SP3E0| |A4:\*\*1SP3E0| |A5:\*\*1GP3E1| |  |  |  |  |  |  |
|  |  |  |  | Annotation | 1GP2E0 | 1SP2E0 | 1SP3E0 | 1SP3E0 | 1GP3E1 |
|  |  |  |  | Evidence | 1 | 1 | 1 | 1 | 2 |
|  |  |  |  | Focus | 1 | 4 | 4 | 4 | 1 |
|  |  |  |  | Polarity | 6 | 6 | 7 | 7 | 7 |
| 165 | 9798 | 9110171\_253 | Therefore, we hypothesize that all of the conserved noncoding segments are (or were) required functionally for the proper expression of the btk locus. |A1:\*\*1GP2E3| |A2:\*\*1SP2E3| |A3:\*\*1SP2E0| |A4:\*\*1SP3E1| |A5:\*\*1GP1E3| |  |  |  |  |  |  |
|  |  |  |  | Annotation | 1GP2E3 | 1SP2E3 | 1SP2E0 | 1SP3E1 | 1GP1E3 |
|  |  |  |  | Evidence | 4 | 4 | 1 | 2 | 4 |
|  |  |  |  | Focus | 1 | 4 | 4 | 4 | 1 |
|  |  |  |  | Polarity | 6 | 6 | 6 | 7 | 5 |
| 166 | 3328 | 11156984\_154 | Those cases, underlined in Table 3, may be due either to other constraints on codon usage, |A4:\*\*1SP2E3| |A5:\*\*1SP3E3| such as selection or dinucleotide bias (K ARLIN and BURGE 1995 ), |A4:\*\*2SP3E2| |A5:\*\*2SP3E2| or to an artifact of insufficient sampling. |A4:\*\*3SP3E0| |A1:\*\*1SP1E23| |A2:\*\*1SP2E23| |A3:\*\*1SP1E3| |A5:\*\*3SP3E3| |  |  |  |  |  |  |
|  |  |  |  | Annotation | 1SP1E23 1SP1E23 1SP1E23 | 1SP2E23 1SP2E23 1SP2E23 | 1SP1E3 1SP1E3 1SP1E3 | 1SP2E3 2SP3E2 3SP3E0 | 1SP3E3 2SP3E2 3SP3E3 |
|  |  |  |  | Evidence | 3 3 3 | 3 3 3 | 4 4 4 | 4 3 1 | 4 3 4 |
|  |  |  |  | Focus | 4 4 4 | 4 4 4 | 4 4 4 | 4 4 4 | 4 4 4 |
|  |  |  |  | Polarity | 5 5 5 | 6 6 6 | 5 5 5 | 6 7 7 | 7 7 7 |
| 167 | 8066 | 11472942\_112 | Mutator strains eliminate the need for chemical or physical mutagenesis, |A5:\*\*1SP3E0| but their application has been limited by their genetic instability and by the need to stabilize the strains after successful evolution. |A5:\*\*2SN3E0| |A1:\*\*1GP1E1| |A2:\*\*1SP3E1| |A3:\*\*1SP3E0| |A4:\*\*1SP3E0| |  |  |  |  |  |  |
|  |  |  |  | Annotation | 1GP1E1 1GP1E1 | 1SP3E1 1SP3E1 | 1SP3E0 1SP3E0 | 1SP3E0 1SP3E0 | 1SP3E0 2SN3E0 |
|  |  |  |  | Evidence | 2 2 | 2 2 | 1 1 | 1 1 | 1 1 |
|  |  |  |  | Focus | 1 1 | 4 4 | 4 4 | 4 4 | 4 4 |
|  |  |  |  | Polarity | 5 5 | 7 7 | 7 7 | 7 7 | 7 1 |
| 168 | 8788 | 12421717\_1 | In the amphipod crustacean, Parhyale hawaiensis, the first few embryonic cleavages are total and generate a stereotypical arrangement of cells. |A1:\*\*1GP3E0| |A2:\*\*1SP3E0| |A3:\*\*1SP1E0| |A4:\*\*1SP3E0| |A5:\*\*1SP3E0| |  |  |  |  |  |  |
|  |  |  |  | Annotation | 1GP3E0 | 1SP3E0 | 1SP1E0 | 1SP3E0 | 1SP3E0 |
|  |  |  |  | Evidence | 1 | 1 | 1 | 1 | 1 |
|  |  |  |  | Focus | 1 | 4 | 4 | 4 | 4 |
|  |  |  |  | Polarity | 7 | 7 | 5 | 7 | 7 |
| 169 | 918 | 12821645\_53 | Northern blot analysis revealed strong Fatp4 mRNA expression in Fatp4 +/+ intestine, |A3:\*\*1MSP3E3| |A1:\*\*1SP3E3+| |A5:\*\*1SP3E3+| whereas Fatp4 mRNA in Fatp4 -/- intestine could not be detected, |A3:\*\*2SN3E3| indicating a severe loss-of-function allele of Fatp4 (Fig. 1 D). |A3:\*\*3SP3E3| |A1:\*\*2SN3E3-| |A2:\*\*1SP3E3+| |A4:\*\*1MN3E3-| |A5:\*\*2SN3E3-| |  |  |  |  |  |  |
|  |  |  |  | Annotation | 1SP3E3+ 2SN3E3- 2SN3E3- | 1SP3E3+ 1SP3E3+ 1SP3E3+ | 1MSP3E3 2SN3E3 3SP3E3 | 1MN3E3- 1MN3E3- 1MN3E3- | 1SP3E3+ 2SN3E3- 2SN3E3- |
|  |  |  |  | Evidence | 4 4 4 | 4 4 4 | 4 4 4 | 4 4 4 | 4 4 4 |
|  |  |  |  | Focus | 4 4 4 | 4 4 4 | 6 4 4 | 2 2 2 | 4 4 4 |
|  |  |  |  | Polarity | 7 1 1 | 7 7 7 | 7 1 7 | 1 1 1 | 7 1 1 |
| 170 | 8835 | 9727029\_208 | No other binding partner of SOCS-2 has been reported; |A4:\*\*1SP3E3| specifically, |A3:\*\*1SN3E1| SOCS-2 does not bind to JAK-2 (9). |A3:\*\*2SN3E2| |A1:\*\*1SN3E2| |A2:\*\*1SN3E2| |A4:\*\*2SN3E0| |A5:\*\*1SN3E2| |  |  |  |  |  |  |
|  |  |  |  | Annotation | 1SN3E2 1SN3E2 1SN3E2 | 1SN3E2 1SN3E2 1SN3E2 | 1SN3E1 1SN3E1 2SN3E2 | 1SP3E3 2SN3E0 2SN3E0 | 1SN3E2 1SN3E2 1SN3E2 |
|  |  |  |  | Evidence | 3 3 3 | 3 3 3 | 2 2 3 | 4 1 1 | 3 3 3 |
|  |  |  |  | Focus | 4 4 4 | 4 4 4 | 4 4 4 | 4 4 4 | 4 4 4 |
|  |  |  |  | Polarity | 1 1 1 | 1 1 1 | 1 1 1 | 7 1 1 | 1 1 1 |
| 171 | 4961 | 10629051\_246 | We have previously shown the importance of the p38 MAP kinase pathway |A5:\*\*1SP3E1| on the production of IFN-gamma in CD4+ Th1 effector cells (44). |A5:\*\*2SP3E2+| |A1:\*\*1SP2E2| |A2:\*\*1SP2E2| |A3:\*\*1SP3E2| |A4:\*\*1SP3E2| |  |  |  |  |  |  |
|  |  |  |  | Annotation | 1SP2E2 1SP2E2 | 1SP2E2 1SP2E2 | 1SP3E2 1SP3E2 | 1SP3E2 1SP3E2 | 1SP3E1 2SP3E2+ |
|  |  |  |  | Evidence | 3 3 | 3 3 | 3 3 | 3 3 | 2 3 |
|  |  |  |  | Focus | 4 4 | 4 4 | 4 4 | 4 4 | 4 4 |
|  |  |  |  | Polarity | 6 6 | 6 6 | 7 7 | 7 7 | 7 7 |
| 172 | 1926 | 9750192\_199 | For the first primer set (197F/199R) the PCR conditions were as follows: initial denaturation at 95 degrees C for 5 min, denaturation at 95 degrees C for 15 sec, |A5:\*\*1SP3E3-| annealing at 56 degrees C for 15 sec, and extension at 72 degrees C for 30 sec for a total of 30 cycles. |A5:\*\*2SP3E3+| |A1:\*\*1MP3E3| |A2:\*\*1MP3E3| |A3:\*\*1SP3E3| |A4:\*\*1MP3E3| |  |  |  |  |  |  |
|  |  |  |  | Annotation | 1MP3E3 1MP3E3 | 1MP3E3 1MP3E3 | 1SP3E3 1SP3E3 | 1MP3E3 1MP3E3 | 1SP3E3- 2SP3E3+ |
|  |  |  |  | Evidence | 4 4 | 4 4 | 4 4 | 4 4 | 4 4 |
|  |  |  |  | Focus | 2 2 | 2 2 | 4 4 | 2 2 | 4 4 |
|  |  |  |  | Polarity | 7 7 | 7 7 | 7 7 | 7 7 | 7 7 |
| 173 | 9131 | 11381111\_42 | Using the N-back fMRI paradigm, Mattay et al. recently reported analogous inefficiency in hypodopaminergic patients with Parkinson's disease. |A1:\*\*1MP3E1| |A2:\*\*1SP3E1| |A3:\*\*1SP3E1| |A4:\*\*1SP3E3| |A5:\*\*1MP3E2| |  |  |  |  |  |  |
|  |  |  |  | Annotation | 1MP3E1 | 1SP3E1 | 1SP3E1 | 1SP3E3 | 1MP3E2 |
|  |  |  |  | Evidence | 2 | 2 | 2 | 4 | 3 |
|  |  |  |  | Focus | 2 | 4 | 4 | 4 | 2 |
|  |  |  |  | Polarity | 7 | 7 | 7 | 7 | 7 |
| 174 | 8450 | 9139668\_25 | Fig. 3. Induction of boxA-lacZ RNA synthesis in nusAcs10 and nusA + strains harboring pUV16. |A1:\*\*1SP3E3+| |A2:\*\*1SP3E3+| |A3:\*\*1SP3E3| |A4:\*\*1SP3E3| |A5:\*\*1SP3E3+| |  |  |  |  |  |  |
|  |  |  |  | Annotation | 1SP3E3+ | 1SP3E3+ | 1SP3E3 | 1SP3E3 | 1SP3E3+ |
|  |  |  |  | Evidence | 4 | 4 | 4 | 4 | 4 |
|  |  |  |  | Focus | 4 | 4 | 4 | 4 | 4 |
|  |  |  |  | Polarity | 7 | 7 | 7 | 7 | 7 |
| 175 | 3660 | 11395449\_46 | Primer extension determination of the processing cuts on the RNA transcripts was performed according to techniques described previously (24). |A1:\*\*1MP2E2| |A2:\*\*1MP2E2| |A3:\*\*1SP3E2| |A4:\*\*1MP3E2| |A5:\*\*1MP3E2| |  |  |  |  |  |  |
|  |  |  |  | Annotation | 1MP2E2 | 1MP2E2 | 1SP3E2 | 1MP3E2 | 1MP3E2 |
|  |  |  |  | Evidence | 3 | 3 | 3 | 3 | 3 |
|  |  |  |  | Focus | 2 | 2 | 4 | 2 | 2 |
|  |  |  |  | Polarity | 6 | 6 | 7 | 7 | 7 |
| 176 | 5172 | 12820963\_54 | The ratio of phospho-c-Jun to c-Jun is plotted in the histogram (upper right) as the mean plus-or-minus SD of three experiments (\*p < 0.05). |A1:\*\*1MP2E3| |A2:\*\*1MP2E3| |A3:\*\*1SP3E3| |A4:\*\*1GP3E0| |A5:\*\*1MP3E3| |  |  |  |  |  |  |
|  |  |  |  | Annotation | 1MP2E3 | 1MP2E3 | 1SP3E3 | 1GP3E0 | 1MP3E3 |
|  |  |  |  | Evidence | 4 | 4 | 4 | 1 | 4 |
|  |  |  |  | Focus | 2 | 2 | 4 | 1 | 2 |
|  |  |  |  | Polarity | 6 | 6 | 7 | 7 | 7 |
| 177 | 8506 | 7592715\_64 | The reason for this is not clear |A5:\*\*1GN3E3| but could be due to the unfavorable cleavage bias of A and C residues by the nuclease (see lanes 1 and 2 in Fig. 3 B). |A5:\*\*2SP2E3| |A1:\*\*1SN1E3| |A2:\*\*1SN2E3| |A3:\*\*1SP0E3| |A4:\*\*1SP2E3| |  |  |  |  |  |  |
|  |  |  |  | Annotation | 1SN1E3 1SN1E3 | 1SN2E3 1SN2E3 | 1SP0E3 1SP0E3 | 1SP2E3 1SP2E3 | 1GN3E3 2SP2E3 |
|  |  |  |  | Evidence | 4 4 | 4 4 | 4 4 | 4 4 | 4 4 |
|  |  |  |  | Focus | 4 4 | 4 4 | 4 4 | 4 4 | 1 4 |
|  |  |  |  | Polarity | 3 3 | 2 2 | 4 4 | 6 6 | 1 6 |
| 178 | 3364 | 9244305\_109 | To identify the cleavage site, the in vitro-translated MEKK-1 protein was cleaved with apoptotic MDCK cell extract or caspase-3 and subjected to radiolabeled sequencing (see Experimental Procedures). |A1:\*\*1SP3E3| |A2:\*\*1SP3E3| |A3:\*\*1SP3E3| |A4:\*\*1SP3E3| |A5:\*\*1MP3E3| |  |  |  |  |  |  |
|  |  |  |  | Annotation | 1SP3E3 | 1SP3E3 | 1SP3E3 | 1SP3E3 | 1MP3E3 |
|  |  |  |  | Evidence | 4 | 4 | 4 | 4 | 4 |
|  |  |  |  | Focus | 4 | 4 | 4 | 4 | 2 |
|  |  |  |  | Polarity | 7 | 7 | 7 | 7 | 7 |
| 179 | 4602 | 10799487\_99 | Similar phenomena were observed with the other T213 and T214 mutants, |A3:\*\*1SP3E3| but only in the case of T214V were the low and high spin species almost completely resolved chromatographically. |A3:\*\*2MSP1E3| |A1:\*\*1MP3E3| |A2:\*\*1MP3E3| |A4:\*\*1MP3E3| |A5:\*\*1MP3E3| |  |  |  |  |  |  |
|  |  |  |  | Annotation | 1MP3E3 1MP3E3 | 1MP3E3 1MP3E3 | 1SP3E3 2MSP1E3 | 1MP3E3 1MP3E3 | 1MP3E3 1MP3E3 |
|  |  |  |  | Evidence | 4 4 | 4 4 | 4 4 | 4 4 | 4 4 |
|  |  |  |  | Focus | 2 2 | 2 2 | 4 6 | 2 2 | 2 2 |
|  |  |  |  | Polarity | 7 7 | 7 7 | 7 5 | 7 7 | 7 7 |
| 180 | 5777 | 12663533\_48 | Nucleic acid preparations and manipulations followed standard protocols (A USUBEL et al. 2002 ). |A2:\*\*1SP2E2| |A1:\*\*1GP2E2| |A3:\*\*1SP3E2| |A4:\*\*1SP3E0| |A5:\*\*1MP3E2| |  |  |  |  |  |  |
|  |  |  |  | Annotation | 1GP2E2 | 1SP2E2 | 1SP3E2 | 1SP3E0 | 1MP3E2 |
|  |  |  |  | Evidence | 3 | 3 | 3 | 1 | 3 |
|  |  |  |  | Focus | 1 | 4 | 4 | 4 | 2 |
|  |  |  |  | Polarity | 6 | 6 | 7 | 7 | 7 |
| 181 | 7886 | 9497367\_163 | To test this possibility, as a model unstable protein we have chosen I-kappaB, which becomes extremely short lived after exposure to TNFalpha. |A1:\*\*1SP2E3| |A2:\*\*1SP2E3| |A3:\*\*1SP3E3| |A4:\*\*1SP3E1| |A5:\*\*1SP3E3| |  |  |  |  |  |  |
|  |  |  |  | Annotation | 1SP2E3 | 1SP2E3 | 1SP3E3 | 1SP3E1 | 1SP3E3 |
|  |  |  |  | Evidence | 4 | 4 | 4 | 2 | 4 |
|  |  |  |  | Focus | 4 | 4 | 4 | 4 | 4 |
|  |  |  |  | Polarity | 6 | 6 | 7 | 7 | 7 |
| 182 | 3952 | 9671490\_204 | Expression of this mutant beta-catenin with LEF-1 in transfected Neuro2A cells |A5:\*\*1SP3E3| stimulated the activity of the LEF-CAT reporter gene to a level similar to that with the wt beta-catenin (Fig. 5C). |A5:\*\*2SP3E3+| |A1:\*\*1SP3E3+| |A2:\*\*1SP3E3+| |A3:\*\*1SP3E3| |A4:\*\*1SP3E3| |  |  |  |  |  |  |
|  |  |  |  | Annotation | 1SP3E3+ 1SP3E3+ | 1SP3E3+ 1SP3E3+ | 1SP3E3 1SP3E3 | 1SP3E3 1SP3E3 | 1SP3E3 2SP3E3+ |
|  |  |  |  | Evidence | 4 4 | 4 4 | 4 4 | 4 4 | 4 4 |
|  |  |  |  | Focus | 4 4 | 4 4 | 4 4 | 4 4 | 4 4 |
|  |  |  |  | Polarity | 7 7 | 7 7 | 7 7 | 7 7 | 7 7 |
| 183 | 5747 | 9422604\_61 | A 2.7-kb KpnI- EcoRI fragment from pRHB614 was ligated to pBluescript II KS digested with KpnI and EcoRI, yielding pRHB599. |A1:\*\*1SP3E3| |A2:\*\*1SP3E3| |A3:\*\*1MP3E3| |A4:\*\*1SP3E0| |A5:\*\*1MP3E3| |  |  |  |  |  |  |
|  |  |  |  | Annotation | 1SP3E3 | 1SP3E3 | 1MP3E3 | 1SP3E0 | 1MP3E3 |
|  |  |  |  | Evidence | 4 | 4 | 4 | 1 | 4 |
|  |  |  |  | Focus | 4 | 4 | 2 | 4 | 2 |
|  |  |  |  | Polarity | 7 | 7 | 7 | 7 | 7 |
| 184 | 6255 | 10380930\_360 | The tensor B22 (6.80) is substantially smaller than B11 (36.94) and B33 (45.70). |A1:\*\*1GP3E0| |A2:\*\*1GP3E0| |A3:\*\*1GP3E0| |A4:\*\*1SP3E0| |A5:\*\*1GP3E3| |  |  |  |  |  |  |
|  |  |  |  | Annotation | 1GP3E0 | 1GP3E0 | 1GP3E0 | 1SP3E0 | 1GP3E3 |
|  |  |  |  | Evidence | 1 | 1 | 1 | 1 | 4 |
|  |  |  |  | Focus | 1 | 1 | 1 | 4 | 1 |
|  |  |  |  | Polarity | 7 | 7 | 7 | 7 | 7 |
| 185 | 7026 | 10890911\_19 | Activation of Wnt signaling by binding of Wnt to the Frizzled family of receptors |A5:\*\*1SP3E0| inhibits glycogen-synthase kinase-dependent phosphorylation of beta-catenin |A1:\*\*1SN3E0-| |A2:\*\*1SP3E0-| and |A5:\*\*2SP3E0-| results in an increase in beta-catenin protein levels. |A5:\*\*3SP3E0+| |A1:\*\*2SP3E0+| |A2:\*\*2SP3E0+| |A3:\*\*1SP3E0| |A4:\*\*1SP3E0+| |  |  |  |  |  |  |
|  |  |  |  | Annotation | 1SN3E0- 1SN3E0- 2SP3E0+ 2SP3E0+ | 1SP3E0- 1SP3E0- 2SP3E0+ 2SP3E0+ | 1SP3E0 1SP3E0 1SP3E0 1SP3E0 | 1SP3E0+ 1SP3E0+ 1SP3E0+ 1SP3E0+ | 1SP3E0 2SP3E0- 2SP3E0- 3SP3E0+ |
|  |  |  |  | Evidence | 1 1 1 1 | 1 1 1 1 | 1 1 1 1 | 1 1 1 1 | 1 1 1 1 |
|  |  |  |  | Focus | 4 4 4 4 | 4 4 4 4 | 4 4 4 4 | 4 4 4 4 | 4 4 4 4 |
|  |  |  |  | Polarity | 1 1 7 7 | 7 7 7 7 | 7 7 7 7 | 7 7 7 7 | 7 7 7 7 |
| 186 | 5538 | 11533250\_217 | The kinetics of Wnt pathway-associated genes (encoding beta-catenin, TCF4, Dishevelled-1, Frizzled, axin, and APC) are shown. |A1:\*\*1SP3E3| |A2:\*\*1SP3E1| |A3:\*\*1SP3E3| |A4:\*\*1SP3E3| |A5:\*\*1SP3E3| |  |  |  |  |  |  |
|  |  |  |  | Annotation | 1SP3E3 | 1SP3E1 | 1SP3E3 | 1SP3E3 | 1SP3E3 |
|  |  |  |  | Evidence | 4 | 2 | 4 | 4 | 4 |
|  |  |  |  | Focus | 4 | 4 | 4 | 4 | 4 |
|  |  |  |  | Polarity | 7 | 7 | 7 | 7 | 7 |
| 187 | 6368 | 9145916\_173 | The same reticular pattern of receptor immunofluorescence was seen in the dimmest and the brightest cells. |A1:\*\*1MP3E3| |A2:\*\*1SP3E3| |A3:\*\*1SP3E3| |A4:\*\*1SP3E3| |A5:\*\*1SP3E3| |  |  |  |  |  |  |
|  |  |  |  | Annotation | 1MP3E3 | 1SP3E3 | 1SP3E3 | 1SP3E3 | 1SP3E3 |
|  |  |  |  | Evidence | 4 | 4 | 4 | 4 | 4 |
|  |  |  |  | Focus | 2 | 4 | 4 | 4 | 4 |
|  |  |  |  | Polarity | 7 | 7 | 7 | 7 | 7 |
| 188 | 3147 | 9418906\_104 | Each DNA fragment was subcloned into the respective restriction sites of the pUAST vector. |A1:\*\*1SP3E3| |A2:\*\*1MP3E3| |A3:\*\*1MP3E3| |A4:\*\*1SP3E0| |A5:\*\*1SP3E3| |  |  |  |  |  |  |
|  |  |  |  | Annotation | 1SP3E3 | 1MP3E3 | 1MP3E3 | 1SP3E0 | 1SP3E3 |
|  |  |  |  | Evidence | 4 | 4 | 4 | 1 | 4 |
|  |  |  |  | Focus | 4 | 2 | 2 | 4 | 4 |
|  |  |  |  | Polarity | 7 | 7 | 7 | 7 | 7 |
| 189 | 3193 | 10611225\_196 | Twenty percent of the input His-tagged protein was subjected to SDS-PAGE followed by Western blotting with anti-His antibody (upper panel). |A1:\*\*1MP3E3| |A2:\*\*1MP3E3| |A3:\*\*1MP3E3| |A4:\*\*1MP3E3| |A5:\*\*1MP3E3| |  |  |  |  |  |  |
|  |  |  |  | Annotation | 1MP3E3 | 1MP3E3 | 1MP3E3 | 1MP3E3 | 1MP3E3 |
|  |  |  |  | Evidence | 4 | 4 | 4 | 4 | 4 |
|  |  |  |  | Focus | 2 | 2 | 2 | 2 | 2 |
|  |  |  |  | Polarity | 7 | 7 | 7 | 7 | 7 |
| 190 | 7754 | 11371357\_206 | GFP, a self-replenishing fluorescent marker, allows identification and isolation of transferred effector cells in a real-time mode, |A2:\*\*1SP3E0| |A3:\*\*1SP3E0| |A5:\*\*1GP3E2| while it does not interfere with the T cells' encephalitogenic function (Flu gel et al., 1999). |A2:\*\*1SN3E2| |A1:\*\*1GP3E2| |A3:\*\*2SN3E2| |A4:\*\*1SP3E2| |A5:\*\*2GN3E2| |  |  |  |  |  |  |
|  |  |  |  | Annotation | 1GP3E2 1GP3E2 | 1SP3E0 1SN3E2 | 1SP3E0 2SN3E2 | 1SP3E2 1SP3E2 | 1GP3E2 2GN3E2 |
|  |  |  |  | Evidence | 3 3 | 1 3 | 1 3 | 3 3 | 3 3 |
|  |  |  |  | Focus | 1 1 | 4 4 | 4 4 | 4 4 | 1 1 |
|  |  |  |  | Polarity | 7 7 | 7 1 | 7 1 | 7 7 | 7 1 |
| 191 | 5965 | 9844016\_84 | The linear range of amplification was determined in a 30-cycle RT-PCR amplification of reverse transcribed, randomly primed cDNAs, with increasing amounts (0-400 ng) of starting total RNA obtained from one schizophrenic brain (Fig. 3 A). |A1:\*\*1SP3E3+| |A2:\*\*1MP3E3| |A3:\*\*1MSP3E3+| |A4:\*\*1SP3E3| |A5:\*\*1MP3E3| |  |  |  |  |  |  |
|  |  |  |  | Annotation | 1SP3E3+ | 1MP3E3 | 1MSP3E3+ | 1SP3E3 | 1MP3E3 |
|  |  |  |  | Evidence | 4 | 4 | 4 | 4 | 4 |
|  |  |  |  | Focus | 4 | 2 | 6 | 4 | 2 |
|  |  |  |  | Polarity | 7 | 7 | 7 | 7 | 7 |
| 192 | 2567 | 10523653\_174 | A 750-bp PstI fragment internal to the PTP2 open reading frame, a 1-kb ClaI fragment internal to the PTP3 open reading frame, and a 1.1-kb BglII- ClaI fragment from TUB1 were used to produce 32P-labeled probes (63). |A1:\*\*1SP3E23| |A2:\*\*1SP3E23| |A3:\*\*1MSP3E3| |A4:\*\*1SP3E2| |A5:\*\*1SP3E2| |  |  |  |  |  |  |
|  |  |  |  | Annotation | 1SP3E23 | 1SP3E23 | 1MSP3E3 | 1SP3E2 | 1SP3E2 |
|  |  |  |  | Evidence | 3 | 3 | 4 | 3 | 3 |
|  |  |  |  | Focus | 4 | 4 | 6 | 4 | 4 |
|  |  |  |  | Polarity | 7 | 7 | 7 | 7 | 7 |
| 193 | 5720 | 10671457\_74 | Samples were incubated at 60 degrees C for 5 min, |A5:\*\*1MP3E3| and the reaction was stopped by adding 25 mul of 20% formic acid. |A5:\*\*2MP3E3-| |A1:\*\*1MP3E3| |A2:\*\*1SP3E3| |A3:\*\*1SP3E3| |A4:\*\*1MP3E3| |  |  |  |  |  |  |
|  |  |  |  | Annotation | 1MP3E3 1MP3E3 | 1SP3E3 1SP3E3 | 1SP3E3 1SP3E3 | 1MP3E3 1MP3E3 | 1MP3E3 2MP3E3- |
|  |  |  |  | Evidence | 4 4 | 4 4 | 4 4 | 4 4 | 4 4 |
|  |  |  |  | Focus | 2 2 | 4 4 | 4 4 | 2 2 | 2 2 |
|  |  |  |  | Polarity | 7 7 | 7 7 | 7 7 | 7 7 | 7 7 |
| 194 | 5168 | 12058040\_46 | Arrow indicates the Pus3p protein; M indicates molecular weight markers in thousands. |A1:\*\*1SP3E0| |A2:\*\*1SP3E0| |A3:\*\*1SP3E0| |A4:\*\*1SP3E3| |A5:\*\*1GP3E3| |  |  |  |  |  |  |
|  |  |  |  | Annotation | 1SP3E0 | 1SP3E0 | 1SP3E0 | 1SP3E3 | 1GP3E3 |
|  |  |  |  | Evidence | 1 | 1 | 1 | 4 | 4 |
|  |  |  |  | Focus | 4 | 4 | 4 | 4 | 1 |
|  |  |  |  | Polarity | 7 | 7 | 7 | 7 | 7 |
| 195 | 4953 | 12481028\_7 | Valproic acid (2 mmol/kg for 15 days, twice a day) reverted L-methionine-induced down-regulation of reelin and GAD67 in both WT and heterozygous reeler mice, |A5:\*\*1MP3E3| suggesting an epigenetic action through the inhibition of histone deacetylases. |A5:\*\*2SP2E0| |A1:\*\*1SN3E3-| |A2:\*\*1SN3E3-| |A3:\*\*1SP3E3-| |A4:\*\*1SN2E1-| |  |  |  |  |  |  |
|  |  |  |  | Annotation | 1SN3E3- 1SN3E3- | 1SN3E3- 1SN3E3- | 1SP3E3- 1SP3E3- | 1SN2E1- 1SN2E1- | 1MP3E3 2SP2E0 |
|  |  |  |  | Evidence | 4 4 | 4 4 | 4 4 | 2 2 | 4 1 |
|  |  |  |  | Focus | 4 4 | 4 4 | 4 4 | 4 4 | 2 4 |
|  |  |  |  | Polarity | 1 1 | 1 1 | 7 7 | 2 2 | 7 6 |
| 196 | 8672 | 11500393\_48 | Clinical isolates of Pseudomonas aeruginosa and Staphylococcus aureus were obtained from the clinical microbiology laboratory at the Shriners Hospital for Children, Galveston Burns Unit, and were heat-killed at 56 degrees C for 1 h. |A1:\*\*1SP3E3| |A2:\*\*1SP3E3| |A3:\*\*1SP3E3| |A4:\*\*1SP3E3| |A5:\*\*1GP3E3| |  |  |  |  |  |  |
|  |  |  |  | Annotation | 1SP3E3 | 1SP3E3 | 1SP3E3 | 1SP3E3 | 1GP3E3 |
|  |  |  |  | Evidence | 4 | 4 | 4 | 4 | 4 |
|  |  |  |  | Focus | 4 | 4 | 4 | 4 | 1 |
|  |  |  |  | Polarity | 7 | 7 | 7 | 7 | 7 |
| 197 | 7727 | 12087098\_77 | The kinase activity was determined as the ratio of radioactive signal versus Western signal. |A1:\*\*1SP3E3| |A2:\*\*1SP3E3| |A3:\*\*1SP3E0| |A4:\*\*1SP3E3| |A5:\*\*1MP3E3| |  |  |  |  |  |  |
|  |  |  |  | Annotation | 1SP3E3 | 1SP3E3 | 1SP3E0 | 1SP3E3 | 1MP3E3 |
|  |  |  |  | Evidence | 4 | 4 | 1 | 4 | 4 |
|  |  |  |  | Focus | 4 | 4 | 4 | 4 | 2 |
|  |  |  |  | Polarity | 7 | 7 | 7 | 7 | 7 |
| 198 | 334 | 9570815\_130 | The analysis in Figure 1 B suggests that in five cases the side cuts were largely successful. |A1:\*\*1GP2E3| |A2:\*\*1GP2E3| |A3:\*\*1GP2E3| |A4:\*\*1SP2E3| |A5:\*\*1GP3E3| |  |  |  |  |  |  |
|  |  |  |  | Annotation | 1GP2E3 | 1GP2E3 | 1GP2E3 | 1SP2E3 | 1GP3E3 |
|  |  |  |  | Evidence | 4 | 4 | 4 | 4 | 4 |
|  |  |  |  | Focus | 1 | 1 | 1 | 4 | 1 |
|  |  |  |  | Polarity | 6 | 6 | 6 | 6 | 7 |
| 199 | 2922 | 10197535\_25 | Mutations in the M3C and M4N segments, |A5:\*\*1GP3E0| but not in the preM1 segment, |A5:\*\*2GN3E0| exhibited shifts of the reversal potential for Ca2+ Ca2 subsequent to covalent modification. |A5:\*\*3GP3E0| |A1:\*\*1SP3E3| |A2:\*\*1SP3E3| |A3:\*\*1SN3E3| |A4:\*\*1SN3E3| |  |  |  |  |  |  |
|  |  |  |  | Annotation | 1SP3E3 1SP3E3 1SP3E3 | 1SP3E3 1SP3E3 1SP3E3 | 1SN3E3 1SN3E3 1SN3E3 | 1SN3E3 1SN3E3 1SN3E3 | 1GP3E0 2GN3E0 3GP3E0 |
|  |  |  |  | Evidence | 4 4 4 | 4 4 4 | 4 4 4 | 4 4 4 | 1 1 1 |
|  |  |  |  | Focus | 4 4 4 | 4 4 4 | 4 4 4 | 4 4 4 | 1 1 1 |
|  |  |  |  | Polarity | 7 7 7 | 7 7 7 | 1 1 1 | 1 1 1 | 7 1 7 |
| 200 | 4240 | 11909946\_1 | Hypoxia (low-oxygen tension) is an important physiological stress that influences responses to a wide range of pathologies, including stroke, infarction, and tumorigenesis. |A1:\*\*1GP3E0| |A2:\*\*1SP3E0| |A3:\*\*1SP3E0| |A4:\*\*1SP3E0| |A5:\*\*1GP3E0| |  |  |  |  |  |  |
|  |  |  |  | Annotation | 1GP3E0 | 1SP3E0 | 1SP3E0 | 1SP3E0 | 1GP3E0 |
|  |  |  |  | Evidence | 1 | 1 | 1 | 1 | 1 |
|  |  |  |  | Focus | 1 | 4 | 4 | 4 | 1 |
|  |  |  |  | Polarity | 7 | 7 | 7 | 7 | 7 |
| 201 | 7837 | 12359197\_4 | The daily aspiration required significantly more attendances for aspiration (P<0.005) |A1:\*\*1GP3E3| |A2:\*\*1SP3E3| |A3:\*\*1SP2E3| and the time from surgery to final aspiration was not reduced. CONCLUSION: |A1:\*\*2GN3E3| |A2:\*\*1SN3E3| |A3:\*\*1SN3E3| |A4:\*\*1GN2E0| |A5:\*\*1SP3E3| |  |  |  |  |  |  |
|  |  |  |  | Annotation | 1GP3E3 2GN3E3 | 1SP3E3 1SN3E3 | 1SP2E3 1SN3E3 | 1GN2E0 1GN2E0 | 1SP3E3 1SP3E3 |
|  |  |  |  | Evidence | 4 4 | 4 4 | 4 4 | 1 1 | 4 4 |
|  |  |  |  | Focus | 1 1 | 4 4 | 4 4 | 1 1 | 4 4 |
|  |  |  |  | Polarity | 7 1 | 7 1 | 6 1 | 2 2 | 7 7 |
| 202 | 7243 | 11278880\_53 | CD9P-1 is the protein coded by the KIAA1436 gene ( ). |A1:\*\*1SP3E2| |A2:\*\*1SP3E2| |A3:\*\*1SP3E0| |A4:\*\*1SP3E0| |A5:\*\*1GP3E2| |  |  |  |  |  |  |
|  |  |  |  | Annotation | 1SP3E2 | 1SP3E2 | 1SP3E0 | 1SP3E0 | 1GP3E2 |
|  |  |  |  | Evidence | 3 | 3 | 1 | 1 | 3 |
|  |  |  |  | Focus | 4 | 4 | 4 | 4 | 1 |
|  |  |  |  | Polarity | 7 | 7 | 7 | 7 | 7 |
| 203 | 2163 | 11948703\_2 | Although its mechanism of action remains unknown, |A3:\*\*1SP0E0| |A5:\*\*1GP0E0| irradiated T-helper cell-induced immunosuppression is the main theory. |A3:\*\*1SP2E0| |A1:\*\*1GP0E0| |A2:\*\*1SN0E0| |A4:\*\*1SP0E0| |A5:\*\*2SP1E0| |  |  |  |  |  |  |
|  |  |  |  | Annotation | 1GP0E0 1GP0E0 | 1SN0E0 1SN0E0 | 1SP0E0 1SP2E0 | 1SP0E0 1SP0E0 | 1GP0E0 2SP1E0 |
|  |  |  |  | Evidence | 1 1 | 1 1 | 1 1 | 1 1 | 1 1 |
|  |  |  |  | Focus | 1 1 | 4 4 | 4 4 | 4 4 | 1 4 |
|  |  |  |  | Polarity | 4 4 | 4 4 | 4 6 | 4 4 | 4 5 |
| 204 | 8117 | 11021528\_12 | This interaction results in an increased T cell proliferation, IL-2 production, |A1:\*\*1SP3E3+| and resistance to apoptosis (Linsley et al. 1991a ; Croft et al. 1992). |A1:\*\*2SP3E23+| |A2:\*\*1SP3E23+| |A3:\*\*1SP3E2| |A4:\*\*1SP3E2+| |A5:\*\*1SP3E2+| |  |  |  |  |  |  |
|  |  |  |  | Annotation | 1SP3E3+ 2SP3E23+ | 1SP3E23+ 1SP3E23+ | 1SP3E2 1SP3E2 | 1SP3E2+ 1SP3E2+ | 1SP3E2+ 1SP3E2+ |
|  |  |  |  | Evidence | 4 3 | 3 3 | 3 3 | 3 3 | 3 3 |
|  |  |  |  | Focus | 4 4 | 4 4 | 4 4 | 4 4 | 4 4 |
|  |  |  |  | Polarity | 7 7 | 7 7 | 7 7 | 7 7 | 7 7 |
| 205 | 1934 | 9437015\_85 | An intensity ratio of 1.0 represented cytoplasmic fluorescence equal to that in the nucleus. |A1:\*\*1GP3E0| |A2:\*\*1SP3E0| |A3:\*\*1SP3E0| |A4:\*\*1SP3E0| |A5:\*\*1SP2E3| |  |  |  |  |  |  |
|  |  |  |  | Annotation | 1GP3E0 | 1SP3E0 | 1SP3E0 | 1SP3E0 | 1SP2E3 |
|  |  |  |  | Evidence | 1 | 1 | 1 | 1 | 4 |
|  |  |  |  | Focus | 1 | 4 | 4 | 4 | 4 |
|  |  |  |  | Polarity | 7 | 7 | 7 | 7 | 6 |
| 206 | 5208 | 12620409\_324 | The gradients were centrifuged in an SW41 Beckman rotor at 38,000 rpm for 120 min at 4 degrees C. Fourteen fractions of equal volume (750 l) were collected from the bottom of the tubes. |A1:\*\*1MP3E3| |A2:\*\*1MP3E3| |A3:\*\*1MP3E3| |A4:\*\*1MP3E3| |A5:\*\*1MP3E3| |  |  |  |  |  |  |
|  |  |  |  | Annotation | 1MP3E3 | 1MP3E3 | 1MP3E3 | 1MP3E3 | 1MP3E3 |
|  |  |  |  | Evidence | 4 | 4 | 4 | 4 | 4 |
|  |  |  |  | Focus | 2 | 2 | 2 | 2 | 2 |
|  |  |  |  | Polarity | 7 | 7 | 7 | 7 | 7 |
| 207 | 2007 | 11861554\_195 | We conclude that rad-51 is involved in resistance to IR during embryonic growth. |A1:\*\*1SP3E1| |A2:\*\*1SP3E3| |A3:\*\*1SP3E3| |A4:\*\*1SP3E3-| |A5:\*\*1SP3E3| |  |  |  |  |  |  |
|  |  |  |  | Annotation | 1SP3E1 | 1SP3E3 | 1SP3E3 | 1SP3E3- | 1SP3E3 |
|  |  |  |  | Evidence | 2 | 4 | 4 | 4 | 4 |
|  |  |  |  | Focus | 4 | 4 | 4 | 4 | 4 |
|  |  |  |  | Polarity | 7 | 7 | 7 | 7 | 7 |
| 208 | 2498 | 11438670\_259 | These cells were also stained for the mitosis-specific phosphorylated form of histone H3 to unambiguously distinguish mitotic cells (18). |A1:\*\*1SP3E23| |A2:\*\*1SP3E23| |A3:\*\*1SP3E3| |A4:\*\*1SP0E0| |A5:\*\*1SP3E2| |  |  |  |  |  |  |
|  |  |  |  | Annotation | 1SP3E23 | 1SP3E23 | 1SP3E3 | 1SP0E0 | 1SP3E2 |
|  |  |  |  | Evidence | 3 | 3 | 4 | 1 | 3 |
|  |  |  |  | Focus | 4 | 4 | 4 | 4 | 4 |
|  |  |  |  | Polarity | 7 | 7 | 7 | 4 | 7 |
| 209 | 6773 | 11257128\_140 | Demyelination often began at one end of the myelin sheath, |A5:\*\*1SP3E3-| progressing from the paranodal region to the Schwann cell internode (data not shown). |A5:\*\*2SP3E3+| |A1:\*\*1SP3E1| |A2:\*\*1SP3E1| |A3:\*\*1SP3E3| |A4:\*\*1SP3E3| |  |  |  |  |  |  |
|  |  |  |  | Annotation | 1SP3E1 1SP3E1 | 1SP3E1 1SP3E1 | 1SP3E3 1SP3E3 | 1SP3E3 1SP3E3 | 1SP3E3- 2SP3E3+ |
|  |  |  |  | Evidence | 2 2 | 2 2 | 4 4 | 4 4 | 4 4 |
|  |  |  |  | Focus | 4 4 | 4 4 | 4 4 | 4 4 | 4 4 |
|  |  |  |  | Polarity | 7 7 | 7 7 | 7 7 | 7 7 | 7 7 |
| 210 | 8119 | 10508204\_96 | Tissues comprising liver, heart, lungs, intestine and stomach, spleen, kidneys, and aorta were excised, blotted dry on lint-free tissues, and placed in sample containers on ice to be stored at -80 degrees C until analysis. |A1:\*\*1MP3E3| |A2:\*\*1MP3E3| |A3:\*\*1MP3E3| |A4:\*\*1SP3E3| |A5:\*\*1MP3E3| |  |  |  |  |  |  |
|  |  |  |  | Annotation | 1MP3E3 | 1MP3E3 | 1MP3E3 | 1SP3E3 | 1MP3E3 |
|  |  |  |  | Evidence | 4 | 4 | 4 | 4 | 4 |
|  |  |  |  | Focus | 2 | 2 | 2 | 4 | 2 |
|  |  |  |  | Polarity | 7 | 7 | 7 | 7 | 7 |
| 211 | 3966 | 12107429\_5 | Northern blotting of embryonic stages demonstrated a 3.2-kb transcript present in several embryonic tissues, including kidney, brain, heart, and lung, in a fashion confirmatory with the RNA-fingerprinting data. |A1:\*\*1MP3E3| |A2:\*\*1SP3E3| |A3:\*\*1MSP3E3| |A4:\*\*1MP3E3| |A5:\*\*1MP3E3| |  |  |  |  |  |  |
|  |  |  |  | Annotation | 1MP3E3 | 1SP3E3 | 1MSP3E3 | 1MP3E3 | 1MP3E3 |
|  |  |  |  | Evidence | 4 | 4 | 4 | 4 | 4 |
|  |  |  |  | Focus | 2 | 4 | 6 | 2 | 2 |
|  |  |  |  | Polarity | 7 | 7 | 7 | 7 | 7 |
| 212 | 9283 | 11731474\_1 | The Wnt-responsive transcription factor LEF1 can activate transcription in association with beta-catenin |A1:\*\*1SP2E0+| |A2:\*\*1SP1E0+| |A5:\*\*1SP3E0+| and repress transcription in association with Groucho. |A1:\*\*2SN3E0| |A2:\*\*1SP3E0-| |A3:\*\*1SP3E0| |A4:\*\*1SP3E3| |A5:\*\*2SP3E0-| |  |  |  |  |  |  |
|  |  |  |  | Annotation | 1SP2E0+ 2SN3E0 | 1SP1E0+ 1SP3E0- | 1SP3E0 1SP3E0 | 1SP3E3 1SP3E3 | 1SP3E0+ 2SP3E0- |
|  |  |  |  | Evidence | 1 1 | 1 1 | 1 1 | 4 4 | 1 1 |
|  |  |  |  | Focus | 4 4 | 4 4 | 4 4 | 4 4 | 4 4 |
|  |  |  |  | Polarity | 6 1 | 5 7 | 7 7 | 7 7 | 7 7 |
| 213 | 5900 | 12530981\_103 | Cell lysates from Tpn / cells +/ mK3 /cells +/mK3 and +/ Tpn +/Tpn reconstitution were blotted with anti-tapasin, -mK3, and -GFP antibodies. |A1:\*\*1MP3E3| |A2:\*\*1MP3E3| |A3:\*\*1SP3E3| |A4:\*\*1SP3E3| |A5:\*\*1MP3E3| |  |  |  |  |  |  |
|  |  |  |  | Annotation | 1MP3E3 | 1MP3E3 | 1SP3E3 | 1SP3E3 | 1MP3E3 |
|  |  |  |  | Evidence | 4 | 4 | 4 | 4 | 4 |
|  |  |  |  | Focus | 2 | 2 | 4 | 4 | 2 |
|  |  |  |  | Polarity | 7 | 7 | 7 | 7 | 7 |
| 214 | 4945 | 11466270\_83 | Outer membrane protein profiles (12) of bacteria and the ability of bacteria to attach to plastic ( 35) were assayed as described elsewhere. |A1:\*\*1GP0E23| |A2:\*\*1SP3E23| |A3:\*\*1SP3E2| |A4:\*\*1SP3E1| |A5:\*\*1MP3E2| |  |  |  |  |  |  |
|  |  |  |  | Annotation | 1GP0E23 | 1SP3E23 | 1SP3E2 | 1SP3E1 | 1MP3E2 |
|  |  |  |  | Evidence | 3 | 3 | 3 | 2 | 3 |
|  |  |  |  | Focus | 1 | 4 | 4 | 4 | 2 |
|  |  |  |  | Polarity | 4 | 7 | 7 | 7 | 7 |
| 215 | 7607 | 10348843\_160 | Deletion of MID2 in two viable but slow-growing beta-1,6-glucan beta-1, 6-glucan synthesis mutants, kre6delta (42) and kre9delta (5), |A4:\*\*1SP3E2-| |A5:\*\*1SP3E2| partially restores growth rate (Fig. 3A, and B). |A4:\*\*2SP2E3| |A1:\*\*1SP3E23| |A2:\*\*1SP3E23| |A3:\*\*1SP1E2| |A5:\*\*2SP3E3+| |  |  |  |  |  |  |
|  |  |  |  | Annotation | 1SP3E23 1SP3E23 | 1SP3E23 1SP3E23 | 1SP1E2 1SP1E2 | 1SP3E2- 2SP2E3 | 1SP3E2 2SP3E3+ |
|  |  |  |  | Evidence | 3 3 | 3 3 | 3 3 | 3 4 | 3 4 |
|  |  |  |  | Focus | 4 4 | 4 4 | 4 4 | 4 4 | 4 4 |
|  |  |  |  | Polarity | 7 7 | 7 7 | 5 5 | 7 6 | 7 7 |
| 216 | 1428 | 7592782\_12 | There are two optima for the inactivation rate: one at pH 6.0-7.0, and the other at approximately pH 4.0 (Fig. 2). |A1:\*\*1SP3E3| |A2:\*\*1SP3E3-| |A3:\*\*1SP3E3| |A4:\*\*1SP2E3| |A5:\*\*1GP3E3| |  |  |  |  |  |  |
|  |  |  |  | Annotation | 1SP3E3 | 1SP3E3- | 1SP3E3 | 1SP2E3 | 1GP3E3 |
|  |  |  |  | Evidence | 4 | 4 | 4 | 4 | 4 |
|  |  |  |  | Focus | 4 | 4 | 4 | 4 | 1 |
|  |  |  |  | Polarity | 7 | 7 | 7 | 6 | 7 |
| 217 | 4047 | 9971740\_35 | More recent ultrastructural analyses have shown that this axonal enlargement is caused by an accumulation of neurofilaments |A4:\*\*1SP3E1| and membranous organelles proximal to the site of constriction (e.g., Schmidt and Plurad, 1985 ; LeBeau et al., 1988 ). |A1:\*\*1SP3E2| |A2:\*\*1SP3E2| |A3:\*\*1SP2E2| |A4:\*\*2SP3E2| |A5:\*\*1SP3E2+| |  |  |  |  |  |  |
|  |  |  |  | Annotation | 1SP3E2 1SP3E2 | 1SP3E2 1SP3E2 | 1SP2E2 1SP2E2 | 1SP3E1 2SP3E2 | 1SP3E2+ 1SP3E2+ |
|  |  |  |  | Evidence | 3 3 | 3 3 | 3 3 | 2 3 | 3 3 |
|  |  |  |  | Focus | 4 4 | 4 4 | 4 4 | 4 4 | 4 4 |
|  |  |  |  | Polarity | 7 7 | 7 7 | 6 6 | 7 7 | 7 7 |
| 218 | 9917 | 12091905\_1 | Staphylococcus aureus is a human pathogen that secretes proteins that contribute to bacterial colonization. |A1:\*\*1GP3E0| |A2:\*\*1SP3E0| |A3:\*\*1SP3E0| |A4:\*\*1SP3E0| |A5:\*\*1SP3E0+| |  |  |  |  |  |  |
|  |  |  |  | Annotation | 1GP3E0 | 1SP3E0 | 1SP3E0 | 1SP3E0 | 1SP3E0+ |
|  |  |  |  | Evidence | 1 | 1 | 1 | 1 | 1 |
|  |  |  |  | Focus | 1 | 4 | 4 | 4 | 4 |
|  |  |  |  | Polarity | 7 | 7 | 7 | 7 | 7 |
| 219 | 8689 | 9390512\_167 | It has been estimated that systematic mapping by association requires a marker map of density at least 100-fold greater greaterR than that for linkage mapping. |A1:\*\*1GP2E0| |A2:\*\*1SP2E0| |A3:\*\*1SP2E0| |A4:\*\*1SP3E1+| |A5:\*\*1SP2E1| |  |  |  |  |  |  |
|  |  |  |  | Annotation | 1GP2E0 | 1SP2E0 | 1SP2E0 | 1SP3E1+ | 1SP2E1 |
|  |  |  |  | Evidence | 1 | 1 | 1 | 2 | 2 |
|  |  |  |  | Focus | 1 | 4 | 4 | 4 | 4 |
|  |  |  |  | Polarity | 6 | 6 | 6 | 7 | 6 |
| 220 | 7329 | 12230083\_1 | Mental health services in the treatment of late-life depression are critical in the primary care arena. |A1:\*\*1GP3E0| |A2:\*\*1GP3E0| |A3:\*\*1SP3E0| |A4:\*\*1GP3E0| |A5:\*\*1SP3E0| |  |  |  |  |  |  |
|  |  |  |  | Annotation | 1GP3E0 | 1GP3E0 | 1SP3E0 | 1GP3E0 | 1SP3E0 |
|  |  |  |  | Evidence | 1 | 1 | 1 | 1 | 1 |
|  |  |  |  | Focus | 1 | 1 | 4 | 1 | 4 |
|  |  |  |  | Polarity | 7 | 7 | 7 | 7 | 7 |
| 221 | 3737 | 9488491\_120 | Supershift experiments with antibodies against E2F indicated that E2F4 is the major family member |A5:\*\*1MP3E3| whose levels were increased in the presence of TAg (data not shown). |A5:\*\*2MP3E3+| |A1:\*\*1SP3E1+| |A2:\*\*1SP3E1+| |A3:\*\*1MSP2E3| |A4:\*\*1MP3E3+| |  |  |  |  |  |  |
|  |  |  |  | Annotation | 1SP3E1+ 1SP3E1+ | 1SP3E1+ 1SP3E1+ | 1MSP2E3 1MSP2E3 | 1MP3E3+ 1MP3E3+ | 1MP3E3 2MP3E3+ |
|  |  |  |  | Evidence | 2 2 | 2 2 | 4 4 | 4 4 | 4 4 |
|  |  |  |  | Focus | 4 4 | 4 4 | 6 6 | 2 2 | 2 2 |
|  |  |  |  | Polarity | 7 7 | 7 7 | 6 6 | 7 7 | 7 7 |
| 222 | 7415 | 12370247\_99 | Akr1p by itself is apparently sufficient for activity. |A1:\*\*1SP3E0| |A2:\*\*1SP3E0| |A3:\*\*1SP3E0| |A4:\*\*1SP2E0| |A5:\*\*1GP3E0| |  |  |  |  |  |  |
|  |  |  |  | Annotation | 1SP3E0 | 1SP3E0 | 1SP3E0 | 1SP2E0 | 1GP3E0 |
|  |  |  |  | Evidence | 1 | 1 | 1 | 1 | 1 |
|  |  |  |  | Focus | 4 | 4 | 4 | 4 | 1 |
|  |  |  |  | Polarity | 7 | 7 | 7 | 6 | 7 |
| 223 | 687 | 11950881\_2 | The cadherin family of cell adhesion receptors, located in the adherens junction, interact homophilically to mediate strong cell-cell adhesion. |A1:\*\*1SP3E0| |A2:\*\*1SP3E0| |A3:\*\*1SP3E0| |A4:\*\*1SP3E0| |A5:\*\*1SP3E0| |  |  |  |  |  |  |
|  |  |  |  | Annotation | 1SP3E0 | 1SP3E0 | 1SP3E0 | 1SP3E0 | 1SP3E0 |
|  |  |  |  | Evidence | 1 | 1 | 1 | 1 | 1 |
|  |  |  |  | Focus | 4 | 4 | 4 | 4 | 4 |
|  |  |  |  | Polarity | 7 | 7 | 7 | 7 | 7 |
| 224 | 5816 | 11438517\_147 | Cells stably expressing 3Rtau or 4Rtau were treated as described for Fig. 5. |A2:\*\*1SP3E3| |A3:\*\*1MP3E3| |A4:\*\*1SP3E3| Cells were scraped into sample buffer without DTT and proteins separated by SDS-PAGE, Western-blotted, and probed with a polyclonal antibody to tau, TP70. a, 3RtauCHO cells; b, 4RtauCHO cells. |A2:\*\*1MP3E3| |A1:\*\*1MP3E3| |A3:\*\*2MP3E3| |A4:\*\*2MP3E3| |A5:\*\*1MP3E3| |  |  |  |  |  |  |
|  |  |  |  | Annotation | 1MP3E3 1MP3E3 | 1SP3E3 1MP3E3 | 1MP3E3 2MP3E3 | 1SP3E3 2MP3E3 | 1MP3E3 1MP3E3 |
|  |  |  |  | Evidence | 4 4 | 4 4 | 4 4 | 4 4 | 4 4 |
|  |  |  |  | Focus | 2 2 | 4 2 | 2 2 | 4 2 | 2 2 |
|  |  |  |  | Polarity | 7 7 | 7 7 | 7 7 | 7 7 | 7 7 |
| 225 | 2591 | 9700168\_39 | Northern blot analysis of total RNA (20 mug/lane) was performed |A5:\*\*1MP3E3| as previously described (Lehrach et al., 1977). |A5:\*\*2MP3E2| |A1:\*\*1MP3E23| |A2:\*\*1MP3E23| |A3:\*\*1MP3E2| |A4:\*\*1SP3E2| |  |  |  |  |  |  |
|  |  |  |  | Annotation | 1MP3E23 1MP3E23 | 1MP3E23 1MP3E23 | 1MP3E2 1MP3E2 | 1SP3E2 1SP3E2 | 1MP3E3 2MP3E2 |
|  |  |  |  | Evidence | 3 3 | 3 3 | 3 3 | 3 3 | 4 3 |
|  |  |  |  | Focus | 2 2 | 2 2 | 2 2 | 4 4 | 2 2 |
|  |  |  |  | Polarity | 7 7 | 7 7 | 7 7 | 7 7 | 7 7 |
| 226 | 8515 | 8548831\_198 | Whereas only the human acrocentric chromosomes 13, 14, 15, 21, and 22 have rDNA repeats and secondary constrictions, all human chromosomes have a primary constriction at the site of the centromere. |A1:\*\*1SP3E0| |A2:\*\*1SP3E0| |A3:\*\*1SP3E0| |A4:\*\*1SP3E0| |A5:\*\*1SP3E1| |  |  |  |  |  |  |
|  |  |  |  | Annotation | 1SP3E0 | 1SP3E0 | 1SP3E0 | 1SP3E0 | 1SP3E1 |
|  |  |  |  | Evidence | 1 | 1 | 1 | 1 | 2 |
|  |  |  |  | Focus | 4 | 4 | 4 | 4 | 4 |
|  |  |  |  | Polarity | 7 | 7 | 7 | 7 | 7 |
| 227 | 5634 | 12132659\_12 | Therefore, emotionally-induced sweating can enhance the release of organic substrates. |A1:\*\*1GP1E1| |A2:\*\*1GP2E1+| |A3:\*\*1SP3E0| |A4:\*\*1SP3E0| |A5:\*\*1SP3E1| |  |  |  |  |  |  |
|  |  |  |  | Annotation | 1GP1E1 | 1GP2E1+ | 1SP3E0 | 1SP3E0 | 1SP3E1 |
|  |  |  |  | Evidence | 2 | 2 | 1 | 1 | 2 |
|  |  |  |  | Focus | 1 | 1 | 4 | 4 | 4 |
|  |  |  |  | Polarity | 5 | 6 | 7 | 7 | 7 |
| 228 | 2907 | 9657992\_89 | After the addition of 5 mul of 0.25x Tris-borate-EDTA (TBE) containing 40% glycerol, incubated samples were run on a 6% polyacrylamide gel (79:1) in 0.25x TBE, followed by electroblotting onto a nylon membrane (Hybond-N; Amersham) and fixation by UV cross-linking. |A1:\*\*1MP3E3| |A2:\*\*1MP3E3| |A3:\*\*1MP3E3| |A4:\*\*1MP3E3| |A5:\*\*1MP3E3| |  |  |  |  |  |  |
|  |  |  |  | Annotation | 1MP3E3 | 1MP3E3 | 1MP3E3 | 1MP3E3 | 1MP3E3 |
|  |  |  |  | Evidence | 4 | 4 | 4 | 4 | 4 |
|  |  |  |  | Focus | 2 | 2 | 2 | 2 | 2 |
|  |  |  |  | Polarity | 7 | 7 | 7 | 7 | 7 |
| 229 | 7226 | 10075737\_178 | Indeed, the kinetics of calpain activity paralleled the initiation and progressive loss of membrane integrity (Figs. 3 and 5). |A1:\*\*1SP3E3| |A2:\*\*1SP3E3-| |A3:\*\*1SP2E3| |A4:\*\*1SP3E3-| |A5:\*\*1SP3E3+| |  |  |  |  |  |  |
|  |  |  |  | Annotation | 1SP3E3 | 1SP3E3- | 1SP2E3 | 1SP3E3- | 1SP3E3+ |
|  |  |  |  | Evidence | 4 | 4 | 4 | 4 | 4 |
|  |  |  |  | Focus | 4 | 4 | 4 | 4 | 4 |
|  |  |  |  | Polarity | 7 | 7 | 6 | 7 | 7 |
| 230 | 658 | 12204687\_25 | HRP-2 has been shown to bind multiple molecules of heme and to mediate heme polymerization in vitro [10, 11]. |A1:\*\*1SP3E2| |A2:\*\*1SP3E2| |A3:\*\*1SP3E2| |A4:\*\*1SP3E2| |A5:\*\*1SP3E2+| |  |  |  |  |  |  |
|  |  |  |  | Annotation | 1SP3E2 | 1SP3E2 | 1SP3E2 | 1SP3E2 | 1SP3E2+ |
|  |  |  |  | Evidence | 3 | 3 | 3 | 3 | 3 |
|  |  |  |  | Focus | 4 | 4 | 4 | 4 | 4 |
|  |  |  |  | Polarity | 7 | 7 | 7 | 7 | 7 |
| 231 | 8691 | 8621600\_27 | Additional experiments are required to assess |A5:\*\*1SP3E1| whether this region contains phosphorylation sites involved in receptor uncoupling and to identify the putative kinase participating in such regulation. |A5:\*\*2SP1E1| |A1:\*\*1SP3E3| |A2:\*\*1SP1E1| |A3:\*\*1SP3E0| |A4:\*\*1SP3E1| |  |  |  |  |  |  |
|  |  |  |  | Annotation | 1SP3E3 1SP3E3 | 1SP1E1 1SP1E1 | 1SP3E0 1SP3E0 | 1SP3E1 1SP3E1 | 1SP3E1 2SP1E1 |
|  |  |  |  | Evidence | 4 4 | 2 2 | 1 1 | 2 2 | 2 2 |
|  |  |  |  | Focus | 4 4 | 4 4 | 4 4 | 4 4 | 4 4 |
|  |  |  |  | Polarity | 7 7 | 5 5 | 7 7 | 7 7 | 7 5 |
| 232 | 5780 | 11861568\_153 | Comparison of standardized intervals reveals variable recombination along the male chromosomes. |A1:\*\*1GP3E0| |A2:\*\*1SP3E0| |A3:\*\*1SP3E3| |A4:\*\*1SP3E0| |A5:\*\*1SP3E3| |  |  |  |  |  |  |
|  |  |  |  | Annotation | 1GP3E0 | 1SP3E0 | 1SP3E3 | 1SP3E0 | 1SP3E3 |
|  |  |  |  | Evidence | 1 | 1 | 4 | 1 | 4 |
|  |  |  |  | Focus | 1 | 4 | 4 | 4 | 4 |
|  |  |  |  | Polarity | 7 | 7 | 7 | 7 | 7 |
| 233 | 9837 | 11526156\_127 | SEM images of mature (48-h) C. dubliniensis NCPF 3949 biofilms formed on PMMA. |A1:\*\*1GP3E3| |A2:\*\*1GP3E3| |A3:\*\*1SP3E3| |A4:\*\*1SP3E3| |A5:\*\*1MP3E3| |  |  |  |  |  |  |
|  |  |  |  | Annotation | 1GP3E3 | 1GP3E3 | 1SP3E3 | 1SP3E3 | 1MP3E3 |
|  |  |  |  | Evidence | 4 | 4 | 4 | 4 | 4 |
|  |  |  |  | Focus | 1 | 1 | 4 | 4 | 2 |
|  |  |  |  | Polarity | 7 | 7 | 7 | 7 | 7 |
| 234 | 9005 | 11923998\_4 | Caregivers are encouraged to answer infant cries swiftly, consistently, and comprehensively. |A1:\*\*1GP3E3| |A2:\*\*1GP3E0| |A3:\*\*1GP3E0| |A4:\*\*1GP3E0| |A5:\*\*1GP3E3| |  |  |  |  |  |  |
|  |  |  |  | Annotation | 1GP3E3 | 1GP3E0 | 1GP3E0 | 1GP3E0 | 1GP3E3 |
|  |  |  |  | Evidence | 4 | 1 | 1 | 1 | 4 |
|  |  |  |  | Focus | 1 | 1 | 1 | 1 | 1 |
|  |  |  |  | Polarity | 7 | 7 | 7 | 7 | 7 |
| 235 | 6424 | 9390512\_193 | The T102C polymorphism does not change the amino acid sequence of the protein, |A1:\*\*1SN3E0| |A2:\*\*1SN3E0| |A3:\*\*1SN3E0| |A4:\*\*1SN3E0| |A5:\*\*1GN3E2| although it is possible that it could affect the secondary structure and stability of the mRNA (Arranz et al. 1995). |A1:\*\*2SP1E2| |A2:\*\*1SP2E2| |A3:\*\*2SP2E2| |A4:\*\*2SP2E2| |A5:\*\*2GP2E2| |  |  |  |  |  |  |
|  |  |  |  | Annotation | 1SN3E0 2SP1E2 | 1SN3E0 1SP2E2 | 1SN3E0 2SP2E2 | 1SN3E0 2SP2E2 | 1GN3E2 2GP2E2 |
|  |  |  |  | Evidence | 1 3 | 1 3 | 1 3 | 1 3 | 3 3 |
|  |  |  |  | Focus | 4 4 | 4 4 | 4 4 | 4 4 | 1 1 |
|  |  |  |  | Polarity | 1 5 | 1 6 | 1 6 | 1 6 | 1 6 |
| 236 | 1614 | 10988293\_12 | Furthermore, the general CaMK inhibitor, KN-93, |A5:\*\*1GP3E2| blocks cell proliferation before S phase in both cycling cells |A3:\*\*1SP3E0-| and those stimulated to enter the cell cycle from G0 ( ). |A3:\*\*2SP3E0| |A1:\*\*1SN3E2| |A2:\*\*1SP3E2-| |A4:\*\*1SP3E0| |A5:\*\*2SP3E2-| |  |  |  |  |  |  |
|  |  |  |  | Annotation | 1SN3E2 1SN3E2 1SN3E2 | 1SP3E2- 1SP3E2- 1SP3E2- | 1SP3E0- 1SP3E0- 2SP3E0 | 1SP3E0 1SP3E0 1SP3E0 | 1GP3E2 2SP3E2- 2SP3E2- |
|  |  |  |  | Evidence | 3 3 3 | 3 3 3 | 1 1 1 | 1 1 1 | 3 3 3 |
|  |  |  |  | Focus | 4 4 4 | 4 4 4 | 4 4 4 | 4 4 4 | 1 4 4 |
|  |  |  |  | Polarity | 1 1 1 | 7 7 7 | 7 7 7 | 7 7 7 | 7 7 7 |
| 237 | 9517 | 12163473\_174 | Additionally, some proteins involved in motility and adhesion are substrates for both calpains and caspases (e.g., FAK, actin, and cortactin), |A2:\*\*1SP3E0| and some of these proteins are also substrates of Src (for review see Tatosyan and Mizenina, 2000), |A1:\*\*1SP3E23| whose phosphorylation is modulated by E4orf4 expression (positively and negatively for cortactin and FAK, respectively) ( Lavoie et al., 2000). |A1:\*\*2SP3E23| |A2:\*\*1SP3E2| |A3:\*\*1SP3E2| |A4:\*\*1SP3E2| |A5:\*\*1SP3E2| |  |  |  |  |  |  |
|  |  |  |  | Annotation | 1SP3E23 1SP3E23 2SP3E23 | 1SP3E0 1SP3E2 1SP3E2 | 1SP3E2 1SP3E2 1SP3E2 | 1SP3E2 1SP3E2 1SP3E2 | 1SP3E2 1SP3E2 1SP3E2 |
|  |  |  |  | Evidence | 3 3 3 | 1 3 3 | 3 3 3 | 3 3 3 | 3 3 3 |
|  |  |  |  | Focus | 4 4 4 | 4 4 4 | 4 4 4 | 4 4 4 | 4 4 4 |
|  |  |  |  | Polarity | 7 7 7 | 7 7 7 | 7 7 7 | 7 7 7 | 7 7 7 |
| 238 | 2691 | 10618205\_98 | Cells were fixed with 1% (vol/vol) glutaraldehyde, stained with 0.05% acridine orange (vol/vol), and then filtrated through polycarbonate filters (Nuclepore Track-Etch membrane; 0.2-mum pore size; Corning Costar, Boston, Mass.). |A1:\*\*1MP3E3| |A2:\*\*1MP3E3| |A3:\*\*1MP3E3| |A4:\*\*1MP3E3| |A5:\*\*1MP3E3| |  |  |  |  |  |  |
|  |  |  |  | Annotation | 1MP3E3 | 1MP3E3 | 1MP3E3 | 1MP3E3 | 1MP3E3 |
|  |  |  |  | Evidence | 4 | 4 | 4 | 4 | 4 |
|  |  |  |  | Focus | 2 | 2 | 2 | 2 | 2 |
|  |  |  |  | Polarity | 7 | 7 | 7 | 7 | 7 |
| 239 | 9315 | 10197540\_118 | Periods of acoustic stimulation of 8 volumes each alternated with rest conditions of the same duration (Figure 2, bottom). |A1:\*\*1GP3E3| |A2:\*\*1GP3E3| |A3:\*\*1SP3E3| |A4:\*\*1MP3E3| |A5:\*\*1MP3E3| |  |  |  |  |  |  |
|  |  |  |  | Annotation | 1GP3E3 | 1GP3E3 | 1SP3E3 | 1MP3E3 | 1MP3E3 |
|  |  |  |  | Evidence | 4 | 4 | 4 | 4 | 4 |
|  |  |  |  | Focus | 1 | 1 | 4 | 2 | 2 |
|  |  |  |  | Polarity | 7 | 7 | 7 | 7 | 7 |
| 240 | 9896 | 10454358\_192 | In mutants, we found only a slight initial depression of EPSCs |A1:\*\*1SN3E3| and a single LTPAMPA, at 156.7 plus-or-minus 15.6% with no rebound of NMDA receptor-mediated EPSCs (105 plus-or-minus 3.3%) following 60 muM ACPD (Fig. 5b; 25 neurons, 13 mice; P < 0.01). |A1:\*\*2SP3E3| |A2:\*\*1SP3E3| |A3:\*\*1SP3E3| |A4:\*\*1MP3E3| |A5:\*\*1MP3E3| |  |  |  |  |  |  |
|  |  |  |  | Annotation | 1SN3E3 2SP3E3 | 1SP3E3 1SP3E3 | 1SP3E3 1SP3E3 | 1MP3E3 1MP3E3 | 1MP3E3 1MP3E3 |
|  |  |  |  | Evidence | 4 4 | 4 4 | 4 4 | 4 4 | 4 4 |
|  |  |  |  | Focus | 4 4 | 4 4 | 4 4 | 2 2 | 2 2 |
|  |  |  |  | Polarity | 1 7 | 7 7 | 7 7 | 7 7 | 7 7 |
| 241 | 4158 | 9727068\_82 | Membranes were blocked overnight at 4 degrees C in 5% dry milk. |A1:\*\*1SN3E3| |A2:\*\*1SN3E3| |A3:\*\*1MP3E3| |A4:\*\*1GP3E0| |A5:\*\*1MP3E3| |  |  |  |  |  |  |
|  |  |  |  | Annotation | 1SN3E3 | 1SN3E3 | 1MP3E3 | 1GP3E0 | 1MP3E3 |
|  |  |  |  | Evidence | 4 | 4 | 4 | 1 | 4 |
|  |  |  |  | Focus | 4 | 4 | 2 | 1 | 2 |
|  |  |  |  | Polarity | 1 | 1 | 7 | 7 | 7 |
| 242 | 3602 | 11063675\_279 | HAARER, B. K., A. CORBETT, Y. KWEON, A. S. PETZOLD, and P. SILVER et al., 1996 SEC3 mutations are synthetically lethal with profilin mutations and cause defects in diploid-specific bud-site selection. |A1:\*\*1SP3E3| |A2:\*\*1SP3E3| |A3:\*\*1SP3E0| |A4:\*\*1SP3E3| |A5:\*\*1SP3E2| |  |  |  |  |  |  |
|  |  |  |  | Annotation | 1SP3E3 | 1SP3E3 | 1SP3E0 | 1SP3E3 | 1SP3E2 |
|  |  |  |  | Evidence | 4 | 4 | 1 | 4 | 3 |
|  |  |  |  | Focus | 4 | 4 | 4 | 4 | 4 |
|  |  |  |  | Polarity | 7 | 7 | 7 | 7 | 7 |
| 243 | 483 | 11713265\_276 | The cells were incubated for an additional 1 h to allow all conjugated dextran to be taken up into lysosomes (21). |A1:\*\*1SP3E23| |A2:\*\*1SP3E23| |A3:\*\*1MP3E2| |A4:\*\*1MP3E2| |A5:\*\*1MP3E2| |  |  |  |  |  |  |
|  |  |  |  | Annotation | 1SP3E23 | 1SP3E23 | 1MP3E2 | 1MP3E2 | 1MP3E2 |
|  |  |  |  | Evidence | 3 | 3 | 3 | 3 | 3 |
|  |  |  |  | Focus | 4 | 4 | 2 | 2 | 2 |
|  |  |  |  | Polarity | 7 | 7 | 7 | 7 | 7 |
| 244 | 1973 | 11509657\_92 | For technical reasons, GFP-c-CRD ( Mr, 36,000) was used for mass spectrometry (MS), |A1:\*\*1MP3E3| and deltaGFP-c-CRD ( Mr, 14,400), in which most of the GFP region was deleted, was used for peptide analysis and thioredoxin-dependent reduction experiments. |A1:\*\*2MP3E3| |A2:\*\*1MP3E3| |A3:\*\*1MSP3E3| |A4:\*\*1MP3E3| |A5:\*\*1MP3E3| |  |  |  |  |  |  |
|  |  |  |  | Annotation | 1MP3E3 2MP3E3 | 1MP3E3 1MP3E3 | 1MSP3E3 1MSP3E3 | 1MP3E3 1MP3E3 | 1MP3E3 1MP3E3 |
|  |  |  |  | Evidence | 4 4 | 4 4 | 4 4 | 4 4 | 4 4 |
|  |  |  |  | Focus | 2 2 | 2 2 | 6 6 | 2 2 | 2 2 |
|  |  |  |  | Polarity | 7 7 | 7 7 | 7 7 | 7 7 | 7 7 |
| 245 | 1053 | 12957289\_168 | Conditioned media were concentrated by TCA precipitation, normalized for protein concentration, and immunoblotted with an anti-VEGF antibody. |A1:\*\*1MP3E3| |A2:\*\*1MP3E3| |A3:\*\*1MP3E3| |A4:\*\*1MP3E3| |A5:\*\*1MP3E3| |  |  |  |  |  |  |
|  |  |  |  | Annotation | 1MP3E3 | 1MP3E3 | 1MP3E3 | 1MP3E3 | 1MP3E3 |
|  |  |  |  | Evidence | 4 | 4 | 4 | 4 | 4 |
|  |  |  |  | Focus | 2 | 2 | 2 | 2 | 2 |
|  |  |  |  | Polarity | 7 | 7 | 7 | 7 | 7 |
| 246 | 8980 | 12551960\_21 | This facilitates the correlation of cellular and molecular events with the behavioral change, as well as targeted interventions in identified brain areas at discrete phases of the use-dependent behavioral change. |A1:\*\*1GP3E0| |A2:\*\*1SP2E0| |A3:\*\*1SP3E0| |A4:\*\*1SP3E0| |A5:\*\*1GP3E0| |  |  |  |  |  |  |
|  |  |  |  | Annotation | 1GP3E0 | 1SP2E0 | 1SP3E0 | 1SP3E0 | 1GP3E0 |
|  |  |  |  | Evidence | 1 | 1 | 1 | 1 | 1 |
|  |  |  |  | Focus | 1 | 4 | 4 | 4 | 1 |
|  |  |  |  | Polarity | 7 | 6 | 7 | 7 | 7 |
| 247 | 3160 | 11687499\_101 | Among these are a Wnt homologue and a homologue of Mex-3, which is required for anterior-posterior patterning in C. elegans; |A4:\*\*1SP3E0| both, therefore, are good candidates for mRNAs involved in axis formation ( Sasakura et al. 1998a, Satou 1999). |A1:\*\*1SP3E2| |A2:\*\*1SP3E2| |A3:\*\*1SP3E2| |A4:\*\*2SP3E2| |A5:\*\*1SP3E2| |  |  |  |  |  |  |
|  |  |  |  | Annotation | 1SP3E2 1SP3E2 | 1SP3E2 1SP3E2 | 1SP3E2 1SP3E2 | 1SP3E0 2SP3E2 | 1SP3E2 1SP3E2 |
|  |  |  |  | Evidence | 3 3 | 3 3 | 3 3 | 1 3 | 3 3 |
|  |  |  |  | Focus | 4 4 | 4 4 | 4 4 | 4 4 | 4 4 |
|  |  |  |  | Polarity | 7 7 | 7 7 | 7 7 | 7 7 | 7 7 |
| 248 | 7648 | 11930903\_15 | In addition to inhibition of prostaglandin synthesis, |A2:\*\*1SP3E0-| |A5:\*\*1SP3E0-| damage resulted in an increase of cyclooxygenase-2 protein expression. |A2:\*\*2SP3E3+| |A1:\*\*1SN3E3+| |A3:\*\*1SP3E0| |A4:\*\*1SN3E0+| |A5:\*\*2SP3E0+| |  |  |  |  |  |  |
|  |  |  |  | Annotation | 1SN3E3+ 1SN3E3+ | 1SP3E0- 2SP3E3+ | 1SP3E0 1SP3E0 | 1SN3E0+ 1SN3E0+ | 1SP3E0- 2SP3E0+ |
|  |  |  |  | Evidence | 4 4 | 1 4 | 1 1 | 1 1 | 1 1 |
|  |  |  |  | Focus | 4 4 | 4 4 | 4 4 | 4 4 | 4 4 |
|  |  |  |  | Polarity | 1 1 | 7 7 | 7 7 | 1 1 | 7 7 |
| 249 | 5279 | 8789948\_269 | For example, the current response of the heteromeric 2/ 1 NMDA receptor channel is more than two orders of magnitude largerR than that of the homomeric 1 channel in a Xenopus oocyte expression system. |A1:\*\*1SP3E0| |A2:\*\*1SP3E1| |A3:\*\*1SP3E0| |A4:\*\*1SP3E0| |A5:\*\*1SP3E1| |  |  |  |  |  |  |
|  |  |  |  | Annotation | 1SP3E0 | 1SP3E1 | 1SP3E0 | 1SP3E0 | 1SP3E1 |
|  |  |  |  | Evidence | 1 | 2 | 1 | 1 | 2 |
|  |  |  |  | Focus | 4 | 4 | 4 | 4 | 4 |
|  |  |  |  | Polarity | 7 | 7 | 7 | 7 | 7 |
| 250 | 8423 | 9560277\_113 | To determine whether the observed decrease in cartridge density reflected a generalized decrease in GAT-1 immunoreactivity in all GABA terminals, |A2:\*\*1SP2E3-| |A3:\*\*1SP2E0| |A5:\*\*1SP3E0-| two measures were made of all GAT-1-labeled boutons. |A2:\*\*2SP3E3| |A1:\*\*1SP2E3| |A3:\*\*2SP3E3| |A4:\*\*1SP3E1-| |A5:\*\*2SP3E3| |  |  |  |  |  |  |
|  |  |  |  | Annotation | 1SP2E3 1SP2E3 | 1SP2E3- 2SP3E3 | 1SP2E0 2SP3E3 | 1SP3E1- 1SP3E1- | 1SP3E0- 2SP3E3 |
|  |  |  |  | Evidence | 4 4 | 4 4 | 1 4 | 2 2 | 1 4 |
|  |  |  |  | Focus | 4 4 | 4 4 | 4 4 | 4 4 | 4 4 |
|  |  |  |  | Polarity | 6 6 | 6 7 | 6 7 | 7 7 | 7 7 |
| 251 | 3382 | 11306348\_198 | Consensus sequences derived from peptides selected by mutant WW domains. |A1:\*\*1SP3E3| |A2:\*\*1SP3E3| |A3:\*\*1SP3E0| |A4:\*\*1SP3E0| |A5:\*\*1SP3E0| |  |  |  |  |  |  |
|  |  |  |  | Annotation | 1SP3E3 | 1SP3E3 | 1SP3E0 | 1SP3E0 | 1SP3E0 |
|  |  |  |  | Evidence | 4 | 4 | 1 | 1 | 1 |
|  |  |  |  | Focus | 4 | 4 | 4 | 4 | 4 |
|  |  |  |  | Polarity | 7 | 7 | 7 | 7 | 7 |
| 252 | 6048 | 11533250\_309 | Wnt signaling plays a critical role in carcinogenesis (48). |A1:\*\*1SP3E2| |A2:\*\*1SP3E2| |A3:\*\*1SP3E0| |A4:\*\*1SP3E2| |A5:\*\*1SP3E2| |  |  |  |  |  |  |
|  |  |  |  | Annotation | 1SP3E2 | 1SP3E2 | 1SP3E0 | 1SP3E2 | 1SP3E2 |
|  |  |  |  | Evidence | 3 | 3 | 1 | 3 | 3 |
|  |  |  |  | Focus | 4 | 4 | 4 | 4 | 4 |
|  |  |  |  | Polarity | 7 | 7 | 7 | 7 | 7 |
| 253 | 4511 | 11779820\_94 | The outcomes suggest that breaks within tracts lead to intramolecular recombination (or possibly sister chromatid conversion without exchange), giving rise to expansions and contractions, |A3:\*\*1SP2E3| |A5:\*\*1SP2E1| and that breaks with no remnant of repeat units are invasive into the homolog and lead to conversion. |A3:\*\*2SN3E0| |A1:\*\*1GP3E0| |A2:\*\*1SP3E1| |A4:\*\*1SP3E1| |A5:\*\*2SN3E1| |  |  |  |  |  |  |
|  |  |  |  | Annotation | 1GP3E0 1GP3E0 | 1SP3E1 1SP3E1 | 1SP2E3 2SN3E0 | 1SP3E1 1SP3E1 | 1SP2E1 2SN3E1 |
|  |  |  |  | Evidence | 1 1 | 2 2 | 4 1 | 2 2 | 2 2 |
|  |  |  |  | Focus | 1 1 | 4 4 | 4 4 | 4 4 | 4 4 |
|  |  |  |  | Polarity | 7 7 | 7 7 | 6 1 | 7 7 | 6 1 |
| 254 | 9431 | 12135932\_7 | The multiple spinal cords remain but are enclosed in a single spinal column as in the human diastematomyelia. |A1:\*\*1GP3E0| |A2:\*\*1SP3E0| |A3:\*\*1SP3E0| |A4:\*\*1SP3E0| |A5:\*\*1SP3E1| |  |  |  |  |  |  |
|  |  |  |  | Annotation | 1GP3E0 | 1SP3E0 | 1SP3E0 | 1SP3E0 | 1SP3E1 |
|  |  |  |  | Evidence | 1 | 1 | 1 | 1 | 2 |
|  |  |  |  | Focus | 1 | 4 | 4 | 4 | 4 |
|  |  |  |  | Polarity | 7 | 7 | 7 | 7 | 7 |
| 255 | 9696 | 9560277\_125 | In the one study that did report a decrease in the density of small presumably nonpyramidal neurons in PFC layer 2 (31), |A4:\*\*1SP1E2| the decrease appeared to be associated with the presence of affective traits ( 32). |A4:\*\*2SP1E2-| |A1:\*\*1GP3E2-| |A2:\*\*1SP3E2-| |A3:\*\*1SP2E2| |A5:\*\*1SP3E2-| |  |  |  |  |  |  |
|  |  |  |  | Annotation | 1GP3E2- 1GP3E2- | 1SP3E2- 1SP3E2- | 1SP2E2 1SP2E2 | 1SP1E2 2SP1E2- | 1SP3E2- 1SP3E2- |
|  |  |  |  | Evidence | 3 3 | 3 3 | 3 3 | 3 3 | 3 3 |
|  |  |  |  | Focus | 1 1 | 4 4 | 4 4 | 4 4 | 4 4 |
|  |  |  |  | Polarity | 7 7 | 7 7 | 6 6 | 5 5 | 7 7 |
| 256 | 4029 | 9268348\_7 | The conventional names are listed together with the new systematic names in Table I. |A1:\*\*1GP3E3| |A2:\*\*1GP3E3| |A4:\*\*1GP3E2| Avidity, specificity, and MV-inhibitory property of anti-CD46 mAbs mAb |A5:\*\*1SP3E3| a Avidity for human CD46 |A5:\*\*2SP3E3+| b Binding to simian CD46 |A5:\*\*3SP3E3| c Inhibition of sH binding |A5:\*\*4SP3E3-| d nM I.1.-a (E4.3) |A5:\*\*5SP3E3| |A1:\*\*2SP0E0| |A2:\*\*2SP3E3-| |A3:\*\*1SP3E3| |A4:\*\*2SP3E0| |  |  |  |  |  |  |
|  |  |  |  | Annotation | 1GP3E3 2SP0E0 2SP0E0 2SP0E0 2SP0E0 2SP0E0 | 1GP3E3 2SP3E3- 2SP3E3- 2SP3E3- 2SP3E3- 2SP3E3- | 1SP3E3 1SP3E3 1SP3E3 1SP3E3 1SP3E3 1SP3E3 | 1GP3E2 2SP3E0 2SP3E0 2SP3E0 2SP3E0 2SP3E0 | 1SP3E3 1SP3E3 2SP3E3+ 3SP3E3 4SP3E3- 5SP3E3 |
|  |  |  |  | Evidence | 4 1 1 1 1 1 | 4 4 4 4 4 4 | 4 4 4 4 4 4 | 3 1 1 1 1 1 | 4 4 4 4 4 4 |
|  |  |  |  | Focus | 1 4 4 4 4 4 | 1 4 4 4 4 4 | 4 4 4 4 4 4 | 1 4 4 4 4 4 | 4 4 4 4 4 4 |
|  |  |  |  | Polarity | 7 4 4 4 4 4 | 7 7 7 7 7 7 | 7 7 7 7 7 7 | 7 7 7 7 7 7 | 7 7 7 7 7 7 |
| 257 | 1636 | 8616896\_167 | (A) RNA was derived from in vitro transcribed L - Vneodelta[X/Si DNA (wild type [wt]) or L - Vneo\*delta[X/Si DNA (stop codon [sc). |A1:\*\*1SP3E3| |A2:\*\*1SP3E3| |A3:\*\*1SP3E3| |A4:\*\*1MP3E3| |A5:\*\*1SP2E3| |  |  |  |  |  |  |
|  |  |  |  | Annotation | 1SP3E3 | 1SP3E3 | 1SP3E3 | 1MP3E3 | 1SP2E3 |
|  |  |  |  | Evidence | 4 | 4 | 4 | 4 | 4 |
|  |  |  |  | Focus | 4 | 4 | 4 | 2 | 4 |
|  |  |  |  | Polarity | 7 | 7 | 7 | 7 | 6 |
| 258 | 4595 | 9058589\_164 | In addition, the identification of genes already known |A5:\*\*1SP3E1| but not described to be regulated by phenobarbital |A5:\*\*2SN3E0| opens the possibility to study their regulation |A5:\*\*3SP2E0| in comparison to genes whose regulations have been studied for many years (e.g., cytochromes P450). |A5:\*\*4SP3E1| |A1:\*\*1SP2E3| |A2:\*\*1SP1E0| |A3:\*\*1SP2E0| |A4:\*\*1SN1E2| |  |  |  |  |  |  |
|  |  |  |  | Annotation | 1SP2E3 1SP2E3 1SP2E3 1SP2E3 | 1SP1E0 1SP1E0 1SP1E0 1SP1E0 | 1SP2E0 1SP2E0 1SP2E0 1SP2E0 | 1SN1E2 1SN1E2 1SN1E2 1SN1E2 | 1SP3E1 2SN3E0 3SP2E0 4SP3E1 |
|  |  |  |  | Evidence | 4 4 4 4 | 1 1 1 1 | 1 1 1 1 | 3 3 3 3 | 2 1 1 2 |
|  |  |  |  | Focus | 4 4 4 4 | 4 4 4 4 | 4 4 4 4 | 4 4 4 4 | 4 4 4 4 |
|  |  |  |  | Polarity | 6 6 6 6 | 5 5 5 5 | 6 6 6 6 | 3 3 3 3 | 7 1 6 7 |
| 259 | 2718 | 11358869\_32 | One FoxH1 homolog has been identified in mouse (also known as FAST2 or FoxH1a), as well as in human (FoxH1) and zebrafish ( schmalspur) (Labbe et al. 1998 Zhou ; et al. 1998 ; Pogoda et al. 2000 ; Sirotkin et al. 2000 ). |A1:\*\*1SP2E2| |A2:\*\*1SP3E2| |A3:\*\*1SP3E2| |A4:\*\*1SP3E2| |A5:\*\*1SP3E2| |  |  |  |  |  |  |
|  |  |  |  | Annotation | 1SP2E2 | 1SP3E2 | 1SP3E2 | 1SP3E2 | 1SP3E2 |
|  |  |  |  | Evidence | 3 | 3 | 3 | 3 | 3 |
|  |  |  |  | Focus | 4 | 4 | 4 | 4 | 4 |
|  |  |  |  | Polarity | 6 | 7 | 7 | 7 | 7 |
| 260 | 4477 | 10464322\_5 | Activation of Src kinase by the latter |A5:\*\*1GP3E0+| was also prevented by N2O3 scavenger homocysteine. |A5:\*\*2GP3E0-| |A1:\*\*1SN3E3| |A2:\*\*1SP3E3| |A3:\*\*1SP2E0| |A4:\*\*1SP3E0| |  |  |  |  |  |  |
|  |  |  |  | Annotation | 1SN3E3 1SN3E3 | 1SP3E3 1SP3E3 | 1SP2E0 1SP2E0 | 1SP3E0 1SP3E0 | 1GP3E0+ 2GP3E0- |
|  |  |  |  | Evidence | 4 4 | 4 4 | 1 1 | 1 1 | 1 1 |
|  |  |  |  | Focus | 4 4 | 4 4 | 4 4 | 4 4 | 1 1 |
|  |  |  |  | Polarity | 1 1 | 7 7 | 6 6 | 7 7 | 7 7 |
| 261 | 1740 | 10702487\_218 | The moderate numbers of S. aureus organisms in milk |A5:\*\*1GP3E0| could explain this phenomenon |A5:\*\*2GP2E0| by being below the threshold necessary |A5:\*\*3GP3E0-| for the triggering of an effective inflammatory response. |A5:\*\*4GP3E0+| |A1:\*\*1GP1E0| |A2:\*\*1SP2E0| |A3:\*\*1SP2E0| |A4:\*\*1SP3E3+| |  |  |  |  |  |  |
|  |  |  |  | Annotation | 1GP1E0 1GP1E0 1GP1E0 1GP1E0 | 1SP2E0 1SP2E0 1SP2E0 1SP2E0 | 1SP2E0 1SP2E0 1SP2E0 1SP2E0 | 1SP3E3+ 1SP3E3+ 1SP3E3+ 1SP3E3+ | 1GP3E0 2GP2E0 3GP3E0- 4GP3E0+ |
|  |  |  |  | Evidence | 1 1 1 1 | 1 1 1 1 | 1 1 1 1 | 4 4 4 4 | 1 1 1 1 |
|  |  |  |  | Focus | 1 1 1 1 | 4 4 4 4 | 4 4 4 4 | 4 4 4 4 | 1 1 1 1 |
|  |  |  |  | Polarity | 5 5 5 5 | 6 6 6 6 | 6 6 6 6 | 7 7 7 7 | 7 6 7 7 |
| 262 | 666 | 11713294\_284 | Likewise, inhibition of PtdIns 3-kinase |A5:\*\*1SP3E1-| or expression of dominant-negative Rac |A5:\*\*2SP3E1+| prevented F(ab'')2 anti-Ig-triggered BCR-F-actin association and receptor internalization, respectively. |A5:\*\*3SP3E1-| |A1:\*\*1SN3E3| |A2:\*\*1SP3E3| |A3:\*\*1SP3E0| |A4:\*\*1SN3E0| |  |  |  |  |  |  |
|  |  |  |  | Annotation | 1SN3E3 1SN3E3 1SN3E3 | 1SP3E3 1SP3E3 1SP3E3 | 1SP3E0 1SP3E0 1SP3E0 | 1SN3E0 1SN3E0 1SN3E0 | 1SP3E1- 2SP3E1+ 3SP3E1- |
|  |  |  |  | Evidence | 4 4 4 | 4 4 4 | 1 1 1 | 1 1 1 | 2 2 2 |
|  |  |  |  | Focus | 4 4 4 | 4 4 4 | 4 4 4 | 4 4 4 | 4 4 4 |
|  |  |  |  | Polarity | 1 1 1 | 7 7 7 | 7 7 7 | 1 1 1 | 7 7 7 |
| 263 | 7504 | 9539806\_37 | Animals were anesthetized with an injection of isotonic MgCl2 into the hemocoel and then were pinned ventral side up in a Sylgard-coated Petri dish. |A1:\*\*1GP3E3| |A2:\*\*1MP3E3| |A3:\*\*1SP3E3| |A4:\*\*1MP3E3| |A5:\*\*1SP3E3| |  |  |  |  |  |  |
|  |  |  |  | Annotation | 1GP3E3 | 1MP3E3 | 1SP3E3 | 1MP3E3 | 1SP3E3 |
|  |  |  |  | Evidence | 4 | 4 | 4 | 4 | 4 |
|  |  |  |  | Focus | 1 | 2 | 4 | 2 | 4 |
|  |  |  |  | Polarity | 7 | 7 | 7 | 7 | 7 |
| 264 | 7910 | 10369671\_207 | Contractile vacuoles are specialized organelles in protists living under hypo-osmotic conditions. |A1:\*\*1SP3E0| |A2:\*\*1SP3E0| |A3:\*\*1SP3E0| |A4:\*\*1SP3E0| |A5:\*\*1SP3E0| |  |  |  |  |  |  |
|  |  |  |  | Annotation | 1SP3E0 | 1SP3E0 | 1SP3E0 | 1SP3E0 | 1SP3E0 |
|  |  |  |  | Evidence | 1 | 1 | 1 | 1 | 1 |
|  |  |  |  | Focus | 4 | 4 | 4 | 4 | 4 |
|  |  |  |  | Polarity | 7 | 7 | 7 | 7 | 7 |
| 265 | 322 | 11781319\_68 | Numbers represent the corresponding constructs in B., untransfected cells; lane GFP, cells transfected with empty GFP vector. |A1:\*\*1SP3E0| |A2:\*\*1SP3E1| |A3:\*\*1SP3E3| |A4:\*\*1SP3E0| |A5:\*\*1MP3E3| |  |  |  |  |  |  |
|  |  |  |  | Annotation | 1SP3E0 | 1SP3E1 | 1SP3E3 | 1SP3E0 | 1MP3E3 |
|  |  |  |  | Evidence | 1 | 2 | 4 | 1 | 4 |
|  |  |  |  | Focus | 4 | 4 | 4 | 4 | 2 |
|  |  |  |  | Polarity | 7 | 7 | 7 | 7 | 7 |
| 266 | 3496 | 9333244\_13 | Furthermore, we find that cerberus is a potent inhibitor of Wnt signalling. |A1:\*\*1SP3E3| |A2:\*\*1SP3E3-| |A3:\*\*1SP2E3| |A4:\*\*1SP3E3| |A5:\*\*1SP3E1| |  |  |  |  |  |  |
|  |  |  |  | Annotation | 1SP3E3 | 1SP3E3- | 1SP2E3 | 1SP3E3 | 1SP3E1 |
|  |  |  |  | Evidence | 4 | 4 | 4 | 4 | 2 |
|  |  |  |  | Focus | 4 | 4 | 4 | 4 | 4 |
|  |  |  |  | Polarity | 7 | 7 | 6 | 7 | 7 |
| 267 | 2777 | 9753320\_196 | By e14.5 vascular endothelial cells seem to obliterate completely the optic cup of the Apaf1 mutant (Figure 3D). |A1:\*\*1SP2E3| |A2:\*\*1SP2E3| |A3:\*\*1SP2E3| |A4:\*\*1SP3E3| |A5:\*\*1SP3E3| |  |  |  |  |  |  |
|  |  |  |  | Annotation | 1SP2E3 | 1SP2E3 | 1SP2E3 | 1SP3E3 | 1SP3E3 |
|  |  |  |  | Evidence | 4 | 4 | 4 | 4 | 4 |
|  |  |  |  | Focus | 4 | 4 | 4 | 4 | 4 |
|  |  |  |  | Polarity | 6 | 6 | 6 | 7 | 7 |
| 268 | 4810 | 9560277\_107 | Although 13 of the schizophrenic subjects were receiving antipsychotic medications at the time of death, |A5:\*\*1GP3E1| several lines of evidence suggest that |A5:\*\*2GP2E1| this treatment did not account for the decreased cartridge density. |A5:\*\*3GN3E1-| |A1:\*\*1GP3E3-| |A2:\*\*1SN3E3-| |A3:\*\*1SN3E3| |A4:\*\*1SN2E3| |  |  |  |  |  |  |
|  |  |  |  | Annotation | 1GP3E3- 1GP3E3- 1GP3E3- | 1SN3E3- 1SN3E3- 1SN3E3- | 1SN3E3 1SN3E3 1SN3E3 | 1SN2E3 1SN2E3 1SN2E3 | 1GP3E1 2GP2E1 3GN3E1- |
|  |  |  |  | Evidence | 4 4 4 | 4 4 4 | 4 4 4 | 4 4 4 | 2 2 2 |
|  |  |  |  | Focus | 1 1 1 | 4 4 4 | 4 4 4 | 4 4 4 | 1 1 1 |
|  |  |  |  | Polarity | 7 7 7 | 1 1 1 | 1 1 1 | 2 2 2 | 7 6 1 |
| 269 | 2463 | 12419232\_35 | We reasoned that it might be possible to mimic this evolutionary history |A5:\*\*1GP2E3| by fusing the DNA binding domains of two highly divergent LAGLIDADG homing endonucleases to generate a catalytically active chimeric endonuclease. |A5:\*\*2SP3E3| |A1:\*\*1SP1E3| |A2:\*\*1SP1E1| |A3:\*\*1SP1E0| |A4:\*\*1SP2E3+| |  |  |  |  |  |  |
|  |  |  |  | Annotation | 1SP1E3 1SP1E3 | 1SP1E1 1SP1E1 | 1SP1E0 1SP1E0 | 1SP2E3+ 1SP2E3+ | 1GP2E3 2SP3E3 |
|  |  |  |  | Evidence | 4 4 | 2 2 | 1 1 | 4 4 | 4 4 |
|  |  |  |  | Focus | 4 4 | 4 4 | 4 4 | 4 4 | 1 4 |
|  |  |  |  | Polarity | 5 5 | 5 5 | 5 5 | 6 6 | 6 7 |
| 270 | 932 | 9463478\_64 | DNA topoisomerase II cleavage was carried out in a volume of 15 mul at 28 degrees for 6 min. |A1:\*\*1MP3E3| |A2:\*\*1MP3E3| |A3:\*\*1SP3E3| |A4:\*\*1MP3E3| |A5:\*\*1MP3E3| |  |  |  |  |  |  |
|  |  |  |  | Annotation | 1MP3E3 | 1MP3E3 | 1SP3E3 | 1MP3E3 | 1MP3E3 |
|  |  |  |  | Evidence | 4 | 4 | 4 | 4 | 4 |
|  |  |  |  | Focus | 2 | 2 | 4 | 2 | 2 |
|  |  |  |  | Polarity | 7 | 7 | 7 | 7 | 7 |
| 271 | 7244 | 9139704\_6 | Why are beta1 subunits not sulfated in other alphabeta1 heterodimers? |A1:\*\*1SN0E0| |A2:\*\*1SN3E0| |A3:\*\*1SN0E0| |A4:\*\*1SN3E0| |A5:\*\*1GN3E0| |  |  |  |  |  |  |
|  |  |  |  | Annotation | 1SN0E0 | 1SN3E0 | 1SN0E0 | 1SN3E0 | 1GN3E0 |
|  |  |  |  | Evidence | 1 | 1 | 1 | 1 | 1 |
|  |  |  |  | Focus | 4 | 4 | 4 | 4 | 1 |
|  |  |  |  | Polarity | 4 | 1 | 4 | 1 | 1 |
| 272 | 796 | 10200329\_25 | Both issues can be effectively examined by testing first-degree relatives of schizophrenic patients. |A1:\*\*1GP1E3| |A2:\*\*1SP2E0| |A3:\*\*1SP2E0| |A4:\*\*1GP3E0| |A5:\*\*1GP3E0| |  |  |  |  |  |  |
|  |  |  |  | Annotation | 1GP1E3 | 1SP2E0 | 1SP2E0 | 1GP3E0 | 1GP3E0 |
|  |  |  |  | Evidence | 4 | 1 | 1 | 1 | 1 |
|  |  |  |  | Focus | 1 | 4 | 4 | 1 | 1 |
|  |  |  |  | Polarity | 5 | 6 | 6 | 7 | 7 |
| 273 | 2785 | 11238885\_8 | Since Dach1 mutants die shortly after birth, |A1:\*\*1SP3E0| |A5:\*\*1GP3E3| it remains possible that Dach1 is required for postnatal development of these structures. |A1:\*\*2SP3E3| |A2:\*\*1SP2E0| |A3:\*\*1SP1E0| |A4:\*\*1SP2E0| |A5:\*\*2GP2E3| |  |  |  |  |  |  |
|  |  |  |  | Annotation | 1SP3E0 2SP3E3 | 1SP2E0 1SP2E0 | 1SP1E0 1SP1E0 | 1SP2E0 1SP2E0 | 1GP3E3 2GP2E3 |
|  |  |  |  | Evidence | 1 4 | 1 1 | 1 1 | 1 1 | 4 4 |
|  |  |  |  | Focus | 4 4 | 4 4 | 4 4 | 4 4 | 1 1 |
|  |  |  |  | Polarity | 7 7 | 6 6 | 5 5 | 6 6 | 7 6 |
| 274 | 1118 | 12018456\_5 | RESULTS: There were no prolonged wound complications, |A1:\*\*1GN3E3| |A2:\*\*1SN3E3| |A3:\*\*1SN3E3| |A5:\*\*1GN3E3| and only one positive microscopic margin was detected. |A1:\*\*2GP3E3| |A2:\*\*1SP3E3| |A3:\*\*2SP1E3| |A4:\*\*1SP3E3| |A5:\*\*2GP3E3| |  |  |  |  |  |  |
|  |  |  |  | Annotation | 1GN3E3 2GP3E3 | 1SN3E3 1SP3E3 | 1SN3E3 2SP1E3 | 1SP3E3 1SP3E3 | 1GN3E3 2GP3E3 |
|  |  |  |  | Evidence | 4 4 | 4 4 | 4 4 | 4 4 | 4 4 |
|  |  |  |  | Focus | 1 1 | 4 4 | 4 4 | 4 4 | 1 1 |
|  |  |  |  | Polarity | 1 7 | 1 7 | 1 5 | 7 7 | 1 7 |
| 275 | 9904 | 9271115\_212 | If both LIG4 and Ku are involved in illegitimate DNA end-joining, one possible explanation of the apparent discrepancies in their mutant phenotypes could be that |A5:\*\*1GP2E0| Ku proteins have a nonspecific DNA end protection role, |A1:\*\*1SP1E0| |A3:\*\*1SP1E0| |A4:\*\*1SN2E0| whereas LIG4 might recognize a much more limited range of DNA substrates; |A5:\*\*2SP3E0| |A1:\*\*2SP3E0| if such specific DNA end arrangements are only rarely generated in consequence to DNA-damaging treatment with UV, MMS, or ionizing radiation, |A5:\*\*3MP3E3| it would be expected that the contribution of LIG4 to repair of such damage is unimportant. |A5:\*\*4GN2E0| |A1:\*\*3SP2E0| |A2:\*\*1SP1E1| |A3:\*\*2SP2E0| |A4:\*\*2SP2E0| |  |  |  |  |  |  |
|  |  |  |  | Annotation | 1SP1E0 1SP1E0 2SP3E0 3SP2E0 3SP2E0 | 1SP1E1 1SP1E1 1SP1E1 1SP1E1 1SP1E1 | 1SP1E0 1SP1E0 2SP2E0 2SP2E0 2SP2E0 | 1SN2E0 1SN2E0 2SP2E0 2SP2E0 2SP2E0 | 1GP2E0 2SP3E0 2SP3E0 3MP3E3 4GN2E0 |
|  |  |  |  | Evidence | 1 1 1 1 1 | 2 2 2 2 2 | 1 1 1 1 1 | 1 1 1 1 1 | 1 1 1 4 1 |
|  |  |  |  | Focus | 4 4 4 4 4 | 4 4 4 4 4 | 4 4 4 4 4 | 4 4 4 4 4 | 1 4 4 2 1 |
|  |  |  |  | Polarity | 5 5 7 6 6 | 5 5 5 5 5 | 5 5 6 6 6 | 2 2 6 6 6 | 6 7 7 7 2 |
| 276 | 4237 | 10329711\_65 | As the photo-cross-linking reaction with M. CviBIII was almost quantitative with respect to the duplex ODN, a crude reaction mixture of 32P-labeled TCGI/TCGA(S) and M. CviBIII was subjected to proteolytic fragmentation with chymotrypsin, and the time course of the protease reaction was analyzed by denaturing PAGE (Fig. 5). |A1:\*\*1MP3E3| |A2:\*\*1MP3E3| |A3:\*\*1SP3E3| |A4:\*\*1SP3E3| |A5:\*\*1MP3E3| |  |  |  |  |  |  |
|  |  |  |  | Annotation | 1MP3E3 | 1MP3E3 | 1SP3E3 | 1SP3E3 | 1MP3E3 |
|  |  |  |  | Evidence | 4 | 4 | 4 | 4 | 4 |
|  |  |  |  | Focus | 2 | 2 | 4 | 4 | 2 |
|  |  |  |  | Polarity | 7 | 7 | 7 | 7 | 7 |
| 277 | 1696 | 12464703\_24 | Similarly, concurrent activation of the putamen and supplementary motor area during administration of the pursuit rotor motor-skill learning test (Grafton et al. 1992, 1994 ), |A4:\*\*1SP3E2| |A1:\*\*1SP3E2| |A2:\*\*1SP3E2+| |A5:\*\*1SP3E2| would suggest that the motor circuit of the basal ganglia may be important during motor-skill learning. |A4:\*\*2SP2E1| |A1:\*\*2SP1E0| |A2:\*\*2SP2E1| |A3:\*\*1SP2E2| |A5:\*\*2SP2E2| |  |  |  |  |  |  |
|  |  |  |  | Annotation | 1SP3E2 2SP1E0 | 1SP3E2+ 2SP2E1 | 1SP2E2 1SP2E2 | 1SP3E2 2SP2E1 | 1SP3E2 2SP2E2 |
|  |  |  |  | Evidence | 3 1 | 3 2 | 3 3 | 3 2 | 3 3 |
|  |  |  |  | Focus | 4 4 | 4 4 | 4 4 | 4 4 | 4 4 |
|  |  |  |  | Polarity | 7 5 | 7 6 | 6 6 | 7 6 | 7 6 |
| 278 | 7222 | 11390642\_160 | Cellular lysates containing 150 mug of protein were either resolved directly by SDS-PAGE (lanes 1, 2, 5, and 6) or were treated with 5 U of calf intestinal phosphatase (CIP) in the absence (lane 3) or in the presence (+ INH) of phosphatase inhibitors, including 10 mM beta-glycerophosphate and 10 mM NaF (lane 4). |A1:\*\*1MP3E3| |A2:\*\*1MP3E3| |A3:\*\*1MP2E3| |A4:\*\*1SP3E3| |A5:\*\*1MP3E3| |  |  |  |  |  |  |
|  |  |  |  | Annotation | 1MP3E3 | 1MP3E3 | 1MP2E3 | 1SP3E3 | 1MP3E3 |
|  |  |  |  | Evidence | 4 | 4 | 4 | 4 | 4 |
|  |  |  |  | Focus | 2 | 2 | 2 | 4 | 2 |
|  |  |  |  | Polarity | 7 | 7 | 6 | 7 | 7 |
| 279 | 4435 | 11292757\_128 | Neutrophils treated with gram-negative and gram-positive sera showed DNA fragmentation in the form of a ladder pattern, |A5:\*\*1MP3E3| further confirming the presence of an apoptotic factor in the sera of infected patients (data not shown). |A5:\*\*2SP3E3| |A1:\*\*1SP3E1| |A2:\*\*1SP3E1| |A3:\*\*1MSP3E3| |A4:\*\*1SP3E3| |  |  |  |  |  |  |
|  |  |  |  | Annotation | 1SP3E1 1SP3E1 | 1SP3E1 1SP3E1 | 1MSP3E3 1MSP3E3 | 1SP3E3 1SP3E3 | 1MP3E3 2SP3E3 |
|  |  |  |  | Evidence | 2 2 | 2 2 | 4 4 | 4 4 | 4 4 |
|  |  |  |  | Focus | 4 4 | 4 4 | 6 6 | 4 4 | 2 4 |
|  |  |  |  | Polarity | 7 7 | 7 7 | 7 7 | 7 7 | 7 7 |
| 280 | 3662 | 12167350\_7 | We also used the basic reproduction ratio to investigate the evolution of parasite virulence in relation to the migration decisions of susceptible hosts. |A1:\*\*1GP3E3| |A2:\*\*1SP3E3| |A3:\*\*1SP3E3| |A4:\*\*1SP3E3| |A5:\*\*1MP3E3| |  |  |  |  |  |  |
|  |  |  |  | Annotation | 1GP3E3 | 1SP3E3 | 1SP3E3 | 1SP3E3 | 1MP3E3 |
|  |  |  |  | Evidence | 4 | 4 | 4 | 4 | 4 |
|  |  |  |  | Focus | 1 | 4 | 4 | 4 | 2 |
|  |  |  |  | Polarity | 7 | 7 | 7 | 7 | 7 |
| 281 | 9537 | 12270131\_15 | Other loci, intron 1, and codons 243, 325, and 594, did not show a difference between cancer patients and controls. |A1:\*\*1SN3E0| |A2:\*\*1SN3E0| |A3:\*\*1SP3E3| |A4:\*\*1SN3E0| |A5:\*\*1GN3E3| |  |  |  |  |  |  |
|  |  |  |  | Annotation | 1SN3E0 | 1SN3E0 | 1SP3E3 | 1SN3E0 | 1GN3E3 |
|  |  |  |  | Evidence | 1 | 1 | 4 | 1 | 4 |
|  |  |  |  | Focus | 4 | 4 | 4 | 4 | 1 |
|  |  |  |  | Polarity | 1 | 1 | 7 | 1 | 1 |
| 282 | 936 | 9111038\_5 | In a later paper, the same authors ( ) used the transfection of cells by annexin III cDNA to demonstrate that the phosphohydrolase activity was dependent on the expression of annexin III. |A1:\*\*1SP3E2| |A2:\*\*1SP3E2| |A3:\*\*1SP3E1| |A4:\*\*1SP3E3| |A5:\*\*1SMP3E2| |  |  |  |  |  |  |
|  |  |  |  | Annotation | 1SP3E2 | 1SP3E2 | 1SP3E1 | 1SP3E3 | 1SMP3E2 |
|  |  |  |  | Evidence | 3 | 3 | 2 | 4 | 3 |
|  |  |  |  | Focus | 4 | 4 | 4 | 4 | 6 |
|  |  |  |  | Polarity | 7 | 7 | 7 | 7 | 7 |
| 283 | 5650 | 9390512\_112 | This region was also first identified in the JHU/MIT (Pulver et al. 1995 ) genome scan. |A1:\*\*1SP3E23| |A2:\*\*1SP3E2| |A3:\*\*1MSP3E2| |A4:\*\*1SP3E2| |A5:\*\*1GP3E2| |  |  |  |  |  |  |
|  |  |  |  | Annotation | 1SP3E23 | 1SP3E2 | 1MSP3E2 | 1SP3E2 | 1GP3E2 |
|  |  |  |  | Evidence | 3 | 3 | 3 | 3 | 3 |
|  |  |  |  | Focus | 4 | 4 | 6 | 4 | 1 |
|  |  |  |  | Polarity | 7 | 7 | 7 | 7 | 7 |
| 284 | 7178 | 9673225\_25 | The precipitated proteins were removed by centrifugation and after being washed were resuspended in buffer containing 8 M urea, 0.5% (wt/vol) CHAPS {3-[(3-cholamidopropyl)-dimethyl ammonio-1-propanesulfonate}, and 5% (vol/vol) glycerol. |A1:\*\*1MP3E3| |A2:\*\*1MP3E3| |A3:\*\*1MP3E3| |A4:\*\*1MP3E3| |A5:\*\*1MP3E3| |  |  |  |  |  |  |
|  |  |  |  | Annotation | 1MP3E3 | 1MP3E3 | 1MP3E3 | 1MP3E3 | 1MP3E3 |
|  |  |  |  | Evidence | 4 | 4 | 4 | 4 | 4 |
|  |  |  |  | Focus | 2 | 2 | 2 | 2 | 2 |
|  |  |  |  | Polarity | 7 | 7 | 7 | 7 | 7 |
| 285 | 7106 | 9827799\_244 | At 3 days postinoculation TEV-GFP foci were visualized using a long-wavelength UV light (Blak-Ray Long Wave Length Ultraviolet Lamp, Model B 100 AP). |A1:\*\*1SP3E3| |A2:\*\*1SP3E3| |A3:\*\*1SP3E3| |A4:\*\*1SP3E3| |A5:\*\*1MP3E3| |  |  |  |  |  |  |
|  |  |  |  | Annotation | 1SP3E3 | 1SP3E3 | 1SP3E3 | 1SP3E3 | 1MP3E3 |
|  |  |  |  | Evidence | 4 | 4 | 4 | 4 | 4 |
|  |  |  |  | Focus | 4 | 4 | 4 | 4 | 2 |
|  |  |  |  | Polarity | 7 | 7 | 7 | 7 | 7 |
| 286 | 8350 | 12364586\_19 | Identification of schizophrenia genes is particularly challenging, |A5:\*\*1GP3E0| because the disease may encompass several entities that have not yet been defined, |A5:\*\*2GN2E0| limiting the accuracy of schizophrenia diagnosis as a phenotypic definition. |A5:\*\*3GP3E0| |A1:\*\*1GP1E0| |A2:\*\*1SN2E1| |A3:\*\*1SN1E0| |A4:\*\*1SN1E0| |  |  |  |  |  |  |
|  |  |  |  | Annotation | 1GP1E0 1GP1E0 1GP1E0 | 1SN2E1 1SN2E1 1SN2E1 | 1SN1E0 1SN1E0 1SN1E0 | 1SN1E0 1SN1E0 1SN1E0 | 1GP3E0 2GN2E0 3GP3E0 |
|  |  |  |  | Evidence | 1 1 1 | 2 2 2 | 1 1 1 | 1 1 1 | 1 1 1 |
|  |  |  |  | Focus | 1 1 1 | 4 4 4 | 4 4 4 | 4 4 4 | 1 1 1 |
|  |  |  |  | Polarity | 5 5 5 | 2 2 2 | 3 3 3 | 3 3 3 | 7 2 7 |
| 287 | 7129 | 11751639\_226 | LEF-1 isoforms that lack the inhibitory amino-terminal domain bind chromatin more avidly and function as potent feedback inhibitors of Wnt signaling in vivo (dominant negative DN LEF-1). |A1:\*\*1SP3E0| |A2:\*\*1SP3E0-| |A3:\*\*1SP2E1| |A4:\*\*1SP2E1| |A5:\*\*1SP3E1| |  |  |  |  |  |  |
|  |  |  |  | Annotation | 1SP3E0 | 1SP3E0- | 1SP2E1 | 1SP2E1 | 1SP3E1 |
|  |  |  |  | Evidence | 1 | 1 | 2 | 2 | 2 |
|  |  |  |  | Focus | 4 | 4 | 4 | 4 | 4 |
|  |  |  |  | Polarity | 7 | 7 | 6 | 6 | 7 |
| 288 | 3359 | 9390512\_247 | For example, Penrose 1948 put forward a series of arguments why anticipation in myotonic dystrophy might be artifactual. |A1:\*\*1GP1E1| |A2:\*\*1SP1E2| |A3:\*\*1SP2E2| |A4:\*\*1GP2E2| |A5:\*\*1GP3E2| |  |  |  |  |  |  |
|  |  |  |  | Annotation | 1GP1E1 | 1SP1E2 | 1SP2E2 | 1GP2E2 | 1GP3E2 |
|  |  |  |  | Evidence | 2 | 3 | 3 | 3 | 3 |
|  |  |  |  | Focus | 1 | 4 | 4 | 1 | 1 |
|  |  |  |  | Polarity | 5 | 5 | 6 | 6 | 7 |
| 289 | 2044 | 12711603\_26 | Research Fellow of the Japan Society for the Promotion of Science. |A1:\*\*1GP3E0| |A2:\*\*1GP3E0| |A3:\*\*1GP3E3| |A4:\*\*1GP3E0| |A5:\*\*1GP3E0| |  |  |  |  |  |  |
|  |  |  |  | Annotation | 1GP3E0 | 1GP3E0 | 1GP3E3 | 1GP3E0 | 1GP3E0 |
|  |  |  |  | Evidence | 1 | 1 | 4 | 1 | 1 |
|  |  |  |  | Focus | 1 | 1 | 1 | 1 | 1 |
|  |  |  |  | Polarity | 7 | 7 | 7 | 7 | 7 |
| 290 | 330 | 11566983\_221 | Infection with this clone was allowed to persist for 7 months, and then postinfection clones were recovered. |A1:\*\*1SP3E3| |A2:\*\*1SP3E3| |A3:\*\*1SP3E3| |A4:\*\*1SP0E0| |A5:\*\*1GP3E3| |  |  |  |  |  |  |
|  |  |  |  | Annotation | 1SP3E3 | 1SP3E3 | 1SP3E3 | 1SP0E0 | 1GP3E3 |
|  |  |  |  | Evidence | 4 | 4 | 4 | 1 | 4 |
|  |  |  |  | Focus | 4 | 4 | 4 | 4 | 1 |
|  |  |  |  | Polarity | 7 | 7 | 7 | 4 | 7 |
| 291 | 6033 | 12372287\_282 | Five-second prepulses (140mV to 50mV) were applied, followed by a constant test pulse to 0mV. |A1:\*\*1GP3E3| |A2:\*\*1GP3E3| |A3:\*\*1SP3E3| |A4:\*\*1MP3E0| |A5:\*\*1MP3E3| |  |  |  |  |  |  |
|  |  |  |  | Annotation | 1GP3E3 | 1GP3E3 | 1SP3E3 | 1MP3E0 | 1MP3E3 |
|  |  |  |  | Evidence | 4 | 4 | 4 | 1 | 4 |
|  |  |  |  | Focus | 1 | 1 | 4 | 2 | 2 |
|  |  |  |  | Polarity | 7 | 7 | 7 | 7 | 7 |
| 292 | 6483 | 10629053\_379 | Expression of COUP-TF is required for efficient RARbeta induction by RA in cancer cells. |A1:\*\*1SP3E3| |A2:\*\*1SP3E0| |A3:\*\*1SP3E0| |A4:\*\*1SP3E0| |A5:\*\*1SP3E0| |  |  |  |  |  |  |
|  |  |  |  | Annotation | 1SP3E3 | 1SP3E0 | 1SP3E0 | 1SP3E0 | 1SP3E0 |
|  |  |  |  | Evidence | 4 | 1 | 1 | 1 | 1 |
|  |  |  |  | Focus | 4 | 4 | 4 | 4 | 4 |
|  |  |  |  | Polarity | 7 | 7 | 7 | 7 | 7 |
| 293 | 1692 | 12138073\_5 | Clinical groupings were based on the extent of joint restriction: minimal (group A), and moderate-severe (with supinatory foot deformity (group B), or with pronatory foot deformity (group C)). |A1:\*\*1GP3E3| |A2:\*\*1GP3E3| |A3:\*\*1SP3E3| |A4:\*\*1GP3E3| |A5:\*\*1GP3E3| |  |  |  |  |  |  |
|  |  |  |  | Annotation | 1GP3E3 | 1GP3E3 | 1SP3E3 | 1GP3E3 | 1GP3E3 |
|  |  |  |  | Evidence | 4 | 4 | 4 | 4 | 4 |
|  |  |  |  | Focus | 1 | 1 | 4 | 1 | 1 |
|  |  |  |  | Polarity | 7 | 7 | 7 | 7 | 7 |
| 294 | 3814 | 10973452\_119 | These observations indicate that although cryopreserved cells from the MACS appear to be suitable for other laboratory studies (12), |A2:\*\*1SP2E2| |A3:\*\*1SP2E2| |A4:\*\*1SP3E2| the use of fresh cells may be preferable for evaluating cytokine responses in vitro. |A2:\*\*2SP2E0| |A1:\*\*1SP1E23| |A3:\*\*2SP1E0| |A4:\*\*2MP2E0| |A5:\*\*1GP3E2| |  |  |  |  |  |  |
|  |  |  |  | Annotation | 1SP1E23 1SP1E23 | 1SP2E2 2SP2E0 | 1SP2E2 2SP1E0 | 1SP3E2 2MP2E0 | 1GP3E2 1GP3E2 |
|  |  |  |  | Evidence | 3 3 | 3 1 | 3 1 | 3 1 | 3 3 |
|  |  |  |  | Focus | 4 4 | 4 4 | 4 4 | 4 2 | 1 1 |
|  |  |  |  | Polarity | 5 5 | 6 6 | 6 5 | 7 6 | 7 7 |
| 295 | 8502 | 12372285\_126 | The tyrosine phosphorylation level of Plex-A2 was reduced without NP-1, |A2:\*\*1SP3E3-| |A4:\*\*1SP3E0-| |A5:\*\*1SP3E1-| suggesting that NP-1 might have a cooperative effect on Plex-A2 phosphorylation by Fyn. |A2:\*\*2SP2E1| |A1:\*\*1SP1E3-| |A3:\*\*1SP2E0| |A4:\*\*2SP2E1| |A5:\*\*2SP2E1| |  |  |  |  |  |  |
|  |  |  |  | Annotation | 1SP1E3- 1SP1E3- | 1SP3E3- 2SP2E1 | 1SP2E0 1SP2E0 | 1SP3E0- 2SP2E1 | 1SP3E1- 2SP2E1 |
|  |  |  |  | Evidence | 4 4 | 4 2 | 1 1 | 1 2 | 2 2 |
|  |  |  |  | Focus | 4 4 | 4 4 | 4 4 | 4 4 | 4 4 |
|  |  |  |  | Polarity | 5 5 | 7 6 | 6 6 | 7 6 | 7 6 |
| 296 | 9162 | 11448948\_98 | Cell extracts (50 mug of total cellular protein) were analyzed by SDS-PAGE, |A1:\*\*1MP3E3| and the expression of p27 was determined by Western blotting. |A1:\*\*2MP3E3| |A2:\*\*1MP3E3| |A3:\*\*1MP3E3| |A4:\*\*1MP3E3| |A5:\*\*1MP3E3| |  |  |  |  |  |  |
|  |  |  |  | Annotation | 1MP3E3 2MP3E3 | 1MP3E3 1MP3E3 | 1MP3E3 1MP3E3 | 1MP3E3 1MP3E3 | 1MP3E3 1MP3E3 |
|  |  |  |  | Evidence | 4 4 | 4 4 | 4 4 | 4 4 | 4 4 |
|  |  |  |  | Focus | 2 2 | 2 2 | 2 2 | 2 2 | 2 2 |
|  |  |  |  | Polarity | 7 7 | 7 7 | 7 7 | 7 7 | 7 7 |
| 297 | 788 | 10790380\_113 | Rv3135, a member of the PPE family of proteins, was uniquely variable. |A1:\*\*1SP3E3| |A2:\*\*1SP3E0| |A3:\*\*1SP3E3| |A4:\*\*1SP3E0| |A5:\*\*1GP3E0| |  |  |  |  |  |  |
|  |  |  |  | Annotation | 1SP3E3 | 1SP3E0 | 1SP3E3 | 1SP3E0 | 1GP3E0 |
|  |  |  |  | Evidence | 4 | 1 | 4 | 1 | 1 |
|  |  |  |  | Focus | 4 | 4 | 4 | 4 | 1 |
|  |  |  |  | Polarity | 7 | 7 | 7 | 7 | 7 |
| 298 | 4355 | 11076973\_263 | However, injections of the anti - Lva antibody cause severe furrowing defects (Fig 6 A). |A1:\*\*1SP3E3| |A2:\*\*1SP3E3| |A3:\*\*1SP3E3| |A4:\*\*1SP3E3| |A5:\*\*1GP3E3| |  |  |  |  |  |  |
|  |  |  |  | Annotation | 1SP3E3 | 1SP3E3 | 1SP3E3 | 1SP3E3 | 1GP3E3 |
|  |  |  |  | Evidence | 4 | 4 | 4 | 4 | 4 |
|  |  |  |  | Focus | 4 | 4 | 4 | 4 | 1 |
|  |  |  |  | Polarity | 7 | 7 | 7 | 7 | 7 |
| 299 | 7722 | 9741697\_133 | Sphingomyelin and CPHS added separately or in combination to 1% Triton X-100 were solubilized by the detergent. |A1:\*\*1SP3E3| |A2:\*\*1MP3E3| |A3:\*\*1MP3E3| |A4:\*\*1MP3E0| |A5:\*\*1MP3E3| |  |  |  |  |  |  |
|  |  |  |  | Annotation | 1SP3E3 | 1MP3E3 | 1MP3E3 | 1MP3E0 | 1MP3E3 |
|  |  |  |  | Evidence | 4 | 4 | 4 | 1 | 4 |
|  |  |  |  | Focus | 4 | 2 | 2 | 2 | 2 |
|  |  |  |  | Polarity | 7 | 7 | 7 | 7 | 7 |
| 300 | 3355 | 10077572\_20 | Cytoplasmic GSH is maintained in its reduced form through the action of the ubiquitous enzyme GSH reductase, |A5:\*\*1SP3E0| which catalyzes the reaction: GSSG + NADPH + H+ 2 GSH + NADP+. |A5:\*\*2SP3E0+| |A1:\*\*1SP3E3| |A2:\*\*1SP3E0| |A3:\*\*1SP3E0| |A4:\*\*1SP3E0| |  |  |  |  |  |  |
|  |  |  |  | Annotation | 1SP3E3 1SP3E3 | 1SP3E0 1SP3E0 | 1SP3E0 1SP3E0 | 1SP3E0 1SP3E0 | 1SP3E0 2SP3E0+ |
|  |  |  |  | Evidence | 4 4 | 1 1 | 1 1 | 1 1 | 1 1 |
|  |  |  |  | Focus | 4 4 | 4 4 | 4 4 | 4 4 | 4 4 |
|  |  |  |  | Polarity | 7 7 | 7 7 | 7 7 | 7 7 | 7 7 |
| 301 | 3350 | 10635333\_195 | We are grateful to J. Blenis (Harvard Medical School, Boston, MA) for the RSK and RSK mutant expression vectors and RSK antibodies, E. Olson (University of Texas, Dallas, TX) for the PKC expression vectors, and P. Johnson (NCI-Frederick Cancer Research and Development Program) for the mouse C/EBP construct. |A1:\*\*1GP3E0| |A2:\*\*1SP3E3| |A3:\*\*1SP3E0| |A4:\*\*1SP3E3| |A5:\*\*1GP3E0| |  |  |  |  |  |  |
|  |  |  |  | Annotation | 1GP3E0 | 1SP3E3 | 1SP3E0 | 1SP3E3 | 1GP3E0 |
|  |  |  |  | Evidence | 1 | 4 | 1 | 4 | 1 |
|  |  |  |  | Focus | 1 | 4 | 4 | 4 | 1 |
|  |  |  |  | Polarity | 7 | 7 | 7 | 7 | 7 |
| 302 | 5003 | 9520407\_209 | Thus, the transition itself may involve the loss of one or more critically coordinated magnesium ions, believed to exist in the 8-12 turn and between the D and T loops. |A1:\*\*1SP1E3| |A2:\*\*1SP2E0| |A3:\*\*1SP1E0| |A4:\*\*1SP1E1-| |A5:\*\*1SP3E1| |  |  |  |  |  |  |
|  |  |  |  | Annotation | 1SP1E3 | 1SP2E0 | 1SP1E0 | 1SP1E1- | 1SP3E1 |
|  |  |  |  | Evidence | 4 | 1 | 1 | 2 | 2 |
|  |  |  |  | Focus | 4 | 4 | 4 | 4 | 4 |
|  |  |  |  | Polarity | 5 | 6 | 5 | 5 | 7 |
| 303 | 5862 | 9660872\_275 | Enomoto (1996) has suggested that microtubules bind signaling factors that are released either in a controlled way under normal conditions, or globally when microtubules are disrupted. |A1:\*\*1SP3E1| |A2:\*\*1SP3E2| |A3:\*\*1SP2E0| |A4:\*\*1SP2E2| |A5:\*\*1SP3E2| |  |  |  |  |  |  |
|  |  |  |  | Annotation | 1SP3E1 | 1SP3E2 | 1SP2E0 | 1SP2E2 | 1SP3E2 |
|  |  |  |  | Evidence | 2 | 3 | 1 | 3 | 3 |
|  |  |  |  | Focus | 4 | 4 | 4 | 4 | 4 |
|  |  |  |  | Polarity | 7 | 7 | 6 | 6 | 7 |
| 304 | 5803 | 12134252\_1 | Human herpesvirus (HHV)-6 is a beta-herpesvirus-like human cytomegalovirus (HCMV) with the potential to reactivate in immunocompromised persons. |A1:\*\*1GP3E0| |A2:\*\*1SP3E0+| |A3:\*\*1SP3E0| |A4:\*\*1SP3E0| |A5:\*\*1SP3E0| |  |  |  |  |  |  |
|  |  |  |  | Annotation | 1GP3E0 | 1SP3E0+ | 1SP3E0 | 1SP3E0 | 1SP3E0 |
|  |  |  |  | Evidence | 1 | 1 | 1 | 1 | 1 |
|  |  |  |  | Focus | 1 | 4 | 4 | 4 | 4 |
|  |  |  |  | Polarity | 7 | 7 | 7 | 7 | 7 |
| 305 | 4071 | 9405414\_29 | Probably because there is redundancy of function with other MS channels |A3:\*\*1SP2E0| |A5:\*\*1GP2E0-| no phenotype has yet been reported for the E. coli mscL-null mutant. |A3:\*\*2SN3E0| |A1:\*\*1SP2E1| |A2:\*\*1SN3E0| |A4:\*\*1SN2E0| |A5:\*\*2GN3E1| |  |  |  |  |  |  |
|  |  |  |  | Annotation | 1SP2E1 1SP2E1 | 1SN3E0 1SN3E0 | 1SP2E0 2SN3E0 | 1SN2E0 1SN2E0 | 1GP2E0- 2GN3E1 |
|  |  |  |  | Evidence | 2 2 | 1 1 | 1 1 | 1 1 | 1 2 |
|  |  |  |  | Focus | 4 4 | 4 4 | 4 4 | 4 4 | 1 1 |
|  |  |  |  | Polarity | 6 6 | 1 1 | 6 1 | 2 2 | 6 1 |
| 306 | 8569 | 9054509\_207 | The intimate link between resistance and extent of spontaneous leaf lesions has been studied on the basis of an allelic series of 95 chemically induced mlo alleles (Habekuss and Hentrich, 1988). |A1:\*\*1SP2E2| |A2:\*\*1SP2E2| |A3:\*\*1SP3E2| |A4:\*\*1SP3E2| |A5:\*\*1SP3E2| |  |  |  |  |  |  |
|  |  |  |  | Annotation | 1SP2E2 | 1SP2E2 | 1SP3E2 | 1SP3E2 | 1SP3E2 |
|  |  |  |  | Evidence | 3 | 3 | 3 | 3 | 3 |
|  |  |  |  | Focus | 4 | 4 | 4 | 4 | 4 |
|  |  |  |  | Polarity | 6 | 6 | 7 | 7 | 7 |
| 307 | 3906 | 10101156\_185 | These thermosensitive alleles, especially dcp1-2, should be useful for the analysis of mRNA decay ( e.g., see J ACOBS ANDERSON and PARKER 1998 ). |A1:\*\*1SP2E2| |A2:\*\*1SP2E2| |A3:\*\*1SP1E0| |A4:\*\*1SP2E2| |A5:\*\*1GP3E2| |  |  |  |  |  |  |
|  |  |  |  | Annotation | 1SP2E2 | 1SP2E2 | 1SP1E0 | 1SP2E2 | 1GP3E2 |
|  |  |  |  | Evidence | 3 | 3 | 1 | 3 | 3 |
|  |  |  |  | Focus | 4 | 4 | 4 | 4 | 1 |
|  |  |  |  | Polarity | 6 | 6 | 5 | 6 | 7 |
| 308 | 1538 | 10391869\_46 | Pentoxifylline (5.5 mg/animal) was given 3 h after the toxin, and LPS was injected at 4 h. |A1:\*\*1SP3E3| |A2:\*\*1SP3E3| |A3:\*\*1SP3E3| |A4:\*\*1MP3E3| |A5:\*\*1MP3E3| |  |  |  |  |  |  |
|  |  |  |  | Annotation | 1SP3E3 | 1SP3E3 | 1SP3E3 | 1MP3E3 | 1MP3E3 |
|  |  |  |  | Evidence | 4 | 4 | 4 | 4 | 4 |
|  |  |  |  | Focus | 4 | 4 | 4 | 2 | 2 |
|  |  |  |  | Polarity | 7 | 7 | 7 | 7 | 7 |
| 309 | 2601 | 12729560\_305 | A role of G signaling and PKC in the regulation of Cdc42 by Wnt-11 |A1:\*\*1SP0E0| |A2:\*\*1SP0E0| |A3:\*\*1SP3E0| |A4:\*\*1SP3E0| |A5:\*\*1SP3E0| |  |  |  |  |  |  |
|  |  |  |  | Annotation | 1SP0E0 | 1SP0E0 | 1SP3E0 | 1SP3E0 | 1SP3E0 |
|  |  |  |  | Evidence | 1 | 1 | 1 | 1 | 1 |
|  |  |  |  | Focus | 4 | 4 | 4 | 4 | 4 |
|  |  |  |  | Polarity | 4 | 4 | 7 | 7 | 7 |
| 310 | 2899 | 9463371\_104 | Interaction of various recombinant PDI fragments with biotinylated `scrambled'' RNase. |A1:\*\*1SP0E0| |A2:\*\*1SP0E0| |A3:\*\*1SP3E0| |A4:\*\*1SP3E0| |A5:\*\*1SP3E0| |  |  |  |  |  |  |
|  |  |  |  | Annotation | 1SP0E0 | 1SP0E0 | 1SP3E0 | 1SP3E0 | 1SP3E0 |
|  |  |  |  | Evidence | 1 | 1 | 1 | 1 | 1 |
|  |  |  |  | Focus | 4 | 4 | 4 | 4 | 4 |
|  |  |  |  | Polarity | 4 | 4 | 7 | 7 | 7 |
| 311 | 5710 | 10569766\_71 | This vector permits the expression of cloned genes containing the initial ATG codon of the signal sequence and is expressed with an appended (His)6 tail at the C terminus to facilitate purification. |A1:\*\*1SP3E0| |A2:\*\*1SP3E1| |A3:\*\*1SP3E0| |A4:\*\*1SP3E0| |A5:\*\*1SP3E3+| |  |  |  |  |  |  |
|  |  |  |  | Annotation | 1SP3E0 | 1SP3E1 | 1SP3E0 | 1SP3E0 | 1SP3E3+ |
|  |  |  |  | Evidence | 1 | 2 | 1 | 1 | 4 |
|  |  |  |  | Focus | 4 | 4 | 4 | 4 | 4 |
|  |  |  |  | Polarity | 7 | 7 | 7 | 7 | 7 |
| 312 | 6958 | 12113534\_5 | This current study was performed to validate our earlier findings in a separate, statewide, population-based dataset of trauma victims. |A1:\*\*1GP3E3| |A2:\*\*1GP3E3| |A3:\*\*1GP3E3| |A4:\*\*1GP3E3| |A5:\*\*1GP3E3| |  |  |  |  |  |  |
|  |  |  |  | Annotation | 1GP3E3 | 1GP3E3 | 1GP3E3 | 1GP3E3 | 1GP3E3 |
|  |  |  |  | Evidence | 4 | 4 | 4 | 4 | 4 |
|  |  |  |  | Focus | 1 | 1 | 1 | 1 | 1 |
|  |  |  |  | Polarity | 7 | 7 | 7 | 7 | 7 |
| 313 | 8838 | 12496348\_6 | Abnormalities in the brain's dopamine system have been postulated for both schizophrenia (SCHIZ) and bipolar disorder (BPD) ( 1-4). |A1:\*\*1GP3E2| |A2:\*\*1SP3E2| |A3:\*\*1SP3E2| |A4:\*\*1SP3E2| |A5:\*\*1GP3E2| |  |  |  |  |  |  |
|  |  |  |  | Annotation | 1GP3E2 | 1SP3E2 | 1SP3E2 | 1SP3E2 | 1GP3E2 |
|  |  |  |  | Evidence | 3 | 3 | 3 | 3 | 3 |
|  |  |  |  | Focus | 1 | 4 | 4 | 4 | 1 |
|  |  |  |  | Polarity | 7 | 7 | 7 | 7 | 7 |
| 314 | 7581 | 9407054\_68 | The maximum levels of TNF-alpha and IL-1beta elevated in plasma following the LPS administration (1 and 5 h later, respectively) were then examined. |A1:\*\*1SP3E3| |A2:\*\*1SP3E3| |A3:\*\*1SP3E3| |A4:\*\*1SP3E3+| |A5:\*\*1MP3E3+| |  |  |  |  |  |  |
|  |  |  |  | Annotation | 1SP3E3 | 1SP3E3 | 1SP3E3 | 1SP3E3+ | 1MP3E3+ |
|  |  |  |  | Evidence | 4 | 4 | 4 | 4 | 4 |
|  |  |  |  | Focus | 4 | 4 | 4 | 4 | 2 |
|  |  |  |  | Polarity | 7 | 7 | 7 | 7 | 7 |
| 315 | 2130 | 12102333\_2 | Mean values (M +/- SD) for the investigated parameters (on dry weight basis) were: 23.0 +/- 3.9%, 10.7 +/- 2.4% and 2.17 +/- 0.31, respectively. |A1:\*\*1SP3E3| |A2:\*\*1SP3E3| |A3:\*\*1SP3E3| |A4:\*\*1GP3E0| |A5:\*\*1GP3E3| |  |  |  |  |  |  |
|  |  |  |  | Annotation | 1SP3E3 | 1SP3E3 | 1SP3E3 | 1GP3E0 | 1GP3E3 |
|  |  |  |  | Evidence | 4 | 4 | 4 | 1 | 4 |
|  |  |  |  | Focus | 4 | 4 | 4 | 1 | 1 |
|  |  |  |  | Polarity | 7 | 7 | 7 | 7 | 7 |
| 316 | 9889 | 9469582\_186 | To confirm the HPGC cholesterol profile results, plasmas from PD 72953-treated rats were subjected to lipoprotein electrophoresis and oil red O staining |A1:\*\*1SP3E3| |A2:\*\*1SP2E3| |A3:\*\*1SP3E3| |A4:\*\*1SP3E3| |A5:\*\*1MP3E3| |  |  |  |  |  |  |
|  |  |  |  | Annotation | 1SP3E3 | 1SP2E3 | 1SP3E3 | 1SP3E3 | 1MP3E3 |
|  |  |  |  | Evidence | 4 | 4 | 4 | 4 | 4 |
|  |  |  |  | Focus | 4 | 4 | 4 | 4 | 2 |
|  |  |  |  | Polarity | 7 | 6 | 7 | 7 | 7 |
| 317 | 2315 | 12006917\_1 | BACKGROUND: C-reactive protein (CRP) has been linked to cardiovascular disease and atherosclerosis. |A1:\*\*1GP3E1| |A2:\*\*1SP2E0| |A3:\*\*1SP3E0| |A4:\*\*1SP2E0| |A5:\*\*1GP3E1| |  |  |  |  |  |  |
|  |  |  |  | Annotation | 1GP3E1 | 1SP2E0 | 1SP3E0 | 1SP2E0 | 1GP3E1 |
|  |  |  |  | Evidence | 2 | 1 | 1 | 1 | 2 |
|  |  |  |  | Focus | 1 | 4 | 4 | 4 | 1 |
|  |  |  |  | Polarity | 7 | 6 | 7 | 6 | 7 |
| 318 | 9455 | 9362061\_239 | This process, termed "`constitutive-like secretion"` from ISGs, has slower kinetics than constitutive secretion from the TGN in other cell types. |A1:\*\*1SP3E3| |A2:\*\*1SP3E1| |A3:\*\*1SP3E0| |A4:\*\*1SP3E0| |A5:\*\*1GP3E0| |  |  |  |  |  |  |
|  |  |  |  | Annotation | 1SP3E3 | 1SP3E1 | 1SP3E0 | 1SP3E0 | 1GP3E0 |
|  |  |  |  | Evidence | 4 | 2 | 1 | 1 | 1 |
|  |  |  |  | Focus | 4 | 4 | 4 | 4 | 1 |
|  |  |  |  | Polarity | 7 | 7 | 7 | 7 | 7 |
| 319 | 9313 | 12099308\_6 | There are numerous formulas for estimating fetal weight, and they are all error prone. |A1:\*\*1GP0E0| |A2:\*\*1GP1E0| |A3:\*\*1SP3E0| |A4:\*\*1SP3E0| |A5:\*\*1GP3E0| |  |  |  |  |  |  |
|  |  |  |  | Annotation | 1GP0E0 | 1GP1E0 | 1SP3E0 | 1SP3E0 | 1GP3E0 |
|  |  |  |  | Evidence | 1 | 1 | 1 | 1 | 1 |
|  |  |  |  | Focus | 1 | 1 | 4 | 4 | 1 |
|  |  |  |  | Polarity | 4 | 5 | 7 | 7 | 7 |
| 320 | 340 | 9560277\_95 | However, these laminar differences did not achieve statistical significance. |A1:\*\*1GN3E1| |A2:\*\*1GN3E1| |A3:\*\*1SN3E0| |A4:\*\*1SN2E0| |A5:\*\*1GN3E1| |  |  |  |  |  |  |
|  |  |  |  | Annotation | 1GN3E1 | 1GN3E1 | 1SN3E0 | 1SN2E0 | 1GN3E1 |
|  |  |  |  | Evidence | 2 | 2 | 1 | 1 | 2 |
|  |  |  |  | Focus | 1 | 1 | 4 | 4 | 1 |
|  |  |  |  | Polarity | 1 | 1 | 1 | 2 | 1 |
| 321 | 2333 | 9490721\_124 | HRP (5 mg/ ml) was internalized for 30 min, |A1:\*\*1MP3E3| and the Golgi was labeled with NBD-ceramide, as described in Materials and Methods. |A1:\*\*2MP3E3| |A2:\*\*1MP3E3| |A3:\*\*1MP3E3| |A4:\*\*1MP3E3| |A5:\*\*1MP3E3| |  |  |  |  |  |  |
|  |  |  |  | Annotation | 1MP3E3 2MP3E3 | 1MP3E3 1MP3E3 | 1MP3E3 1MP3E3 | 1MP3E3 1MP3E3 | 1MP3E3 1MP3E3 |
|  |  |  |  | Evidence | 4 4 | 4 4 | 4 4 | 4 4 | 4 4 |
|  |  |  |  | Focus | 2 2 | 2 2 | 2 2 | 2 2 | 2 2 |
|  |  |  |  | Polarity | 7 7 | 7 7 | 7 7 | 7 7 | 7 7 |
| 322 | 6446 | 10702503\_54 | Densitometric tracks from each lane were normalized with respect to a molecular size standard (250-bp ladder; Gibco/BRL, Gaithersburg, Md.), which was included in four lanes on every gel, and then were compared in a pairwise fashion with tracks from other lanes from the same gel or different gels. |A1:\*\*1MP3E3| |A2:\*\*1MSP3E3| |A3:\*\*1MP3E3| |A4:\*\*1MP3E3| |A5:\*\*1MP3E3| |  |  |  |  |  |  |
|  |  |  |  | Annotation | 1MP3E3 | 1MSP3E3 | 1MP3E3 | 1MP3E3 | 1MP3E3 |
|  |  |  |  | Evidence | 4 | 4 | 4 | 4 | 4 |
|  |  |  |  | Focus | 2 | 6 | 2 | 2 | 2 |
|  |  |  |  | Polarity | 7 | 7 | 7 | 7 | 7 |
| 323 | 180 | 9334316\_288 | Thus, Bru has multiple roles in development; given its role in repression of osk mRNA translation, |A2:\*\*1SP2E1-| |A3:\*\*1SP3E0| |A4:\*\*1SP3E3-| |A5:\*\*1SP3E1| we expect that Bru regulates the translation of multiple transcripts. |A2:\*\*1SP1E0| |A1:\*\*1SN2E3| |A3:\*\*2SP1E0| |A4:\*\*2SP2E0| |A5:\*\*2SP2E0| |  |  |  |  |  |  |
|  |  |  |  | Annotation | 1SN2E3 1SN2E3 | 1SP2E1- 1SP1E0 | 1SP3E0 2SP1E0 | 1SP3E3- 2SP2E0 | 1SP3E1 2SP2E0 |
|  |  |  |  | Evidence | 4 4 | 2 1 | 1 1 | 4 1 | 2 1 |
|  |  |  |  | Focus | 4 4 | 4 4 | 4 4 | 4 4 | 4 4 |
|  |  |  |  | Polarity | 2 2 | 6 5 | 7 5 | 7 6 | 7 6 |
| 324 | 2699 | 11526013\_78 | During the first step, the molecular mass was determined from the measured m/z values for the protonated molecules. |A1:\*\*1GP3E3| |A2:\*\*1GP3E3| |A3:\*\*1SP3E3| |A4:\*\*1SP3E3| |A5:\*\*1MP3E3| |  |  |  |  |  |  |
|  |  |  |  | Annotation | 1GP3E3 | 1GP3E3 | 1SP3E3 | 1SP3E3 | 1MP3E3 |
|  |  |  |  | Evidence | 4 | 4 | 4 | 4 | 4 |
|  |  |  |  | Focus | 1 | 1 | 4 | 4 | 2 |
|  |  |  |  | Polarity | 7 | 7 | 7 | 7 | 7 |
| 325 | 3503 | 9488491\_278 | In an effort to characterize the domains of BKV TAg required for the induction of E2F, |A4:\*\*1SP3E3+| we used mutations in the pRb-binding and J domains to show that both of these domains are required for the induction of E2F activity. |A4:\*\*2SP3E3| |A1:\*\*1SP3E3| |A2:\*\*1SP3E3| |A3:\*\*1SP3E3+| |A5:\*\*1SP3E3| |  |  |  |  |  |  |
|  |  |  |  | Annotation | 1SP3E3 1SP3E3 | 1SP3E3 1SP3E3 | 1SP3E3+ 1SP3E3+ | 1SP3E3+ 2SP3E3 | 1SP3E3 1SP3E3 |
|  |  |  |  | Evidence | 4 4 | 4 4 | 4 4 | 4 4 | 4 4 |
|  |  |  |  | Focus | 4 4 | 4 4 | 4 4 | 4 4 | 4 4 |
|  |  |  |  | Polarity | 7 7 | 7 7 | 7 7 | 7 7 | 7 7 |
| 326 | 8326 | 8978603\_322 | DNA was prepared from whole blood using the Puregene kit. |A1:\*\*1SP3E3| |A2:\*\*1SP3E3| |A3:\*\*1MP3E3| |A4:\*\*1SP3E3| |A5:\*\*1MP3E3| |  |  |  |  |  |  |
|  |  |  |  | Annotation | 1SP3E3 | 1SP3E3 | 1MP3E3 | 1SP3E3 | 1MP3E3 |
|  |  |  |  | Evidence | 4 | 4 | 4 | 4 | 4 |
|  |  |  |  | Focus | 4 | 4 | 2 | 4 | 2 |
|  |  |  |  | Polarity | 7 | 7 | 7 | 7 | 7 |
| 327 | 2036 | 9512515\_50 | Two of these ES cell clones were used to generate chimeric mice, and both successfully contributed to the germ line. |A1:\*\*1SP3E3| |A2:\*\*1SP3E3| |A3:\*\*1SP3E3| |A4:\*\*1SP3E3| |A5:\*\*1GP3E3| |  |  |  |  |  |  |
|  |  |  |  | Annotation | 1SP3E3 | 1SP3E3 | 1SP3E3 | 1SP3E3 | 1GP3E3 |
|  |  |  |  | Evidence | 4 | 4 | 4 | 4 | 4 |
|  |  |  |  | Focus | 4 | 4 | 4 | 4 | 1 |
|  |  |  |  | Polarity | 7 | 7 | 7 | 7 | 7 |
| 328 | 6163 | 11682507\_123 | Interestingly, this genotype Ba infection was the only genotype Ba sample in the entire study. |A1:\*\*1GP3E3| |A2:\*\*1SP3E3| |A3:\*\*1SP3E3| |A4:\*\*1SP3E3| |A5:\*\*1GP3E1| |  |  |  |  |  |  |
|  |  |  |  | Annotation | 1GP3E3 | 1SP3E3 | 1SP3E3 | 1SP3E3 | 1GP3E1 |
|  |  |  |  | Evidence | 4 | 4 | 4 | 4 | 2 |
|  |  |  |  | Focus | 1 | 4 | 4 | 4 | 1 |
|  |  |  |  | Polarity | 7 | 7 | 7 | 7 | 7 |
| 329 | 4628 | 9827803\_115 | The vessels in the cortex of the v-null embryo are markedly dilated and filled with nucleated red blood cells (M) in comparison with the wild-type cortex (L). |A1:\*\*1SP3E0| |A2:\*\*1SP3E0| |A3:\*\*1SP3E0| |A4:\*\*1SP3E0| |A5:\*\*1GP3E3| |  |  |  |  |  |  |
|  |  |  |  | Annotation | 1SP3E0 | 1SP3E0 | 1SP3E0 | 1SP3E0 | 1GP3E3 |
|  |  |  |  | Evidence | 1 | 1 | 1 | 1 | 4 |
|  |  |  |  | Focus | 4 | 4 | 4 | 4 | 1 |
|  |  |  |  | Polarity | 7 | 7 | 7 | 7 | 7 |
| 330 | 4688 | 9844016\_20 | Varying levels of gamma2S and gamma2L occur in cerebral cortex (36-39). |A1:\*\*1SP3E2| |A2:\*\*1SP3E2| |A3:\*\*1SP3E2| |A4:\*\*1SP3E2| |A5:\*\*1GP3E2| |  |  |  |  |  |  |
|  |  |  |  | Annotation | 1SP3E2 | 1SP3E2 | 1SP3E2 | 1SP3E2 | 1GP3E2 |
|  |  |  |  | Evidence | 3 | 3 | 3 | 3 | 3 |
|  |  |  |  | Focus | 4 | 4 | 4 | 4 | 1 |
|  |  |  |  | Polarity | 7 | 7 | 7 | 7 | 7 |
| 331 | 7017 | 12081574\_4 | SHR 4 to 6 weeks of age were pre-hypertensive, 6 to 7 weeks old had mild hypertension, and 8 to 13 weeks old had severe hypertension. |A1:\*\*1SP3E3| |A2:\*\*1SP3E3| |A3:\*\*1SP3E3| |A4:\*\*1SP3E0| |A5:\*\*1GP3E1| |  |  |  |  |  |  |
|  |  |  |  | Annotation | 1SP3E3 | 1SP3E3 | 1SP3E3 | 1SP3E0 | 1GP3E1 |
|  |  |  |  | Evidence | 4 | 4 | 4 | 1 | 2 |
|  |  |  |  | Focus | 4 | 4 | 4 | 4 | 1 |
|  |  |  |  | Polarity | 7 | 7 | 7 | 7 | 7 |
| 332 | 5918 | 10811829\_69 | Neurite Outgrowth Assay Cells were fixed 24 h after transfection transfection. |A1:\*\*1SP3E3| |A2:\*\*1MP3E3| |A3:\*\*1SP3E3| |A4:\*\*1MP3E3| |A5:\*\*1MP3E3| |  |  |  |  |  |  |
|  |  |  |  | Annotation | 1SP3E3 | 1MP3E3 | 1SP3E3 | 1MP3E3 | 1MP3E3 |
|  |  |  |  | Evidence | 4 | 4 | 4 | 4 | 4 |
|  |  |  |  | Focus | 4 | 2 | 4 | 2 | 2 |
|  |  |  |  | Polarity | 7 | 7 | 7 | 7 | 7 |
| 333 | 8988 | 12362329\_7 | The information in YPRC-PDB is updated easily and it is available to authorized users on the World Wide Web (http://yprcpdb.proteomix.org/ |A1:\*\*1GP3E0| |A2:\*\*1GP3E2| |A3:\*\*1SP3E3| |A4:\*\*1SP3E3| |A5:\*\*1GP3E3| |  |  |  |  |  |  |
|  |  |  |  | Annotation | 1GP3E0 | 1GP3E2 | 1SP3E3 | 1SP3E3 | 1GP3E3 |
|  |  |  |  | Evidence | 1 | 3 | 4 | 4 | 4 |
|  |  |  |  | Focus | 1 | 1 | 4 | 4 | 1 |
|  |  |  |  | Polarity | 7 | 7 | 7 | 7 | 7 |
| 334 | 3508 | 10449458\_65 | The reaction was carried out in a RoboCycler Gradient 96 Temperature Cycler (Stratagene) for 60 min at 37 degrees C and then for 10 min at 65 degrees C. |A1:\*\*1MP3E3| |A2:\*\*1MP3E3| |A3:\*\*1SP3E3| |A4:\*\*1MP3E3| |A5:\*\*1MP3E3| |  |  |  |  |  |  |
|  |  |  |  | Annotation | 1MP3E3 | 1MP3E3 | 1SP3E3 | 1MP3E3 | 1MP3E3 |
|  |  |  |  | Evidence | 4 | 4 | 4 | 4 | 4 |
|  |  |  |  | Focus | 2 | 2 | 4 | 2 | 2 |
|  |  |  |  | Polarity | 7 | 7 | 7 | 7 | 7 |
| 335 | 8346 | 10617652\_12 | Conceivably, medulloblastomas may recapitulate some properties of these cells |A3:\*\*1SP1E0| and thereby undergo apoptosis upon Ras activation. |A3:\*\*2SP3E0| |A1:\*\*1SP1E0| |A2:\*\*1SP2E0+| |A4:\*\*1SP2E0| |A5:\*\*1SP1E0| |  |  |  |  |  |  |
|  |  |  |  | Annotation | 1SP1E0 1SP1E0 | 1SP2E0+ 1SP2E0+ | 1SP1E0 2SP3E0 | 1SP2E0 1SP2E0 | 1SP1E0 1SP1E0 |
|  |  |  |  | Evidence | 1 1 | 1 1 | 1 1 | 1 1 | 1 1 |
|  |  |  |  | Focus | 4 4 | 4 4 | 4 4 | 4 4 | 4 4 |
|  |  |  |  | Polarity | 5 5 | 6 6 | 5 7 | 6 6 | 5 5 |
| 336 | 5515 | 11292744\_211 | This difference may have resulted from a more disparate geographic distribution of the latter samples, as the ticks that were used to experimentally infect the dogs probably were collected in a more constrained region of the state of New York than that from which the clinically diagnosed samples originated. |A1:\*\*1GP1E3| |A2:\*\*1GP2E3| |A3:\*\*1MP2E3| |A4:\*\*1MP2E1-| |A5:\*\*1GP3E3| |  |  |  |  |  |  |
|  |  |  |  | Annotation | 1GP1E3 | 1GP2E3 | 1MP2E3 | 1MP2E1- | 1GP3E3 |
|  |  |  |  | Evidence | 4 | 4 | 4 | 2 | 4 |
|  |  |  |  | Focus | 1 | 1 | 2 | 2 | 1 |
|  |  |  |  | Polarity | 5 | 6 | 6 | 6 | 7 |
| 337 | 9848 | 9407085\_1 | RESULTS Comparison of Activities of the H1t and H1d Promoters in NIH3T3 Cells |A5:\*\*1SP3E3| It was previously reported that the promoter from common somatic H1 variant H1d was considerably stronger than the H1t promoter in directing expression of the chloramphenicol acetyl transferase gene in stably transfected mouse L cells ( ). |A5:\*\*2SP3E2| |A1:\*\*1SP3E23| |A2:\*\*1SP2E12| |A3:\*\*1SP3E3| |A4:\*\*1SP3E3| |  |  |  |  |  |  |
|  |  |  |  | Annotation | 1SP3E23 1SP3E23 | 1SP2E12 1SP2E12 | 1SP3E3 1SP3E3 | 1SP3E3 1SP3E3 | 1SP3E3 2SP3E2 |
|  |  |  |  | Evidence | 3 3 | 2 2 | 4 4 | 4 4 | 4 3 |
|  |  |  |  | Focus | 4 4 | 4 4 | 4 4 | 4 4 | 4 4 |
|  |  |  |  | Polarity | 7 7 | 6 6 | 7 7 | 7 7 | 7 7 |
| 338 | 2766 | 11466272\_125 | Another distinguishing characteristic of DPG-dependent and Mn-dependent Gpms is that they differ in their optimum pH ( 4). |A1:\*\*1SP3E2| |A2:\*\*1SP3E2| |A3:\*\*1SP3E2| |A4:\*\*1SP3E2| |A5:\*\*1SP3E2| |  |  |  |  |  |  |
|  |  |  |  | Annotation | 1SP3E2 | 1SP3E2 | 1SP3E2 | 1SP3E2 | 1SP3E2 |
|  |  |  |  | Evidence | 3 | 3 | 3 | 3 | 3 |
|  |  |  |  | Focus | 4 | 4 | 4 | 4 | 4 |
|  |  |  |  | Polarity | 7 | 7 | 7 | 7 | 7 |
| 339 | 553 | 8752214\_140 | Detection and quantitation of endogenous noggin and BMP proteins in the embryo remains problematic. |A1:\*\*1SP0E0| |A2:\*\*1SP0E0| |A3:\*\*1SP3E0| |A4:\*\*1SP3E0| |A5:\*\*1GP3E0| |  |  |  |  |  |  |
|  |  |  |  | Annotation | 1SP0E0 | 1SP0E0 | 1SP3E0 | 1SP3E0 | 1GP3E0 |
|  |  |  |  | Evidence | 1 | 1 | 1 | 1 | 1 |
|  |  |  |  | Focus | 4 | 4 | 4 | 4 | 1 |
|  |  |  |  | Polarity | 4 | 4 | 7 | 7 | 7 |
| 340 | 6780 | 10037791\_261 | Consistent with this possibility, Jemmerson et al. (1999) have obtained evidence that mouse cytochrome c does assume a different conformation early in apoptosis of a T cell hybridoma. |A1:\*\*1SP3E3| |A2:\*\*1SP3E2| |A3:\*\*1SP3E3| |A4:\*\*1SN3E2| |A5:\*\*1SP3E2| |  |  |  |  |  |  |
|  |  |  |  | Annotation | 1SP3E3 | 1SP3E2 | 1SP3E3 | 1SN3E2 | 1SP3E2 |
|  |  |  |  | Evidence | 4 | 3 | 4 | 3 | 3 |
|  |  |  |  | Focus | 4 | 4 | 4 | 4 | 4 |
|  |  |  |  | Polarity | 7 | 7 | 7 | 1 | 7 |
| 341 | 7271 | 10588960\_98 | The introduction of these 'identifiers, and the subsequent induction of heteroduplex formation with the PCR product containing the mutation, |A5:\*\*1SP3E2| results in an increased degree of mismatch between the heteroduplex strands |A5:\*\*2SP3E2+| |A2:\*\*1SP3E1+| which is responsible for the greatly reduced mobility of heteroduplexes on a non-denaturing polyacrylamide gel  (33)(35). |A5:\*\*3SP3E2-| |A1:\*\*1SP3E2+| |A2:\*\*2SP3E2-| |A3:\*\*1SP3E2| |A4:\*\*1SP3E0| |  |  |  |  |  |  |
|  |  |  |  | Annotation | 1SP3E2+ 1SP3E2+ 1SP3E2+ | 1SP3E1+ 1SP3E1+ 2SP3E2- | 1SP3E2 1SP3E2 1SP3E2 | 1SP3E0 1SP3E0 1SP3E0 | 1SP3E2 2SP3E2+ 3SP3E2- |
|  |  |  |  | Evidence | 3 3 3 | 2 2 3 | 3 3 3 | 1 1 1 | 3 3 3 |
|  |  |  |  | Focus | 4 4 4 | 4 4 4 | 4 4 4 | 4 4 4 | 4 4 4 |
|  |  |  |  | Polarity | 7 7 7 | 7 7 7 | 7 7 7 | 7 7 7 | 7 7 7 |
| 342 | 7640 | 10421629\_23 | Wnt signaling modifies Axin. |A1:\*\*1SP3E0| |A2:\*\*1SP3E0| |A3:\*\*1SP3E0| |A4:\*\*1SP3E0| |A5:\*\*1GP3E0| |  |  |  |  |  |  |
|  |  |  |  | Annotation | 1SP3E0 | 1SP3E0 | 1SP3E0 | 1SP3E0 | 1GP3E0 |
|  |  |  |  | Evidence | 1 | 1 | 1 | 1 | 1 |
|  |  |  |  | Focus | 4 | 4 | 4 | 4 | 1 |
|  |  |  |  | Polarity | 7 | 7 | 7 | 7 | 7 |
| 343 | 431 | 11086989\_238 | Both of these steps are crucial, as evidenced by the fact that rdgC mutants and arr2(S366A) mutants |A5:\*\*1GP3E1| both undergo rapid light-dependent retinal degeneration. |A5:\*\*2GP3E0-| |A1:\*\*1SP3E0| |A2:\*\*1SP3E1| |A3:\*\*1SP2E0| |A4:\*\*1SP3E0| |  |  |  |  |  |  |
|  |  |  |  | Annotation | 1SP3E0 1SP3E0 | 1SP3E1 1SP3E1 | 1SP2E0 1SP2E0 | 1SP3E0 1SP3E0 | 1GP3E1 2GP3E0- |
|  |  |  |  | Evidence | 1 1 | 2 2 | 1 1 | 1 1 | 2 1 |
|  |  |  |  | Focus | 4 4 | 4 4 | 4 4 | 4 4 | 1 1 |
|  |  |  |  | Polarity | 7 7 | 7 7 | 6 6 | 7 7 | 7 7 |
| 344 | 2837 | 11325993\_17 | A superior marker of current or recent infection is required to clarify the clinical importance of C. pneumoniae infection in chronic diseases such as atherosclerosis. |A1:\*\*1GP3E0| |A2:\*\*1SP3E0| |A3:\*\*1SP3E0| |A4:\*\*1SP3E0| |A5:\*\*1GP3E0| |  |  |  |  |  |  |
|  |  |  |  | Annotation | 1GP3E0 | 1SP3E0 | 1SP3E0 | 1SP3E0 | 1GP3E0 |
|  |  |  |  | Evidence | 1 | 1 | 1 | 1 | 1 |
|  |  |  |  | Focus | 1 | 4 | 4 | 4 | 1 |
|  |  |  |  | Polarity | 7 | 7 | 7 | 7 | 7 |
| 345 | 5784 | 12198154\_5 | In addition, caspase-10 can not substitute for caspase-8, as the defect in apoptosis induction observed in caspase-8-deficient cells could not be rescued by overexpression of caspase-10. |A1:\*\*1SP1E0| |A2:\*\*1SN3E0| |A3:\*\*1SN3E3| |A4:\*\*1SN3E3+| |A5:\*\*1SN3E1| |  |  |  |  |  |  |
|  |  |  |  | Annotation | 1SP1E0 | 1SN3E0 | 1SN3E3 | 1SN3E3+ | 1SN3E1 |
|  |  |  |  | Evidence | 1 | 1 | 4 | 4 | 2 |
|  |  |  |  | Focus | 4 | 4 | 4 | 4 | 4 |
|  |  |  |  | Polarity | 5 | 1 | 1 | 1 | 1 |
| 346 | 7382 | 11841944\_480 | The identities of the coupled products were confirmed by electrospray mass spectrometry. |A1:\*\*1MP3E3| |A2:\*\*1SP3E3| |A3:\*\*1SP3E3| |A4:\*\*1SP3E3| |A5:\*\*1MP3E3| |  |  |  |  |  |  |
|  |  |  |  | Annotation | 1MP3E3 | 1SP3E3 | 1SP3E3 | 1SP3E3 | 1MP3E3 |
|  |  |  |  | Evidence | 4 | 4 | 4 | 4 | 4 |
|  |  |  |  | Focus | 2 | 4 | 4 | 4 | 2 |
|  |  |  |  | Polarity | 7 | 7 | 7 | 7 | 7 |
| 347 | 1128 | 10722617\_100 | After washing in TBS-0.1% Tween 20, the bound antibodies were reacted with alkaline phosphatase-conjugated secondary antibodies (1/5,000 in TBS-0.1% Tween 20-3% neonatal goat serum), and membranes were developed with the chemiluminescence substrate CDP-Star (Boehringer). |A1:\*\*1SP3E3| |A2:\*\*1MP3E3| |A3:\*\*1SP3E3| |A4:\*\*1MP3E3| |A5:\*\*1MP3E3| |  |  |  |  |  |  |
|  |  |  |  | Annotation | 1SP3E3 | 1MP3E3 | 1SP3E3 | 1MP3E3 | 1MP3E3 |
|  |  |  |  | Evidence | 4 | 4 | 4 | 4 | 4 |
|  |  |  |  | Focus | 4 | 2 | 4 | 2 | 2 |
|  |  |  |  | Polarity | 7 | 7 | 7 | 7 | 7 |
| 348 | 9234 | 10669745\_255 | In good agreement with previous results (32), |A5:\*\*1GP3E2| |A4:\*\*1SP3E2| we found that the overexpression of the wild-type protein, |A5:\*\*2GP3E3+| |A3:\*\*1SP3E2| but not of Vav (delta1-66), |A5:\*\*3GN3E3| |A3:\*\*1SN3E0| induced a high basal activity of NF-AT in nonstimulated cells (Fig. 6A). |A5:\*\*4SP3E3+| |A1:\*\*1SP3E3+| |A2:\*\*1SP3E23+| |A3:\*\*1SP3E3| |A4:\*\*2SP3E3+| |  |  |  |  |  |  |
|  |  |  |  | Annotation | 1SP3E3+ 1SP3E3+ 1SP3E3+ 1SP3E3+ | 1SP3E23+ 1SP3E23+ 1SP3E23+ 1SP3E23+ | 1SP3E2 1SP3E2 1SN3E0 1SP3E3 | 1SP3E2 2SP3E3+ 2SP3E3+ 2SP3E3+ | 1GP3E2 2GP3E3+ 3GN3E3 4SP3E3+ |
|  |  |  |  | Evidence | 4 4 4 4 | 3 3 3 3 | 3 3 1 4 | 3 4 4 4 | 3 4 4 4 |
|  |  |  |  | Focus | 4 4 4 4 | 4 4 4 4 | 4 4 4 4 | 4 4 4 4 | 1 1 1 4 |
|  |  |  |  | Polarity | 7 7 7 7 | 7 7 7 7 | 7 7 1 7 | 7 7 7 7 | 7 7 1 7 |
| 349 | 6356 | 9560277\_72 | Serial sections were collected beginning at the tissue Epon tissue-Epon interface. |A1:\*\*1GP3E3| |A2:\*\*1SP3E3| |A3:\*\*1SP3E3| |A4:\*\*1SP3E0| |A5:\*\*1GP3E3| |  |  |  |  |  |  |
|  |  |  |  | Annotation | 1GP3E3 | 1SP3E3 | 1SP3E3 | 1SP3E0 | 1GP3E3 |
|  |  |  |  | Evidence | 4 | 4 | 4 | 1 | 4 |
|  |  |  |  | Focus | 1 | 4 | 4 | 4 | 1 |
|  |  |  |  | Polarity | 7 | 7 | 7 | 7 | 7 |
| 350 | 941 | 11960809\_4 | Subjects performed blocks of randomly mixed prosaccades and antisaccades. |A1:\*\*1GP3E0| |A2:\*\*1GP3E0| |A3:\*\*1SP3E3-| |A4:\*\*1GP3E0| |A5:\*\*1GP3E3| |  |  |  |  |  |  |
|  |  |  |  | Annotation | 1GP3E0 | 1GP3E0 | 1SP3E3- | 1GP3E0 | 1GP3E3 |
|  |  |  |  | Evidence | 1 | 1 | 4 | 1 | 4 |
|  |  |  |  | Focus | 1 | 1 | 4 | 1 | 1 |
|  |  |  |  | Polarity | 7 | 7 | 7 | 7 | 7 |
| 351 | 7267 | 11069105\_48 | Phosphorylation of His6-PFK-2 did not affect FBPase-2 activity (see Supplementary material). |A1:\*\*1SN3E0| |A2:\*\*1SN3E1| |A3:\*\*1SN3E3| |A4:\*\*1SN3E3| |A5:\*\*1SN3E3| |  |  |  |  |  |  |
|  |  |  |  | Annotation | 1SN3E0 | 1SN3E1 | 1SN3E3 | 1SN3E3 | 1SN3E3 |
|  |  |  |  | Evidence | 1 | 2 | 4 | 4 | 4 |
|  |  |  |  | Focus | 4 | 4 | 4 | 4 | 4 |
|  |  |  |  | Polarity | 1 | 1 | 1 | 1 | 1 |
| 352 | 9148 | 9618506\_133 | hsREV3p does not however appear to be involved in normal DNA replication, |A3:\*\*1SN3E3| |A5:\*\*1SN3E3| because the MSU-1.2-10A-42 cells that express high levels of hsREV3 antisense RNA in the absence of tet were found to replicate at the normal rate (data not shown). |A3:\*\*2SP3E3| |A1:\*\*1SP3E1| |A2:\*\*1SN3E1| |A4:\*\*1SN2E3+| |A5:\*\*2SP3E3| |  |  |  |  |  |  |
|  |  |  |  | Annotation | 1SP3E1 1SP3E1 | 1SN3E1 1SN3E1 | 1SN3E3 2SP3E3 | 1SN2E3+ 1SN2E3+ | 1SN3E3 2SP3E3 |
|  |  |  |  | Evidence | 2 2 | 2 2 | 4 4 | 4 4 | 4 4 |
|  |  |  |  | Focus | 4 4 | 4 4 | 4 4 | 4 4 | 4 4 |
|  |  |  |  | Polarity | 7 7 | 1 1 | 1 7 | 2 2 | 1 7 |
| 353 | 2480 | 11015381\_56 | When the dogs were euthanized at the conclusion of the study, additional tissues, including blood, heart, spleen, kidneys, bladder, cerebrospinal fluid, and joints, were also examined. |A1:\*\*1GP3E3| |A2:\*\*1SP3E3| |A3:\*\*1SP3E3| |A4:\*\*1SP3E3| |A5:\*\*1GP3E3| |  |  |  |  |  |  |
|  |  |  |  | Annotation | 1GP3E3 | 1SP3E3 | 1SP3E3 | 1SP3E3 | 1GP3E3 |
|  |  |  |  | Evidence | 4 | 4 | 4 | 4 | 4 |
|  |  |  |  | Focus | 1 | 4 | 4 | 4 | 1 |
|  |  |  |  | Polarity | 7 | 7 | 7 | 7 | 7 |
| 354 | 7430 | 8626420\_59 | Total activated p85/PI 3-K activity = total activated PI 3-K activity - total activated PI 3-K activity. |A1:\*\*1SP3E0| |A2:\*\*1SP3E0| |A3:\*\*1SP3E3| |A4:\*\*1SP3E3| |A5:\*\*1GP3E0| |  |  |  |  |  |  |
|  |  |  |  | Annotation | 1SP3E0 | 1SP3E0 | 1SP3E3 | 1SP3E3 | 1GP3E0 |
|  |  |  |  | Evidence | 1 | 1 | 4 | 4 | 1 |
|  |  |  |  | Focus | 4 | 4 | 4 | 4 | 1 |
|  |  |  |  | Polarity | 7 | 7 | 7 | 7 | 7 |
| 355 | 5217 | 10618080\_136 | An incidental finding with potential usefulness was also noted during the course of this work. |A1:\*\*1GP3E3| |A2:\*\*1SP3E3| |A3:\*\*1SP3E3| |A4:\*\*1SP2E3| |A5:\*\*1GP2E3| |  |  |  |  |  |  |
|  |  |  |  | Annotation | 1GP3E3 | 1SP3E3 | 1SP3E3 | 1SP2E3 | 1GP2E3 |
|  |  |  |  | Evidence | 4 | 4 | 4 | 4 | 4 |
|  |  |  |  | Focus | 1 | 4 | 4 | 4 | 1 |
|  |  |  |  | Polarity | 7 | 7 | 7 | 6 | 6 |
| 356 | 5378 | 11902611\_8 | Five subjects showed enamel demineralization on a total of 30 teeth at the time of de-bonding. |A1:\*\*1SP3E3| |A2:\*\*1SP3E3| |A3:\*\*1SP3E3| |A4:\*\*1GP3E0| |A5:\*\*1GP3E3-| |  |  |  |  |  |  |
|  |  |  |  | Annotation | 1SP3E3 | 1SP3E3 | 1SP3E3 | 1GP3E0 | 1GP3E3- |
|  |  |  |  | Evidence | 4 | 4 | 4 | 1 | 4 |
|  |  |  |  | Focus | 4 | 4 | 4 | 1 | 1 |
|  |  |  |  | Polarity | 7 | 7 | 7 | 7 | 7 |
| 357 | 1854 | 11102376\_354 | This is consistent with the kinase activity of Trc being functionally important for bristle morphogenesis. |A1:\*\*1SP3E0| |A2:\*\*1SP3E1| |A3:\*\*1SP3E0| |A4:\*\*1SP3E0| |A5:\*\*1SP3E0| |  |  |  |  |  |  |
|  |  |  |  | Annotation | 1SP3E0 | 1SP3E1 | 1SP3E0 | 1SP3E0 | 1SP3E0 |
|  |  |  |  | Evidence | 1 | 2 | 1 | 1 | 1 |
|  |  |  |  | Focus | 4 | 4 | 4 | 4 | 4 |
|  |  |  |  | Polarity | 7 | 7 | 7 | 7 | 7 |
| 358 | 226 | 12183451\_12 | Unlike most conventional chemotherapeutic drugs, ALP does not target the DNA but acts at the level of cell membranes. |A1:\*\*1SN3E0| |A2:\*\*1SN3E0| |A3:\*\*1SN3E0| |A4:\*\*1SN3E0| |A5:\*\*1GN3E0| |  |  |  |  |  |  |
|  |  |  |  | Annotation | 1SN3E0 | 1SN3E0 | 1SN3E0 | 1SN3E0 | 1GN3E0 |
|  |  |  |  | Evidence | 1 | 1 | 1 | 1 | 1 |
|  |  |  |  | Focus | 4 | 4 | 4 | 4 | 1 |
|  |  |  |  | Polarity | 1 | 1 | 1 | 1 | 1 |
| 359 | 4586 | 7608173\_59 | However, the cell surface expression of transferrin receptor was reduced by 44%. |A1:\*\*1SP3E3| |A2:\*\*1SP3E3-| |A3:\*\*1SP3E3| |A4:\*\*1SP3E0| |A5:\*\*1GP3E3-| |  |  |  |  |  |  |
|  |  |  |  | Annotation | 1SP3E3 | 1SP3E3- | 1SP3E3 | 1SP3E0 | 1GP3E3- |
|  |  |  |  | Evidence | 4 | 4 | 4 | 1 | 4 |
|  |  |  |  | Focus | 4 | 4 | 4 | 4 | 1 |
|  |  |  |  | Polarity | 7 | 7 | 7 | 7 | 7 |
| 360 | 7990 | 11894887\_3 | Until the late 1960s, developmental texts reassured parents and teachers that homosexual behavior among adolescents was transitory and quite normal. |A1:\*\*1GP3E0| |A2:\*\*1GP3E1| |A3:\*\*1SP3E0| |A4:\*\*1GP3E0| |A5:\*\*1GP3E1| |  |  |  |  |  |  |
|  |  |  |  | Annotation | 1GP3E0 | 1GP3E1 | 1SP3E0 | 1GP3E0 | 1GP3E1 |
|  |  |  |  | Evidence | 1 | 2 | 1 | 1 | 2 |
|  |  |  |  | Focus | 1 | 1 | 4 | 1 | 1 |
|  |  |  |  | Polarity | 7 | 7 | 7 | 7 | 7 |
| 361 | 9683 | 11905996\_7 | These data indicate that mechanisms relating to AP-1 transcription factor complexes underlying nerve growth factor-mediated enhancement of cholinergic gene expression may differ from those required for morphological differentiation. |A1:\*\*1SP1E3| |A2:\*\*1SP2E3| |A3:\*\*1SP1E3| |A4:\*\*1SP3E3| |A5:\*\*1SP3E1| |  |  |  |  |  |  |
|  |  |  |  | Annotation | 1SP1E3 | 1SP2E3 | 1SP1E3 | 1SP3E3 | 1SP3E1 |
|  |  |  |  | Evidence | 4 | 4 | 4 | 4 | 2 |
|  |  |  |  | Focus | 4 | 4 | 4 | 4 | 4 |
|  |  |  |  | Polarity | 5 | 6 | 5 | 7 | 7 |
| 362 | 61 | 11326002\_39 | Protease sequences have been submitted to the GenBank database with the accession numbers AF247007 to AF247038. |A1:\*\*1GP3E1| |A2:\*\*1SP3E3| |A3:\*\*1SP3E3| |A4:\*\*1SP3E3| |A5:\*\*1GP3E3| |  |  |  |  |  |  |
|  |  |  |  | Annotation | 1GP3E1 | 1SP3E3 | 1SP3E3 | 1SP3E3 | 1GP3E3 |
|  |  |  |  | Evidence | 2 | 4 | 4 | 4 | 4 |
|  |  |  |  | Focus | 1 | 4 | 4 | 4 | 1 |
|  |  |  |  | Polarity | 7 | 7 | 7 | 7 | 7 |
| 363 | 3460 | 12048123\_4 | ApoE\*3 Leiden mice developed marked hypercholesterolaemia, and early Type I 'fatty streak' lesions, |A1:\*\*1SP3E3| following consumption of an atherogenic diet high in saturated fat and cholesterol, and containing sodium cholate, for up to 4 weeks. |A1:\*\*2SP3E3| |A2:\*\*1SP3E3| |A3:\*\*1SP3E3| |A4:\*\*1SP3E3| |A5:\*\*1SMP3E3| |  |  |  |  |  |  |
|  |  |  |  | Annotation | 1SP3E3 2SP3E3 | 1SP3E3 1SP3E3 | 1SP3E3 1SP3E3 | 1SP3E3 1SP3E3 | 1SMP3E3 1SMP3E3 |
|  |  |  |  | Evidence | 4 4 | 4 4 | 4 4 | 4 4 | 4 4 |
|  |  |  |  | Focus | 4 4 | 4 4 | 4 4 | 4 4 | 6 6 |
|  |  |  |  | Polarity | 7 7 | 7 7 | 7 7 | 7 7 | 7 7 |
| 364 | 2862 | 9614070\_287 | FPLC data demonstrated that the reaction of cisplatin with rHA (1:1, 1 mM) |A5:\*\*1SP3E3| was accompanied by a decrease in the amount of monomeric albumin to an average of 58%, |A2:\*\*1SP3E3-| |A1:\*\*1SP3E3-| |A4:\*\*1MP3E3-| |A5:\*\*2SP3E3-| by an increase of dimer content, |A2:\*\*1SP3E0+| and appearance of higher molecular mass polymers (Fig. 7 B). |A2:\*\*1SP3E3| |A1:\*\*2SP3E3+| |A3:\*\*1SP3E3| |A4:\*\*2SP3E3+| |A5:\*\*3SP3E3+| |  |  |  |  |  |  |
|  |  |  |  | Annotation | 1SP3E3- 1SP3E3- 2SP3E3+ 2SP3E3+ | 1SP3E3- 1SP3E3- 1SP3E0+ 1SP3E3 | 1SP3E3 1SP3E3 1SP3E3 1SP3E3 | 1MP3E3- 1MP3E3- 2SP3E3+ 2SP3E3+ | 1SP3E3 2SP3E3- 3SP3E3+ 3SP3E3+ |
|  |  |  |  | Evidence | 4 4 4 4 | 4 4 1 4 | 4 4 4 4 | 4 4 4 4 | 4 4 4 4 |
|  |  |  |  | Focus | 4 4 4 4 | 4 4 4 4 | 4 4 4 4 | 2 2 4 4 | 4 4 4 4 |
|  |  |  |  | Polarity | 7 7 7 7 | 7 7 7 7 | 7 7 7 7 | 7 7 7 7 | 7 7 7 7 |
| 365 | 7493 | 10671457\_171 | At least 19 cellulosomal polypeptides have been cloned and sequenced (2). |A1:\*\*1SP3E2| |A2:\*\*1SP3E2| |A3:\*\*1SP2E2| |A4:\*\*1SP3E2| |A5:\*\*1GP3E2| |  |  |  |  |  |  |
|  |  |  |  | Annotation | 1SP3E2 | 1SP3E2 | 1SP2E2 | 1SP3E2 | 1GP3E2 |
|  |  |  |  | Evidence | 3 | 3 | 3 | 3 | 3 |
|  |  |  |  | Focus | 4 | 4 | 4 | 4 | 1 |
|  |  |  |  | Polarity | 7 | 7 | 6 | 7 | 7 |
| 366 | 2433 | 9835626\_215 | The ability of TIMP-1 to inhibit apoptosis in an MMP-independent manner is consistent with |A5:\*\*1SP3E2| observations of growth promotion by TIMPs in a variety of cell types ( 13). |A1:\*\*1SP3E2| |A2:\*\*1SP3E2| |A3:\*\*1SP3E2| |A4:\*\*1SP3E2-| |A5:\*\*2SP3E2+| |  |  |  |  |  |  |
|  |  |  |  | Annotation | 1SP3E2 1SP3E2 | 1SP3E2 1SP3E2 | 1SP3E2 1SP3E2 | 1SP3E2- 1SP3E2- | 1SP3E2 2SP3E2+ |
|  |  |  |  | Evidence | 3 3 | 3 3 | 3 3 | 3 3 | 3 3 |
|  |  |  |  | Focus | 4 4 | 4 4 | 4 4 | 4 4 | 4 4 |
|  |  |  |  | Polarity | 7 7 | 7 7 | 7 7 | 7 7 | 7 7 |
| 367 | 5919 | 11038179\_17 | One current model for nuclear transport is that nuclear carriers move through the NPC by repeated association - dissociation reactions with NPC proteins, a process that has been called "`facilitated diffusion"` diffusion" |A5:\*\*1SP3E2| and that appears not to require an energy source (Kose et al. 1997 ; Ribbeck et al. 1998 ; Schwoebel et al. 1998 ; Englmeier et al. 1999 ; Talcott and Moore 1999 ). |A5:\*\*2SN3E2| |A1:\*\*1SP3E2| |A2:\*\*1SP3E2| |A3:\*\*1SP3E2| |A4:\*\*1SP3E2| |  |  |  |  |  |  |
|  |  |  |  | Annotation | 1SP3E2 1SP3E2 | 1SP3E2 1SP3E2 | 1SP3E2 1SP3E2 | 1SP3E2 1SP3E2 | 1SP3E2 2SN3E2 |
|  |  |  |  | Evidence | 3 3 | 3 3 | 3 3 | 3 3 | 3 3 |
|  |  |  |  | Focus | 4 4 | 4 4 | 4 4 | 4 4 | 4 4 |
|  |  |  |  | Polarity | 7 7 | 7 7 | 7 7 | 7 7 | 7 1 |
| 368 | 5074 | 12361201\_4 | Serum levels of tissue type plasminogen activator (tPA) activity, plasminogen activator inhibitor-I (PAI-1) activity, tPA antigen, PAI-I antigen, FVII, FX, and fibrinogen were assayed after both treatments. |A1:\*\*1SP3E3| |A2:\*\*1SP3E3| |A3:\*\*1SP3E3| |A4:\*\*1SP3E3| |A5:\*\*1MP3E3| |  |  |  |  |  |  |
|  |  |  |  | Annotation | 1SP3E3 | 1SP3E3 | 1SP3E3 | 1SP3E3 | 1MP3E3 |
|  |  |  |  | Evidence | 4 | 4 | 4 | 4 | 4 |
|  |  |  |  | Focus | 4 | 4 | 4 | 4 | 2 |
|  |  |  |  | Polarity | 7 | 7 | 7 | 7 | 7 |
| 369 | 2518 | 12359347\_1 | Bromodichloromethane (BDCM) and bromoform (TBM) have been demonstrated to be colon carcinogens in male and female F344/N rats following administration by corn oil gavage. |A1:\*\*1SP3E1| |A2:\*\*1SP3E1| |A3:\*\*1SP3E3| |A4:\*\*1SP3E3| |A5:\*\*1MP3E1| |  |  |  |  |  |  |
|  |  |  |  | Annotation | 1SP3E1 | 1SP3E1 | 1SP3E3 | 1SP3E3 | 1MP3E1 |
|  |  |  |  | Evidence | 2 | 2 | 4 | 4 | 2 |
|  |  |  |  | Focus | 4 | 4 | 4 | 4 | 2 |
|  |  |  |  | Polarity | 7 | 7 | 7 | 7 | 7 |
| 370 | 6819 | 9580622\_172 | Despite clear evidence that mibefradil is effective in the treatment of hypertension and stable angina pectoris and the fact that it is devoid of negative inotropic effects, |A5:\*\*1SP3E1| results of a limited number of studies to date that have examined this compound in the setting of heart failure are somewhat inconsistent. |A5:\*\*2SP1E1| |A1:\*\*1GP2E1| |A2:\*\*1SP1E1| |A3:\*\*1MP1E1| |A4:\*\*1MP1E1| |  |  |  |  |  |  |
|  |  |  |  | Annotation | 1GP2E1 1GP2E1 | 1SP1E1 1SP1E1 | 1MP1E1 1MP1E1 | 1MP1E1 1MP1E1 | 1SP3E1 2SP1E1 |
|  |  |  |  | Evidence | 2 2 | 2 2 | 2 2 | 2 2 | 2 2 |
|  |  |  |  | Focus | 1 1 | 4 4 | 2 2 | 2 2 | 4 4 |
|  |  |  |  | Polarity | 6 6 | 5 5 | 5 5 | 5 5 | 7 5 |
| 371 | 9061 | 10747966\_58 | Ba2+ is distinguished from angiotensin II and DMPP by not stimulating phospholipase C activity |A2:\*\*1SP3E0-| |A3:\*\*1SN3E0| |A5:\*\*1SN3E2| and an increase in Ins(1,4,5)P3 in chromaffin cells ( , ) |A2:\*\*2SP3E2+| |A1:\*\*1SP3E2+| |A4:\*\*1SN3E3+| |A5:\*\*2SP3E2+| In summary, the characteristics of the rapid movement of PH-GFP to and from the plasma membrane upon stimulation of the cells supports the conclusion that the probe binds to the plasma membrane pool of PtdIns-4,5-P2. |A2:\*\*3SP3E1| |A1:\*\*2SP3E1| |A3:\*\*2SP3E3| |A4:\*\*2SP3E3| |A5:\*\*3SP3E1| |  |  |  |  |  |  |
|  |  |  |  | Annotation | 1SP3E2+ 1SP3E2+ 2SP3E1 | 1SP3E0- 2SP3E2+ 3SP3E1 | 1SN3E0 2SP3E3 2SP3E3 | 1SN3E3+ 1SN3E3+ 2SP3E3 | 1SN3E2 2SP3E2+ 3SP3E1 |
|  |  |  |  | Evidence | 3 3 2 | 1 3 2 | 1 4 4 | 4 4 4 | 3 3 2 |
|  |  |  |  | Focus | 4 4 4 | 4 4 4 | 4 4 4 | 4 4 4 | 4 4 4 |
|  |  |  |  | Polarity | 7 7 7 | 7 7 7 | 1 7 7 | 1 1 7 | 1 7 7 |
| 372 | 1317 | 9390512\_151 | Another design, the Transmission Disequilibrium Test (TDT; Terwilliger and Ott 1992 ; Spielman et al. 1993 ), |A4:\*\*1SP3E2| tests the transmission of a particular allele from a parent to the affected individual using the other untransmitted allele from the parent as the control. |A4:\*\*2SP3E0| |A1:\*\*1MP3E2| |A2:\*\*1SP3E2| |A3:\*\*1SP3E0| |A5:\*\*1MP3E2| |  |  |  |  |  |  |
|  |  |  |  | Annotation | 1MP3E2 1MP3E2 | 1SP3E2 1SP3E2 | 1SP3E0 1SP3E0 | 1SP3E2 2SP3E0 | 1MP3E2 1MP3E2 |
|  |  |  |  | Evidence | 3 3 | 3 3 | 1 1 | 3 1 | 3 3 |
|  |  |  |  | Focus | 2 2 | 4 4 | 4 4 | 4 4 | 2 2 |
|  |  |  |  | Polarity | 7 7 | 7 7 | 7 7 | 7 7 | 7 7 |
| 373 | 5525 | 9768758\_281 | In the current model, 79.8% of the H2-M residues have highly favored angles, while 1.6% (5 residues) have disallowed angles. |A1:\*\*1SP3E3| |A2:\*\*1SP3E3| |A3:\*\*1SP3E3| |A4:\*\*1SP3E3| |A5:\*\*1GP3E3| |  |  |  |  |  |  |
|  |  |  |  | Annotation | 1SP3E3 | 1SP3E3 | 1SP3E3 | 1SP3E3 | 1GP3E3 |
|  |  |  |  | Evidence | 4 | 4 | 4 | 4 | 4 |
|  |  |  |  | Focus | 4 | 4 | 4 | 4 | 1 |
|  |  |  |  | Polarity | 7 | 7 | 7 | 7 | 7 |
| 374 | 8892 | 12136945\_6 | The presented results suggest that urine neopterin levels are related to the activation of T cells in malignant lymphoma. |A1:\*\*1SP3E3| |A2:\*\*1SP3E3+| |A3:\*\*1SP2E3| |A4:\*\*1SP2E1| |A5:\*\*1GP2E3| |  |  |  |  |  |  |
|  |  |  |  | Annotation | 1SP3E3 | 1SP3E3+ | 1SP2E3 | 1SP2E1 | 1GP2E3 |
|  |  |  |  | Evidence | 4 | 4 | 4 | 2 | 4 |
|  |  |  |  | Focus | 4 | 4 | 4 | 4 | 1 |
|  |  |  |  | Polarity | 7 | 7 | 6 | 6 | 6 |
| 375 | 1362 | 9573199\_301 | Results reported in the early literature of Middlebrook and Cohn suggest that INH resistance in M. tuberculosis can occur via metabolic defects that may be similar to the ndh defects found in M. smegmatis (26). |A1:\*\*1SP1E2| |A2:\*\*1SP2E2| |A3:\*\*1SP1E2| |A4:\*\*1SP3E2| |A5:\*\*1SP3E2| |  |  |  |  |  |  |
|  |  |  |  | Annotation | 1SP1E2 | 1SP2E2 | 1SP1E2 | 1SP3E2 | 1SP3E2 |
|  |  |  |  | Evidence | 3 | 3 | 3 | 3 | 3 |
|  |  |  |  | Focus | 4 | 4 | 4 | 4 | 4 |
|  |  |  |  | Polarity | 5 | 6 | 5 | 7 | 7 |
| 376 | 1763 | 11371351\_82 | Activation by some mammalian HSPs may even involve TLRs (Ohashi et al., 2000). |A1:\*\*1SP1E2| |A2:\*\*1SP2E2+| |A3:\*\*1SP1E2| |A4:\*\*1SP2E2| |A5:\*\*1GP2E2| |  |  |  |  |  |  |
|  |  |  |  | Annotation | 1SP1E2 | 1SP2E2+ | 1SP1E2 | 1SP2E2 | 1GP2E2 |
|  |  |  |  | Evidence | 3 | 3 | 3 | 3 | 3 |
|  |  |  |  | Focus | 4 | 4 | 4 | 4 | 1 |
|  |  |  |  | Polarity | 5 | 6 | 5 | 6 | 6 |
| 377 | 3414 | 9742132\_89 | Transcripts of the appropriate length were eluted from polyacrylamide urea gel slices as previously described (33). |A1:\*\*1SP3E23| |A2:\*\*1MP3E23| |A3:\*\*1SP3E3| |A4:\*\*1MP3E2| |A5:\*\*1MP3E2| |  |  |  |  |  |  |
|  |  |  |  | Annotation | 1SP3E23 | 1MP3E23 | 1SP3E3 | 1MP3E2 | 1MP3E2 |
|  |  |  |  | Evidence | 3 | 3 | 4 | 3 | 3 |
|  |  |  |  | Focus | 4 | 2 | 4 | 2 | 2 |
|  |  |  |  | Polarity | 7 | 7 | 7 | 7 | 7 |
| 378 | 3740 | 11591709\_31 | Whether CysLT2R might also couple to the Gi/o class in certain tissues in unknown. |A1:\*\*1SP0E0| |A2:\*\*1SN3E0| |A3:\*\*1SP1E0| |A4:\*\*1SP0E0| |A5:\*\*1GP0E0| |  |  |  |  |  |  |
|  |  |  |  | Annotation | 1SP0E0 | 1SN3E0 | 1SP1E0 | 1SP0E0 | 1GP0E0 |
|  |  |  |  | Evidence | 1 | 1 | 1 | 1 | 1 |
|  |  |  |  | Focus | 4 | 4 | 4 | 4 | 1 |
|  |  |  |  | Polarity | 4 | 1 | 5 | 4 | 4 |
| 379 | 1303 | 9560277\_19 | Consequently, alterations in chandelier neurons in schizophrenia could account, at least in part, for the functional abnormalities observed in the PFC and interconnected brain regions in schizophrenia. |A1:\*\*1SP2E0| |A2:\*\*1SP2E0| |A3:\*\*1SP2E0| |A4:\*\*1SP2E0| |A5:\*\*1GP3E0| |  |  |  |  |  |  |
|  |  |  |  | Annotation | 1SP2E0 | 1SP2E0 | 1SP2E0 | 1SP2E0 | 1GP3E0 |
|  |  |  |  | Evidence | 1 | 1 | 1 | 1 | 1 |
|  |  |  |  | Focus | 4 | 4 | 4 | 4 | 1 |
|  |  |  |  | Polarity | 6 | 6 | 6 | 6 | 7 |
| 380 | 9284 | 10983980\_13 | These cell type-specific proteins bind to pre-mRNAs at discrete sites and activate (Tra) |A5:\*\*1SP3E2+| |A2:\*\*1SP3E1+| or repress (PSI and Sxl) splice sites |A5:\*\*2SP3E-| by either recruiting splicing factors to the proper splice site (Lynch and Maniatis 1996 ) |A5:\*\*3SP3E2| or blocking basal splicing factors from selecting the default splice site ( Siebel et al. 1992 ; Valcarcel et al. 1993 ). |A5:\*\*4SP3E2-| |A1:\*\*1SP3E2| |A2:\*\*2SP3E2-| |A3:\*\*1SP3E2| |A4:\*\*1SP3E2| |  |  |  |  |  |  |
|  |  |  |  | Annotation | 1SP3E2 1SP3E2 1SP3E2 1SP3E2 | 1SP3E1+ 2SP3E2- 2SP3E2- 2SP3E2- | 1SP3E2 1SP3E2 1SP3E2 1SP3E2 | 1SP3E2 1SP3E2 1SP3E2 1SP3E2 | 1SP3E2+ 3SP3E2 3SP3E2 4SP3E2- |
|  |  |  |  | Evidence | 3 3 3 3 | 2 3 3 3 | 3 3 3 3 | 3 3 3 3 | 3 3 3 3 |
|  |  |  |  | Focus | 4 4 4 4 | 4 4 4 4 | 4 4 4 4 | 4 4 4 4 | 4 4 4 4 |
|  |  |  |  | Polarity | 7 7 7 7 | 7 7 7 7 | 7 7 7 7 | 7 7 7 7 | 7 7 7 7 |
| 381 | 3318 | 7673177\_42 | Our results show that cAMP in combination with insulin stimulates the activity and phosphorylation of p70 |A5:\*\*1SP3E3+| as demonstrated by immune complex kinase and mobility shift assays. |A5:\*\*2MP3E3| |A1:\*\*1MSP3E3+| |A2:\*\*1SP3E3+| |A3:\*\*1MSP3E3| |A4:\*\*1SP3E3+| |  |  |  |  |  |  |
|  |  |  |  | Annotation | 1MSP3E3+ 1MSP3E3+ | 1SP3E3+ 1SP3E3+ | 1MSP3E3 1MSP3E3 | 1SP3E3+ 1SP3E3+ | 1SP3E3+ 2MP3E3 |
|  |  |  |  | Evidence | 4 4 | 4 4 | 4 4 | 4 4 | 4 4 |
|  |  |  |  | Focus | 6 6 | 4 4 | 6 6 | 4 4 | 4 2 |
|  |  |  |  | Polarity | 7 7 | 7 7 | 7 7 | 7 7 | 7 7 |
| 382 | 8724 | 10523671\_278 | The resulting GSTG4ADp-Gal80p complex was washed and subsequently incubated in the presence or absence of galactose and ATP at 4 degrees C for 2 h with whole-cell extract of Gly-Lac-grown cells of Sc817 (gal4delta gal80delta gal3delta) or Sc817 carrying pMEGA3-delta4delta80 ( GAL3). |A1:\*\*1MP3E3| |A2:\*\*1MP3E3| |A3:\*\*1MP3E3| |A4:\*\*1MP3E3| |A5:\*\*1MP3E3| |  |  |  |  |  |  |
|  |  |  |  | Annotation | 1MP3E3 | 1MP3E3 | 1MP3E3 | 1MP3E3 | 1MP3E3 |
|  |  |  |  | Evidence | 4 | 4 | 4 | 4 | 4 |
|  |  |  |  | Focus | 2 | 2 | 2 | 2 | 2 |
|  |  |  |  | Polarity | 7 | 7 | 7 | 7 | 7 |
| 383 | 8187 | 9880523\_156 | The peak of p38 MAKP activity was increased by 3.1 plus-or-minus 0.5- and 3.8 plus-or-minus 0.4-fold over the basal level in the presence of 100 and 300 ng/ml TL1, respectively. |A1:\*\*1SP3E3+| |A2:\*\*1SP3E3| |A3:\*\*1SP3E3| |A4:\*\*1SP3E3+| |A5:\*\*1MP3E3+| |  |  |  |  |  |  |
|  |  |  |  | Annotation | 1SP3E3+ | 1SP3E3 | 1SP3E3 | 1SP3E3+ | 1MP3E3+ |
|  |  |  |  | Evidence | 4 | 4 | 4 | 4 | 4 |
|  |  |  |  | Focus | 4 | 4 | 4 | 4 | 2 |
|  |  |  |  | Polarity | 7 | 7 | 7 | 7 | 7 |
| 384 | 3898 | 9576760\_121 | The recovered IgG/+ANX fraction accounted for 2-6% of the total IgG from patients, but less than 0.01% of that from the controls. |A1:\*\*1SP3E3| |A2:\*\*1SP3E3| |A3:\*\*1SP2E3| |A4:\*\*1SP3E3-| |A5:\*\*1GP3E3| |  |  |  |  |  |  |
|  |  |  |  | Annotation | 1SP3E3 | 1SP3E3 | 1SP2E3 | 1SP3E3- | 1GP3E3 |
|  |  |  |  | Evidence | 4 | 4 | 4 | 4 | 4 |
|  |  |  |  | Focus | 4 | 4 | 4 | 4 | 1 |
|  |  |  |  | Polarity | 7 | 7 | 6 | 7 | 7 |
| 385 | 2202 | 11573002\_16 | The causes of schizophrenia are not known, |A1:\*\*1GN0E0| |A3:\*\*1SN3E0| |A5:\*\*1GN3E2| but it is increasingly considered a neurodevelopmental disorder ( 2, 3). |A1:\*\*2GP3E2| |A2:\*\*1SN3E2| |A3:\*\*2SP3E2| |A4:\*\*1SN3E2+| |A5:\*\*2GP2E2| |  |  |  |  |  |  |
|  |  |  |  | Annotation | 1GN0E0 2GP3E2 | 1SN3E2 1SN3E2 | 1SN3E0 2SP3E2 | 1SN3E2+ 1SN3E2+ | 1GN3E2 2GP2E2 |
|  |  |  |  | Evidence | 1 3 | 3 3 | 1 3 | 3 3 | 3 3 |
|  |  |  |  | Focus | 1 1 | 4 4 | 4 4 | 4 4 | 1 1 |
|  |  |  |  | Polarity | 4 7 | 1 1 | 1 7 | 1 1 | 1 6 |
| 386 | 7856 | 12208047\_8 | Expression of all five feIFN-alpha subtypes in Chinese hamster ovary (CHO) cells was confirmed by Western blot analysis, |A1:\*\*1MP3E3| and all resulting proteins were glycosylated. |A1:\*\*2MP3E3| |A2:\*\*1MP3E3| |A3:\*\*1SP3E3| |A4:\*\*1SP3E3| |A5:\*\*1MP3E3| |  |  |  |  |  |  |
|  |  |  |  | Annotation | 1MP3E3 2MP3E3 | 1MP3E3 1MP3E3 | 1SP3E3 1SP3E3 | 1SP3E3 1SP3E3 | 1MP3E3 1MP3E3 |
|  |  |  |  | Evidence | 4 4 | 4 4 | 4 4 | 4 4 | 4 4 |
|  |  |  |  | Focus | 2 2 | 2 2 | 4 4 | 4 4 | 2 2 |
|  |  |  |  | Polarity | 7 7 | 7 7 | 7 7 | 7 7 | 7 7 |
| 387 | 628 | 9684747\_154 | When 18:1 c was present at sn -2, both enzymes derived >95% of acyl groups from sn -2. |A1:\*\*1SP3E3| |A2:\*\*1SP3E3| |A3:\*\*1SP3E3| |A4:\*\*1SP3E3| |A5:\*\*1GP3E0| |  |  |  |  |  |  |
|  |  |  |  | Annotation | 1SP3E3 | 1SP3E3 | 1SP3E3 | 1SP3E3 | 1GP3E0 |
|  |  |  |  | Evidence | 4 | 4 | 4 | 4 | 1 |
|  |  |  |  | Focus | 4 | 4 | 4 | 4 | 1 |
|  |  |  |  | Polarity | 7 | 7 | 7 | 7 | 7 |
| 388 | 4241 | 12460988\_197 | In this paper, we demonstrate that the G-domain of atToc159 mediates and likely regulates chloroplast targeting by GTP binding and hydrolysis. |A1:\*\*1SP3E3| |A2:\*\*1SP3E3| |A3:\*\*1SP2E3| |A4:\*\*1SP2E3| |A5:\*\*1GP3E3| |  |  |  |  |  |  |
|  |  |  |  | Annotation | 1SP3E3 | 1SP3E3 | 1SP2E3 | 1SP2E3 | 1GP3E3 |
|  |  |  |  | Evidence | 4 | 4 | 4 | 4 | 4 |
|  |  |  |  | Focus | 4 | 4 | 4 | 4 | 1 |
|  |  |  |  | Polarity | 7 | 7 | 6 | 6 | 7 |
| 389 | 8132 | 9230313\_89 | In addition, a cDNA clone representing a chicken homolog was isolated, and its predicted amino acid sequence was 66% identical to mAxin (Figure 3a). |A1:\*\*1SP3E3| |A2:\*\*1SP3E3| |A3:\*\*1MSP3E3| |A4:\*\*1SP2E3| |A5:\*\*1GP3E3| |  |  |  |  |  |  |
|  |  |  |  | Annotation | 1SP3E3 | 1SP3E3 | 1MSP3E3 | 1SP2E3 | 1GP3E3 |
|  |  |  |  | Evidence | 4 | 4 | 4 | 4 | 4 |
|  |  |  |  | Focus | 4 | 4 | 6 | 4 | 1 |
|  |  |  |  | Polarity | 7 | 7 | 7 | 6 | 7 |
| 390 | 1700 | 9716414\_73 | Development of maxillary incisors and all mandibular teeth was found to be initiated |A5:\*\*1GP3E3+| but arrested at the bud stage, |A5:\*\*2SP3E3-| whereas maxillary molar tooth germs were present at the correct stage in all of the embryos examined (Fig. 3). |A5:\*\*3SP3E3| |A1:\*\*1SP3E3| |A2:\*\*1SP3E3| |A3:\*\*1SP3E3| |A4:\*\*1SP3E3| |  |  |  |  |  |  |
|  |  |  |  | Annotation | 1SP3E3 1SP3E3 1SP3E3 | 1SP3E3 1SP3E3 1SP3E3 | 1SP3E3 1SP3E3 1SP3E3 | 1SP3E3 1SP3E3 1SP3E3 | 1GP3E3+ 2SP3E3- 3SP3E3 |
|  |  |  |  | Evidence | 4 4 4 | 4 4 4 | 4 4 4 | 4 4 4 | 4 4 4 |
|  |  |  |  | Focus | 4 4 4 | 4 4 4 | 4 4 4 | 4 4 4 | 1 4 4 |
|  |  |  |  | Polarity | 7 7 7 | 7 7 7 | 7 7 7 | 7 7 7 | 7 7 7 |
| 391 | 4128 | 9797273\_67 | Immulon II microplates (Dynatech Laboratories, McLean, Va.) were coated with 100 ng of phage Q38. |A1:\*\*1SP3E3| |A2:\*\*1SP3E3| |A3:\*\*1SP3E3| |A4:\*\*1SP3E3| |A5:\*\*1MP3E3| |  |  |  |  |  |  |
|  |  |  |  | Annotation | 1SP3E3 | 1SP3E3 | 1SP3E3 | 1SP3E3 | 1MP3E3 |
|  |  |  |  | Evidence | 4 | 4 | 4 | 4 | 4 |
|  |  |  |  | Focus | 4 | 4 | 4 | 4 | 2 |
|  |  |  |  | Polarity | 7 | 7 | 7 | 7 | 7 |
| 392 | 7366 | 11483515\_259 | The PP2A AC heterodimer dephosphorylates APC and axin in vitro, |A2:\*\*1SP3E0-| |A3:\*\*1SP3E0| |A5:\*\*1SP3E2-| and these findings also led to the suggestion that PP2A could activate Wnt signaling ( Willert et al., 1999; Ikeda et al., 2000). |A1:\*\*1SP3E2| |A2:\*\*2SP2E2+| |A3:\*\*2SP2E2| |A4:\*\*1SP2E2| |A5:\*\*2SP2E2| |  |  |  |  |  |  |
|  |  |  |  | Annotation | 1SP3E2 1SP3E2 | 1SP3E0- 2SP2E2+ | 1SP3E0 2SP2E2 | 1SP2E2 1SP2E2 | 1SP3E2- 2SP2E2 |
|  |  |  |  | Evidence | 3 3 | 1 3 | 1 3 | 3 3 | 3 3 |
|  |  |  |  | Focus | 4 4 | 4 4 | 4 4 | 4 4 | 4 4 |
|  |  |  |  | Polarity | 7 7 | 7 6 | 7 6 | 6 6 | 7 6 |
| 393 | 7 | 9230313\_188 | The observation that the major Axin mRNA is disrupted in two different alleles |A5:\*\*1SP3E1-| that cause axial duplications |A5:\*\*2SP3E0+| suggested that the normal gene product plays a negative regulatory role at some step in axis formation. |A5:\*\*3SP2E0| |A1:\*\*1SP3E1| |A2:\*\*1SP3E1| |A3:\*\*1SP3E3| |A4:\*\*1SP3E3-| |  |  |  |  |  |  |
|  |  |  |  | Annotation | 1SP3E1 1SP3E1 1SP3E1 | 1SP3E1 1SP3E1 1SP3E1 | 1SP3E3 1SP3E3 1SP3E3 | 1SP3E3- 1SP3E3- 1SP3E3- | 1SP3E1- 2SP3E0+ 3SP2E0 |
|  |  |  |  | Evidence | 2 2 2 | 2 2 2 | 4 4 4 | 4 4 4 | 2 1 1 |
|  |  |  |  | Focus | 4 4 4 | 4 4 4 | 4 4 4 | 4 4 4 | 4 4 4 |
|  |  |  |  | Polarity | 7 7 7 | 7 7 7 | 7 7 7 | 7 7 7 | 7 7 6 |
| 394 | 453 | 10958697\_328 | At 24 h after transfection, the confluent cultures were either incubated in GM for 1 further day (GM) or shifted to DM for 1 day (DM1) or 2 days (DM2). |A1:\*\*1SP3E3| |A2:\*\*1SP3E3| |A3:\*\*1MP3E3| |A4:\*\*1MP3E3| |A5:\*\*1MP3E3| |  |  |  |  |  |  |
|  |  |  |  | Annotation | 1SP3E3 | 1SP3E3 | 1MP3E3 | 1MP3E3 | 1MP3E3 |
|  |  |  |  | Evidence | 4 | 4 | 4 | 4 | 4 |
|  |  |  |  | Focus | 4 | 4 | 2 | 2 | 2 |
|  |  |  |  | Polarity | 7 | 7 | 7 | 7 | 7 |
| 395 | 281 | 11792723\_172 | Excluding unincorporated [1,1-3H]hexadecanol, it is evident that the myelin fraction contained less than 10% of net lipid incorporated radioactivity between 30 and 240 min, and less than 3% at 240 min. |A1:\*\*1SP3E3| |A2:\*\*1SP3E3| |A3:\*\*1SP3E3| |A4:\*\*1MP3E3| |A5:\*\*1MSP3E3| |  |  |  |  |  |  |
|  |  |  |  | Annotation | 1SP3E3 | 1SP3E3 | 1SP3E3 | 1MP3E3 | 1MSP3E3 |
|  |  |  |  | Evidence | 4 | 4 | 4 | 4 | 4 |
|  |  |  |  | Focus | 4 | 4 | 4 | 2 | 6 |
|  |  |  |  | Polarity | 7 | 7 | 7 | 7 | 7 |
| 396 | 4936 | 12138207\_254 | Because many of the cell cycle genes whose transient expression is diminished in the presence of Wnt |A5:\*\*1SP3E2-| are known to be regulated by E2F (25), |A5:\*\*2SP3E2| |A3:\*\*1SP2E2-| |A4:\*\*1SP2E2-| we hypothesized that E2F activity is reduced in Wnt-expressing cells. |A5:\*\*3SP2E3-| |A1:\*\*1SP2E23| |A2:\*\*1SP2E23-| |A3:\*\*2SP1E3-| |A4:\*\*2SP1E3-| |  |  |  |  |  |  |
|  |  |  |  | Annotation | 1SP2E23 1SP2E23 1SP2E23 | 1SP2E23- 1SP2E23- 1SP2E23- | 1SP2E2- 1SP2E2- 2SP1E3- | 1SP2E2- 1SP2E2- 2SP1E3- | 1SP3E2- 2SP3E2 3SP2E3- |
|  |  |  |  | Evidence | 3 3 3 | 3 3 3 | 3 3 4 | 3 3 4 | 3 3 4 |
|  |  |  |  | Focus | 4 4 4 | 4 4 4 | 4 4 4 | 4 4 4 | 4 4 4 |
|  |  |  |  | Polarity | 6 6 6 | 6 6 6 | 6 6 5 | 6 6 5 | 7 7 6 |
| 397 | 5741 | 10653722\_90 | The lipase activity of lactococcal colonies was determined qualitatively by use of an agar plate assay medium containing 1% Tributyrin (Sigma) as a substrate and with nisin at 0.5 mug/ml for JIM7048 and JIM7022. |A1:\*\*1MP3E3| |A2:\*\*1MP3E3| |A3:\*\*1MSP3E3| |A4:\*\*1MP3E3| |A5:\*\*1MP3E3| |  |  |  |  |  |  |
|  |  |  |  | Annotation | 1MP3E3 | 1MP3E3 | 1MSP3E3 | 1MP3E3 | 1MP3E3 |
|  |  |  |  | Evidence | 4 | 4 | 4 | 4 | 4 |
|  |  |  |  | Focus | 2 | 2 | 6 | 2 | 2 |
|  |  |  |  | Polarity | 7 | 7 | 7 | 7 | 7 |
| 398 | 8552 | 11182318\_363 | The solution was stirred for 72 h, extracted with 25 ml ether, and washed with 2x5 ml ice-cold H2O followed by 2x5 ml saturated NaCl salt solution. |A1:\*\*1MP3E3| |A2:\*\*1MP3E3| |A3:\*\*1MP3E3| |A4:\*\*1MP3E3| |A5:\*\*1MP3E3| |  |  |  |  |  |  |
|  |  |  |  | Annotation | 1MP3E3 | 1MP3E3 | 1MP3E3 | 1MP3E3 | 1MP3E3 |
|  |  |  |  | Evidence | 4 | 4 | 4 | 4 | 4 |
|  |  |  |  | Focus | 2 | 2 | 2 | 2 | 2 |
|  |  |  |  | Polarity | 7 | 7 | 7 | 7 | 7 |
| 399 | 5156 | 11698393\_11 | B, Southern blot analysis of the wild type MAT2 loci. |A1:\*\*1MP3E0| |A2:\*\*1MP3E0| |A3:\*\*1MSP3E0| |A4:\*\*1MP3E0| |A5:\*\*1MP3E3| |  |  |  |  |  |  |
|  |  |  |  | Annotation | 1MP3E0 | 1MP3E0 | 1MSP3E0 | 1MP3E0 | 1MP3E3 |
|  |  |  |  | Evidence | 1 | 1 | 1 | 1 | 4 |
|  |  |  |  | Focus | 2 | 2 | 6 | 2 | 2 |
|  |  |  |  | Polarity | 7 | 7 | 7 | 7 | 7 |
| 400 | 9347 | 9802902\_257 | One molecule that has been shown to a play a role in ARF6 cytoskeletal remodeling is POR1. |A1:\*\*1SP3E1| |A2:\*\*1SP3E1| |A3:\*\*1SP3E0| |A4:\*\*1SP1E0| |A5:\*\*1GP3E1| |  |  |  |  |  |  |
|  |  |  |  | Annotation | 1SP3E1 | 1SP3E1 | 1SP3E0 | 1SP1E0 | 1GP3E1 |
|  |  |  |  | Evidence | 2 | 2 | 1 | 1 | 2 |
|  |  |  |  | Focus | 4 | 4 | 4 | 4 | 1 |
|  |  |  |  | Polarity | 7 | 7 | 7 | 5 | 7 |
| 401 | 6056 | 11086011\_71 | Based on prior analysis of other mal mutants |A5:\*\*1GP3E1| we estimate that presence of mal25-1 |A5:\*\*2GP2E3| leads to an 400-fold increase in minichromosome loss (Fleig et al. 1996 ; 1996; Beinhauer et al. 1997 ). |A5:\*\*3GP3E2+| |A1:\*\*1SP3E23+| |A2:\*\*1SP3E23+| |A3:\*\*1SP3E2| |A4:\*\*1SP3E2| |  |  |  |  |  |  |
|  |  |  |  | Annotation | 1SP3E23+ 1SP3E23+ 1SP3E23+ | 1SP3E23+ 1SP3E23+ 1SP3E23+ | 1SP3E2 1SP3E2 1SP3E2 | 1SP3E2 1SP3E2 1SP3E2 | 1GP3E1 2GP2E3 3GP3E2+ |
|  |  |  |  | Evidence | 3 3 3 | 3 3 3 | 3 3 3 | 3 3 3 | 2 4 3 |
|  |  |  |  | Focus | 4 4 4 | 4 4 4 | 4 4 4 | 4 4 4 | 1 1 1 |
|  |  |  |  | Polarity | 7 7 7 | 7 7 7 | 7 7 7 | 7 7 7 | 7 6 7 |
| 402 | 3653 | 10052460\_216 | Since the three - amino acid insertion does not alter the way in which the homeodomain binds DNA (Wolberger et al., 1991), |A5:\*\*1SN3E2| |A1:\*\*1SN3E2| |A2:\*\*1SN3E2| |A3:\*\*1SN3E2| |A4:\*\*1SN3E2| the insertion may have originated as a benign mutation |A5:\*\*2SP2E0| that only later acquired the function of mediating interactions with other homeodomain proteins. |A5:\*\*3SP3E0| |A1:\*\*2SP1E0| |A2:\*\*2SP2E0| |A3:\*\*2SP1E0| |A4:\*\*2SP2E0| |  |  |  |  |  |  |
|  |  |  |  | Annotation | 1SN3E2 2SP1E0 2SP1E0 | 1SN3E2 2SP2E0 2SP2E0 | 1SN3E2 2SP1E0 2SP1E0 | 1SN3E2 2SP2E0 2SP2E0 | 1SN3E2 2SP2E0 3SP3E0 |
|  |  |  |  | Evidence | 3 1 1 | 3 1 1 | 3 1 1 | 3 1 1 | 3 1 1 |
|  |  |  |  | Focus | 4 4 4 | 4 4 4 | 4 4 4 | 4 4 4 | 4 4 4 |
|  |  |  |  | Polarity | 1 5 5 | 1 6 6 | 1 5 5 | 1 6 6 | 1 6 7 |
| 403 | 1850 | 10978286\_313 | Lack of a genetic interaction suggests that these genes may act in separate pathways to regulate the death process. |A1:\*\*1SP1E0| |A2:\*\*1SP2E1| |A3:\*\*1SP2E0| |A4:\*\*1SP2E1| |A5:\*\*1SP1E0| |  |  |  |  |  |  |
|  |  |  |  | Annotation | 1SP1E0 | 1SP2E1 | 1SP2E0 | 1SP2E1 | 1SP1E0 |
|  |  |  |  | Evidence | 1 | 2 | 1 | 2 | 1 |
|  |  |  |  | Focus | 4 | 4 | 4 | 4 | 4 |
|  |  |  |  | Polarity | 5 | 6 | 6 | 6 | 5 |
| 404 | 713 | 11483581\_17 | In the present study, we examined the conditions for identifying the genome of origin for a specific genomic sequence, using the genomic signature concept. |A1:\*\*1SP3E3| |A2:\*\*1SP3E3| |A3:\*\*1SP3E3| |A4:\*\*1SP3E3| |A5:\*\*1SP3E3| |  |  |  |  |  |  |
|  |  |  |  | Annotation | 1SP3E3 | 1SP3E3 | 1SP3E3 | 1SP3E3 | 1SP3E3 |
|  |  |  |  | Evidence | 4 | 4 | 4 | 4 | 4 |
|  |  |  |  | Focus | 4 | 4 | 4 | 4 | 4 |
|  |  |  |  | Polarity | 7 | 7 | 7 | 7 | 7 |
| 405 | 4691 | 10788396\_110 | Also, six clones from the filter biofilm and one clone for each APS reductase-positive strain were sequenced using the facilities of The Biotechnology Center of the University of Illinois. |A1:\*\*1SP3E3| |A2:\*\*1SP3E3| |A3:\*\*1MP2E0| |A4:\*\*1MP3E3| |A5:\*\*1MP3E3| |  |  |  |  |  |  |
|  |  |  |  | Annotation | 1SP3E3 | 1SP3E3 | 1MP2E0 | 1MP3E3 | 1MP3E3 |
|  |  |  |  | Evidence | 4 | 4 | 1 | 4 | 4 |
|  |  |  |  | Focus | 4 | 4 | 2 | 2 | 2 |
|  |  |  |  | Polarity | 7 | 7 | 6 | 7 | 7 |
| 406 | 1472 | 10862613\_42 | H2O2 is known to damage both DNA and protein via reactive oxygen species. |A1:\*\*1SP3E0| |A2:\*\*1SP3E0| |A3:\*\*1SP3E0| |A4:\*\*1SP3E0| |A5:\*\*1SP3E0| |  |  |  |  |  |  |
|  |  |  |  | Annotation | 1SP3E0 | 1SP3E0 | 1SP3E0 | 1SP3E0 | 1SP3E0 |
|  |  |  |  | Evidence | 1 | 1 | 1 | 1 | 1 |
|  |  |  |  | Focus | 4 | 4 | 4 | 4 | 4 |
|  |  |  |  | Polarity | 7 | 7 | 7 | 7 | 7 |
| 407 | 5934 | 9727009\_53 | The adenovirus Addl312 designated AdCNTL was used to control for viral infection (kindly provided by T. Shenk) (33). |A1:\*\*1SP3E23| |A2:\*\*1SP3E23| |A3:\*\*1SP3E2| |A4:\*\*1SP3E2| |A5:\*\*1GP3E2| |  |  |  |  |  |  |
|  |  |  |  | Annotation | 1SP3E23 | 1SP3E23 | 1SP3E2 | 1SP3E2 | 1GP3E2 |
|  |  |  |  | Evidence | 3 | 3 | 3 | 3 | 3 |
|  |  |  |  | Focus | 4 | 4 | 4 | 4 | 1 |
|  |  |  |  | Polarity | 7 | 7 | 7 | 7 | 7 |
| 408 | 3708 | 9390512\_26 | Mendelian diseases follow very specific patterns of inheritance in families (recessive, dominant, or X-linked). |A1:\*\*1GP3E0| |A2:\*\*1SP3E0| |A3:\*\*1SP3E0| |A4:\*\*1SP3E0| |A5:\*\*1GP3E0| |  |  |  |  |  |  |
|  |  |  |  | Annotation | 1GP3E0 | 1SP3E0 | 1SP3E0 | 1SP3E0 | 1GP3E0 |
|  |  |  |  | Evidence | 1 | 1 | 1 | 1 | 1 |
|  |  |  |  | Focus | 1 | 4 | 4 | 4 | 1 |
|  |  |  |  | Polarity | 7 | 7 | 7 | 7 | 7 |
| 409 | 3327 | 10882667\_122 | These sera from B. microti-infected patients did not react with EK-rP44-2hv antigen (OD405 < 0.139). |A1:\*\*1SN3E1| |A2:\*\*1SN3E3| |A3:\*\*1SN3E3| |A4:\*\*1SN3E0| |A5:\*\*1SN3E3| |  |  |  |  |  |  |
|  |  |  |  | Annotation | 1SN3E1 | 1SN3E3 | 1SN3E3 | 1SN3E0 | 1SN3E3 |
|  |  |  |  | Evidence | 2 | 4 | 4 | 1 | 4 |
|  |  |  |  | Focus | 4 | 4 | 4 | 4 | 4 |
|  |  |  |  | Polarity | 1 | 1 | 1 | 1 | 1 |
| 410 | 1984 | 8557638\_2 | This conclusion is based on the following observations: |A5:\*\*1GP3E1| 1) IL-1beta and IFN independently activate ERK1/ERK2 and increase NOS2 mRNA abundance in cardiac myocytes; |A5:\*\*2SP3E1+| |A2:\*\*1SP3E1+| |A3:\*\*1SP3E3+| |A4:\*\*1SP3E3+| 2) IL-1beta but not IFN activates ERK1/ERK2 and increases NOS2 mRNA levels in CMEC; |A5:\*\*3SN3E1+| |A1:\*\*1SP3E1+| |A2:\*\*2SN3E1+| |A3:\*\*2SN3E0+| |A4:\*\*2SN3E0+| 3) inhibition of IFN - and IL-1beta-linked signaling proteins |A5:\*\*4SP3E1-| leading to activation of ERK1/ERK2 in cardiac myocytes (i.e. PKCs and Ras) |A5:\*\*5SP3E1+| |A2:\*\*3SN3E1+| |A3:\*\*3SP3E0+| |A4:\*\*3SP3E0+| also inhibited IFN - and IL-1beta-induced NOS2 expression in these cells; |A5:\*\*6SP3E1-| |A1:\*\*2SN3E1+| |A2:\*\*4SP3E1-| |A3:\*\*4SP3E0-| |A4:\*\*4SP3E0-| 4) nonreceptor-mediated activation of ERK2, induced by the phosphoserine protein phosphatase inhibitor okadaic acid, induced NOS2 expression in CMEC; |A5:\*\*7SP3E1+| |A1:\*\*3SP3E1+| and |A3:\*\*5SP3E0+| |A4:\*\*5SP3E0+| 5) inhibition of IL-1beta-induced activation of MEK and ERK1/ERK2 in CMEC by PD 98059 |A1:\*\*4SN3E1+| also suppressed NOS2 induction in these cells. |A5:\*\*8SP3E1-| |A1:\*\*5SN3E1| |A2:\*\*5SP3E1-| |A3:\*\*6SP3E0-| |A4:\*\*6SP3E0-| |  |  |  |  |  |  |
|  |  |  |  | Annotation | 1SP3E1+ 1SP3E1+ 1SP3E1+ 2SN3E1+ 2SN3E1+ 2SN3E1+ 3SP3E1+ 4SN3E1+ 4SN3E1+ 5SN3E1 | 1SP3E1+ 1SP3E1+ 2SN3E1+ 3SN3E1+ 3SN3E1+ 4SP3E1- 5SP3E1- 5SP3E1- 5SP3E1- 5SP3E1- | 1SP3E3+ 1SP3E3+ 2SN3E0+ 3SP3E0+ 3SP3E0+ 4SP3E0- 5SP3E0+ 5SP3E0+ 6SP3E0- 6SP3E0- | 1SP3E3+ 1SP3E3+ 2SN3E0+ 3SP3E0+ 3SP3E0+ 4SP3E0- 5SP3E0+ 5SP3E0+ 6SP3E0- 6SP3E0- | 1GP3E1 2SP3E1+ 3SN3E1+ 4SP3E1- 5SP3E1+ 6SP3E1- 7SP3E1+ 8SP3E1- 8SP3E1- 8SP3E1- |
|  |  |  |  | Evidence | 2 2 2 2 2 2 2 2 2 2 | 2 2 2 2 2 2 2 2 2 2 | 4 4 1 1 1 1 1 1 1 1 | 4 4 1 1 1 1 1 1 1 1 | 2 2 2 2 2 2 2 2 2 2 |
|  |  |  |  | Focus | 4 4 4 4 4 4 4 4 4 4 | 4 4 4 4 4 4 4 4 4 4 | 4 4 4 4 4 4 4 4 4 4 | 4 4 4 4 4 4 4 4 4 4 | 1 4 4 4 4 4 4 4 4 4 |
|  |  |  |  | Polarity | 7 7 7 1 1 1 7 1 1 1 | 7 7 1 1 1 7 7 7 7 7 | 7 7 1 7 7 7 7 7 7 7 | 7 7 1 7 7 7 7 7 7 7 | 7 7 1 7 7 7 7 7 7 7 |
| 411 | 7330 | 10611247\_42 | For luciferase assays and stimulation of 293, the cells were plated at 105/35-mm-diameter dish 24 h prior to transfection. |A1:\*\*1MP3E3| |A2:\*\*1MP3E3| |A3:\*\*1MP3E3| |A4:\*\*1MP3E3| |A5:\*\*1MP3E3| |  |  |  |  |  |  |
|  |  |  |  | Annotation | 1MP3E3 | 1MP3E3 | 1MP3E3 | 1MP3E3 | 1MP3E3 |
|  |  |  |  | Evidence | 4 | 4 | 4 | 4 | 4 |
|  |  |  |  | Focus | 2 | 2 | 2 | 2 | 2 |
|  |  |  |  | Polarity | 7 | 7 | 7 | 7 | 7 |
| 412 | 4854 | 7706290\_41 | The initiation of disulfide bond formation coincided with the removal of the DTT during dialysis (see Fig. 3). |A1:\*\*1SP3E3| |A2:\*\*1SP3E3| |A3:\*\*1SP3E3| |A4:\*\*1SP3E3| |A5:\*\*1GP3E3| |  |  |  |  |  |  |
|  |  |  |  | Annotation | 1SP3E3 | 1SP3E3 | 1SP3E3 | 1SP3E3 | 1GP3E3 |
|  |  |  |  | Evidence | 4 | 4 | 4 | 4 | 4 |
|  |  |  |  | Focus | 4 | 4 | 4 | 4 | 1 |
|  |  |  |  | Polarity | 7 | 7 | 7 | 7 | 7 |
| 413 | 6857 | 9651682\_17 | If one ablates P7.p later on, however, P8.p has an epidermal fate [5, 6]. |A1:\*\*1SP3E2| |A2:\*\*1SP3E2| |A3:\*\*1SP3E2| |A4:\*\*1SP3E2| |A5:\*\*1GP3E2| |  |  |  |  |  |  |
|  |  |  |  | Annotation | 1SP3E2 | 1SP3E2 | 1SP3E2 | 1SP3E2 | 1GP3E2 |
|  |  |  |  | Evidence | 3 | 3 | 3 | 3 | 3 |
|  |  |  |  | Focus | 4 | 4 | 4 | 4 | 1 |
|  |  |  |  | Polarity | 7 | 7 | 7 | 7 | 7 |
| 414 | 3583 | 8663305\_77 | Open circles, sodium acetate; closed circles, Tris maleate; open squares, NaHepes; closed squares, Tris-HCl. |A1:\*\*1GP3E0| |A2:\*\*1GP3E0| |A3:\*\*1SP3E0| |A4:\*\*1SP3E0| |A5:\*\*1GP3E0| |  |  |  |  |  |  |
|  |  |  |  | Annotation | 1GP3E0 | 1GP3E0 | 1SP3E0 | 1SP3E0 | 1GP3E0 |
|  |  |  |  | Evidence | 1 | 1 | 1 | 1 | 1 |
|  |  |  |  | Focus | 1 | 1 | 4 | 4 | 1 |
|  |  |  |  | Polarity | 7 | 7 | 7 | 7 | 7 |
| 415 | 8884 | 11358691\_168 | A plasmid of 4000 bp was isolated from transformed E. coli XL1-Blue cells grown on LBcam and named pGSVII. |A1:\*\*1SP3E3| |A2:\*\*1SP3E3| |A3:\*\*1MP3E3| |A4:\*\*1SP3E0| |A5:\*\*1MP3E3| |  |  |  |  |  |  |
|  |  |  |  | Annotation | 1SP3E3 | 1SP3E3 | 1MP3E3 | 1SP3E0 | 1MP3E3 |
|  |  |  |  | Evidence | 4 | 4 | 4 | 1 | 4 |
|  |  |  |  | Focus | 4 | 4 | 2 | 4 | 2 |
|  |  |  |  | Polarity | 7 | 7 | 7 | 7 | 7 |
| 416 | 7549 | 10781541\_31 | The site of Tn 5 insertion was determined by constructing a plasmid library of the transposon insertion mutant, followed by screening the library for clones resistant to kanamycin kanamycin. |A1:\*\*1SP3E3| |A2:\*\*1SP3E3| |A3:\*\*1MSP3E3| |A4:\*\*1MP3E0| |A5:\*\*1MP3E3| |  |  |  |  |  |  |
|  |  |  |  | Annotation | 1SP3E3 | 1SP3E3 | 1MSP3E3 | 1MP3E0 | 1MP3E3 |
|  |  |  |  | Evidence | 4 | 4 | 4 | 1 | 4 |
|  |  |  |  | Focus | 4 | 4 | 6 | 2 | 2 |
|  |  |  |  | Polarity | 7 | 7 | 7 | 7 | 7 |
| 417 | 9817 | 12055200\_217 | In addition, the Wnt co-receptors LRP5 and LRP6 may mediate the Wnt10b signal |A5:\*\*1SP2E0| because both of these genes are expressed in 3T3-L1 preadipocytes and stromal vascular cells. |A5:\*\*2SP3E0| |A1:\*\*1SP1E3| |A2:\*\*1SP2E1| |A3:\*\*1SP3E0| |A4:\*\*1SP2E0| |  |  |  |  |  |  |
|  |  |  |  | Annotation | 1SP1E3 1SP1E3 | 1SP2E1 1SP2E1 | 1SP3E0 1SP3E0 | 1SP2E0 1SP2E0 | 1SP2E0 2SP3E0 |
|  |  |  |  | Evidence | 4 4 | 2 2 | 1 1 | 1 1 | 1 1 |
|  |  |  |  | Focus | 4 4 | 4 4 | 4 4 | 4 4 | 4 4 |
|  |  |  |  | Polarity | 5 5 | 6 6 | 7 7 | 6 6 | 6 7 |
| 418 | 3957 | 9813087\_66 | The Cl channel blocker diphenylamine-2-carboxylic acid (DPC) also inhibited luciferin-luciferase activity. |A1:\*\*1SP3E3| |A2:\*\*1SP3E0-| |A3:\*\*1SP3E0| |A4:\*\*1SP3E0-| |A5:\*\*1GP3E0-| |  |  |  |  |  |  |
|  |  |  |  | Annotation | 1SP3E3 | 1SP3E0- | 1SP3E0 | 1SP3E0- | 1GP3E0- |
|  |  |  |  | Evidence | 4 | 1 | 1 | 1 | 1 |
|  |  |  |  | Focus | 4 | 4 | 4 | 4 | 1 |
|  |  |  |  | Polarity | 7 | 7 | 7 | 7 | 7 |
| 419 | 621 | 11087000\_385 | Sound was delivered to the tympanic membrane by a closed acoustic system comprised of two Bruel & Kjaer 4133 1/2-inch microphones for delivering tones and a single Bruel & Kjaer 3135 1/4-inch microphone for monitoring sound pressure at the tympanum. |A1:\*\*1GP3E3| |A2:\*\*1GP3E3| |A3:\*\*1SP3E3| |A4:\*\*1SP3E3| |A5:\*\*1MP3E3| |  |  |  |  |  |  |
|  |  |  |  | Annotation | 1GP3E3 | 1GP3E3 | 1SP3E3 | 1SP3E3 | 1MP3E3 |
|  |  |  |  | Evidence | 4 | 4 | 4 | 4 | 4 |
|  |  |  |  | Focus | 1 | 1 | 4 | 4 | 2 |
|  |  |  |  | Polarity | 7 | 7 | 7 | 7 | 7 |
| 420 | 3108 | 11313360\_6 | We tested the ability of staurosporine to inhibit EH-evoked secretion of ETH by EGs in vitro. |A1:\*\*1SN3E3| |A2:\*\*1SP3E3| |A3:\*\*1SP3E3| |A4:\*\*1SP3E3-| |A5:\*\*1GP3E3| |  |  |  |  |  |  |
|  |  |  |  | Annotation | 1SN3E3 | 1SP3E3 | 1SP3E3 | 1SP3E3- | 1GP3E3 |
|  |  |  |  | Evidence | 4 | 4 | 4 | 4 | 4 |
|  |  |  |  | Focus | 4 | 4 | 4 | 4 | 1 |
|  |  |  |  | Polarity | 1 | 7 | 7 | 7 | 7 |
| 421 | 5903 | 11278429\_9 | In this case, p56 lck activation is detectable early (5 min after activation), as is the case in IPP stimulation of gamma9delta2 T cells. |A1:\*\*1SP3E3| |A2:\*\*1SP3E3+| |A3:\*\*1SP3E3| |A4:\*\*1SP3E3+| |A5:\*\*1SP3E3+| |  |  |  |  |  |  |
|  |  |  |  | Annotation | 1SP3E3 | 1SP3E3+ | 1SP3E3 | 1SP3E3+ | 1SP3E3+ |
|  |  |  |  | Evidence | 4 | 4 | 4 | 4 | 4 |
|  |  |  |  | Focus | 4 | 4 | 4 | 4 | 4 |
|  |  |  |  | Polarity | 7 | 7 | 7 | 7 | 7 |
| 422 | 8317 | 9224811\_228 | Whether this is due to an extended TR-binding site or binding of an additional protein(s) is not clear; |A3:\*\*1SP1E0| |A1:\*\*1SP0E3| |A2:\*\*1SP0E0| |A4:\*\*1SP0E0| |A5:\*\*1GN0E0| however, the fact that protein binding occurs further upstream of the P450R-TRE |A3:\*\*2SP3E0| |A5:\*\*2GP3E0| may explain the small but significant drop in T3 induction found in constructs p-575R and p-564R. |A3:\*\*3SP1E0| |A1:\*\*2SP1E0| |A2:\*\*2SP1E0| |A4:\*\*2SP1E0-| |A5:\*\*3GP2E0-| |  |  |  |  |  |  |
|  |  |  |  | Annotation | 1SP0E3 2SP1E0 2SP1E0 | 1SP0E0 2SP1E0 2SP1E0 | 1SP1E0 2SP3E0 3SP1E0 | 1SP0E0 2SP1E0- 2SP1E0- | 1GN0E0 2GP3E0 3GP2E0- |
|  |  |  |  | Evidence | 4 1 1 | 1 1 1 | 1 1 1 | 1 1 1 | 1 1 1 |
|  |  |  |  | Focus | 4 4 4 | 4 4 4 | 4 4 4 | 4 4 4 | 1 1 1 |
|  |  |  |  | Polarity | 4 5 5 | 4 5 5 | 5 7 5 | 4 5 5 | 4 7 6 |
| 423 | 9880 | 12086892\_22 | The VEGF family is comprised of five structurally related members, including VEGF-A, placenta growth factor (PlGF), VEGF-B, VEGF-C, and VEGF-D (Eriksson and Alitalo, 1999 ). |A1:\*\*1SP3E2| |A2:\*\*1SP3E2| |A3:\*\*1SP3E2| |A4:\*\*1SP3E2| |A5:\*\*1GP3E2| |  |  |  |  |  |  |
|  |  |  |  | Annotation | 1SP3E2 | 1SP3E2 | 1SP3E2 | 1SP3E2 | 1GP3E2 |
|  |  |  |  | Evidence | 3 | 3 | 3 | 3 | 3 |
|  |  |  |  | Focus | 4 | 4 | 4 | 4 | 1 |
|  |  |  |  | Polarity | 7 | 7 | 7 | 7 | 7 |
| 424 | 2366 | 12062057\_130 | Because Drosophila males do not undergo recombination or assemble a synaptonemal complex, |A3:\*\*1SN3E0| it is unlikely that ORD functions exclusively in these processes during female meiosis. |A3:\*\*2SP2E0| |A1:\*\*1SP3E1| |A2:\*\*1SN3E1| |A4:\*\*1SN3E0| |A5:\*\*1SN3E0| |  |  |  |  |  |  |
|  |  |  |  | Annotation | 1SP3E1 1SP3E1 | 1SN3E1 1SN3E1 | 1SN3E0 2SP2E0 | 1SN3E0 1SN3E0 | 1SN3E0 1SN3E0 |
|  |  |  |  | Evidence | 2 2 | 2 2 | 1 1 | 1 1 | 1 1 |
|  |  |  |  | Focus | 4 4 | 4 4 | 4 4 | 4 4 | 4 4 |
|  |  |  |  | Polarity | 7 7 | 1 1 | 1 6 | 1 1 | 1 1 |
| 425 | 5871 | 9271353\_95 | These data demonstrate that NQO1 can reduce TQ considerably more efficiently than CoQ10. |A1:\*\*1SP1E3| |A2:\*\*1SP2E1-| |A3:\*\*1SP2E3| |A4:\*\*1SP3E3-| |A5:\*\*1GP3E3-| |  |  |  |  |  |  |
|  |  |  |  | Annotation | 1SP1E3 | 1SP2E1- | 1SP2E3 | 1SP3E3- | 1GP3E3- |
|  |  |  |  | Evidence | 4 | 2 | 4 | 4 | 4 |
|  |  |  |  | Focus | 4 | 4 | 4 | 4 | 1 |
|  |  |  |  | Polarity | 5 | 6 | 6 | 7 | 7 |
| 426 | 7151 | 10698947\_306 | The recombinant plasmid was named pAD2 and was used to overproduce CrgA. |A1:\*\*1SP3E3| |A2:\*\*1SP3E3| |A3:\*\*1MSP3E3| |A4:\*\*1SP3E0| |A5:\*\*1GP3E3| |  |  |  |  |  |  |
|  |  |  |  | Annotation | 1SP3E3 | 1SP3E3 | 1MSP3E3 | 1SP3E0 | 1GP3E3 |
|  |  |  |  | Evidence | 4 | 4 | 4 | 1 | 4 |
|  |  |  |  | Focus | 4 | 4 | 6 | 4 | 1 |
|  |  |  |  | Polarity | 7 | 7 | 7 | 7 | 7 |
| 427 | 9187 | 11030628\_402 | A similar approach was used for generating the anti-p-Ser-46 polyclonal antibody. |A1:\*\*1SP3E3| |A2:\*\*1SP3E3| |A3:\*\*1MP3E3| |A4:\*\*1SP3E0| |A5:\*\*1GP3E3| |  |  |  |  |  |  |
|  |  |  |  | Annotation | 1SP3E3 | 1SP3E3 | 1MP3E3 | 1SP3E0 | 1GP3E3 |
|  |  |  |  | Evidence | 4 | 4 | 4 | 1 | 4 |
|  |  |  |  | Focus | 4 | 4 | 2 | 4 | 1 |
|  |  |  |  | Polarity | 7 | 7 | 7 | 7 | 7 |
| 428 | 2878 | 9475757\_238 | CAUSSE, M. A., T. M. FULTON, Y. G. CHO, S. N. AHN, and J. CHUNWONGSE et al., 1994 Saturated molecular map of the rice genome based on an interspecific backcross population. |A1:\*\*1SP3E3| |A2:\*\*1SP3E3| |A3:\*\*1SP3E0| |A4:\*\*1SP3E2| |A5:\*\*1SP3E2| |  |  |  |  |  |  |
|  |  |  |  | Annotation | 1SP3E3 | 1SP3E3 | 1SP3E0 | 1SP3E2 | 1SP3E2 |
|  |  |  |  | Evidence | 4 | 4 | 1 | 3 | 3 |
|  |  |  |  | Focus | 4 | 4 | 4 | 4 | 4 |
|  |  |  |  | Polarity | 7 | 7 | 7 | 7 | 7 |
| 429 | 7092 | 11325960\_1 | Substantial evidence exists in literature to suggest that placental protein 14 (PP14) (recently renamed glycodelin A), exhibits immunosuppressive properties and is an indispensable macromolecule in the maternal system for the establishment, maintenance, and progression of pregnancy. |A1:\*\*1SP3E1| |A2:\*\*1SP3E1| |A3:\*\*1SP3E1| |A4:\*\*1SP3E1| |A5:\*\*1GP3E1| |  |  |  |  |  |  |
|  |  |  |  | Annotation | 1SP3E1 | 1SP3E1 | 1SP3E1 | 1SP3E1 | 1GP3E1 |
|  |  |  |  | Evidence | 2 | 2 | 2 | 2 | 2 |
|  |  |  |  | Focus | 4 | 4 | 4 | 4 | 1 |
|  |  |  |  | Polarity | 7 | 7 | 7 | 7 | 7 |
| 430 | 8673 | 12618190\_43 | The 40-mer RNA aptamer construct for crystallization trials lacks the hairpin loop and is composed of two strands with single complementary base overhangs at their 5 ends (Figure 1A). |A1:\*\*1SP3E3| |A2:\*\*1SP3E3| |A3:\*\*1SP3E3| |A4:\*\*1SP3E3| |A5:\*\*1SP3E3| |  |  |  |  |  |  |
|  |  |  |  | Annotation | 1SP3E3 | 1SP3E3 | 1SP3E3 | 1SP3E3 | 1SP3E3 |
|  |  |  |  | Evidence | 4 | 4 | 4 | 4 | 4 |
|  |  |  |  | Focus | 4 | 4 | 4 | 4 | 4 |
|  |  |  |  | Polarity | 7 | 7 | 7 | 7 | 7 |
| 431 | 1929 | 9560388\_594 | ZACHARIAE, W. and K. NASMYTH, 1996 TPR proteins required for anaphase progression mediate ubiquitination of mitotic B-type cyclins in yeast. |A1:\*\*1SP3E3| |A2:\*\*1SP3E3| |A3:\*\*1SP3E0| |A4:\*\*1SP3E2| |A5:\*\*1SP3E2| |  |  |  |  |  |  |
|  |  |  |  | Annotation | 1SP3E3 | 1SP3E3 | 1SP3E0 | 1SP3E2 | 1SP3E2 |
|  |  |  |  | Evidence | 4 | 4 | 1 | 3 | 3 |
|  |  |  |  | Focus | 4 | 4 | 4 | 4 | 4 |
|  |  |  |  | Polarity | 7 | 7 | 7 | 7 | 7 |
| 432 | 5339 | 12618405\_146 | As expected from the conservation of the active site sequence motif, recombinant FoTRI201 produced in E. coli could specifically transfer an acetyl group to C-3 of T-2 toxin, nivalenol (NIV), and deoxynivalenol (DON) (Fig 4C). |A1:\*\*1SP1E3| |A2:\*\*1SP2E3| |A3:\*\*1SP3E3| |A4:\*\*1SP3E3| |A5:\*\*1SP3E3| |  |  |  |  |  |  |
|  |  |  |  | Annotation | 1SP1E3 | 1SP2E3 | 1SP3E3 | 1SP3E3 | 1SP3E3 |
|  |  |  |  | Evidence | 4 | 4 | 4 | 4 | 4 |
|  |  |  |  | Focus | 4 | 4 | 4 | 4 | 4 |
|  |  |  |  | Polarity | 5 | 6 | 7 | 7 | 7 |
| 433 | 1609 | 9736749\_86 | Furthermore, a comparison of the two subject groups with regard to the global scale and skew of the hippocampus in three dimensions as derived from the transformations gave a similarly weak result (Hotelling's t2 = 11.8, df = 6, P = 0.19). |A1:\*\*1SP3E3| |A2:\*\*1SP3E3| |A3:\*\*1SP3E3| |A4:\*\*1SP1E0| |A5:\*\*1SP3E3| |  |  |  |  |  |  |
|  |  |  |  | Annotation | 1SP3E3 | 1SP3E3 | 1SP3E3 | 1SP1E0 | 1SP3E3 |
|  |  |  |  | Evidence | 4 | 4 | 4 | 1 | 4 |
|  |  |  |  | Focus | 4 | 4 | 4 | 4 | 4 |
|  |  |  |  | Polarity | 7 | 7 | 7 | 5 | 7 |
| 434 | 1159 | 9671490\_44 | Subclones containing the amino-terminal deletion mutant LEF-1delta56 and the LEF-1HMG domain were constructed by using naturally occurring restriction enzyme sites, HpaI and NdeI, respectively. |A1:\*\*1SP3E3| |A2:\*\*1SP3E3| |A3:\*\*1MP3E3| |A4:\*\*1SP3E0| |A5:\*\*1MP3E3| |  |  |  |  |  |  |
|  |  |  |  | Annotation | 1SP3E3 | 1SP3E3 | 1MP3E3 | 1SP3E0 | 1MP3E3 |
|  |  |  |  | Evidence | 4 | 4 | 4 | 1 | 4 |
|  |  |  |  | Focus | 4 | 4 | 2 | 4 | 2 |
|  |  |  |  | Polarity | 7 | 7 | 7 | 7 | 7 |
| 435 | 6772 | 10952693\_5 | This was in part due to impaired NO contribution NO-contribution in MI (-50%, P<0.05 versus no MI). |A1:\*\*1SN3E3| |A2:\*\*1GP3E3| |A3:\*\*1SP3E1| |A4:\*\*1SP2E0| |A5:\*\*1GP3E0| |  |  |  |  |  |  |
|  |  |  |  | Annotation | 1SN3E3 | 1GP3E3 | 1SP3E1 | 1SP2E0 | 1GP3E0 |
|  |  |  |  | Evidence | 4 | 4 | 2 | 1 | 1 |
|  |  |  |  | Focus | 4 | 1 | 4 | 4 | 1 |
|  |  |  |  | Polarity | 1 | 7 | 7 | 6 | 7 |
| 436 | 7217 | 11526109\_72 | Additionally, when both the PH and PTB domains were mutated, |A3:\*\*1SP3E0| |A5:\*\*1SP3E0| the reduction in plasma membrane association was not detectably different from the effect of the R28C mutation alone. |A3:\*\*2SN3E3| |A1:\*\*1SP3E3| |A2:\*\*1SN3E3| |A4:\*\*1SN3E0-| |A5:\*\*2SN3E0| |  |  |  |  |  |  |
|  |  |  |  | Annotation | 1SP3E3 1SP3E3 | 1SN3E3 1SN3E3 | 1SP3E0 2SN3E3 | 1SN3E0- 1SN3E0- | 1SP3E0 2SN3E0 |
|  |  |  |  | Evidence | 4 4 | 4 4 | 1 4 | 1 1 | 1 1 |
|  |  |  |  | Focus | 4 4 | 4 4 | 4 4 | 4 4 | 4 4 |
|  |  |  |  | Polarity | 7 7 | 1 1 | 7 1 | 1 1 | 7 1 |
| 437 | 1349 | 10454379\_186 | In mollusks, for example, evidence favors the interpretation that both functions are closely related or identical. |A1:\*\*1GP3E0| |A2:\*\*1GP3E1| |A3:\*\*1SP3E1| |A4:\*\*1SP3E0| |A5:\*\*1SP3E1| |  |  |  |  |  |  |
|  |  |  |  | Annotation | 1GP3E0 | 1GP3E1 | 1SP3E1 | 1SP3E0 | 1SP3E1 |
|  |  |  |  | Evidence | 1 | 2 | 2 | 1 | 2 |
|  |  |  |  | Focus | 1 | 1 | 4 | 4 | 4 |
|  |  |  |  | Polarity | 7 | 7 | 7 | 7 | 7 |
| 438 | 3489 | 12626754\_6 | Our study demonstrates that Wnt-5A augments primitive hematopoietic development in vivo |A5:\*\*1SP3E3+| and represents an in vivo regulator of hematopoietic stem cell function in the human. |A5:\*\*2SP3E3| |A1:\*\*1SP3E3| |A2:\*\*1SP3E3| |A3:\*\*1SP3E3| |A4:\*\*1SP3E3| |  |  |  |  |  |  |
|  |  |  |  | Annotation | 1SP3E3 1SP3E3 | 1SP3E3 1SP3E3 | 1SP3E3 1SP3E3 | 1SP3E3 1SP3E3 | 1SP3E3+ 2SP3E3 |
|  |  |  |  | Evidence | 4 4 | 4 4 | 4 4 | 4 4 | 4 4 |
|  |  |  |  | Focus | 4 4 | 4 4 | 4 4 | 4 4 | 4 4 |
|  |  |  |  | Polarity | 7 7 | 7 7 | 7 7 | 7 7 | 7 7 |
| 439 | 5263 | 9671454\_109 | Then, to normalize the transfection efficiency, 50 mul of stop-and-glow buffer was added, and the renilla luciferase activity was determined. |A1:\*\*1MP3E3| |A2:\*\*1MP3E3| |A3:\*\*1MP3E3| |A4:\*\*1SP3E0| |A5:\*\*1MP3E3| |  |  |  |  |  |  |
|  |  |  |  | Annotation | 1MP3E3 | 1MP3E3 | 1MP3E3 | 1SP3E0 | 1MP3E3 |
|  |  |  |  | Evidence | 4 | 4 | 4 | 1 | 4 |
|  |  |  |  | Focus | 2 | 2 | 2 | 4 | 2 |
|  |  |  |  | Polarity | 7 | 7 | 7 | 7 | 7 |
| 440 | 5571 | 11438642\_282 | At present much attention is focused on the possibility that Rrn3p activity is controlled by a regulatory cycle |A5:\*\*1SP2E0| in which it dissociates from Pol I during initiation, |A5:\*\*2SP3E0| is inactivated, |A1:\*\*1SP3E3-| |A2:\*\*1SP2E1-| and must be reactivated before it can direct initiation by a second polymerase. |A5:\*\*3SP3E0+| |A1:\*\*2SP2E3| |A2:\*\*2SP2E0| |A3:\*\*1SP2E0| |A4:\*\*1SP3E0| |  |  |  |  |  |  |
|  |  |  |  | Annotation | 1SP3E3- 1SP3E3- 1SP3E3- 2SP2E3 | 1SP2E1- 1SP2E1- 1SP2E1- 2SP2E0 | 1SP2E0 1SP2E0 1SP2E0 1SP2E0 | 1SP3E0 1SP3E0 1SP3E0 1SP3E0 | 1SP2E0 2SP3E0 3SP3E0+ 3SP3E0+ |
|  |  |  |  | Evidence | 4 4 4 4 | 2 2 2 1 | 1 1 1 1 | 1 1 1 1 | 1 1 1 1 |
|  |  |  |  | Focus | 4 4 4 4 | 4 4 4 4 | 4 4 4 4 | 4 4 4 4 | 4 4 4 4 |
|  |  |  |  | Polarity | 7 7 7 6 | 6 6 6 6 | 6 6 6 6 | 7 7 7 7 | 6 7 7 7 |
| 441 | 9758 | 9472020\_311 | In experiments where the effect of eIF2alpha phosphorylation was assessed, eIF2 in binary complex buffer was first incubated with or without 1 mul (~0.1 mug) of purified rabbit reticulocyte HCR kinase (Jackson and Hunt 1985 ) for 10 min at 23 degrees C. |A1:\*\*1MP3E3| |A2:\*\*1MP3E23| |A3:\*\*1SP3E3| |A4:\*\*1MP3E3| |A5:\*\*1MP3E3| |  |  |  |  |  |  |
|  |  |  |  | Annotation | 1MP3E3 | 1MP3E23 | 1SP3E3 | 1MP3E3 | 1MP3E3 |
|  |  |  |  | Evidence | 4 | 3 | 4 | 4 | 4 |
|  |  |  |  | Focus | 2 | 2 | 4 | 2 | 2 |
|  |  |  |  | Polarity | 7 | 7 | 7 | 7 | 7 |
| 442 | 6479 | 9395407\_275 | For RNA in situ hybridization, we followed the protocol of Tautz and Pfeifle [57] with modifications. |A1:\*\*1MP3E23| |A2:\*\*1SP3E23| |A3:\*\*1MP3E2| |A4:\*\*1MP3E2| |A5:\*\*1MP3E3| |  |  |  |  |  |  |
|  |  |  |  | Annotation | 1MP3E23 | 1SP3E23 | 1MP3E2 | 1MP3E2 | 1MP3E3 |
|  |  |  |  | Evidence | 3 | 3 | 3 | 3 | 4 |
|  |  |  |  | Focus | 2 | 4 | 2 | 2 | 2 |
|  |  |  |  | Polarity | 7 | 7 | 7 | 7 | 7 |
| 443 | 5547 | 11443132\_9 | Oxidative stress was induced by a 15-min pulse of H2O2 (25, 50, or 100 muM), and cell survival was measured 18 h later. |A1:\*\*1SP3E3| |A2:\*\*1SP3E3| |A3:\*\*1MP3E3+| |A4:\*\*1MP3E3+| |A5:\*\*1SP3E3+| |  |  |  |  |  |  |
|  |  |  |  | Annotation | 1SP3E3 | 1SP3E3 | 1MP3E3+ | 1MP3E3+ | 1SP3E3+ |
|  |  |  |  | Evidence | 4 | 4 | 4 | 4 | 4 |
|  |  |  |  | Focus | 4 | 4 | 2 | 2 | 4 |
|  |  |  |  | Polarity | 7 | 7 | 7 | 7 | 7 |
| 444 | 9485 | 9727029\_229 | Our finding that hSOCS-2 binds only to the autophosphorylated IGF-I receptor is consistent with hSOCS-2 SH2 domain binding to a phosphotyrosine containing motif in the receptor. |A1:\*\*1SP3E3| |A2:\*\*1SP3E3| |A3:\*\*1SP3E3| |A4:\*\*1SP3E3| |A5:\*\*1SP3E3| |  |  |  |  |  |  |
|  |  |  |  | Annotation | 1SP3E3 | 1SP3E3 | 1SP3E3 | 1SP3E3 | 1SP3E3 |
|  |  |  |  | Evidence | 4 | 4 | 4 | 4 | 4 |
|  |  |  |  | Focus | 4 | 4 | 4 | 4 | 4 |
|  |  |  |  | Polarity | 7 | 7 | 7 | 7 | 7 |
| 445 | 848 | 11159982\_69 | Successful deletion and mutation in the resultant clones were confirmed by restriction analysis with EcoRV and finally verified by automated DNA sequencing. |A1:\*\*1SP3E3| |A2:\*\*1SP3E3| |A3:\*\*1MP3E3| |A4:\*\*1MP3E3| |A5:\*\*1MP3E3| |  |  |  |  |  |  |
|  |  |  |  | Annotation | 1SP3E3 | 1SP3E3 | 1MP3E3 | 1MP3E3 | 1MP3E3 |
|  |  |  |  | Evidence | 4 | 4 | 4 | 4 | 4 |
|  |  |  |  | Focus | 4 | 4 | 2 | 2 | 2 |
|  |  |  |  | Polarity | 7 | 7 | 7 | 7 | 7 |
| 446 | 6810 | 10394365\_19 | The T = 4 capsids corresponding to a particular primary patient isolate (CW) but truncated after aa 149 have been crystallized (Wynne et al. 1999). |A1:\*\*1SP3E2| |A2:\*\*1SP3E2| |A3:\*\*1MP3E2| |A4:\*\*1SP3E2| |A5:\*\*1GP3E2| |  |  |  |  |  |  |
|  |  |  |  | Annotation | 1SP3E2 | 1SP3E2 | 1MP3E2 | 1SP3E2 | 1GP3E2 |
|  |  |  |  | Evidence | 3 | 3 | 3 | 3 | 3 |
|  |  |  |  | Focus | 4 | 4 | 2 | 4 | 1 |
|  |  |  |  | Polarity | 7 | 7 | 7 | 7 | 7 |
| 447 | 2951 | 9407054\_69 | As shown in Fig. 6 A, the plasma level of TNF-alpha in LPS-treated PLA2R-deficient mice was significantly lower than that in wild-type mice (male PLA2R+/+, PLA2R, 3.64 plus-or-minus 1.17 ng/ml; male PLA2R /, 1.54 plus-or-minus 0.49 ng/ml, p = 0.0143, female PLA2R+/+, PLA2R, 9.61 |A1:\*\*1SP3E3-| |A2:\*\*1SP3E3-| |A3:\*\*1SP3E3| |A4:\*\*1SP3E3| |A5:\*\*1GP3E3| |  |  |  |  |  |  |
|  |  |  |  | Annotation | 1SP3E3- | 1SP3E3- | 1SP3E3 | 1SP3E3 | 1GP3E3 |
|  |  |  |  | Evidence | 4 | 4 | 4 | 4 | 4 |
|  |  |  |  | Focus | 4 | 4 | 4 | 4 | 1 |
|  |  |  |  | Polarity | 7 | 7 | 7 | 7 | 7 |
| 448 | 9185 | 12046995\_1 | GOALS: The physicians' decision-making process in terminal care is complex: medical, ethical, legal and psychological aspects are all involved, particularly in critical situations. |A1:\*\*1SP3E0| |A2:\*\*1SP3E0| |A3:\*\*1SP3E0| |A4:\*\*1GP3E0| |A5:\*\*1GP3E0| |  |  |  |  |  |  |
|  |  |  |  | Annotation | 1SP3E0 | 1SP3E0 | 1SP3E0 | 1GP3E0 | 1GP3E0 |
|  |  |  |  | Evidence | 1 | 1 | 1 | 1 | 1 |
|  |  |  |  | Focus | 4 | 4 | 4 | 1 | 1 |
|  |  |  |  | Polarity | 7 | 7 | 7 | 7 | 7 |
| 449 | 6588 | 11118449\_51 | However, the GutR system has its unique aspects that are different from the MalT system. |A1:\*\*1SP3E0| |A2:\*\*1SP3E0| |A3:\*\*1SP3E0| |A4:\*\*1SP3E0| |A5:\*\*1MP3E0| |  |  |  |  |  |  |
|  |  |  |  | Annotation | 1SP3E0 | 1SP3E0 | 1SP3E0 | 1SP3E0 | 1MP3E0 |
|  |  |  |  | Evidence | 1 | 1 | 1 | 1 | 1 |
|  |  |  |  | Focus | 4 | 4 | 4 | 4 | 2 |
|  |  |  |  | Polarity | 7 | 7 | 7 | 7 | 7 |
| 450 | 4183 | 10681542\_1 | Expression and Purification of Wild Type and Mutant Enzymes-- The porcine NADP-dependent isocitrate dehydrogenase, with glutamine substituted for arginine at each of the positions 101, 110, 120, and 133, were generated using expression vector pMALcIDP1 by a megaprimer PCR method ( ). |A1:\*\*1SP3E23| |A2:\*\*1SP3E23| |A3:\*\*1MP3E3| |A4:\*\*1MP3E0| |A5:\*\*1SMP3E3| |  |  |  |  |  |  |
|  |  |  |  | Annotation | 1SP3E23 | 1SP3E23 | 1MP3E3 | 1MP3E0 | 1SMP3E3 |
|  |  |  |  | Evidence | 3 | 3 | 4 | 1 | 4 |
|  |  |  |  | Focus | 4 | 4 | 2 | 2 | 6 |
|  |  |  |  | Polarity | 7 | 7 | 7 | 7 | 7 |
| 451 | 6390 | 9030530\_2 | As to GSTs, only Ya Ya Ya-Ya and Ya Yc Ya-Yc could catalyze the reduction of cholesterol 7 hydroperoxides 7-hydroperoxides (Table I). |A1:\*\*1SP2E3| |A2:\*\*1SP2E3| |A3:\*\*1SP3E3-| |A4:\*\*1SP3E3-| |A5:\*\*1SP3E3+| |  |  |  |  |  |  |
|  |  |  |  | Annotation | 1SP2E3 | 1SP2E3 | 1SP3E3- | 1SP3E3- | 1SP3E3+ |
|  |  |  |  | Evidence | 4 | 4 | 4 | 4 | 4 |
|  |  |  |  | Focus | 4 | 4 | 4 | 4 | 4 |
|  |  |  |  | Polarity | 6 | 6 | 7 | 7 | 7 |
| 452 | 3989 | 12750340\_43 | Tissue samples of P. leucopus and P. californicus were furnished by the Peromyscus Stock Center ( http://stkctr.biol. |A1:\*\*1SP3E3| |A2:\*\*1SP3E3| |A3:\*\*1SP3E0| |A4:\*\*1SP3E0| |A5:\*\*1GP3E3| |  |  |  |  |  |  |
|  |  |  |  | Annotation | 1SP3E3 | 1SP3E3 | 1SP3E0 | 1SP3E0 | 1GP3E3 |
|  |  |  |  | Evidence | 4 | 4 | 1 | 1 | 4 |
|  |  |  |  | Focus | 4 | 4 | 4 | 4 | 1 |
|  |  |  |  | Polarity | 7 | 7 | 7 | 7 | 7 |
| 453 | 5564 | 12216476\_5 | We injected puromysin aminonucleoside(PAN) to induce the nephrotic rat state and isolated kidney lysosomes from normal and nephrotic rat kidney cortex by our methods. |A1:\*\*1SP3E3| |A2:\*\*1SP3E3| |A3:\*\*1MP3E3| |A4:\*\*1SP3E3| |A5:\*\*1MP3E3| |  |  |  |  |  |  |
|  |  |  |  | Annotation | 1SP3E3 | 1SP3E3 | 1MP3E3 | 1SP3E3 | 1MP3E3 |
|  |  |  |  | Evidence | 4 | 4 | 4 | 4 | 4 |
|  |  |  |  | Focus | 4 | 4 | 2 | 4 | 2 |
|  |  |  |  | Polarity | 7 | 7 | 7 | 7 | 7 |
| 454 | 6235 | 11014821\_339 | It should be noted that several of the same Doa alleles that fail to interact with ras pathway components interact to various extents with other mutations affecting eye and imaginal development (C. DU and L. RABINOW, unpublished results), as well as with those influencing sex determination ( D U et al. 1998 ). |A1:\*\*1SP3E2| |A2:\*\*1SP3E2| |A3:\*\*1SP3E2| |A4:\*\*1SP3E2| |A5:\*\*1SP3E2| |  |  |  |  |  |  |
|  |  |  |  | Annotation | 1SP3E2 | 1SP3E2 | 1SP3E2 | 1SP3E2 | 1SP3E2 |
|  |  |  |  | Evidence | 3 | 3 | 3 | 3 | 3 |
|  |  |  |  | Focus | 4 | 4 | 4 | 4 | 4 |
|  |  |  |  | Polarity | 7 | 7 | 7 | 7 | 7 |
| 455 | 4145 | 11238455\_281 | These cell lines all exhibit higher GSH levels |A2:\*\*1SP3E1+| than controls after glutamate exposure |A5:\*\*1MP3E3+| and lower levels of ROS and intracellular Ca2+. |A2:\*\*1SP3E0-| |A1:\*\*1SP3E0| |A3:\*\*1SP3E0| |A4:\*\*1SP3E3| |A5:\*\*2MP3E3-| |  |  |  |  |  |  |
|  |  |  |  | Annotation | 1SP3E0 1SP3E0 1SP3E0 | 1SP3E1+ 1SP3E0- 1SP3E0- | 1SP3E0 1SP3E0 1SP3E0 | 1SP3E3 1SP3E3 1SP3E3 | 1MP3E3+ 1MP3E3+ 2MP3E3- |
|  |  |  |  | Evidence | 1 1 1 | 2 1 1 | 1 1 1 | 4 4 4 | 4 4 4 |
|  |  |  |  | Focus | 4 4 4 | 4 4 4 | 4 4 4 | 4 4 4 | 2 2 2 |
|  |  |  |  | Polarity | 7 7 7 | 7 7 7 | 7 7 7 | 7 7 7 | 7 7 7 |
| 456 | 8598 | 12021257\_88 | Thus, CsA had only a minor effect on cytochrome c release as determined by both NGF rescue and immunocytochemistry. |A1:\*\*1SP3E3| |A2:\*\*1SP3E3| |A3:\*\*1SP3E1| |A4:\*\*1SP3E3| |A5:\*\*1GP3E1| |  |  |  |  |  |  |
|  |  |  |  | Annotation | 1SP3E3 | 1SP3E3 | 1SP3E1 | 1SP3E3 | 1GP3E1 |
|  |  |  |  | Evidence | 4 | 4 | 2 | 4 | 2 |
|  |  |  |  | Focus | 4 | 4 | 4 | 4 | 1 |
|  |  |  |  | Polarity | 7 | 7 | 7 | 7 | 7 |
| 457 | 8355 | 9584203\_287 | Tax coimmunoprecipitates with cyclin D1 and/or cyclin D3. |A1:\*\*1SP3E0| |A2:\*\*1SP3E0| |A3:\*\*1SP3E0| |A4:\*\*1SP3E0| |A5:\*\*1GP3E0| |  |  |  |  |  |  |
|  |  |  |  | Annotation | 1SP3E0 | 1SP3E0 | 1SP3E0 | 1SP3E0 | 1GP3E0 |
|  |  |  |  | Evidence | 1 | 1 | 1 | 1 | 1 |
|  |  |  |  | Focus | 4 | 4 | 4 | 4 | 1 |
|  |  |  |  | Polarity | 7 | 7 | 7 | 7 | 7 |
| 458 | 2446 | 12086872\_128 | The number of genes required for optimal class assignment varied between classes. |A1:\*\*1SP3E0| |A2:\*\*1SP3E0| |A3:\*\*1SP1E0| |A4:\*\*1SP2E0| |A5:\*\*1GP3E0| |  |  |  |  |  |  |
|  |  |  |  | Annotation | 1SP3E0 | 1SP3E0 | 1SP1E0 | 1SP2E0 | 1GP3E0 |
|  |  |  |  | Evidence | 1 | 1 | 1 | 1 | 1 |
|  |  |  |  | Focus | 4 | 4 | 4 | 4 | 1 |
|  |  |  |  | Polarity | 7 | 7 | 5 | 6 | 7 |
| 459 | 9114 | 11779869\_62 | The fact that the association of synaptophysin and dynamin is dependent on dissociation of synaptophysin and VAMP 2 is consistent with a role for the dynamin-synaptophysin complex in a step of the SV cycle after SNARE complex formation. |A1:\*\*1SP3E0| |A2:\*\*1SP3E1| |A3:\*\*1SP3E0| |A4:\*\*1SP3E0| |A5:\*\*1SP3E0| |  |  |  |  |  |  |
|  |  |  |  | Annotation | 1SP3E0 | 1SP3E1 | 1SP3E0 | 1SP3E0 | 1SP3E0 |
|  |  |  |  | Evidence | 1 | 2 | 1 | 1 | 1 |
|  |  |  |  | Focus | 4 | 4 | 4 | 4 | 4 |
|  |  |  |  | Polarity | 7 | 7 | 7 | 7 | 7 |
| 460 | 335 | 9660865\_84 | Intensities of signals were measured by NIH Image 1.58 software. |A1:\*\*1MP3E3| |A2:\*\*1MP3E1| |A3:\*\*1SP3E3| |A4:\*\*1SP3E3| |A5:\*\*1MP3E3| |  |  |  |  |  |  |
|  |  |  |  | Annotation | 1MP3E3 | 1MP3E1 | 1SP3E3 | 1SP3E3 | 1MP3E3 |
|  |  |  |  | Evidence | 4 | 2 | 4 | 4 | 4 |
|  |  |  |  | Focus | 2 | 2 | 4 | 4 | 2 |
|  |  |  |  | Polarity | 7 | 7 | 7 | 7 | 7 |
| 461 | 7675 | 10383396\_7 | Others have argued that Ins(1,3,4)P3 is converted to Ins(3,4,5,6)P4 by the sequential actions of Ins(1,3,4)P3 6-kinase, Ins(1,3,4,6)P4 1-phosphatase, and Ins(3,4,6)P3 5-kinase ( , , ). |A1:\*\*1SP2E2| |A2:\*\*1SP2E2| |A3:\*\*1SP1E1| |A4:\*\*1SP1E1| |A5:\*\*1SP3E2| |  |  |  |  |  |  |
|  |  |  |  | Annotation | 1SP2E2 | 1SP2E2 | 1SP1E1 | 1SP1E1 | 1SP3E2 |
|  |  |  |  | Evidence | 3 | 3 | 2 | 2 | 3 |
|  |  |  |  | Focus | 4 | 4 | 4 | 4 | 4 |
|  |  |  |  | Polarity | 6 | 6 | 5 | 5 | 7 |
| 462 | 1652 | 9736749\_63 | The second step of the transformation, performed in blocks of tissue containing the left and right hippocampus, was driven by individual voxel gray scale values but was constrained by fluid physical properties. |A1:\*\*1GP3E3| |A2:\*\*1SP3E1| |A3:\*\*1SP3E0| |A4:\*\*1SP3E0| |A5:\*\*1MP3E3| |  |  |  |  |  |  |
|  |  |  |  | Annotation | 1GP3E3 | 1SP3E1 | 1SP3E0 | 1SP3E0 | 1MP3E3 |
|  |  |  |  | Evidence | 4 | 2 | 1 | 1 | 4 |
|  |  |  |  | Focus | 1 | 4 | 4 | 4 | 2 |
|  |  |  |  | Polarity | 7 | 7 | 7 | 7 | 7 |
| 463 | 2059 | 12207891\_360 | N. Takahashi, S. Ishihara, S. Takada, S. Tsukita and A. Nagafuchi, Posttranscriptional regulation of -catenin expression is required for Wnt signaling in L cells. |A1:\*\*1SP3E3| |A2:\*\*1SP3E3| |A3:\*\*1SP3E0| |A4:\*\*1SP3E0| |A5:\*\*1SP3E2| |  |  |  |  |  |  |
|  |  |  |  | Annotation | 1SP3E3 | 1SP3E3 | 1SP3E0 | 1SP3E0 | 1SP3E2 |
|  |  |  |  | Evidence | 4 | 4 | 1 | 1 | 3 |
|  |  |  |  | Focus | 4 | 4 | 4 | 4 | 4 |
|  |  |  |  | Polarity | 7 | 7 | 7 | 7 | 7 |
| 464 | 6592 | 12668660\_20 | Mitochondrial control of apoptosis is governed by the BCL-2 family of proteins, which include anti-apoptotic BCL-2 and BCL-xL and pro-apoptotic BAX and BAK; |A3:\*\*1SP3E0| the balance between these opposing members is regulated by a third subgroup called the "`BH3-only"` proteins ( Cory and Adams, 2002). |A3:\*\*2SP3E2| |A1:\*\*1SP3E2| |A2:\*\*1SP3E2| |A4:\*\*1SP3E2| |A5:\*\*1SP3E2| |  |  |  |  |  |  |
|  |  |  |  | Annotation | 1SP3E2 1SP3E2 | 1SP3E2 1SP3E2 | 1SP3E0 2SP3E2 | 1SP3E2 1SP3E2 | 1SP3E2 1SP3E2 |
|  |  |  |  | Evidence | 3 3 | 3 3 | 1 3 | 3 3 | 3 3 |
|  |  |  |  | Focus | 4 4 | 4 4 | 4 4 | 4 4 | 4 4 |
|  |  |  |  | Polarity | 7 7 | 7 7 | 7 7 | 7 7 | 7 7 |
| 465 | 53 | 9336330\_116 | The mean maximal startle response to the 120 dB stimulus presented alone and when preceded by 70, 75 and 80 dB prepulse stimuli (in the aged animal experiment) or by 67, 70 and 75 dB (in the septal lesion experiments) was measured. |A1:\*\*1GP3E3| |A2:\*\*1MP3E3| |A3:\*\*1SP3E3| |A4:\*\*1SP3E0| |A5:\*\*1MP3E3| |  |  |  |  |  |  |
|  |  |  |  | Annotation | 1GP3E3 | 1MP3E3 | 1SP3E3 | 1SP3E0 | 1MP3E3 |
|  |  |  |  | Evidence | 4 | 4 | 4 | 1 | 4 |
|  |  |  |  | Focus | 1 | 2 | 4 | 4 | 2 |
|  |  |  |  | Polarity | 7 | 7 | 7 | 7 | 7 |
| 466 | 8611 | 10601345\_71 | The hybridization was carried out using an RPA kit (Ambion Inc.) and following the manufacturer's instructions. |A1:\*\*1SP3E3| |A2:\*\*1SP3E3| |A3:\*\*1MP3E3| |A4:\*\*1MP3E3| |A5:\*\*1MP3E3| |  |  |  |  |  |  |
|  |  |  |  | Annotation | 1SP3E3 | 1SP3E3 | 1MP3E3 | 1MP3E3 | 1MP3E3 |
|  |  |  |  | Evidence | 4 | 4 | 4 | 4 | 4 |
|  |  |  |  | Focus | 4 | 4 | 2 | 2 | 2 |
|  |  |  |  | Polarity | 7 | 7 | 7 | 7 | 7 |
| 467 | 4048 | 12711303\_82 | DCAP does not affect the canonical Wnt signal pathway |A1:\*\*1SN3E0| |A2:\*\*1SN3E0| |A3:\*\*1SN3E0| |A4:\*\*1SN3E0| |A5:\*\*1SN3E0| |  |  |  |  |  |  |
|  |  |  |  | Annotation | 1SN3E0 | 1SN3E0 | 1SN3E0 | 1SN3E0 | 1SN3E0 |
|  |  |  |  | Evidence | 1 | 1 | 1 | 1 | 1 |
|  |  |  |  | Focus | 4 | 4 | 4 | 4 | 4 |
|  |  |  |  | Polarity | 1 | 1 | 1 | 1 | 1 |
| 468 | 7840 | 10449458\_167 | Meanwhile, several groups had gained experience in methods for detection of rhinovirus by RT-PCR, and technological developments such as sample preparation kits and advanced microtiter-format heating blocks favored a switch from virus isolation to RNA detection by RT-PCR. |A1:\*\*1SP3E0| |A2:\*\*1SP3E1| |A3:\*\*1MP3E0| |A4:\*\*1MP3E0| |A5:\*\*1SP3E1| |  |  |  |  |  |  |
|  |  |  |  | Annotation | 1SP3E0 | 1SP3E1 | 1MP3E0 | 1MP3E0 | 1SP3E1 |
|  |  |  |  | Evidence | 1 | 2 | 1 | 1 | 2 |
|  |  |  |  | Focus | 4 | 4 | 2 | 2 | 4 |
|  |  |  |  | Polarity | 7 | 7 | 7 | 7 | 7 |
| 469 | 5438 | 10224275\_193 | Nuclei were stained for 15 min at room temperature with fresh 4'', 6-diamidinophenylindole 4'',6-diamidinophenylindole (DAPI) solution (1 mug/ml) in phosphate buffer, pH 7, 0.02% Tween-20 containing 0.1 mg/ml p-phenylene diamine as antifading agent. |A1:\*\*1MP3E3| |A2:\*\*1MP3E3| |A3:\*\*1MP3E3| |A4:\*\*1MP3E3| |A5:\*\*1MP3E3| |  |  |  |  |  |  |
|  |  |  |  | Annotation | 1MP3E3 | 1MP3E3 | 1MP3E3 | 1MP3E3 | 1MP3E3 |
|  |  |  |  | Evidence | 4 | 4 | 4 | 4 | 4 |
|  |  |  |  | Focus | 2 | 2 | 2 | 2 | 2 |
|  |  |  |  | Polarity | 7 | 7 | 7 | 7 | 7 |
| 470 | 9698 | 11689693\_158 | Interestingly, in more than 90% of transfected cells, GFP-KiD1 alone led to a twofold increase in actin stress fibers (Fig. 2F, a) |A1:\*\*1SP3E3+| |A2:\*\*1SP3E3+| while GFP-KiD2 alone triggered stress fiber disassembly and elongated cell morphology (Fig. 2F, b), |A1:\*\*2SP3E3| |A2:\*\*2SP3E1| |A3:\*\*1SP3E3+| |A4:\*\*1SP3E3+| |A5:\*\*1SP3E3+| which suggests that KiD1 and KiD2 also inhibit endogenous RhoG and RhoA proteins. |A1:\*\*3SN3E3| |A2:\*\*3SP3E1-| |A3:\*\*2SP2E0-| |A4:\*\*1SP2E0-| |A5:\*\*1SP2E3-| |  |  |  |  |  |  |
|  |  |  |  | Annotation | 1SP3E3+ 2SP3E3 3SN3E3 | 1SP3E3+ 2SP3E1 3SP3E1- | 1SP3E3+ 1SP3E3+ 2SP2E0- | 1SP3E3+ 1SP3E3+ 1SP2E0- | 1SP3E3+ 1SP3E3+ 1SP2E3- |
|  |  |  |  | Evidence | 4 4 4 | 4 2 2 | 4 4 1 | 4 4 1 | 4 4 4 |
|  |  |  |  | Focus | 4 4 4 | 4 4 4 | 4 4 4 | 4 4 4 | 4 4 4 |
|  |  |  |  | Polarity | 7 7 1 | 7 7 7 | 7 7 6 | 7 7 6 | 7 7 6 |
| 471 | 1909 | 12743027\_165 | GSK-3beta is a negative regulator of Wnt signalling (Cohen and Frame, 2001), \*1SN3E2 |A2:\*\*1SN3E2| |A3:\*\*1SP3E2| |A4:\*\*1SP3E2| which is inactivated by phosphorylation mediated by Wnt signals or ILK. |A1:\*\*2SN3E3| |A2:\*\*2SP3E0-| |A3:\*\*2SP3E2| |A4:\*\*2SP3E0| |A5:\*\*1SP3E2| |  |  |  |  |  |  |
|  |  |  |  | Annotation | 2SN3E3 2SN3E3 | 1SN3E2 2SP3E0- | 1SP3E2 2SP3E2 | 1SP3E2 2SP3E0 | 1SP3E2 1SP3E2 |
|  |  |  |  | Evidence | 4 4 | 3 1 | 3 3 | 3 1 | 3 3 |
|  |  |  |  | Focus | 4 4 | 4 4 | 4 4 | 4 4 | 4 4 |
|  |  |  |  | Polarity | 1 1 | 1 7 | 7 7 | 7 7 | 7 7 |
| 472 | 1117 | 9528791\_100 | Far-Western blot analysis was performed as described by Tarn et al. ( 51) |A3:\*\*1MP3E2| |A4:\*\*1MP3E2| except that 2 x 10^5^ to 4 x 10^5^ cpm of 35S-labeled Prp19p or Snt309p per ml was used in the incubation with blots. |A1:\*\*1MP3E23| |A2:\*\*1MP3E23| |A3:\*\*2MP3E0| |A4:\*\*2MP3E0| |A5:\*\*1MP3E3| |  |  |  |  |  |  |
|  |  |  |  | Annotation | 1MP3E23 1MP3E23 | 1MP3E23 1MP3E23 | 1MP3E2 2MP3E0 | 1MP3E2 2MP3E0 | 1MP3E3 1MP3E3 |
|  |  |  |  | Evidence | 3 3 | 3 3 | 3 1 | 3 1 | 4 4 |
|  |  |  |  | Focus | 2 2 | 2 2 | 2 2 | 2 2 | 2 2 |
|  |  |  |  | Polarity | 7 7 | 7 7 | 7 7 | 7 7 | 7 7 |
| 473 | 1405 | 9844016\_80 | In the schizophrenic cohort, there was a 21% decrease in layer I ( P = 0.510), a 34% decrease in layer II ( P = 0.046), a 33% decrease in layer III ( P = 0.040), a 29% decrease in layer IV ( P = 0.089), a 31% decrease in layer V ( P = 0.080), and a 29% decrease in layer VI ( P = 0.112). |A1:\*\*1SP3E3-| |A2:\*\*1SP3E3-| |A3:\*\*1SP3E3-| |A4:\*\*1SP3E0-| |A5:\*\*1SP3E1| |  |  |  |  |  |  |
|  |  |  |  | Annotation | 1SP3E3- | 1SP3E3- | 1SP3E3- | 1SP3E0- | 1SP3E1 |
|  |  |  |  | Evidence | 4 | 4 | 4 | 1 | 2 |
|  |  |  |  | Focus | 4 | 4 | 4 | 4 | 4 |
|  |  |  |  | Polarity | 7 | 7 | 7 | 7 | 7 |
| 474 | 1077 | 11923448\_21 | We explored whether this developmental disruption would, in turn, trigger behavioral changes similar to those observed in animals with the permanent excitotoxic lesion. |A1:\*\*1GP2E3| |A2:\*\*1GP2E3| |A3:\*\*1SP3E3| |A4:\*\*1SP3E3| |A5:\*\*1SP3E3| |  |  |  |  |  |  |
|  |  |  |  | Annotation | 1GP2E3 | 1GP2E3 | 1SP3E3 | 1SP3E3 | 1SP3E3 |
|  |  |  |  | Evidence | 4 | 4 | 4 | 4 | 4 |
|  |  |  |  | Focus | 1 | 1 | 4 | 4 | 4 |
|  |  |  |  | Polarity | 6 | 6 | 7 | 7 | 7 |
| 475 | 9932 | 9835621\_223 | A relationship between the generation of ROIs and NF-kappaB activation has been suggested in ceramide-induced apoptosis (9). |A1:\*\*1SP3E2| |A2:\*\*1SP3E2+| |A3:\*\*1SP2E2| |A4:\*\*1SP3E2| |A5:\*\*1SP3E2| |  |  |  |  |  |  |
|  |  |  |  | Annotation | 1SP3E2 | 1SP3E2+ | 1SP2E2 | 1SP3E2 | 1SP3E2 |
|  |  |  |  | Evidence | 3 | 3 | 3 | 3 | 3 |
|  |  |  |  | Focus | 4 | 4 | 4 | 4 | 4 |
|  |  |  |  | Polarity | 7 | 7 | 6 | 7 | 7 |
| 476 | 1155 | 12206470\_11 | Hormonal male contraception, with indirect suppression of spermatogenesis by decreasing gonadotrophin output, is a further choice. |A1:\*\*1SN2E0-| |A2:\*\*1SP1E0-| |A3:\*\*1SP3E0| |A4:\*\*1SP3E0| |A5:\*\*1GP3E0-| |  |  |  |  |  |  |
|  |  |  |  | Annotation | 1SN2E0- | 1SP1E0- | 1SP3E0 | 1SP3E0 | 1GP3E0- |
|  |  |  |  | Evidence | 1 | 1 | 1 | 1 | 1 |
|  |  |  |  | Focus | 4 | 4 | 4 | 4 | 1 |
|  |  |  |  | Polarity | 2 | 5 | 7 | 7 | 7 |
| 477 | 6193 | 9390512\_162 | The limitation of association studies is that candidate genes, as well as functional polymorphisms within these genes or at least polymorphisms in strong disequilibrium, must be identified first before the test can be performed. |A1:\*\*1GP2E0| |A2:\*\*1SP2E0| |A3:\*\*1SP3E0| |A4:\*\*1SP3E0| |A5:\*\*1SP3E0| |  |  |  |  |  |  |
|  |  |  |  | Annotation | 1GP2E0 | 1SP2E0 | 1SP3E0 | 1SP3E0 | 1SP3E0 |
|  |  |  |  | Evidence | 1 | 1 | 1 | 1 | 1 |
|  |  |  |  | Focus | 1 | 4 | 4 | 4 | 4 |
|  |  |  |  | Polarity | 6 | 6 | 7 | 7 | 7 |
| 478 | 6172 | 9316835\_74 | The reaction mixture was treated with 10% Na2CO3 and extracted twice with ethyl acetate. |A1:\*\*1MP3E3| |A2:\*\*1MP3E3| |A3:\*\*1MP3E3| |A4:\*\*1MP3E3| |A5:\*\*1MP3E3| |  |  |  |  |  |  |
|  |  |  |  | Annotation | 1MP3E3 | 1MP3E3 | 1MP3E3 | 1MP3E3 | 1MP3E3 |
|  |  |  |  | Evidence | 4 | 4 | 4 | 4 | 4 |
|  |  |  |  | Focus | 2 | 2 | 2 | 2 | 2 |
|  |  |  |  | Polarity | 7 | 7 | 7 | 7 | 7 |
| 479 | 982 | 11976265\_6 | The results reveal that beta-cells express Rhes |A3:\*\*1SP3E3| |A4:\*\*1SP3E3| |A5:\*\*1GP3E1| and suggest that changes in the expression of this molecule may regulate the sensitivity of beta-cells to imidazoline secretagogues. |A3:\*\*2SP2E1| |A1:\*\*1SP1E3| |A2:\*\*1SP1E3| |A4:\*\*2SP2E1| |A5:\*\*2GP2E1| |  |  |  |  |  |  |
|  |  |  |  | Annotation | 1SP1E3 1SP1E3 | 1SP1E3 1SP1E3 | 1SP3E3 2SP2E1 | 1SP3E3 2SP2E1 | 1GP3E1 2GP2E1 |
|  |  |  |  | Evidence | 4 4 | 4 4 | 4 2 | 4 2 | 2 2 |
|  |  |  |  | Focus | 4 4 | 4 4 | 4 4 | 4 4 | 1 1 |
|  |  |  |  | Polarity | 5 5 | 5 5 | 7 6 | 7 6 | 7 6 |
| 480 | 390 | 11163269\_276 | However, among sensory neurons of the L4 DRG, |A5:\*\*1SP3E0| ShcB / mice exhibit a significant reduction in the number of TrkA+ and IB4+ neurons and small-caliber axon fibers. |A5:\*\*2SP3E1-| |A1:\*\*1SP3E0| |A2:\*\*1SP3E0| |A3:\*\*1SP3E0-| |A4:\*\*1SP3E0-| |  |  |  |  |  |  |
|  |  |  |  | Annotation | 1SP3E0 1SP3E0 | 1SP3E0 1SP3E0 | 1SP3E0- 1SP3E0- | 1SP3E0- 1SP3E0- | 1SP3E0 2SP3E1- |
|  |  |  |  | Evidence | 1 1 | 1 1 | 1 1 | 1 1 | 1 2 |
|  |  |  |  | Focus | 4 4 | 4 4 | 4 4 | 4 4 | 4 4 |
|  |  |  |  | Polarity | 7 7 | 7 7 | 7 7 | 7 7 | 7 7 |
| 481 | 5445 | 10197998\_44 | The PCR fragment was digested with SalI and EcoRI and then ligated to pHY300 PLK that had been digested with SalI and EcoRI, yielding pHYG1. |A1:\*\*1SP3E3| |A2:\*\*1MP3E3| |A3:\*\*1MP3E3| |A4:\*\*1MP3E3| |A5:\*\*1MP3E3| |  |  |  |  |  |  |
|  |  |  |  | Annotation | 1SP3E3 | 1MP3E3 | 1MP3E3 | 1MP3E3 | 1MP3E3 |
|  |  |  |  | Evidence | 4 | 4 | 4 | 4 | 4 |
|  |  |  |  | Focus | 4 | 2 | 2 | 2 | 2 |
|  |  |  |  | Polarity | 7 | 7 | 7 | 7 | 7 |
| 482 | 370 | 10704443\_144 | We could also detect this cross-linked product in vesicles derived from emp24- E178A mutant (data not shown), confirming that vesicle budding occurred using these membranes. |A1:\*\*1SP3E1| |A2:\*\*1SP3E1| |A3:\*\*1SP3E3| |A4:\*\*1SP3E3| |A5:\*\*1SP3E3| |  |  |  |  |  |  |
|  |  |  |  | Annotation | 1SP3E1 | 1SP3E1 | 1SP3E3 | 1SP3E3 | 1SP3E3 |
|  |  |  |  | Evidence | 2 | 2 | 4 | 4 | 4 |
|  |  |  |  | Focus | 4 | 4 | 4 | 4 | 4 |
|  |  |  |  | Polarity | 7 | 7 | 7 | 7 | 7 |
| 483 | 4675 | 11526012\_71 | The column was equilibrated with 20 mM Tris-HCl (pH 8.0) containing 150 mM NaCl, and the separation was carried out isocratically at a flow rate of 0.4 ml/min. |A1:\*\*1MP3E3| |A2:\*\*1MP3E3| |A3:\*\*1MP3E3| |A4:\*\*1MP3E3| |A5:\*\*1MP3E3| |  |  |  |  |  |  |
|  |  |  |  | Annotation | 1MP3E3 | 1MP3E3 | 1MP3E3 | 1MP3E3 | 1MP3E3 |
|  |  |  |  | Evidence | 4 | 4 | 4 | 4 | 4 |
|  |  |  |  | Focus | 2 | 2 | 2 | 2 | 2 |
|  |  |  |  | Polarity | 7 | 7 | 7 | 7 | 7 |
| 484 | 4262 | 11238375\_1 | Small GTP-binding proteins of the Rho-family, Rho, Rac, and Cdc42, have been traditionally linked to the regulation of the cellular actin-based cytoskeleton. |A1:\*\*1SP3E1| |A2:\*\*1SP3E0| |A3:\*\*1SP3E0| |A4:\*\*1SP3E0| |A5:\*\*1SP3E0| |  |  |  |  |  |  |
|  |  |  |  | Annotation | 1SP3E1 | 1SP3E0 | 1SP3E0 | 1SP3E0 | 1SP3E0 |
|  |  |  |  | Evidence | 2 | 1 | 1 | 1 | 1 |
|  |  |  |  | Focus | 4 | 4 | 4 | 4 | 4 |
|  |  |  |  | Polarity | 7 | 7 | 7 | 7 | 7 |
| 485 | 4575 | 10207100\_114 | The result of a BLAST ( 1) search indicated that this sequence was identical to that of the gene encoding the TAFII40 protein ( 28). |A1:\*\*1SP3E23| |A2:\*\*1SP3E23| |A3:\*\*1SP3E2| |A4:\*\*1SP3E2| |A5:\*\*1SP3E2| |  |  |  |  |  |  |
|  |  |  |  | Annotation | 1SP3E23 | 1SP3E23 | 1SP3E2 | 1SP3E2 | 1SP3E2 |
|  |  |  |  | Evidence | 3 | 3 | 3 | 3 | 3 |
|  |  |  |  | Focus | 4 | 4 | 4 | 4 | 4 |
|  |  |  |  | Polarity | 7 | 7 | 7 | 7 | 7 |
| 486 | 3850 | 8756721\_235 | Assay is described in detail elsewhere (van de Wetering et al., 1991). |A1:\*\*1MP3E2| |A2:\*\*1SP3E2| |A3:\*\*1SP3E2| |A4:\*\*1SP3E2| |A5:\*\*1MP3E2| |  |  |  |  |  |  |
|  |  |  |  | Annotation | 1MP3E2 | 1SP3E2 | 1SP3E2 | 1SP3E2 | 1MP3E2 |
|  |  |  |  | Evidence | 3 | 3 | 3 | 3 | 3 |
|  |  |  |  | Focus | 2 | 4 | 4 | 4 | 2 |
|  |  |  |  | Polarity | 7 | 7 | 7 | 7 | 7 |
| 487 | 2507 | 10619028\_478 | We thank R. Jain and C. Nassif for help with sequencing and cloning. |A1:\*\*1GP3E0| |A2:\*\*1GP3E3| |A3:\*\*1SP3E3| |A4:\*\*1GP3E3| |A5:\*\*1GP3E0| |  |  |  |  |  |  |
|  |  |  |  | Annotation | 1GP3E0 | 1GP3E3 | 1SP3E3 | 1GP3E3 | 1GP3E0 |
|  |  |  |  | Evidence | 1 | 4 | 4 | 4 | 1 |
|  |  |  |  | Focus | 1 | 1 | 4 | 1 | 1 |
|  |  |  |  | Polarity | 7 | 7 | 7 | 7 | 7 |
| 488 | 4389 | 8598046\_323 | Flies bearing new insertions have two-toned eyes, with lighter lower halves. |A1:\*\*1GP3E0| |A2:\*\*1SP3E0| |A3:\*\*1SP3E0| |A4:\*\*1GP3E0| |A5:\*\*1SP3E1| |  |  |  |  |  |  |
|  |  |  |  | Annotation | 1GP3E0 | 1SP3E0 | 1SP3E0 | 1GP3E0 | 1SP3E1 |
|  |  |  |  | Evidence | 1 | 1 | 1 | 1 | 2 |
|  |  |  |  | Focus | 1 | 4 | 4 | 1 | 4 |
|  |  |  |  | Polarity | 7 | 7 | 7 | 7 | 7 |
| 489 | 3344 | 12061758\_4 | In Experiments 2B and 2C, we manipulated the amount of weight that people simulated lifting in order to address potential alternative explanations of the inflation effect. |A1:\*\*1GP3E3| |A2:\*\*1GP3E3| |A3:\*\*1GP3E3| |A4:\*\*1GP3E3| |A5:\*\*1GP3E3| |  |  |  |  |  |  |
|  |  |  |  | Annotation | 1GP3E3 | 1GP3E3 | 1GP3E3 | 1GP3E3 | 1GP3E3 |
|  |  |  |  | Evidence | 4 | 4 | 4 | 4 | 4 |
|  |  |  |  | Focus | 1 | 1 | 1 | 1 | 1 |
|  |  |  |  | Polarity | 7 | 7 | 7 | 7 | 7 |
| 490 | 927 | 12037584\_7 | In contrast, resident macrophages from the peritoneum showed the expected NO response, |A5:\*\*1SP3E3| and purified Leydig cells produced significant NO regardless of the presence or absence of LPS. |A5:\*\*2SP3E3+| |A1:\*\*1SP3E3| |A2:\*\*1SP3E3| |A3:\*\*1SN3E3| |A4:\*\*1SN3E0| |  |  |  |  |  |  |
|  |  |  |  | Annotation | 1SP3E3 1SP3E3 | 1SP3E3 1SP3E3 | 1SN3E3 1SN3E3 | 1SN3E0 1SN3E0 | 1SP3E3 2SP3E3+ |
|  |  |  |  | Evidence | 4 4 | 4 4 | 4 4 | 1 1 | 4 4 |
|  |  |  |  | Focus | 4 4 | 4 4 | 4 4 | 4 4 | 4 4 |
|  |  |  |  | Polarity | 7 7 | 7 7 | 1 1 | 1 1 | 7 7 |
| 491 | 3808 | 11997515\_561 | Axin, a negative regulator of the Wnt signaling pathway, |A1:\*\*1SP3E0| directly interacts with Adenomatous Polyposis Coli and regulates the stabilization of beta-catenin. |A1:\*\*1SP3E0| |A2:\*\*1SP3E0| |A3:\*\*1SP3E0| |A4:\*\*1SP3E0| |A5:\*\*1SP3E0| |  |  |  |  |  |  |
|  |  |  |  | Annotation | 1SP3E0 1SP3E0 | 1SP3E0 1SP3E0 | 1SP3E0 1SP3E0 | 1SP3E0 1SP3E0 | 1SP3E0 1SP3E0 |
|  |  |  |  | Evidence | 1 1 | 1 1 | 1 1 | 1 1 | 1 1 |
|  |  |  |  | Focus | 4 4 | 4 4 | 4 4 | 4 4 | 4 4 |
|  |  |  |  | Polarity | 7 7 | 7 7 | 7 7 | 7 7 | 7 7 |
| 492 | 2910 | 11313474\_245 | Thus it is unlikely that the mRNA transport and alternative splicing activities of hnRNPA1 are involved in the ability of hnRNPA1 to influence NF-kappaB-dependent transcription. |A1:\*\*1SP3E1| |A2:\*\*1SN2E1| |A3:\*\*1SP1E0| |A4:\*\*1SP3E0| |A5:\*\*1SN3E0| |  |  |  |  |  |  |
|  |  |  |  | Annotation | 1SP3E1 | 1SN2E1 | 1SP1E0 | 1SP3E0 | 1SN3E0 |
|  |  |  |  | Evidence | 2 | 2 | 1 | 1 | 1 |
|  |  |  |  | Focus | 4 | 4 | 4 | 4 | 4 |
|  |  |  |  | Polarity | 7 | 2 | 5 | 7 | 1 |
| 493 | 7647 | 11960452\_9 | The structure of the "molecular cap" with its disrupted terminal base pair |A5:\*\*1SP3E0| may also be helpful for modeling how quinolones block re-ligation of DNA strands in the active site of gyrases. |A5:\*\*2SP2E0-| |A1:\*\*1SP1E0| |A2:\*\*1SP1E0| |A3:\*\*1SP2E0| |A4:\*\*1SP2E0| |  |  |  |  |  |  |
|  |  |  |  | Annotation | 1SP1E0 1SP1E0 | 1SP1E0 1SP1E0 | 1SP2E0 1SP2E0 | 1SP2E0 1SP2E0 | 1SP3E0 2SP2E0- |
|  |  |  |  | Evidence | 1 1 | 1 1 | 1 1 | 1 1 | 1 1 |
|  |  |  |  | Focus | 4 4 | 4 4 | 4 4 | 4 4 | 4 4 |
|  |  |  |  | Polarity | 5 5 | 5 5 | 6 6 | 6 6 | 7 6 |
| 494 | 97 | 10102275\_203 | There is a poor structural correspondence between Kir 2.1 and KcsA in the residues at the M2/M2 intersubunit interface and the pore-lining residues. |A1:\*\*1GP3E0| |A2:\*\*1SP3E0| |A3:\*\*1SP3E0| |A4:\*\*1SP3E0| |A5:\*\*1SP3E0| |  |  |  |  |  |  |
|  |  |  |  | Annotation | 1GP3E0 | 1SP3E0 | 1SP3E0 | 1SP3E0 | 1SP3E0 |
|  |  |  |  | Evidence | 1 | 1 | 1 | 1 | 1 |
|  |  |  |  | Focus | 1 | 4 | 4 | 4 | 4 |
|  |  |  |  | Polarity | 7 | 7 | 7 | 7 | 7 |
| 495 | 3061 | 7721857\_3 | From this screen 28 independent plasmids were isolated and grouped into three families on the basis of Southern analysis. |A1:\*\*1MSP3E3| |A2:\*\*1MP3E3| |A3:\*\*1MSP3E3| |A4:\*\*1MP3E3| |A5:\*\*1MP3E3| |  |  |  |  |  |  |
|  |  |  |  | Annotation | 1MSP3E3 | 1MP3E3 | 1MSP3E3 | 1MP3E3 | 1MP3E3 |
|  |  |  |  | Evidence | 4 | 4 | 4 | 4 | 4 |
|  |  |  |  | Focus | 6 | 2 | 6 | 2 | 2 |
|  |  |  |  | Polarity | 7 | 7 | 7 | 7 | 7 |
| 496 | 8118 | 10205165\_240 | This implies that alphavbeta3-dependent cell migration is co-ordinated with cell proliferation, which is interesting since alphavbeta3 plays an important role in regulating both intra- and extracellular activities that are involved in promoting cell migration and cell growth. |A1:\*\*1SP3E3| |A2:\*\*1SP3E1| |A3:\*\*1SP3E0| |A4:\*\*1SP3E0| |A5:\*\*1SP3E0| |  |  |  |  |  |  |
|  |  |  |  | Annotation | 1SP3E3 | 1SP3E1 | 1SP3E0 | 1SP3E0 | 1SP3E0 |
|  |  |  |  | Evidence | 4 | 2 | 1 | 1 | 1 |
|  |  |  |  | Focus | 4 | 4 | 4 | 4 | 4 |
|  |  |  |  | Polarity | 7 | 7 | 7 | 7 | 7 |
| 497 | 2654 | 11713294\_281 | Formation of these complexes required that responding B cells express the ITIM-bearing FcgammaRII. |A1:\*\*1SP3E0| |A2:\*\*1SP3E0| |A3:\*\*1SP3E0| |A4:\*\*1SP3E0| |A5:\*\*1SP3E0| |  |  |  |  |  |  |
|  |  |  |  | Annotation | 1SP3E0 | 1SP3E0 | 1SP3E0 | 1SP3E0 | 1SP3E0 |
|  |  |  |  | Evidence | 1 | 1 | 1 | 1 | 1 |
|  |  |  |  | Focus | 4 | 4 | 4 | 4 | 4 |
|  |  |  |  | Polarity | 7 | 7 | 7 | 7 | 7 |
| 498 | 7409 | 9020177\_12 | DTP overexpression is demonstrated by the SDS-PAGE profile, depicted in Fig. |A1:\*\*1SP3E3+| |A2:\*\*1SP3E3| |A3:\*\*1MP3E3| |A4:\*\*1SP3E3+| |A5:\*\*1SP3E3+| |  |  |  |  |  |  |
|  |  |  |  | Annotation | 1SP3E3+ | 1SP3E3 | 1MP3E3 | 1SP3E3+ | 1SP3E3+ |
|  |  |  |  | Evidence | 4 | 4 | 4 | 4 | 4 |
|  |  |  |  | Focus | 4 | 4 | 2 | 4 | 4 |
|  |  |  |  | Polarity | 7 | 7 | 7 | 7 | 7 |
| 499 | 8412 | 8702594\_44 | The low field region of the 1H spectrum (5.8-4.4 ppm) was complex indicating the sample to be a mixture of two related decasaccharides. |A1:\*\*1SP3E3| |A2:\*\*1SP3E3| |A3:\*\*1SP3E0| |A4:\*\*1SP3E0| |A5:\*\*1SMP3E3| |  |  |  |  |  |  |
|  |  |  |  | Annotation | 1SP3E3 | 1SP3E3 | 1SP3E0 | 1SP3E0 | 1SMP3E3 |
|  |  |  |  | Evidence | 4 | 4 | 1 | 1 | 4 |
|  |  |  |  | Focus | 4 | 4 | 4 | 4 | 6 |
|  |  |  |  | Polarity | 7 | 7 | 7 | 7 | 7 |
| 500 | 158 | 10207091\_43 | Briefly, the phosphorothioate-containing oligonucleotides were 5''-end labeled with 32P by using T4 polynucleotide kinase and annealed to a fivefold excess of its unlabeled complement. |A1:\*\*1SP3E3| |A2:\*\*1MP3E3| |A3:\*\*1MP3E3| |A4:\*\*1MP3E3| |A5:\*\*1MP3E3| |  |  |  |  |  |  |
|  |  |  |  | Annotation | 1SP3E3 | 1MP3E3 | 1MP3E3 | 1MP3E3 | 1MP3E3 |
|  |  |  |  | Evidence | 4 | 4 | 4 | 4 | 4 |
|  |  |  |  | Focus | 4 | 2 | 2 | 2 | 2 |
|  |  |  |  | Polarity | 7 | 7 | 7 | 7 | 7 |
| 501 | 7012 | 7721857\_41 | Thus, the cross-linking of CDC34 to itself reflects a specific set of interactions between CDC34 monomers. |A1:\*\*1SP3E0| |A2:\*\*1SP1E1| |A3:\*\*1SP3E0| |A4:\*\*1SP3E0| |A5:\*\*1SP3E1| |  |  |  |  |  |  |
|  |  |  |  | Annotation | 1SP3E0 | 1SP1E1 | 1SP3E0 | 1SP3E0 | 1SP3E1 |
|  |  |  |  | Evidence | 1 | 2 | 1 | 1 | 2 |
|  |  |  |  | Focus | 4 | 4 | 4 | 4 | 4 |
|  |  |  |  | Polarity | 7 | 5 | 7 | 7 | 7 |
| 502 | 2263 | 10611225\_100 | However, deletion of the eIF4E binding site markedly (80%) reduced cap-dependent 40S ribosomal binding (compare lanes 5 and 6). |A1:\*\*1SP3E3-| |A2:\*\*1SP3E3-| |A3:\*\*1SP3E3-| |A4:\*\*1SP3E3-| |A5:\*\*1SP3E3-| |  |  |  |  |  |  |
|  |  |  |  | Annotation | 1SP3E3- | 1SP3E3- | 1SP3E3- | 1SP3E3- | 1SP3E3- |
|  |  |  |  | Evidence | 4 | 4 | 4 | 4 | 4 |
|  |  |  |  | Focus | 4 | 4 | 4 | 4 | 4 |
|  |  |  |  | Polarity | 7 | 7 | 7 | 7 | 7 |
| 503 | 8241 | 12184965\_6 | RESULTS: Activities of daily living (ADL) dependency (odds ratio [OR] = 5.3, P =.03), years since symptoms first started (OR = 1.84, P =.03), and the presence of somatic comorbidity (OR = 0.48, P =.02) independently contributed to the prediction of the presence or absence of dementia. |A1:\*\*1GP3E3| |A2:\*\*1SP3E3| |A3:\*\*1SP3E3| |A4:\*\*1SP3E3| |A5:\*\*1SP3E1| |  |  |  |  |  |  |
|  |  |  |  | Annotation | 1GP3E3 | 1SP3E3 | 1SP3E3 | 1SP3E3 | 1SP3E1 |
|  |  |  |  | Evidence | 4 | 4 | 4 | 4 | 2 |
|  |  |  |  | Focus | 1 | 4 | 4 | 4 | 4 |
|  |  |  |  | Polarity | 7 | 7 | 7 | 7 | 7 |
| 504 | 3040 | 9802880\_25 | Nonetheless, previous work has established that these cancers overexpress many mitogenic growth factors and their receptors ( 23) |A4:\*\*1SP3E2+| |A5:\*\*1SP3E2+| including a number of heparin-binding growth factors such as FGF1, FGF2, FGF5, HB-EGF, and amphiregulin ( 24-27). |A4:\*\*2SP3E0| |A1:\*\*1SP3E2| |A2:\*\*1SP3E2| |A3:\*\*1SP3E2+| |A5:\*\*2SP3E2| |  |  |  |  |  |  |
|  |  |  |  | Annotation | 1SP3E2 1SP3E2 | 1SP3E2 1SP3E2 | 1SP3E2+ 1SP3E2+ | 1SP3E2+ 2SP3E0 | 1SP3E2+ 2SP3E2 |
|  |  |  |  | Evidence | 3 3 | 3 3 | 3 3 | 3 1 | 3 3 |
|  |  |  |  | Focus | 4 4 | 4 4 | 4 4 | 4 4 | 4 4 |
|  |  |  |  | Polarity | 7 7 | 7 7 | 7 7 | 7 7 | 7 7 |
| 505 | 4731 | 12718879\_139 | Reactions containing inhibitors (lanes 5-7) were incubated for 30 min. |A1:\*\*1MP3E3| |A2:\*\*1MP3E3| |A3:\*\*1SP3E3| |A4:\*\*1MP3E3| |A5:\*\*1MP3E3| |  |  |  |  |  |  |
|  |  |  |  | Annotation | 1MP3E3 | 1MP3E3 | 1SP3E3 | 1MP3E3 | 1MP3E3 |
|  |  |  |  | Evidence | 4 | 4 | 4 | 4 | 4 |
|  |  |  |  | Focus | 2 | 2 | 4 | 2 | 2 |
|  |  |  |  | Polarity | 7 | 7 | 7 | 7 | 7 |
| 506 | 6421 | 12000196\_5 | The mutation was found in an Italian family with 5 patients over 3 generations. |A1:\*\*1GP3E3| |A2:\*\*1SP3E3| |A3:\*\*1SP3E1| |A4:\*\*1SP3E0| |A5:\*\*1SP3E3| |  |  |  |  |  |  |
|  |  |  |  | Annotation | 1GP3E3 | 1SP3E3 | 1SP3E1 | 1SP3E0 | 1SP3E3 |
|  |  |  |  | Evidence | 4 | 4 | 2 | 1 | 4 |
|  |  |  |  | Focus | 1 | 4 | 4 | 4 | 4 |
|  |  |  |  | Polarity | 7 | 7 | 7 | 7 | 7 |
| 507 | 6558 | 9390512\_143 | Interestingly, the lod score increased to 2.6 when individuals with affective disorders (bipolar, major depression) were included in the analyses. |A1:\*\*1GP3E3+| |A2:\*\*1SP3E3+| |A3:\*\*1SP3E3+| |A4:\*\*1SP3E0+| |A5:\*\*1SP3E3| |  |  |  |  |  |  |
|  |  |  |  | Annotation | 1GP3E3+ | 1SP3E3+ | 1SP3E3+ | 1SP3E0+ | 1SP3E3 |
|  |  |  |  | Evidence | 4 | 4 | 4 | 1 | 4 |
|  |  |  |  | Focus | 1 | 4 | 4 | 4 | 4 |
|  |  |  |  | Polarity | 7 | 7 | 7 | 7 | 7 |
| 508 | 7071 | 9242700\_12 | These RNAs were capable of hybridizing with globin mRNA (,). |A1:\*\*1SP3E23| |A2:\*\*1SP3E23| |A3:\*\*1SP3E1| |A4:\*\*1SP3E0| |A5:\*\*1SP3E2| |  |  |  |  |  |  |
|  |  |  |  | Annotation | 1SP3E23 | 1SP3E23 | 1SP3E1 | 1SP3E0 | 1SP3E2 |
|  |  |  |  | Evidence | 3 | 3 | 2 | 1 | 3 |
|  |  |  |  | Focus | 4 | 4 | 4 | 4 | 4 |
|  |  |  |  | Polarity | 7 | 7 | 7 | 7 | 7 |
| 509 | 2198 | 9545294\_157 | The spectrum of oxidized XylT exhibited three maxima due to the chromophore at 336, 416, and 456 nm, as well as a shoulder near 540 nm. |A1:\*\*1SP3E0| |A2:\*\*1SP2E3| |A3:\*\*1SP3E3| |A4:\*\*1SP3E3| |A5:\*\*1MP3E3| |  |  |  |  |  |  |
|  |  |  |  | Annotation | 1SP3E0 | 1SP2E3 | 1SP3E3 | 1SP3E3 | 1MP3E3 |
|  |  |  |  | Evidence | 1 | 4 | 4 | 4 | 4 |
|  |  |  |  | Focus | 4 | 4 | 4 | 4 | 2 |
|  |  |  |  | Polarity | 7 | 6 | 7 | 7 | 7 |
| 510 | 9714 | 10821833\_31 | We note that an intense DNase I cut site, but not hydroxyl radical cut site, also occurs at 31, being strong in both naked 5 S rDNA and the 5 S nucleosome ( star in Fig. 2 B, lanes 5-8). |A1:\*\*1SP3E3| |A2:\*\*1SP3E3| |A3:\*\*1SN3E3| |A4:\*\*1SN3E3| |A5:\*\*1MP3E3| |  |  |  |  |  |  |
|  |  |  |  | Annotation | 1SP3E3 | 1SP3E3 | 1SN3E3 | 1SN3E3 | 1MP3E3 |
|  |  |  |  | Evidence | 4 | 4 | 4 | 4 | 4 |
|  |  |  |  | Focus | 4 | 4 | 4 | 4 | 2 |
|  |  |  |  | Polarity | 7 | 7 | 1 | 1 | 7 |
| 511 | 4478 | 11238208\_115 | In summary, transcript quantification by LightCycler can be successfully applied to gain insight into temporal bacterial gene expression in vivo if performed carefully. |A1:\*\*1SP2E0| |A2:\*\*1SP2E0| |A3:\*\*1SP3E0| |A4:\*\*1MP3E3| |A5:\*\*1SMP3E3| |  |  |  |  |  |  |
|  |  |  |  | Annotation | 1SP2E0 | 1SP2E0 | 1SP3E0 | 1MP3E3 | 1SMP3E3 |
|  |  |  |  | Evidence | 1 | 1 | 1 | 4 | 4 |
|  |  |  |  | Focus | 4 | 4 | 4 | 2 | 6 |
|  |  |  |  | Polarity | 6 | 6 | 7 | 7 | 7 |
| 512 | 6706 | 9585507\_83 | In posterior Ubx clones in the haltere disc, Wg is expressed along the DV boundary (Fig. 3C), |A2:\*\*1SP3E2| |A3:\*\*1SP3E3| |A4:\*\*1SP3E3| |A5:\*\*1SP3E3+| suggesting that Ubx represses the posterior portion of the Wg expression pattern. |A2:\*\*1SP3E3-| |A1:\*\*1SN3E3| |A3:\*\*2SP3E1-| |A4:\*\*2SP3E1-| |A5:\*\*2SP2E3-| |  |  |  |  |  |  |
|  |  |  |  | Annotation | 1SN3E3 1SN3E3 | 1SP3E2 1SP3E3- | 1SP3E3 2SP3E1- | 1SP3E3 2SP3E1- | 1SP3E3+ 2SP2E3- |
|  |  |  |  | Evidence | 4 4 | 3 4 | 4 2 | 4 2 | 4 4 |
|  |  |  |  | Focus | 4 4 | 4 4 | 4 4 | 4 4 | 4 4 |
|  |  |  |  | Polarity | 1 1 | 7 7 | 7 7 | 7 7 | 7 6 |
| 513 | 5476 | 10768944\_173 | Similar results have been obtained in rhesus monkeys (24) and in mice (11). |A1:\*\*1SP3E2| |A2:\*\*1SP3E2| |A3:\*\*1SP3E2| |A4:\*\*1SP3E2| |A5:\*\*1GP3E2| |  |  |  |  |  |  |
|  |  |  |  | Annotation | 1SP3E2 | 1SP3E2 | 1SP3E2 | 1SP3E2 | 1GP3E2 |
|  |  |  |  | Evidence | 3 | 3 | 3 | 3 | 3 |
|  |  |  |  | Focus | 4 | 4 | 4 | 4 | 1 |
|  |  |  |  | Polarity | 7 | 7 | 7 | 7 | 7 |
| 514 | 7379 | 12230117\_1 | Oral administration of the perilla leaf extract (PLE) to mice inhibits inflammation, allergic response, and tumor necrosis factor-alpha production. |A1:\*\*1GN3E0| |A2:\*\*1SP3E0-| |A3:\*\*1SP3E1-| |A4:\*\*1SP3E0-| |A5:\*\*1SP3E0-| |  |  |  |  |  |  |
|  |  |  |  | Annotation | 1GN3E0 | 1SP3E0- | 1SP3E1- | 1SP3E0- | 1SP3E0- |
|  |  |  |  | Evidence | 1 | 1 | 2 | 1 | 1 |
|  |  |  |  | Focus | 1 | 4 | 4 | 4 | 4 |
|  |  |  |  | Polarity | 1 | 7 | 7 | 7 | 7 |
| 515 | 5696 | 11238388\_271 | The collection of A-clones in the A. nidulans project mapped to the centromeres (P RADE et al. 1997 ). |A1:\*\*1SP3E2| |A2:\*\*1SP3E2| |A3:\*\*1SP3E2| |A4:\*\*1SP3E2| |A5:\*\*1SP3E2| |  |  |  |  |  |  |
|  |  |  |  | Annotation | 1SP3E2 | 1SP3E2 | 1SP3E2 | 1SP3E2 | 1SP3E2 |
|  |  |  |  | Evidence | 3 | 3 | 3 | 3 | 3 |
|  |  |  |  | Focus | 4 | 4 | 4 | 4 | 4 |
|  |  |  |  | Polarity | 7 | 7 | 7 | 7 | 7 |
| 516 | 7306 | 9797273\_90 | Membranes were incubated in a renaturation buffer (0.1 M Tris-HCl [pH 7.0], 2% Triton X-100 [28]) for 30 min. |A1:\*\*1MP3E3| |A2:\*\*1MP3E3| |A3:\*\*1MP3E3| |A4:\*\*1MP3E3| |A5:\*\*1MP3E3| |  |  |  |  |  |  |
|  |  |  |  | Annotation | 1MP3E3 | 1MP3E3 | 1MP3E3 | 1MP3E3 | 1MP3E3 |
|  |  |  |  | Evidence | 4 | 4 | 4 | 4 | 4 |
|  |  |  |  | Focus | 2 | 2 | 2 | 2 | 2 |
|  |  |  |  | Polarity | 7 | 7 | 7 | 7 | 7 |
| 517 | 9267 | 9463381\_233 | Briefly, lysates prepared from 1-2x10^6^ cells were incubated with ~5 mug primary antibody followed by protein G- or protein A-Sepharose (Pharmacia) and then pelleted, washed, fractionated by SDS-PAGE and transferred to nitrocellulose membranes by electroblotting. |A1:\*\*1MP3E3| |A2:\*\*1MP3E3| |A3:\*\*1MP3E3| |A4:\*\*1MP3E3| |A5:\*\*1MP3E3| |  |  |  |  |  |  |
|  |  |  |  | Annotation | 1MP3E3 | 1MP3E3 | 1MP3E3 | 1MP3E3 | 1MP3E3 |
|  |  |  |  | Evidence | 4 | 4 | 4 | 4 | 4 |
|  |  |  |  | Focus | 2 | 2 | 2 | 2 | 2 |
|  |  |  |  | Polarity | 7 | 7 | 7 | 7 | 7 |
| 518 | 5798 | 12224816\_5 | After adjustment for age, gender, diabetes mellitus, hypertension, smoking, body mass index, total cholesterol, and high density lipoprotein cholesterol, the odds ratio for acute myocardial infarction was 2.97 (95% confidence interval, 1.37-6.41; |A1:\*\*1SP3E3| |A2:\*\*1SP3E3| |A3:\*\*1SP3E3| |A4:\*\*1SP3E3| |A5:\*\*1SP3E3| |  |  |  |  |  |  |
|  |  |  |  | Annotation | 1SP3E3 | 1SP3E3 | 1SP3E3 | 1SP3E3 | 1SP3E3 |
|  |  |  |  | Evidence | 4 | 4 | 4 | 4 | 4 |
|  |  |  |  | Focus | 4 | 4 | 4 | 4 | 4 |
|  |  |  |  | Polarity | 7 | 7 | 7 | 7 | 7 |
| 519 | 1121 | 12034774\_21 | The RhoA - actin signaling pathway regulates a subset of SRF target genes including vinculin, beta-actin, SRF itself, SM22, and sm-alpha actin ( Sotiropoulos et al., 1999; Gineitis and Treisman, 2001; Mack et al., 2001). |A1:\*\*1SP3E2| |A2:\*\*1SP3E2| |A3:\*\*1SP3E2| |A4:\*\*1SP3E2| |A5:\*\*1SP3E2| |  |  |  |  |  |  |
|  |  |  |  | Annotation | 1SP3E2 | 1SP3E2 | 1SP3E2 | 1SP3E2 | 1SP3E2 |
|  |  |  |  | Evidence | 3 | 3 | 3 | 3 | 3 |
|  |  |  |  | Focus | 4 | 4 | 4 | 4 | 4 |
|  |  |  |  | Polarity | 7 | 7 | 7 | 7 | 7 |
| 520 | 6800 | 9139723\_91 | C, AtT-20/PAM-1 cells expressing P-CIP10 were incubated in medium containing [35S]Met/Cys for 15 min ( P) and chased for 1, 2, or 4 h before immunoprecipitation of P-CIP10. |A1:\*\*1MP3E3| |A2:\*\*1MP3E3| |A3:\*\*1MP3E3| |A4:\*\*1MP3E3| |A5:\*\*1MP3E3| |  |  |  |  |  |  |
|  |  |  |  | Annotation | 1MP3E3 | 1MP3E3 | 1MP3E3 | 1MP3E3 | 1MP3E3 |
|  |  |  |  | Evidence | 4 | 4 | 4 | 4 | 4 |
|  |  |  |  | Focus | 2 | 2 | 2 | 2 | 2 |
|  |  |  |  | Polarity | 7 | 7 | 7 | 7 | 7 |
| 521 | 9602 | 9732415\_78 | Cytochrome P450 content was determined as described by Omura and Sato (1964). |A1:\*\*1SP3E23| |A2:\*\*1SP3E23| |A3:\*\*1SP3E1| |A4:\*\*1SP3E2| |A5:\*\*1GP3E2| |  |  |  |  |  |  |
|  |  |  |  | Annotation | 1SP3E23 | 1SP3E23 | 1SP3E1 | 1SP3E2 | 1GP3E2 |
|  |  |  |  | Evidence | 3 | 3 | 2 | 3 | 3 |
|  |  |  |  | Focus | 4 | 4 | 4 | 4 | 1 |
|  |  |  |  | Polarity | 7 | 7 | 7 | 7 | 7 |
| 522 | 4112 | 9636222\_165 | Although PC-PLC is involved in several models of programmed cell death, PC-PLC regulates glutamate-induced nerve cell death through a different mechanism. |A1:\*\*1SP3E0| |A2:\*\*1SP3E0| |A3:\*\*1SP3E0| |A4:\*\*1SP3E0| |A5:\*\*1SP3E0| |  |  |  |  |  |  |
|  |  |  |  | Annotation | 1SP3E0 | 1SP3E0 | 1SP3E0 | 1SP3E0 | 1SP3E0 |
|  |  |  |  | Evidence | 1 | 1 | 1 | 1 | 1 |
|  |  |  |  | Focus | 4 | 4 | 4 | 4 | 4 |
|  |  |  |  | Polarity | 7 | 7 | 7 | 7 | 7 |
| 523 | 9454 | 9742132\_201 | Moreover, they demonstrate that ribose methylation of the injected cyclin B1 RNA is very inefficient |A5:\*\*1SN3E1| and appears not to require polyadenylation. |A5:\*\*2SN2E1| |A1:\*\*1SP3E1| |A2:\*\*1SP3E1| |A3:\*\*1SN2E3| |A4:\*\*1SN2E3| |  |  |  |  |  |  |
|  |  |  |  | Annotation | 1SP3E1 1SP3E1 | 1SP3E1 1SP3E1 | 1SN2E3 1SN2E3 | 1SN2E3 1SN2E3 | 1SN3E1 2SN2E1 |
|  |  |  |  | Evidence | 2 2 | 2 2 | 4 4 | 4 4 | 2 2 |
|  |  |  |  | Focus | 4 4 | 4 4 | 4 4 | 4 4 | 4 4 |
|  |  |  |  | Polarity | 7 7 | 7 7 | 2 2 | 2 2 | 1 2 |
| 524 | 644 | 11502253\_334 | Further analysis of the function and regulation of Ngn2 and other bHLH genes will shed more light |A5:\*\*1SP3E0| on how these genes may act to synchronize genetic pathways that specify neuronal identity with the progression of neural lineage development. |A5:\*\*2SP0E0| |A1:\*\*1SP1E0| |A2:\*\*1SP1E1| |A3:\*\*1SP3E0| |A4:\*\*1SP2E3| |  |  |  |  |  |  |
|  |  |  |  | Annotation | 1SP1E0 1SP1E0 | 1SP1E1 1SP1E1 | 1SP3E0 1SP3E0 | 1SP2E3 1SP2E3 | 1SP3E0 2SP0E0 |
|  |  |  |  | Evidence | 1 1 | 2 2 | 1 1 | 4 4 | 1 1 |
|  |  |  |  | Focus | 4 4 | 4 4 | 4 4 | 4 4 | 4 4 |
|  |  |  |  | Polarity | 5 5 | 5 5 | 7 7 | 6 6 | 7 4 |
| 525 | 7056 | 9208845\_330 | Positive cultures were subcloned twice by limiting dilution in 96-well plates. |A1:\*\*1MP3E3| |A2:\*\*1MP3E3| |A3:\*\*1MP3E3| |A4:\*\*1MP3E3| |A5:\*\*1MP3E3| |  |  |  |  |  |  |
|  |  |  |  | Annotation | 1MP3E3 | 1MP3E3 | 1MP3E3 | 1MP3E3 | 1MP3E3 |
|  |  |  |  | Evidence | 4 | 4 | 4 | 4 | 4 |
|  |  |  |  | Focus | 2 | 2 | 2 | 2 | 2 |
|  |  |  |  | Polarity | 7 | 7 | 7 | 7 | 7 |
| 526 | 5004 | 11076964\_267 | Contribution of Myo1p Domains to Function ll known defects of deltamyo1 cells are corrected by a construct with just the head and TH1 domains, |A5:\*\*1SP3E1| a protein similar to a short-tailed myosin-I - like brush border myosin-I. |A5:\*\*2SP3E0| |A1:\*\*1SP3E0| |A2:\*\*1SP3E0| |A3:\*\*1SP3E0| |A4:\*\*1SP3E0| |  |  |  |  |  |  |
|  |  |  |  | Annotation | 1SP3E0 1SP3E0 | 1SP3E0 1SP3E0 | 1SP3E0 1SP3E0 | 1SP3E0 1SP3E0 | 1SP3E1 2SP3E0 |
|  |  |  |  | Evidence | 1 1 | 1 1 | 1 1 | 1 1 | 2 1 |
|  |  |  |  | Focus | 4 4 | 4 4 | 4 4 | 4 4 | 4 4 |
|  |  |  |  | Polarity | 7 7 | 7 7 | 7 7 | 7 7 | 7 7 |
| 527 | 7459 | 11113198\_146 | No significant differences were found between the wild type and any of the mutant forms of CDC42, |A5:\*\*1SN3E1| |A3:\*\*1SN2E3| |A4:\*\*1SN2E3| demonstrating that developmental phenotypes are caused by altered function |A5:\*\*2SP3E1| and not by intracellular amounts of the different Cdc42p mutant proteins. |A5:\*\*3SN3E0| |A1:\*\*1SP3E3| |A2:\*\*1SP3E3| |A3:\*\*2SN3E3| |A4:\*\*2SN3E3| |  |  |  |  |  |  |
|  |  |  |  | Annotation | 1SP3E3 1SP3E3 1SP3E3 | 1SP3E3 1SP3E3 1SP3E3 | 1SN2E3 2SN3E3 2SN3E3 | 1SN2E3 2SN3E3 2SN3E3 | 1SN3E1 2SP3E1 3SN3E0 |
|  |  |  |  | Evidence | 4 4 4 | 4 4 4 | 4 4 4 | 4 4 4 | 2 2 1 |
|  |  |  |  | Focus | 4 4 4 | 4 4 4 | 4 4 4 | 4 4 4 | 4 4 4 |
|  |  |  |  | Polarity | 7 7 7 | 7 7 7 | 2 1 1 | 2 1 1 | 1 7 1 |
| 528 | 463 | 10197532\_65 | At this stage in development, when axons are contacting most of the dendrite surface, we found that the dendrite marker MAP2 was most useful for identifying axonal versus dendritic domains; labeled axons were identified as myc-positive, MAP2-negative, long, fine caliber processes that typically extended far beyond the somatodendritic domain. |A1:\*\*1SP3E3| |A2:\*\*1SP3E3| |A3:\*\*1SP3E3| |A4:\*\*1SP3E3| |A5:\*\*1SP3E3| |  |  |  |  |  |  |
|  |  |  |  | Annotation | 1SP3E3 | 1SP3E3 | 1SP3E3 | 1SP3E3 | 1SP3E3 |
|  |  |  |  | Evidence | 4 | 4 | 4 | 4 | 4 |
|  |  |  |  | Focus | 4 | 4 | 4 | 4 | 4 |
|  |  |  |  | Polarity | 7 | 7 | 7 | 7 | 7 |
| 529 | 8627 | 12194857\_194 | However, the molecular machinery required for the formation of these late endosomal compartments has only recently begun to be characterized. |A1:\*\*1GP2E0| |A2:\*\*1SP2E0| |A3:\*\*1SP3E1| |A4:\*\*1SP3E1| |A5:\*\*1SP3E1| |  |  |  |  |  |  |
|  |  |  |  | Annotation | 1GP2E0 | 1SP2E0 | 1SP3E1 | 1SP3E1 | 1SP3E1 |
|  |  |  |  | Evidence | 1 | 1 | 2 | 2 | 2 |
|  |  |  |  | Focus | 1 | 4 | 4 | 4 | 4 |
|  |  |  |  | Polarity | 6 | 6 | 7 | 7 | 7 |
| 530 | 8468 | 12033758\_5 | A hypothesis about the noise that frequently accompanies this release is offered |A5:\*\*1SP1E1| and includes anatomic, physiologic, and functional models of articular release. |A5:\*\*2SP3E0| |A1:\*\*1GP3E1| |A2:\*\*1GP3E1| |A3:\*\*1SP2E1| |A4:\*\*1SP2E1| |  |  |  |  |  |  |
|  |  |  |  | Annotation | 1GP3E1 1GP3E1 | 1GP3E1 1GP3E1 | 1SP2E1 1SP2E1 | 1SP2E1 1SP2E1 | 1SP1E1 2SP3E0 |
|  |  |  |  | Evidence | 2 2 | 2 2 | 2 2 | 2 2 | 2 1 |
|  |  |  |  | Focus | 1 1 | 1 1 | 4 4 | 4 4 | 4 4 |
|  |  |  |  | Polarity | 7 7 | 7 7 | 6 6 | 6 6 | 5 7 |
| 531 | 8917 | 10688668\_126 | Protein concentrations in cytoplasmic and nuclear extracts were measured with a Bio-Rad protein assay kit, and samples containing 20 mug were prepared and used for SDS-PAGE and Western blotting (see "Immunoprecipitation, immunoblotting, and immunocomplex kinase assays"` above). |A1:\*\*1MP3E3| |A2:\*\*1MSP3E3| |A3:\*\*1MP3E3| |A4:\*\*1MP3E3| |A5:\*\*1MP3E3| |  |  |  |  |  |  |
|  |  |  |  | Annotation | 1MP3E3 | 1MSP3E3 | 1MP3E3 | 1MP3E3 | 1MP3E3 |
|  |  |  |  | Evidence | 4 | 4 | 4 | 4 | 4 |
|  |  |  |  | Focus | 2 | 6 | 2 | 2 | 2 |
|  |  |  |  | Polarity | 7 | 7 | 7 | 7 | 7 |
| 532 | 7566 | 10523671\_113 | Plasmid LexAG4-225-797 (pAKS44), which encodes a LexA (aa 1 to 202)-Gal4 (aa 225 to 797) fusion protein, was constructed by fusing GAL4 codons 225 to 797 to LEXA codons 1 to 202 in pEG202 (from R. Brent). |A1:\*\*1SP3E3| |A2:\*\*1MP3E23| |A3:\*\*1MP3E1| |A4:\*\*1SP3E0| |A5:\*\*1MP3E3| |  |  |  |  |  |  |
|  |  |  |  | Annotation | 1SP3E3 | 1MP3E23 | 1MP3E1 | 1SP3E0 | 1MP3E3 |
|  |  |  |  | Evidence | 4 | 3 | 2 | 1 | 4 |
|  |  |  |  | Focus | 4 | 2 | 2 | 4 | 2 |
|  |  |  |  | Polarity | 7 | 7 | 7 | 7 | 7 |
| 533 | 2001 | 10523632\_9 | This suggests a distinct nonredundant role for Fps/Fes in signaling from the GM-CSF receptor |A3:\*\*1SP2E0| |A4:\*\*1SP2E0| that does not extend to the closely related IL-3 receptor. |A3:\*\*2SN3E0| |A1:\*\*1SP3E0| |A2:\*\*1SP3E1| |A4:\*\*2SN3E0| |A5:\*\*1SN3E1| |  |  |  |  |  |  |
|  |  |  |  | Annotation | 1SP3E0 1SP3E0 | 1SP3E1 1SP3E1 | 1SP2E0 2SN3E0 | 1SP2E0 2SN3E0 | 1SN3E1 1SN3E1 |
|  |  |  |  | Evidence | 1 1 | 2 2 | 1 1 | 1 1 | 2 2 |
|  |  |  |  | Focus | 4 4 | 4 4 | 4 4 | 4 4 | 4 4 |
|  |  |  |  | Polarity | 7 7 | 7 7 | 6 1 | 6 1 | 1 1 |
| 534 | 2361 | 9809063\_90 | After 2 days, the cells were radiolabeled with 35S-methionine for 2 hr, then extracts were prepared and protein complexes were immunoprecipitated using antibodies specific for Pim-1 (lanes 1, 4, and 7) or p100 (anti-FLAG; lanes 2, 5, and 8), or with control, nonimmune antibodies (lanes 3, 6, and 9). |A1:\*\*1MP3E3| |A2:\*\*1MP3E3| |A3:\*\*1MP3E3| |A4:\*\*1MP3E3| |A5:\*\*1MP3E3| |  |  |  |  |  |  |
|  |  |  |  | Annotation | 1MP3E3 | 1MP3E3 | 1MP3E3 | 1MP3E3 | 1MP3E3 |
|  |  |  |  | Evidence | 4 | 4 | 4 | 4 | 4 |
|  |  |  |  | Focus | 2 | 2 | 2 | 2 | 2 |
|  |  |  |  | Polarity | 7 | 7 | 7 | 7 | 7 |
| 535 | 7904 | 9813013\_155 | Sedimentation velocity was measured at 50,000 rpm at 20 degrees C with scans shown at 10-min intervals. |A1:\*\*1MP3E3| |A2:\*\*1MP3E3| |A3:\*\*1SP3E3| |A4:\*\*1SP3E3| |A5:\*\*1MP3E3| |  |  |  |  |  |  |
|  |  |  |  | Annotation | 1MP3E3 | 1MP3E3 | 1SP3E3 | 1SP3E3 | 1MP3E3 |
|  |  |  |  | Evidence | 4 | 4 | 4 | 4 | 4 |
|  |  |  |  | Focus | 2 | 2 | 4 | 4 | 2 |
|  |  |  |  | Polarity | 7 | 7 | 7 | 7 | 7 |
| 536 | 7336 | 10799454\_50 | Briefly, MT-2 cells (AIDS Research and Reference Reagent Program, NIH) and PHA-stimulated PBMCs from donors were infected with primary HIV-1 isolates in parallel. |A1:\*\*1SP3E3| |A2:\*\*1SP3E3| |A3:\*\*1SP3E1| |A4:\*\*1SP3E0| |A5:\*\*1MP3E3| |  |  |  |  |  |  |
|  |  |  |  | Annotation | 1SP3E3 | 1SP3E3 | 1SP3E1 | 1SP3E0 | 1MP3E3 |
|  |  |  |  | Evidence | 4 | 4 | 2 | 1 | 4 |
|  |  |  |  | Focus | 4 | 4 | 4 | 4 | 2 |
|  |  |  |  | Polarity | 7 | 7 | 7 | 7 | 7 |
| 537 | 5323 | 9891076\_293 | It has been suggested that PKC mediates inhibition of GSK-3 by Wnt signaling (8). |A1:\*\*1SN3E2| |A2:\*\*1SP2E2-| |A3:\*\*1SP2E2-| |A4:\*\*1SP2E2-| |A5:\*\*1SP2E2| |  |  |  |  |  |  |
|  |  |  |  | Annotation | 1SN3E2 | 1SP2E2- | 1SP2E2- | 1SP2E2- | 1SP2E2 |
|  |  |  |  | Evidence | 3 | 3 | 3 | 3 | 3 |
|  |  |  |  | Focus | 4 | 4 | 4 | 4 | 4 |
|  |  |  |  | Polarity | 1 | 6 | 6 | 6 | 6 |
| 538 | 4307 | 10611252\_35 | Comparison of the amino acid sequence of the full-length 1.8-kb ORF with that of human XRCC1 by using Multiple Alignment Construction and Analysis Workbench (MACAW) |A5:\*\*1MP3E3| revealed significant homology to human XRCC1 (37.7% identity; 55.2% similarity). |A5:\*\*2SP3E3| |A1:\*\*1SP3E0| |A2:\*\*1SP3E3| |A3:\*\*1SP3E3| |A4:\*\*1SP3E3| |  |  |  |  |  |  |
|  |  |  |  | Annotation | 1SP3E0 1SP3E0 | 1SP3E3 1SP3E3 | 1SP3E3 1SP3E3 | 1SP3E3 1SP3E3 | 1MP3E3 2SP3E3 |
|  |  |  |  | Evidence | 1 1 | 4 4 | 4 4 | 4 4 | 4 4 |
|  |  |  |  | Focus | 4 4 | 4 4 | 4 4 | 4 4 | 2 4 |
|  |  |  |  | Polarity | 7 7 | 7 7 | 7 7 | 7 7 | 7 7 |
| 539 | 4434 | 10585437\_3 | In contrast to the C-domain, the size of the CP1 insertion is conserved across bacteria, archaebacteria, and eukaryotes. |A1:\*\*1SP3E0| |A2:\*\*1SP3E0| |A3:\*\*1SP3E0| |A4:\*\*1SP3E0| |A5:\*\*1SP3E0| |  |  |  |  |  |  |
|  |  |  |  | Annotation | 1SP3E0 | 1SP3E0 | 1SP3E0 | 1SP3E0 | 1SP3E0 |
|  |  |  |  | Evidence | 1 | 1 | 1 | 1 | 1 |
|  |  |  |  | Focus | 4 | 4 | 4 | 4 | 4 |
|  |  |  |  | Polarity | 7 | 7 | 7 | 7 | 7 |
| 540 | 2320 | 9844016\_72 | These show the pattern of distribution of gamma2 mRNA transcripts across all six cortical layers. |A1:\*\*1SP3E0| |A2:\*\*1SP3E1| |A3:\*\*1SP3E3| |A4:\*\*1SP3E3| |A5:\*\*1SP3E3| |  |  |  |  |  |  |
|  |  |  |  | Annotation | 1SP3E0 | 1SP3E1 | 1SP3E3 | 1SP3E3 | 1SP3E3 |
|  |  |  |  | Evidence | 1 | 2 | 4 | 4 | 4 |
|  |  |  |  | Focus | 4 | 4 | 4 | 4 | 4 |
|  |  |  |  | Polarity | 7 | 7 | 7 | 7 | 7 |
| 541 | 7911 | 9784521\_183 | These localizations are consistent with the sequential development of the apoptotic process, |A5:\*\*1SP3E3| |A3:\*\*1SP3E0| i.e., |A4:\*\*1SP3E0| the initial expression of the p53 apoptosis-inducing protein in the parabasal layers, |A5:\*\*2SP3E3+| and consequent detection of DNA damage in the more superficial layers (Fig. 1B and C). |A5:\*\*3SP3E3| |A1:\*\*1SP3E3| |A2:\*\*1SP3E3| |A3:\*\*2SP3E3| |A4:\*\*2SP3E3| |  |  |  |  |  |  |
|  |  |  |  | Annotation | 1SP3E3 1SP3E3 1SP3E3 1SP3E3 | 1SP3E3 1SP3E3 1SP3E3 1SP3E3 | 1SP3E0 2SP3E3 2SP3E3 2SP3E3 | 1SP3E0 1SP3E0 2SP3E3 2SP3E3 | 1SP3E3 2SP3E3+ 2SP3E3+ 3SP3E3 |
|  |  |  |  | Evidence | 4 4 4 4 | 4 4 4 4 | 1 4 4 4 | 1 1 4 4 | 4 4 4 4 |
|  |  |  |  | Focus | 4 4 4 4 | 4 4 4 4 | 4 4 4 4 | 4 4 4 4 | 4 4 4 4 |
|  |  |  |  | Polarity | 7 7 7 7 | 7 7 7 7 | 7 7 7 7 | 7 7 7 7 | 7 7 7 7 |
| 542 | 774 | 10090727\_119 | Although the area of expression is expanded in the compound mutant embryos as compared with wild-type embryos, |A3:\*\*1SP3E0| |A4:\*\*1SP3E0| we favor the view that the expanded expression is due to the generation of excess neural ectoderm at the expense of mesoderm, |A3:\*\*2SP3E3| |A4:\*\*2SP3E3| |A5:\*\*1SP3E2+| rather than a loss of negative regulation, which has been shown to operate in the absence of Wnt signals (Cavallo et al. 1998; Waltzer and Bienz 1998 ). |A3:\*\*3SP3E2-| |A1:\*\*1SP3E23| |A2:\*\*1SP3E23| |A4:\*\*3SP3E2-| |A5:\*\*2SP3E2-| |  |  |  |  |  |  |
|  |  |  |  | Annotation | 1SP3E23 1SP3E23 1SP3E23 | 1SP3E23 1SP3E23 1SP3E23 | 1SP3E0 2SP3E3 3SP3E2- | 1SP3E0 2SP3E3 3SP3E2- | 1SP3E2+ 1SP3E2+ 2SP3E2- |
|  |  |  |  | Evidence | 3 3 3 | 3 3 3 | 1 4 3 | 1 4 3 | 3 3 3 |
|  |  |  |  | Focus | 4 4 4 | 4 4 4 | 4 4 4 | 4 4 4 | 4 4 4 |
|  |  |  |  | Polarity | 7 7 7 | 7 7 7 | 7 7 7 | 7 7 7 | 7 7 7 |
| 543 | 8137 | 12005883\_6 | These results suggest the possibility that the relative change in correlation dimension can be a useful global measure of brain dynamics, |A5:\*\*1SP2E1| e.g., in determining the levels of mental activity, even if little is known about the underlying neurological processes. |A5:\*\*2SP3E1| |A1:\*\*1GP3E3| |A2:\*\*1SP2E3| |A3:\*\*1SP2E3| |A4:\*\*1SP2E3| |  |  |  |  |  |  |
|  |  |  |  | Annotation | 1GP3E3 1GP3E3 | 1SP2E3 1SP2E3 | 1SP2E3 1SP2E3 | 1SP2E3 1SP2E3 | 1SP2E1 2SP3E1 |
|  |  |  |  | Evidence | 4 4 | 4 4 | 4 4 | 4 4 | 2 2 |
|  |  |  |  | Focus | 1 1 | 4 4 | 4 4 | 4 4 | 4 4 |
|  |  |  |  | Polarity | 7 7 | 6 6 | 6 6 | 6 6 | 6 7 |
| 544 | 3409 | 10985388\_101 | The secondary antibodies were either Cy3- or Cy2-conjugated anti-guinea pig, Cy3-conjugated anti-rabbit and Cy3-conjugated anti-mouse (Jackson ImmunoResearch Laboratories). |A1:\*\*1SP2E3| |A2:\*\*1SP2E3| |A3:\*\*1SP2E1| |A4:\*\*1SP3E0| |A5:\*\*1MP3E3| |  |  |  |  |  |  |
|  |  |  |  | Annotation | 1SP2E3 | 1SP2E3 | 1SP2E1 | 1SP3E0 | 1MP3E3 |
|  |  |  |  | Evidence | 4 | 4 | 2 | 1 | 4 |
|  |  |  |  | Focus | 4 | 4 | 4 | 4 | 2 |
|  |  |  |  | Polarity | 6 | 6 | 6 | 7 | 7 |
| 545 | 843 | 9390512\_196 | Despite the fact that both D3 and 5-HT2AR genes seem to have at best a weak effect on schizophrenia susceptibility, |A5:\*\*1SP3E1| these could in principle be important findings, as they could offer an anchor for further biological studies. |A5:\*\*2SP3E0| |A1:\*\*1SP2E0| |A2:\*\*1SP2E0| |A3:\*\*1SP2E0| |A4:\*\*1SP2E0| |  |  |  |  |  |  |
|  |  |  |  | Annotation | 1SP2E0 1SP2E0 | 1SP2E0 1SP2E0 | 1SP2E0 1SP2E0 | 1SP2E0 1SP2E0 | 1SP3E1 2SP3E0 |
|  |  |  |  | Evidence | 1 1 | 1 1 | 1 1 | 1 1 | 2 1 |
|  |  |  |  | Focus | 4 4 | 4 4 | 4 4 | 4 4 | 4 4 |
|  |  |  |  | Polarity | 6 6 | 6 6 | 6 6 | 6 6 | 7 7 |
| 546 | 2906 | 9331371\_185 | One of the two residues mutated in the g region (T1080) is conserved in all RNA polymerases, and like the sua8 suppressors in the c and d regions (see above), the position of the sua8 suppressor in the h region is conserved in all RNA polymerases aligned aligned. |A1:\*\*1SP3E0| |A2:\*\*1SP3E1| |A3:\*\*1SP3E3| |A4:\*\*1SP3E3| |A5:\*\*1SP3E3| |  |  |  |  |  |  |
|  |  |  |  | Annotation | 1SP3E0 | 1SP3E1 | 1SP3E3 | 1SP3E3 | 1SP3E3 |
|  |  |  |  | Evidence | 1 | 2 | 4 | 4 | 4 |
|  |  |  |  | Focus | 4 | 4 | 4 | 4 | 4 |
|  |  |  |  | Polarity | 7 | 7 | 7 | 7 | 7 |
| 547 | 5618 | 8986719\_222 | Therefore, this JAK3-independent IL-4 signaling pathway may be important in leukocytes when levels of JAK3 are minimal. |A1:\*\*1SP1E1| |A2:\*\*1SP2E1| |A3:\*\*1SP1E0| |A4:\*\*1SP2E0| |A5:\*\*1SP2E1| |  |  |  |  |  |  |
|  |  |  |  | Annotation | 1SP1E1 | 1SP2E1 | 1SP1E0 | 1SP2E0 | 1SP2E1 |
|  |  |  |  | Evidence | 2 | 2 | 1 | 1 | 2 |
|  |  |  |  | Focus | 4 | 4 | 4 | 4 | 4 |
|  |  |  |  | Polarity | 5 | 6 | 5 | 6 | 6 |
| 548 | 9819 | 9555896\_80 | With glucose and succinate, growth of P. putida commenced at very low pO2 values and showed a saturation curve with increasing pO2. |A1:\*\*1SP3E3| |A2:\*\*1SP3E3| |A3:\*\*1SP3E3| |A4:\*\*1SP3E3+| |A5:\*\*1SP3E3| |  |  |  |  |  |  |
|  |  |  |  | Annotation | 1SP3E3 | 1SP3E3 | 1SP3E3 | 1SP3E3+ | 1SP3E3 |
|  |  |  |  | Evidence | 4 | 4 | 4 | 4 | 4 |
|  |  |  |  | Focus | 4 | 4 | 4 | 4 | 4 |
|  |  |  |  | Polarity | 7 | 7 | 7 | 7 | 7 |
| 549 | 667 | 8996174\_85 | In the rabbit aorta and the prostate phenylephrine elicited contractions with potencies (EC50) of approximately 0.3 and 4 muM, respectively. |A1:\*\*1SP3E0| |A2:\*\*1SP3E0| |A3:\*\*1SP3E0| |A4:\*\*1SP3E0| |A5:\*\*1SP3E3| |  |  |  |  |  |  |
|  |  |  |  | Annotation | 1SP3E0 | 1SP3E0 | 1SP3E0 | 1SP3E0 | 1SP3E3 |
|  |  |  |  | Evidence | 1 | 1 | 1 | 1 | 4 |
|  |  |  |  | Focus | 4 | 4 | 4 | 4 | 4 |
|  |  |  |  | Polarity | 7 | 7 | 7 | 7 | 7 |
| 550 | 4878 | 11090626\_321 | In this case, another Flp monomer, instead of a special enzyme, may be sufficient to resolve the covalent linkage between the tyrosine and DNA, |A5:\*\*1SP2E3| even if the original attacking Flp monomer has become denatured and/or degraded. |A5:\*\*2SP3E3| |A1:\*\*1SP1E1| |A2:\*\*1SP1E1| |A3:\*\*1SP2E0| |A4:\*\*1SP2E0| |  |  |  |  |  |  |
|  |  |  |  | Annotation | 1SP1E1 1SP1E1 | 1SP1E1 1SP1E1 | 1SP2E0 1SP2E0 | 1SP2E0 1SP2E0 | 1SP2E3 2SP3E3 |
|  |  |  |  | Evidence | 2 2 | 2 2 | 1 1 | 1 1 | 4 4 |
|  |  |  |  | Focus | 4 4 | 4 4 | 4 4 | 4 4 | 4 4 |
|  |  |  |  | Polarity | 5 5 | 5 5 | 6 6 | 6 6 | 6 7 |
| 551 | 9432 | 11319104\_56 | Forty-one cycles of amplification were done under the following conditions: denaturation at 95 degrees C for 1 min, annealing at 66 degrees C for 1 min, and extension at 72 degrees C for 2 min. |A1:\*\*1MP3E3| |A2:\*\*1MP3E3| |A3:\*\*1MP3E3| |A4:\*\*1MP3E3| |A5:\*\*1MP3E3| |  |  |  |  |  |  |
|  |  |  |  | Annotation | 1MP3E3 | 1MP3E3 | 1MP3E3 | 1MP3E3 | 1MP3E3 |
|  |  |  |  | Evidence | 4 | 4 | 4 | 4 | 4 |
|  |  |  |  | Focus | 2 | 2 | 2 | 2 | 2 |
|  |  |  |  | Polarity | 7 | 7 | 7 | 7 | 7 |
| 552 | 2769 | 11913030\_3 | Changes in the usual way of speaking are frequently observed after behavioural treatments for adults who stutter, |A3:\*\*1SP3E1| |A4:\*\*1SP3E1| |A5:\*\*1SP3E1| and it is possible that operant treatments for children also invoke such changes. |A3:\*\*2SP2E0| |A1:\*\*1GP2E0| |A2:\*\*1GP2E0| |A4:\*\*2SP2E0| |A5:\*\*2SP2E1| |  |  |  |  |  |  |
|  |  |  |  | Annotation | 1GP2E0 1GP2E0 | 1GP2E0 1GP2E0 | 1SP3E1 2SP2E0 | 1SP3E1 2SP2E0 | 1SP3E1 2SP2E1 |
|  |  |  |  | Evidence | 1 1 | 1 1 | 2 1 | 2 1 | 2 2 |
|  |  |  |  | Focus | 1 1 | 1 1 | 4 4 | 4 4 | 4 4 |
|  |  |  |  | Polarity | 6 6 | 6 6 | 7 6 | 7 6 | 7 6 |
| 553 | 2267 | 12810698\_239 | These findings differ from those obtained with embryos and cancer cells that have a strongly activated Wnt pathway |A5:\*\*1SP3E12| in which expression of E-cadherin antagonizes beta-catenin/TCF signaling ( Heasman et al., 1994; Funayama et al., 1995; Fagotto et al., 1996; Sanson et al., 1996; Gottardi et al., 2001). |A5:\*\*2SP3E2-| |A1:\*\*1SP3E2| |A2:\*\*1SP3E12| |A3:\*\*1SP3E2+| |A4:\*\*1SP3E2| |  |  |  |  |  |  |
|  |  |  |  | Annotation | 1SP3E2 1SP3E2 | 1SP3E12 1SP3E12 | 1SP3E2+ 1SP3E2+ | 1SP3E2 1SP3E2 | 1SP3E12 2SP3E2- |
|  |  |  |  | Evidence | 3 3 | 2 2 | 3 3 | 3 3 | 2 3 |
|  |  |  |  | Focus | 4 4 | 4 4 | 4 4 | 4 4 | 4 4 |
|  |  |  |  | Polarity | 7 7 | 7 7 | 7 7 | 7 7 | 7 7 |
| 554 | 6009 | 11294857\_2 | Several cell types, including neuroendocrine chromaffin cells, have evolved to sense oxygen levels and initiate specific adaptive responses to hypoxia. |A1:\*\*1SP3E0| |A2:\*\*1SP3E0| |A3:\*\*1SP3E0| |A4:\*\*1SP3E0| |A5:\*\*1SP3E1+| |  |  |  |  |  |  |
|  |  |  |  | Annotation | 1SP3E0 | 1SP3E0 | 1SP3E0 | 1SP3E0 | 1SP3E1+ |
|  |  |  |  | Evidence | 1 | 1 | 1 | 1 | 2 |
|  |  |  |  | Focus | 4 | 4 | 4 | 4 | 4 |
|  |  |  |  | Polarity | 7 | 7 | 7 | 7 | 7 |
| 555 | 81 | 9153218\_14 | We found that transcription of the rrn operon in intact spinach plants is regulated by usage of two promoters. |A1:\*\*1SP3E3| |A2:\*\*1SP3E3| |A3:\*\*1SP3E3| |A4:\*\*1SP3E3| |A5:\*\*1SP3E3| |  |  |  |  |  |  |
|  |  |  |  | Annotation | 1SP3E3 | 1SP3E3 | 1SP3E3 | 1SP3E3 | 1SP3E3 |
|  |  |  |  | Evidence | 4 | 4 | 4 | 4 | 4 |
|  |  |  |  | Focus | 4 | 4 | 4 | 4 | 4 |
|  |  |  |  | Polarity | 7 | 7 | 7 | 7 | 7 |
| 556 | 9151 | 12477929\_29 | However, comparison of the increase in the morbid risk of schizophrenia associated with the identified PRODH2 variation |A3:\*\*1SP3E0+| |A4:\*\*1SP3E0+| |A5:\*\*1GP3E0+| to the risk associated with the 22q11 microdeletion could not exclude contribution from other genes in the region. |A3:\*\*2SN3E0| |A1:\*\*1SP2E0| |A2:\*\*1SP3E0| |A4:\*\*2SN3E0| |A5:\*\*2SN3E0| |  |  |  |  |  |  |
|  |  |  |  | Annotation | 1SP2E0 1SP2E0 | 1SP3E0 1SP3E0 | 1SP3E0+ 2SN3E0 | 1SP3E0+ 2SN3E0 | 1GP3E0+ 2SN3E0 |
|  |  |  |  | Evidence | 1 1 | 1 1 | 1 1 | 1 1 | 1 1 |
|  |  |  |  | Focus | 4 4 | 4 4 | 4 4 | 4 4 | 1 4 |
|  |  |  |  | Polarity | 6 6 | 7 7 | 7 1 | 7 1 | 7 1 |
| 557 | 7067 | 10358016\_35 | Ca2+-induced beta-hexosaminidase release was reduced by approximately 35% in the mastoparan-treated cells (Fig. 4 b), consistent with the similarly reduced (40%) levels of cytosolic cAMP detected in response to forskolin after mastoparan treatment (Fig. 4 d). |A1:\*\*1SP3E3-| |A2:\*\*1SP3E3-| |A3:\*\*1SP3E3-| |A4:\*\*1SP3E3-| |A5:\*\*1SP3E3-| |  |  |  |  |  |  |
|  |  |  |  | Annotation | 1SP3E3- | 1SP3E3- | 1SP3E3- | 1SP3E3- | 1SP3E3- |
|  |  |  |  | Evidence | 4 | 4 | 4 | 4 | 4 |
|  |  |  |  | Focus | 4 | 4 | 4 | 4 | 4 |
|  |  |  |  | Polarity | 7 | 7 | 7 | 7 | 7 |
| 558 | 5250 | 11333245\_158 | These clustered breaksites are indicated by brackets, and the breaksite sequences are aligned. |A1:\*\*1SP3E1| |A2:\*\*1GP3E1| |A3:\*\*1SP3E0| |A4:\*\*1SP3E0| |A5:\*\*1GP3E3| |  |  |  |  |  |  |
|  |  |  |  | Annotation | 1SP3E1 | 1GP3E1 | 1SP3E0 | 1SP3E0 | 1GP3E3 |
|  |  |  |  | Evidence | 2 | 2 | 1 | 1 | 4 |
|  |  |  |  | Focus | 4 | 1 | 4 | 4 | 1 |
|  |  |  |  | Polarity | 7 | 7 | 7 | 7 | 7 |
| 559 | 7314 | 11792731\_168 | The accretion of DHA during fetal brain development has been widely studied. |A1:\*\*1SP3E1| |A2:\*\*1SP3E0| |A3:\*\*1SP3E0| |A4:\*\*1SP3E0| |A5:\*\*1GP3E1| |  |  |  |  |  |  |
|  |  |  |  | Annotation | 1SP3E1 | 1SP3E0 | 1SP3E0 | 1SP3E0 | 1GP3E1 |
|  |  |  |  | Evidence | 2 | 1 | 1 | 1 | 2 |
|  |  |  |  | Focus | 4 | 4 | 4 | 4 | 1 |
|  |  |  |  | Polarity | 7 | 7 | 7 | 7 | 7 |
| 560 | 6510 | 9643351\_181 | While the initial lipid compositions of the incubation media resulted in the formation of uniform, mixed lipid mixed-lipid micelles, |A3:\*\*1SP3E0| |A4:\*\*1SP3E0| the aggregation state of medium lipids could be altered during cell incubations by the disproportional depletion of medium lipids stemming from the unequal rates of lipid uptake. |A3:\*\*2SP2E0| |A1:\*\*1SP2E0| |A2:\*\*1SP2E0| |A4:\*\*2SP2E0| |A5:\*\*1SP3E1| |  |  |  |  |  |  |
|  |  |  |  | Annotation | 1SP2E0 1SP2E0 | 1SP2E0 1SP2E0 | 1SP3E0 2SP2E0 | 1SP3E0 2SP2E0 | 1SP3E1 1SP3E1 |
|  |  |  |  | Evidence | 1 1 | 1 1 | 1 1 | 1 1 | 2 2 |
|  |  |  |  | Focus | 4 4 | 4 4 | 4 4 | 4 4 | 4 4 |
|  |  |  |  | Polarity | 6 6 | 6 6 | 7 6 | 7 6 | 7 7 |
| 561 | 4837 | 10417164\_42 | Mice were infected intravenously via the tail vein with 2 x 10^5^ live bacilli in 100 mul, as determined by viable counts on 7H10 agar plates (Difco Laboratories, Detroit, Mich.). |A1:\*\*1MP3E3| |A2:\*\*1MP3E3| |A3:\*\*1SP3E3| |A4:\*\*1MP3E2| |A5:\*\*1MP3E3| |  |  |  |  |  |  |
|  |  |  |  | Annotation | 1MP3E3 | 1MP3E3 | 1SP3E3 | 1MP3E2 | 1MP3E3 |
|  |  |  |  | Evidence | 4 | 4 | 4 | 3 | 4 |
|  |  |  |  | Focus | 2 | 2 | 4 | 2 | 2 |
|  |  |  |  | Polarity | 7 | 7 | 7 | 7 | 7 |
| 562 | 7907 | 12019609\_3 | Production of 14CO2 was measured by incubating cells in media containing either D-[U-14C] glucose, [1-14C] n-butyrate, L-[U-14C] glutamine, D-[U-14C]glucose, [1-14C]n-butyrate, L-[U-14C]glutamine, or [1-14C]propionate with or without competing substrates. |A1:\*\*1MP3E3| |A2:\*\*1MP3E3| |A3:\*\*1MP3E3| |A4:\*\*1MP3E3| |A5:\*\*1MP3E3| |  |  |  |  |  |  |
|  |  |  |  | Annotation | 1MP3E3 | 1MP3E3 | 1MP3E3 | 1MP3E3 | 1MP3E3 |
|  |  |  |  | Evidence | 4 | 4 | 4 | 4 | 4 |
|  |  |  |  | Focus | 2 | 2 | 2 | 2 | 2 |
|  |  |  |  | Polarity | 7 | 7 | 7 | 7 | 7 |
| 563 | 2084 | 9671478\_48 | In the presence of cellular extracts, a 200-bp fragment can still serve as a substrate ( 6). |A1:\*\*1SP2E2| |A2:\*\*1SP2E2| |A3:\*\*1SP3E2| |A4:\*\*1SP3E2| |A5:\*\*1GP3E2| |  |  |  |  |  |  |
|  |  |  |  | Annotation | 1SP2E2 | 1SP2E2 | 1SP3E2 | 1SP3E2 | 1GP3E2 |
|  |  |  |  | Evidence | 3 | 3 | 3 | 3 | 3 |
|  |  |  |  | Focus | 4 | 4 | 4 | 4 | 1 |
|  |  |  |  | Polarity | 6 | 6 | 7 | 7 | 7 |
| 564 | 508 | 10473520\_100 | Following the identification of BDV RNA in peripheral blood mononuclear cells (PBMC) of experimentally infected rats ( 23), Bode et al. ( 5) |A3:\*\*1SP3E2| |A4:\*\*1SP3E2| reported finding BDV protein in CD14+ PBMC and RNA in PBMC from psychiatric patients. |A1:\*\*1SP3E2| |A2:\*\*1SP3E2| |A3:\*\*2SP3E1| |A4:\*\*2SP3E1| |A5:\*\*1SP3E2| |  |  |  |  |  |  |
|  |  |  |  | Annotation | 1SP3E2 1SP3E2 | 1SP3E2 1SP3E2 | 1SP3E2 2SP3E1 | 1SP3E2 2SP3E1 | 1SP3E2 1SP3E2 |
|  |  |  |  | Evidence | 3 3 | 3 3 | 3 2 | 3 2 | 3 3 |
|  |  |  |  | Focus | 4 4 | 4 4 | 4 4 | 4 4 | 4 4 |
|  |  |  |  | Polarity | 7 7 | 7 7 | 7 7 | 7 7 | 7 7 |
| 565 | 8989 | 11978848\_33 | Specifically, no significant differences in callosal areas were found between monozygotic (MZ) co-twins discordant for schizophrenia (Casanova et al., 1990b), |A2:\*\*1SN3E23| |A5:\*\*1GN3E2| suggesting no disease-specific influences. |A2:\*\*2SN3E3| |A1:\*\*1SN3E23| |A3:\*\*1SN3E2| |A4:\*\*1SN3E2| |A5:\*\*2GN2E2| |  |  |  |  |  |  |
|  |  |  |  | Annotation | 1SN3E23 1SN3E23 | 1SN3E23 2SN3E3 | 1SN3E2 1SN3E2 | 1SN3E2 1SN3E2 | 1GN3E2 2GN2E2 |
|  |  |  |  | Evidence | 3 3 | 3 4 | 3 3 | 3 3 | 3 3 |
|  |  |  |  | Focus | 4 4 | 4 4 | 4 4 | 4 4 | 1 1 |
|  |  |  |  | Polarity | 1 1 | 1 1 | 1 1 | 1 1 | 1 2 |
| 566 | 2471 | 9531542\_53 | Splice variants of CD44 are expressed in putative limb ectoderm and become localized to the AER. |A1:\*\*1SP3E1| |A2:\*\*1SP3E1| |A3:\*\*1SP3E0| |A4:\*\*1SP3E0| |A5:\*\*1SP3E1| |  |  |  |  |  |  |
|  |  |  |  | Annotation | 1SP3E1 | 1SP3E1 | 1SP3E0 | 1SP3E0 | 1SP3E1 |
|  |  |  |  | Evidence | 2 | 2 | 1 | 1 | 2 |
|  |  |  |  | Focus | 4 | 4 | 4 | 4 | 4 |
|  |  |  |  | Polarity | 7 | 7 | 7 | 7 | 7 |
| 567 | 5105 | 11812828\_180 | SFRPs bind to Wnt ligands and modulate their action. |A1:\*\*1SP3E0| |A2:\*\*1SP3E0| |A3:\*\*1SP3E0| |A4:\*\*1SP3E0| |A5:\*\*1GP3E0| |  |  |  |  |  |  |
|  |  |  |  | Annotation | 1SP3E0 | 1SP3E0 | 1SP3E0 | 1SP3E0 | 1GP3E0 |
|  |  |  |  | Evidence | 1 | 1 | 1 | 1 | 1 |
|  |  |  |  | Focus | 4 | 4 | 4 | 4 | 1 |
|  |  |  |  | Polarity | 7 | 7 | 7 | 7 | 7 |
| 568 | 4882 | 12176308\_19 | The cellular zonation and multi-functionality of these digestive filaments suggest another example of a cnidarian structure at the organ level of complexity. |A1:\*\*1GP2E0| |A2:\*\*1SP2E1| |A3:\*\*1SP2E1| |A4:\*\*1SP2E1| |A5:\*\*1SP2E0| |  |  |  |  |  |  |
|  |  |  |  | Annotation | 1GP2E0 | 1SP2E1 | 1SP2E1 | 1SP2E1 | 1SP2E0 |
|  |  |  |  | Evidence | 1 | 2 | 2 | 2 | 1 |
|  |  |  |  | Focus | 1 | 4 | 4 | 4 | 4 |
|  |  |  |  | Polarity | 6 | 6 | 6 | 6 | 6 |
| 569 | 8152 | 9331371\_244 | Forty microliters of whole cell extract (Maroney et al. 1990) |A3:\*\*1SP3E2| |A4:\*\*1SP3E2| |A5:\*\*1MP3E2| was incubated with an equal volume of preimmune or alpha-CSH499 beads. |A3:\*\*2MP3E3| |A1:\*\*1MP3E23| |A2:\*\*1MP3E23| |A4:\*\*2MP3E3| |A5:\*\*1MP3E3| |  |  |  |  |  |  |
|  |  |  |  | Annotation | 1MP3E23 1MP3E23 | 1MP3E23 1MP3E23 | 1SP3E2 2MP3E3 | 1SP3E2 2MP3E3 | 1MP3E2 1MP3E3 |
|  |  |  |  | Evidence | 3 3 | 3 3 | 3 4 | 3 4 | 3 4 |
|  |  |  |  | Focus | 2 2 | 2 2 | 4 2 | 4 2 | 2 2 |
|  |  |  |  | Polarity | 7 7 | 7 7 | 7 7 | 7 7 | 7 7 |
| 570 | 7128 | 12821645\_212 | Fatp4 -/- mice display a significant thickening of the epidermis with a "`too-small-for-size"` volume and a more compact dermis, |A5:\*\*1SP3E1| accompanied by a smaller number of pilo-sebaceous structures and a reduced amount of fatty tissue in the hypodermis. |A5:\*\*2SP3E1-| |A1:\*\*1SP3E0-| |A2:\*\*1SP3E0-| |A3:\*\*1MP3E3| |A4:\*\*1MP3E3| |  |  |  |  |  |  |
|  |  |  |  | Annotation | 1SP3E0- 1SP3E0- | 1SP3E0- 1SP3E0- | 1MP3E3 1MP3E3 | 1MP3E3 1MP3E3 | 1SP3E1 2SP3E1- |
|  |  |  |  | Evidence | 1 1 | 1 1 | 4 4 | 4 4 | 2 2 |
|  |  |  |  | Focus | 4 4 | 4 4 | 2 2 | 2 2 | 4 4 |
|  |  |  |  | Polarity | 7 7 | 7 7 | 7 7 | 7 7 | 7 7 |
| 571 | 4972 | 9573199\_200 | The Ndh enzyme of E. coli catalyzes the first step in the respiratory chain: NADH oxidation |A5:\*\*1SP3E2+| and quinone reduction (54). |A5:\*\*2SP3E2-| |A1:\*\*1SP3E2| |A2:\*\*1SP3E2| |A3:\*\*1SP3E2| |A4:\*\*1SP3E2| |  |  |  |  |  |  |
|  |  |  |  | Annotation | 1SP3E2 1SP3E2 | 1SP3E2 1SP3E2 | 1SP3E2 1SP3E2 | 1SP3E2 1SP3E2 | 1SP3E2+ 2SP3E2- |
|  |  |  |  | Evidence | 3 3 | 3 3 | 3 3 | 3 3 | 3 3 |
|  |  |  |  | Focus | 4 4 | 4 4 | 4 4 | 4 4 | 4 4 |
|  |  |  |  | Polarity | 7 7 | 7 7 | 7 7 | 7 7 | 7 7 |
| 572 | 5764 | 10197540\_144 | The white/gray matter boundary was finally tesselated in a single step using two triangles for each side of a voxel located at the margin of white matter. |A1:\*\*1GP3E3| |A2:\*\*1GP2E3| |A3:\*\*1SP3E3| |A4:\*\*1SP3E3| |A5:\*\*1MP3E3| |  |  |  |  |  |  |
|  |  |  |  | Annotation | 1GP3E3 | 1GP2E3 | 1SP3E3 | 1SP3E3 | 1MP3E3 |
|  |  |  |  | Evidence | 4 | 4 | 4 | 4 | 4 |
|  |  |  |  | Focus | 1 | 1 | 4 | 4 | 2 |
|  |  |  |  | Polarity | 7 | 6 | 7 | 7 | 7 |
| 573 | 5296 | 11086983\_95 | Yellow bars denote distribution of all expressed genes within the same microarray comparison. |A1:\*\*1MP3E3| |A2:\*\*1SP3E0| |A3:\*\*1MP3E1| |A4:\*\*1MP3E3| |A5:\*\*1GP3E0| |  |  |  |  |  |  |
|  |  |  |  | Annotation | 1MP3E3 | 1SP3E0 | 1MP3E1 | 1MP3E3 | 1GP3E0 |
|  |  |  |  | Evidence | 4 | 1 | 2 | 4 | 1 |
|  |  |  |  | Focus | 2 | 4 | 2 | 2 | 1 |
|  |  |  |  | Polarity | 7 | 7 | 7 | 7 | 7 |
| 574 | 3065 | 11861558\_172 | The rectangles indicate the critical regions I, II, and III defined by the deletions deltaB1, deltaDEB-342, and delta4R, respectively. |A1:\*\*1SP3E3| |A2:\*\*1SP2E0| |A3:\*\*1SP3E3| |A4:\*\*1SP3E3| |A5:\*\*1GP3E0| |  |  |  |  |  |  |
|  |  |  |  | Annotation | 1SP3E3 | 1SP2E0 | 1SP3E3 | 1SP3E3 | 1GP3E0 |
|  |  |  |  | Evidence | 4 | 1 | 4 | 4 | 1 |
|  |  |  |  | Focus | 4 | 4 | 4 | 4 | 1 |
|  |  |  |  | Polarity | 7 | 6 | 7 | 7 | 7 |
| 575 | 7405 | 10953060\_17 | There are tight correlations between ligand structure, cytotoxicity, and DNA binding kinetics for the 9-aminoacridine-4-carboxamide class of compounds (Atwell et al., 1984; Denny et al., 1986, 1987; Rewcastle et al., 1986; Wakelin et al., 1987). |A1:\*\*1SP3E2| |A2:\*\*1SP3E2| |A3:\*\*1SP3E2| |A4:\*\*1SP3E2| |A5:\*\*1SP3E2| |  |  |  |  |  |  |
|  |  |  |  | Annotation | 1SP3E2 | 1SP3E2 | 1SP3E2 | 1SP3E2 | 1SP3E2 |
|  |  |  |  | Evidence | 3 | 3 | 3 | 3 | 3 |
|  |  |  |  | Focus | 4 | 4 | 4 | 4 | 4 |
|  |  |  |  | Polarity | 7 | 7 | 7 | 7 | 7 |
| 576 | 4462 | 10555149\_176 | PPARdelta and PPAR activity was assessed as transcriptional activity of the DRE and ACO luciferase reporters, respectively. |A1:\*\*1MP3E3| |A2:\*\*1SP2E3| |A3:\*\*1SP3E3| |A4:\*\*1SP3E3| |A5:\*\*1SP3E3| |  |  |  |  |  |  |
|  |  |  |  | Annotation | 1MP3E3 | 1SP2E3 | 1SP3E3 | 1SP3E3 | 1SP3E3 |
|  |  |  |  | Evidence | 4 | 4 | 4 | 4 | 4 |
|  |  |  |  | Focus | 2 | 4 | 4 | 4 | 4 |
|  |  |  |  | Polarity | 7 | 6 | 7 | 7 | 7 |
| 577 | 3815 | 9430227\_165 | Previous studies have shown that the death domains of TNFR1 and DR3 are similarly responsible for both these activities, |A1:\*\*1SP3E1| |A2:\*\*1SP3E1| and these results can be explained mechanistically by the ability of their death domains to recruit TRADD ( Tartaglia et al. 1993 ; Kitson et al. 1996 ). |A1:\*\*2SP2E2| |A2:\*\*1SP2E2| |A3:\*\*1SP3E2| |A4:\*\*1SP3E2| |A5:\*\*1SP3E2| |  |  |  |  |  |  |
|  |  |  |  | Annotation | 1SP3E1 2SP2E2 | 1SP3E1 1SP2E2 | 1SP3E2 1SP3E2 | 1SP3E2 1SP3E2 | 1SP3E2 1SP3E2 |
|  |  |  |  | Evidence | 2 3 | 2 3 | 3 3 | 3 3 | 3 3 |
|  |  |  |  | Focus | 4 4 | 4 4 | 4 4 | 4 4 | 4 4 |
|  |  |  |  | Polarity | 7 6 | 7 6 | 7 7 | 7 7 | 7 7 |
| 578 | 7582 | 9592097\_319 | LTP can be induced as early as postnatal day 2 in the rat hippocampus (Durand et al., 1996). |A1:\*\*1SP2E2| |A2:\*\*1SP2E2| |A3:\*\*1SP3E2+| |A4:\*\*1SP3E2+| |A5:\*\*1SP3E2| |  |  |  |  |  |  |
|  |  |  |  | Annotation | 1SP2E2 | 1SP2E2 | 1SP3E2+ | 1SP3E2+ | 1SP3E2 |
|  |  |  |  | Evidence | 3 | 3 | 3 | 3 | 3 |
|  |  |  |  | Focus | 4 | 4 | 4 | 4 | 4 |
|  |  |  |  | Polarity | 6 | 6 | 7 | 7 | 7 |
| 579 | 3301 | 12006406\_6 | Separate multipoint fine-mapping of this locus with independent markers replicated the linkage results (lod 2.64). |A1:\*\*1GP3E0| |A2:\*\*1SP3E3| |A3:\*\*1SP3E3| |A4:\*\*1SP3E3| |A5:\*\*1SP3E1+| |  |  |  |  |  |  |
|  |  |  |  | Annotation | 1GP3E0 | 1SP3E3 | 1SP3E3 | 1SP3E3 | 1SP3E1+ |
|  |  |  |  | Evidence | 1 | 4 | 4 | 4 | 2 |
|  |  |  |  | Focus | 1 | 4 | 4 | 4 | 4 |
|  |  |  |  | Polarity | 7 | 7 | 7 | 7 | 7 |
| 580 | 9014 | 11738031\_19 | Phosphorylated CREB (pCREB), together with a CREB binding protein, which serves as a transcriptional coactivator, |A3:\*\*1SP3E0| |A4:\*\*1SP3E0| regulates transcription for a large class of genes and has been implicated in learning and memory (Silva et al., 1998; Josselyn et al., 2001 ). |A3:\*\*2SP3E2| |A1:\*\*1SP3E2| |A2:\*\*1SP3E2| |A4:\*\*2SP3E2| |A5:\*\*1SP3E2| |  |  |  |  |  |  |
|  |  |  |  | Annotation | 1SP3E2 1SP3E2 | 1SP3E2 1SP3E2 | 1SP3E0 2SP3E2 | 1SP3E0 2SP3E2 | 1SP3E2 1SP3E2 |
|  |  |  |  | Evidence | 3 3 | 3 3 | 1 3 | 1 3 | 3 3 |
|  |  |  |  | Focus | 4 4 | 4 4 | 4 4 | 4 4 | 4 4 |
|  |  |  |  | Polarity | 7 7 | 7 7 | 7 7 | 7 7 | 7 7 |
| 581 | 8651 | 9456323\_81 | CHO-orf4-7 cells were incubated in the presence of 1 mug/ml doxycycline and 50 muM zVAD-fmk for 36 h. Nonadherent and adherent cells were collected, washed in sucrose buffer, and then fixed with 3% glutaraldehyde in 0.1 M cacodylate buffer, pH 7.2-7.4, for 90 min at room temperature. |A1:\*\*1MP3E3| |A2:\*\*1MP3E3| |A3:\*\*1MP3E3| |A4:\*\*1MP3E3| |A5:\*\*1MP3E3| |  |  |  |  |  |  |
|  |  |  |  | Annotation | 1MP3E3 | 1MP3E3 | 1MP3E3 | 1MP3E3 | 1MP3E3 |
|  |  |  |  | Evidence | 4 | 4 | 4 | 4 | 4 |
|  |  |  |  | Focus | 2 | 2 | 2 | 2 | 2 |
|  |  |  |  | Polarity | 7 | 7 | 7 | 7 | 7 |
| 582 | 7717 | 10428976\_379 | The beta barrel structure proposed by Mann et al. (33) |A4:\*\*1SP3E2| for betaC:B may be the triacylglycerol-binding domain of hAPOB that accepts "`shuttled"` monomeric triacylglycerol from the beta barrel structure proposed by Mann et al. (33) for the betaC:MTP domain of hMTP. |A1:\*\*1SP1E2| |A2:\*\*1SP2E2| |A3:\*\*1SP2E2| |A4:\*\*2SP2E2| |A5:\*\*1SP3E2| |  |  |  |  |  |  |
|  |  |  |  | Annotation | 1SP1E2 1SP1E2 | 1SP2E2 1SP2E2 | 1SP2E2 1SP2E2 | 1SP3E2 2SP2E2 | 1SP3E2 1SP3E2 |
|  |  |  |  | Evidence | 3 3 | 3 3 | 3 3 | 3 3 | 3 3 |
|  |  |  |  | Focus | 4 4 | 4 4 | 4 4 | 4 4 | 4 4 |
|  |  |  |  | Polarity | 5 5 | 6 6 | 6 6 | 7 6 | 7 7 |
| 583 | 2332 | 10022929\_11 | Frequently, the protein-protein interactions are mediated by adapter proteins, a group of noncatalytic proteins specialized in mediating multiprotein complex formation. |A1:\*\*1SP3E0| |A2:\*\*1SP3E0| |A3:\*\*1SP2E0| |A4:\*\*1SP2E0| |A5:\*\*1SP3E0| |  |  |  |  |  |  |
|  |  |  |  | Annotation | 1SP3E0 | 1SP3E0 | 1SP2E0 | 1SP2E0 | 1SP3E0 |
|  |  |  |  | Evidence | 1 | 1 | 1 | 1 | 1 |
|  |  |  |  | Focus | 4 | 4 | 4 | 4 | 4 |
|  |  |  |  | Polarity | 7 | 7 | 6 | 6 | 7 |
| 584 | 2088 | 10545115\_287 | Thus, we consider it likely that PML and Daxx are transcriptional modulators whose effects on gene expression (up- or downregulation) |A3:\*\*1SP3E3| |A4:\*\*1SP3E3| may vary depending on interactions with other factors or a variety of circumstances. |A3:\*\*2SP2E0| |A1:\*\*1SP1E3| |A2:\*\*1SP3E1| |A4:\*\*2SP2E0| |A5:\*\*1SP3E3| |  |  |  |  |  |  |
|  |  |  |  | Annotation | 1SP1E3 1SP1E3 | 1SP3E1 1SP3E1 | 1SP3E3 2SP2E0 | 1SP3E3 2SP2E0 | 1SP3E3 1SP3E3 |
|  |  |  |  | Evidence | 4 4 | 2 2 | 4 1 | 4 1 | 4 4 |
|  |  |  |  | Focus | 4 4 | 4 4 | 4 4 | 4 4 | 4 4 |
|  |  |  |  | Polarity | 5 5 | 7 7 | 7 6 | 7 6 | 7 7 |
| 585 | 3517 | 11078735\_41 | Only marginal activity was observed with guanosine, |A3:\*\*1SP1E3| |A4:\*\*1SP1E3| and neither 2', 3'-dideoxynucleosides nor hypoxanthine arabinoside served as substrates. |A3:\*\*2SN3E3| |A1:\*\*1SP3E3| |A2:\*\*1SP3E3| |A4:\*\*2SN3E3| |A5:\*\*1GP3E3| |  |  |  |  |  |  |
|  |  |  |  | Annotation | 1SP3E3 1SP3E3 | 1SP3E3 1SP3E3 | 1SP1E3 2SN3E3 | 1SP1E3 2SN3E3 | 1GP3E3 1GP3E3 |
|  |  |  |  | Evidence | 4 4 | 4 4 | 4 4 | 4 4 | 4 4 |
|  |  |  |  | Focus | 4 4 | 4 4 | 4 4 | 4 4 | 1 1 |
|  |  |  |  | Polarity | 7 7 | 7 7 | 5 1 | 5 1 | 7 7 |
| 586 | 3915 | 10456878\_91 | Plasma was collected by centrifugation and stored at 70 degrees C until the day of the assay. |A1:\*\*1MP3E3| |A2:\*\*1MP3E3| |A3:\*\*1MP3E3| |A4:\*\*1MP3E3| |A5:\*\*1MP3E3| |  |  |  |  |  |  |
|  |  |  |  | Annotation | 1MP3E3 | 1MP3E3 | 1MP3E3 | 1MP3E3 | 1MP3E3 |
|  |  |  |  | Evidence | 4 | 4 | 4 | 4 | 4 |
|  |  |  |  | Focus | 2 | 2 | 2 | 2 | 2 |
|  |  |  |  | Polarity | 7 | 7 | 7 | 7 | 7 |
| 587 | 1680 | 9099694\_45 | REFERENCES Haritos, A. A., Blacher, R., Stein, S., Caldarella, J., and Horecker, B. L. (1985) Proc. |A1:\*\*1GP3E0| |A2:\*\*1GP3E2| |A3:\*\*1SP3E0| |A4:\*\*1SP3E0| |A5:\*\*1GP3E2| |  |  |  |  |  |  |
|  |  |  |  | Annotation | 1GP3E0 | 1GP3E2 | 1SP3E0 | 1SP3E0 | 1GP3E2 |
|  |  |  |  | Evidence | 1 | 3 | 1 | 1 | 3 |
|  |  |  |  | Focus | 1 | 1 | 4 | 4 | 1 |
|  |  |  |  | Polarity | 7 | 7 | 7 | 7 | 7 |
| 588 | 3413 | 11545071\_283 | If it turns out that the rodents are most distantly related to the other groups of mammals, |A3:\*\*1SP2E0| |A5:\*\*1GP2E1| then the K associated with H197Y in the ancestral rodent pigment will be >8. |A3:\*\*2SP3E0| |A1:\*\*1SP2E0| |A2:\*\*1SP2E1| |A4:\*\*1SP3E0| |A5:\*\*2GP3E1| |  |  |  |  |  |  |
|  |  |  |  | Annotation | 1SP2E0 1SP2E0 | 1SP2E1 1SP2E1 | 1SP2E0 2SP3E0 | 1SP3E0 1SP3E0 | 1GP2E1 2GP3E1 |
|  |  |  |  | Evidence | 1 1 | 2 2 | 1 1 | 1 1 | 2 2 |
|  |  |  |  | Focus | 4 4 | 4 4 | 4 4 | 4 4 | 1 1 |
|  |  |  |  | Polarity | 6 6 | 6 6 | 6 7 | 7 7 | 6 7 |
| 589 | 6803 | 11560910\_272 | This work was supported in part by grant MCB-9727906 from the National Science Foundation. |A1:\*\*1GP3E0| |A2:\*\*1GP3E0| |A3:\*\*1SP3E3| |A4:\*\*1SP3E3| |A5:\*\*1GP3E0| |  |  |  |  |  |  |
|  |  |  |  | Annotation | 1GP3E0 | 1GP3E0 | 1SP3E3 | 1SP3E3 | 1GP3E0 |
|  |  |  |  | Evidence | 1 | 1 | 4 | 4 | 1 |
|  |  |  |  | Focus | 1 | 1 | 4 | 4 | 1 |
|  |  |  |  | Polarity | 7 | 7 | 7 | 7 | 7 |
| 590 | 7627 | 11591658\_87 | The nucleotide sequences of the R. rubrum glnK and glnJ regions have been deposited in the GenBank database under accession numbers AF207908 and AF329498, respectively. |A1:\*\*1GP3E1| |A2:\*\*1SP3E3| |A3:\*\*1SP3E3| |A4:\*\*1SP3E3| |A5:\*\*1GP3E0| |  |  |  |  |  |  |
|  |  |  |  | Annotation | 1GP3E1 | 1SP3E3 | 1SP3E3 | 1SP3E3 | 1GP3E0 |
|  |  |  |  | Evidence | 2 | 4 | 4 | 4 | 1 |
|  |  |  |  | Focus | 1 | 4 | 4 | 4 | 1 |
|  |  |  |  | Polarity | 7 | 7 | 7 | 7 | 7 |
| 591 | 4360 | 10625639\_34 | Moreover, the UV-visible spectra of the as-isolated proteins are all similar, with copper(II) d-d bands at ~680 nm and shoulders at ~450 nm. |A1:\*\*1SP3E0| |A2:\*\*1SP3E0| |A3:\*\*1SP3E3| |A4:\*\*1SP3E3| |A5:\*\*1SMP3E3| |  |  |  |  |  |  |
|  |  |  |  | Annotation | 1SP3E0 | 1SP3E0 | 1SP3E3 | 1SP3E3 | 1SMP3E3 |
|  |  |  |  | Evidence | 1 | 1 | 4 | 4 | 4 |
|  |  |  |  | Focus | 4 | 4 | 4 | 4 | 6 |
|  |  |  |  | Polarity | 7 | 7 | 7 | 7 | 7 |
| 592 | 9169 | 9643348\_216 | The work described was supported by a National Institutes of Health (NIH) Program Project Grant, HL41633. |A1:\*\*1GP3E0| |A2:\*\*1GP3E0| |A3:\*\*1SP3E3| |A4:\*\*1SP3E3| |A5:\*\*1GP3E0| |  |  |  |  |  |  |
|  |  |  |  | Annotation | 1GP3E0 | 1GP3E0 | 1SP3E3 | 1SP3E3 | 1GP3E0 |
|  |  |  |  | Evidence | 1 | 1 | 4 | 4 | 1 |
|  |  |  |  | Focus | 1 | 1 | 4 | 4 | 1 |
|  |  |  |  | Polarity | 7 | 7 | 7 | 7 | 7 |
| 593 | 9745 | 11516955\_2 | We found that, when cornering, he spent most of the time looking close to, |A3:\*\*1GP2E3| |A4:\*\*1GP2E3| |A5:\*\*1GP3E3| but not exactly at, the tangent points on the inside edges of the bends. |A3:\*\*2GN1E0| |A1:\*\*1GP3E3| |A2:\*\*1GP3E3| |A4:\*\*2GN1E0| |A5:\*\*2GN3E3| |  |  |  |  |  |  |
|  |  |  |  | Annotation | 1GP3E3 1GP3E3 | 1GP3E3 1GP3E3 | 1GP2E3 2GN1E0 | 1GP2E3 2GN1E0 | 1GP3E3 2GN3E3 |
|  |  |  |  | Evidence | 4 4 | 4 4 | 4 1 | 4 1 | 4 4 |
|  |  |  |  | Focus | 1 1 | 1 1 | 1 1 | 1 1 | 1 1 |
|  |  |  |  | Polarity | 7 7 | 7 7 | 6 3 | 6 3 | 7 1 |
| 594 | 591 | 10978289\_247 | The mRNA can be detected in the germ cells throughout their migration through the midgut and into the gonadal mesoderm (data not shown) |A1:\*\*1SP2E1| |A2:\*\*1SP2E1| and is present in the embryonic gonad of stage 15 embryos ( Fig 6G). |A1:\*\*2SP3E3| |A2:\*\*2SP3E3| |A3:\*\*1SP3E3| |A4:\*\*1SP3E3| |A5:\*\*1SP3E3| |  |  |  |  |  |  |
|  |  |  |  | Annotation | 1SP2E1 2SP3E3 | 1SP2E1 2SP3E3 | 1SP3E3 1SP3E3 | 1SP3E3 1SP3E3 | 1SP3E3 1SP3E3 |
|  |  |  |  | Evidence | 2 4 | 2 4 | 4 4 | 4 4 | 4 4 |
|  |  |  |  | Focus | 4 4 | 4 4 | 4 4 | 4 4 | 4 4 |
|  |  |  |  | Polarity | 6 7 | 6 7 | 7 7 | 7 7 | 7 7 |
| 595 | 7060 | 10330189\_286 | We tested whether strong activation could be observed in normal human T lymphocytes (Fig. 7C). |A1:\*\*1SP1E3| |A2:\*\*1SP2E3| |A3:\*\*1SP2E3| |A4:\*\*1SP2E3| |A5:\*\*1GP3E3| |  |  |  |  |  |  |
|  |  |  |  | Annotation | 1SP1E3 | 1SP2E3 | 1SP2E3 | 1SP2E3 | 1GP3E3 |
|  |  |  |  | Evidence | 4 | 4 | 4 | 4 | 4 |
|  |  |  |  | Focus | 4 | 4 | 4 | 4 | 1 |
|  |  |  |  | Polarity | 5 | 6 | 6 | 6 | 7 |
| 596 | 1017 | 12137974\_3 | AM, ET-1, and NO were detected in all of the follicular fluid samples and their concentrations were similar in spontaneous and stimulated cycles. |A1:\*\*1SP3E3| |A2:\*\*1SP3E3| |A3:\*\*1SP3E3| |A4:\*\*1SP3E3| |A5:\*\*1SP3E3| |  |  |  |  |  |  |
|  |  |  |  | Annotation | 1SP3E3 | 1SP3E3 | 1SP3E3 | 1SP3E3 | 1SP3E3 |
|  |  |  |  | Evidence | 4 | 4 | 4 | 4 | 4 |
|  |  |  |  | Focus | 4 | 4 | 4 | 4 | 4 |
|  |  |  |  | Polarity | 7 | 7 | 7 | 7 | 7 |
| 597 | 594 | 11861563\_9 | This novel numb allele, as well as previously described ones, was shown to affect the fly's rhythm of locomotor activity. |A1:\*\*1GP3E3| |A2:\*\*1SP3E1| |A3:\*\*1SP3E1| |A4:\*\*1SP3E1| |A5:\*\*1SP3E1| |  |  |  |  |  |  |
|  |  |  |  | Annotation | 1GP3E3 | 1SP3E1 | 1SP3E1 | 1SP3E1 | 1SP3E1 |
|  |  |  |  | Evidence | 4 | 2 | 2 | 2 | 2 |
|  |  |  |  | Focus | 1 | 4 | 4 | 4 | 4 |
|  |  |  |  | Polarity | 7 | 7 | 7 | 7 | 7 |
| 598 | 1825 | 12132254\_1 | In the eyes of the intellectually curious William Gies, dentistry and dental education in 1926 was mechanical, empirical, commercial, reparative, and isolated from other disciplines. |A1:\*\*1GP3E3| |A2:\*\*1GP3E0| |A3:\*\*1SP3E1| |A4:\*\*1GP3E3| |A5:\*\*1GP3E0| |  |  |  |  |  |  |
|  |  |  |  | Annotation | 1GP3E3 | 1GP3E0 | 1SP3E1 | 1GP3E3 | 1GP3E0 |
|  |  |  |  | Evidence | 4 | 1 | 2 | 4 | 1 |
|  |  |  |  | Focus | 1 | 1 | 4 | 1 | 1 |
|  |  |  |  | Polarity | 7 | 7 | 7 | 7 | 7 |
| 599 | 7006 | 9367983\_165 | The twofold difference in complex abundance in the presence of TFIID and TFIIA can be compared with the 7-fold and 3.5-fold differences in the strengths of these promoters in vitro and in vivo, respectively (Fig. 1B; Lo and Smale 1996). |A1:\*\*1SP3E3| |A2:\*\*1SP3E2| |A3:\*\*1SP2E2| |A4:\*\*1SP2E2| |A5:\*\*1SP3E2| |  |  |  |  |  |  |
|  |  |  |  | Annotation | 1SP3E3 | 1SP3E2 | 1SP2E2 | 1SP2E2 | 1SP3E2 |
|  |  |  |  | Evidence | 4 | 3 | 3 | 3 | 3 |
|  |  |  |  | Focus | 4 | 4 | 4 | 4 | 4 |
|  |  |  |  | Polarity | 7 | 7 | 6 | 6 | 7 |
| 600 | 1105 | 11438670\_1 | Two alleles of the Drosophila melanogaster Rfc4 ( DmRfc4) gene, which encodes subunit 4 of the replication factor C (RFC) complex, cause striking defects in mitotic chromosome cohesion and condensation. |A1:\*\*1SP3E0| |A2:\*\*1SP3E0| |A3:\*\*1SP3E0| |A4:\*\*1SP3E0| |A5:\*\*1SP3E1| |  |  |  |  |  |  |
|  |  |  |  | Annotation | 1SP3E0 | 1SP3E0 | 1SP3E0 | 1SP3E0 | 1SP3E1 |
|  |  |  |  | Evidence | 1 | 1 | 1 | 1 | 2 |
|  |  |  |  | Focus | 4 | 4 | 4 | 4 | 4 |
|  |  |  |  | Polarity | 7 | 7 | 7 | 7 | 7 |
| 601 | 5312 | 12074291\_3 | Following stabilisation, all three cats underwent exploratory surgery. |A1:\*\*1GP3E0| |A2:\*\*1GP3E0| |A3:\*\*1SP3E0| |A4:\*\*1SP3E0| |A5:\*\*1GP3E3| |  |  |  |  |  |  |
|  |  |  |  | Annotation | 1GP3E0 | 1GP3E0 | 1SP3E0 | 1SP3E0 | 1GP3E3 |
|  |  |  |  | Evidence | 1 | 1 | 1 | 1 | 4 |
|  |  |  |  | Focus | 1 | 1 | 4 | 4 | 1 |
|  |  |  |  | Polarity | 7 | 7 | 7 | 7 | 7 |
| 602 | 4401 | 10992459\_191 | Although it is well known that plasmid DNA derived from bacteria acts as a nonspecific adjuvant in the stimulation of a Th1 response ( 31), |A5:\*\*1SP3E2| |A1:\*\*1SP3E2| |A2:\*\*1SP3E2| |A3:\*\*1SP3E2| |A4:\*\*1SP3E2| several lines of evidence indicate that the protection induced by our multicomponent DNA vaccine was not due to a nonspecific immunostimulatory effect of the vector DNA |A5:\*\*2SN3E3| but rather due to immune responses induced by sequences specific to Leishmania. |A5:\*\*3SP3E3| |A1:\*\*2SP3E1| |A2:\*\*2SP3E1| |A3:\*\*2SN3E1| |A4:\*\*2SN3E1| |  |  |  |  |  |  |
|  |  |  |  | Annotation | 1SP3E2 2SP3E1 2SP3E1 | 1SP3E2 2SP3E1 2SP3E1 | 1SP3E2 2SN3E1 2SN3E1 | 1SP3E2 2SN3E1 2SN3E1 | 1SP3E2 2SN3E3 3SP3E3 |
|  |  |  |  | Evidence | 3 2 2 | 3 2 2 | 3 2 2 | 3 2 2 | 3 4 4 |
|  |  |  |  | Focus | 4 4 4 | 4 4 4 | 4 4 4 | 4 4 4 | 4 4 4 |
|  |  |  |  | Polarity | 7 7 7 | 7 7 7 | 7 1 1 | 7 1 1 | 7 1 7 |
| 603 | 4666 | 11238885\_218 | These data suggest that the physiological state of homozygotes may be normal before and just after birth, but a failure to suckle may contribute to the lethal phenotype. |A1:\*\*1SP1E3| |A2:\*\*1SP3E1| |A3:\*\*1SP2E3| |A4:\*\*1SP2E3| |A5:\*\*1SP2E1| |  |  |  |  |  |  |
|  |  |  |  | Annotation | 1SP1E3 | 1SP3E1 | 1SP2E3 | 1SP2E3 | 1SP2E1 |
|  |  |  |  | Evidence | 4 | 2 | 4 | 4 | 2 |
|  |  |  |  | Focus | 4 | 4 | 4 | 4 | 4 |
|  |  |  |  | Polarity | 5 | 7 | 6 | 6 | 6 |
| 604 | 4157 | 9298899\_246 | All data were integrated and scaled with DENZO and SCALEPACK (Otwinowski, 1993 ) ( Table 1A). |A1:\*\*1GP3E23| |A2:\*\*1GP3E23| |A3:\*\*1SP3E3| |A4:\*\*1SP3E3| |A5:\*\*1GP3E23| |  |  |  |  |  |  |
|  |  |  |  | Annotation | 1GP3E23 | 1GP3E23 | 1SP3E3 | 1SP3E3 | 1GP3E23 |
|  |  |  |  | Evidence | 3 | 3 | 4 | 4 | 3 |
|  |  |  |  | Focus | 1 | 1 | 4 | 4 | 1 |
|  |  |  |  | Polarity | 7 | 7 | 7 | 7 | 7 |
| 605 | 1534 | 10585437\_16 | In the natural environment, modified bases in M. Jannaschii tRNATyr may help to stabilize the tRNA structure above 73 degrees C (which corresponds to the Tm of the transcript). |A1:\*\*1SP1E0| |A2:\*\*1SP2E0| |A3:\*\*1SP2E0| |A4:\*\*1SP2E0| |A5:\*\*1SP2E0| |  |  |  |  |  |  |
|  |  |  |  | Annotation | 1SP1E0 | 1SP2E0 | 1SP2E0 | 1SP2E0 | 1SP2E0 |
|  |  |  |  | Evidence | 1 | 1 | 1 | 1 | 1 |
|  |  |  |  | Focus | 4 | 4 | 4 | 4 | 4 |
|  |  |  |  | Polarity | 5 | 6 | 6 | 6 | 6 |
| 606 | 1813 | 9813103\_186 | Distribution of beta1-integrin and actin in cells plated for 30 min on fibronectin. |A1:\*\*1SP3E3| |A2:\*\*1SP3E0| |A3:\*\*1SP3E0| |A4:\*\*1SP3E0| |A5:\*\*1MP3E0| |  |  |  |  |  |  |
|  |  |  |  | Annotation | 1SP3E3 | 1SP3E0 | 1SP3E0 | 1SP3E0 | 1MP3E0 |
|  |  |  |  | Evidence | 4 | 1 | 1 | 1 | 1 |
|  |  |  |  | Focus | 4 | 4 | 4 | 4 | 2 |
|  |  |  |  | Polarity | 7 | 7 | 7 | 7 | 7 |
| 607 | 9592 | 12235085\_13 | After logistic regression, young age (p=0.0008), female sex (p=0.01), long duration of infection (p=0.0001), and HLA DRB1\*11 DRB1x11 (p=0.050) were more strongly associated with persistence of normal ALT. |A1:\*\*1SP3E3| |A2:\*\*1SP3E3| |A3:\*\*1SP2E3| |A4:\*\*1SP3E3| |A5:\*\*1SP3E3| |  |  |  |  |  |  |
|  |  |  |  | Annotation | 1SP3E3 | 1SP3E3 | 1SP2E3 | 1SP3E3 | 1SP3E3 |
|  |  |  |  | Evidence | 4 | 4 | 4 | 4 | 4 |
|  |  |  |  | Focus | 4 | 4 | 4 | 4 | 4 |
|  |  |  |  | Polarity | 7 | 7 | 6 | 7 | 7 |
| 608 | 5757 | 12431371\_173 | In contrast, as shown in Figures 4B and 4C, when cultured in the absence of survival factors, BAD+/+ and BAD3SA/3SA MEFs underwent equivalent levels of cell death after treatment with anti-Fas antibodies, TNF, irradiation, or etoposide, |A3:\*\*1MP3E3| |A4:\*\*1MP3E3| as might be expected, |A5:\*\*1SP3E3| as BAD is dephosphorylated in the absence of survival factors in both wild-type and BAD3SA/3SA cells. |A5:\*\*2SP3E3-| |A1:\*\*1SP1E3| |A2:\*\*1SP1E3| |A3:\*\*2SP2E0| |A4:\*\*2SP2E0| |  |  |  |  |  |  |
|  |  |  |  | Annotation | 1SP1E3 1SP1E3 1SP1E3 | 1SP1E3 1SP1E3 1SP1E3 | 1MP3E3 2SP2E0 2SP2E0 | 1MP3E3 2SP2E0 2SP2E0 | 1SP3E3 1SP3E3 2SP3E3- |
|  |  |  |  | Evidence | 4 4 4 | 4 4 4 | 4 1 1 | 4 1 1 | 4 4 4 |
|  |  |  |  | Focus | 4 4 4 | 4 4 4 | 2 4 4 | 2 4 4 | 4 4 4 |
|  |  |  |  | Polarity | 5 5 5 | 5 5 5 | 7 6 6 | 7 6 6 | 7 7 7 |
| 609 | 8898 | 10454504\_25 | We reported recently the pharmacological properties of the FP receptor of Swiss mouse 3T3 fibroblasts (Griffin et al., 1997) and A7r5 rat thoracic aorta vascular smooth muscle cells (Griffin et al., 1998 ). |A3:\*\*1SP3E2| |A1:\*\*1SP3E2| |A2:\*\*1SP3E23| |A4:\*\*1SP3E2| |A5:\*\*1GP3E2| |  |  |  |  |  |  |
|  |  |  |  | Annotation | 1SP3E2 | 1SP3E23 | 1SP3E2 | 1SP3E2 | 1GP3E2 |
|  |  |  |  | Evidence | 3 | 3 | 3 | 3 | 3 |
|  |  |  |  | Focus | 4 | 4 | 4 | 4 | 1 |
|  |  |  |  | Polarity | 7 | 7 | 7 | 7 | 7 |
| 610 | 5084 | 9673245\_62 | The invasiveness of C. jejuni isolates was determined according to the method of Wassenaar et al. (60). |A1:\*\*1SP3E23| |A2:\*\*1SP3E23| |A3:\*\*1SP3E2| |A4:\*\*1SP3E2| |A5:\*\*1MP3E2| |  |  |  |  |  |  |
|  |  |  |  | Annotation | 1SP3E23 | 1SP3E23 | 1SP3E2 | 1SP3E2 | 1MP3E2 |
|  |  |  |  | Evidence | 3 | 3 | 3 | 3 | 3 |
|  |  |  |  | Focus | 4 | 4 | 4 | 4 | 2 |
|  |  |  |  | Polarity | 7 | 7 | 7 | 7 | 7 |
| 611 | 3257 | 10653722\_217 | Interestingly, the different bands observed previously were not present in the same cell fractions (Fig. 7). |A1:\*\*1SN3E3| |A2:\*\*1SN3E3| |A3:\*\*1SN3E3| |A4:\*\*1SN3E3| |A5:\*\*1GN3E3| |  |  |  |  |  |  |
|  |  |  |  | Annotation | 1SN3E3 | 1SN3E3 | 1SN3E3 | 1SN3E3 | 1GN3E3 |
|  |  |  |  | Evidence | 4 | 4 | 4 | 4 | 4 |
|  |  |  |  | Focus | 4 | 4 | 4 | 4 | 1 |
|  |  |  |  | Polarity | 1 | 1 | 1 | 1 | 1 |
| 612 | 1461 | 9560277\_14 | Finally, ligand binding studies have reported abnormalities in PFC GABAA receptors in schizophrenia ( 9). |A1:\*\*1SP3E2| |A2:\*\*1SP3E2| |A3:\*\*1SP3E2| |A4:\*\*1SP3E2| |A5:\*\*1SP3E2| |  |  |  |  |  |  |
|  |  |  |  | Annotation | 1SP3E2 | 1SP3E2 | 1SP3E2 | 1SP3E2 | 1SP3E2 |
|  |  |  |  | Evidence | 3 | 3 | 3 | 3 | 3 |
|  |  |  |  | Focus | 4 | 4 | 4 | 4 | 4 |
|  |  |  |  | Polarity | 7 | 7 | 7 | 7 | 7 |
| 613 | 1557 | 8910317\_116 | Comm. 12, 661-669 Valle, V. G. R., Fagian, M. M., Parentoni, L. S., Meinicke, A. R., Vercesi, A. E. (1993) Arch. |A1:\*\*1GP3E0| |A2:\*\*1GP3E0| |A3:\*\*1GP3E0| |A4:\*\*1GP3E0| |A5:\*\*1GP3E2| |  |  |  |  |  |  |
|  |  |  |  | Annotation | 1GP3E0 | 1GP3E0 | 1GP3E0 | 1GP3E0 | 1GP3E2 |
|  |  |  |  | Evidence | 1 | 1 | 1 | 1 | 3 |
|  |  |  |  | Focus | 1 | 1 | 1 | 1 | 1 |
|  |  |  |  | Polarity | 7 | 7 | 7 | 7 | 7 |
| 614 | 9664 | 11713291\_66 | Clearly, hsp70i affects multiple apoptotic pathways, |A1:\*\*1SP3E0| |A3:\*\*1SP3E0| |A4:\*\*1SP3E0| |A5:\*\*1SP3E1| and cell type-specific differences may account for the various points of hsp70i intervention. |A1:\*\*2SP1E0| |A2:\*\*1SP2E0| |A3:\*\*2SP2E0| |A4:\*\*2SP2E0| |A5:\*\*2SP2E0| |  |  |  |  |  |  |
|  |  |  |  | Annotation | 1SP3E0 2SP1E0 | 1SP2E0 1SP2E0 | 1SP3E0 2SP2E0 | 1SP3E0 2SP2E0 | 1SP3E1 2SP2E0 |
|  |  |  |  | Evidence | 1 1 | 1 1 | 1 1 | 1 1 | 2 1 |
|  |  |  |  | Focus | 4 4 | 4 4 | 4 4 | 4 4 | 4 4 |
|  |  |  |  | Polarity | 7 5 | 6 6 | 7 6 | 7 6 | 7 6 |
| 615 | 8860 | 9390512\_109 | A multi-center collaborative effort (Schizophrenia Collaborative Linkage Group for chromosome 22 1996 ) |A4:\*\*1SP3E2| analyzed genotypings of this marker provided by 11 independent research groups worldwide, |A5:\*\*1SP3E1| by employing an affected sib-pair analysis on a sample of 296 affected sib-pairs, |A5:\*\*2MP3E1| and revealed significant allele sharing by affected individuals for this marker. |A5:\*\*3SP3E1| |A1:\*\*1SP3E3| |A2:\*\*1SP3E2| |A3:\*\*1SP3E1| |A4:\*\*2SP3E3| |  |  |  |  |  |  |
|  |  |  |  | Annotation | 1SP3E3 1SP3E3 1SP3E3 1SP3E3 | 1SP3E2 1SP3E2 1SP3E2 1SP3E2 | 1SP3E1 1SP3E1 1SP3E1 1SP3E1 | 1SP3E2 2SP3E3 2SP3E3 2SP3E3 | 1SP3E1 1SP3E1 2MP3E1 3SP3E1 |
|  |  |  |  | Evidence | 4 4 4 4 | 3 3 3 3 | 2 2 2 2 | 3 4 4 4 | 2 2 2 2 |
|  |  |  |  | Focus | 4 4 4 4 | 4 4 4 4 | 4 4 4 4 | 4 4 4 4 | 4 4 2 4 |
|  |  |  |  | Polarity | 7 7 7 7 | 7 7 7 7 | 7 7 7 7 | 7 7 7 7 | 7 7 7 7 |
| 616 | 1247 | 8706137\_83 | Taken together, the results shown in Figure 3 indicated that both ATP binding and specific DNA |A3:\*\*1SP3E3| |A4:\*\*1SP3E3| (i.e., a sequence bearing the two XylR binding sites in the same face of the DNA helix) are required for optimal multimerization of the activator at the Pu enhancer. |A3:\*\*2SP3E0| |A1:\*\*1SP3E3| |A2:\*\*1SP3E3| |A4:\*\*2SP3E0| |A5:\*\*1SP3E3| |  |  |  |  |  |  |
|  |  |  |  | Annotation | 1SP3E3 1SP3E3 | 1SP3E3 1SP3E3 | 1SP3E3 2SP3E0 | 1SP3E3 2SP3E0 | 1SP3E3 1SP3E3 |
|  |  |  |  | Evidence | 4 4 | 4 4 | 4 1 | 4 1 | 4 4 |
|  |  |  |  | Focus | 4 4 | 4 4 | 4 4 | 4 4 | 4 4 |
|  |  |  |  | Polarity | 7 7 | 7 7 | 7 7 | 7 7 | 7 7 |
| 617 | 6112 | 12269295\_2 | A possible common denominator among disrupted processes is the use of attention. |A1:\*\*1GP3E0| |A2:\*\*1GP1E0| |A3:\*\*1GP2E0| |A4:\*\*1GP2E0| |A5:\*\*1GP3E0| |  |  |  |  |  |  |
|  |  |  |  | Annotation | 1GP3E0 | 1GP1E0 | 1GP2E0 | 1GP2E0 | 1GP3E0 |
|  |  |  |  | Evidence | 1 | 1 | 1 | 1 | 1 |
|  |  |  |  | Focus | 1 | 1 | 1 | 1 | 1 |
|  |  |  |  | Polarity | 7 | 5 | 6 | 6 | 7 |
| 618 | 3135 | 11930015\_21 | Most lipoproteins are expressed in many tissues and cell types, including the brain and cerebrospinal fluid ( 6). |A1:\*\*1SP3E2| |A2:\*\*1SP3E2| |A3:\*\*1SP2E2| |A4:\*\*1SP2E2| |A5:\*\*1GP3E2| |  |  |  |  |  |  |
|  |  |  |  | Annotation | 1SP3E2 | 1SP3E2 | 1SP2E2 | 1SP2E2 | 1GP3E2 |
|  |  |  |  | Evidence | 3 | 3 | 3 | 3 | 3 |
|  |  |  |  | Focus | 4 | 4 | 4 | 4 | 1 |
|  |  |  |  | Polarity | 7 | 7 | 6 | 6 | 7 |
| 619 | 9910 | 9566974\_74 | Purified recombinant alpha-catenin (0.1 mg/ml in 100 mM NH4HCO3, pH 7.8) was incubated overnight with endoproteinase Glu-C (V8, 25 mug/ml; Boehringer Mannheim GmbH, Mannheim, Germany). |A1:\*\*1MP3E3| |A2:\*\*1MP3E3| |A3:\*\*1MP3E3| |A4:\*\*1MP3E3| |A5:\*\*1MP3E3| |  |  |  |  |  |  |
|  |  |  |  | Annotation | 1MP3E3 | 1MP3E3 | 1MP3E3 | 1MP3E3 | 1MP3E3 |
|  |  |  |  | Evidence | 4 | 4 | 4 | 4 | 4 |
|  |  |  |  | Focus | 2 | 2 | 2 | 2 | 2 |
|  |  |  |  | Polarity | 7 | 7 | 7 | 7 | 7 |
| 620 | 2401 | 10884434\_14 | First, four of five imaging studies have documented an increase in the striatal accumulation of [18]fluorodopa or [11]dopa in patients with schizophrenia ( 8-12). |A1:\*\*1GP3E2+| |A2:\*\*1SP3E2+| |A3:\*\*1SP1E2+| |A4:\*\*1SP3E2+| |A5:\*\*1GP3E2+| |  |  |  |  |  |  |
|  |  |  |  | Annotation | 1GP3E2+ | 1SP3E2+ | 1SP1E2+ | 1SP3E2+ | 1GP3E2+ |
|  |  |  |  | Evidence | 3 | 3 | 3 | 3 | 3 |
|  |  |  |  | Focus | 1 | 4 | 4 | 4 | 1 |
|  |  |  |  | Polarity | 7 | 7 | 5 | 7 | 7 |
| 621 | 5266 | 11083827\_202 | To confirm the EC7372-induced apoptosis and to quantify the process, we performed flow cytometric experiments. |A1:\*\*1MP3E3| |A2:\*\*1MP3E3| |A3:\*\*1MP3E3| |A4:\*\*1MP3E3| |A5:\*\*1GP3E3| |  |  |  |  |  |  |
|  |  |  |  | Annotation | 1MP3E3 | 1MP3E3 | 1MP3E3 | 1MP3E3 | 1GP3E3 |
|  |  |  |  | Evidence | 4 | 4 | 4 | 4 | 4 |
|  |  |  |  | Focus | 2 | 2 | 2 | 2 | 1 |
|  |  |  |  | Polarity | 7 | 7 | 7 | 7 | 7 |
| 622 | 6449 | 11689693\_322 | Although the molecular mechanisms remain to be characterized, |A5:\*\*1GP3E0| the binding of RhoG to the central coiled coil coiled-coil region of kinectin might elicit structural changes in the whole complex, |A5:\*\*2SP2E0| eventually enhancing kinesin motor activity. |A5:\*\*3SP3E0+| |A1:\*\*1SP1E0| |A2:\*\*1SP1E0| |A3:\*\*1SP3E0| |A4:\*\*1SP2E0| |  |  |  |  |  |  |
|  |  |  |  | Annotation | 1SP1E0 1SP1E0 1SP1E0 | 1SP1E0 1SP1E0 1SP1E0 | 1SP3E0 1SP3E0 1SP3E0 | 1SP2E0 1SP2E0 1SP2E0 | 1GP3E0 2SP2E0 3SP3E0+ |
|  |  |  |  | Evidence | 1 1 1 | 1 1 1 | 1 1 1 | 1 1 1 | 1 1 1 |
|  |  |  |  | Focus | 4 4 4 | 4 4 4 | 4 4 4 | 4 4 4 | 1 4 4 |
|  |  |  |  | Polarity | 5 5 5 | 5 5 5 | 7 7 7 | 6 6 6 | 7 6 7 |
| 623 | 3317 | 9763447\_297 | Therefore, the bright spot(s) were interpreted as mitotic spindle pole bodies. |A1:\*\*1SP3E3| |A2:\*\*1SP3E3| |A3:\*\*1SP3E0| |A4:\*\*1SP3E0| |A5:\*\*1GP3E3| |  |  |  |  |  |  |
|  |  |  |  | Annotation | 1SP3E3 | 1SP3E3 | 1SP3E0 | 1SP3E0 | 1GP3E3 |
|  |  |  |  | Evidence | 4 | 4 | 1 | 1 | 4 |
|  |  |  |  | Focus | 4 | 4 | 4 | 4 | 1 |
|  |  |  |  | Polarity | 7 | 7 | 7 | 7 | 7 |
| 624 | 1005 | 11891243\_1 | Wounding chickpea (Cicer arietinum) internodes or cotyledons resulted in an increase in the steady-state level of copper amine oxidase (CuAO) expression both locally and systemically. |A1:\*\*1SP3E3+| |A2:\*\*1SP3E3+| |A3:\*\*1SP3E1+| |A4:\*\*1SP3E0+| |A5:\*\*1SP3E1+| |  |  |  |  |  |  |
|  |  |  |  | Annotation | 1SP3E3+ | 1SP3E3+ | 1SP3E1+ | 1SP3E0+ | 1SP3E1+ |
|  |  |  |  | Evidence | 4 | 4 | 2 | 1 | 2 |
|  |  |  |  | Focus | 4 | 4 | 4 | 4 | 4 |
|  |  |  |  | Polarity | 7 | 7 | 7 | 7 | 7 |
| 625 | 191 | 12135986\_20 | Similarly, in chick embryos, what appeared to be sustained activation of beta-catenin |A5:\*\*1GP2E2| led to excessive feather and scale morphogenesis ( Noramly et al., 1999; Widelitz et al., 2000). |A1:\*\*1SP3E2| |A2:\*\*1SP3E2+| |A3:\*\*1SP3E2| |A4:\*\*1SP3E2| |A5:\*\*2GP3E2+| |  |  |  |  |  |  |
|  |  |  |  | Annotation | 1SP3E2 1SP3E2 | 1SP3E2+ 1SP3E2+ | 1SP3E2 1SP3E2 | 1SP3E2 1SP3E2 | 1GP2E2 2GP3E2+ |
|  |  |  |  | Evidence | 3 3 | 3 3 | 3 3 | 3 3 | 3 3 |
|  |  |  |  | Focus | 4 4 | 4 4 | 4 4 | 4 4 | 1 1 |
|  |  |  |  | Polarity | 7 7 | 7 7 | 7 7 | 7 7 | 6 7 |
| 626 | 6387 | 9390512\_46 | Theoretical analyses as well as data from recent molecular genetic studies suggest that high genetic heterogeneity exists in schizophrenia (Risch ( Risch 1994 ; Tsuang and Faraone 1995), 1995 ), |A1:\*\*1SP2E2| |A2:\*\*1SP3E2| |A3:\*\*1SP3E2| |A4:\*\*1SP2E2| |A5:\*\*1SP3E2| which, unlike Mendelian type Mendelian-type heterogeneity, has the form of overlapping sets of interacting genes predisposing individuals to the same disease. |A1:\*\*2SP3E0| |A2:\*\*2SP3E0| |A3:\*\*2SP3E0| |A4:\*\*2SP3E0| |A5:\*\*2SN3E0| |  |  |  |  |  |  |
|  |  |  |  | Annotation | 1SP2E2 2SP3E0 | 1SP3E2 2SP3E0 | 1SP3E2 2SP3E0 | 1SP2E2 2SP3E0 | 1SP3E2 2SN3E0 |
|  |  |  |  | Evidence | 3 1 | 3 1 | 3 1 | 3 1 | 3 1 |
|  |  |  |  | Focus | 4 4 | 4 4 | 4 4 | 4 4 | 4 4 |
|  |  |  |  | Polarity | 6 7 | 7 7 | 7 7 | 6 7 | 7 1 |
| 627 | 4133 | 9287303\_27 | Therefore, hydrophobic rather than ionic interactions play an important role in the binding. |A1:\*\*1SP3E0| |A2:\*\*1SP3E0| |A3:\*\*1SP3E0| |A4:\*\*1SP3E3| |A5:\*\*1GP3E0| |  |  |  |  |  |  |
|  |  |  |  | Annotation | 1SP3E0 | 1SP3E0 | 1SP3E0 | 1SP3E3 | 1GP3E0 |
|  |  |  |  | Evidence | 1 | 1 | 1 | 4 | 1 |
|  |  |  |  | Focus | 4 | 4 | 4 | 4 | 1 |
|  |  |  |  | Polarity | 7 | 7 | 7 | 7 | 7 |
| 628 | 1369 | 11134330\_41 | Electrophoretic mobility shift assays (EMSAs) were performed as described elsewhere ( 8). |A1:\*\*1MP3E2| |A2:\*\*1MP3E2| |A3:\*\*1MP3E2| |A4:\*\*1MP3E2| |A5:\*\*1MP3E23| |  |  |  |  |  |  |
|  |  |  |  | Annotation | 1MP3E2 | 1MP3E2 | 1MP3E2 | 1MP3E2 | 1MP3E23 |
|  |  |  |  | Evidence | 3 | 3 | 3 | 3 | 3 |
|  |  |  |  | Focus | 2 | 2 | 2 | 2 | 2 |
|  |  |  |  | Polarity | 7 | 7 | 7 | 7 | 7 |
| 629 | 6326 | 10725337\_359 | Cytokeratin disassembly was primarily mediated by effector caspases, as these inclusions were larger and more pronounced in MCF-7 cells in the presence of exogenously expressed caspases-3 or -7. |A1:\*\*1SP3E3| |A2:\*\*1SP3E3| |A3:\*\*1SP3E1-| |A4:\*\*1SP3E0| |A5:\*\*1SP3E1| |  |  |  |  |  |  |
|  |  |  |  | Annotation | 1SP3E3 | 1SP3E3 | 1SP3E1- | 1SP3E0 | 1SP3E1 |
|  |  |  |  | Evidence | 4 | 4 | 2 | 1 | 2 |
|  |  |  |  | Focus | 4 | 4 | 4 | 4 | 4 |
|  |  |  |  | Polarity | 7 | 7 | 7 | 7 | 7 |
| 630 | 7340 | 9252397\_30 | Fig. 3. Repair of UV-induced cyclobutane pyrimidine dimers along the promoter and transcription initiation site of the human JUN gene in normal human fibroblasts. |A1:\*\*1MP3E3| |A2:\*\*1SP3E3| |A3:\*\*1SP3E3| |A4:\*\*1SP3E3| |A5:\*\*1SP3E3| |  |  |  |  |  |  |
|  |  |  |  | Annotation | 1MP3E3 | 1SP3E3 | 1SP3E3 | 1SP3E3 | 1SP3E3 |
|  |  |  |  | Evidence | 4 | 4 | 4 | 4 | 4 |
|  |  |  |  | Focus | 2 | 4 | 4 | 4 | 4 |
|  |  |  |  | Polarity | 7 | 7 | 7 | 7 | 7 |
| 631 | 3739 | 11923448\_17 | Other evidence of molecular and electrophysiological changes in the medial prefrontal cortex of these animals supports this interpretation (Bertolino et al., 1999 ; O'Donnell et al., 1999 ; Stine et al., 2001). |A1:\*\*1SP2E2| |A2:\*\*1SP3E2| |A3:\*\*1SP3E2| |A4:\*\*1SP3E2| |A5:\*\*1SP3E2| |  |  |  |  |  |  |
|  |  |  |  | Annotation | 1SP2E2 | 1SP3E2 | 1SP3E2 | 1SP3E2 | 1SP3E2 |
|  |  |  |  | Evidence | 3 | 3 | 3 | 3 | 3 |
|  |  |  |  | Focus | 4 | 4 | 4 | 4 | 4 |
|  |  |  |  | Polarity | 6 | 7 | 7 | 7 | 7 |
| 632 | 3195 | 7657661\_13 | Lysates containing the wild type and the mutant B-crystallin were run on 12% gel. |A1:\*\*1MP3E3| |A2:\*\*1MP3E3| |A3:\*\*1MP3E3| |A4:\*\*1MP3E3| |A5:\*\*1MP3E3| |  |  |  |  |  |  |
|  |  |  |  | Annotation | 1MP3E3 | 1MP3E3 | 1MP3E3 | 1MP3E3 | 1MP3E3 |
|  |  |  |  | Evidence | 4 | 4 | 4 | 4 | 4 |
|  |  |  |  | Focus | 2 | 2 | 2 | 2 | 2 |
|  |  |  |  | Polarity | 7 | 7 | 7 | 7 | 7 |
| 633 | 5374 | 12438416\_256 | In contrast, the 75- and 50-kD fragments were detected only in vitro (Fig. 3) |A3:\*\*1MP3E3| |A4:\*\*1MP3E3| |A5:\*\*1SP3E3| and no immunoreactive species of these sizes were observed in vivo using any of the p115 antibodies. |A1:\*\*1SP3E3| |A2:\*\*1SP3E3| |A3:\*\*2MN3E1| |A4:\*\*2MN3E3| |A5:\*\*2SN3E3| |  |  |  |  |  |  |
|  |  |  |  | Annotation | 1SP3E3 1SP3E3 | 1SP3E3 1SP3E3 | 1MP3E3 2MN3E1 | 1MP3E3 2MN3E3 | 1SP3E3 2SN3E3 |
|  |  |  |  | Evidence | 4 4 | 4 4 | 4 2 | 4 4 | 4 4 |
|  |  |  |  | Focus | 4 4 | 4 4 | 2 2 | 2 2 | 4 4 |
|  |  |  |  | Polarity | 7 7 | 7 7 | 7 1 | 7 1 | 7 1 |
| 634 | 3633 | 9802880\_101 | The hybridization buffer contained 0.6 M NaCl, 1 mM EDTA, 10 mM Tris-HCl (pH 7.5), 0.25% SDS, 200 mug/ml yeast tRNA, 1x Denhardt's solution, 10% dextran sulfate, 40% formamide, and 100 ng/ml of the indicated digoxigenin-labeled riboprobe. |A1:\*\*1MP3E0| |A2:\*\*1MP3E3| |A3:\*\*1MP3E3| |A4:\*\*1MP3E3| |A5:\*\*1MP3E3| |  |  |  |  |  |  |
|  |  |  |  | Annotation | 1MP3E0 | 1MP3E3 | 1MP3E3 | 1MP3E3 | 1MP3E3 |
|  |  |  |  | Evidence | 1 | 4 | 4 | 4 | 4 |
|  |  |  |  | Focus | 2 | 2 | 2 | 2 | 2 |
|  |  |  |  | Polarity | 7 | 7 | 7 | 7 | 7 |
| 635 | 2805 | 10653739\_111 | Cell numbers determined for individual production wells were highly variable, depending upon the sampling date and origin of the samples. |A1:\*\*1GP3E3| |A2:\*\*1SP3E3| |A3:\*\*1SP3E0| |A4:\*\*1SP3E0| |A5:\*\*1GP3E0| |  |  |  |  |  |  |
|  |  |  |  | Annotation | 1GP3E3 | 1SP3E3 | 1SP3E0 | 1SP3E0 | 1GP3E0 |
|  |  |  |  | Evidence | 4 | 4 | 1 | 1 | 1 |
|  |  |  |  | Focus | 1 | 4 | 4 | 4 | 1 |
|  |  |  |  | Polarity | 7 | 7 | 7 | 7 | 7 |
| 636 | 6055 | 9575187\_274 | Prior studies have shown that enzymatic processing of APP is altered in AD |A5:\*\*1GP3E1| such that there is reduced cleavage at the alpha-secretase site |A5:\*\*2SP3E1-| |A1:\*\*1SP3E1-| |A2:\*\*1SP3E1-| |A3:\*\*1SP3E2-| |A4:\*\*1SP3E2-| and increased cleavage at the beta- and gamma-secretase sites (for a review, see Refs. |A5:\*\*3SP3E2+| |A1:\*\*2SP3E1+| |A2:\*\*2SP2E2+| |A3:\*\*2SP3E3+| |A4:\*\*2SP3E3+| |  |  |  |  |  |  |
|  |  |  |  | Annotation | 1SP3E1- 1SP3E1- 2SP3E1+ | 1SP3E1- 1SP3E1- 2SP2E2+ | 1SP3E2- 1SP3E2- 2SP3E3+ | 1SP3E2- 1SP3E2- 2SP3E3+ | 1GP3E1 2SP3E1- 3SP3E2+ |
|  |  |  |  | Evidence | 2 2 2 | 2 2 3 | 3 3 4 | 3 3 4 | 2 2 3 |
|  |  |  |  | Focus | 4 4 4 | 4 4 4 | 4 4 4 | 4 4 4 | 1 4 4 |
|  |  |  |  | Polarity | 7 7 7 | 7 7 6 | 7 7 7 | 7 7 7 | 7 7 7 |
| 637 | 7189 | 9763456\_267 | Thus, Box C'' is not equivalent to Box C either in sequence or as a NoLE. |A1:\*\*1GN3E0| |A2:\*\*1GN3E0| |A3:\*\*1SN3E0| |A4:\*\*1SN3E0| |A5:\*\*1GN3E1| |  |  |  |  |  |  |
|  |  |  |  | Annotation | 1GN3E0 | 1GN3E0 | 1SN3E0 | 1SN3E0 | 1GN3E1 |
|  |  |  |  | Evidence | 1 | 1 | 1 | 1 | 2 |
|  |  |  |  | Focus | 1 | 1 | 4 | 4 | 1 |
|  |  |  |  | Polarity | 1 | 1 | 1 | 1 | 1 |
| 638 | 7851 | 8550627\_128 | To test that cross-linking was just intramolecular and did not generate any artifactual binding of labeled rhodanese, |A5:\*\*1SN3E3| GroEL was incubated with native rhodanese instead of denatured rhodanese. |A5:\*\*2MP3E3| |A1:\*\*1SP3E3| |A2:\*\*1SP3E3| |A3:\*\*1SP2E1| |A4:\*\*1SP2E2-| |  |  |  |  |  |  |
|  |  |  |  | Annotation | 1SP3E3 1SP3E3 | 1SP3E3 1SP3E3 | 1SP2E1 1SP2E1 | 1SP2E2- 1SP2E2- | 1SN3E3 2MP3E3 |
|  |  |  |  | Evidence | 4 4 | 4 4 | 2 2 | 3 3 | 4 4 |
|  |  |  |  | Focus | 4 4 | 4 4 | 4 4 | 4 4 | 4 2 |
|  |  |  |  | Polarity | 7 7 | 7 7 | 6 6 | 6 6 | 1 7 |
| 639 | 1270 | 12125911\_3 | We have previously reported that the tyrosine kinase activity of c-Src belonging to the Src family kinase is increased together with altered tyrosine phosphorylation of several cellular proteins in the in vitro model of decidualization. |A1:\*\*1SP3E1+| |A2:\*\*1SP3E1+| |A3:\*\*1SP3E1+| |A4:\*\*1SP3E3+| |A5:\*\*1SP3E1+| |  |  |  |  |  |  |
|  |  |  |  | Annotation | 1SP3E1+ | 1SP3E1+ | 1SP3E1+ | 1SP3E3+ | 1SP3E1+ |
|  |  |  |  | Evidence | 2 | 2 | 2 | 4 | 2 |
|  |  |  |  | Focus | 4 | 4 | 4 | 4 | 4 |
|  |  |  |  | Polarity | 7 | 7 | 7 | 7 | 7 |
| 640 | 4864 | 10878041\_11 | H. pullorum is now known to colonize many chicken flocks and is commonly isolated from the cecal contents and carcasses of slaughtered chickens ( 1, 2). |A1:\*\*1GP3E2| |A2:\*\*1SP3E2| |A3:\*\*1SP3E2| |A4:\*\*1SP3E2| |A5:\*\*1GP3E2| |  |  |  |  |  |  |
|  |  |  |  | Annotation | 1GP3E2 | 1SP3E2 | 1SP3E2 | 1SP3E2 | 1GP3E2 |
|  |  |  |  | Evidence | 3 | 3 | 3 | 3 | 3 |
|  |  |  |  | Focus | 1 | 4 | 4 | 4 | 1 |
|  |  |  |  | Polarity | 7 | 7 | 7 | 7 | 7 |
| 641 | 6405 | 10966427\_147 | Thus, while the E. coli pyk mutant cannot convert PEP to pyruvate via PYK activity, |A1:\*\*1SN2E0| |A2:\*\*1SN3E0| |A3:\*\*1SN3E0| |A4:\*\*1SN3E0| |A5:\*\*1SN3E1| PEP can enter the trichloroacetic acid cycle via PEP carboxylase activity (PEP to oxaloacetate). |A1:\*\*2SP2E0| |A2:\*\*2SP3E0| |A3:\*\*2SP3E0| |A4:\*\*2SP3E0| |A5:\*\*2SP3E0| |  |  |  |  |  |  |
|  |  |  |  | Annotation | 1SN2E0 2SP2E0 | 1SN3E0 2SP3E0 | 1SN3E0 2SP3E0 | 1SN3E0 2SP3E0 | 1SN3E1 2SP3E0 |
|  |  |  |  | Evidence | 1 1 | 1 1 | 1 1 | 1 1 | 2 1 |
|  |  |  |  | Focus | 4 4 | 4 4 | 4 4 | 4 4 | 4 4 |
|  |  |  |  | Polarity | 2 6 | 1 7 | 1 7 | 1 7 | 1 7 |
| 642 | 2540 | 9520407\_49 | Numerals 1 and 30 correspond to the numbering of native tRNA. |A1:\*\*1SP3E0| |A2:\*\*1SP3E0| |A3:\*\*1SP3E0| |A4:\*\*1SP3E0| |A5:\*\*1GP3E0| |  |  |  |  |  |  |
|  |  |  |  | Annotation | 1SP3E0 | 1SP3E0 | 1SP3E0 | 1SP3E0 | 1GP3E0 |
|  |  |  |  | Evidence | 1 | 1 | 1 | 1 | 1 |
|  |  |  |  | Focus | 4 | 4 | 4 | 4 | 1 |
|  |  |  |  | Polarity | 7 | 7 | 7 | 7 | 7 |
| 643 | 4331 | 10391869\_55 | The chi2 test was used in the data analysis of the in vivo protective effects of pentoxifylline. |A1:\*\*1MP3E3| |A2:\*\*1MP3E3| |A3:\*\*1SP3E1| |A4:\*\*1SP3E0| |A5:\*\*1MP3E3| |  |  |  |  |  |  |
|  |  |  |  | Annotation | 1MP3E3 | 1MP3E3 | 1SP3E1 | 1SP3E0 | 1MP3E3 |
|  |  |  |  | Evidence | 4 | 4 | 2 | 1 | 4 |
|  |  |  |  | Focus | 2 | 2 | 4 | 4 | 2 |
|  |  |  |  | Polarity | 7 | 7 | 7 | 7 | 7 |
| 644 | 7521 | 10454382\_99 | All HU flies were subjected to paraffin histology after the behavioral experiment. |A1:\*\*1SP3E3| |A2:\*\*1SP3E3| |A3:\*\*1SP3E1| |A4:\*\*1SP3E0| |A5:\*\*1MP3E3| |  |  |  |  |  |  |
|  |  |  |  | Annotation | 1SP3E3 | 1SP3E3 | 1SP3E1 | 1SP3E0 | 1MP3E3 |
|  |  |  |  | Evidence | 4 | 4 | 2 | 1 | 4 |
|  |  |  |  | Focus | 4 | 4 | 4 | 4 | 2 |
|  |  |  |  | Polarity | 7 | 7 | 7 | 7 | 7 |
| 645 | 9661 | 10207001\_23 | However, questions still remain as to whether the E2 transactivator functions within nuclear speckles and whether the speckle pattern of E2 expression is critical for E2's function in facilitating splicing. |A1:\*\*1SP0E0| |A2:\*\*1SP1E0| |A3:\*\*1SP3E0| |A4:\*\*1SP2E0| |A5:\*\*1SP1E0| |  |  |  |  |  |  |
|  |  |  |  | Annotation | 1SP0E0 | 1SP1E0 | 1SP3E0 | 1SP2E0 | 1SP1E0 |
|  |  |  |  | Evidence | 1 | 1 | 1 | 1 | 1 |
|  |  |  |  | Focus | 4 | 4 | 4 | 4 | 4 |
|  |  |  |  | Polarity | 4 | 5 | 7 | 6 | 5 |
| 646 | 6918 | 9663388\_6 | Newly forming ER tubules extended only in a microtubule plus-end direction towards the cell periphery: |A5:\*\*1SP3E3| 31.4% by TACs and 68.6% by the membrane sliding mechanism. |A5:\*\*2MP3E3| |A1:\*\*1SP3E0| |A2:\*\*1SP3E0| |A3:\*\*1SP3E3| |A4:\*\*1MP3E3| |  |  |  |  |  |  |
|  |  |  |  | Annotation | 1SP3E0 1SP3E0 | 1SP3E0 1SP3E0 | 1SP3E3 1SP3E3 | 1MP3E3 1MP3E3 | 1SP3E3 2MP3E3 |
|  |  |  |  | Evidence | 1 1 | 1 1 | 4 4 | 4 4 | 4 4 |
|  |  |  |  | Focus | 4 4 | 4 4 | 4 4 | 2 2 | 4 2 |
|  |  |  |  | Polarity | 7 7 | 7 7 | 7 7 | 7 7 | 7 7 |
| 647 | 5718 | 9396787\_59 | The 125I-labeled material was synthesized at Amersham (Arlington Heights, IL) using the same starting peptide and radioactive Bolton-Hunter reagent. |A1:\*\*1GP3E3| |A2:\*\*1MP3E3| |A3:\*\*1SP3E1| |A4:\*\*1SP3E3| |A5:\*\*1MP3E3| |  |  |  |  |  |  |
|  |  |  |  | Annotation | 1GP3E3 | 1MP3E3 | 1SP3E1 | 1SP3E3 | 1MP3E3 |
|  |  |  |  | Evidence | 4 | 4 | 2 | 4 | 4 |
|  |  |  |  | Focus | 1 | 2 | 4 | 4 | 2 |
|  |  |  |  | Polarity | 7 | 7 | 7 | 7 | 7 |
| 648 | 8072 | 11278665\_3 | Here we determine that similar mechanisms are operational in regulating the apoptotic signaling of other death receptors. |A1:\*\*1GP3E3| |A2:\*\*1SP3E3| |A3:\*\*1SP3E3| |A4:\*\*1SP3E3| |A5:\*\*1GP3E3| |  |  |  |  |  |  |
|  |  |  |  | Annotation | 1GP3E3 | 1SP3E3 | 1SP3E3 | 1SP3E3 | 1GP3E3 |
|  |  |  |  | Evidence | 4 | 4 | 4 | 4 | 4 |
|  |  |  |  | Focus | 1 | 4 | 4 | 4 | 1 |
|  |  |  |  | Polarity | 7 | 7 | 7 | 7 | 7 |
| 649 | 2679 | 12871907\_278 | The values of their major axes (in micrometers) and fluorescence intensities (rfiu; relative fluorescent intensity unit) are arranged in a scatter plot in Fig 9. |A1:\*\*1SP3E3| |A2:\*\*1SP3E3| |A3:\*\*1SP3E3| |A4:\*\*1SP3E3| |A5:\*\*1MP3E3| |  |  |  |  |  |  |
|  |  |  |  | Annotation | 1SP3E3 | 1SP3E3 | 1SP3E3 | 1SP3E3 | 1MP3E3 |
|  |  |  |  | Evidence | 4 | 4 | 4 | 4 | 4 |
|  |  |  |  | Focus | 4 | 4 | 4 | 4 | 2 |
|  |  |  |  | Polarity | 7 | 7 | 7 | 7 | 7 |
| 650 | 4 | 9425154\_262 | There, syntaxin was shown to suffice for alpha-SNAP binding. |A1:\*\*1SP3E3| |A2:\*\*1SP3E1| |A3:\*\*1SP3E1| |A4:\*\*1SP3E1| |A5:\*\*1GP3E1| |  |  |  |  |  |  |
|  |  |  |  | Annotation | 1SP3E3 | 1SP3E1 | 1SP3E1 | 1SP3E1 | 1GP3E1 |
|  |  |  |  | Evidence | 4 | 2 | 2 | 2 | 2 |
|  |  |  |  | Focus | 4 | 4 | 4 | 4 | 1 |
|  |  |  |  | Polarity | 7 | 7 | 7 | 7 | 7 |
| 651 | 7870 | 10801849\_83 | Control lanes 1, 2, 7, and 8 were as in A. |A5:\*\*1GP3E3| C, in vitro translated p59 was mixed with in vitro translated p17 ( lane 4), p12 ( lane 5), or p17 and p12 ( lane 6). |A1:\*\*1MP3E3| |A2:\*\*1MP3E3| |A3:\*\*1MP3E3| |A4:\*\*1MP3E3| |A5:\*\*2MP3E3| |  |  |  |  |  |  |
|  |  |  |  | Annotation | 1MP3E3 1MP3E3 | 1MP3E3 1MP3E3 | 1MP3E3 1MP3E3 | 1MP3E3 1MP3E3 | 1GP3E3 2MP3E3 |
|  |  |  |  | Evidence | 4 4 | 4 4 | 4 4 | 4 4 | 4 4 |
|  |  |  |  | Focus | 2 2 | 2 2 | 2 2 | 2 2 | 1 2 |
|  |  |  |  | Polarity | 7 7 | 7 7 | 7 7 | 7 7 | 7 7 |
| 652 | 4587 | 12095722\_1 | In order to determine which technical aspects of the Austin Moore hemiarthroplasty procedure affect the outcome, we reviewed 243 patients with a non-pathological intracapsular femoral neck fracture who had, Austin Moore uncemented hemiarthroplasty. |A1:\*\*1GP3E3| |A2:\*\*1SP3E3| |A3:\*\*1SP3E3| |A4:\*\*1SP3E3| |A5:\*\*1MP3E3| |  |  |  |  |  |  |
|  |  |  |  | Annotation | 1GP3E3 | 1SP3E3 | 1SP3E3 | 1SP3E3 | 1MP3E3 |
|  |  |  |  | Evidence | 4 | 4 | 4 | 4 | 4 |
|  |  |  |  | Focus | 1 | 4 | 4 | 4 | 2 |
|  |  |  |  | Polarity | 7 | 7 | 7 | 7 | 7 |
| 653 | 9636 | 11029439\_54 | Mutants were grown in broth in the absence of chloramphenicol to allow loss of the resident pVJT128 plasmid, as described previously (38). |A1:\*\*1SP3E23| |A2:\*\*1SP3E23| |A3:\*\*1SP3E2-| |A4:\*\*1SP3E2-| |A5:\*\*1MP3E2| |  |  |  |  |  |  |
|  |  |  |  | Annotation | 1SP3E23 | 1SP3E23 | 1SP3E2- | 1SP3E2- | 1MP3E2 |
|  |  |  |  | Evidence | 3 | 3 | 3 | 3 | 3 |
|  |  |  |  | Focus | 4 | 4 | 4 | 4 | 2 |
|  |  |  |  | Polarity | 7 | 7 | 7 | 7 | 7 |
| 654 | 8610 | 9763432\_98 | Whereas the inhibitor did not significantly influence recovery of total BAX associated with mitochondria (Fig. 2 C, lanes 3 and 4), |A2:\*\*1SN3E3| |A3:\*\*1SN3E3| |A4:\*\*1SN3E3| |A5:\*\*1SN3E3| it reduced the amount of alkaline-resistant membrane-integrated BAX |A1:\*\*1SN3E3-| that was recovered with the organelle (Fig. 2 C, lanes 7 and 8). |A1:\*\*2SP3E3+| |A2:\*\*2SP3E3-| |A3:\*\*2SP3E3-| |A4:\*\*2SP3E3-| |A5:\*\*2SP3E3-| |  |  |  |  |  |  |
|  |  |  |  | Annotation | 1SN3E3- 1SN3E3- 2SP3E3+ | 1SN3E3 2SP3E3- 2SP3E3- | 1SN3E3 2SP3E3- 2SP3E3- | 1SN3E3 2SP3E3- 2SP3E3- | 1SN3E3 2SP3E3- 2SP3E3- |
|  |  |  |  | Evidence | 4 4 4 | 4 4 4 | 4 4 4 | 4 4 4 | 4 4 4 |
|  |  |  |  | Focus | 4 4 4 | 4 4 4 | 4 4 4 | 4 4 4 | 4 4 4 |
|  |  |  |  | Polarity | 1 1 7 | 1 7 7 | 1 7 7 | 1 7 7 | 1 7 7 |
| 655 | 6257 | 10966383\_227 | It is noteworthy that the enzyme is quite stable even in 8 M urea, which causes complete denaturation of ordinary proteins. |A1:\*\*1SP3E0| |A2:\*\*1SP3E0-| |A3:\*\*1SP3E0| |A4:\*\*1SP3E3| |A5:\*\*1SP3E3| |  |  |  |  |  |  |
|  |  |  |  | Annotation | 1SP3E0 | 1SP3E0- | 1SP3E0 | 1SP3E3 | 1SP3E3 |
|  |  |  |  | Evidence | 1 | 1 | 1 | 4 | 4 |
|  |  |  |  | Focus | 4 | 4 | 4 | 4 | 4 |
|  |  |  |  | Polarity | 7 | 7 | 7 | 7 | 7 |
| 656 | 4205 | 9447995\_293 | It was observed that XAP2 was present in the 9S form under these new conditions (Fig. 8B and E). |A1:\*\*1SP3E3| |A2:\*\*1SP3E3| |A3:\*\*1SP3E3| |A4:\*\*1SP3E3| |A5:\*\*1SP3E3| |  |  |  |  |  |  |
|  |  |  |  | Annotation | 1SP3E3 | 1SP3E3 | 1SP3E3 | 1SP3E3 | 1SP3E3 |
|  |  |  |  | Evidence | 4 | 4 | 4 | 4 | 4 |
|  |  |  |  | Focus | 4 | 4 | 4 | 4 | 4 |
|  |  |  |  | Polarity | 7 | 7 | 7 | 7 | 7 |
| 657 | 122 | 9407036\_28 | Additionally, maximal induction of the PMR2A/ENA1 gene |A4:\*\*1SP3E0+| in response to high environmental salt requires calcineurin activation by Ca2+/calmodulin (Garciadeblas et al. 1993; Cunningham and Fink 1996 ). |A4:\*\*2SP3E2+| |A1:\*\*1SP3E2| |A2:\*\*1SP3E2+| |A3:\*\*1SP3E2+| |A5:\*\*1SP3E2| |  |  |  |  |  |  |
|  |  |  |  | Annotation | 1SP3E2 1SP3E2 | 1SP3E2+ 1SP3E2+ | 1SP3E2+ 1SP3E2+ | 1SP3E0+ 2SP3E2+ | 1SP3E2 1SP3E2 |
|  |  |  |  | Evidence | 3 3 | 3 3 | 3 3 | 1 3 | 3 3 |
|  |  |  |  | Focus | 4 4 | 4 4 | 4 4 | 4 4 | 4 4 |
|  |  |  |  | Polarity | 7 7 | 7 7 | 7 7 | 7 7 | 7 7 |
| 658 | 6755 | 9169478\_14 | p21 ras, which is activated by IL-2 ( , ), has also been suggested as a proximal activator for the kinase ( ). |A1:\*\*1SP3E2| |A2:\*\*1SP3E2+| |A3:\*\*1SP2E0| |A4:\*\*1SP2E1| |A5:\*\*1SP3E2| |  |  |  |  |  |  |
|  |  |  |  | Annotation | 1SP3E2 | 1SP3E2+ | 1SP2E0 | 1SP2E1 | 1SP3E2 |
|  |  |  |  | Evidence | 3 | 3 | 1 | 2 | 3 |
|  |  |  |  | Focus | 4 | 4 | 4 | 4 | 4 |
|  |  |  |  | Polarity | 7 | 7 | 6 | 6 | 7 |
| 659 | 321 | 9837930\_186 | Noteworthy is that the majority of beta-cells oscillate at the control glucose concentration of 11 mM. |A1:\*\*1GP3E0| |A2:\*\*1SP3E0| |A3:\*\*1SP3E0| |A4:\*\*1SP3E0| |A5:\*\*1SP3E1| |  |  |  |  |  |  |
|  |  |  |  | Annotation | 1GP3E0 | 1SP3E0 | 1SP3E0 | 1SP3E0 | 1SP3E1 |
|  |  |  |  | Evidence | 1 | 1 | 1 | 1 | 2 |
|  |  |  |  | Focus | 1 | 4 | 4 | 4 | 4 |
|  |  |  |  | Polarity | 7 | 7 | 7 | 7 | 7 |
| 660 | 3827 | 0009835588\_22 | Traditional plating techniques commonly result in assessment of the diversity of less than 10% of the total bacterial community present (23, 29). |A1:\*\*1SP3E2| |A2:\*\*1SP3E2| |A3:\*\*1MP3E3| |A4:\*\*1MP3E3| |A5:\*\*1GP3E2| |  |  |  |  |  |  |
|  |  |  |  | Annotation | 1SP3E2 | 1SP3E2 | 1MP3E3 | 1MP3E3 | 1GP3E2 |
|  |  |  |  | Evidence | 3 | 3 | 4 | 4 | 3 |
|  |  |  |  | Focus | 4 | 4 | 2 | 2 | 1 |
|  |  |  |  | Polarity | 7 | 7 | 7 | 7 | 7 |
| 661 | 1592 | 10985343\_206 | Scanning the specimens either sequentially or simultaneously using the three excitation lines in the krypton-argon laser (488 nm for DiO, 568 nm for DiI, and 647 nm for DiD) with three separate barrier filter sets (522 plus-or-minus 35 nm for DiO, 580 plus-or-minus 32 nm for DiI, and 680 plus-or-minus 32 nm for DiD) gave good separation of the three image planes. |A1:\*\*1SP3E0| |A2:\*\*1SP3E0| |A3:\*\*1MP3E3| |A4:\*\*1MP3E3| |A5:\*\*1MP3E3| |  |  |  |  |  |  |
|  |  |  |  | Annotation | 1SP3E0 | 1SP3E0 | 1MP3E3 | 1MP3E3 | 1MP3E3 |
|  |  |  |  | Evidence | 1 | 1 | 4 | 4 | 4 |
|  |  |  |  | Focus | 4 | 4 | 2 | 2 | 2 |
|  |  |  |  | Polarity | 7 | 7 | 7 | 7 | 7 |
| 662 | 2283 | 12058018\_193 | Yet the cta4-null mutant displayed pleiotropic cellular phenotypes. |A1:\*\*1SP3E3| |A2:\*\*1SP3E1| |A3:\*\*1SP3E0| |A4:\*\*1SP3E0| |A5:\*\*1SP3E1| |  |  |  |  |  |  |
|  |  |  |  | Annotation | 1SP3E3 | 1SP3E1 | 1SP3E0 | 1SP3E0 | 1SP3E1 |
|  |  |  |  | Evidence | 4 | 2 | 1 | 1 | 2 |
|  |  |  |  | Focus | 4 | 4 | 4 | 4 | 4 |
|  |  |  |  | Polarity | 7 | 7 | 7 | 7 | 7 |
| 663 | 756 | 11248093\_29 | We also show that DCC recruits caspase-3 and caspase-9, resulting in the activation of caspase-3 via caspase-9. |A1:\*\*1SP3E3+| |A2:\*\*1SP3E3+| |A3:\*\*1SP3E3+| |A4:\*\*1SP3E3+| |A5:\*\*1SP3E3+| |  |  |  |  |  |  |
|  |  |  |  | Annotation | 1SP3E3+ | 1SP3E3+ | 1SP3E3+ | 1SP3E3+ | 1SP3E3+ |
|  |  |  |  | Evidence | 4 | 4 | 4 | 4 | 4 |
|  |  |  |  | Focus | 4 | 4 | 4 | 4 | 4 |
|  |  |  |  | Polarity | 7 | 7 | 7 | 7 | 7 |
| 664 | 5145 | 12473692\_221 | Other crest derivatives such as sympathetic ganglia (sg), the enteric nervous system (ENS), and Schwann cell precursors along peripheral nerves (sp) form independently of beta-catenin activity. |A1:\*\*1SP3E0| |A2:\*\*1SP3E0| |A3:\*\*1SP3E0| |A4:\*\*1SP3E0| |A5:\*\*1GP3E0| |  |  |  |  |  |  |
|  |  |  |  | Annotation | 1SP3E0 | 1SP3E0 | 1SP3E0 | 1SP3E0 | 1GP3E0 |
|  |  |  |  | Evidence | 1 | 1 | 1 | 1 | 1 |
|  |  |  |  | Focus | 4 | 4 | 4 | 4 | 1 |
|  |  |  |  | Polarity | 7 | 7 | 7 | 7 | 7 |
| 665 | 5422 | 9566919\_25 | IkappaB proteins sequester these transcription factors in the cytoplasm |A5:\*\*1SP3E2| and inhibit their DNA binding (reviewed in references 6, 8, and 28). |A5:\*\*2SP3E2-| |A1:\*\*1SN3E2| |A2:\*\*1SP3E2-| |A3:\*\*1SP3E3-| |A4:\*\*1SP3E3-| |  |  |  |  |  |  |
|  |  |  |  | Annotation | 1SN3E2 1SN3E2 | 1SP3E2- 1SP3E2- | 1SP3E3- 1SP3E3- | 1SP3E3- 1SP3E3- | 1SP3E2 2SP3E2- |
|  |  |  |  | Evidence | 3 3 | 3 3 | 4 4 | 4 4 | 3 3 |
|  |  |  |  | Focus | 4 4 | 4 4 | 4 4 | 4 4 | 4 4 |
|  |  |  |  | Polarity | 1 1 | 7 7 | 7 7 | 7 7 | 7 7 |
| 666 | 6518 | 10531276\_100 | Then the coverslips were transferred to a 24-well plate. |A1:\*\*1MP3E3| |A2:\*\*1MP3E3| |A3:\*\*1MP3E3| |A4:\*\*1MP3E3| |A5:\*\*1MP3E3| |  |  |  |  |  |  |
|  |  |  |  | Annotation | 1MP3E3 | 1MP3E3 | 1MP3E3 | 1MP3E3 | 1MP3E3 |
|  |  |  |  | Evidence | 4 | 4 | 4 | 4 | 4 |
|  |  |  |  | Focus | 2 | 2 | 2 | 2 | 2 |
|  |  |  |  | Polarity | 7 | 7 | 7 | 7 | 7 |
| 667 | 2203 | 10966423\_126 | When in log-phase growth, the mutant is more resistant than the parent strain to H2O2 and tBOOH killing, |A3:\*\*1SP3E0| |A4:\*\*1SP3E0| |A5:\*\*1SP3E2| but not to MD killing (6). |A3:\*\*2SN3E2| |A1:\*\*1SP3E2| |A2:\*\*1SP3E2| |A4:\*\*2SN3E2| |A5:\*\*2SN3E2| |  |  |  |  |  |  |
|  |  |  |  | Annotation | 1SP3E2 1SP3E2 | 1SP3E2 1SP3E2 | 1SP3E0 2SN3E2 | 1SP3E0 2SN3E2 | 1SP3E2 2SN3E2 |
|  |  |  |  | Evidence | 3 3 | 3 3 | 1 3 | 1 3 | 3 3 |
|  |  |  |  | Focus | 4 4 | 4 4 | 4 4 | 4 4 | 4 4 |
|  |  |  |  | Polarity | 7 7 | 7 7 | 7 1 | 7 1 | 7 1 |
| 668 | 2536 | 11160439\_14 | Targeting of neuropeptide neuromodulator systems capable of concomitantly regulating all affected transmitter systems |A3:\*\*1SP3E0| |A4:\*\*1SP3E0| may therefore be a promising approach for the development of increasingly effective and side-effect-free antipsychotic drugs. |A3:\*\*2SP2E0+| |A1:\*\*1SP1E0| |A2:\*\*1SP2E0| |A4:\*\*2SP2E0+| |A5:\*\*1SP3E1| |  |  |  |  |  |  |
|  |  |  |  | Annotation | 1SP1E0 1SP1E0 | 1SP2E0 1SP2E0 | 1SP3E0 2SP2E0+ | 1SP3E0 2SP2E0+ | 1SP3E1 1SP3E1 |
|  |  |  |  | Evidence | 1 1 | 1 1 | 1 1 | 1 1 | 2 2 |
|  |  |  |  | Focus | 4 4 | 4 4 | 4 4 | 4 4 | 4 4 |
|  |  |  |  | Polarity | 5 5 | 6 6 | 7 6 | 7 6 | 7 7 |
| 669 | 5055 | 9362059\_60 | To detect the long C-strands from newly synthesized telomeres |A5:\*\*1SP3E3| but not the shorter mature macronuclear telomeres, |A5:\*\*2SN3E3| |A3:\*\*1SN3E0| |A4:\*\*1SN3E0| the filters were hybridized overnight with a 5 end-labeled 64-base oligonucleotide (G4T4)8 at 46 degrees C in 400 mM NaCl, 10 mM Tris, pH 8.0, 1 mM EDTA, 50% formamide, 0.1% SDS, and 5x Denhardt's solution. |A5:\*\*3MP3E3| |A1:\*\*1MP3E3| |A2:\*\*1MP3E3| |A3:\*\*2MP3E3| |A4:\*\*2MP3E3| |  |  |  |  |  |  |
|  |  |  |  | Annotation | 1MP3E3 1MP3E3 1MP3E3 | 1MP3E3 1MP3E3 1MP3E3 | 1SN3E0 1SN3E0 2MP3E3 | 1SN3E0 1SN3E0 2MP3E3 | 1SP3E3 2SN3E3 3MP3E3 |
|  |  |  |  | Evidence | 4 4 4 | 4 4 4 | 1 1 4 | 1 1 4 | 4 4 4 |
|  |  |  |  | Focus | 2 2 2 | 2 2 2 | 4 4 2 | 4 4 2 | 4 4 2 |
|  |  |  |  | Polarity | 7 7 7 | 7 7 7 | 1 1 7 | 1 1 7 | 7 1 7 |
| 670 | 4449 | 10618246\_209 | Although many factors may affect the growth of specific bacteria at different root locations, |A3:\*\*1SP2E0| |A4:\*\*1SP2E0| |A5:\*\*1GP3E0| plant iron nutritional status explained approximately 20 to 40% of the total variation in community structure at all of the root locations that were sampled. |A3:\*\*2MP3E3| |A1:\*\*1GP3E3| |A2:\*\*1SP3E3| |A4:\*\*2MP3E3| |A5:\*\*2GP3E1| |  |  |  |  |  |  |
|  |  |  |  | Annotation | 1GP3E3 1GP3E3 | 1SP3E3 1SP3E3 | 1SP2E0 2MP3E3 | 1SP2E0 2MP3E3 | 1GP3E0 2GP3E1 |
|  |  |  |  | Evidence | 4 4 | 4 4 | 1 4 | 1 4 | 1 2 |
|  |  |  |  | Focus | 1 1 | 4 4 | 4 2 | 4 2 | 1 1 |
|  |  |  |  | Polarity | 7 7 | 7 7 | 6 7 | 6 7 | 7 7 |
| 671 | 6574 | 12136945\_5 | In the patients with malignant lymphoma, parallel changes in serum sIL-2R and urine neopterin were observed. |A1:\*\*1SP3E3| |A2:\*\*1SP3E3| |A3:\*\*1SP3E3| |A4:\*\*1SP3E3| |A5:\*\*1GP3E3| |  |  |  |  |  |  |
|  |  |  |  | Annotation | 1SP3E3 | 1SP3E3 | 1SP3E3 | 1SP3E3 | 1GP3E3 |
|  |  |  |  | Evidence | 4 | 4 | 4 | 4 | 4 |
|  |  |  |  | Focus | 4 | 4 | 4 | 4 | 1 |
|  |  |  |  | Polarity | 7 | 7 | 7 | 7 | 7 |
| 672 | 6764 | 9573050\_80 | Gels were blotted and probed with anti lambda repressor anti-lambda-repressor antibody. |A1:\*\*1MP3E3| |A2:\*\*1MP3E3| |A3:\*\*1MP3E3| |A4:\*\*1MP3E3| |A5:\*\*1MP3E3| |  |  |  |  |  |  |
|  |  |  |  | Annotation | 1MP3E3 | 1MP3E3 | 1MP3E3 | 1MP3E3 | 1MP3E3 |
|  |  |  |  | Evidence | 4 | 4 | 4 | 4 | 4 |
|  |  |  |  | Focus | 2 | 2 | 2 | 2 | 2 |
|  |  |  |  | Polarity | 7 | 7 | 7 | 7 | 7 |
| 673 | 1028 | 9696748\_41 | LPS and OmpA may thus be contributed by the recipient cell to the electron-dense region mentioned above |A5:\*\*1SP3E3| and are postulated to act as receptors during conjugation. |A5:\*\*2SP2E3| |A1:\*\*1SP1E0| |A2:\*\*1SP2E0| |A3:\*\*1SP2E0| |A4:\*\*1SP2E3| |  |  |  |  |  |  |
|  |  |  |  | Annotation | 1SP1E0 1SP1E0 | 1SP2E0 1SP2E0 | 1SP2E0 1SP2E0 | 1SP2E3 1SP2E3 | 1SP3E3 2SP2E3 |
|  |  |  |  | Evidence | 1 1 | 1 1 | 1 1 | 4 4 | 4 4 |
|  |  |  |  | Focus | 4 4 | 4 4 | 4 4 | 4 4 | 4 4 |
|  |  |  |  | Polarity | 5 5 | 6 6 | 6 6 | 6 6 | 7 6 |
| 674 | 8848 | 9472020\_312 | The presence of HCR did not affect binary complex formation. |A1:\*\*1GN3E0| |A2:\*\*1SN3E0| |A3:\*\*1SN3E0| |A4:\*\*1SN3E0| |A5:\*\*1GN3E0| |  |  |  |  |  |  |
|  |  |  |  | Annotation | 1GN3E0 | 1SN3E0 | 1SN3E0 | 1SN3E0 | 1GN3E0 |
|  |  |  |  | Evidence | 1 | 1 | 1 | 1 | 1 |
|  |  |  |  | Focus | 1 | 4 | 4 | 4 | 1 |
|  |  |  |  | Polarity | 1 | 1 | 1 | 1 | 1 |
| 675 | 9850 | 12139595\_1 | BACKGROUND: Nitric oxide (NO), when inhaled, has a synergistic effect with airway recruitment strategies such as positive endexpiratory pressure (PEEP) or continuous positive airway pressure (CPAP) in improving oxygenation in lung injury. |A1:\*\*1SP3E0| |A2:\*\*1SP3E0| |A3:\*\*1SP3E0| |A4:\*\*1SP3E0| |A5:\*\*1SP3E0| |  |  |  |  |  |  |
|  |  |  |  | Annotation | 1SP3E0 | 1SP3E0 | 1SP3E0 | 1SP3E0 | 1SP3E0 |
|  |  |  |  | Evidence | 1 | 1 | 1 | 1 | 1 |
|  |  |  |  | Focus | 4 | 4 | 4 | 4 | 4 |
|  |  |  |  | Polarity | 7 | 7 | 7 | 7 | 7 |
| 676 | 4522 | 10617567\_12 | A requirement for Wnt3a in the specification of trunk and tail paraxial somitic mesoderm fates has been demonstrated by mutant analyses. |A1:\*\*1SP3E1| |A2:\*\*1SP3E1| |A3:\*\*1SP3E1| |A4:\*\*1SP3E3| |A5:\*\*1GP3E1| |  |  |  |  |  |  |
|  |  |  |  | Annotation | 1SP3E1 | 1SP3E1 | 1SP3E1 | 1SP3E3 | 1GP3E1 |
|  |  |  |  | Evidence | 2 | 2 | 2 | 4 | 2 |
|  |  |  |  | Focus | 4 | 4 | 4 | 4 | 1 |
|  |  |  |  | Polarity | 7 | 7 | 7 | 7 | 7 |
| 677 | 8725 | 11133470\_80 | The highest percentages were detected in the upper layers (0- to 3.25-cm depth). |A1:\*\*1GP3E3| |A2:\*\*1GP3E3| |A3:\*\*1GP3E3| |A4:\*\*1GP3E3| |A5:\*\*1GP3E3| |  |  |  |  |  |  |
|  |  |  |  | Annotation | 1GP3E3 | 1GP3E3 | 1GP3E3 | 1GP3E3 | 1GP3E3 |
|  |  |  |  | Evidence | 4 | 4 | 4 | 4 | 4 |
|  |  |  |  | Focus | 1 | 1 | 1 | 1 | 1 |
|  |  |  |  | Polarity | 7 | 7 | 7 | 7 | 7 |
| 678 | 6453 | 10583997\_148 | Commercial RNA from Saccharomyces cerevisiae (Boehringer Mannheim, Meylan, France) |A4:\*\*1SP3E3| was diluted in phosphate buffer (pH 7.1) and added to the sieved and dried soil samples (2 ml g of soil 1) to final concentrations of 20, 50, and 100 mg of RNA g (dry weight) of soil 1. |A4:\*\*2MP3E3| |A1:\*\*1MP3E3| |A2:\*\*1MP3E3| |A3:\*\*1MP3E3| |A5:\*\*1MP3E3| |  |  |  |  |  |  |
|  |  |  |  | Annotation | 1MP3E3 1MP3E3 | 1MP3E3 1MP3E3 | 1MP3E3 1MP3E3 | 1SP3E3 2MP3E3 | 1MP3E3 1MP3E3 |
|  |  |  |  | Evidence | 4 4 | 4 4 | 4 4 | 4 4 | 4 4 |
|  |  |  |  | Focus | 2 2 | 2 2 | 2 2 | 4 2 | 2 2 |
|  |  |  |  | Polarity | 7 7 | 7 7 | 7 7 | 7 7 | 7 7 |
| 679 | 56 | 12895418\_291 | The Nodal signaling pathway has conserved roles in regulating asymmetry and laterality throughout vertebrates (Hamada et al., 2002; Boorman and Shimeld, 2002). |A1:\*\*1SP3E2| |A2:\*\*1SP3E2| |A3:\*\*1SP3E2| |A4:\*\*1SP3E2| |A5:\*\*1GP3E2| |  |  |  |  |  |  |
|  |  |  |  | Annotation | 1SP3E2 | 1SP3E2 | 1SP3E2 | 1SP3E2 | 1GP3E2 |
|  |  |  |  | Evidence | 3 | 3 | 3 | 3 | 3 |
|  |  |  |  | Focus | 4 | 4 | 4 | 4 | 1 |
|  |  |  |  | Polarity | 7 | 7 | 7 | 7 | 7 |
| 680 | 2129 | 9323141\_159 | AlF4 did not significantly affect velocity or directionality of movement of TCs. |A1:\*\*1SN3E0| |A2:\*\*1SN3E0| |A3:\*\*1SN2E0| |A4:\*\*1SN3E0| |A5:\*\*1GN3E0| |  |  |  |  |  |  |
|  |  |  |  | Annotation | 1SN3E0 | 1SN3E0 | 1SN2E0 | 1SN3E0 | 1GN3E0 |
|  |  |  |  | Evidence | 1 | 1 | 1 | 1 | 1 |
|  |  |  |  | Focus | 4 | 4 | 4 | 4 | 1 |
|  |  |  |  | Polarity | 1 | 1 | 2 | 1 | 1 |
| 681 | 3463 | 11287180\_223 | Frzb-1, for example, coimmunoprecipitates with Wnt-5A, |A5:\*\*1GP3E1| suggesting that it interacts with the Wnt protein, |A5:\*\*2GP2E0| |A1:\*\*1SP3E0| |A2:\*\*1SP3E0| |A3:\*\*1SP2E0| |A4:\*\*1SP2E0| but it does not block Wnt-5A signaling in a Xenopus functional assay for Wnt-mediated axis duplication. |A5:\*\*3SN3E0| |A1:\*\*2SN3E0| |A2:\*\*2SN3E0| |A3:\*\*2SN3E0| |A4:\*\*2SN3E0| |  |  |  |  |  |  |
|  |  |  |  | Annotation | 1SP3E0 1SP3E0 2SN3E0 | 1SP3E0 1SP3E0 2SN3E0 | 1SP2E0 1SP2E0 2SN3E0 | 1SP2E0 1SP2E0 2SN3E0 | 1GP3E1 2GP2E0 3SN3E0 |
|  |  |  |  | Evidence | 1 1 1 | 1 1 1 | 1 1 1 | 1 1 1 | 2 1 1 |
|  |  |  |  | Focus | 4 4 4 | 4 4 4 | 4 4 4 | 4 4 4 | 1 1 4 |
|  |  |  |  | Polarity | 7 7 1 | 7 7 1 | 6 6 1 | 6 6 1 | 7 6 1 |
| 682 | 2630 | 12020667\_1 | In the animal kingdom, species-specific differences with regard to the absorption of intact carotenoids are observed. |A1:\*\*1SP3E0| |A2:\*\*1SP3E1| |A3:\*\*1SP3E0| |A4:\*\*1SP3E3| |A5:\*\*1GP3E1| |  |  |  |  |  |  |
|  |  |  |  | Annotation | 1SP3E0 | 1SP3E1 | 1SP3E0 | 1SP3E3 | 1GP3E1 |
|  |  |  |  | Evidence | 1 | 2 | 1 | 4 | 2 |
|  |  |  |  | Focus | 4 | 4 | 4 | 4 | 1 |
|  |  |  |  | Polarity | 7 | 7 | 7 | 7 | 7 |
| 683 | 452 | 9501077\_32 | Significant local structural changes may occur when Gag is cleaved, |A3:\*\*1SP2E0| |A4:\*\*1SP2E0| |A5:\*\*1SP2E0| particularly given the dramatic global changes that accompany viral maturation. |A3:\*\*2SP3E0| |A1:\*\*1SP1E0| |A2:\*\*1SP2E0| |A4:\*\*2SP3E0| |A5:\*\*2SP3E0| |  |  |  |  |  |  |
|  |  |  |  | Annotation | 1SP1E0 1SP1E0 | 1SP2E0 1SP2E0 | 1SP2E0 2SP3E0 | 1SP2E0 2SP3E0 | 1SP2E0 2SP3E0 |
|  |  |  |  | Evidence | 1 1 | 1 1 | 1 1 | 1 1 | 1 1 |
|  |  |  |  | Focus | 4 4 | 4 4 | 4 4 | 4 4 | 4 4 |
|  |  |  |  | Polarity | 5 5 | 6 6 | 6 7 | 6 7 | 6 7 |
| 684 | 8408 | 12408808\_227 | Heterozygous siblings (F) and talin mutant (G) embryos are stained for the germ cell marker vasa (green; arrows show germ cells; yolk autofluorescence is also green), F-actin (red), and fasciclin 3 (blue). |A1:\*\*1SP3E1| |A2:\*\*1SP3E1| |A3:\*\*1MP3E3| |A4:\*\*1MP3E3| |A5:\*\*1MP3E3| |  |  |  |  |  |  |
|  |  |  |  | Annotation | 1SP3E1 | 1SP3E1 | 1MP3E3 | 1MP3E3 | 1MP3E3 |
|  |  |  |  | Evidence | 2 | 2 | 4 | 4 | 4 |
|  |  |  |  | Focus | 4 | 4 | 2 | 2 | 2 |
|  |  |  |  | Polarity | 7 | 7 | 7 | 7 | 7 |
| 685 | 8452 | 11181754\_138 | In previous studies, we attributed the increase in triglycerides in apoA-II-Tg mice to their functional LCAT deficiency (4) (5) (6). |A3:\*\*1SP3E2+| |A1:\*\*1SP3E2+| |A2:\*\*1SP3E23+| |A4:\*\*1SP3E2+| |A5:\*\*1SP3E2| |  |  |  |  |  |  |
|  |  |  |  | Annotation | 1SP3E2+ | 1SP3E23+ | 1SP3E2+ | 1SP3E2+ | 1SP3E2 |
|  |  |  |  | Evidence | 3 | 3 | 3 | 3 | 3 |
|  |  |  |  | Focus | 4 | 4 | 4 | 4 | 4 |
|  |  |  |  | Polarity | 7 | 7 | 7 | 7 | 7 |
| 686 | 8965 | 12022468\_7 | In this review, we focus on these novel roles of synapsins during the early stages of development. |A1:\*\*1SP3E3| |A2:\*\*1SP3E3| |A3:\*\*1SP3E3| |A4:\*\*1SP3E3| |A5:\*\*1SP3E3| |  |  |  |  |  |  |
|  |  |  |  | Annotation | 1SP3E3 | 1SP3E3 | 1SP3E3 | 1SP3E3 | 1SP3E3 |
|  |  |  |  | Evidence | 4 | 4 | 4 | 4 | 4 |
|  |  |  |  | Focus | 4 | 4 | 4 | 4 | 4 |
|  |  |  |  | Polarity | 7 | 7 | 7 | 7 | 7 |
| 687 | 8828 | 8702594\_96 | Cell extracts of MC58 (L3 immunotype strain phi3) and the lgt B mutant were used in glycosyltransferase assays with FCHASE-aminophenyl-beta-GlcNAc as an acceptor molecule. |A1:\*\*1MP3E3| |A2:\*\*1MP3E3| |A3:\*\*1SP3E3| |A4:\*\*1SP3E3| |A5:\*\*1MP3E3| |  |  |  |  |  |  |
|  |  |  |  | Annotation | 1MP3E3 | 1MP3E3 | 1SP3E3 | 1SP3E3 | 1MP3E3 |
|  |  |  |  | Evidence | 4 | 4 | 4 | 4 | 4 |
|  |  |  |  | Focus | 2 | 2 | 4 | 4 | 2 |
|  |  |  |  | Polarity | 7 | 7 | 7 | 7 | 7 |
| 688 | 6177 | 9729523\_44 | Throughout, the data are expressed as means plus-or-minus standard deviations consistently. |A1:\*\*1SP3E3| |A2:\*\*1SP3E1| |A3:\*\*1GP3E3| |A4:\*\*1GP3E3| |A5:\*\*1GP3E1| |  |  |  |  |  |  |
|  |  |  |  | Annotation | 1SP3E3 | 1SP3E1 | 1GP3E3 | 1GP3E3 | 1GP3E1 |
|  |  |  |  | Evidence | 4 | 2 | 4 | 4 | 2 |
|  |  |  |  | Focus | 4 | 4 | 1 | 1 | 1 |
|  |  |  |  | Polarity | 7 | 7 | 7 | 7 | 7 |
| 689 | 1841 | 9671478\_271 | In the future, the simplicity of the reaction system could facilitate isolation of a synaptic complex by gel shift or identification of contact sites between the RAG proteins and DNA by UV cross-linking. |A1:\*\*1SP2E0| |A2:\*\*1SP1E0| |A3:\*\*1MP2E3| |A4:\*\*1MP2E3| |A5:\*\*1GP3E0| |  |  |  |  |  |  |
|  |  |  |  | Annotation | 1SP2E0 | 1SP1E0 | 1MP2E3 | 1MP2E3 | 1GP3E0 |
|  |  |  |  | Evidence | 1 | 1 | 4 | 4 | 1 |
|  |  |  |  | Focus | 4 | 4 | 2 | 2 | 1 |
|  |  |  |  | Polarity | 6 | 5 | 6 | 6 | 7 |
| 690 | 6811 | 9211885\_40 | A good correlation was observed between growth rate of clones in the presence of the drug and the presence of Leu or Ile in the third position and a hydrophobic amino acid at the C terminus of the peptide. |A1:\*\*1SP3E3| |A2:\*\*1SP3E3| |A3:\*\*1SP3E1| |A4:\*\*1SP3E3| |A5:\*\*1GP3E3| |  |  |  |  |  |  |
|  |  |  |  | Annotation | 1SP3E3 | 1SP3E3 | 1SP3E1 | 1SP3E3 | 1GP3E3 |
|  |  |  |  | Evidence | 4 | 4 | 2 | 4 | 4 |
|  |  |  |  | Focus | 4 | 4 | 4 | 4 | 1 |
|  |  |  |  | Polarity | 7 | 7 | 7 | 7 | 7 |
| 691 | 401 | 12068020\_27 | The thrombin-thrombomodulin interaction can therefore be used as an instructive model system for illustrating the role of hot spots in protein-protein interactions |A1:\*\*1SP1E0| and to dissect the structural origin of residue-residue coupling in epitopes involved in protein recognition. |A1:\*\*2SP1E0| |A2:\*\*1SP2E0| |A3:\*\*1SP3E0| |A4:\*\*1SP3E0| |A5:\*\*1SP3E1| |  |  |  |  |  |  |
|  |  |  |  | Annotation | 1SP1E0 2SP1E0 | 1SP2E0 1SP2E0 | 1SP3E0 1SP3E0 | 1SP3E0 1SP3E0 | 1SP3E1 1SP3E1 |
|  |  |  |  | Evidence | 1 1 | 1 1 | 1 1 | 1 1 | 2 2 |
|  |  |  |  | Focus | 4 4 | 4 4 | 4 4 | 4 4 | 4 4 |
|  |  |  |  | Polarity | 5 5 | 6 6 | 7 7 | 7 7 | 7 7 |
| 692 | 1289 | 10555146\_268 | These data provide novel evidence for the intricate regulation of VE-cadherin function by the extracellular SPP receptors EDG-1 and EDG-3. |A1:\*\*1SP3E0| |A2:\*\*1SP3E1| |A3:\*\*1SP3E3| |A4:\*\*1SP3E3| |A5:\*\*1SP3E1| |  |  |  |  |  |  |
|  |  |  |  | Annotation | 1SP3E0 | 1SP3E1 | 1SP3E3 | 1SP3E3 | 1SP3E1 |
|  |  |  |  | Evidence | 1 | 2 | 4 | 4 | 2 |
|  |  |  |  | Focus | 4 | 4 | 4 | 4 | 4 |
|  |  |  |  | Polarity | 7 | 7 | 7 | 7 | 7 |
| 693 | 9184 | 12667443\_350 | Thus, tumor-derived mutations might represent double-hits by concomitantly abrogating the transcriptional and mitochondrial apoptotic activity of p53. |A1:\*\*1SP1E0| |A2:\*\*1SP1E0| |A3:\*\*1SP1E0-| |A4:\*\*1SP2E0| |A5:\*\*1SP2E1| |  |  |  |  |  |  |
|  |  |  |  | Annotation | 1SP1E0 | 1SP1E0 | 1SP1E0- | 1SP2E0 | 1SP2E1 |
|  |  |  |  | Evidence | 1 | 1 | 1 | 1 | 2 |
|  |  |  |  | Focus | 4 | 4 | 4 | 4 | 4 |
|  |  |  |  | Polarity | 5 | 5 | 5 | 6 | 6 |
| 694 | 7150 | 9927430\_207 | Measurements were made using a 1 mm pathlength cell and data analyzed using the JASCO software. |A1:\*\*1GP3E3| |A2:\*\*1GP3E3| |A3:\*\*1SP3E3| |A4:\*\*1SP3E3| |A5:\*\*1MP3E3| |  |  |  |  |  |  |
|  |  |  |  | Annotation | 1GP3E3 | 1GP3E3 | 1SP3E3 | 1SP3E3 | 1MP3E3 |
|  |  |  |  | Evidence | 4 | 4 | 4 | 4 | 4 |
|  |  |  |  | Focus | 1 | 1 | 4 | 4 | 2 |
|  |  |  |  | Polarity | 7 | 7 | 7 | 7 | 7 |
| 695 | 7160 | 9560393\_131 | Three of the 54 revertants isolated at 30 degrees, and both revertants isolated at 36 degrees, contained dominant suppressors. |A1:\*\*1SP3E3| |A2:\*\*1SP3E3| |A3:\*\*1SP3E3| |A4:\*\*1SP3E3| |A5:\*\*1MGP3E3| |  |  |  |  |  |  |
|  |  |  |  | Annotation | 1SP3E3 | 1SP3E3 | 1SP3E3 | 1SP3E3 | 1MGP3E3 |
|  |  |  |  | Evidence | 4 | 4 | 4 | 4 | 4 |
|  |  |  |  | Focus | 4 | 4 | 4 | 4 | 3 |
|  |  |  |  | Polarity | 7 | 7 | 7 | 7 | 7 |
| 696 | 2634 | 12045187\_188 | Consistent with our results, |A5:\*\*1GP3E3| nuclear PML structures are essential for TNFalpha-mediated death (Wang et al., 1998), |A1:\*\*1SP3E2| |A2:\*\*1SP3E23| and PML has been linked to both caspase-dependent and -independent death pathways ( Wang et al. , 1998; Quignon et al. , 1998). |A1:\*\*2SP3E2| |A2:\*\*2SP3E2| |A3:\*\*1SP3E2| |A4:\*\*1SP3E2| |A5:\*\*2SP3E2| |  |  |  |  |  |  |
|  |  |  |  | Annotation | 1SP3E2 1SP3E2 2SP3E2 | 1SP3E23 1SP3E23 2SP3E2 | 1SP3E2 1SP3E2 1SP3E2 | 1SP3E2 1SP3E2 1SP3E2 | 1GP3E3 2SP3E2 2SP3E2 |
|  |  |  |  | Evidence | 3 3 3 | 3 3 3 | 3 3 3 | 3 3 3 | 4 3 3 |
|  |  |  |  | Focus | 4 4 4 | 4 4 4 | 4 4 4 | 4 4 4 | 1 4 4 |
|  |  |  |  | Polarity | 7 7 7 | 7 7 7 | 7 7 7 | 7 7 7 | 7 7 7 |
| 697 | 5832 | 11136978\_100 | Dynamic studies of PI3K and N-Ras indicate both rapid association and dissociation of the two proteins (Figure 2B). |A1:\*\*1SP3E3| |A2:\*\*1SP3E3| |A3:\*\*1SP3E3| |A4:\*\*1SP3E3| |A5:\*\*1SP3E3| |  |  |  |  |  |  |
|  |  |  |  | Annotation | 1SP3E3 | 1SP3E3 | 1SP3E3 | 1SP3E3 | 1SP3E3 |
|  |  |  |  | Evidence | 4 | 4 | 4 | 4 | 4 |
|  |  |  |  | Focus | 4 | 4 | 4 | 4 | 4 |
|  |  |  |  | Polarity | 7 | 7 | 7 | 7 | 7 |
| 698 | 4658 | 9539423\_136 | UT6-b detects sequences that were present in most of the chromosomes in the two U. maydis strains tested. |A1:\*\*1SP3E3| |A2:\*\*1SP3E3| |A3:\*\*1SP2E3| |A4:\*\*1SP3E3| |A5:\*\*1GP3E1| |  |  |  |  |  |  |
|  |  |  |  | Annotation | 1SP3E3 | 1SP3E3 | 1SP2E3 | 1SP3E3 | 1GP3E1 |
|  |  |  |  | Evidence | 4 | 4 | 4 | 4 | 2 |
|  |  |  |  | Focus | 4 | 4 | 4 | 4 | 1 |
|  |  |  |  | Polarity | 7 | 7 | 6 | 7 | 7 |
| 699 | 5684 | 10788451\_67 | Ca2+ was washed off between the two applications, and 200 muM taurolithocholate was perfused for 1 min. |A1:\*\*1SP3E3| |A2:\*\*1SP3E3| |A3:\*\*1SP3E3| |A4:\*\*1SP3E3| |A5:\*\*1MP3E3| |  |  |  |  |  |  |
|  |  |  |  | Annotation | 1SP3E3 | 1SP3E3 | 1SP3E3 | 1SP3E3 | 1MP3E3 |
|  |  |  |  | Evidence | 4 | 4 | 4 | 4 | 4 |
|  |  |  |  | Focus | 4 | 4 | 4 | 4 | 2 |
|  |  |  |  | Polarity | 7 | 7 | 7 | 7 | 7 |
| 700 | 130 | 9315549\_11 | In-hospital mortality rates declined during the period from 1993 through 1995 and were lower than predicted |A5:\*\*1GP3E0-| despite the increase in risk. |A5:\*\*2GP3E0+| |A1:\*\*1GP3E3-| |A2:\*\*1GP2E3-| |A3:\*\*1SP3E0-| |A4:\*\*1GP3E0-| |  |  |  |  |  |  |
|  |  |  |  | Annotation | 1GP3E3- 1GP3E3- | 1GP2E3- 1GP2E3- | 1SP3E0- 1SP3E0- | 1GP3E0- 1GP3E0- | 1GP3E0- 2GP3E0+ |
|  |  |  |  | Evidence | 4 4 | 4 4 | 1 1 | 1 1 | 1 1 |
|  |  |  |  | Focus | 1 1 | 1 1 | 4 4 | 1 1 | 1 1 |
|  |  |  |  | Polarity | 7 7 | 6 6 | 7 7 | 7 7 | 7 7 |
| 701 | 7790 | 12051669\_2 | Their model implied that the weighted average of the chemical shifts of the beta-P (MgADP) for S1-bound MgADP asymptotically approaches a high temperature limit. |A1:\*\*1SP3E1| |A2:\*\*1SP3E1+| |A3:\*\*1SP3E3+| |A4:\*\*1SP3E3+| |A5:\*\*1SP3E1| |  |  |  |  |  |  |
|  |  |  |  | Annotation | 1SP3E1 | 1SP3E1+ | 1SP3E3+ | 1SP3E3+ | 1SP3E1 |
|  |  |  |  | Evidence | 2 | 2 | 4 | 4 | 2 |
|  |  |  |  | Focus | 4 | 4 | 4 | 4 | 4 |
|  |  |  |  | Polarity | 7 | 7 | 7 | 7 | 7 |
| 702 | 9018 | 0010224003\_59 | The filters were rinsed once with 5 ml of the corresponding growth medium, which was maintained at 30 degrees C. |A1:\*\*1MP3E3| |A2:\*\*1MP3E3| |A3:\*\*1MP3E3| |A4:\*\*1MP3E3| |A5:\*\*1MP3E3| |  |  |  |  |  |  |
|  |  |  |  | Annotation | 1MP3E3 | 1MP3E3 | 1MP3E3 | 1MP3E3 | 1MP3E3 |
|  |  |  |  | Evidence | 4 | 4 | 4 | 4 | 4 |
|  |  |  |  | Focus | 2 | 2 | 2 | 2 | 2 |
|  |  |  |  | Polarity | 7 | 7 | 7 | 7 | 7 |
| 703 | 8015 | 9679052\_34 | It is hypothesized that part of the signal transduction pathway involves the stimulation of stretch-activated ion channels/carriers that are activated by the gravity stimulus (Sievers et al. 1991). |A1:\*\*1SP2E2| |A2:\*\*1SP2E2| |A3:\*\*1SP2E2+| |A4:\*\*1SP2E2+| |A5:\*\*1SP2E2| |  |  |  |  |  |  |
|  |  |  |  | Annotation | 1SP2E2 | 1SP2E2 | 1SP2E2+ | 1SP2E2+ | 1SP2E2 |
|  |  |  |  | Evidence | 3 | 3 | 3 | 3 | 3 |
|  |  |  |  | Focus | 4 | 4 | 4 | 4 | 4 |
|  |  |  |  | Polarity | 6 | 6 | 6 | 6 | 6 |
| 704 | 5947 | 11904424\_26 | In epithelial and endothelial cells, integrin ligation regulates cell survival such that detachment from the ECM rapidly induces apoptosis (17, 18). |A1:\*\*1SP3E2| |A2:\*\*1SP3E2+| |A3:\*\*1SP3E2| |A4:\*\*1SP3E2| |A5:\*\*1SP3E2+| |  |  |  |  |  |  |
|  |  |  |  | Annotation | 1SP3E2 | 1SP3E2+ | 1SP3E2 | 1SP3E2 | 1SP3E2+ |
|  |  |  |  | Evidence | 3 | 3 | 3 | 3 | 3 |
|  |  |  |  | Focus | 4 | 4 | 4 | 4 | 4 |
|  |  |  |  | Polarity | 7 | 7 | 7 | 7 | 7 |
| 705 | 6852 | 9539738\_26 | The E2 protein is phosphorylated in its central hinge region |A1:\*\*1SP3E3| |A2:\*\*1SP2E0| and previous studies showed that |A3:\*\*1SP3E2| |A4:\*\*1SP3E2| at least one of the phosphorylation sites was critical for transformation functions (ref. 11 and Fig. 1). |A1:\*\*2SP3E23| |A2:\*\*2SP3E23| |A3:\*\*2SP3E3| |A4:\*\*2SP3E3| |A5:\*\*1SP3E2| |  |  |  |  |  |  |
|  |  |  |  | Annotation | 1SP3E3 2SP3E23 2SP3E23 | 1SP2E0 2SP3E23 2SP3E23 | 1SP3E2 1SP3E2 2SP3E3 | 1SP3E2 1SP3E2 2SP3E3 | 1SP3E2 1SP3E2 1SP3E2 |
|  |  |  |  | Evidence | 4 3 3 | 1 3 3 | 3 3 4 | 3 3 4 | 3 3 3 |
|  |  |  |  | Focus | 4 4 4 | 4 4 4 | 4 4 4 | 4 4 4 | 4 4 4 |
|  |  |  |  | Polarity | 7 7 7 | 6 7 7 | 7 7 7 | 7 7 7 | 7 7 7 |
| 706 | 5174 | 9649502\_256 | p53A cells were transfected transiently with 10 mug of pCEP4-myc-LA(1-406) by electroporation |A3:\*\*1MP3E3| |A4:\*\*1MP3E3| to detect transient expression of a lamin fragment as a control protein (Rao et al. 1996). |A3:\*\*2SP3E2| |A1:\*\*1SP3E23| |A2:\*\*1MP3E23| |A4:\*\*2SP3E2| |A5:\*\*1MP3E2| |  |  |  |  |  |  |
|  |  |  |  | Annotation | 1SP3E23 1SP3E23 | 1MP3E23 1MP3E23 | 1MP3E3 2SP3E2 | 1MP3E3 2SP3E2 | 1MP3E2 1MP3E2 |
|  |  |  |  | Evidence | 3 3 | 3 3 | 4 3 | 4 3 | 3 3 |
|  |  |  |  | Focus | 4 4 | 2 2 | 2 4 | 2 4 | 2 2 |
|  |  |  |  | Polarity | 7 7 | 7 7 | 7 7 | 7 7 | 7 7 |
| 707 | 1491 | 9539806\_93 | Likewise, juvenile sensory neurons had a mean action potential duration (1.48 msec +/ 0.1), |A5:\*\*1SP3E3| which was not significantly different from the duration of the adult action potential (mean = 1.44 +/ 0.2). |A5:\*\*2SN3E3| |A1:\*\*1SP3E3| |A2:\*\*1SP3E3| |A3:\*\*1SP3E3| |A4:\*\*1SP3E3| |  |  |  |  |  |  |
|  |  |  |  | Annotation | 1SP3E3 1SP3E3 | 1SP3E3 1SP3E3 | 1SP3E3 1SP3E3 | 1SP3E3 1SP3E3 | 1SP3E3 2SN3E3 |
|  |  |  |  | Evidence | 4 4 | 4 4 | 4 4 | 4 4 | 4 4 |
|  |  |  |  | Focus | 4 4 | 4 4 | 4 4 | 4 4 | 4 4 |
|  |  |  |  | Polarity | 7 7 | 7 7 | 7 7 | 7 7 | 7 1 |
| 708 | 9386 | 11438668\_397 | Modulation of Wnt signaling by Axin and Axil. |A1:\*\*1SP2E0| |A2:\*\*1SP2E0| |A3:\*\*1SP3E0| |A4:\*\*1SP3E0| |A5:\*\*1SP3E0| |  |  |  |  |  |  |
|  |  |  |  | Annotation | 1SP2E0 | 1SP2E0 | 1SP3E0 | 1SP3E0 | 1SP3E0 |
|  |  |  |  | Evidence | 1 | 1 | 1 | 1 | 1 |
|  |  |  |  | Focus | 4 | 4 | 4 | 4 | 4 |
|  |  |  |  | Polarity | 6 | 6 | 7 | 7 | 7 |
| 709 | 4743 | 10557338\_14 | The efficacy of these drugs in treating positive symptoms of schizophrenia (such as hallucinations and delusions) is highly correlated with their affinity for postsynaptic dopamine (D2) receptors (1), |A3:\*\*1SP3E3| |A4:\*\*1SP3E3| |A5:\*\*1SP3E2| suggesting that psychotic symptoms might be caused by excessive dopaminergic effects or hyperdopaminergia (2). |A3:\*\*2SP2E3| |A1:\*\*1SP1E2| |A2:\*\*1SP3E2| |A4:\*\*2SP2E3| |A5:\*\*2SP2E2| |  |  |  |  |  |  |
|  |  |  |  | Annotation | 1SP1E2 1SP1E2 | 1SP3E2 1SP3E2 | 1SP3E3 2SP2E3 | 1SP3E3 2SP2E3 | 1SP3E2 2SP2E2 |
|  |  |  |  | Evidence | 3 3 | 3 3 | 4 4 | 4 4 | 3 3 |
|  |  |  |  | Focus | 4 4 | 4 4 | 4 4 | 4 4 | 4 4 |
|  |  |  |  | Polarity | 5 5 | 7 7 | 7 6 | 7 6 | 7 6 |
| 710 | 48 | 9390512\_19 | Second, twin studies comparing the concordance for schizophrenia in monozygotic (MZ) twin pairs (who, in principle, share all of their genes) with the concordance in dizygotic (DZ) twin pairs (who share half of their genes) |A5:\*\*1SP3E3| demonstrated that the MZ concordance rate (46%-48%) is significantly higher than the DZ rate (4%-14%). |A5:\*\*2SP3E3+| |A1:\*\*1GP3E1| |A2:\*\*1SP3E3| |A3:\*\*1SP3E3| |A4:\*\*1SP3E3| |  |  |  |  |  |  |
|  |  |  |  | Annotation | 1GP3E1 1GP3E1 | 1SP3E3 1SP3E3 | 1SP3E3 1SP3E3 | 1SP3E3 1SP3E3 | 1SP3E3 2SP3E3+ |
|  |  |  |  | Evidence | 2 2 | 4 4 | 4 4 | 4 4 | 4 4 |
|  |  |  |  | Focus | 1 1 | 4 4 | 4 4 | 4 4 | 4 4 |
|  |  |  |  | Polarity | 7 7 | 7 7 | 7 7 | 7 7 | 7 7 |
| 711 | 1213 | 11238224\_132 | In contrast to RNA studies, antimicrobial peptides were also found in phagocytes (i.e., alveolar macrophages) and lymphocytes. |A1:\*\*1SP3E3| |A2:\*\*1SP3E3| |A3:\*\*1SP3E0| |A4:\*\*1SP3E0| |A5:\*\*1SP3E1| |  |  |  |  |  |  |
|  |  |  |  | Annotation | 1SP3E3 | 1SP3E3 | 1SP3E0 | 1SP3E0 | 1SP3E1 |
|  |  |  |  | Evidence | 4 | 4 | 1 | 1 | 2 |
|  |  |  |  | Focus | 4 | 4 | 4 | 4 | 4 |
|  |  |  |  | Polarity | 7 | 7 | 7 | 7 | 7 |
| 712 | 9344 | 8706137\_179 | Multimer formation is catalyzed by the DNA of the region that bears two phased binding sites. |A1:\*\*1SP3E3| |A2:\*\*1SP3E1| |A3:\*\*1SP3E0| |A4:\*\*1SP3E0| |A5:\*\*1SP3E0| |  |  |  |  |  |  |
|  |  |  |  | Annotation | 1SP3E3 | 1SP3E1 | 1SP3E0 | 1SP3E0 | 1SP3E0 |
|  |  |  |  | Evidence | 4 | 2 | 1 | 1 | 1 |
|  |  |  |  | Focus | 4 | 4 | 4 | 4 | 4 |
|  |  |  |  | Polarity | 7 | 7 | 7 | 7 | 7 |
| 713 | 7548 | 11010904\_168 | This result emphasizes further the importance of optimizing production of SAT and cysteine desulfhydrase. |A1:\*\*1SP3E0| |A2:\*\*1SP3E0| |A3:\*\*1SP3E3| |A4:\*\*1SP3E3| |A5:\*\*1SP3E3| |  |  |  |  |  |  |
|  |  |  |  | Annotation | 1SP3E0 | 1SP3E0 | 1SP3E3 | 1SP3E3 | 1SP3E3 |
|  |  |  |  | Evidence | 1 | 1 | 4 | 4 | 4 |
|  |  |  |  | Focus | 4 | 4 | 4 | 4 | 4 |
|  |  |  |  | Polarity | 7 | 7 | 7 | 7 | 7 |
| 714 | 8950 | 10787438\_182 | Transfer of stearate caused a fast decline of intrinsic fluorescence of BSA |A2:\*\*1SP3E1-| |A3:\*\*1SP3E0-| |A4:\*\*1SP3E0-| |A5:\*\*1SP3E3-| and reached, in contrast to heptafluorostearate, the final equilibrium within 3 min (Fig 8A). |A2:\*\*2SP3E3| |A1:\*\*1MP3E3| |A3:\*\*2SP3E3| |A4:\*\*2SP3E3| |A5:\*\*2SP3E3| |  |  |  |  |  |  |
|  |  |  |  | Annotation | 1MP3E3 1MP3E3 | 1SP3E1- 2SP3E3 | 1SP3E0- 2SP3E3 | 1SP3E0- 2SP3E3 | 1SP3E3- 2SP3E3 |
|  |  |  |  | Evidence | 4 4 | 2 4 | 1 4 | 1 4 | 4 4 |
|  |  |  |  | Focus | 2 2 | 4 4 | 4 4 | 4 4 | 4 4 |
|  |  |  |  | Polarity | 7 7 | 7 7 | 7 7 | 7 7 | 7 7 |
| 715 | 1776 | 11086011\_253 | When Ran-GTP levels were lowered in these extracts, spindle assembly was blocked, |A1:\*\*1SN3E3| |A2:\*\*1SP3E3-| |A5:\*\*1SP3E3-| whereas high Ran-GTP levels promoted formation of spindle structures. |A1:\*\*2SP3E3| |A2:\*\*2SP3E3| |A3:\*\*1SP3E0| |A4:\*\*1SP3E0| |A5:\*\*2SP3E3+| |  |  |  |  |  |  |
|  |  |  |  | Annotation | 1SN3E3 2SP3E3 | 1SP3E3- 2SP3E3 | 1SP3E0 1SP3E0 | 1SP3E0 1SP3E0 | 1SP3E3- 2SP3E3+ |
|  |  |  |  | Evidence | 4 4 | 4 4 | 1 1 | 1 1 | 4 4 |
|  |  |  |  | Focus | 4 4 | 4 4 | 4 4 | 4 4 | 4 4 |
|  |  |  |  | Polarity | 1 7 | 7 7 | 7 7 | 7 7 | 7 7 |
| 716 | 1928 | 9744866\_70 | While previous studies correlated induced p18 expression and association with CDK4 and CDK6 during in vitro myogenesis and adipogenesis (Franklin and Xiong 1996 ; Phelps and Xiong 1998 ), |A1:\*\*1SP3E2| |A2:\*\*1SP3E2| |A3:\*\*1SP3E2+| |A4:\*\*1SP3E2+| |A5:\*\*1SP3E2| we did not detect abnormal muscle or adipose development in p18-null animals, |A3:\*\*2SN3E3| |A4:\*\*2SN3E3| This indicates that p18 does not have an essential role in causing cell cycle withdrawal during the differentiation of these two tissues. |A1:\*\*2SN3E3| |A2:\*\*2SN3E3| |A3:\*\*3SN3E0| |A4:\*\*3SN3E0| |A5:\*\*2SN3E3| |  |  |  |  |  |  |
|  |  |  |  | Annotation | 1SP3E2 2SN3E3 2SN3E3 | 1SP3E2 2SN3E3 2SN3E3 | 1SP3E2+ 2SN3E3 3SN3E0 | 1SP3E2+ 2SN3E3 3SN3E0 | 1SP3E2 2SN3E3 2SN3E3 |
|  |  |  |  | Evidence | 3 4 4 | 3 4 4 | 3 4 1 | 3 4 1 | 3 4 4 |
|  |  |  |  | Focus | 4 4 4 | 4 4 4 | 4 4 4 | 4 4 4 | 4 4 4 |
|  |  |  |  | Polarity | 7 1 1 | 7 1 1 | 7 1 1 | 7 1 1 | 7 1 1 |
| 717 | 7605 | 12464613\_1251 | Because 12(S) HETE 12(S)HETE had no effect on PI3K signaling (Fig. 4), |A2:\*\*1SP3E3-| |A3:\*\*1SN3E3| |A4:\*\*1SN3E3| |A5:\*\*1SN3E3| these results imply that the synergistic effect of EGF and 12(S) HETE 12( S)HETE on PKCbeta activation requires EGF-dependent signaling through PI3K. |A2:\*\*2SP3E0| |A1:\*\*1SP3E3| |A3:\*\*2SP3E3| |A4:\*\*2SP3E3| |A5:\*\*2SP3E3| |  |  |  |  |  |  |
|  |  |  |  | Annotation | 1SP3E3 1SP3E3 | 1SP3E3- 2SP3E0 | 1SN3E3 2SP3E3 | 1SN3E3 2SP3E3 | 1SN3E3 2SP3E3 |
|  |  |  |  | Evidence | 4 4 | 4 1 | 4 4 | 4 4 | 4 4 |
|  |  |  |  | Focus | 4 4 | 4 4 | 4 4 | 4 4 | 4 4 |
|  |  |  |  | Polarity | 7 7 | 7 7 | 1 7 | 1 7 | 1 7 |
| 718 | 4939 | 10567577\_150 | In control studies, levels of each of the SCAN domain containing GAL4 fusions were shown to be comparable, and the ability of each of the fusions to bind a GAL4 site was similar (data not shown). |A1:\*\*1SP3E1| |A2:\*\*1SP3E1| |A3:\*\*1SP3E3| |A4:\*\*1SP3E3| |A5:\*\*1SP3E3| |  |  |  |  |  |  |
|  |  |  |  | Annotation | 1SP3E1 | 1SP3E1 | 1SP3E3 | 1SP3E3 | 1SP3E3 |
|  |  |  |  | Evidence | 2 | 2 | 4 | 4 | 4 |
|  |  |  |  | Focus | 4 | 4 | 4 | 4 | 4 |
|  |  |  |  | Polarity | 7 | 7 | 7 | 7 | 7 |
| 719 | 4846 | 9281602\_169 | Thus, the ligand binding domain (26, 27) and the intracellular domains of the receptor that control the specificity of linkage to G proteins (28, 29) of mGluRs |A3:\*\*1SP3E2| |A4:\*\*1SP3E2| have little in common with other G protein-coupled receptors. |A3:\*\*2SP3E0| |A1:\*\*1SP3E2| |A2:\*\*1SP3E2| |A4:\*\*2SP3E0| |A5:\*\*1SP3E2| |  |  |  |  |  |  |
|  |  |  |  | Annotation | 1SP3E2 1SP3E2 | 1SP3E2 1SP3E2 | 1SP3E2 2SP3E0 | 1SP3E2 2SP3E0 | 1SP3E2 1SP3E2 |
|  |  |  |  | Evidence | 3 3 | 3 3 | 3 1 | 3 1 | 3 3 |
|  |  |  |  | Focus | 4 4 | 4 4 | 4 4 | 4 4 | 4 4 |
|  |  |  |  | Polarity | 7 7 | 7 7 | 7 7 | 7 7 | 7 7 |
| 720 | 6991 | 12208670\_8 | CRH did not have any effect on oestrogen release stimulated by increasing concentrations of IGF-I |A1:\*\*1SN3E3+| |A2:\*\*1SN3E3+| |A3:\*\*1SN3E0+| |A4:\*\*1SN3E0+| |A5:\*\*1SN3E3+| and its suppressive effect on FSH-stimulated oestrogen release was overcome by the addition of low doses of exogenous IGF-I. |A1:\*\*2SP3E3| |A2:\*\*2SP3E3| |A3:\*\*2SP3E0| |A4:\*\*2SP3E0| |A5:\*\*2SP3E3| |  |  |  |  |  |  |
|  |  |  |  | Annotation | 1SN3E3+ 2SP3E3 | 1SN3E3+ 2SP3E3 | 1SN3E0+ 2SP3E0 | 1SN3E0+ 2SP3E0 | 1SN3E3+ 2SP3E3 |
|  |  |  |  | Evidence | 4 4 | 4 4 | 1 1 | 1 1 | 4 4 |
|  |  |  |  | Focus | 4 4 | 4 4 | 4 4 | 4 4 | 4 4 |
|  |  |  |  | Polarity | 1 7 | 1 7 | 1 7 | 1 7 | 1 7 |
| 721 | 3244 | 10506154\_25 | Tel.: 615-322-4384; Fax: 615-343-3794; E-mail: jackie.corbin@mcmail.vanderbilt.edu. |A1:\*\*1GP3E0| |A2:\*\*1GP3E3| |A3:\*\*1GP3E0| |A4:\*\*1GP3E0| |A5:\*\*1GP3E0| |  |  |  |  |  |  |
|  |  |  |  | Annotation | 1GP3E0 | 1GP3E3 | 1GP3E0 | 1GP3E0 | 1GP3E0 |
|  |  |  |  | Evidence | 1 | 4 | 1 | 1 | 1 |
|  |  |  |  | Focus | 1 | 1 | 1 | 1 | 1 |
|  |  |  |  | Polarity | 7 | 7 | 7 | 7 | 7 |
| 722 | 2881 | 9362061\_133 | Xyloside was added to the cells during the last 30 min to label newly synthesized [35]GAG chains. |A1:\*\*1SP3E23| |A2:\*\*1SP3E23| |A3:\*\*1MP3E3| |A4:\*\*1MP3E3| |A5:\*\*1MP3E3| |  |  |  |  |  |  |
|  |  |  |  | Annotation | 1SP3E23 | 1SP3E23 | 1MP3E3 | 1MP3E3 | 1MP3E3 |
|  |  |  |  | Evidence | 3 | 3 | 4 | 4 | 4 |
|  |  |  |  | Focus | 4 | 4 | 2 | 2 | 2 |
|  |  |  |  | Polarity | 7 | 7 | 7 | 7 | 7 |
| 723 | 4369 | 11546819\_85 | At varying times, hemolysis was determined by pelleting intact erythrocytes and measuring the release of hemoglobin as described under "Experimental Procedures." |A1:\*\*1MP3E3| |A2:\*\*1MP3E3| In an attempt to determine whether the hemolytic potential of bound SLS could be reversed by protease treatment, |A1:\*\*2SP2E3| SLS was bound to sheep erythrocytes at 17 degrees C for 15 min. |A1:\*\*3SP3E3| |A2:\*\*2SP2E3| |A3:\*\*1MP3E3| |A4:\*\*1MP3E3| |A5:\*\*1MP3E3| |  |  |  |  |  |  |
|  |  |  |  | Annotation | 1MP3E3 2SP2E3 3SP3E3 | 1MP3E3 2SP2E3 2SP2E3 | 1MP3E3 1MP3E3 1MP3E3 | 1MP3E3 1MP3E3 1MP3E3 | 1MP3E3 1MP3E3 1MP3E3 |
|  |  |  |  | Evidence | 4 4 4 | 4 4 4 | 4 4 4 | 4 4 4 | 4 4 4 |
|  |  |  |  | Focus | 2 4 4 | 2 4 4 | 2 2 2 | 2 2 2 | 2 2 2 |
|  |  |  |  | Polarity | 7 6 7 | 7 6 6 | 7 7 7 | 7 7 7 | 7 7 7 |
| 724 | 1331 | 10611236\_68 | As shown in Fig. 1, the induction of c- jun and the collagenase gene was very efficient in wild-type cells. |A1:\*\*1SP3E3| |A2:\*\*1SP3E3| |A3:\*\*1SP3E3+| |A4:\*\*1SP3E3+| |A5:\*\*1SP3E3| |  |  |  |  |  |  |
|  |  |  |  | Annotation | 1SP3E3 | 1SP3E3 | 1SP3E3+ | 1SP3E3+ | 1SP3E3 |
|  |  |  |  | Evidence | 4 | 4 | 4 | 4 | 4 |
|  |  |  |  | Focus | 4 | 4 | 4 | 4 | 4 |
|  |  |  |  | Polarity | 7 | 7 | 7 | 7 | 7 |
| 725 | 2887 | 10455129\_5 | Moreover, in such cultures, insulin ( ), GH ( ), glucocorticoids ( ), and amino acid availability ( ) have been widely reported to regulate IGF-I gene expression. |A1:\*\*1SP3E2| |A2:\*\*1SP3E2| |A3:\*\*1MP3E3| |A4:\*\*1MP3E3| |A5:\*\*1SP3E1| |  |  |  |  |  |  |
|  |  |  |  | Annotation | 1SP3E2 | 1SP3E2 | 1MP3E3 | 1MP3E3 | 1SP3E1 |
|  |  |  |  | Evidence | 3 | 3 | 4 | 4 | 2 |
|  |  |  |  | Focus | 4 | 4 | 2 | 2 | 4 |
|  |  |  |  | Polarity | 7 | 7 | 7 | 7 | 7 |
| 726 | 742 | 11160081\_59 | The resulting PCR products were individually cloned into pGEM7 using XhoI and NsiI to yield plasmids pEA4 and pVALGOX, respectively. |A1:\*\*1SP3E3| |A2:\*\*1MP3E3| |A3:\*\*1SP3E3| |A4:\*\*1SP3E3| |A5:\*\*1MP3E3| |  |  |  |  |  |  |
|  |  |  |  | Annotation | 1SP3E3 | 1MP3E3 | 1SP3E3 | 1SP3E3 | 1MP3E3 |
|  |  |  |  | Evidence | 4 | 4 | 4 | 4 | 4 |
|  |  |  |  | Focus | 4 | 2 | 4 | 4 | 2 |
|  |  |  |  | Polarity | 7 | 7 | 7 | 7 | 7 |
| 727 | 7661 | 10751396\_68 | These results suggest that the chromatin organization of both nuc B and A in these cells was not dependent on the GR, |A3:\*\*1SP3E3| |A4:\*\*1SP3E3| |A5:\*\*1SN2E3| since cells not expressing the GR (M10) exhibited enzyme cleavage similar to those cells expressing the GR (GR2). |A3:\*\*2SN3E0| |A1:\*\*1SN3E3| |A4:\*\*2SN3E0| |A5:\*\*2SN3E3| View larger version (27K): [in this window] [in a new window] Fig. 5. Nuc B and nuc A exhibit differential restriction endonuclease hypersensitivity. |A3:\*\*3SP3E3| |A1:\*\*2SP3E3| |A2:\*\*1SP3E3| |A4:\*\*3SP3E3| |A5:\*\*3SP3E3| |  |  |  |  |  |  |
|  |  |  |  | Annotation | 1SN3E3 1SN3E3 2SP3E3 | 1SP3E3 1SP3E3 1SP3E3 | 1SP3E3 2SN3E0 3SP3E3 | 1SP3E3 2SN3E0 3SP3E3 | 1SN2E3 2SN3E3 3SP3E3 |
|  |  |  |  | Evidence | 4 4 4 | 4 4 4 | 4 1 4 | 4 1 4 | 4 4 4 |
|  |  |  |  | Focus | 4 4 4 | 4 4 4 | 4 4 4 | 4 4 4 | 4 4 4 |
|  |  |  |  | Polarity | 1 1 7 | 7 7 7 | 7 1 7 | 7 1 7 | 2 1 7 |
| 728 | 2060 | 9162023\_2 | A good correlation between aromatic amino acid at this position and MAO A-type substrate specificity was seen in trout MAO ( ), |A2:\*\*1SP3E2| |A3:\*\*1SP3E0| |A4:\*\*1SP3E0| which has Phe at this position and properties in substrate specificity and inhibitor sensitivity more like those of mammalian MAO A, |A3:\*\*2SP3E0-| |A4:\*\*2SP3E0-| although it does share a similar extent of homology (about 70%) with both mammalian MAO A and MAO B. B. |A5:\*\*1SP3E2| MAO A has a similar affinity for most substrates with aromatic rings, yet over a 1000-fold difference in the affinity among substrates was observed for MAO B. B. |A1:\*\*1SP3E23| |A3:\*\*3SN3E3| |A4:\*\*3SN3E3| |A5:\*\*2SP3E1| The finding of participation of the aromatic side chain in substrate recognition of MAO A suggests that pi-pi interaction between aromatic rings of substrates and the enzyme plays a major part in their interaction and could explain why MAO A has a similar affinity for aromatic substrates substrates. |A1:\*\*2SP2E0| |A2:\*\*2SP2E3| |A3:\*\*4SP2E3| |A4:\*\*4SP2E3| |A5:\*\*3SP2E3| |  |  |  |  |  |  |
|  |  |  |  | Annotation | 1SP3E23 1SP3E23 1SP3E23 1SP3E23 2SP2E0 | 1SP3E2 2SP2E3 2SP2E3 2SP2E3 2SP2E3 | 1SP3E0 2SP3E0- 3SN3E3 3SN3E3 4SP2E3 | 1SP3E0 2SP3E0- 3SN3E3 3SN3E3 4SP2E3 | 1SP3E2 1SP3E2 1SP3E2 2SP3E1 3SP2E3 |
|  |  |  |  | Evidence | 3 3 3 3 1 | 3 4 4 4 4 | 1 1 4 4 4 | 1 1 4 4 4 | 3 3 3 2 4 |
|  |  |  |  | Focus | 4 4 4 4 4 | 4 4 4 4 4 | 4 4 4 4 4 | 4 4 4 4 4 | 4 4 4 4 4 |
|  |  |  |  | Polarity | 7 7 7 7 6 | 7 6 6 6 6 | 7 7 1 1 6 | 7 7 1 1 6 | 7 7 7 7 6 |
| 729 | 9194 | 11598069\_103 | Under both conditions, an increase in LPS-induced TNF-alpha release from MNC was observed with increasing amounts of LBP (Fig. 2); |A1:\*\*1SP3E3+| |A2:\*\*1SP3E3+| |A3:\*\*1SP3E3+| |A4:\*\*1SP3E3+| |A5:\*\*1SP3E3+| no significant difference resulted from the different sequences of LPS and LBP addition (data not shown). |A1:\*\*2SP3E1| |A2:\*\*2SP3E1| |A3:\*\*2SN3E3| |A4:\*\*2SN3E3| |A5:\*\*2SN3E3| |  |  |  |  |  |  |
|  |  |  |  | Annotation | 1SP3E3+ 2SP3E1 | 1SP3E3+ 2SP3E1 | 1SP3E3+ 2SN3E3 | 1SP3E3+ 2SN3E3 | 1SP3E3+ 2SN3E3 |
|  |  |  |  | Evidence | 4 2 | 4 2 | 4 4 | 4 4 | 4 4 |
|  |  |  |  | Focus | 4 4 | 4 4 | 4 4 | 4 4 | 4 4 |
|  |  |  |  | Polarity | 7 7 | 7 7 | 7 1 | 7 1 | 7 1 |
| 730 | 8186 | 12139595\_8 | iNO), which was interpreted as the result of progressive dilution with nitrogen. |A1:\*\*1SP3E3| |A2:\*\*1SP3E1| |A3:\*\*1SP2E1| |A4:\*\*1SP2E1| |A5:\*\*1SP3E1| |  |  |  |  |  |  |
|  |  |  |  | Annotation | 1SP3E3 | 1SP3E1 | 1SP2E1 | 1SP2E1 | 1SP3E1 |
|  |  |  |  | Evidence | 4 | 2 | 2 | 2 | 2 |
|  |  |  |  | Focus | 4 | 4 | 4 | 4 | 4 |
|  |  |  |  | Polarity | 7 | 7 | 6 | 6 | 7 |
| 731 | 1606 | 9605989\_112 | The paired Student t test was performed to compare the effects of LPS and/or GM-CSF treatments on the production of IL-1beta and TNF-alpha at each time point. |A1:\*\*1MP3E3| |A2:\*\*1SP3E3| |A3:\*\*1SP3E3| |A4:\*\*1SP3E3| |A5:\*\*1MP3E3| |  |  |  |  |  |  |
|  |  |  |  | Annotation | 1MP3E3 | 1SP3E3 | 1SP3E3 | 1SP3E3 | 1MP3E3 |
|  |  |  |  | Evidence | 4 | 4 | 4 | 4 | 4 |
|  |  |  |  | Focus | 2 | 4 | 4 | 4 | 2 |
|  |  |  |  | Polarity | 7 | 7 | 7 | 7 | 7 |
| 732 | 2235 | 9606218\_223 | There is a remarkable association of skin laxity and fragility with the targeted lumican null mutation seen in this study, |A1:\*\*1SP3E3| |A2:\*\*1SP2E3| |A3:\*\*1SP3E3| |A4:\*\*1SP3E3| |A5:\*\*1SP3E3| and in the targeted decorin and more recently developed thrombospondin 2 null mutations ( 12, 23). |A1:\*\*2SP3E23| |A2:\*\*2SP2E2| |A3:\*\*2SP3E2| |A4:\*\*2SP3E2| |A5:\*\*2SP3E2| |  |  |  |  |  |  |
|  |  |  |  | Annotation | 1SP3E3 2SP3E23 | 1SP2E3 2SP2E2 | 1SP3E3 2SP3E2 | 1SP3E3 2SP3E2 | 1SP3E3 2SP3E2 |
|  |  |  |  | Evidence | 4 3 | 4 3 | 4 3 | 4 3 | 4 3 |
|  |  |  |  | Focus | 4 4 | 4 4 | 4 4 | 4 4 | 4 4 |
|  |  |  |  | Polarity | 7 7 | 6 6 | 7 7 | 7 7 | 7 7 |
| 733 | 1278 | 7775475\_7 | The APP was also detected by mAb 6E10 which recognizes an epitope of A4 (data not shown). |A1:\*\*1SP3E1| |A2:\*\*1SP3E1| |A3:\*\*1SP3E3| |A4:\*\*1SP3E3| |A5:\*\*1SP3E3| |  |  |  |  |  |  |
|  |  |  |  | Annotation | 1SP3E1 | 1SP3E1 | 1SP3E3 | 1SP3E3 | 1SP3E3 |
|  |  |  |  | Evidence | 2 | 2 | 4 | 4 | 4 |
|  |  |  |  | Focus | 4 | 4 | 4 | 4 | 4 |
|  |  |  |  | Polarity | 7 | 7 | 7 | 7 | 7 |
| 734 | 3166 | 12455503\_193 | Weaker staining is also apparent at 87A/C (Figure 6A). |A1:\*\*1SP3E3| |A2:\*\*1SP3E3| |A3:\*\*1MP3E3| |A4:\*\*1MP3E3| |A5:\*\*1GP3E3| |  |  |  |  |  |  |
|  |  |  |  | Annotation | 1SP3E3 | 1SP3E3 | 1MP3E3 | 1MP3E3 | 1GP3E3 |
|  |  |  |  | Evidence | 4 | 4 | 4 | 4 | 4 |
[truncated: 143,297 more chars]
