# Supplementary material for: How to Get the Most out of Your Curation Effort
Source: PLoS Comput Biol. 2009 May 22;5(5):e1000391. doi: 10.1371/journal.pcbi.1000391 (PMC2678295; doi:10.1371/journal.pcbi.1000391)
Supplement: Dataset S1 — Full dataset produced by the two-round annotation effort described in this study. (1.79 MB ZIP) [file pcbi.1000391.s002.zip › annotation-data/original8.html]

| # | No. in file | Id | Sentence | Columns | A | I | L | M | N | O | T | Y |
| --- | --- | --- | --- | --- | --- | --- | --- | --- | --- | --- | --- | --- |
| 1 | 1 | 9482734\_10 | These results suggest that rAxin negatively regulates the Wnt signaling pathway by interacting with GSK-3beta and beta-catenin |I:\*\*1SP1E0| and mediating the signal from GSK-3beta to beta-catenin. |I:\*\*2SP1E0| |L:\*\*1SN1E3| |T:\*\*1SN1E1| |
|  |  |  |  | Annotation |  | 1SP1E0 2SP1E0 | 1SN1E3 1SN1E3 |  |  |  | 1SN1E1 1SN1E1 |  |
|  |  |  |  | Evidence |  | 1 1 | 4 4 |  |  |  | 2 2 |  |
|  |  |  |  | Focus |  | 4 4 | 4 4 |  |  |  | 4 4 |  |
|  |  |  |  | Polarity |  | 5 5 | 3 3 |  |  |  | 3 3 |  |
| 2 | 2 | 11070089\_241 | Interestingly, TGF-beta signaling can directly interact with WNT signaling by a complex between beta-cateninT-cell factor/Leukemia enhancer factor and Smad4 on the Xtwn promoter ( 53). |I:\*\*1SP3E2| |L:\*\*1SP3E2| |T:\*\*1SP3E2| |
|  |  |  |  | Annotation |  | 1SP3E2 | 1SP3E2 |  |  |  | 1SP3E2 |  |
|  |  |  |  | Evidence |  | 3 | 3 |  |  |  | 3 |  |
|  |  |  |  | Focus |  | 4 | 4 |  |  |  | 4 |  |
|  |  |  |  | Polarity |  | 7 | 7 |  |  |  | 7 |  |
| 3 | 3 | 10612398\_26 | Because null mutations in toxR and toxT abolish CT and TcpA expression in the El Tor biotype |I:\*\*1SP3E0| and also attenuate virulence, |I:\*\*2SP3E0-| |L:\*\*1SN3E0| |T:\*\*1SP3E1| it is likely that the ToxR regulon has functional similarities between the two biotypes |I:\*\*3SP1E0| despite the clear differences in the inducing parameters observed in vitro. |I:\*\*4SP3E0| |L:\*\*2SP1E0| |T:\*\*2SP2E1| |
|  |  |  |  | Annotation |  | 1SP3E0 2SP3E0- 3SP1E0 4SP3E0 | 1SN3E0 1SN3E0 2SP1E0 2SP1E0 |  |  |  | 1SP3E1 1SP3E1 2SP2E1 2SP2E1 |  |
|  |  |  |  | Evidence |  | 1 1 1 1 | 1 1 1 1 |  |  |  | 2 2 2 2 |  |
|  |  |  |  | Focus |  | 4 4 4 4 | 4 4 4 4 |  |  |  | 4 4 4 4 |  |
|  |  |  |  | Polarity |  | 7 7 5 7 | 1 1 5 5 |  |  |  | 7 7 6 6 |  |
| 4 | 4 | 9425154\_262 | There, syntaxin was shown to suffice for alpha-SNAP binding. |I:\*\*1SP3E1| |L:\*\*1SP3E3| |T:\*\*1SP3E1| |
|  |  |  |  | Annotation |  | 1SP3E1 | 1SP3E3 |  |  |  | 1SP3E1 |  |
|  |  |  |  | Evidence |  | 2 | 4 |  |  |  | 2 |  |
|  |  |  |  | Focus |  | 4 | 4 |  |  |  | 4 |  |
|  |  |  |  | Polarity |  | 7 | 7 |  |  |  | 7 |  |
| 5 | 5 | 12079780\_95 | Specific C-terminal additions were observed in all of the selected clones when compared to the native protein sequence (Figure 4) . |I:\*\*1SP3E3| |L:\*\*1SP3E3| |T:\*\*1SP3E3| |
|  |  |  |  | Annotation |  | 1SP3E3 | 1SP3E3 |  |  |  | 1SP3E3 |  |
|  |  |  |  | Evidence |  | 4 | 4 |  |  |  | 4 |  |
|  |  |  |  | Focus |  | 4 | 4 |  |  |  | 4 |  |
|  |  |  |  | Polarity |  | 7 | 7 |  |  |  | 7 |  |
| 6 | 6 | 12445773\_270 | Under the transcription conditions, 30% of RNA transcripts were initiated with DePCoA (i.e., 30% CoA-RNA and 70% pppRNA) [15] . |I:\*\*1SP3E2| |L:\*\*1SP3E2| |T:\*\*1SP3E2| |
|  |  |  |  | Annotation |  | 1SP3E2 | 1SP3E2 |  |  |  | 1SP3E2 |  |
|  |  |  |  | Evidence |  | 3 | 3 |  |  |  | 3 |  |
|  |  |  |  | Focus |  | 4 | 4 |  |  |  | 4 |  |
|  |  |  |  | Polarity |  | 7 | 7 |  |  |  | 7 |  |
| 7 | 7 | 9230313\_188 | The observation that the major Axin mRNA is disrupted in two different alleles that cause axial duplications |I:\*\*1SP3E0| |L:\*\*1SN3E0| suggested that the normal gene product plays a negative regulatory role at some step in axis formation. |I:\*\*2SP1E0-| |L:\*\*2SN1E0| |T:\*\*1SP2E1| |
|  |  |  |  | Annotation |  | 1SP3E0 2SP1E0- | 1SN3E0 2SN1E0 |  |  |  | 1SP2E1 1SP2E1 |  |
|  |  |  |  | Evidence |  | 1 1 | 1 1 |  |  |  | 2 2 |  |
|  |  |  |  | Focus |  | 4 4 | 4 4 |  |  |  | 4 4 |  |
|  |  |  |  | Polarity |  | 7 5 | 1 3 |  |  |  | 6 6 |  |
| 8 | 8 | 10850496\_331 | Frank Ratcliff and Ana Montserrat Martin Hernandez provided access to unpublished tobacco rattle virus vector clones for which we are especially grateful. |I:\*\*1GP3E3| |L:\*\*1GP3E0| |T:\*\*1GP3E3| |
|  |  |  |  | Annotation |  | 1GP3E3 | 1GP3E0 |  |  |  | 1GP3E3 |  |
|  |  |  |  | Evidence |  | 4 | 1 |  |  |  | 4 |  |
|  |  |  |  | Focus |  | 1 | 1 |  |  |  | 1 |  |
|  |  |  |  | Polarity |  | 7 | 7 |  |  |  | 7 |  |
| 9 | 9 | 12110172\_40 | We show that double mutants between mes-1 or src-1 and each of several Wnt/g signaling components exhibit a complete loss of P2/EMS signaling, including a loss of the A/P division orientation in the EMS cell. |I:\*\*1SP3E3| |L:\*\*1SN3E3| |T:\*\*1SP3E3-| |
|  |  |  |  | Annotation |  | 1SP3E3 | 1SN3E3 |  |  |  | 1SP3E3- |  |
|  |  |  |  | Evidence |  | 4 | 4 |  |  |  | 4 |  |
|  |  |  |  | Focus |  | 4 | 4 |  |  |  | 4 |  |
|  |  |  |  | Polarity |  | 7 | 1 |  |  |  | 7 |  |
| 10 | 10 | 12218310\_7 | In June 2001, 15 patients were on home hemodialysis, 8 on training. |I:\*\*1MGP3E0| |L:\*\*1MP3E3| |T:\*\*1MP3E0| |
|  |  |  |  | Annotation |  | 1MGP3E0 | 1MP3E3 |  |  |  | 1MP3E0 |  |
|  |  |  |  | Evidence |  | 1 | 4 |  |  |  | 1 |  |
|  |  |  |  | Focus |  | 3 | 2 |  |  |  | 2 |  |
|  |  |  |  | Polarity |  | 7 | 7 |  |  |  | 7 |  |
| 11 | 11 | 12184266\_4 | Multialignment of the amino acid sequences between porcine and other mammalian species revealed that the sequences are highly conserved. |I:\*\*1SP3E0| |L:\*\*1SP3E0| |T:\*\*1SP3E1| |
|  |  |  |  | Annotation |  | 1SP3E0 | 1SP3E0 |  |  |  | 1SP3E1 |  |
|  |  |  |  | Evidence |  | 1 | 1 |  |  |  | 2 |  |
|  |  |  |  | Focus |  | 4 | 4 |  |  |  | 4 |  |
|  |  |  |  | Polarity |  | 7 | 7 |  |  |  | 7 |  |
| 12 | 12 | 10506157\_7 | RIalpha binding to domain B was lost or reduced with Y335V, A336V, and A340V mutations |I:\*\*1SP3E3-| |L:\*\*1SN3E3| |T:\*\*1SP3E0-| but retained with M343V/M344I double mutations (Fig. 1 a). |I:\*\*2SP3E3| |L:\*\*2SP3E3| |T:\*\*2SP3E3| |
|  |  |  |  | Annotation |  | 1SP3E3- 2SP3E3 | 1SN3E3 2SP3E3 |  |  |  | 1SP3E0- 2SP3E3 |  |
|  |  |  |  | Evidence |  | 4 4 | 4 4 |  |  |  | 1 4 |  |
|  |  |  |  | Focus |  | 4 4 | 4 4 |  |  |  | 4 4 |  |
|  |  |  |  | Polarity |  | 7 7 | 1 7 |  |  |  | 7 7 |  |
| 13 | 13 | 10428033\_161 | Thus, a simple purification of the intact holoenzyme can be effected using nickel affinity chromatography (see Experimental Procedures). |I:\*\*1MSP3E3| |L:\*\*1SMP1E3| |T:\*\*1MP3E3| |
|  |  |  |  | Annotation |  | 1MSP3E3 | 1SMP1E3 |  |  |  | 1MP3E3 |  |
|  |  |  |  | Evidence |  | 4 | 4 |  |  |  | 4 |  |
|  |  |  |  | Focus |  | 6 | 6 |  |  |  | 2 |  |
|  |  |  |  | Polarity |  | 7 | 5 |  |  |  | 7 |  |
| 14 | 14 | 10933391\_19 | Wnt signaling involves binding of a Wnt protein to a receptor of the frizzled family, |L:\*\*1SP3E2| inactivation of GSK-3 kinase activity, |L:\*\*2SN3E2| and stabilization of -catenin in the cytosol ( Willert et al. 1997 ). |L:\*\*3SP3E2| |I:\*\*1SP3E2| |T:\*\*1SP3E2| |
|  |  |  |  | Annotation |  | 1SP3E2 1SP3E2 1SP3E2 | 1SP3E2 2SN3E2 3SP3E2 |  |  |  | 1SP3E2 1SP3E2 1SP3E2 |  |
|  |  |  |  | Evidence |  | 3 3 3 | 3 3 3 |  |  |  | 3 3 3 |  |
|  |  |  |  | Focus |  | 4 4 4 | 4 4 4 |  |  |  | 4 4 4 |  |
|  |  |  |  | Polarity |  | 7 7 7 | 7 1 7 |  |  |  | 7 7 7 |  |
| 15 | 15 | 11149951\_16 | Dopamine receptors are divided into two subclasses, D1 and D2. |I:\*\*1SP3E0| |L:\*\*1SP3E0| |T:\*\*1GP3E1| |
|  |  |  |  | Annotation |  | 1SP3E0 | 1SP3E0 |  |  |  | 1GP3E1 |  |
|  |  |  |  | Evidence |  | 1 | 1 |  |  |  | 2 |  |
|  |  |  |  | Focus |  | 4 | 4 |  |  |  | 1 |  |
|  |  |  |  | Polarity |  | 7 | 7 |  |  |  | 7 |  |
| 16 | 16 | 11545738\_278 | For assays in which a ribonuclease A treatment was included, the resin was washed three times, incubated with 100 g/ml ribonuclease A in 100 l of wash buffer for 15 min at room temperature, and washed three more times. |I:\*\*1MP3E0| |L:\*\*1MP3E3| |T:\*\*1MP3E3| |
|  |  |  |  | Annotation |  | 1MP3E0 | 1MP3E3 |  |  |  | 1MP3E3 |  |
|  |  |  |  | Evidence |  | 1 | 4 |  |  |  | 4 |  |
|  |  |  |  | Focus |  | 2 | 2 |  |  |  | 2 |  |
|  |  |  |  | Polarity |  | 7 | 7 |  |  |  | 7 |  |
| 17 | 17 | 9614202\_38 | The resulting pellet was resuspended in the appropriate volume (40 mug of protein/400 mul) of assay buffer (buffer A plus 140 mug/ml bacitracin, 10 muM captopril, 1 mM dithiothreitol, and 0.1% bovine serum albumin). |I:\*\*1MP3E0| |L:\*\*1MP3E3| |T:\*\*1MP3E3| |
|  |  |  |  | Annotation |  | 1MP3E0 | 1MP3E3 |  |  |  | 1MP3E3 |  |
|  |  |  |  | Evidence |  | 1 | 4 |  |  |  | 4 |  |
|  |  |  |  | Focus |  | 2 | 2 |  |  |  | 2 |  |
|  |  |  |  | Polarity |  | 7 | 7 |  |  |  | 7 |  |
| 18 | 18 | 12123617\_312 | The aim of this hypothesis-driven study was, however, to identify the human equivalent of macaque anterior intraparietal area |I:\*\*1SGP0E0| by exploiting its crossmodal characteristics. |I:\*\*2MP3E0| |L:\*\*1GP3E0| |T:\*\*1GP3E3| |
|  |  |  |  | Annotation |  | 1SGP0E0 2MP3E0 | 1GP3E0 1GP3E0 |  |  |  | 1GP3E3 1GP3E3 |  |
|  |  |  |  | Evidence |  | 1 1 | 1 1 |  |  |  | 4 4 |  |
|  |  |  |  | Focus |  | 5 2 | 1 1 |  |  |  | 1 1 |  |
|  |  |  |  | Polarity |  | 4 7 | 7 7 |  |  |  | 7 7 |  |
| 19 | 19 | 11841936\_261 | The c-Src-as1 protein, homogeneously phosphorylated on Tyr 527, |I:\*\*1SP3E3| was purified as previously described for the wild-type protein [42] . |I:\*\*2MGP3E3| |L:\*\*1MP3E2| |T:\*\*1MP3E2| |
|  |  |  |  | Annotation |  | 1SP3E3 2MGP3E3 | 1MP3E2 1MP3E2 |  |  |  | 1MP3E2 1MP3E2 |  |
|  |  |  |  | Evidence |  | 4 4 | 3 3 |  |  |  | 3 3 |  |
|  |  |  |  | Focus |  | 4 3 | 2 2 |  |  |  | 2 2 |  |
|  |  |  |  | Polarity |  | 7 7 | 7 7 |  |  |  | 7 7 |  |
| 20 | 20 | 9473049\_184 | Second, according to the DLVO theory ( 20), there is a large free energy barrier that inhibits a particle from coming into close contact with the surface. |I:\*\*1SP3E3| |L:\*\*1SN3E2| |T:\*\*1GP3E2| |
|  |  |  |  | Annotation |  | 1SP3E3 | 1SN3E2 |  |  |  | 1GP3E2 |  |
|  |  |  |  | Evidence |  | 4 | 3 |  |  |  | 3 |  |
|  |  |  |  | Focus |  | 4 | 4 |  |  |  | 1 |  |
|  |  |  |  | Polarity |  | 7 | 1 |  |  |  | 7 |  |
| 21 | 21 | 12900447\_125 | (D) Wise functions extracellularly to block induction of siamois and Xnr3 by the Wnt pathway in ventral marginal zone (VMZ) explants. |I:\*\*ERROR| |L:\*\*1SN3E0+| |T:\*\*1SP3E1| |
|  |  |  |  | Annotation |  | ERROR | 1SN3E0+ |  |  |  | 1SP3E1 |  |
|  |  |  |  | Evidence |  | -1000 | 1 |  |  |  | 2 |  |
|  |  |  |  | Focus |  | -1000 | 4 |  |  |  | 4 |  |
|  |  |  |  | Polarity |  | -1000 | 1 |  |  |  | 7 |  |
| 22 | 22 | 12107431\_1 | We have analysed protein trafficking during the differentiation of rat L6 myoblasts into myotubes. |I:\*\*1SGP3E3| |L:\*\*1GSP3E3| |T:\*\*1MP3E1| |
|  |  |  |  | Annotation |  | 1SGP3E3 | 1GSP3E3 |  |  |  | 1MP3E1 |  |
|  |  |  |  | Evidence |  | 4 | 4 |  |  |  | 2 |  |
|  |  |  |  | Focus |  | 5 | 5 |  |  |  | 2 |  |
|  |  |  |  | Polarity |  | 7 | 7 |  |  |  | 7 |  |
| 23 | 23 | 10508081\_67 | Micropipettes were calibrated before and after each experiment by delivering aliquots of the respective tracer solution directly into LSC vials filled with scintillation cocktail. |I:\*\*1MP3E3| |L:\*\*1MP3E3| |T:\*\*1MP3E1| |
|  |  |  |  | Annotation |  | 1MP3E3 | 1MP3E3 |  |  |  | 1MP3E1 |  |
|  |  |  |  | Evidence |  | 4 | 4 |  |  |  | 2 |  |
|  |  |  |  | Focus |  | 2 | 2 |  |  |  | 2 |  |
|  |  |  |  | Polarity |  | 7 | 7 |  |  |  | 7 |  |
| 24 | 24 | 9187256\_159 | Indeed, Wu et al. ( 24) showed that sodium salicylate could inhibit the induction of COX in human umbilical endothelial cells stimulated by IL-1beta. |I:\*\*1SP3E2| |L:\*\*1SN1E2+| |T:\*\*1SN3E2+| |
|  |  |  |  | Annotation |  | 1SP3E2 | 1SN1E2+ |  |  |  | 1SN3E2+ |  |
|  |  |  |  | Evidence |  | 3 | 3 |  |  |  | 3 |  |
|  |  |  |  | Focus |  | 4 | 4 |  |  |  | 4 |  |
|  |  |  |  | Polarity |  | 7 | 3 |  |  |  | 1 |  |
| 25 | 25 | 10862775\_20 | The positions of molecular mass standards are shown on the right. |I:\*\*1MGP3E3| |L:\*\*1SP3E3| |T:\*\*1GP3E3| |
|  |  |  |  | Annotation |  | 1MGP3E3 | 1SP3E3 |  |  |  | 1GP3E3 |  |
|  |  |  |  | Evidence |  | 4 | 4 |  |  |  | 4 |  |
|  |  |  |  | Focus |  | 3 | 4 |  |  |  | 1 |  |
|  |  |  |  | Polarity |  | 7 | 7 |  |  |  | 7 |  |
| 26 | 26 | 10748004\_34 | These two adapter proteins lead to the activation of NFkappaB as well as the activation of the stress-activated protein kinases (SAPKs) such as c-Jun N-terminal kinase (JNK) and p38 MAPK |T:\*\*1SP3E1| |I:\*\*1SP3E0| |L:\*\*1SP3E2+| IL-1 also activates NFkappaB and SAPKs using a similar pathway initiated by ligand binding to the type 1 IL-1 receptor (IL-1R1) and involving adapter proteins MyD88, IL-1 receptor-associated kinase (IRAK), and TRAF6 ( 36, 37). |T:\*\*1SP3E2| |I:\*\*1SP3E2| |L:\*\*1SP3E2+| |
|  |  |  |  | Annotation |  | 1SP3E0 1SP3E2 | 1SP3E2+ 1SP3E2+ |  |  |  | 1SP3E1 1SP3E2 |  |
|  |  |  |  | Evidence |  | 1 3 | 3 3 |  |  |  | 2 3 |  |
|  |  |  |  | Focus |  | 4 4 | 4 4 |  |  |  | 4 4 |  |
|  |  |  |  | Polarity |  | 7 7 | 7 7 |  |  |  | 7 7 |  |
| 27 | 27 | 11897598\_3 | Azithromycin-trimethoprim-sulfamethoxazole, azithromycin-ceftazidime, and azithromycin-doxycycline or azithromycin-trimethoprim-sulfamethoxazole inhibited 40, 20, and 22% of Stenotrophomonas maltophilia, Burkholderia cepacia complex, and Achromobacter (Alcaligenes) xylosoxidans strains, respectively. |I:\*\*1SP3E0| |L:\*\*1SP3E3-| |T:\*\*1SN3E1| |
|  |  |  |  | Annotation |  | 1SP3E0 | 1SP3E3- |  |  |  | 1SN3E1 |  |
|  |  |  |  | Evidence |  | 1 | 4 |  |  |  | 2 |  |
|  |  |  |  | Focus |  | 4 | 4 |  |  |  | 4 |  |
|  |  |  |  | Polarity |  | 7 | 7 |  |  |  | 1 |  |
| 28 | 28 | 10790410\_139 | In over 96% of the pots planted, at least one plant germinated from the four seeds sown, and the plant that was retained after thinning survived to reproduction. |I:\*\*1SGP3E0| |L:\*\*1SP3E0| |T:\*\*1SP3E1| |
|  |  |  |  | Annotation |  | 1SGP3E0 | 1SP3E0 |  |  |  | 1SP3E1 |  |
|  |  |  |  | Evidence |  | 1 | 1 |  |  |  | 2 |  |
|  |  |  |  | Focus |  | 5 | 4 |  |  |  | 4 |  |
|  |  |  |  | Polarity |  | 7 | 7 |  |  |  | 7 |  |
| 29 | 29 | 11606579\_46 | This proposal is consistent with the fact |I:\*\*1SP3E0| that the R72A substitution does not affect incorporation of alpha-BH3-dTTP, alpha-BH3-AZTTP, nor alpha-BH3-d4TP analogues, |I:\*\*2SN3E2| |L:\*\*1SN3E2| |T:\*\*1SN3E1| but leads to a severely impaired RT (>200-fold) using natural dNTPs ( ). |I:\*\*3SP3E2| |L:\*\*2SP3E2| |T:\*\*2SP3E1| |
|  |  |  |  | Annotation |  | 1SP3E0 2SN3E2 3SP3E2 | 1SN3E2 1SN3E2 2SP3E2 |  |  |  | 1SN3E1 1SN3E1 2SP3E1 |  |
|  |  |  |  | Evidence |  | 1 3 3 | 3 3 3 |  |  |  | 2 2 2 |  |
|  |  |  |  | Focus |  | 4 4 4 | 4 4 4 |  |  |  | 4 4 4 |  |
|  |  |  |  | Polarity |  | 7 1 7 | 1 1 7 |  |  |  | 1 1 7 |  |
| 30 | 30 | 10721990\_248 | Here we show that WNT-7a, secreted by GCs, plays a role in this process. |I:\*\*1SP3E3| |L:\*\*1SP3E3| |T:\*\*1SP3E3| |
|  |  |  |  | Annotation |  | 1SP3E3 | 1SP3E3 |  |  |  | 1SP3E3 |  |
|  |  |  |  | Evidence |  | 4 | 4 |  |  |  | 4 |  |
|  |  |  |  | Focus |  | 4 | 4 |  |  |  | 4 |  |
|  |  |  |  | Polarity |  | 7 | 7 |  |  |  | 7 |  |
| 31 | 31 | 9584202\_310 | We found that L-DNase II induces the cleavage of DNA into an oligonucleosomal ladder, a hallmark of apoptosis, and the appearance of nuclear pycnosis, resembling morphological changes seen in apoptosis. |I:\*\*1SP3E3| |L:\*\*1SP2E3| |T:\*\*1SP3E1+| |
|  |  |  |  | Annotation |  | 1SP3E3 | 1SP2E3 |  |  |  | 1SP3E1+ |  |
|  |  |  |  | Evidence |  | 4 | 4 |  |  |  | 2 |  |
|  |  |  |  | Focus |  | 4 | 4 |  |  |  | 4 |  |
|  |  |  |  | Polarity |  | 7 | 6 |  |  |  | 7 |  |
| 32 | 32 | 10619431\_99 | VAB-1-AP binding to cells expressing VAB-2/EFN-1 was detectable |I:\*\*1SP3E3| by staining for AP ( Figure 2F and Figure 2G) |I:\*\*2MP3E3| |L:\*\*1SP3E3| and was abolished |I:\*\*3MGP3E3| by pretreatment with PI-PLC or with anti-VAB-2/EFN-1 antibodies ( Figure 2G, and data not shown). |I:\*\*4MP3E3| |L:\*\*2SP3E3-| |T:\*\*1SP3E3| |
|  |  |  |  | Annotation |  | 1SP3E3 2MP3E3 3MGP3E3 4MP3E3 | 1SP3E3 1SP3E3 2SP3E3- 2SP3E3- |  |  |  | 1SP3E3 1SP3E3 1SP3E3 1SP3E3 |  |
|  |  |  |  | Evidence |  | 4 4 4 4 | 4 4 4 4 |  |  |  | 4 4 4 4 |  |
|  |  |  |  | Focus |  | 4 2 3 2 | 4 4 4 4 |  |  |  | 4 4 4 4 |  |
|  |  |  |  | Polarity |  | 7 7 7 7 | 7 7 7 7 |  |  |  | 7 7 7 7 |  |
| 33 | 33 | 9244304\_297 | Total RNA was extracted from 560 mul of plasma (QIAamp; Qiagen). |I:\*\*1MP3E0| |L:\*\*1MP3E3| |T:\*\*1MP3E3| |
|  |  |  |  | Annotation |  | 1MP3E0 | 1MP3E3 |  |  |  | 1MP3E3 |  |
|  |  |  |  | Evidence |  | 1 | 4 |  |  |  | 4 |  |
|  |  |  |  | Focus |  | 2 | 2 |  |  |  | 2 |  |
|  |  |  |  | Polarity |  | 7 | 7 |  |  |  | 7 |  |
| 34 | 34 | 11703922\_220 | FGFR1 could regulate the Wnt signaling pathway at the streak by influencing levels of cytosolic -catenin. |I:\*\*1SP3E0| |L:\*\*1SP1E0| |T:\*\*1SP1E1| |
|  |  |  |  | Annotation |  | 1SP3E0 | 1SP1E0 |  |  |  | 1SP1E1 |  |
|  |  |  |  | Evidence |  | 1 | 1 |  |  |  | 2 |  |
|  |  |  |  | Focus |  | 4 | 4 |  |  |  | 4 |  |
|  |  |  |  | Polarity |  | 7 | 5 |  |  |  | 5 |  |
| 35 | 35 | 11320079\_15 | None of the refolded mutants, however, possessed hydrolase activity, |I:\*\*1SN3E0| implying that His106 is an important catalytic residue. |I:\*\*2SP1E0| |L:\*\*1SP1E0| |T:\*\*1SP3E1| |
|  |  |  |  | Annotation |  | 1SN3E0 2SP1E0 | 1SP1E0 1SP1E0 |  |  |  | 1SP3E1 1SP3E1 |  |
|  |  |  |  | Evidence |  | 1 1 | 1 1 |  |  |  | 2 2 |  |
|  |  |  |  | Focus |  | 4 4 | 4 4 |  |  |  | 4 4 |  |
|  |  |  |  | Polarity |  | 1 5 | 5 5 |  |  |  | 7 7 |  |
| 36 | 36 | 9786955\_260 | Consequently, it can be anticipated that in addition to CDKs, additional pathway-specific elements must distinguish the mechanism by which DNA-damaging agents trigger neuronal apoptosis. |I:\*\*1SP1E3| |L:\*\*1SP2E0| |T:\*\*1SP3E1+| |
|  |  |  |  | Annotation |  | 1SP1E3 | 1SP2E0 |  |  |  | 1SP3E1+ |  |
|  |  |  |  | Evidence |  | 4 | 1 |  |  |  | 2 |  |
|  |  |  |  | Focus |  | 4 | 4 |  |  |  | 4 |  |
|  |  |  |  | Polarity |  | 5 | 6 |  |  |  | 7 |  |
| 37 | 37 | 7642521\_26 | Supported by National Institutes of Health Training Grant HL 076922. |I:\*\*1GP3E3| |L:\*\*1GP3E3| |T:\*\*1GP3E3| |
|  |  |  |  | Annotation |  | 1GP3E3 | 1GP3E3 |  |  |  | 1GP3E3 |  |
|  |  |  |  | Evidence |  | 4 | 4 |  |  |  | 4 |  |
|  |  |  |  | Focus |  | 1 | 1 |  |  |  | 1 |  |
|  |  |  |  | Polarity |  | 7 | 7 |  |  |  | 7 |  |
| 38 | 38 | 9431909\_27 | Fifty-milliliter aliquots of each spirochetal culture were centrifuged at 10,000 x g for 30 min and the supernatants were removed. |I:\*\*1MP3E0| |L:\*\*1MP3E3| |T:\*\*1MP3E3| |
|  |  |  |  | Annotation |  | 1MP3E0 | 1MP3E3 |  |  |  | 1MP3E3 |  |
|  |  |  |  | Evidence |  | 1 | 4 |  |  |  | 4 |  |
|  |  |  |  | Focus |  | 2 | 2 |  |  |  | 2 |  |
|  |  |  |  | Polarity |  | 7 | 7 |  |  |  | 7 |  |
| 39 | 39 | 9261171\_75 | Immunoblot analysis ( panel A) and kinase activity ( panel B) of PKB from vanadate and peroxovanadate- stimulated adipocytes. |I:\*\*1SGP3E3| |L:\*\*1SP3E3+| |T:\*\*1MP3E3| |
|  |  |  |  | Annotation |  | 1SGP3E3 | 1SP3E3+ |  |  |  | 1MP3E3 |  |
|  |  |  |  | Evidence |  | 4 | 4 |  |  |  | 4 |  |
|  |  |  |  | Focus |  | 5 | 4 |  |  |  | 2 |  |
|  |  |  |  | Polarity |  | 7 | 7 |  |  |  | 7 |  |
| 40 | 40 | 12837243\_281 | sFRPs block Wnt signaling through direct binding (Lescher et al., 1998 ). |I:\*\*1SP3E2| |L:\*\*1SP3E2-| |T:\*\*1SN3E2| |
|  |  |  |  | Annotation |  | 1SP3E2 | 1SP3E2- |  |  |  | 1SN3E2 |  |
|  |  |  |  | Evidence |  | 3 | 3 |  |  |  | 3 |  |
|  |  |  |  | Focus |  | 4 | 4 |  |  |  | 4 |  |
|  |  |  |  | Polarity |  | 7 | 7 |  |  |  | 1 |  |
| 41 | 41 | 10688669\_423 | Regulation of glycogen synthase kinase 3beta and downstream Wnt signaling by Axin. |I:\*\*ERROR| |L:\*\*1SP3E0| |T:\*\*1GP3E0| |
|  |  |  |  | Annotation |  | ERROR | 1SP3E0 |  |  |  | 1GP3E0 |  |
|  |  |  |  | Evidence |  | -1000 | 1 |  |  |  | 1 |  |
|  |  |  |  | Focus |  | -1000 | 4 |  |  |  | 1 |  |
|  |  |  |  | Polarity |  | -1000 | 7 |  |  |  | 7 |  |
| 42 | 42 | 8798681\_28 | Tel.: 81-6-879-3510; Fax: 81-6-879-3519; E-mail: ykurachi@pharma2.med.osaka-u.ac.jp. |I:\*\*1GP3E3| |L:\*\*1GP3E3| |T:\*\*1GP3E3| |
|  |  |  |  | Annotation |  | 1GP3E3 | 1GP3E3 |  |  |  | 1GP3E3 |  |
|  |  |  |  | Evidence |  | 4 | 4 |  |  |  | 4 |  |
|  |  |  |  | Focus |  | 1 | 1 |  |  |  | 1 |  |
|  |  |  |  | Polarity |  | 7 | 7 |  |  |  | 7 |  |
| 43 | 43 | 9312078\_25 | Indeed, this was the case (Fig. 4). |I:\*\*1GP3E3| |L:\*\*1SP3E3| |T:\*\*1SP3E3| |
|  |  |  |  | Annotation |  | 1GP3E3 | 1SP3E3 |  |  |  | 1SP3E3 |  |
|  |  |  |  | Evidence |  | 4 | 4 |  |  |  | 4 |  |
|  |  |  |  | Focus |  | 1 | 4 |  |  |  | 4 |  |
|  |  |  |  | Polarity |  | 7 | 7 |  |  |  | 7 |  |
| 44 | 44 | 9539416\_290 | The genetic characterization of these mutant forms of Gbeta led to two conclusions: (1) Gbetagamma is a target of Galpha-mediated adaptation |L:\*\*1SP3E3| and |I:\*\*1SP3E0| |T:\*\*1SP3E1| (2) the adaptive mechanism or mechanisms impaired by the lesions in STE4 do not involve Gbeta phosphorylation. |I:\*\*2SN3E0| |L:\*\*2SN3E3-| |T:\*\*2SN3E1| |
|  |  |  |  | Annotation |  | 1SP3E0 1SP3E0 2SN3E0 | 1SP3E3 2SN3E3- 2SN3E3- |  |  |  | 1SP3E1 1SP3E1 2SN3E1 |  |
|  |  |  |  | Evidence |  | 1 1 1 | 4 4 4 |  |  |  | 2 2 2 |  |
|  |  |  |  | Focus |  | 4 4 4 | 4 4 4 |  |  |  | 4 4 4 |  |
|  |  |  |  | Polarity |  | 7 7 1 | 7 1 1 |  |  |  | 7 7 1 |  |
| 45 | 45 | 9581763\_481 | Slices were transferred to slides, air dried for 4 hr, dipped in Xylene for 3 min, and mounted with coverslips using Permount. |I:\*\*1MP3E0| |L:\*\*1MP3E3| |T:\*\*1MP3E3| |
|  |  |  |  | Annotation |  | 1MP3E0 | 1MP3E3 |  |  |  | 1MP3E3 |  |
|  |  |  |  | Evidence |  | 1 | 4 |  |  |  | 4 |  |
|  |  |  |  | Focus |  | 2 | 2 |  |  |  | 2 |  |
|  |  |  |  | Polarity |  | 7 | 7 |  |  |  | 7 |  |
| 46 | 46 | 12403814\_127 | Thus, from the dozens of different proteins present in the cytoskeleton preparations, myosin Va bound specifically to three structurally related IF proteins. |I:\*\*1SP3E0| |L:\*\*1SP3E0| |T:\*\*1SP3E1| |
|  |  |  |  | Annotation |  | 1SP3E0 | 1SP3E0 |  |  |  | 1SP3E1 |  |
|  |  |  |  | Evidence |  | 1 | 1 |  |  |  | 2 |  |
|  |  |  |  | Focus |  | 4 | 4 |  |  |  | 4 |  |
|  |  |  |  | Polarity |  | 7 | 7 |  |  |  | 7 |  |
| 47 | 47 | 10816498\_200 | It includes 16 potential sites for O-linked glycan attachment, 9 possible N-linked glycosylation sites, and a putative glycosaminoglycan attachment site ( 28). |I:\*\*1SP3E2| |L:\*\*1SP3E2| |T:\*\*1SP3E2| |
|  |  |  |  | Annotation |  | 1SP3E2 | 1SP3E2 |  |  |  | 1SP3E2 |  |
|  |  |  |  | Evidence |  | 3 | 3 |  |  |  | 3 |  |
|  |  |  |  | Focus |  | 4 | 4 |  |  |  | 4 |  |
|  |  |  |  | Polarity |  | 7 | 7 |  |  |  | 7 |  |
| 48 | 48 | 9390512\_19 | Second, twin studies comparing the concordance for schizophrenia in monozygotic (MZ) twin pairs |I:\*\*1SGP3E1| (who, in principle, share all of their genes) |I:\*\*2SP3E1| with the concordance in dizygotic (DZ) twin pairs |I:\*\*3SGP3E1| (who share half of their genes) |I:\*\*4SP3E1| demonstrated that the MZ concordance rate (46%-48%) is significantly higher than the DZ rate (4%-14%). |I:\*\*5SP3E1| |L:\*\*1SP3E0| |T:\*\*1SP3E1| |
|  |  |  |  | Annotation |  | 1SGP3E1 2SP3E1 3SGP3E1 4SP3E1 5SP3E1 | 1SP3E0 1SP3E0 1SP3E0 1SP3E0 1SP3E0 |  |  |  | 1SP3E1 1SP3E1 1SP3E1 1SP3E1 1SP3E1 |  |
|  |  |  |  | Evidence |  | 2 2 2 2 2 | 1 1 1 1 1 |  |  |  | 2 2 2 2 2 |  |
|  |  |  |  | Focus |  | 5 4 5 4 4 | 4 4 4 4 4 |  |  |  | 4 4 4 4 4 |  |
|  |  |  |  | Polarity |  | 7 7 7 7 7 | 7 7 7 7 7 |  |  |  | 7 7 7 7 7 |  |
| 49 | 49 | 10473520\_117 | In our ECLIA system, the profiles of BDV antibody differed between patients with psychiatric disorders and control donors. |I:\*\*1MP3E3| |L:\*\*1SP3E3| |T:\*\*1SP3E1| |
|  |  |  |  | Annotation |  | 1MP3E3 | 1SP3E3 |  |  |  | 1SP3E1 |  |
|  |  |  |  | Evidence |  | 4 | 4 |  |  |  | 2 |  |
|  |  |  |  | Focus |  | 2 | 4 |  |  |  | 4 |  |
|  |  |  |  | Polarity |  | 7 | 7 |  |  |  | 7 |  |
| 50 | 50 | 9784528\_37 | The pellets of both cultures were adjusted to 125 Klett units by resuspending in ice-cold invasion medium. |I:\*\*1MP3E0| |L:\*\*1MP3E3| |T:\*\*1MP3E3| |
|  |  |  |  | Annotation |  | 1MP3E0 | 1MP3E3 |  |  |  | 1MP3E3 |  |
|  |  |  |  | Evidence |  | 1 | 4 |  |  |  | 4 |  |
|  |  |  |  | Focus |  | 2 | 2 |  |  |  | 2 |  |
|  |  |  |  | Polarity |  | 7 | 7 |  |  |  | 7 |  |
| 51 | 51 | 9566905\_9 | These results indicate that Axil negatively regulates the Wnt signaling pathway by mediating GSK-3beta-dependent phosphorylation of beta-catenin, thereby inhibiting axis formation. |I:\*\*1SP3E0| |L:\*\*1SP1E3-| |T:\*\*1SN3E1| |
|  |  |  |  | Annotation |  | 1SP3E0 | 1SP1E3- |  |  |  | 1SN3E1 |  |
|  |  |  |  | Evidence |  | 1 | 4 |  |  |  | 2 |  |
|  |  |  |  | Focus |  | 4 | 4 |  |  |  | 4 |  |
|  |  |  |  | Polarity |  | 7 | 5 |  |  |  | 1 |  |
| 52 | 52 | 11238388\_237 | The Lorist6Xh is 6.6 kbp. +, Watson strand; -, the Crick strand. |I:\*\*1SGP3E3| |L:\*\*1SP3E0| |T:\*\*1SP3E1| |
|  |  |  |  | Annotation |  | 1SGP3E3 | 1SP3E0 |  |  |  | 1SP3E1 |  |
|  |  |  |  | Evidence |  | 4 | 1 |  |  |  | 2 |  |
|  |  |  |  | Focus |  | 5 | 4 |  |  |  | 4 |  |
|  |  |  |  | Polarity |  | 7 | 7 |  |  |  | 7 |  |
| 53 | 53 | 9336330\_116 | The mean maximal startle response to the 120 dB stimulus presented alone |I:\*\*1SP3E0| and when preceded by 70, 75 and 80 dB prepulse stimuli (in the aged animal experiment) or by 67, 70 and 75 dB (in the septal lesion experiments) was measured. |I:\*\*2MP3E0| |L:\*\*1MP3E3| |T:\*\*1MP3E3| |
|  |  |  |  | Annotation |  | 1SP3E0 2MP3E0 | 1MP3E3 1MP3E3 |  |  |  | 1MP3E3 1MP3E3 |  |
|  |  |  |  | Evidence |  | 1 1 | 4 4 |  |  |  | 4 4 |  |
|  |  |  |  | Focus |  | 4 2 | 2 2 |  |  |  | 2 2 |  |
|  |  |  |  | Polarity |  | 7 7 | 7 7 |  |  |  | 7 7 |  |
| 54 | 54 | 10338499\_58 | (B6 x DBA/2)F1 mice were bred from parents obtained from Jackson Laboratories, and at the age of 8 weeks they were mated with B6 mice. |I:\*\*1MP3E0| |L:\*\*1MP3E3| |T:\*\*1MP3E3| |
|  |  |  |  | Annotation |  | 1MP3E0 | 1MP3E3 |  |  |  | 1MP3E3 |  |
|  |  |  |  | Evidence |  | 1 | 4 |  |  |  | 4 |  |
|  |  |  |  | Focus |  | 2 | 2 |  |  |  | 2 |  |
|  |  |  |  | Polarity |  | 7 | 7 |  |  |  | 7 |  |
| 55 | 55 | 9755208\_163 | A related motivation for surveying microsatellites was to assess species-effective population size, |I:\*\*1SGP0E0| as this is also useful in interpreting the demography of this species. |I:\*\*2SGP3E0| |L:\*\*1GP3E0| |T:\*\*1GP3E3| |
|  |  |  |  | Annotation |  | 1SGP0E0 2SGP3E0 | 1GP3E0 1GP3E0 |  |  |  | 1GP3E3 1GP3E3 |  |
|  |  |  |  | Evidence |  | 1 1 | 1 1 |  |  |  | 4 4 |  |
|  |  |  |  | Focus |  | 5 5 | 1 1 |  |  |  | 1 1 |  |
|  |  |  |  | Polarity |  | 4 7 | 7 7 |  |  |  | 7 7 |  |
| 56 | 56 | 12895418\_291 | The Nodal signaling pathway has conserved roles in regulating asymmetry and laterality throughout vertebrates (Hamada et al., 2002 ; Boorman and Shimeld, 2002 ). |I:\*\*1SP3E2| |L:\*\*1SP3E2| |T:\*\*1GP3E2| |
|  |  |  |  | Annotation |  | 1SP3E2 | 1SP3E2 |  |  |  | 1GP3E2 |  |
|  |  |  |  | Evidence |  | 3 | 3 |  |  |  | 3 |  |
|  |  |  |  | Focus |  | 4 | 4 |  |  |  | 1 |  |
|  |  |  |  | Polarity |  | 7 | 7 |  |  |  | 7 |  |
| 57 | 57 | 9572968\_52 | From enrichments, pure cultures were obtained by repeated streaking of diluted samples on agar plates (1.0% [wt/vol] Difco agar) with lake water medium. |I:\*\*1MP3E0| |L:\*\*1MP3E3| |T:\*\*1MP3E3| |
|  |  |  |  | Annotation |  | 1MP3E0 | 1MP3E3 |  |  |  | 1MP3E3 |  |
|  |  |  |  | Evidence |  | 1 | 4 |  |  |  | 4 |  |
|  |  |  |  | Focus |  | 2 | 2 |  |  |  | 2 |  |
|  |  |  |  | Polarity |  | 7 | 7 |  |  |  | 7 |  |
| 58 | 58 | 12174719\_8 | Having the dog "sit" and praising it lead to a positive correlation in all pairs. |I:\*\*1SP3E0| |L:\*\*1SP3E0+| |T:\*\*1SP3E3| |
|  |  |  |  | Annotation |  | 1SP3E0 | 1SP3E0+ |  |  |  | 1SP3E3 |  |
|  |  |  |  | Evidence |  | 1 | 1 |  |  |  | 4 |  |
|  |  |  |  | Focus |  | 4 | 4 |  |  |  | 4 |  |
|  |  |  |  | Polarity |  | 7 | 7 |  |  |  | 7 |  |
| 59 | 59 | 9331360\_24 | Alternative splicing has previously been shown to modulate the functional properties of Shaker voltage-gated K+ channels ( Iverson et al. 1988 ; Timpe et al. 1988 ). |I:\*\*1SP3E2| |L:\*\*1SP3E2| |T:\*\*1SP3E2| |
|  |  |  |  | Annotation |  | 1SP3E2 | 1SP3E2 |  |  |  | 1SP3E2 |  |
|  |  |  |  | Evidence |  | 3 | 3 |  |  |  | 3 |  |
|  |  |  |  | Focus |  | 4 | 4 |  |  |  | 4 |  |
|  |  |  |  | Polarity |  | 7 | 7 |  |  |  | 7 |  |
| 60 | 60 | 10811836\_235 | It is speculated that collagen X may comprise the hexagonal network ( Kwan et al. 1991 ; Chan and Jacenko 1998 ), |I:\*\*1SP1E2| and may even persist in the marrow stroma (our unpublished observations). |I:\*\*2SP1E3| |L:\*\*1SP2E23| |T:\*\*1SP1E2| |
|  |  |  |  | Annotation |  | 1SP1E2 2SP1E3 | 1SP2E23 1SP2E23 |  |  |  | 1SP1E2 1SP1E2 |  |
|  |  |  |  | Evidence |  | 3 4 | 3 3 |  |  |  | 3 3 |  |
|  |  |  |  | Focus |  | 4 4 | 4 4 |  |  |  | 4 4 |  |
|  |  |  |  | Polarity |  | 5 5 | 6 6 |  |  |  | 5 5 |  |
| 61 | 61 | 11326002\_39 | Protease sequences have been submitted to the GenBank database with the accession numbers AF247007 to AF247038. |I:\*\*1SGP3E3| |L:\*\*1GP3E3| |T:\*\*1GP3E3| |
|  |  |  |  | Annotation |  | 1SGP3E3 | 1GP3E3 |  |  |  | 1GP3E3 |  |
|  |  |  |  | Evidence |  | 4 | 4 |  |  |  | 4 |  |
|  |  |  |  | Focus |  | 5 | 1 |  |  |  | 1 |  |
|  |  |  |  | Polarity |  | 7 | 7 |  |  |  | 7 |  |
| 62 | 62 | 10679322\_60 | As shown in Figure 1c, cotransfection of Mek-DD together with a kinase inactive Rsk1 allele (Rsk1 ), which carries inactivating point mutations in both the amino-terminal and carboxy-terminal kinase domains (K112R,K464R), eliminated the protective effect of Mek-DD. |I:\*\*1SP3E3| |L:\*\*1SP3E3-| |T:\*\*1SN3E3| |
|  |  |  |  | Annotation |  | 1SP3E3 | 1SP3E3- |  |  |  | 1SN3E3 |  |
|  |  |  |  | Evidence |  | 4 | 4 |  |  |  | 4 |  |
|  |  |  |  | Focus |  | 4 | 4 |  |  |  | 4 |  |
|  |  |  |  | Polarity |  | 7 | 7 |  |  |  | 1 |  |
| 63 | 63 | 9039267\_292 | For immunoprecipitations, the supernatant was incubated with 100 mul of 12CA5 hybridoma supernatant, which was coupled to protein A sepharose beads at 4 degrees C. |I:\*\*1MP3E0| |L:\*\*1MP3E3| |T:\*\*1MP3E3| |
|  |  |  |  | Annotation |  | 1MP3E0 | 1MP3E3 |  |  |  | 1MP3E3 |  |
|  |  |  |  | Evidence |  | 1 | 4 |  |  |  | 4 |  |
|  |  |  |  | Focus |  | 2 | 2 |  |  |  | 2 |  |
|  |  |  |  | Polarity |  | 7 | 7 |  |  |  | 7 |  |
| 64 | 64 | 11896138\_11 | Moreover, gender specific effects of the two components of the effort-reward imbalance model were observed. |I:\*\*1SP3E0| |L:\*\*1SP3E3| |T:\*\*1SP3E1| |
|  |  |  |  | Annotation |  | 1SP3E0 | 1SP3E3 |  |  |  | 1SP3E1 |  |
|  |  |  |  | Evidence |  | 1 | 4 |  |  |  | 2 |  |
|  |  |  |  | Focus |  | 4 | 4 |  |  |  | 4 |  |
|  |  |  |  | Polarity |  | 7 | 7 |  |  |  | 7 |  |
| 65 | 65 | 12216498\_8 | Was classified from the point of clinical view in three series: N series: 27 patient TBC+ BK-, P Series: 28 patient TBC+ BK+ and EP Series: 2 patient with extra pulmonary TBC. |I:\*\*ERROR| |L:\*\*1MP3E3| |T:\*\*1SP3E3| |
|  |  |  |  | Annotation |  | ERROR | 1MP3E3 |  |  |  | 1SP3E3 |  |
|  |  |  |  | Evidence |  | -1000 | 4 |  |  |  | 4 |  |
|  |  |  |  | Focus |  | -1000 | 2 |  |  |  | 4 |  |
|  |  |  |  | Polarity |  | -1000 | 7 |  |  |  | 7 |  |
| 66 | 66 | 10197537\_330 | The following measures were assessed for both halves of the test session |I:\*\*1SGP0E3| by the Polytrack video system: number of entries into the central region, total time spent in the central region, and overall motor activity (transitions between all five regions). |I:\*\*2MP3E3| |L:\*\*1MP3E3| |T:\*\*1MP3E3| |
|  |  |  |  | Annotation |  | 1SGP0E3 2MP3E3 | 1MP3E3 1MP3E3 |  |  |  | 1MP3E3 1MP3E3 |  |
|  |  |  |  | Evidence |  | 4 4 | 4 4 |  |  |  | 4 4 |  |
|  |  |  |  | Focus |  | 5 2 | 2 2 |  |  |  | 2 2 |  |
|  |  |  |  | Polarity |  | 4 7 | 7 7 |  |  |  | 7 7 |  |
| 67 | 67 | 12183362\_30 | In the present study, we describe a novel protein, |I:\*\*1SGP3E3| which inhibits the canonical Wnt pathway |L:\*\*1SP3E3-| and promotes signaling of the Wnt/JNK pathway. |I:\*\*2SP3E3| |L:\*\*2SP3E3+| |T:\*\*1GP3E3| |
|  |  |  |  | Annotation |  | 1SGP3E3 2SP3E3 2SP3E3 | 1SP3E3- 1SP3E3- 2SP3E3+ |  |  |  | 1GP3E3 1GP3E3 1GP3E3 |  |
|  |  |  |  | Evidence |  | 4 4 4 | 4 4 4 |  |  |  | 4 4 4 |  |
|  |  |  |  | Focus |  | 5 4 4 | 4 4 4 |  |  |  | 1 1 1 |  |
|  |  |  |  | Polarity |  | 7 7 7 | 7 7 7 |  |  |  | 7 7 7 |  |
| 68 | 68 | 11518719\_4 | There is a high likelihood that members of this family, such as NAGABA and NAAla, share similar mechanisms of action, in particular with respect to the biosynthetic and degradatory pathways. |I:\*\*1SP2E0| |L:\*\*1SP2E0| |T:\*\*1SP2E1| |
|  |  |  |  | Annotation |  | 1SP2E0 | 1SP2E0 |  |  |  | 1SP2E1 |  |
|  |  |  |  | Evidence |  | 1 | 1 |  |  |  | 2 |  |
|  |  |  |  | Focus |  | 4 | 4 |  |  |  | 4 |  |
|  |  |  |  | Polarity |  | 6 | 6 |  |  |  | 6 |  |
| 69 | 69 | 8996225\_25 | Recent studies have demonstrated the involvement of alpha-bungarotoxin-sensitive nAChRs in ( )-nicotine-evoked Ca++ influx in ciliary ganglion neurons (Vijayaraghavan et al., 1992 ; Zhang et al., 1994 ), hippocampal neurons (Alkondon and Albuquerque, 1993 ) and at the synaptic junctions of the medial habenula and interpeduncular nuclei (McGehee et al., 1995 ). |I:\*\*1SP3E2| |L:\*\*1SP3E2| |T:\*\*1SP3E2| |
|  |  |  |  | Annotation |  | 1SP3E2 | 1SP3E2 |  |  |  | 1SP3E2 |  |
|  |  |  |  | Evidence |  | 3 | 3 |  |  |  | 3 |  |
|  |  |  |  | Focus |  | 4 | 4 |  |  |  | 4 |  |
|  |  |  |  | Polarity |  | 7 | 7 |  |  |  | 7 |  |
| 70 | 70 | 12860969\_315 | The TUNEL-positive cells (green) were distinguished from autofluorescent macrophages (red) through the use of a merge red/green filter. |I:\*\*1MP3E3| |L:\*\*1MP3E3| |T:\*\*1MP3E3| |
|  |  |  |  | Annotation |  | 1MP3E3 | 1MP3E3 |  |  |  | 1MP3E3 |  |
|  |  |  |  | Evidence |  | 4 | 4 |  |  |  | 4 |  |
|  |  |  |  | Focus |  | 2 | 2 |  |  |  | 2 |  |
|  |  |  |  | Polarity |  | 7 | 7 |  |  |  | 7 |  |
| 71 | 71 | 10671522\_64 | The majority of the PC12 cells show only a diffuse background staining that is similar to the level of staining observed with preimmune antiserum. |I:\*\*1SP3E0| |L:\*\*1SMP3E3| |T:\*\*1SP3E3| |
|  |  |  |  | Annotation |  | 1SP3E0 | 1SMP3E3 |  |  |  | 1SP3E3 |  |
|  |  |  |  | Evidence |  | 1 | 4 |  |  |  | 4 |  |
|  |  |  |  | Focus |  | 4 | 6 |  |  |  | 4 |  |
|  |  |  |  | Polarity |  | 7 | 7 |  |  |  | 7 |  |
| 72 | 72 | 9799801\_195 | sPLA 2 did not attack triacylglycerols |I:\*\*1SN3E0| |L:\*\*1SN3E3| and in this respect differed from hepatic and lipoprotein lipases |T:\*\*1SN3E1| which attack both glycerophospholipids and triacylglycerols. |T:\*\*2SP3E1| |I:\*\*2SP3E0| |L:\*\*2SP3E3| |
|  |  |  |  | Annotation |  | 1SN3E0 2SP3E0 2SP3E0 | 1SN3E3 2SP3E3 2SP3E3 |  |  |  | 1SN3E1 1SN3E1 2SP3E1 |  |
|  |  |  |  | Evidence |  | 1 1 1 | 4 4 4 |  |  |  | 2 2 2 |  |
|  |  |  |  | Focus |  | 4 4 4 | 4 4 4 |  |  |  | 4 4 4 |  |
|  |  |  |  | Polarity |  | 1 7 7 | 1 7 7 |  |  |  | 1 1 7 |  |
| 73 | 73 | 10757778\_289 | Whether r is large or small, the reduction in mean fitness from 1 is thus still of the order of mu. |I:\*\*1SP3E0-| |L:\*\*1SP3E0-| |T:\*\*1SP3E3| |
|  |  |  |  | Annotation |  | 1SP3E0- | 1SP3E0- |  |  |  | 1SP3E3 |  |
|  |  |  |  | Evidence |  | 1 | 1 |  |  |  | 4 |  |
|  |  |  |  | Focus |  | 4 | 4 |  |  |  | 4 |  |
|  |  |  |  | Polarity |  | 7 | 7 |  |  |  | 7 |  |
| 74 | 74 | 11756460\_9 | It has been suggested that the two chemically distinct motifs recognize different surfaces on the clathrin amino-terminal domain ( ). |I:\*\*1SP1E2| |L:\*\*1SP1E2| |T:\*\*1SP3E2| |
|  |  |  |  | Annotation |  | 1SP1E2 | 1SP1E2 |  |  |  | 1SP3E2 |  |
|  |  |  |  | Evidence |  | 3 | 3 |  |  |  | 3 |  |
|  |  |  |  | Focus |  | 4 | 4 |  |  |  | 4 |  |
|  |  |  |  | Polarity |  | 5 | 5 |  |  |  | 7 |  |
| 75 | 75 | 9566975\_857 | Wnt signaling polarizes an early C. elegans blastomore to distinguish endoderm from mesoderm. |I:\*\*1SP3E0| |L:\*\*1GSP3E0| |T:\*\*1SP3E1| |
|  |  |  |  | Annotation |  | 1SP3E0 | 1GSP3E0 |  |  |  | 1SP3E1 |  |
|  |  |  |  | Evidence |  | 1 | 1 |  |  |  | 2 |  |
|  |  |  |  | Focus |  | 4 | 5 |  |  |  | 4 |  |
|  |  |  |  | Polarity |  | 7 | 7 |  |  |  | 7 |  |
| 76 | 76 | 9725910\_384 | ANPK may also be involved in stabilization of the interaction between amino- and carboxyl-terminal regions of AR, |I:\*\*1SP1E0| an event known to be influenced by some other coregulatory proteins (Ikonen et al., 1997 ). |I:\*\*2SP3E2| |L:\*\*1SP1E2| |T:\*\*1SP1E2| |
|  |  |  |  | Annotation |  | 1SP1E0 2SP3E2 | 1SP1E2 1SP1E2 |  |  |  | 1SP1E2 1SP1E2 |  |
|  |  |  |  | Evidence |  | 1 3 | 3 3 |  |  |  | 3 3 |  |
|  |  |  |  | Focus |  | 4 4 | 4 4 |  |  |  | 4 4 |  |
|  |  |  |  | Polarity |  | 5 7 | 5 5 |  |  |  | 5 5 |  |
| 77 | 77 | 11124806\_42 | These data provide further evidence that regulation of chromatin structure is an important mechanism for the control of target gene expression by the Wg and Wnt pathways. |I:\*\*1SP3E0| |L:\*\*1SP2E3| |T:\*\*1SP3E1| |
|  |  |  |  | Annotation |  | 1SP3E0 | 1SP2E3 |  |  |  | 1SP3E1 |  |
|  |  |  |  | Evidence |  | 1 | 4 |  |  |  | 2 |  |
|  |  |  |  | Focus |  | 4 | 4 |  |  |  | 4 |  |
|  |  |  |  | Polarity |  | 7 | 6 |  |  |  | 7 |  |
| 78 | 78 | 11438668\_130 | The top panel shows the interaction between Smad3 and Axin. |I:\*\*1SGP3E0| |L:\*\*1SP3E3| |T:\*\*1SP3E3| |
|  |  |  |  | Annotation |  | 1SGP3E0 | 1SP3E3 |  |  |  | 1SP3E3 |  |
|  |  |  |  | Evidence |  | 1 | 4 |  |  |  | 4 |  |
|  |  |  |  | Focus |  | 5 | 4 |  |  |  | 4 |  |
|  |  |  |  | Polarity |  | 7 | 7 |  |  |  | 7 |  |
| 79 | 79 | 9407023\_209 | The genetic evidence |I:\*\*1SP3E3| that Wnt proteins require Fz proteins for signaling, |I:\*\*2SP0E3| although lacking in flies, |I:\*\*3SN3E3| |L:\*\*1SP3E3-| is accumulating in C. elegans (Table 3), |I:\*\*4SP3E3+| though the story is a little complicated. |I:\*\*5SGP3E3| |L:\*\*2SP3E3+| |T:\*\*1SP2E3| |
|  |  |  |  | Annotation |  | 1SP3E3 2SP0E3 3SN3E3 4SP3E3+ 5SGP3E3 | 1SP3E3- 1SP3E3- 1SP3E3- 2SP3E3+ 2SP3E3+ |  |  |  | 1SP2E3 1SP2E3 1SP2E3 1SP2E3 1SP2E3 |  |
|  |  |  |  | Evidence |  | 4 4 4 4 4 | 4 4 4 4 4 |  |  |  | 4 4 4 4 4 |  |
|  |  |  |  | Focus |  | 4 4 4 4 5 | 4 4 4 4 4 |  |  |  | 4 4 4 4 4 |  |
|  |  |  |  | Polarity |  | 7 4 1 7 7 | 7 7 7 7 7 |  |  |  | 6 6 6 6 6 |  |
| 80 | 80 | 12438416\_331 | Reactions were performed in buffer A (100 mM Hepes, pH 7.0, 10% sucrose, 0.1% CHAPS, 10 mM DTT) for caspases 3, 7, and 8, or buffer B (100 mM Mes, pH 6.5, 10% sucrose, 0.1% CHAPS, 10 mM DTT) for caspase-2. |I:\*\*1MP3E0| |L:\*\*1MP3E3| |T:\*\*1MP3E3| |
|  |  |  |  | Annotation |  | 1MP3E0 | 1MP3E3 |  |  |  | 1MP3E3 |  |
|  |  |  |  | Evidence |  | 1 | 4 |  |  |  | 4 |  |
|  |  |  |  | Focus |  | 2 | 2 |  |  |  | 2 |  |
|  |  |  |  | Polarity |  | 7 | 7 |  |  |  | 7 |  |
| 81 | 81 | 9153218\_14 | We found that transcription of the rrn operon in intact spinach plants is regulated by usage of two promoters. |I:\*\*1SP3E3| |L:\*\*1SP3E3| |T:\*\*1SP3E1| |
|  |  |  |  | Annotation |  | 1SP3E3 | 1SP3E3 |  |  |  | 1SP3E1 |  |
|  |  |  |  | Evidence |  | 4 | 4 |  |  |  | 2 |  |
|  |  |  |  | Focus |  | 4 | 4 |  |  |  | 4 |  |
|  |  |  |  | Polarity |  | 7 | 7 |  |  |  | 7 |  |
| 82 | 82 | 11282620\_143 | However, the concentrations of these heavy metals in the oily sludge and in the soil were much lower according to standards, |I:\*\*1SP3E0| |L:\*\*1SP3E0| |T:\*\*1SP3E1| and these concentrations were not toxic to most soil bacteria. |I:\*\*2SN3E0| |L:\*\*2SN3E0| |T:\*\*2SN3E1| |
|  |  |  |  | Annotation |  | 1SP3E0 2SN3E0 | 1SP3E0 2SN3E0 |  |  |  | 1SP3E1 2SN3E1 |  |
|  |  |  |  | Evidence |  | 1 1 | 1 1 |  |  |  | 2 2 |  |
|  |  |  |  | Focus |  | 4 4 | 4 4 |  |  |  | 4 4 |  |
|  |  |  |  | Polarity |  | 7 1 | 7 1 |  |  |  | 7 1 |  |
| 83 | 83 | 10555146\_242 | These data suggest that the majority of calcium changes and ERK activation are mediated by the Gi-coupled SPP receptor EDG-1. |I:\*\*1SP1E0| |L:\*\*1SP1E3| |T:\*\*1SP2E1| |
|  |  |  |  | Annotation |  | 1SP1E0 | 1SP1E3 |  |  |  | 1SP2E1 |  |
|  |  |  |  | Evidence |  | 1 | 4 |  |  |  | 2 |  |
|  |  |  |  | Focus |  | 4 | 4 |  |  |  | 4 |  |
|  |  |  |  | Polarity |  | 5 | 5 |  |  |  | 6 |  |
| 84 | 84 | 12137974\_7 | study suggests a possible regulatory effect of the sexual hormones on AM production by the ovary during the ovulatory process. |I:\*\*ERROR| |L:\*\*1SP1E0| |T:\*\*1SP1E1| |
|  |  |  |  | Annotation |  | ERROR | 1SP1E0 |  |  |  | 1SP1E1 |  |
|  |  |  |  | Evidence |  | -1000 | 1 |  |  |  | 2 |  |
|  |  |  |  | Focus |  | -1000 | 4 |  |  |  | 4 |  |
|  |  |  |  | Polarity |  | -1000 | 5 |  |  |  | 5 |  |
| 85 | 85 | 11112790\_127 | The induction was partially reduced |I:\*\*1SP3E3-| when the two motifs, CCAAT and CCACA, of ERSE were disrupted |L:\*\*1SP3E3-| ( line 2), |I:\*\*2SP3E3| indicating both that the effect of ATF6(366) is dependent on the cis-element and that other elements are involved in this induction. |I:\*\*3SP1E3| |L:\*\*2SP1E3+| |T:\*\*1SP2E3-| |
|  |  |  |  | Annotation |  | 1SP3E3- 2SP3E3 2SP3E3 3SP1E3 | 1SP3E3- 1SP3E3- 2SP1E3+ 2SP1E3+ |  |  |  | 1SP2E3- 1SP2E3- 1SP2E3- 1SP2E3- |  |
|  |  |  |  | Evidence |  | 4 4 4 4 | 4 4 4 4 |  |  |  | 4 4 4 4 |  |
|  |  |  |  | Focus |  | 4 4 4 4 | 4 4 4 4 |  |  |  | 4 4 4 4 |  |
|  |  |  |  | Polarity |  | 7 7 7 5 | 7 7 5 5 |  |  |  | 6 6 6 6 |  |
| 86 | 86 | 9927446\_257 | They hypothesized that the inviability of pol3-01 rad27delta mutants was not due to mutational load |I:\*\*1SN1E1| because diploids homozygous for both mutations were also inviable. |I:\*\*2SP3E1| |L:\*\*1SN1E1| |T:\*\*1SN2E0| |
|  |  |  |  | Annotation |  | 1SN1E1 2SP3E1 | 1SN1E1 1SN1E1 |  |  |  | 1SN2E0 1SN2E0 |  |
|  |  |  |  | Evidence |  | 2 2 | 2 2 |  |  |  | 1 1 |  |
|  |  |  |  | Focus |  | 4 4 | 4 4 |  |  |  | 4 4 |  |
|  |  |  |  | Polarity |  | 3 7 | 3 3 |  |  |  | 2 2 |  |
| 87 | 87 | 10454564\_97 | The pdelta10Not reporter plasmid used in these studies contains a partial cDNA sequence of the C. fasciculata homolog of the Trypanosoma cruzi flagellar calcium binding protein (CaBP) as a reporter ( 8). |I:\*\*1MP3E2| |L:\*\*1MP3E2| |T:\*\*1MP3E2| |
|  |  |  |  | Annotation |  | 1MP3E2 | 1MP3E2 |  |  |  | 1MP3E2 |  |
|  |  |  |  | Evidence |  | 3 | 3 |  |  |  | 3 |  |
|  |  |  |  | Focus |  | 2 | 2 |  |  |  | 2 |  |
|  |  |  |  | Polarity |  | 7 | 7 |  |  |  | 7 |  |
| 88 | 88 | 9628893\_63 | Subsequent to binding, mitochondria were separated from unbound actin filaments by centrifugation through a sucrose cushion (25% sucrose, 20 mM Hepes-KOH, pH 7.4, 1 mM PMSF, and protease inhibitor cocktail) at 12,500 g for 10 min. |I:\*\*1MP3E0| |L:\*\*1MP3E3| |T:\*\*1MP3E3| |
|  |  |  |  | Annotation |  | 1MP3E0 | 1MP3E3 |  |  |  | 1MP3E3 |  |
|  |  |  |  | Evidence |  | 1 | 4 |  |  |  | 4 |  |
|  |  |  |  | Focus |  | 2 | 2 |  |  |  | 2 |  |
|  |  |  |  | Polarity |  | 7 | 7 |  |  |  | 7 |  |
| 89 | 89 | 9870975\_65 | After completion of post-fixation, brains were embedded in gelatin and cryoprotected in a 20% sucrose/formalin solution for 3-4 d. |I:\*\*1MP3E0| |L:\*\*1MP3E3| |T:\*\*1MP3E3| |
|  |  |  |  | Annotation |  | 1MP3E0 | 1MP3E3 |  |  |  | 1MP3E3 |  |
|  |  |  |  | Evidence |  | 1 | 4 |  |  |  | 4 |  |
|  |  |  |  | Focus |  | 2 | 2 |  |  |  | 2 |  |
|  |  |  |  | Polarity |  | 7 | 7 |  |  |  | 7 |  |
| 90 | 90 | 11507088\_12 | Previous reports ( ) showed that inhibition of hsp90 binding leads to destabilization of mutant p53. |I:\*\*1SP3E2| |L:\*\*1SP3E2-| |T:\*\*1SP3E2-| |
|  |  |  |  | Annotation |  | 1SP3E2 | 1SP3E2- |  |  |  | 1SP3E2- |  |
|  |  |  |  | Evidence |  | 3 | 3 |  |  |  | 3 |  |
|  |  |  |  | Focus |  | 4 | 4 |  |  |  | 4 |  |
|  |  |  |  | Polarity |  | 7 | 7 |  |  |  | 7 |  |
| 91 | 91 | 11086983\_283 | The essence of our model incorporates |I:\*\*1SGP3E3| the now widely accepted premise of polygenic complexity of schizophrenia (with a spotlight on PSYN genes), which in turn produces maladaptive responses of developing neural circuitry in an experience-dependent fashion, ultimately resulting in clinical manifestations with a common set of phenotypes. |I:\*\*2SP2E0| |L:\*\*1MP3E3| |T:\*\*1SP3E0| |
|  |  |  |  | Annotation |  | 1SGP3E3 2SP2E0 | 1MP3E3 1MP3E3 |  |  |  | 1SP3E0 1SP3E0 |  |
|  |  |  |  | Evidence |  | 4 1 | 4 4 |  |  |  | 1 1 |  |
|  |  |  |  | Focus |  | 5 4 | 2 2 |  |  |  | 4 4 |  |
|  |  |  |  | Polarity |  | 7 6 | 7 7 |  |  |  | 7 7 |  |
| 92 | 92 | 12750351\_340 | The program used for simulation can be obtained from http://www.uvigo. |I:\*\*1GP3E3| |L:\*\*1GP3E3| |T:\*\*1GP3E3| |
|  |  |  |  | Annotation |  | 1GP3E3 | 1GP3E3 |  |  |  | 1GP3E3 |  |
|  |  |  |  | Evidence |  | 4 | 4 |  |  |  | 4 |  |
|  |  |  |  | Focus |  | 1 | 1 |  |  |  | 1 |  |
|  |  |  |  | Polarity |  | 7 | 7 |  |  |  | 7 |  |
| 93 | 93 | 12163473\_214 | We are most grateful to Dr. Stephen Lee for providing us with the Flag-GFP-NES and Flag-GFP-NLS expression vectors, to Dr. Joan S. Brugge for the chicken c-src (K295R), and to Dr. Y. Lazebnik for the dominant-negative caspase-9 cDNA. |I:\*\*1GP3E3| |L:\*\*1GP3E3| |T:\*\*1GP3E3| |
|  |  |  |  | Annotation |  | 1GP3E3 | 1GP3E3 |  |  |  | 1GP3E3 |  |
|  |  |  |  | Evidence |  | 4 | 4 |  |  |  | 4 |  |
|  |  |  |  | Focus |  | 1 | 1 |  |  |  | 1 |  |
|  |  |  |  | Polarity |  | 7 | 7 |  |  |  | 7 |  |
| 94 | 94 | 9262333\_97 | The ED50 for DOI was estimated to be 12.8 nmoles, |I:\*\*1SP3E0| calculated as an internal ED50 (base line to maximal response). |I:\*\*2MP3E0| |L:\*\*1MP3E3| |T:\*\*1SP3E3| |
|  |  |  |  | Annotation |  | 1SP3E0 2MP3E0 | 1MP3E3 1MP3E3 |  |  |  | 1SP3E3 1SP3E3 |  |
|  |  |  |  | Evidence |  | 1 1 | 4 4 |  |  |  | 4 4 |  |
|  |  |  |  | Focus |  | 4 2 | 2 2 |  |  |  | 4 4 |  |
|  |  |  |  | Polarity |  | 7 7 | 7 7 |  |  |  | 7 7 |  |
| 95 | 95 | 12352631\_15 | Lastly, we have summarized data that R92Q and P46L, and probably as yet undiscovered substitutions, represent very low penetrance mutations |I:\*\*1SP3E3| that may play a much larger role in more broadly defined inflammatory diseases such as rheumatoid arthritis. |I:\*\*2SP1E3| |L:\*\*1SP2E3| |T:\*\*1SP2E1| |
|  |  |  |  | Annotation |  | 1SP3E3 2SP1E3 | 1SP2E3 1SP2E3 |  |  |  | 1SP2E1 1SP2E1 |  |
|  |  |  |  | Evidence |  | 4 4 | 4 4 |  |  |  | 2 2 |  |
|  |  |  |  | Focus |  | 4 4 | 4 4 |  |  |  | 4 4 |  |
|  |  |  |  | Polarity |  | 7 5 | 6 6 |  |  |  | 6 6 |  |
| 96 | 96 | 12109221\_3 | It has been shown that overexpression of TIMPs in tumors of various origins |L:\*\*1SP3E0+| leads to reduced tumor growth and formation of metastases. |L:\*\*2SP3E0-| |I:\*\*1SP3E1+| |T:\*\*1SP3E1-| |
|  |  |  |  | Annotation |  | 1SP3E1+ 1SP3E1+ | 1SP3E0+ 2SP3E0- |  |  |  | 1SP3E1- 1SP3E1- |  |
|  |  |  |  | Evidence |  | 2 2 | 1 1 |  |  |  | 2 2 |  |
|  |  |  |  | Focus |  | 4 4 | 4 4 |  |  |  | 4 4 |  |
|  |  |  |  | Polarity |  | 7 7 | 7 7 |  |  |  | 7 7 |  |
| 97 | 97 | 10102275\_203 | There is a poor structural correspondence between Kir 2.1 and KcsA in the residues at the M2/M2 intersubunit interface and the pore-lining residues. |I:\*\*1SP3E0| |L:\*\*1SP3E0| |T:\*\*1SP3E0| |
|  |  |  |  | Annotation |  | 1SP3E0 | 1SP3E0 |  |  |  | 1SP3E0 |  |
|  |  |  |  | Evidence |  | 1 | 1 |  |  |  | 1 |  |
|  |  |  |  | Focus |  | 4 | 4 |  |  |  | 4 |  |
|  |  |  |  | Polarity |  | 7 | 7 |  |  |  | 7 |  |
| 98 | 98 | 11722893\_77 | The 3A4M5NC was collected from reaction supernatants on a C18 35 cm3 solid-phase extraction cartridge (Millipore, Milford, Mass.) and eluted from the matrix with methanol-water (50:50). |I:\*\*1MP3E0| |L:\*\*1MP3E3| |T:\*\*1MP3E3| |
|  |  |  |  | Annotation |  | 1MP3E0 | 1MP3E3 |  |  |  | 1MP3E3 |  |
|  |  |  |  | Evidence |  | 1 | 4 |  |  |  | 4 |  |
|  |  |  |  | Focus |  | 2 | 2 |  |  |  | 2 |  |
|  |  |  |  | Polarity |  | 7 | 7 |  |  |  | 7 |  |
| 99 | 99 | 10816498\_165 | This adhesion is fixation sensitive and independent of both CD36 and modified band 3. |I:\*\*1SP3E0| |L:\*\*1SP3E0| |T:\*\*1SP3E0| |
|  |  |  |  | Annotation |  | 1SP3E0 | 1SP3E0 |  |  |  | 1SP3E0 |  |
|  |  |  |  | Evidence |  | 1 | 1 |  |  |  | 1 |  |
|  |  |  |  | Focus |  | 4 | 4 |  |  |  | 4 |  |
|  |  |  |  | Polarity |  | 7 | 7 |  |  |  | 7 |  |
| 100 | 100 | 10884290\_179 | The interaction with DMPC causes the four-helix bundle to open into a receptor-active conformation ( 16) ( 34). |I:\*\*1SP3E2| |L:\*\*1SP3E2| |T:\*\*1SP3E2| |
|  |  |  |  | Annotation |  | 1SP3E2 | 1SP3E2 |  |  |  | 1SP3E2 |  |
|  |  |  |  | Evidence |  | 3 | 3 |  |  |  | 3 |  |
|  |  |  |  | Focus |  | 4 | 4 |  |  |  | 4 |  |
|  |  |  |  | Polarity |  | 7 | 7 |  |  |  | 7 |  |
| 101 | 101 | 9694806\_211 | For unexplained reasons, we observed greater attenuation of the p21Cip1 response than that of Mdm2 in ARF-null cells |T:\*\*1SP3E3| |I:\*\*1SP3E3-| |L:\*\*1MSP3E3| implying that not all p53-responsive genes are equally affected by ARF loss. |T:\*\*2SP2E1| |I:\*\*2SN1E3| |L:\*\*2SN1E3-| |
|  |  |  |  | Annotation |  | 1SP3E3- 2SN1E3 | 1MSP3E3 2SN1E3- |  |  |  | 1SP3E3 2SP2E1 |  |
|  |  |  |  | Evidence |  | 4 4 | 4 4 |  |  |  | 4 2 |  |
|  |  |  |  | Focus |  | 4 4 | 6 4 |  |  |  | 4 4 |  |
|  |  |  |  | Polarity |  | 7 3 | 7 3 |  |  |  | 7 6 |  |
| 102 | 102 | 9671481\_288 | The Nut2p-HA protein complemented the inviability of a NUT2 deletion. |I:\*\*1SP3E0| |L:\*\*1SP3E0| |T:\*\*1SP3E1| |
|  |  |  |  | Annotation |  | 1SP3E0 | 1SP3E0 |  |  |  | 1SP3E1 |  |
|  |  |  |  | Evidence |  | 1 | 1 |  |  |  | 2 |  |
|  |  |  |  | Focus |  | 4 | 4 |  |  |  | 4 |  |
|  |  |  |  | Polarity |  | 7 | 7 |  |  |  | 7 |  |
| 103 | 103 | 12093119\_5 | In conclusion, these data suggest that LH (hCG) and FSH regulate steroidogenic enzymes such as 5alphaRed and P450(c17). |I:\*\*1SP1E3| |L:\*\*1SP1E0| |T:\*\*1SP1E1| |
|  |  |  |  | Annotation |  | 1SP1E3 | 1SP1E0 |  |  |  | 1SP1E1 |  |
|  |  |  |  | Evidence |  | 4 | 1 |  |  |  | 2 |  |
|  |  |  |  | Focus |  | 4 | 4 |  |  |  | 4 |  |
|  |  |  |  | Polarity |  | 5 | 5 |  |  |  | 5 |  |
| 104 | 104 | 9421463\_58 | Plates were centrifuged briefly, and target/effector cells were incubated for 18 h. |I:\*\*1MP3E0| |L:\*\*1MP3E3| |T:\*\*1MP3E3| |
|  |  |  |  | Annotation |  | 1MP3E0 | 1MP3E3 |  |  |  | 1MP3E3 |  |
|  |  |  |  | Evidence |  | 1 | 4 |  |  |  | 4 |  |
|  |  |  |  | Focus |  | 2 | 2 |  |  |  | 2 |  |
|  |  |  |  | Polarity |  | 7 | 7 |  |  |  | 7 |  |
| 105 | 105 | 10102266\_220 | Evidence for an autocrine effect on axon elongation has also recently been provided in the case of hepatocyte growth factor (HGF) effects on sympathetic axons (Yang et al., 1998 ). |I:\*\*1SP3E2| |L:\*\*1SP3E2| |T:\*\*1SP3E2| |
|  |  |  |  | Annotation |  | 1SP3E2 | 1SP3E2 |  |  |  | 1SP3E2 |  |
|  |  |  |  | Evidence |  | 3 | 3 |  |  |  | 3 |  |
|  |  |  |  | Focus |  | 4 | 4 |  |  |  | 4 |  |
|  |  |  |  | Polarity |  | 7 | 7 |  |  |  | 7 |  |
| 106 | 106 | 12586067\_193 | However, we have so far been unable to expand purified hematopoietic stem cells (data not shown), |I:\*\*1SN3E3| so the exact nature of the FGF-1 target cell remains elusive. |I:\*\*2SP3E3| |L:\*\*1GN3E1| |T:\*\*1SN3E0| |
|  |  |  |  | Annotation |  | 1SN3E3 2SP3E3 | 1GN3E1 1GN3E1 |  |  |  | 1SN3E0 1SN3E0 |  |
|  |  |  |  | Evidence |  | 4 4 | 2 2 |  |  |  | 1 1 |  |
|  |  |  |  | Focus |  | 4 4 | 1 1 |  |  |  | 4 4 |  |
|  |  |  |  | Polarity |  | 1 7 | 1 1 |  |  |  | 1 1 |  |
| 107 | 107 | 12837387\_87 | In addition to these core domains, an epimerization (E) domain is usually present in those modules that convert an L amino acid into the D form. |I:\*\*1SP3E0| |L:\*\*1GSP3E0| |T:\*\*1SP3E0| |
|  |  |  |  | Annotation |  | 1SP3E0 | 1GSP3E0 |  |  |  | 1SP3E0 |  |
|  |  |  |  | Evidence |  | 1 | 1 |  |  |  | 1 |  |
|  |  |  |  | Focus |  | 4 | 5 |  |  |  | 4 |  |
|  |  |  |  | Polarity |  | 7 | 7 |  |  |  | 7 |  |
| 108 | 108 | 9870960\_70 | In addition, a second group of controls ( n = 13), |I:\*\*1SGP3E3| not rendered anosmic and therefore able to use their navigational map based on atmospheric odors, |I:\*\*2SP3E3| was included |I:\*\*3SGP3E3| to determine whether the behavioral differences observed in series 1 was a consequence of the two groups responding differently to the phase-shift manipulation (see below). |I:\*\*4SGP0E3| |L:\*\*1MP3E3| |T:\*\*1MP3E3| |
|  |  |  |  | Annotation |  | 1SGP3E3 2SP3E3 3SGP3E3 4SGP0E3 | 1MP3E3 1MP3E3 1MP3E3 1MP3E3 |  |  |  | 1MP3E3 1MP3E3 1MP3E3 1MP3E3 |  |
|  |  |  |  | Evidence |  | 4 4 4 4 | 4 4 4 4 |  |  |  | 4 4 4 4 |  |
|  |  |  |  | Focus |  | 5 4 5 5 | 2 2 2 2 |  |  |  | 2 2 2 2 |  |
|  |  |  |  | Polarity |  | 7 7 7 4 | 7 7 7 7 |  |  |  | 7 7 7 7 |  |
| 109 | 109 | 10230791\_358 | Ptp69D alleles are as described in Desai et al. 1996 . |I:\*\*1MP3E3| |L:\*\*1SP3E2| |T:\*\*1SP3E2| |
|  |  |  |  | Annotation |  | 1MP3E3 | 1SP3E2 |  |  |  | 1SP3E2 |  |
|  |  |  |  | Evidence |  | 4 | 3 |  |  |  | 3 |  |
|  |  |  |  | Focus |  | 2 | 4 |  |  |  | 4 |  |
|  |  |  |  | Polarity |  | 7 | 7 |  |  |  | 7 |  |
| 110 | 110 | 10652304\_62 | DNA fragments are identified by letter and sizes (base pairs) are shown. |I:\*\*1SGP3E3| |L:\*\*1MP3E3| |T:\*\*1SP3E3| |
|  |  |  |  | Annotation |  | 1SGP3E3 | 1MP3E3 |  |  |  | 1SP3E3 |  |
|  |  |  |  | Evidence |  | 4 | 4 |  |  |  | 4 |  |
|  |  |  |  | Focus |  | 5 | 2 |  |  |  | 4 |  |
|  |  |  |  | Polarity |  | 7 | 7 |  |  |  | 7 |  |
| 111 | 111 | 12691662\_227 | Caspase inhibitors block NMDA-induced neuronal apoptosis (Okamoto et al., 2002 ). |I:\*\*1SP3E2| |L:\*\*1SP3E2-| |T:\*\*1SN3E2| |
|  |  |  |  | Annotation |  | 1SP3E2 | 1SP3E2- |  |  |  | 1SN3E2 |  |
|  |  |  |  | Evidence |  | 3 | 3 |  |  |  | 3 |  |
|  |  |  |  | Focus |  | 4 | 4 |  |  |  | 4 |  |
|  |  |  |  | Polarity |  | 7 | 7 |  |  |  | 1 |  |
| 112 | 112 | 12360142\_5 | RESULTS: The cesarean section rate was 26.4% in this period. |I:\*\*1SP3E3| |L:\*\*1SP3E3| |T:\*\*1SP3E1| |
|  |  |  |  | Annotation |  | 1SP3E3 | 1SP3E3 |  |  |  | 1SP3E1 |  |
|  |  |  |  | Evidence |  | 4 | 4 |  |  |  | 2 |  |
|  |  |  |  | Focus |  | 4 | 4 |  |  |  | 4 |  |
|  |  |  |  | Polarity |  | 7 | 7 |  |  |  | 7 |  |
| 113 | 113 | 10757779\_297 | Nevertheless, C0 is still used as an estimator for R( T) for all figures |I:\*\*1MSP3E3| (except Fig 14), |I:\*\*2MSN3E3| because C0 C0 + sigma100i=1 Ci. |I:\*\*3MSP3E3| |L:\*\*1MP3E3| |T:\*\*1SP3E3| |
|  |  |  |  | Annotation |  | 1MSP3E3 2MSN3E3 3MSP3E3 | 1MP3E3 1MP3E3 1MP3E3 |  |  |  | 1SP3E3 1SP3E3 1SP3E3 |  |
|  |  |  |  | Evidence |  | 4 4 4 | 4 4 4 |  |  |  | 4 4 4 |  |
|  |  |  |  | Focus |  | 6 6 6 | 2 2 2 |  |  |  | 4 4 4 |  |
|  |  |  |  | Polarity |  | 7 1 7 | 7 7 7 |  |  |  | 7 7 7 |  |
| 114 | 114 | 10894724\_84 | Purified nrfA-acs intergenic region fragments were end labeled with [gamma-32]ATP, and 2.5 to 0.5 ng of each fragment was incubated with various amounts of purified FNR DA154. |I:\*\*1MP3E0| |L:\*\*1MP3E3| |T:\*\*1MP3E3| |
|  |  |  |  | Annotation |  | 1MP3E0 | 1MP3E3 |  |  |  | 1MP3E3 |  |
|  |  |  |  | Evidence |  | 1 | 4 |  |  |  | 4 |  |
|  |  |  |  | Focus |  | 2 | 2 |  |  |  | 2 |  |
|  |  |  |  | Polarity |  | 7 | 7 |  |  |  | 7 |  |
| 115 | 115 | 9466757\_78 | For each dilution, 25 mul was plated onto Columbia blood agar plates and the plates were incubated overnight at 37 degrees C in 5% CO2. |I:\*\*1MP3E0| |L:\*\*1MP3E3| |T:\*\*1MP3E3| |
|  |  |  |  | Annotation |  | 1MP3E0 | 1MP3E3 |  |  |  | 1MP3E3 |  |
|  |  |  |  | Evidence |  | 1 | 4 |  |  |  | 4 |  |
|  |  |  |  | Focus |  | 2 | 2 |  |  |  | 2 |  |
|  |  |  |  | Polarity |  | 7 | 7 |  |  |  | 7 |  |
| 116 | 116 | 12524347\_96 | DNA was purified by phenol-chloroform extraction and recovered by ethanol precipitation. |I:\*\*1MP3E0| |L:\*\*1MP3E3| |T:\*\*1MP3E3| |
|  |  |  |  | Annotation |  | 1MP3E0 | 1MP3E3 |  |  |  | 1MP3E3 |  |
|  |  |  |  | Evidence |  | 1 | 4 |  |  |  | 4 |  |
|  |  |  |  | Focus |  | 2 | 2 |  |  |  | 2 |  |
|  |  |  |  | Polarity |  | 7 | 7 |  |  |  | 7 |  |
| 117 | 117 | 11703941\_86 | This type of binding does not occur in imd1 mutant flies (Lemaitre et al., 1995 ). |I:\*\*1SN3E2| |L:\*\*1SN3E2| |T:\*\*1SP3E2| |
|  |  |  |  | Annotation |  | 1SN3E2 | 1SN3E2 |  |  |  | 1SP3E2 |  |
|  |  |  |  | Evidence |  | 3 | 3 |  |  |  | 3 |  |
|  |  |  |  | Focus |  | 4 | 4 |  |  |  | 4 |  |
|  |  |  |  | Polarity |  | 1 | 1 |  |  |  | 7 |  |
| 118 | 118 | 12551933\_45 | We demonstrate that nCLU has one high affinity coiled-coil Ku70 binding domain within its C terminus. |I:\*\*1SP3E3| |L:\*\*1SP3E3| |T:\*\*1SP3E3| |
|  |  |  |  | Annotation |  | 1SP3E3 | 1SP3E3 |  |  |  | 1SP3E3 |  |
|  |  |  |  | Evidence |  | 4 | 4 |  |  |  | 4 |  |
|  |  |  |  | Focus |  | 4 | 4 |  |  |  | 4 |  |
|  |  |  |  | Polarity |  | 7 | 7 |  |  |  | 7 |  |
| 119 | 119 | 11395478\_25 | This frameshift mutation is predicted to generate a truncated SKCa3 polypeptide, SKCa3delta, |I:\*\*1SP1E0| that ends prematurely at a stop codon after residue 286. |I:\*\*2SP3E0| |L:\*\*1SP1E0| |T:\*\*1SP3E3| |
|  |  |  |  | Annotation |  | 1SP1E0 2SP3E0 | 1SP1E0 1SP1E0 |  |  |  | 1SP3E3 1SP3E3 |  |
|  |  |  |  | Evidence |  | 1 1 | 1 1 |  |  |  | 4 4 |  |
|  |  |  |  | Focus |  | 4 4 | 4 4 |  |  |  | 4 4 |  |
|  |  |  |  | Polarity |  | 5 7 | 5 5 |  |  |  | 7 7 |  |
| 120 | 120 | 10200329\_26 | If one assumes that a schizophrenia disposition is genetically transmitted as a dominant trait, |I:\*\*1SP0E3| then about half of these individuals, |I:\*\*2SP2E3| although clinically unaffected by the illness and therefore untreated by antipsychotic medications, |I:\*\*3SN3E3| would be presumed to share a genetic predisposition for schizophrenia. |I:\*\*4SP2E3| |L:\*\*1GP1E0| |T:\*\*1SP1E1| |
|  |  |  |  | Annotation |  | 1SP0E3 2SP2E3 3SN3E3 4SP2E3 | 1GP1E0 1GP1E0 1GP1E0 1GP1E0 |  |  |  | 1SP1E1 1SP1E1 1SP1E1 1SP1E1 |  |
|  |  |  |  | Evidence |  | 4 4 4 4 | 1 1 1 1 |  |  |  | 2 2 2 2 |  |
|  |  |  |  | Focus |  | 4 4 4 4 | 1 1 1 1 |  |  |  | 4 4 4 4 |  |
|  |  |  |  | Polarity |  | 4 6 1 6 | 5 5 5 5 |  |  |  | 5 5 5 5 |  |
| 121 | 121 | 11964912\_10 | The value of adjunctive treatments, such as granulocyte colony-stimulating factor, warrants further evaluation. |I:\*\*1SGP0E3| |L:\*\*1GP3E0| |T:\*\*1SP3E0| |
|  |  |  |  | Annotation |  | 1SGP0E3 | 1GP3E0 |  |  |  | 1SP3E0 |  |
|  |  |  |  | Evidence |  | 4 | 1 |  |  |  | 1 |  |
|  |  |  |  | Focus |  | 5 | 1 |  |  |  | 4 |  |
|  |  |  |  | Polarity |  | 4 | 7 |  |  |  | 7 |  |
| 122 | 122 | 9407036\_28 | Additionally, maximal induction of the PMR2A/ENA1 gene in response to high environmental salt requires calcineurin activation by Ca2+/calmodulin (Garciadeblas et al. 1993 ; Cunningham and Fink 1996 ). |I:\*\*1SP3E2| |L:\*\*1SP3E2+| |T:\*\*1SP3E2| |
|  |  |  |  | Annotation |  | 1SP3E2 | 1SP3E2+ |  |  |  | 1SP3E2 |  |
|  |  |  |  | Evidence |  | 3 | 3 |  |  |  | 3 |  |
|  |  |  |  | Focus |  | 4 | 4 |  |  |  | 4 |  |
|  |  |  |  | Polarity |  | 7 | 7 |  |  |  | 7 |  |
| 123 | 123 | 12498718\_7 | Chromosomal translocations which fuse the Ewing's sarcoma gene EWS to different DNA binding domains result in the creation of chimeric transcription factors that define specific types of embryonal tumors. |I:\*\*1SP3E0| |L:\*\*1SP3E0| |T:\*\*1SP3E1| |
|  |  |  |  | Annotation |  | 1SP3E0 | 1SP3E0 |  |  |  | 1SP3E1 |  |
|  |  |  |  | Evidence |  | 1 | 1 |  |  |  | 2 |  |
|  |  |  |  | Focus |  | 4 | 4 |  |  |  | 4 |  |
|  |  |  |  | Polarity |  | 7 | 7 |  |  |  | 7 |  |
| 124 | 124 | 9463486\_36 | The TA-Cloning kit was purchased from InVitrogen (San Diego, CA). |I:\*\*1MP3E3| |L:\*\*1GMP3E3| |T:\*\*1MP3E1| |
|  |  |  |  | Annotation |  | 1MP3E3 | 1GMP3E3 |  |  |  | 1MP3E1 |  |
|  |  |  |  | Evidence |  | 4 | 4 |  |  |  | 2 |  |
|  |  |  |  | Focus |  | 2 | 3 |  |  |  | 2 |  |
|  |  |  |  | Polarity |  | 7 | 7 |  |  |  | 7 |  |
| 125 | 125 | 12369679\_10 | CONCLUSIONS AND CLINICAL RELEVANCE: The low specificity of WBT of CSF indicated |I:\*\*1SGP3E3| that it is inappropriate to diagnose EPM on the basis of a positive test result alone |I:\*\*2SN3E3| because of the possibility of false-positive test results. |I:\*\*3SP3E3| |L:\*\*1SN1E3| |T:\*\*1SN1E1| |
|  |  |  |  | Annotation |  | 1SGP3E3 2SN3E3 3SP3E3 | 1SN1E3 1SN1E3 1SN1E3 |  |  |  | 1SN1E1 1SN1E1 1SN1E1 |  |
|  |  |  |  | Evidence |  | 4 4 4 | 4 4 4 |  |  |  | 2 2 2 |  |
|  |  |  |  | Focus |  | 5 4 4 | 4 4 4 |  |  |  | 4 4 4 |  |
|  |  |  |  | Polarity |  | 7 1 7 | 3 3 3 |  |  |  | 3 3 3 |  |
| 126 | 126 | 10428961\_294 | Ikeda, S. , Kishida, S. , Yamamoto, H. , Murai, H. , Koyama, S. and Kikuchi, A. (1998) |L:\*\*1GP3E3| Axin, a negative regulator of the Wnt signaling pathway, |L:\*\*2SP3E3-| forms a complex with GSK-3-beta and beta-catenin and promotes GSK-3-beta-dependent phosphorylation of beta-catenin. |L:\*\*3SP3E3+| |I:\*\*1SP3E2| |T:\*\*1SP3E1| |
|  |  |  |  | Annotation |  | 1SP3E2 1SP3E2 1SP3E2 | 1GP3E3 2SP3E3- 3SP3E3+ |  |  |  | 1SP3E1 1SP3E1 1SP3E1 |  |
|  |  |  |  | Evidence |  | 3 3 3 | 4 4 4 |  |  |  | 2 2 2 |  |
|  |  |  |  | Focus |  | 4 4 4 | 1 4 4 |  |  |  | 4 4 4 |  |
|  |  |  |  | Polarity |  | 7 7 7 | 7 7 7 |  |  |  | 7 7 7 |  |
| 127 | 127 | 9450960\_252 | This Cdc25-S287A mutant can compromise the checkpoints involving unreplicated and damaged DNA. |I:\*\*1SP3E0| |L:\*\*1SP3E0| |T:\*\*1SP3E1| |
|  |  |  |  | Annotation |  | 1SP3E0 | 1SP3E0 |  |  |  | 1SP3E1 |  |
|  |  |  |  | Evidence |  | 1 | 1 |  |  |  | 2 |  |
|  |  |  |  | Focus |  | 4 | 4 |  |  |  | 4 |  |
|  |  |  |  | Polarity |  | 7 | 7 |  |  |  | 7 |  |
| 128 | 128 | 10655240\_284 | Also, the number of potential helices may be reduced |I:\*\*1SP3E0-| by requiring that at least one WC covariation be present in each helix. |I:\*\*2SP3E0| |L:\*\*1SP1E0-| |T:\*\*1SP1E1| |
|  |  |  |  | Annotation |  | 1SP3E0- 2SP3E0 | 1SP1E0- 1SP1E0- |  |  |  | 1SP1E1 1SP1E1 |  |
|  |  |  |  | Evidence |  | 1 1 | 1 1 |  |  |  | 2 2 |  |
|  |  |  |  | Focus |  | 4 4 | 4 4 |  |  |  | 4 4 |  |
|  |  |  |  | Polarity |  | 7 7 | 5 5 |  |  |  | 5 5 |  |
| 129 | 129 | 10896157\_17 | AMPA receptors have been proposed to be regulated by at least two distinct mechanisms during the expression of LTP/LTD |T:\*\*1SP3E1| |I:\*\*1SP1E1| |L:\*\*1SP3E2| Protein phosphorylation/dephosphorylation of AMPA receptors has long been thought to be a critical factor in the expression of LTP and LTD ( Raymond et al. 1993a ; Roche et al. 1996 ). |T:\*\*1SP2E2| |I:\*\*1SP2E2| |L:\*\*1SP1E2| |
|  |  |  |  | Annotation |  | 1SP1E1 1SP2E2 | 1SP3E2 1SP1E2 |  |  |  | 1SP3E1 1SP2E2 |  |
|  |  |  |  | Evidence |  | 2 3 | 3 3 |  |  |  | 2 3 |  |
|  |  |  |  | Focus |  | 4 4 | 4 4 |  |  |  | 4 4 |  |
|  |  |  |  | Polarity |  | 5 6 | 7 5 |  |  |  | 7 6 |  |
| 130 | 130 | 9315549\_11 | In-hospital mortality rates declined during the period from 1993 through 1995 |T:\*\*1SN3E1| |I:\*\*1SP3E0-| and were lower than predicted |T:\*\*2SP3E1-| |L:\*\*1SP3E0-| despite the increase in risk. |T:\*\*3SP3E1+| |I:\*\*2SP3E0+| |L:\*\*2SP3E0+| |
|  |  |  |  | Annotation |  | 1SP3E0- 2SP3E0+ 2SP3E0+ | 1SP3E0- 1SP3E0- 2SP3E0+ |  |  |  | 1SN3E1 2SP3E1- 3SP3E1+ |  |
|  |  |  |  | Evidence |  | 1 1 1 | 1 1 1 |  |  |  | 2 2 2 |  |
|  |  |  |  | Focus |  | 4 4 4 | 4 4 4 |  |  |  | 4 4 4 |  |
|  |  |  |  | Polarity |  | 7 7 7 | 7 7 7 |  |  |  | 1 7 7 |  |
| 131 | 131 | 11970895\_405 | Interaction of dishevelled and Xenopus axin-related protein is required for wnt signal transduction. |I:\*\*1SP3E0| |L:\*\*1SP3E0| |T:\*\*1SP3E1| |
|  |  |  |  | Annotation |  | 1SP3E0 | 1SP3E0 |  |  |  | 1SP3E1 |  |
|  |  |  |  | Evidence |  | 1 | 1 |  |  |  | 2 |  |
|  |  |  |  | Focus |  | 4 | 4 |  |  |  | 4 |  |
|  |  |  |  | Polarity |  | 7 | 7 |  |  |  | 7 |  |
| 132 | 132 | 11447157\_70 | The SNP approach is essentially a limited sequencing approach in that only one of the four dideoxynucleotide incorporation reactions is performed. |I:\*\*1SP3E0| |L:\*\*1GP3E0| |T:\*\*1MP3E0| |
|  |  |  |  | Annotation |  | 1SP3E0 | 1GP3E0 |  |  |  | 1MP3E0 |  |
|  |  |  |  | Evidence |  | 1 | 1 |  |  |  | 1 |  |
|  |  |  |  | Focus |  | 4 | 1 |  |  |  | 2 |  |
|  |  |  |  | Polarity |  | 7 | 7 |  |  |  | 7 |  |
| 133 | 133 | 12481028\_4 | This effect of L-methionine was associated with an increase in the number of methylated cytosines in the CpG island of the reelin promoter region. |I:\*\*1SP3E0+| |L:\*\*1SP3E0+| |T:\*\*1SP3E1+| |
|  |  |  |  | Annotation |  | 1SP3E0+ | 1SP3E0+ |  |  |  | 1SP3E1+ |  |
|  |  |  |  | Evidence |  | 1 | 1 |  |  |  | 2 |  |
|  |  |  |  | Focus |  | 4 | 4 |  |  |  | 4 |  |
|  |  |  |  | Polarity |  | 7 | 7 |  |  |  | 7 |  |
| 134 | 134 | 12351171\_146 | Thus, one explanation for the activity of Xbra3 in non-cell autonomous neural induction |I:\*\*1SP3E0| would be that Xbra3 activates production of a Wnt signal |T:\*\*1SP3E1+| |L:\*\*1SP1E0+| which inhibits, by one of these mechanisms, BMP signaling in the responding cap |T:\*\*2SN3E1| |L:\*\*2SP1E0-| leading to expression of neural markers. |T:\*\*3SP3E1| |I:\*\*2SP1E3| |L:\*\*3SP1E0+| |
|  |  |  |  | Annotation |  | 1SP3E0 2SP1E3 2SP1E3 2SP1E3 | 1SP1E0+ 1SP1E0+ 2SP1E0- 3SP1E0+ |  |  |  | 1SP3E1+ 1SP3E1+ 2SN3E1 3SP3E1 |  |
|  |  |  |  | Evidence |  | 1 4 4 4 | 1 1 1 1 |  |  |  | 2 2 2 2 |  |
|  |  |  |  | Focus |  | 4 4 4 4 | 4 4 4 4 |  |  |  | 4 4 4 4 |  |
|  |  |  |  | Polarity |  | 7 5 5 5 | 5 5 5 5 |  |  |  | 7 7 1 7 |  |
| 135 | 135 | 10446135\_126 | C, quantitation of AMT1- lacZ mRNA expression in response to deoxycorticosterone. |I:\*\*ERROR| |L:\*\*1GSP3E0| |T:\*\*1SP3E0| |
|  |  |  |  | Annotation |  | ERROR | 1GSP3E0 |  |  |  | 1SP3E0 |  |
|  |  |  |  | Evidence |  | -1000 | 1 |  |  |  | 1 |  |
|  |  |  |  | Focus |  | -1000 | 5 |  |  |  | 4 |  |
|  |  |  |  | Polarity |  | -1000 | 7 |  |  |  | 7 |  |
| 136 | 136 | 10617578\_2 | We generated renin-deficient mice |I:\*\*1SGP3E3| with targeted replacement of the Ren-1C loci by the lacZ gene in the TT2 ES cells derived from an F1 embryo between C57BL/6 and CBA mice ( ) (Fig. 1 A). |I:\*\*2MP3E3| |L:\*\*1MP3E3| |T:\*\*1MP3E3| |
|  |  |  |  | Annotation |  | 1SGP3E3 2MP3E3 | 1MP3E3 1MP3E3 |  |  |  | 1MP3E3 1MP3E3 |  |
|  |  |  |  | Evidence |  | 4 4 | 4 4 |  |  |  | 4 4 |  |
|  |  |  |  | Focus |  | 5 2 | 2 2 |  |  |  | 2 2 |  |
|  |  |  |  | Polarity |  | 7 7 | 7 7 |  |  |  | 7 7 |  |
| 137 | 137 | 12297496\_102 | E, apoptosis of DU-145 cells after transient transfection with the indicated expression plasmids. |I:\*\*ERROR| |L:\*\*1GSP3E0| |T:\*\*1SP3E0| |
|  |  |  |  | Annotation |  | ERROR | 1GSP3E0 |  |  |  | 1SP3E0 |  |
|  |  |  |  | Evidence |  | -1000 | 1 |  |  |  | 1 |  |
|  |  |  |  | Focus |  | -1000 | 5 |  |  |  | 4 |  |
|  |  |  |  | Polarity |  | -1000 | 7 |  |  |  | 7 |  |
| 138 | 138 | 9763432\_176 | It is interesting in this regard that the NH2 terminus, including the ART domain, is further exposed after membrane insertion of BAX in vivo (Fig. 2 B). |I:\*\*1SP3E3| |L:\*\*1SP3E3| |T:\*\*1SP3E3| |
|  |  |  |  | Annotation |  | 1SP3E3 | 1SP3E3 |  |  |  | 1SP3E3 |  |
|  |  |  |  | Evidence |  | 4 | 4 |  |  |  | 4 |  |
|  |  |  |  | Focus |  | 4 | 4 |  |  |  | 4 |  |
|  |  |  |  | Polarity |  | 7 | 7 |  |  |  | 7 |  |
| 139 | 139 | 12473690\_64 | (B) Quantitative analysis of the data from triplicate experiments including those in A. |I:\*\*ERROR| |L:\*\*ERROR| |T:\*\*ERROR| [35]atToc159 binding to chloroplasts (Binding) was measured as the amount of both full-length atToc159 (atToc159) and 86-kD fragment (159GM) that was associated |I:\*\*1MSP3E0| |L:\*\*1MP3E2| |T:\*\*1SP3E3| but not inserted into the outer membrane. |I:\*\*2MSN3E0| |L:\*\*2MN3E2| |T:\*\*2SN3E1| |
|  |  |  |  | Annotation |  | ERROR 1MSP3E0 2MSN3E0 | ERROR 1MP3E2 2MN3E2 |  |  |  | ERROR 1SP3E3 2SN3E1 |  |
|  |  |  |  | Evidence |  | -1000 1 1 | -1000 3 3 |  |  |  | -1000 4 2 |  |
|  |  |  |  | Focus |  | -1000 6 6 | -1000 2 2 |  |  |  | -1000 4 4 |  |
|  |  |  |  | Polarity |  | -1000 7 1 | -1000 7 1 |  |  |  | -1000 7 1 |  |
| 140 | 140 | 9099694\_25 | The amount of [32P]orthophosphate incorporated into ProTalpha after 20 h of metabolic labeling of mitogen-stimulated mouse splenocytes (in the conditions indicated in "Methods") was about 0.3 nmol/108 cells. |I:\*\*1SP3E3| |L:\*\*1SP3E3| |T:\*\*1SP3E3| |
|  |  |  |  | Annotation |  | 1SP3E3 | 1SP3E3 |  |  |  | 1SP3E3 |  |
|  |  |  |  | Evidence |  | 4 | 4 |  |  |  | 4 |  |
|  |  |  |  | Focus |  | 4 | 4 |  |  |  | 4 |  |
|  |  |  |  | Polarity |  | 7 | 7 |  |  |  | 7 |  |
| 141 | 141 | 10490650\_10 | In metazoans, Wnt signaling causes inhibition of GSK-3beta ( 3), |L:\*\*1SP3E2-| |T:\*\*1SN3E2| which in turn leads to stabilization of cytoplasmic beta-catenin (armadillo in Drosophila) and activation of Wnt target genes ( 2, 32). |L:\*\*2SP3E2+| |I:\*\*1SP3E2| |T:\*\*2SP3E2| |
|  |  |  |  | Annotation |  | 1SP3E2 1SP3E2 | 1SP3E2- 2SP3E2+ |  |  |  | 1SN3E2 2SP3E2 |  |
|  |  |  |  | Evidence |  | 3 3 | 3 3 |  |  |  | 3 3 |  |
|  |  |  |  | Focus |  | 4 4 | 4 4 |  |  |  | 4 4 |  |
|  |  |  |  | Polarity |  | 7 7 | 7 7 |  |  |  | 1 7 |  |
| 142 | 142 | 11566983\_218 | Previous studies suggested that sequence changes in vlsE accumulate over the duration of infection ( 20). |I:\*\*1SP1E2+| |L:\*\*1SP1E2| |T:\*\*1SP3E2| |
|  |  |  |  | Annotation |  | 1SP1E2+ | 1SP1E2 |  |  |  | 1SP3E2 |  |
|  |  |  |  | Evidence |  | 3 | 3 |  |  |  | 3 |  |
|  |  |  |  | Focus |  | 4 | 4 |  |  |  | 4 |  |
|  |  |  |  | Polarity |  | 5 | 5 |  |  |  | 7 |  |
| 143 | 143 | 9271353\_38 | CHO cell lines stably transfected with human NQO1 cDNA were developed as previously described ( 21). |I:\*\*1MP3E3| |L:\*\*1GMP3E2| |T:\*\*1MP3E2| |
|  |  |  |  | Annotation |  | 1MP3E3 | 1GMP3E2 |  |  |  | 1MP3E2 |  |
|  |  |  |  | Evidence |  | 4 | 3 |  |  |  | 3 |  |
|  |  |  |  | Focus |  | 2 | 3 |  |  |  | 2 |  |
|  |  |  |  | Polarity |  | 7 | 7 |  |  |  | 7 |  |
| 144 | 144 | 10866683\_56 | The construction of these linker scanners or deletions has been described previously ( 2). |I:\*\*1MGP3E3| |L:\*\*1GMP3E2| |T:\*\*1MP3E2| |
|  |  |  |  | Annotation |  | 1MGP3E3 | 1GMP3E2 |  |  |  | 1MP3E2 |  |
|  |  |  |  | Evidence |  | 4 | 3 |  |  |  | 3 |  |
|  |  |  |  | Focus |  | 3 | 3 |  |  |  | 2 |  |
|  |  |  |  | Polarity |  | 7 | 7 |  |  |  | 7 |  |
| 145 | 145 | 12446739\_133 | The average of at least three experiments plus-or-minus SD is shown for each supernatant fraction. |I:\*\*1SGP3E3| |L:\*\*1MP3E0| |T:\*\*1SP3E3| |
|  |  |  |  | Annotation |  | 1SGP3E3 | 1MP3E0 |  |  |  | 1SP3E3 |  |
|  |  |  |  | Evidence |  | 4 | 1 |  |  |  | 4 |  |
|  |  |  |  | Focus |  | 5 | 2 |  |  |  | 4 |  |
|  |  |  |  | Polarity |  | 7 | 7 |  |  |  | 7 |  |
| 146 | 146 | 12086863\_135 | Further evidence for a heterogeneous composition of glial tumors |I:\*\*1SGP3E0| was provided by immunohistochemical analysis. |I:\*\*2MP3E0| |L:\*\*1SP3E1| |T:\*\*1SP3E1| |
|  |  |  |  | Annotation |  | 1SGP3E0 2MP3E0 | 1SP3E1 1SP3E1 |  |  |  | 1SP3E1 1SP3E1 |  |
|  |  |  |  | Evidence |  | 1 1 | 2 2 |  |  |  | 2 2 |  |
|  |  |  |  | Focus |  | 5 2 | 4 4 |  |  |  | 4 4 |  |
|  |  |  |  | Polarity |  | 7 7 | 7 7 |  |  |  | 7 7 |  |
| 147 | 147 | 9693372\_6 | Mad forms a heteromeric complex with Drosophila Smad4 (Medea) upon phosphorylation by Thick veins (Tkv), a type I receptor for Dpp. |L:\*\*1SP3E0| |I:\*\*1SP3E0| |T:\*\*1SP3E0| Dad stably associates with Tkv |L:\*\*1SP3E0| and |T:\*\*1SP3E1| thereby inhibits Tkv-induced Mad phosphorylation. |L:\*\*2SP3E0-| |I:\*\*1SP3E0| |T:\*\*2SN3E1| |
|  |  |  |  | Annotation |  | 1SP3E0 1SP3E0 1SP3E0 1SP3E0 | 1SP3E0 1SP3E0 2SP3E0- 2SP3E0- |  |  |  | 1SP3E0 1SP3E1 1SP3E1 2SN3E1 |  |
|  |  |  |  | Evidence |  | 1 1 1 1 | 1 1 1 1 |  |  |  | 1 2 2 2 |  |
|  |  |  |  | Focus |  | 4 4 4 4 | 4 4 4 4 |  |  |  | 4 4 4 4 |  |
|  |  |  |  | Polarity |  | 7 7 7 7 | 7 7 7 7 |  |  |  | 7 7 7 1 |  |
| 148 | 148 | 9614212\_50 | When multiple agonist concentrations were applied to the same oocyte, |I:\*\*1MP3E0| the maximal increase in ICl amplitude varied considerably among different oocytes. |I:\*\*2SP3E0| |L:\*\*1SP3E0| |T:\*\*1SP3E1+| |
|  |  |  |  | Annotation |  | 1MP3E0 2SP3E0 | 1SP3E0 1SP3E0 |  |  |  | 1SP3E1+ 1SP3E1+ |  |
|  |  |  |  | Evidence |  | 1 1 | 1 1 |  |  |  | 2 2 |  |
|  |  |  |  | Focus |  | 2 4 | 4 4 |  |  |  | 4 4 |  |
|  |  |  |  | Polarity |  | 7 7 | 7 7 |  |  |  | 7 7 |  |
| 149 | 149 | 11968731\_3 | Unexpectedly, macroscopic examination of the resected kidney |I:\*\*1MP3E0| revealed multiple yellowish nodules located in the renal pelvis and calyces. |I:\*\*2SP3E0| |L:\*\*1SP3E0| |T:\*\*1SP3E1| |
|  |  |  |  | Annotation |  | 1MP3E0 2SP3E0 | 1SP3E0 1SP3E0 |  |  |  | 1SP3E1 1SP3E1 |  |
|  |  |  |  | Evidence |  | 1 1 | 1 1 |  |  |  | 2 2 |  |
|  |  |  |  | Focus |  | 2 4 | 4 4 |  |  |  | 4 4 |  |
|  |  |  |  | Polarity |  | 7 7 | 7 7 |  |  |  | 7 7 |  |
| 150 | 150 | 9716409\_263 | The requirement for this step in pairing is alleviated |I:\*\*1SP3E0| if homologs are physically attached to each other (analogous to our dimeric circular chromosome). |I:\*\*2SP0E0| |L:\*\*1GP3E0| |T:\*\*1SP3E1| |
|  |  |  |  | Annotation |  | 1SP3E0 2SP0E0 | 1GP3E0 1GP3E0 |  |  |  | 1SP3E1 1SP3E1 |  |
|  |  |  |  | Evidence |  | 1 1 | 1 1 |  |  |  | 2 2 |  |
|  |  |  |  | Focus |  | 4 4 | 1 1 |  |  |  | 4 4 |  |
|  |  |  |  | Polarity |  | 7 4 | 7 7 |  |  |  | 7 7 |  |
| 151 | 151 | 11595750\_12 | Whereas the activation conditions cannot be construed as physiological, |I:\*\*1MGN3E3| |L:\*\*1SN3E3| |T:\*\*1SN3E1| they did cause CEACAM1-L to co-localize with actin (Fig. 1 D) and caused extensive phosphorylation of CEACAM-1 on its Tyr residues (Fig. 2 B). |I:\*\*2SP3E3| |L:\*\*2SP3E3| Concomitantly with these changes, increased amounts of two major proteins at 200 and 45 kDa were observed in the CEACAM1-L immunoprecipitates (Fig. 2 C and Fig. 3). |I:\*\*1SP3E3+| |L:\*\*1SP3E3+| |T:\*\*1SP3E3| |
|  |  |  |  | Annotation |  | 1MGN3E3 2SP3E3 1SP3E3+ | 1SN3E3 2SP3E3 1SP3E3+ |  |  |  | 1SN3E1 1SP3E3 1SP3E3 |  |
|  |  |  |  | Evidence |  | 4 4 4 | 4 4 4 |  |  |  | 2 4 4 |  |
|  |  |  |  | Focus |  | 3 4 4 | 4 4 4 |  |  |  | 4 4 4 |  |
|  |  |  |  | Polarity |  | 1 7 7 | 1 7 7 |  |  |  | 1 7 7 |  |
| 152 | 152 | 12820963\_321 | A set of oligonucleotides (TGAGTTCCGACACTCCATCAGGAATTTCGAAGCTTGGAAATTCCTGATGGAGTGTCGGAACTCACCGTTTTTT and GATCAAAAAACGGTGAGTTCCGACACTCCATCAGGAATTTCCAAGCTTCGAAATTCCTGATGGAGTGTCGGAACTCACG) were annealed and ligated into piNEO cut with BseRI/amHI to generate piNEO-4E-BP1. |I:\*\*1MP3E0| |L:\*\*1MP3E3| |T:\*\*1MP3E3| |
|  |  |  |  | Annotation |  | 1MP3E0 | 1MP3E3 |  |  |  | 1MP3E3 |  |
|  |  |  |  | Evidence |  | 1 | 4 |  |  |  | 4 |  |
|  |  |  |  | Focus |  | 2 | 2 |  |  |  | 2 |  |
|  |  |  |  | Polarity |  | 7 | 7 |  |  |  | 7 |  |
| 153 | 153 | 8662906\_31 | Ras activity, |I:\*\*1SP3E3| measured as the ratio of Ras-bound GTP/(GTP + GDP), |I:\*\*2MP3E3| increased rapidly after the start of treatment with bufalin |I:\*\*3SP3E3+| |L:\*\*1SP3E3+| |T:\*\*1SP3E3+| and then decreased rapidly 30 min after the addition of bufalin (Fig. |I:\*\*4SP3E3-| |L:\*\*2SN3E3-| |T:\*\*2SP3E3-| |
|  |  |  |  | Annotation |  | 1SP3E3 2MP3E3 3SP3E3+ 4SP3E3- | 1SP3E3+ 1SP3E3+ 1SP3E3+ 2SN3E3- |  |  |  | 1SP3E3+ 1SP3E3+ 1SP3E3+ 2SP3E3- |  |
|  |  |  |  | Evidence |  | 4 4 4 4 | 4 4 4 4 |  |  |  | 4 4 4 4 |  |
|  |  |  |  | Focus |  | 4 2 4 4 | 4 4 4 4 |  |  |  | 4 4 4 4 |  |
|  |  |  |  | Polarity |  | 7 7 7 7 | 7 7 7 1 |  |  |  | 7 7 7 7 |  |
| 154 | 154 | 10391869\_6 | Thus, in addition to treating endotoxemias, |I:\*\*1SP3E0| the cumulative in vitro and in vivo data suggest that pentoxifylline may also be useful in abrogating the ill effects of staphylococcal enterotoxins and TSST-1. |I:\*\*2SP1E0| |L:\*\*1SP1E0| |T:\*\*1SP3E1+| |
|  |  |  |  | Annotation |  | 1SP3E0 2SP1E0 | 1SP1E0 1SP1E0 |  |  |  | 1SP3E1+ 1SP3E1+ |  |
|  |  |  |  | Evidence |  | 1 1 | 1 1 |  |  |  | 2 2 |  |
|  |  |  |  | Focus |  | 4 4 | 4 4 |  |  |  | 4 4 |  |
|  |  |  |  | Polarity |  | 7 5 | 5 5 |  |  |  | 7 7 |  |
| 155 | 155 | 11606566\_6 | As shown in Fig. 1, HeLa cells stained with antibody AXIX display a weak punctate staining in the cytoplasm. |I:\*\*1SGP3E3| |L:\*\*1SP3E3| |T:\*\*1SP3E3| |
|  |  |  |  | Annotation |  | 1SGP3E3 | 1SP3E3 |  |  |  | 1SP3E3 |  |
|  |  |  |  | Evidence |  | 4 | 4 |  |  |  | 4 |  |
|  |  |  |  | Focus |  | 5 | 4 |  |  |  | 4 |  |
|  |  |  |  | Polarity |  | 7 | 7 |  |  |  | 7 |  |
| 156 | 156 | 9367983\_269 | The plasmids containing synthetic promoters with multiple GAL4-binding sites upstream of the AdML TATA box and/or the TdT Inr element were described previously (Emami et al. 1995 ). |I:\*\*1MGP3E2| |L:\*\*1SP3E2| |T:\*\*1MP3E2| |
|  |  |  |  | Annotation |  | 1MGP3E2 | 1SP3E2 |  |  |  | 1MP3E2 |  |
|  |  |  |  | Evidence |  | 3 | 3 |  |  |  | 3 |  |
|  |  |  |  | Focus |  | 3 | 4 |  |  |  | 2 |  |
|  |  |  |  | Polarity |  | 7 | 7 |  |  |  | 7 |  |
| 157 | 157 | 11723140\_36 | FIX protein lacking the EGF1 domain or having the EGF1 domain of protein C only has a 10-fold reduced affinity for sTF (Fig. 8 B). |I:\*\*1SP3E3-| |L:\*\*1SP3E3-| |T:\*\*1SP3E3-| |
|  |  |  |  | Annotation |  | 1SP3E3- | 1SP3E3- |  |  |  | 1SP3E3- |  |
|  |  |  |  | Evidence |  | 4 | 4 |  |  |  | 4 |  |
|  |  |  |  | Focus |  | 4 | 4 |  |  |  | 4 |  |
|  |  |  |  | Polarity |  | 7 | 7 |  |  |  | 7 |  |
| 158 | 158 | 10207091\_43 | Briefly, the phosphorothioate-containing oligonucleotides were 5''-end labeled with 32P by using T4 polynucleotide kinase and annealed to a fivefold excess of its unlabeled complement. |I:\*\*1MP3E0| |L:\*\*1MP3E0| |T:\*\*1MP3E3| |
|  |  |  |  | Annotation |  | 1MP3E0 | 1MP3E0 |  |  |  | 1MP3E3 |  |
|  |  |  |  | Evidence |  | 1 | 1 |  |  |  | 4 |  |
|  |  |  |  | Focus |  | 2 | 2 |  |  |  | 2 |  |
|  |  |  |  | Polarity |  | 7 | 7 |  |  |  | 7 |  |
| 159 | 159 | 10358016\_46 | The data represent the average of triplicate determinations plus-or-minus S.D. |I:\*\*ERROR| |L:\*\*ERROR| |T:\*\*ERROR| Microfilament Structure Modulates Ca2+-dependent Exocytosis of Lysosomes-- |I:\*\*ERROR| |L:\*\*ERROR| |T:\*\*ERROR| There is a significant amount of evidence suggesting that the cortical actin network must be rearranged to allow secretory vesicles to contact and fuse with the plasma membrane ( , ). |I:\*\*1SP2E2| |L:\*\*1SP2E1| |T:\*\*1SP3E3| |
|  |  |  |  | Annotation |  | ERROR ERROR 1SP2E2 | ERROR ERROR 1SP2E1 |  |  |  | ERROR ERROR 1SP3E3 |  |
|  |  |  |  | Evidence |  | -1000 -1000 3 | -1000 -1000 2 |  |  |  | -1000 -1000 4 |  |
|  |  |  |  | Focus |  | -1000 -1000 4 | -1000 -1000 4 |  |  |  | -1000 -1000 4 |  |
|  |  |  |  | Polarity |  | -1000 -1000 6 | -1000 -1000 6 |  |  |  | -1000 -1000 7 |  |
| 160 | 160 | 9620370\_48 | Dendrograms based on SAB values were generated by the unweighted pair group method ( 31). |I:\*\*1SGP3E2| |L:\*\*1MP3E2| |T:\*\*1SP3E3| |
|  |  |  |  | Annotation |  | 1SGP3E2 | 1MP3E2 |  |  |  | 1SP3E3 |  |
|  |  |  |  | Evidence |  | 3 | 3 |  |  |  | 4 |  |
|  |  |  |  | Focus |  | 5 | 2 |  |  |  | 4 |  |
|  |  |  |  | Polarity |  | 7 | 7 |  |  |  | 7 |  |
| 161 | 161 | 8550627\_61 | At longer incubation times, |I:\*\*1MP3E3| |T:\*\*1SP3E1-| symmetric particles disappeared at low K concentration (5 mM), |I:\*\*2SP3E3-| |L:\*\*1SP3E3-| |T:\*\*2SP3E3+| but they increased at high K concentration (150 mM) in the same way as shown in Fig. 2. |I:\*\*3SP3E3+| |L:\*\*2SP3E3+| |T:\*\*3SP3E3+| When the GroEL-GroES system was preincubated ( Fig. 6 C) until almost 70% of the population corresponded to symmetric particles, |I:\*\*1MP3E3| |T:\*\*1SP3E3+| the addition of the denatured rhodanese led to the rapid disappearance of those complexes |I:\*\*2SP3E3-| |T:\*\*2SP3E3+| which were replaced by asymmetric particles. |I:\*\*3SP3E3+| |L:\*\*1SP3E3-| |T:\*\*3SP3E3+| |
|  |  |  |  | Annotation |  | 1MP3E3 2SP3E3- 3SP3E3+ 1MP3E3 2SP3E3- 3SP3E3+ | 1SP3E3- 1SP3E3- 2SP3E3+ 1SP3E3- 1SP3E3- 1SP3E3- |  |  |  | 1SP3E1- 2SP3E3+ 3SP3E3+ 1SP3E3+ 2SP3E3+ 3SP3E3+ |  |
|  |  |  |  | Evidence |  | 4 4 4 4 4 4 | 4 4 4 4 4 4 |  |  |  | 2 4 4 4 4 4 |  |
|  |  |  |  | Focus |  | 2 4 4 2 4 4 | 4 4 4 4 4 4 |  |  |  | 4 4 4 4 4 4 |  |
|  |  |  |  | Polarity |  | 7 7 7 7 7 7 | 7 7 7 7 7 7 |  |  |  | 7 7 7 7 7 7 |  |
| 162 | 162 | 10862775\_18 | The electrophoretic migration of the R900A, R921A, and R924A mutants was slightly slowed down compared with the migration of the HepV standard indicated on the left of the gel. |I:\*\*1SP3E3-| |L:\*\*1SP3E3-| |T:\*\*1SP3E3-| |
|  |  |  |  | Annotation |  | 1SP3E3- | 1SP3E3- |  |  |  | 1SP3E3- |  |
|  |  |  |  | Evidence |  | 4 | 4 |  |  |  | 4 |  |
|  |  |  |  | Focus |  | 4 | 4 |  |  |  | 4 |  |
|  |  |  |  | Polarity |  | 7 | 7 |  |  |  | 7 |  |
| 163 | 163 | 10224002\_83 | The PPX reaction was performed at 37 degrees C. Samples (2 mul) were taken periodically and loaded on a polyethyleneimine plate. |I:\*\*1MP3E0| |L:\*\*1MP3E3| |T:\*\*1MP3E3| |
|  |  |  |  | Annotation |  | 1MP3E0 | 1MP3E3 |  |  |  | 1MP3E3 |  |
|  |  |  |  | Evidence |  | 1 | 4 |  |  |  | 4 |  |
|  |  |  |  | Focus |  | 2 | 2 |  |  |  | 2 |  |
|  |  |  |  | Polarity |  | 7 | 7 |  |  |  | 7 |  |
| 164 | 164 | 11290333\_153 | This arrangement would seem to be energetically favorable, |I:\*\*1SP2E0| |T:\*\*1SP3E1| since Asn70 does not directly interact with peptide in Kbm8-SEV9 |I:\*\*2SN3E0| |L:\*\*1GN3E0| as it does in Kbm8-VSV8. |I:\*\*3SP3E0| |L:\*\*2GP3E0| |T:\*\*2SN3E1| |
|  |  |  |  | Annotation |  | 1SP2E0 2SN3E0 3SP3E0 | 1GN3E0 1GN3E0 2GP3E0 |  |  |  | 1SP3E1 2SN3E1 2SN3E1 |  |
|  |  |  |  | Evidence |  | 1 1 1 | 1 1 1 |  |  |  | 2 2 2 |  |
|  |  |  |  | Focus |  | 4 4 4 | 1 1 1 |  |  |  | 4 4 4 |  |
|  |  |  |  | Polarity |  | 6 1 7 | 1 1 7 |  |  |  | 7 1 1 |  |
| 165 | 165 | 12464701\_49 | In summary, if one only knows the aggregate percent optimal responses from a subject, |I:\*\*1SGP0E0| it is difficult to conclude anything about how that subject learned the task. |I:\*\*2SGP3E0| |L:\*\*1GSP0E3| |T:\*\*1SP3E0| |
|  |  |  |  | Annotation |  | 1SGP0E0 2SGP3E0 | 1GSP0E3 1GSP0E3 |  |  |  | 1SP3E0 1SP3E0 |  |
|  |  |  |  | Evidence |  | 1 1 | 4 4 |  |  |  | 1 1 |  |
|  |  |  |  | Focus |  | 5 5 | 5 5 |  |  |  | 4 4 |  |
|  |  |  |  | Polarity |  | 4 7 | 4 4 |  |  |  | 7 7 |  |
| 166 | 166 | 9252125\_89 | A predominant expression on cortical epithelial cells is also consistent with our examination of V 17a+ T cells in SJL mice injected intrathymically with AdcII-Eak. |I:\*\*1SP3E3| |L:\*\*1SP3E3| |T:\*\*1SP3E1| |
|  |  |  |  | Annotation |  | 1SP3E3 | 1SP3E3 |  |  |  | 1SP3E1 |  |
|  |  |  |  | Evidence |  | 4 | 4 |  |  |  | 2 |  |
|  |  |  |  | Focus |  | 4 | 4 |  |  |  | 4 |  |
|  |  |  |  | Polarity |  | 7 | 7 |  |  |  | 7 |  |
| 167 | 167 | 9463386\_217 | L929, HEC-1 and 293T cells were maintained in Eagle's minimun essential medium (MEM) supplemented with 5% fetal bovine serum (FBS). |I:\*\*1MP3E0| |L:\*\*1MP3E3| |T:\*\*1MP3E3| |
|  |  |  |  | Annotation |  | 1MP3E0 | 1MP3E3 |  |  |  | 1MP3E3 |  |
|  |  |  |  | Evidence |  | 1 | 4 |  |  |  | 4 |  |
|  |  |  |  | Focus |  | 2 | 2 |  |  |  | 2 |  |
|  |  |  |  | Polarity |  | 7 | 7 |  |  |  | 7 |  |
| 168 | 168 | 12851457\_12 | Furthermore, in our accompanying paper, we investigated the potential involvement of altered CN signaling in genetic susceptibility to schizophrenia, |I:\*\*1SGP0E3| and we report evidence supporting association of the PPP3CC gene encoding the CNAgamma catalytic subunit with disease ( 8). |I:\*\*2SP3E3| |L:\*\*1SP3E2| |T:\*\*1SP3E2| |
|  |  |  |  | Annotation |  | 1SGP0E3 2SP3E3 | 1SP3E2 1SP3E2 |  |  |  | 1SP3E2 1SP3E2 |  |
|  |  |  |  | Evidence |  | 4 4 | 3 3 |  |  |  | 3 3 |  |
|  |  |  |  | Focus |  | 5 4 | 4 4 |  |  |  | 4 4 |  |
|  |  |  |  | Polarity |  | 4 7 | 7 7 |  |  |  | 7 7 |  |
| 169 | 169 | 9482734\_395 | To construct pGEX-KG/beta-catenin-(423-781), |I:\*\*1MGP3E3| pBSKS/beta-catenin was digested with EcoRI and XhoI, and the 1.4 kb fragment encoding beta-catenin-(423-781) was inserted into the EcoRI- and XhoI-cut pGEX-KG. |I:\*\*2MP3E3| |L:\*\*1MP3E3| |T:\*\*1MP3E3| |
|  |  |  |  | Annotation |  | 1MGP3E3 2MP3E3 | 1MP3E3 1MP3E3 |  |  |  | 1MP3E3 1MP3E3 |  |
|  |  |  |  | Evidence |  | 4 4 | 4 4 |  |  |  | 4 4 |  |
|  |  |  |  | Focus |  | 3 2 | 2 2 |  |  |  | 2 2 |  |
|  |  |  |  | Polarity |  | 7 7 | 7 7 |  |  |  | 7 7 |  |
| 170 | 170 | 9748431\_204 | The close spacing of the genes in the flmAB, flmCD, and flmEF operons suggests that translation of these operons may be governed by a translational coupling mechanism. |I:\*\*1SP1E0| |L:\*\*1GSP1E0| |T:\*\*1SP3E1| |
|  |  |  |  | Annotation |  | 1SP1E0 | 1GSP1E0 |  |  |  | 1SP3E1 |  |
|  |  |  |  | Evidence |  | 1 | 1 |  |  |  | 2 |  |
|  |  |  |  | Focus |  | 4 | 5 |  |  |  | 4 |  |
|  |  |  |  | Polarity |  | 5 | 5 |  |  |  | 7 |  |
| 171 | 171 | 9728920\_184 | NSF may promote the dissociation of the AMPA receptor with GRIP or other synaptic proteins or regulate its accessibility to proteases and protein kinases. |I:\*\*1SP1E0| |L:\*\*1SP1E0-| |T:\*\*1SP3E1+| |
|  |  |  |  | Annotation |  | 1SP1E0 | 1SP1E0- |  |  |  | 1SP3E1+ |  |
|  |  |  |  | Evidence |  | 1 | 1 |  |  |  | 2 |  |
|  |  |  |  | Focus |  | 4 | 4 |  |  |  | 4 |  |
|  |  |  |  | Polarity |  | 5 | 5 |  |  |  | 7 |  |
| 172 | 172 | 11779461\_251 | We identified a novel FH protein, Daam1, which mediates Wnt/z activation of RhoA via the assembly of a Wnt-induced Dvl-RhoA complex. |I:\*\*1SP3E3| |L:\*\*1SP3E3| |T:\*\*1SP3E1+| |
|  |  |  |  | Annotation |  | 1SP3E3 | 1SP3E3 |  |  |  | 1SP3E1+ |  |
|  |  |  |  | Evidence |  | 4 | 4 |  |  |  | 2 |  |
|  |  |  |  | Focus |  | 4 | 4 |  |  |  | 4 |  |
|  |  |  |  | Polarity |  | 7 | 7 |  |  |  | 7 |  |
| 173 | 173 | 10893262\_308 | However, recovery after cytochrome c release may not depend solely on whether a cell is mitotic or postmitotic. |I:\*\*1SN1E0| |L:\*\*1SN1E0| |T:\*\*1SN1E1| |
|  |  |  |  | Annotation |  | 1SN1E0 | 1SN1E0 |  |  |  | 1SN1E1 |  |
|  |  |  |  | Evidence |  | 1 | 1 |  |  |  | 2 |  |
|  |  |  |  | Focus |  | 4 | 4 |  |  |  | 4 |  |
|  |  |  |  | Polarity |  | 3 | 3 |  |  |  | 3 |  |
| 174 | 174 | 9864271\_63 | The medium was acidified and metabolites extracted with ethyl acetate as described above. |I:\*\*1MP3E3| |L:\*\*1MP3E3| |T:\*\*1MP3E3| |
|  |  |  |  | Annotation |  | 1MP3E3 | 1MP3E3 |  |  |  | 1MP3E3 |  |
|  |  |  |  | Evidence |  | 4 | 4 |  |  |  | 4 |  |
|  |  |  |  | Focus |  | 2 | 2 |  |  |  | 2 |  |
|  |  |  |  | Polarity |  | 7 | 7 |  |  |  | 7 |  |
| 175 | 175 | 10790121\_102 | The PCR-RFLP analysis |I:\*\*1MP3E0| |T:\*\*1MP3E1| identified the five M. chelonae isolates as M. abscessus. |I:\*\*2SP3E0| |L:\*\*1SMP3E0| |T:\*\*2SP3E1| |
|  |  |  |  | Annotation |  | 1MP3E0 2SP3E0 | 1SMP3E0 1SMP3E0 |  |  |  | 1MP3E1 2SP3E1 |  |
|  |  |  |  | Evidence |  | 1 1 | 1 1 |  |  |  | 2 2 |  |
|  |  |  |  | Focus |  | 2 4 | 6 6 |  |  |  | 2 4 |  |
|  |  |  |  | Polarity |  | 7 7 | 7 7 |  |  |  | 7 7 |  |
| 176 | 176 | 8986723\_13 | Since all 2 integrins are deficient in these patients, |I:\*\*1SP3E0| |T:\*\*1SP3E1| the relative biologic importance of each of these heterodimers in the various leukocyte adhesion defects was derived indirectly |I:\*\*2SGP3E0| utilizing subunit-specific function-blocking monoclonal antibodies (MAbs). |I:\*\*3MP3E0| |L:\*\*1GMP3E3| |T:\*\*2MP3E3| |
|  |  |  |  | Annotation |  | 1SP3E0 2SGP3E0 3MP3E0 | 1GMP3E3 1GMP3E3 1GMP3E3 |  |  |  | 1SP3E1 2MP3E3 2MP3E3 |  |
|  |  |  |  | Evidence |  | 1 1 1 | 4 4 4 |  |  |  | 2 4 4 |  |
|  |  |  |  | Focus |  | 4 5 2 | 3 3 3 |  |  |  | 4 2 2 |  |
|  |  |  |  | Polarity |  | 7 7 7 | 7 7 7 |  |  |  | 7 7 7 |  |
| 177 | 177 | 8567665\_32 | The spectrum of the 22 ps component ( Fig. 2 a, dotted line) exhibits a maximum coincident with this bleach, |I:\*\*1SP3E3| thereby allowing us to associate this component with the formation of reduced pheophytin. |I:\*\*2SP1E3| |L:\*\*1SP3E3| |T:\*\*1SP3E3| |
|  |  |  |  | Annotation |  | 1SP3E3 2SP1E3 | 1SP3E3 1SP3E3 |  |  |  | 1SP3E3 1SP3E3 |  |
|  |  |  |  | Evidence |  | 4 4 | 4 4 |  |  |  | 4 4 |  |
|  |  |  |  | Focus |  | 4 4 | 4 4 |  |  |  | 4 4 |  |
|  |  |  |  | Polarity |  | 7 5 | 7 7 |  |  |  | 7 7 |  |
| 178 | 178 | 10811836\_202 | However, in every mouse with altered collagen X, hematopoiesis is affected, and is manifested by altered B and T lymphocyte development ( Fig 7 A; Jacenko, O., C.J. Gress, M.R. Campbell, Z. Tao, and D.W. Roberts, manuscript submitted for publication). |I:\*\*1SP3E3| |L:\*\*1SP3E2| |T:\*\*1SP3E3| |
|  |  |  |  | Annotation |  | 1SP3E3 | 1SP3E2 |  |  |  | 1SP3E3 |  |
|  |  |  |  | Evidence |  | 4 | 3 |  |  |  | 4 |  |
|  |  |  |  | Focus |  | 4 | 4 |  |  |  | 4 |  |
|  |  |  |  | Polarity |  | 7 | 7 |  |  |  | 7 |  |
| 179 | 179 | 12113544\_6 | Preoperative administration of gabexate mesilate (preop GM group) substantially ameliorated hepatic I/R injury as compared with the other patients (intraop and without GM groups); |I:\*\*1SP3E0+| |L:\*\*1SP3E3-| postoperative serum transaminase levels were notably decreased in association with marked suppression of IL-6 levels in blood circulation during liver surgery. |I:\*\*2SP3E0-| |L:\*\*2SP3E3+| |T:\*\*1SP3E3-| |
|  |  |  |  | Annotation |  | 1SP3E0+ 2SP3E0- | 1SP3E3- 2SP3E3+ |  |  |  | 1SP3E3- 1SP3E3- |  |
|  |  |  |  | Evidence |  | 1 1 | 4 4 |  |  |  | 4 4 |  |
|  |  |  |  | Focus |  | 4 4 | 4 4 |  |  |  | 4 4 |  |
|  |  |  |  | Polarity |  | 7 7 | 7 7 |  |  |  | 7 7 |  |
| 180 | 180 | 9334316\_288 | Thus, Bru has multiple roles in development; |I:\*\*1SP3E0| |L:\*\*1GSP3E0| given its role in repression of osk mRNA translation, |I:\*\*2SP3E0| we expect that Bru regulates the translation of multiple transcripts. |I:\*\*3SP1E3| |L:\*\*2GSP2E0| |T:\*\*1SP2E1| |
|  |  |  |  | Annotation |  | 1SP3E0 2SP3E0 3SP1E3 | 1GSP3E0 2GSP2E0 2GSP2E0 |  |  |  | 1SP2E1 1SP2E1 1SP2E1 |  |
|  |  |  |  | Evidence |  | 1 1 4 | 1 1 1 |  |  |  | 2 2 2 |  |
|  |  |  |  | Focus |  | 4 4 4 | 5 5 5 |  |  |  | 4 4 4 |  |
|  |  |  |  | Polarity |  | 7 7 5 | 7 6 6 |  |  |  | 6 6 6 |  |
| 181 | 181 | 10970351\_39 | The latter 35 isolates and 68 further B. cepacia complex isolates examined (103 total) were recovered from various sources including patients with CF, patients with non-CF infection, and the natural environment. |I:\*\*1SP3E0| |L:\*\*1MP3E3| |T:\*\*1MP3E3| |
|  |  |  |  | Annotation |  | 1SP3E0 | 1MP3E3 |  |  |  | 1MP3E3 |  |
|  |  |  |  | Evidence |  | 1 | 4 |  |  |  | 4 |  |
|  |  |  |  | Focus |  | 4 | 2 |  |  |  | 2 |  |
|  |  |  |  | Polarity |  | 7 | 7 |  |  |  | 7 |  |
| 182 | 182 | 10428033\_382 | An overnight culture grown in LB glucose was used to dilute cells 1:500 into minimal media supplemented with 0.4% glucose and 0.1% casamino acids. |I:\*\*1SP3E0| |L:\*\*1MP3E3| |T:\*\*1MP3E3| |
|  |  |  |  | Annotation |  | 1SP3E0 | 1MP3E3 |  |  |  | 1MP3E3 |  |
|  |  |  |  | Evidence |  | 1 | 4 |  |  |  | 4 |  |
|  |  |  |  | Focus |  | 4 | 2 |  |  |  | 2 |  |
|  |  |  |  | Polarity |  | 7 | 7 |  |  |  | 7 |  |
| 183 | 183 | 11483515\_374 | Ikeda,S., Kishida,S., Yamamoto,H., Murai,H., Koyama,S. and Kikuchi,A. (1998) |L:\*\*1GP3E3| Axin, a negative regulator of the Wnt signaling pathway, |L:\*\*2GSP3E0-| forms a complex with GSK-3beta and beta-catenin and promotes GSK-3beta-dependent phosphorylation of beta-catenin. |L:\*\*3SP3E3+| |I:\*\*1SP3E2| |T:\*\*1SP3E1| |
|  |  |  |  | Annotation |  | 1SP3E2 1SP3E2 1SP3E2 | 1GP3E3 2GSP3E0- 3SP3E3+ |  |  |  | 1SP3E1 1SP3E1 1SP3E1 |  |
|  |  |  |  | Evidence |  | 3 3 3 | 4 1 4 |  |  |  | 2 2 2 |  |
|  |  |  |  | Focus |  | 4 4 4 | 1 5 4 |  |  |  | 4 4 4 |  |
|  |  |  |  | Polarity |  | 7 7 7 | 7 7 7 |  |  |  | 7 7 7 |  |
| 184 | 184 | 9971749\_155 | We tested soluble laminin 5 effects on early kinase phosphorylation events. |I:\*\*1SGP0E3| |L:\*\*1GMP3E3| |T:\*\*1SP3E0| |
|  |  |  |  | Annotation |  | 1SGP0E3 | 1GMP3E3 |  |  |  | 1SP3E0 |  |
|  |  |  |  | Evidence |  | 4 | 4 |  |  |  | 1 |  |
|  |  |  |  | Focus |  | 5 | 3 |  |  |  | 4 |  |
|  |  |  |  | Polarity |  | 4 | 7 |  |  |  | 7 |  |
| 185 | 185 | 10549623\_154 | D10/MEKK2-GFP, D10/dnMEKK2-GFP, and noninfected D10 cells were activated by treatment with the stimulatory anti-TCR monoclonal antibody, 3D3 ( Kaye et al. 1983 ). |I:\*\*1MP3E2| |L:\*\*1SP3E2+| |T:\*\*1MP3E3| |
|  |  |  |  | Annotation |  | 1MP3E2 | 1SP3E2+ |  |  |  | 1MP3E3 |  |
|  |  |  |  | Evidence |  | 3 | 3 |  |  |  | 4 |  |
|  |  |  |  | Focus |  | 2 | 4 |  |  |  | 2 |  |
|  |  |  |  | Polarity |  | 7 | 7 |  |  |  | 7 |  |
| 186 | 186 | 11927033\_2 | Overall, 22% (24/109) of rats harbored adult worms, and 8% (4/48) of snails harbored A. cantonensis larvae. |I:\*\*1SP3E0| |L:\*\*1SP3E0| |T:\*\*1SP3E3| |
|  |  |  |  | Annotation |  | 1SP3E0 | 1SP3E0 |  |  |  | 1SP3E3 |  |
|  |  |  |  | Evidence |  | 1 | 1 |  |  |  | 4 |  |
|  |  |  |  | Focus |  | 4 | 4 |  |  |  | 4 |  |
|  |  |  |  | Polarity |  | 7 | 7 |  |  |  | 7 |  |
| 187 | 187 | 12072472\_215 | A more definitive determination was not possible by classical genetic analysis, |I:\*\*1SGN3E0| |L:\*\*1GN3E0| mainly due to the heterozygous genetic background of the strains. |I:\*\*2SGP3E0| |L:\*\*2SP3E0| |T:\*\*1SP3E1| |
|  |  |  |  | Annotation |  | 1SGN3E0 2SGP3E0 | 1GN3E0 2SP3E0 |  |  |  | 1SP3E1 1SP3E1 |  |
|  |  |  |  | Evidence |  | 1 1 | 1 1 |  |  |  | 2 2 |  |
|  |  |  |  | Focus |  | 5 5 | 1 4 |  |  |  | 4 4 |  |
|  |  |  |  | Polarity |  | 1 7 | 1 7 |  |  |  | 7 7 |  |
| 188 | 188 | 9742132\_378 | Thus, neither decapping nor cap modification is the explanation for deadenylation-dependent translational repression. |I:\*\*1SGN3E0| |L:\*\*1SN0E0-| |T:\*\*1SN3E1| |
|  |  |  |  | Annotation |  | 1SGN3E0 | 1SN0E0- |  |  |  | 1SN3E1 |  |
|  |  |  |  | Evidence |  | 1 | 1 |  |  |  | 2 |  |
|  |  |  |  | Focus |  | 5 | 4 |  |  |  | 4 |  |
|  |  |  |  | Polarity |  | 1 | 4 |  |  |  | 1 |  |
| 189 | 189 | 11427409\_59 | For this reason, the nasopharyngeal tissue could more appropriately reflect the interactions of HIV type 1 (HIV-1) with the lymphoid immune system. |I:\*\*1SP1E0| |L:\*\*1SP1E0| |T:\*\*1SP3E1| |
|  |  |  |  | Annotation |  | 1SP1E0 | 1SP1E0 |  |  |  | 1SP3E1 |  |
|  |  |  |  | Evidence |  | 1 | 1 |  |  |  | 2 |  |
|  |  |  |  | Focus |  | 4 | 4 |  |  |  | 4 |  |
|  |  |  |  | Polarity |  | 5 | 5 |  |  |  | 7 |  |
| 190 | 190 | 10572142\_48 | The screening probe was amplified by PCR with genomic DNA from Synechocystis sp. strain PCC 6803 as the template and the specific primers 5''-ATG GAT TCA CCA GCT ATT CTT (forward) and 5''-TTA AAC TGG TGT AAT ATT AAC (reverse). |I:\*\*1MP3E0| |L:\*\*1MP3E3| |T:\*\*1MP3E3| |
|  |  |  |  | Annotation |  | 1MP3E0 | 1MP3E3 |  |  |  | 1MP3E3 |  |
|  |  |  |  | Evidence |  | 1 | 4 |  |  |  | 4 |  |
|  |  |  |  | Focus |  | 2 | 2 |  |  |  | 2 |  |
|  |  |  |  | Polarity |  | 7 | 7 |  |  |  | 7 |  |
| 191 | 191 | 12135986\_20 | Similarly, in chick embryos, what appeared to be sustained activation of beta-catenin led to excessive feather and scale morphogenesis ( Noramly et al., 1999; Widelitz et al., 2000). |I:\*\*1SP1E2| |L:\*\*1SP3E2| |T:\*\*1SP3E3+| |
|  |  |  |  | Annotation |  | 1SP1E2 | 1SP3E2 |  |  |  | 1SP3E3+ |  |
|  |  |  |  | Evidence |  | 3 | 3 |  |  |  | 4 |  |
|  |  |  |  | Focus |  | 4 | 4 |  |  |  | 4 |  |
|  |  |  |  | Polarity |  | 5 | 7 |  |  |  | 7 |  |
| 192 | 192 | 11598069\_233 | Both of these experiments provide important information: |I:\*\*1SGP3E0| (i) LBP inhibits the binding of LPS aggregates to LBP intercalated in the target membrane, |L:\*\*1SP3E1-| (ii) LPS inhibits the binding of LBP to the target membrane, |L:\*\*2SP3E1-| |T:\*\*1SN3E1| and (iii) the binding of LPS to LBP is different from that of phospholipids to LBP. |L:\*\*3SP3E1| |I:\*\*2SP3E0| |T:\*\*2SP3E1| |
|  |  |  |  | Annotation |  | 1SGP3E0 2SP3E0 2SP3E0 2SP3E0 | 1SP3E1- 1SP3E1- 2SP3E1- 3SP3E1 |  |  |  | 1SN3E1 1SN3E1 1SN3E1 2SP3E1 |  |
|  |  |  |  | Evidence |  | 1 1 1 1 | 2 2 2 2 |  |  |  | 2 2 2 2 |  |
|  |  |  |  | Focus |  | 5 4 4 4 | 4 4 4 4 |  |  |  | 4 4 4 4 |  |
|  |  |  |  | Polarity |  | 7 7 7 7 | 7 7 7 7 |  |  |  | 1 1 1 7 |  |
| 193 | 193 | 9111038\_2 | Our purification protocol, starting from placental tissue, differs from that described in Ref. |I:\*\*1MGP3E3| and might be less prone to the co-purification of the true enzyme with annexin III. |I:\*\*2MSP3E3| |L:\*\*1GMP1E23| |T:\*\*1MP3E3| |
|  |  |  |  | Annotation |  | 1MGP3E3 2MSP3E3 | 1GMP1E23 1GMP1E23 |  |  |  | 1MP3E3 1MP3E3 |  |
|  |  |  |  | Evidence |  | 4 4 | 3 3 |  |  |  | 4 4 |  |
|  |  |  |  | Focus |  | 3 6 | 3 3 |  |  |  | 2 2 |  |
|  |  |  |  | Polarity |  | 7 7 | 5 5 |  |  |  | 7 7 |  |
| 194 | 194 | 12235123\_209 | A confocal microscopic analysis showed |I:\*\*1MP3E3| that a small but significant amount of DEDD colocalized with caspase-3 - cleaved K18 in the center, |I:\*\*2SP3E3| |L:\*\*1SP3E3| |T:\*\*1SP3E1| but not in the periphery of the cells ( Fig. 5 C). |I:\*\*3SN3E3| |L:\*\*2SN3E3| |T:\*\*2SN3E3| |
|  |  |  |  | Annotation |  | 1MP3E3 2SP3E3 3SN3E3 | 1SP3E3 1SP3E3 2SN3E3 |  |  |  | 1SP3E1 1SP3E1 2SN3E3 |  |
|  |  |  |  | Evidence |  | 4 4 4 | 4 4 4 |  |  |  | 2 2 4 |  |
|  |  |  |  | Focus |  | 2 4 4 | 4 4 4 |  |  |  | 4 4 4 |  |
|  |  |  |  | Polarity |  | 7 7 1 | 7 7 1 |  |  |  | 7 7 1 |  |
| 195 | 195 | 11473988\_68 | AG-CCA, TC-CPE-negative specimens were considered true negatives. |I:\*\*1MP3E0| |L:\*\*1SMP3E3| |T:\*\*1SP3E1| |
|  |  |  |  | Annotation |  | 1MP3E0 | 1SMP3E3 |  |  |  | 1SP3E1 |  |
|  |  |  |  | Evidence |  | 1 | 4 |  |  |  | 2 |  |
|  |  |  |  | Focus |  | 2 | 6 |  |  |  | 4 |  |
|  |  |  |  | Polarity |  | 7 | 7 |  |  |  | 7 |  |
| 196 | 196 | 11930015\_9 | Apo L proteins belong to the group of high density lipoproteins, with all six apo L genes located in close proximity to each other on chromosome 22q12, a confirmed high-susceptibility locus for schizophrenia and close to the region associated with velocardiofacial syndrome that includes symptoms of schizophrenia. |I:\*\*1SP3E0| |L:\*\*1SP3E0| |T:\*\*1SP3E1| |
|  |  |  |  | Annotation |  | 1SP3E0 | 1SP3E0 |  |  |  | 1SP3E1 |  |
|  |  |  |  | Evidence |  | 1 | 1 |  |  |  | 2 |  |
|  |  |  |  | Focus |  | 4 | 4 |  |  |  | 4 |  |
|  |  |  |  | Polarity |  | 7 | 7 |  |  |  | 7 |  |
| 197 | 197 | 10704886\_335 | Recently it was indicated that PKD1 modulates the Wnt signalling cascade by stabilizing endogenous -catenin and stimulating TCF-dependent gene transcription in vitro ( Kim et al., 1999). |I:\*\*1SP3E2| |L:\*\*1SP1E2+| |T:\*\*1SP3E2+| |
|  |  |  |  | Annotation |  | 1SP3E2 | 1SP1E2+ |  |  |  | 1SP3E2+ |  |
|  |  |  |  | Evidence |  | 3 | 3 |  |  |  | 3 |  |
|  |  |  |  | Focus |  | 4 | 4 |  |  |  | 4 |  |
|  |  |  |  | Polarity |  | 7 | 5 |  |  |  | 7 |  |
| 198 | 198 | 10949027\_277 | Bcl-2 binds Bid and has a weaker affinity for tBid ( Luo et al. 1998 ). |I:\*\*1SP3E2| |L:\*\*1SP3E2| |T:\*\*1SP3E2| |
|  |  |  |  | Annotation |  | 1SP3E2 | 1SP3E2 |  |  |  | 1SP3E2 |  |
|  |  |  |  | Evidence |  | 3 | 3 |  |  |  | 3 |  |
|  |  |  |  | Focus |  | 4 | 4 |  |  |  | 4 |  |
|  |  |  |  | Polarity |  | 7 | 7 |  |  |  | 7 |  |
| 199 | 199 | 11408566\_25 | Biochemically, JL13 was found to bind predominantly to the 5-HT2 and the D1 receptor, |L:\*\*1SP3E2| with much less potency for either the D2 or the muscarinic receptor (Bruhwyler et al., 1992 ; Liegeois et al., 1994 ). |L:\*\*2SP3E2-| |I:\*\*1SP3E2| |T:\*\*1SP3E2| |
|  |  |  |  | Annotation |  | 1SP3E2 1SP3E2 | 1SP3E2 2SP3E2- |  |  |  | 1SP3E2 1SP3E2 |  |
|  |  |  |  | Evidence |  | 3 3 | 3 3 |  |  |  | 3 3 |  |
|  |  |  |  | Focus |  | 4 4 | 4 4 |  |  |  | 4 4 |  |
|  |  |  |  | Polarity |  | 7 7 | 7 7 |  |  |  | 7 7 |  |
| 200 | 200 | 12237329\_10 | Clinically important responses to delta9-tetrahydrocannabinol (delta9-THC) and synthetic cannabinoids include the attenuation of nausea and vomiting in cancer chemotherapy, |I:\*\*1SP3E2-| |L:\*\*1GSP3E2-| stimulation of appetite in wasting syndromes, |I:\*\*2SP3E2+| |L:\*\*2SP3E2+| and reduction in intestinal motility (Robson, 2001 ). |I:\*\*3SP3E2-| |L:\*\*3SP3E2-| |T:\*\*1SP3E2| |
|  |  |  |  | Annotation |  | 1SP3E2- 2SP3E2+ 3SP3E2- | 1GSP3E2- 2SP3E2+ 3SP3E2- |  |  |  | 1SP3E2 1SP3E2 1SP3E2 |  |
|  |  |  |  | Evidence |  | 3 3 3 | 3 3 3 |  |  |  | 3 3 3 |  |
|  |  |  |  | Focus |  | 4 4 4 | 5 4 4 |  |  |  | 4 4 4 |  |
|  |  |  |  | Polarity |  | 7 7 7 | 7 7 7 |  |  |  | 7 7 7 |  |
| 201 | 201 | 12297048\_16 | Wnt ligands inactivate this kinase complex, |I:\*\*1SP3E0| allowing the accumulation of beta-catenin |I:\*\*2SP3E0+| and activation of nuclear signaling through the T cell factor |I:\*\*3SP3E0| (TCF)/Lymphoid enhancer factor-1 (LEF-1) family of transcription factors. |I:\*\*4SP3E0| |L:\*\*1SP3E0+| |T:\*\*1SP3E1| |
|  |  |  |  | Annotation |  | 1SP3E0 2SP3E0+ 3SP3E0 4SP3E0 | 1SP3E0+ 1SP3E0+ 1SP3E0+ 1SP3E0+ |  |  |  | 1SP3E1 1SP3E1 1SP3E1 1SP3E1 |  |
|  |  |  |  | Evidence |  | 1 1 1 1 | 1 1 1 1 |  |  |  | 2 2 2 2 |  |
|  |  |  |  | Focus |  | 4 4 4 4 | 4 4 4 4 |  |  |  | 4 4 4 4 |  |
|  |  |  |  | Polarity |  | 7 7 7 7 | 7 7 7 7 |  |  |  | 7 7 7 7 |  |
| 202 | 202 | 10473520\_15 | BDV is experimentally transmissible to other animal species and can also cause encephalomyelitis in a wide range of experimental animals ( 6, 7, 17). |I:\*\*1SP3E2| |L:\*\*1SP3E2| |T:\*\*1SP3E2| |
|  |  |  |  | Annotation |  | 1SP3E2 | 1SP3E2 |  |  |  | 1SP3E2 |  |
|  |  |  |  | Evidence |  | 3 | 3 |  |  |  | 3 |  |
|  |  |  |  | Focus |  | 4 | 4 |  |  |  | 4 |  |
|  |  |  |  | Polarity |  | 7 | 7 |  |  |  | 7 |  |
| 203 | 203 | 9535842\_53 | After incubation at 4 degrees C for 10 min and gentle agitation, cellular mixes were centrifuged at 2,000 x g for 10 min, rinsed once by centrifugation/resuspension in nucleus buffer without Triton X-100, and used as nuclei suspensions at a density of 1-2 x 10^7^ nuclei/ml. |I:\*\*1MP3E0| |L:\*\*1MP3E3| |T:\*\*1MP3E3| Supernatants were centrifuged at 10,000 x g for 10 min and used as cytosol. |I:\*\*1MP3E0| |L:\*\*1MP3E3| |T:\*\*1MP3E3| |
|  |  |  |  | Annotation |  | 1MP3E0 1MP3E0 | 1MP3E3 1MP3E3 |  |  |  | 1MP3E3 1MP3E3 |  |
|  |  |  |  | Evidence |  | 1 1 | 4 4 |  |  |  | 4 4 |  |
|  |  |  |  | Focus |  | 2 2 | 2 2 |  |  |  | 2 2 |  |
|  |  |  |  | Polarity |  | 7 7 | 7 7 |  |  |  | 7 7 |  |
| 204 | 204 | 9844016\_16 | alpha-Subunits are necessary for selectivity of the receptor for benzodiazepines ( 25), |L:\*\*1SP3E2| whereas the gamma2 subunit is essential for high-affinity benzodiazepine binding ( 18, 25-31). |L:\*\*2SP3E2| |I:\*\*1SP3E2| |T:\*\*1SP3E2| |
|  |  |  |  | Annotation |  | 1SP3E2 1SP3E2 | 1SP3E2 2SP3E2 |  |  |  | 1SP3E2 1SP3E2 |  |
|  |  |  |  | Evidence |  | 3 3 | 3 3 |  |  |  | 3 3 |  |
|  |  |  |  | Focus |  | 4 4 | 4 4 |  |  |  | 4 4 |  |
|  |  |  |  | Polarity |  | 7 7 | 7 7 |  |  |  | 7 7 |  |
| 205 | 205 | 9390512\_202 | Indeed, one of the very first molecular studies on schizophrenia, done in the era before the advent of the Human Genome Project, was based on the work of Bassett et al. 1988 , |I:\*\*1SGP3E0| who reported an Asian family in which the two family members with schizophrenia were found to have a partial trisomy of the chromosomal segment 5q11. |I:\*\*2SP3E2| |L:\*\*1GSP3E2| |T:\*\*1SP3E2| |
|  |  |  |  | Annotation |  | 1SGP3E0 2SP3E2 | 1GSP3E2 1GSP3E2 |  |  |  | 1SP3E2 1SP3E2 |  |
|  |  |  |  | Evidence |  | 1 3 | 3 3 |  |  |  | 3 3 |  |
|  |  |  |  | Focus |  | 5 4 | 5 5 |  |  |  | 4 4 |  |
|  |  |  |  | Polarity |  | 7 7 | 7 7 |  |  |  | 7 7 |  |
| 206 | 206 | 11399776\_42 | Bid is a proapoptotic member of the Bcl-2 family proteins that contains only a BH3 domain and can be cleaved by caspase-8 following Fas/TNF receptor-1 activation ( , ). |I:\*\*1SP3E2| |L:\*\*1SP3E0| |T:\*\*1SP3E2| |
|  |  |  |  | Annotation |  | 1SP3E2 | 1SP3E0 |  |  |  | 1SP3E2 |  |
|  |  |  |  | Evidence |  | 3 | 1 |  |  |  | 3 |  |
|  |  |  |  | Focus |  | 4 | 4 |  |  |  | 4 |  |
|  |  |  |  | Polarity |  | 7 | 7 |  |  |  | 7 |  |
| 207 | 207 | 12209241\_2 | The present study was carried out to determine whether the pathological mode of infiltrative growth (INF) of a metastatic liver nodule is useful in predicting recurrence in the remnant liver after hepatic resection. |I:\*\*1SGP0E3| |L:\*\*1GMP0E3| |T:\*\*1SP3E3| |
|  |  |  |  | Annotation |  | 1SGP0E3 | 1GMP0E3 |  |  |  | 1SP3E3 |  |
|  |  |  |  | Evidence |  | 4 | 4 |  |  |  | 4 |  |
|  |  |  |  | Focus |  | 5 | 3 |  |  |  | 4 |  |
|  |  |  |  | Polarity |  | 4 | 4 |  |  |  | 7 |  |
| 208 | 208 | 9524131\_297 | U5200 is a human DEAD box family member that has an essential yeast homolog (Snu246/Brr2/Slt22) (Lauber et al., 1996 ; Noble and Guthrie, 1996 ; Xu et al., 1996 ). |I:\*\*1SP3E2| |L:\*\*1SP3E2| |T:\*\*1SP3E2| |
|  |  |  |  | Annotation |  | 1SP3E2 | 1SP3E2 |  |  |  | 1SP3E2 |  |
|  |  |  |  | Evidence |  | 3 | 3 |  |  |  | 3 |  |
|  |  |  |  | Focus |  | 4 | 4 |  |  |  | 4 |  |
|  |  |  |  | Polarity |  | 7 | 7 |  |  |  | 7 |  |
| 209 | 209 | 11404408\_19 | In rats, kainate causes repeated seizure activity |L:\*\*1SP3E2| and ultimately leads to well defined patterns of destruction of hippocampal CA3 pyramidal neurons and hilar neurons (Ben-Ari, 1985 ). |L:\*\*2SP3E2-| |I:\*\*1SP3E2| |T:\*\*1SP3E2| |
|  |  |  |  | Annotation |  | 1SP3E2 1SP3E2 | 1SP3E2 2SP3E2- |  |  |  | 1SP3E2 1SP3E2 |  |
|  |  |  |  | Evidence |  | 3 3 | 3 3 |  |  |  | 3 3 |  |
|  |  |  |  | Focus |  | 4 4 | 4 4 |  |  |  | 4 4 |  |
|  |  |  |  | Polarity |  | 7 7 | 7 7 |  |  |  | 7 7 |  |
| 210 | 210 | 12078838\_1 | GnRH antagonists induce a rapid decrease in LH and FSH, |I:\*\*1SP3E0-| |L:\*\*1SP3E0+| preventing and interrupting LH surges. |I:\*\*2SP3E0| |L:\*\*2SP3E0-| |T:\*\*1SP3E1-| |
|  |  |  |  | Annotation |  | 1SP3E0- 2SP3E0 | 1SP3E0+ 2SP3E0- |  |  |  | 1SP3E1- 1SP3E1- |  |
|  |  |  |  | Evidence |  | 1 1 | 1 1 |  |  |  | 2 2 |  |
|  |  |  |  | Focus |  | 4 4 | 4 4 |  |  |  | 4 4 |  |
|  |  |  |  | Polarity |  | 7 7 | 7 7 |  |  |  | 7 7 |  |
| 211 | 211 | 9065447\_5 | To exclude a signal delivered by contaminating cells, |I:\*\*1SGP3E0| rtPCR analysis was applied using highly enriched (>99% pure) lung MC. |I:\*\*2MP3E0| |L:\*\*1MP3E3| |T:\*\*1MP3E3| |
|  |  |  |  | Annotation |  | 1SGP3E0 2MP3E0 | 1MP3E3 1MP3E3 |  |  |  | 1MP3E3 1MP3E3 |  |
|  |  |  |  | Evidence |  | 1 1 | 4 4 |  |  |  | 4 4 |  |
|  |  |  |  | Focus |  | 5 2 | 2 2 |  |  |  | 2 2 |  |
|  |  |  |  | Polarity |  | 7 7 | 7 7 |  |  |  | 7 7 |  |
| 212 | 212 | 10329633\_27 | Whether any of these enzymes contribute to the maturation of 16S RNA was not known. |I:\*\*1SN3E0| |L:\*\*1SP0E0| |T:\*\*1SP0E0| |
|  |  |  |  | Annotation |  | 1SN3E0 | 1SP0E0 |  |  |  | 1SP0E0 |  |
|  |  |  |  | Evidence |  | 1 | 1 |  |  |  | 1 |  |
|  |  |  |  | Focus |  | 4 | 4 |  |  |  | 4 |  |
|  |  |  |  | Polarity |  | 1 | 4 |  |  |  | 4 |  |
| 213 | 213 | 10793060\_5 | Such matrix alterations occur during or as a result of tissue injury, |I:\*\*1SP3E0| and thus, the appearance of matricryptic sites within an injury site may provide important new signals to regulate the repair process. |I:\*\*2SP1E0| |L:\*\*1SP1E0| |T:\*\*1SP3E1| |
|  |  |  |  | Annotation |  | 1SP3E0 2SP1E0 | 1SP1E0 1SP1E0 |  |  |  | 1SP3E1 1SP3E1 |  |
|  |  |  |  | Evidence |  | 1 1 | 1 1 |  |  |  | 2 2 |  |
|  |  |  |  | Focus |  | 4 4 | 4 4 |  |  |  | 4 4 |  |
|  |  |  |  | Polarity |  | 7 5 | 5 5 |  |  |  | 7 7 |  |
| 214 | 214 | 9430686\_216 | However, if 2% or more of transfer events were inaccurate |I:\*\*1SP0E0| this could have been easily detected. |I:\*\*2SGP2E0| |L:\*\*1GSP0E0| |T:\*\*1SP3E1| |
|  |  |  |  | Annotation |  | 1SP0E0 2SGP2E0 | 1GSP0E0 1GSP0E0 |  |  |  | 1SP3E1 1SP3E1 |  |
|  |  |  |  | Evidence |  | 1 1 | 1 1 |  |  |  | 2 2 |  |
|  |  |  |  | Focus |  | 4 5 | 5 5 |  |  |  | 4 4 |  |
|  |  |  |  | Polarity |  | 4 6 | 4 4 |  |  |  | 7 7 |  |
| 215 | 215 | 10764813\_28 | The Ca2+ may be directly involved in activating exocytosis; |I:\*\*1SP1E0| |L:\*\*1SP1E0+| however, our data favor a requirement for PKC activation. |I:\*\*2SP1E3| |L:\*\*2SP3E3+| |T:\*\*1SP1E1| |
|  |  |  |  | Annotation |  | 1SP1E0 2SP1E3 | 1SP1E0+ 2SP3E3+ |  |  |  | 1SP1E1 1SP1E1 |  |
|  |  |  |  | Evidence |  | 1 4 | 1 4 |  |  |  | 2 2 |  |
|  |  |  |  | Focus |  | 4 4 | 4 4 |  |  |  | 4 4 |  |
|  |  |  |  | Polarity |  | 5 5 | 5 7 |  |  |  | 5 5 |  |
| 216 | 216 | 11861654\_46 | NADPH/NADH oxidase activity in the cytosolic fraction of incubated cells remained unchanged (data not shown). |I:\*\*1SP3E3| |L:\*\*1SP3E1| |T:\*\*1SP3E1| |
|  |  |  |  | Annotation |  | 1SP3E3 | 1SP3E1 |  |  |  | 1SP3E1 |  |
|  |  |  |  | Evidence |  | 4 | 2 |  |  |  | 2 |  |
|  |  |  |  | Focus |  | 4 | 4 |  |  |  | 4 |  |
|  |  |  |  | Polarity |  | 7 | 7 |  |  |  | 7 |  |
| 217 | 217 | 7814421\_51 | ( ) The abbreviations used are: BHK, baby hamster kidney; PAGE, polyacrylamide gel electrophoresis; TF, tissue factor; TF , recombinant COOH-terminal truncated TF-containing residues 1-219; gla, -carboxyglutamic acid; EGRck, glutamyl-glycyl-arginyl chloromethyl ketone; TPCK, N-tosyl-L-phenylalanine chloromethyl ketone; CAPS, 3-cyclohexylamino-1-propanesulfonic acid; DMEM, Dulbecco's modified Eagle's medium; MBP, maltose binding protein; IPTG, isopropyl-thiogalactopyranoside; MTX, methotrexate. |I:\*\*1MGP3E3| |L:\*\*1GP3E3| |T:\*\*1GP3E3| |
|  |  |  |  | Annotation |  | 1MGP3E3 | 1GP3E3 |  |  |  | 1GP3E3 |  |
|  |  |  |  | Evidence |  | 4 | 4 |  |  |  | 4 |  |
|  |  |  |  | Focus |  | 3 | 1 |  |  |  | 1 |  |
|  |  |  |  | Polarity |  | 7 | 7 |  |  |  | 7 |  |
| 218 | 218 | 9846492\_165 | Consistent with this is the observation that V 8+ T cells in SCID mice reconstituted with wt or gld splenocytes |T:\*\*1MP3E3| showed the same pattern of expansion and deletion following administration of SEB ( Figure 7B). |T:\*\*2SP3E2| |I:\*\*1SP1E3| |L:\*\*1SP3E3| |
|  |  |  |  | Annotation |  | 1SP1E3 1SP1E3 | 1SP3E3 1SP3E3 |  |  |  | 1MP3E3 2SP3E2 |  |
|  |  |  |  | Evidence |  | 4 4 | 4 4 |  |  |  | 4 3 |  |
|  |  |  |  | Focus |  | 4 4 | 4 4 |  |  |  | 2 4 |  |
|  |  |  |  | Polarity |  | 5 5 | 7 7 |  |  |  | 7 7 |  |
| 219 | 219 | 10555147\_218 | Recombinant adenovirus expressing wild type and mutant of human PLB were generated by homologous recombination between plasmid pJM17 and a shuttle plasmid with RSV promoter and SV40 polyA sequences (Wang et al., 1998 ). |I:\*\*1MP3E2| |L:\*\*1MP3E2| |T:\*\*1MP3E2| |
|  |  |  |  | Annotation |  | 1MP3E2 | 1MP3E2 |  |  |  | 1MP3E2 |  |
|  |  |  |  | Evidence |  | 3 | 3 |  |  |  | 3 |  |
|  |  |  |  | Focus |  | 2 | 2 |  |  |  | 2 |  |
|  |  |  |  | Polarity |  | 7 | 7 |  |  |  | 7 |  |
| 220 | 220 | 11369803\_55 | Hi-5 cells produced 2-fold more secreted rPON1A arylesterase activity than Sf9 cells (data not shown). |I:\*\*1SP3E3| |L:\*\*1SP3E1+| |T:\*\*1SP3E1| |
|  |  |  |  | Annotation |  | 1SP3E3 | 1SP3E1+ |  |  |  | 1SP3E1 |  |
|  |  |  |  | Evidence |  | 4 | 2 |  |  |  | 2 |  |
|  |  |  |  | Focus |  | 4 | 4 |  |  |  | 4 |  |
|  |  |  |  | Polarity |  | 7 | 7 |  |  |  | 7 |  |
| 221 | 221 | 10200329\_24 | In addition, we tested a group of first-degree relatives of schizophrenic patients on the same procedures, for two reasons: |I:\*\*1SGP3E3| ( i) to determine whether a motion discrimination deficit is merely a consequence of having a schizophrenic illness or of being treated for that illness with antipsychotic medications; and ( ii) to determine the familiality of the deficits. |I:\*\*2SGP0E3| |L:\*\*1MSP0E3| |T:\*\*1MP3E3| |
|  |  |  |  | Annotation |  | 1SGP3E3 2SGP0E3 | 1MSP0E3 1MSP0E3 |  |  |  | 1MP3E3 1MP3E3 |  |
|  |  |  |  | Evidence |  | 4 4 | 4 4 |  |  |  | 4 4 |  |
|  |  |  |  | Focus |  | 5 5 | 6 6 |  |  |  | 2 2 |  |
|  |  |  |  | Polarity |  | 7 4 | 4 4 |  |  |  | 7 7 |  |
| 222 | 222 | 8576215\_36 | At the indicated times 80-ml aliquots of the growth medium were removed and crude cell membranes were prepared. |I:\*\*1MP3E3| |L:\*\*1MP3E3| |T:\*\*1MP3E3| |
|  |  |  |  | Annotation |  | 1MP3E3 | 1MP3E3 |  |  |  | 1MP3E3 |  |
|  |  |  |  | Evidence |  | 4 | 4 |  |  |  | 4 |  |
|  |  |  |  | Focus |  | 2 | 2 |  |  |  | 2 |  |
|  |  |  |  | Polarity |  | 7 | 7 |  |  |  | 7 |  |
| 223 | 223 | 9799806\_46 | The mononuclear cells were plated into cell culture-treated plastic 35-mm wells (5 million cells per well in 6-well plates to yield 1 million monocytes per well). |I:\*\*1MP3E0| |L:\*\*1MP3E3| |T:\*\*1MP3E3| |
|  |  |  |  | Annotation |  | 1MP3E0 | 1MP3E3 |  |  |  | 1MP3E3 |  |
|  |  |  |  | Evidence |  | 1 | 4 |  |  |  | 4 |  |
|  |  |  |  | Focus |  | 2 | 2 |  |  |  | 2 |  |
|  |  |  |  | Polarity |  | 7 | 7 |  |  |  | 7 |  |
| 224 | 224 | 11713291\_62 | Additionally, hsp70i can exhibit an antiapoptotic function via direct association with the caspase recruitment domain of Apaf-1 and inhibition of apoptosome formation ( 3, 40). |I:\*\*1SP3E2| |L:\*\*1SP3E2| |T:\*\*1SP1E2| |
|  |  |  |  | Annotation |  | 1SP3E2 | 1SP3E2 |  |  |  | 1SP1E2 |  |
|  |  |  |  | Evidence |  | 3 | 3 |  |  |  | 3 |  |
|  |  |  |  | Focus |  | 4 | 4 |  |  |  | 4 |  |
|  |  |  |  | Polarity |  | 7 | 7 |  |  |  | 5 |  |
| 225 | 225 | 10545098\_296 | The concentration of motor in pellet and supernatant was plotted using Kaleidagraph 3.05 (Synergy software), and binding parameters were obtained by fitting rectangular hyperbolae by least squares analysis. |I:\*\*1MP3E3| |L:\*\*1MP3E3| |T:\*\*1MP3E3| |
|  |  |  |  | Annotation |  | 1MP3E3 | 1MP3E3 |  |  |  | 1MP3E3 |  |
|  |  |  |  | Evidence |  | 4 | 4 |  |  |  | 4 |  |
|  |  |  |  | Focus |  | 2 | 2 |  |  |  | 2 |  |
|  |  |  |  | Polarity |  | 7 | 7 |  |  |  | 7 |  |
| 226 | 226 | 12183451\_12 | Unlike most conventional chemotherapeutic drugs, |I:\*\*1SP3E0| ALP does not target the DNA |I:\*\*2SN3E0| |L:\*\*1SN3E0| but acts at the level of cell membranes. |I:\*\*3SP3E0| |L:\*\*2SP3E0| |T:\*\*1SP3E1| |
|  |  |  |  | Annotation |  | 1SP3E0 2SN3E0 3SP3E0 | 1SN3E0 1SN3E0 2SP3E0 |  |  |  | 1SP3E1 1SP3E1 1SP3E1 |  |
|  |  |  |  | Evidence |  | 1 1 1 | 1 1 1 |  |  |  | 2 2 2 |  |
|  |  |  |  | Focus |  | 4 4 4 | 4 4 4 |  |  |  | 4 4 4 |  |
|  |  |  |  | Polarity |  | 7 1 7 | 1 1 7 |  |  |  | 7 7 7 |  |
| 227 | 227 | 10588954\_47 | Normally, for use as a substrate or an inhibitor, at the start of an assay, the purity of each retinoid used in assays was at least 99% or greater. |I:\*\*1MP3E0| |L:\*\*1MP3E3| |T:\*\*1MP3E3| |
|  |  |  |  | Annotation |  | 1MP3E0 | 1MP3E3 |  |  |  | 1MP3E3 |  |
|  |  |  |  | Evidence |  | 1 | 4 |  |  |  | 4 |  |
|  |  |  |  | Focus |  | 2 | 2 |  |  |  | 2 |  |
|  |  |  |  | Polarity |  | 7 | 7 |  |  |  | 7 |  |
| 228 | 228 | 9520479\_109 | ( B) Sympathetic neurons (SCG neurons), cultured for 5-7 days in the presence of NGF, were coinjected with 0.1 mg/ml V12Cdc42 (solid bar) and increasing concentrations of N17Rac1 (hatched bars) or 0.4 mg/ml pRK5 (open bar). |I:\*\*1MP3E3| |L:\*\*1MP3E3| |T:\*\*1MP3E3| |
|  |  |  |  | Annotation |  | 1MP3E3 | 1MP3E3 |  |  |  | 1MP3E3 |  |
|  |  |  |  | Evidence |  | 4 | 4 |  |  |  | 4 |  |
|  |  |  |  | Focus |  | 2 | 2 |  |  |  | 2 |  |
|  |  |  |  | Polarity |  | 7 | 7 |  |  |  | 7 |  |
| 229 | 229 | 11290744\_13 | Several transporters are modulated by post-transcriptional regulation of their trafficking to the plasma membrane and/or by internalization from the plasma membrane to intracellular compartments ( , ). |I:\*\*1SP3E2| |L:\*\*1SP3E0| |T:\*\*1SP3E2| |
|  |  |  |  | Annotation |  | 1SP3E2 | 1SP3E0 |  |  |  | 1SP3E2 |  |
|  |  |  |  | Evidence |  | 3 | 1 |  |  |  | 3 |  |
|  |  |  |  | Focus |  | 4 | 4 |  |  |  | 4 |  |
|  |  |  |  | Polarity |  | 7 | 7 |  |  |  | 7 |  |
| 230 | 230 | 12885555\_225 | It was also reported that secreted Wnt binding proteins can activate the Wnt pathway, such as FZD4S, a soluble-type splicing variant of Frizzled-4 [ Sagara et al 2001]. |I:\*\*1SP3E2| |L:\*\*1SP3E2+| |T:\*\*1SP2E2| |
|  |  |  |  | Annotation |  | 1SP3E2 | 1SP3E2+ |  |  |  | 1SP2E2 |  |
|  |  |  |  | Evidence |  | 3 | 3 |  |  |  | 3 |  |
|  |  |  |  | Focus |  | 4 | 4 |  |  |  | 4 |  |
|  |  |  |  | Polarity |  | 7 | 7 |  |  |  | 6 |  |
| 231 | 231 | 10662691\_161 | UrdGT1b and UrdGT1c transfer different NDP-sugars to different alcohol substrates. |I:\*\*1SP3E0| |L:\*\*1SP3E0| |T:\*\*1SP3E1| |
|  |  |  |  | Annotation |  | 1SP3E0 | 1SP3E0 |  |  |  | 1SP3E1 |  |
|  |  |  |  | Evidence |  | 1 | 1 |  |  |  | 2 |  |
|  |  |  |  | Focus |  | 4 | 4 |  |  |  | 4 |  |
|  |  |  |  | Polarity |  | 7 | 7 |  |  |  | 7 |  |
| 232 | 232 | 11094078\_308 | Inhibition of Ras did not significantly affect PI 3-kinase activity (Fig. 1). |I:\*\*1SN3E3| |L:\*\*1SN3E3-| |T:\*\*1SN3E3| |
|  |  |  |  | Annotation |  | 1SN3E3 | 1SN3E3- |  |  |  | 1SN3E3 |  |
|  |  |  |  | Evidence |  | 4 | 4 |  |  |  | 4 |  |
|  |  |  |  | Focus |  | 4 | 4 |  |  |  | 4 |  |
|  |  |  |  | Polarity |  | 1 | 1 |  |  |  | 1 |  |
| 233 | 233 | 10811813\_98 | Given the density of CACC and E-boxes in 330 to +172, the necessity of this sequence for unloading sensitivity of SERCA1 ( 3636)-pGL3, and the requirement for the sequence between 21 and +27, which contains a CACC site and an E-box, |I:\*\*1SP3E0| it is likely that these elements are critical to transducing transactivation in response to the removal of weight-bearing in the soleus muscle. |I:\*\*2SP1E3| |L:\*\*1SP1E3| |T:\*\*1SP2E3| |
|  |  |  |  | Annotation |  | 1SP3E0 2SP1E3 | 1SP1E3 1SP1E3 |  |  |  | 1SP2E3 1SP2E3 |  |
|  |  |  |  | Evidence |  | 1 4 | 4 4 |  |  |  | 4 4 |  |
|  |  |  |  | Focus |  | 4 4 | 4 4 |  |  |  | 4 4 |  |
|  |  |  |  | Polarity |  | 7 5 | 5 5 |  |  |  | 6 6 |  |
| 234 | 234 | 10821833\_97 | The apparent small modulation of relative repair rates observed in Fig. 8 D is not significant. |I:\*\*1SN3E3| |L:\*\*1SN3E3| |T:\*\*1SP3E3| |
|  |  |  |  | Annotation |  | 1SN3E3 | 1SN3E3 |  |  |  | 1SP3E3 |  |
|  |  |  |  | Evidence |  | 4 | 4 |  |  |  | 4 |  |
|  |  |  |  | Focus |  | 4 | 4 |  |  |  | 4 |  |
|  |  |  |  | Polarity |  | 1 | 1 |  |  |  | 7 |  |
| 235 | 235 | 11003670\_401 | Moreover, these data demonstrate that the combined regulation of S-phase entry and S-phase progression likely underlie the function of RB as a tumor suppressor. |I:\*\*1SP1E0| |L:\*\*1SP1E3-| |T:\*\*1SP2E1| |
|  |  |  |  | Annotation |  | 1SP1E0 | 1SP1E3- |  |  |  | 1SP2E1 |  |
|  |  |  |  | Evidence |  | 1 | 4 |  |  |  | 2 |  |
|  |  |  |  | Focus |  | 4 | 4 |  |  |  | 4 |  |
|  |  |  |  | Polarity |  | 5 | 5 |  |  |  | 6 |  |
| 236 | 236 | 10924491\_45 | The homozygous viability (nu ii) of each copy i was assayed at different generations as the percentage of wild adults + i/+ i in the offspring of a cross between five Cy/+ i females and five Cy/+ i males |I:\*\*1SGP0E0| in a half-pint bottle. |I:\*\*2MP3E0| |L:\*\*1MP3E3| |T:\*\*1MP3E3| |
|  |  |  |  | Annotation |  | 1SGP0E0 2MP3E0 | 1MP3E3 1MP3E3 |  |  |  | 1MP3E3 1MP3E3 |  |
|  |  |  |  | Evidence |  | 1 1 | 4 4 |  |  |  | 4 4 |  |
|  |  |  |  | Focus |  | 5 2 | 2 2 |  |  |  | 2 2 |  |
|  |  |  |  | Polarity |  | 4 7 | 7 7 |  |  |  | 7 7 |  |
| 237 | 237 | 12810961\_7 | Cells expressing TGase2 antisense RNA are less sensitive to apoptosis induced by various agents |T:\*\*1SP3E1-| |I:\*\*1SP3E2-| |L:\*\*1SP3E2| whereas cells overexpressing TGase2 have increased sensitivity ( 13, 14) |T:\*\*2SP3E2+| |I:\*\*2SP3E2+| |L:\*\*2SP3E2+| |
|  |  |  |  | Annotation |  | 1SP3E2- 2SP3E2+ | 1SP3E2 2SP3E2+ |  |  |  | 1SP3E1- 2SP3E2+ |  |
|  |  |  |  | Evidence |  | 3 3 | 3 3 |  |  |  | 2 3 |  |
|  |  |  |  | Focus |  | 4 4 | 4 4 |  |  |  | 4 4 |  |
|  |  |  |  | Polarity |  | 7 7 | 7 7 |  |  |  | 7 7 |  |
| 238 | 238 | 9303536\_75 | Primers were used to amplify a 1086-bp fragment of lacZ and 773 bp fragment of bioA on total RNA isolated from the pL- nutL-N-lacZ gene fusion strain carrying pZH124(N+), pGB2(N ), or pZH126(Nun+). |I:\*\*1MP3E0| |L:\*\*1MP3E3| |T:\*\*1SP3E3| |
|  |  |  |  | Annotation |  | 1MP3E0 | 1MP3E3 |  |  |  | 1SP3E3 |  |
|  |  |  |  | Evidence |  | 1 | 4 |  |  |  | 4 |  |
|  |  |  |  | Focus |  | 2 | 2 |  |  |  | 4 |  |
|  |  |  |  | Polarity |  | 7 | 7 |  |  |  | 7 |  |
| 239 | 239 | 11086989\_170 | It is evident that the retinal degeneration in rdgB is a very complex process. |I:\*\*1SP3E0-| |L:\*\*1GSP3E1| |T:\*\*1SP3E1| |
|  |  |  |  | Annotation |  | 1SP3E0- | 1GSP3E1 |  |  |  | 1SP3E1 |  |
|  |  |  |  | Evidence |  | 1 | 2 |  |  |  | 2 |  |
|  |  |  |  | Focus |  | 4 | 5 |  |  |  | 4 |  |
|  |  |  |  | Polarity |  | 7 | 7 |  |  |  | 7 |  |
| 240 | 240 | 11294848\_56 | These results indicated that CP10A formed a more ordered structure in the presence of lipids. |I:\*\*1SP3E0| |L:\*\*1SP1E3| |T:\*\*1SP2E1| |
|  |  |  |  | Annotation |  | 1SP3E0 | 1SP1E3 |  |  |  | 1SP2E1 |  |
|  |  |  |  | Evidence |  | 1 | 4 |  |  |  | 2 |  |
|  |  |  |  | Focus |  | 4 | 4 |  |  |  | 4 |  |
|  |  |  |  | Polarity |  | 7 | 5 |  |  |  | 6 |  |
| 241 | 241 | 10473520\_95 | In addition, the ECLIA corresponded to IFA in domestic horses that were also seropositive by a sensitive, specific WB assay. |I:\*\*1SP3E0| |L:\*\*1SP3E0| |T:\*\*1MP3E3| |
|  |  |  |  | Annotation |  | 1SP3E0 | 1SP3E0 |  |  |  | 1MP3E3 |  |
|  |  |  |  | Evidence |  | 1 | 1 |  |  |  | 4 |  |
|  |  |  |  | Focus |  | 4 | 4 |  |  |  | 2 |  |
|  |  |  |  | Polarity |  | 7 | 7 |  |  |  | 7 |  |
| 242 | 242 | 9618524\_59 | The cells were treated with as described above and harvested at different time points (3, 6, and 12 hr after PDT). |I:\*\*1MP3E0| |L:\*\*1MP3E3| |T:\*\*1MP3E3| |
|  |  |  |  | Annotation |  | 1MP3E0 | 1MP3E3 |  |  |  | 1MP3E3 |  |
|  |  |  |  | Evidence |  | 1 | 4 |  |  |  | 4 |  |
|  |  |  |  | Focus |  | 2 | 2 |  |  |  | 2 |  |
|  |  |  |  | Polarity |  | 7 | 7 |  |  |  | 7 |  |
| 243 | 243 | 10200329\_29 | We reasoned that, |I:\*\*1GP3E3| if the velocity discrimination deficit were familial |I:\*\*2SP0E3| |L:\*\*1SP3E0| and not a consequence of clinical schizophrenia or its treatment, |I:\*\*3SN0E3| |L:\*\*2SN3E0| then a significant proportion of relatives would show the same motion processing deficits seen in the schizophrenic patients. |I:\*\*4SP0E3| |L:\*\*3SP1E0| |T:\*\*1SP3E0| |
|  |  |  |  | Annotation |  | 1GP3E3 2SP0E3 3SN0E3 4SP0E3 | 1SP3E0 1SP3E0 2SN3E0 3SP1E0 |  |  |  | 1SP3E0 1SP3E0 1SP3E0 1SP3E0 |  |
|  |  |  |  | Evidence |  | 4 4 4 4 | 1 1 1 1 |  |  |  | 1 1 1 1 |  |
|  |  |  |  | Focus |  | 1 4 4 4 | 4 4 4 4 |  |  |  | 4 4 4 4 |  |
|  |  |  |  | Polarity |  | 7 4 4 4 | 7 7 1 5 |  |  |  | 7 7 7 7 |  |
| 244 | 244 | 10377139\_84 | In these experiments, total CFU in each well was determined only on day 7. |I:\*\*1MP3E0| |L:\*\*1MP3E3| |T:\*\*1MP3E3| |
|  |  |  |  | Annotation |  | 1MP3E0 | 1MP3E3 |  |  |  | 1MP3E3 |  |
|  |  |  |  | Evidence |  | 1 | 4 |  |  |  | 4 |  |
|  |  |  |  | Focus |  | 2 | 2 |  |  |  | 2 |  |
|  |  |  |  | Polarity |  | 7 | 7 |  |  |  | 7 |  |
| 245 | 245 | 12438416\_30 | A stable cell line expressing a cleavage-resistant form of p115 delayed Golgi fragmentation during apoptosis. |I:\*\*1SP3E0| |L:\*\*1SP3E0| |T:\*\*1SP3E1| |
|  |  |  |  | Annotation |  | 1SP3E0 | 1SP3E0 |  |  |  | 1SP3E1 |  |
|  |  |  |  | Evidence |  | 1 | 1 |  |  |  | 2 |  |
|  |  |  |  | Focus |  | 4 | 4 |  |  |  | 4 |  |
|  |  |  |  | Polarity |  | 7 | 7 |  |  |  | 7 |  |
| 246 | 246 | 12821644\_126 | In contrast, the hypophosphorylated form of pRb predominated only after 8 h, and those of p130 were first observed after 16 h of FGF treatment ( Fig. 5 A). |I:\*\*1SP3E3| |L:\*\*1SP3E3| |T:\*\*1SP3E2| |
|  |  |  |  | Annotation |  | 1SP3E3 | 1SP3E3 |  |  |  | 1SP3E2 |  |
|  |  |  |  | Evidence |  | 4 | 4 |  |  |  | 3 |  |
|  |  |  |  | Focus |  | 4 | 4 |  |  |  | 4 |  |
|  |  |  |  | Polarity |  | 7 | 7 |  |  |  | 7 |  |
| 247 | 247 | 9603890\_111 | Bootstrap values are shown in boxes with arrows indicating the relevant branch. |I:\*\*1SGP3E3| |L:\*\*1GP3E3| |T:\*\*1SP3E0| |
|  |  |  |  | Annotation |  | 1SGP3E3 | 1GP3E3 |  |  |  | 1SP3E0 |  |
|  |  |  |  | Evidence |  | 4 | 4 |  |  |  | 1 |  |
|  |  |  |  | Focus |  | 5 | 1 |  |  |  | 4 |  |
|  |  |  |  | Polarity |  | 7 | 7 |  |  |  | 7 |  |
| 248 | 248 | 11691915\_186 | SOX7 protein represses Wnt/beta-catenin-stimulated transcription |I:\*\*ERROR| |L:\*\*1SP3E2-| |T:\*\*1SN3E1| Recent data have suggested that different Xenopus SOX proteins could block beta-catenin/TCF-mediated gene activation by competing with TCF factors for binding to beta-catenin ( 33). |I:\*\*1SP3E2| |L:\*\*1SP1E2-| |T:\*\*1SN3E2| |
|  |  |  |  | Annotation |  | ERROR 1SP3E2 | 1SP3E2- 1SP1E2- |  |  |  | 1SN3E1 1SN3E2 |  |
|  |  |  |  | Evidence |  | -1000 3 | 3 3 |  |  |  | 2 3 |  |
|  |  |  |  | Focus |  | -1000 4 | 4 4 |  |  |  | 4 4 |  |
|  |  |  |  | Polarity |  | -1000 7 | 7 5 |  |  |  | 1 1 |  |
| 249 | 249 | 10678986\_105 | H233 is a mixed population of phase variants with (solid symbols) and without (open symbols) ChoP or Galalpha1-4Gal on their LPS ( 37). |I:\*\*1SGP3E2| |L:\*\*1SP3E2| |T:\*\*1SP3E2| |
|  |  |  |  | Annotation |  | 1SGP3E2 | 1SP3E2 |  |  |  | 1SP3E2 |  |
|  |  |  |  | Evidence |  | 3 | 3 |  |  |  | 3 |  |
|  |  |  |  | Focus |  | 5 | 4 |  |  |  | 4 |  |
|  |  |  |  | Polarity |  | 7 | 7 |  |  |  | 7 |  |
| 250 | 250 | 10809757\_43 | This proximal promoter region of the CTGF promoter conferred responsiveness to the SEAP reporter to TGF-beta (Fig. 7). |I:\*\*1SP3E3| |L:\*\*1SP3E3| |T:\*\*1SP3E2| |
|  |  |  |  | Annotation |  | 1SP3E3 | 1SP3E3 |  |  |  | 1SP3E2 |  |
|  |  |  |  | Evidence |  | 4 | 4 |  |  |  | 3 |  |
|  |  |  |  | Focus |  | 4 | 4 |  |  |  | 4 |  |
|  |  |  |  | Polarity |  | 7 | 7 |  |  |  | 7 |  |
| 251 | 251 | 12730091\_3 | VLDL, isolated from human serum |T:\*\*1MP3E3| dose- and time-dependently promoted proliferation |T:\*\*2SP3E3+| |I:\*\*1SP3E0| |L:\*\*1SP3E0+| |
|  |  |  |  | Annotation |  | 1SP3E0 1SP3E0 | 1SP3E0+ 1SP3E0+ |  |  |  | 1MP3E3 2SP3E3+ |  |
|  |  |  |  | Evidence |  | 1 1 | 1 1 |  |  |  | 4 4 |  |
|  |  |  |  | Focus |  | 4 4 | 4 4 |  |  |  | 2 4 |  |
|  |  |  |  | Polarity |  | 7 7 | 7 7 |  |  |  | 7 7 |  |
| 252 | 252 | 9799801\_57 | In other experiments a fixed quantity of sPLA 2 of 2.5 mug/ml was added to various concentrations of lipoproteins (0.25 - 1.0 mg/ml), or sPLA 2 in concentrations of 0.1, 0.5, 1.0, and 2.5 mug/ml was added to fixed concentration of lipoproteins (1 mg/ml). |I:\*\*1MP3E0| |L:\*\*1MP3E3| |T:\*\*1MP3E3| |
|  |  |  |  | Annotation |  | 1MP3E0 | 1MP3E3 |  |  |  | 1MP3E3 |  |
|  |  |  |  | Evidence |  | 1 | 4 |  |  |  | 4 |  |
|  |  |  |  | Focus |  | 2 | 2 |  |  |  | 2 |  |
|  |  |  |  | Polarity |  | 7 | 7 |  |  |  | 7 |  |
| 253 | 253 | 9826719\_130 | NSI"` and "`SI"` denote sequences from patient 129 with nonsyncytium-inducing and syncytium-inducing phenotypes, respectively. |I:\*\*1MGP3E3| |L:\*\*1MP3E3| |T:\*\*1MP3E3| |
|  |  |  |  | Annotation |  | 1MGP3E3 | 1MP3E3 |  |  |  | 1MP3E3 |  |
|  |  |  |  | Evidence |  | 4 | 4 |  |  |  | 4 |  |
|  |  |  |  | Focus |  | 3 | 2 |  |  |  | 2 |  |
|  |  |  |  | Polarity |  | 7 | 7 |  |  |  | 7 |  |
| 254 | 254 | 9539423\_88 | For this purpose, a library enriched for chromosome ends was constructed from the standard laboratory strain FB2. |I:\*\*1SGP3E0| |L:\*\*1MP3E3| |T:\*\*1MP3E3| |
|  |  |  |  | Annotation |  | 1SGP3E0 | 1MP3E3 |  |  |  | 1MP3E3 |  |
|  |  |  |  | Evidence |  | 1 | 4 |  |  |  | 4 |  |
|  |  |  |  | Focus |  | 5 | 2 |  |  |  | 2 |  |
|  |  |  |  | Polarity |  | 7 | 7 |  |  |  | 7 |  |
| 255 | 255 | 10604963\_9 | Whereas gender does not seem to be a factor in the anti-inflammatory efficacy of nonsteroidal anti-inflammatory drugs (NSAIDs) (Walker et al., 1994 ) |T:\*\*1SP3E2| |I:\*\*1SN2E2| |L:\*\*1SN3E2| it does seem relevant in the analgesic effects of this drug class (Walker and Carmody, 1998 ). |T:\*\*2SP3E2| |I:\*\*2SP2E2| |L:\*\*2SP3E2| |
|  |  |  |  | Annotation |  | 1SN2E2 2SP2E2 | 1SN3E2 2SP3E2 |  |  |  | 1SP3E2 2SP3E2 |  |
|  |  |  |  | Evidence |  | 3 3 | 3 3 |  |  |  | 3 3 |  |
|  |  |  |  | Focus |  | 4 4 | 4 4 |  |  |  | 4 4 |  |
|  |  |  |  | Polarity |  | 2 6 | 1 7 |  |  |  | 7 7 |  |
| 256 | 256 | 9486984\_238 | After 5 d of autologous MLTR with CD154-CLL or lacZ-CLL, the T cells were isolated by Ficoll density gradient centrifugation, washed extensively, and then cultured in media for 24 h. |I:\*\*1MP3E0| |L:\*\*1MP3E3| |T:\*\*1MP3E3| |
|  |  |  |  | Annotation |  | 1MP3E0 | 1MP3E3 |  |  |  | 1MP3E3 |  |
|  |  |  |  | Evidence |  | 1 | 4 |  |  |  | 4 |  |
|  |  |  |  | Focus |  | 2 | 2 |  |  |  | 2 |  |
|  |  |  |  | Polarity |  | 7 | 7 |  |  |  | 7 |  |
| 257 | 257 | 12805222\_336 | Li,L., Yuan,H., Weaver,C.D., Mao,J., Farr,G.H.,III, Sussman,D.J., Jonkers,J., Kimelman,D. and Wu,D. (1999a) |L:\*\*1GP3E3| Axin and Frat1 interact with Dvl and GSK, bridging Dvl to GSK in Wnt-mediated regulation of LEF-1. |L:\*\*2SP3E3| |I:\*\*1SP3E2| |T:\*\*1SP3E1| |
|  |  |  |  | Annotation |  | 1SP3E2 1SP3E2 | 1GP3E3 2SP3E3 |  |  |  | 1SP3E1 1SP3E1 |  |
|  |  |  |  | Evidence |  | 3 3 | 4 4 |  |  |  | 2 2 |  |
|  |  |  |  | Focus |  | 4 4 | 1 4 |  |  |  | 4 4 |  |
|  |  |  |  | Polarity |  | 7 7 | 7 7 |  |  |  | 7 7 |  |
| 258 | 258 | 10373560\_57 | To generate adenoviruses with the pAdEasy system ( 22), |I:\*\*1SGP3E2| pSmycMyt1deltaC63 and pSmycMyt1N238AdeltaC63 were constructed by subcloning the KpnI/ XhoI fragments of pCDNAmycMyt1deltaC63 and pCDNAmycMyt1N238AdeltaC63 ( 40), respectively, into the KpnI and XhoI sites of the shuttle vector pShuttle-CMV. |I:\*\*2MP3E2| |L:\*\*1MP3E3| |T:\*\*1MP3E3| |
|  |  |  |  | Annotation |  | 1SGP3E2 2MP3E2 | 1MP3E3 1MP3E3 |  |  |  | 1MP3E3 1MP3E3 |  |
|  |  |  |  | Evidence |  | 3 3 | 4 4 |  |  |  | 4 4 |  |
|  |  |  |  | Focus |  | 5 2 | 2 2 |  |  |  | 2 2 |  |
|  |  |  |  | Polarity |  | 7 7 | 7 7 |  |  |  | 7 7 |  |
| 259 | 259 | 10676812\_238 | After multireference alignment, multivariate statistical analysis, and automated hierarchical classification of the images, class averages of characteristic views were calculated with an improved signal-to-noise ratio. |I:\*\*1MP3E0| |L:\*\*1MP3E3| |T:\*\*1MP3E3| |
|  |  |  |  | Annotation |  | 1MP3E0 | 1MP3E3 |  |  |  | 1MP3E3 |  |
|  |  |  |  | Evidence |  | 1 | 4 |  |  |  | 4 |  |
|  |  |  |  | Focus |  | 2 | 2 |  |  |  | 2 |  |
|  |  |  |  | Polarity |  | 7 | 7 |  |  |  | 7 |  |
| 260 | 260 | 11679314\_82 | After concentration, the solvent extract was precipitated in rapidly stirred cold methanol. |I:\*\*1MP3E0| |L:\*\*1MP3E3| |T:\*\*1MP3E3| |
|  |  |  |  | Annotation |  | 1MP3E0 | 1MP3E3 |  |  |  | 1MP3E3 |  |
|  |  |  |  | Evidence |  | 1 | 4 |  |  |  | 4 |  |
|  |  |  |  | Focus |  | 2 | 2 |  |  |  | 2 |  |
|  |  |  |  | Polarity |  | 7 | 7 |  |  |  | 7 |  |
| 261 | 261 | 10915780\_188 | Since overexpression of Wnt in mammary gland epithelial cells causes tumors ( 21), |I:\*\*1SP3E2| |L:\*\*1SP3E2+| |T:\*\*1SP3E2| we examined whether activation of the Wnt pathway induces ILK activity |I:\*\*2SGP0E3| and elevates cyclin D1 protein levels in vivo in mammary tissue of MMTV-Wnt-1 tumors. |I:\*\*3SGP0E3+| |L:\*\*2SP0E3+| |T:\*\*2SP3E1+| |
|  |  |  |  | Annotation |  | 1SP3E2 2SGP0E3 3SGP0E3+ | 1SP3E2+ 2SP0E3+ 2SP0E3+ |  |  |  | 1SP3E2 2SP3E1+ 2SP3E1+ |  |
|  |  |  |  | Evidence |  | 3 4 4 | 3 4 4 |  |  |  | 3 2 2 |  |
|  |  |  |  | Focus |  | 4 5 5 | 4 4 4 |  |  |  | 4 4 4 |  |
|  |  |  |  | Polarity |  | 7 4 4 | 7 4 4 |  |  |  | 7 7 7 |  |
| 262 | 262 | 10749878\_244 | Furthermore, both calphostin C and the dominant negative PKC inhibit partially the Wnt-mediated Tcf/ef reporter activation. |I:\*\*1SP3E0| |L:\*\*1SP3E0-| |T:\*\*1SN3E1| |
|  |  |  |  | Annotation |  | 1SP3E0 | 1SP3E0- |  |  |  | 1SN3E1 |  |
|  |  |  |  | Evidence |  | 1 | 1 |  |  |  | 2 |  |
|  |  |  |  | Focus |  | 4 | 4 |  |  |  | 4 |  |
|  |  |  |  | Polarity |  | 7 | 7 |  |  |  | 1 |  |
| 263 | 263 | 11986553\_2 | Recent technologic advances in helical computed tomography, including rapid gantry rotation, multidetector arrays, and the use of electrocardiographic gating have resulted in image acquisition which parallels that of electron beam computed tomography. |I:\*\*1SMGP3E0| |L:\*\*1GP3E1| |T:\*\*1SP3E0| |
|  |  |  |  | Annotation |  | 1SMGP3E0 | 1GP3E1 |  |  |  | 1SP3E0 |  |
|  |  |  |  | Evidence |  | 1 | 2 |  |  |  | 1 |  |
|  |  |  |  | Focus |  | 7 | 1 |  |  |  | 4 |  |
|  |  |  |  | Polarity |  | 7 | 7 |  |  |  | 7 |  |
| 264 | 264 | 9673245\_93 | Briefly, developed TLC plates were dried for 30 min in a vacuum desiccator, fixed in 0.4% polyisobutylmethacrylate (Aldrich, Steinheim, Germany) in n-hexane (Merck) for 1.5 min, and dried as before. |I:\*\*1MP3E0| |L:\*\*1MP3E23| |T:\*\*1MP3E3| |
|  |  |  |  | Annotation |  | 1MP3E0 | 1MP3E23 |  |  |  | 1MP3E3 |  |
|  |  |  |  | Evidence |  | 1 | 3 |  |  |  | 4 |  |
|  |  |  |  | Focus |  | 2 | 2 |  |  |  | 2 |  |
|  |  |  |  | Polarity |  | 7 | 7 |  |  |  | 7 |  |
| 265 | 265 | 11070175\_323 | Bronchoalveolar lavage (BAL) fluids were collected by cannulating the trachea and lavaging the lungs with a 1.0 ml of ice-cold PBS. |I:\*\*1MP3E0| |L:\*\*1MP3E3| |T:\*\*1MP3E3| |
|  |  |  |  | Annotation |  | 1MP3E0 | 1MP3E3 |  |  |  | 1MP3E3 |  |
|  |  |  |  | Evidence |  | 1 | 4 |  |  |  | 4 |  |
|  |  |  |  | Focus |  | 2 | 2 |  |  |  | 2 |  |
|  |  |  |  | Polarity |  | 7 | 7 |  |  |  | 7 |  |
| 266 | 266 | 12781364\_154 | Furthermore, the acquisition of an imatinib resistance mutation of FIP1L1-PDGFR in a patient while on imatinib therapy provides convincing evidence that the fusion is the therapeutic target of imatinib in HES (Cools et al., 2003 ). |I:\*\*1SP3E2| |L:\*\*1SP3E2| |T:\*\*1SP3E2| |
|  |  |  |  | Annotation |  | 1SP3E2 | 1SP3E2 |  |  |  | 1SP3E2 |  |
|  |  |  |  | Evidence |  | 3 | 3 |  |  |  | 3 |  |
|  |  |  |  | Focus |  | 4 | 4 |  |  |  | 4 |  |
|  |  |  |  | Polarity |  | 7 | 7 |  |  |  | 7 |  |
| 267 | 267 | 9548592\_79 | Three separate aliquots of an RPE plasma membrane fraction (S2, 5 mg/ml, for method of preparation see reference ( 15)) were diluted 1:5 with one of the three sonicated preparations and incubated at room temperature for 20 min. |I:\*\*1MP3E3| |L:\*\*1MP3E23| |T:\*\*1MP3E3| |
|  |  |  |  | Annotation |  | 1MP3E3 | 1MP3E23 |  |  |  | 1MP3E3 |  |
|  |  |  |  | Evidence |  | 4 | 3 |  |  |  | 4 |  |
|  |  |  |  | Focus |  | 2 | 2 |  |  |  | 2 |  |
|  |  |  |  | Polarity |  | 7 | 7 |  |  |  | 7 |  |
| 268 | 268 | 11756460\_42 | The long and flexible segments of AP180 and epsin 1 may be primarily designed to concentrate adaptors and clathrin from the cytosol to this domain. |I:\*\*1SP1E3| |L:\*\*1SP1E0| |T:\*\*1SP2E3| |
|  |  |  |  | Annotation |  | 1SP1E3 | 1SP1E0 |  |  |  | 1SP2E3 |  |
|  |  |  |  | Evidence |  | 4 | 1 |  |  |  | 4 |  |
|  |  |  |  | Focus |  | 4 | 4 |  |  |  | 4 |  |
|  |  |  |  | Polarity |  | 5 | 5 |  |  |  | 6 |  |
| 269 | 269 | 11788579\_84 | Because both peptide antibodies show a similar cross-reactivity with the overexpressed Rieske proteins |T:\*\*1SP3E1| |I:\*\*1SMP3E3| |L:\*\*1SP3E0+| this result indicates a much lower content of PetC3 in our cytochrome b6 f complex preparation than of PetC1. |T:\*\*2SP2E1| |I:\*\*2SP3E3| |L:\*\*2SP1E0-| |
|  |  |  |  | Annotation |  | 1SMP3E3 2SP3E3 | 1SP3E0+ 2SP1E0- |  |  |  | 1SP3E1 2SP2E1 |  |
|  |  |  |  | Evidence |  | 4 4 | 1 1 |  |  |  | 2 2 |  |
|  |  |  |  | Focus |  | 6 4 | 4 4 |  |  |  | 4 4 |  |
|  |  |  |  | Polarity |  | 7 7 | 7 5 |  |  |  | 7 6 |  |
| 270 | 270 | 10848589\_175 | This observation is the first direct evidence |I:\*\*1SGP3E3| that the regression of the oviduct due to estrogen withdrawal is the result of apoptosis. |I:\*\*2SP3E0-| |L:\*\*1SP3E3| |T:\*\*1SP3E1| |
|  |  |  |  | Annotation |  | 1SGP3E3 2SP3E0- | 1SP3E3 1SP3E3 |  |  |  | 1SP3E1 1SP3E1 |  |
|  |  |  |  | Evidence |  | 4 1 | 4 4 |  |  |  | 2 2 |  |
|  |  |  |  | Focus |  | 5 4 | 4 4 |  |  |  | 4 4 |  |
|  |  |  |  | Polarity |  | 7 7 | 7 7 |  |  |  | 7 7 |  |
| 271 | 271 | 12611883\_37 | 1) APP will bind copper via the CuBD ( hatched box) in response to copper levels in the extracellular milieu and/or inside the cell. |I:\*\*1SP3E0| |L:\*\*1SP3E0| |T:\*\*1SP3E1| |
|  |  |  |  | Annotation |  | 1SP3E0 | 1SP3E0 |  |  |  | 1SP3E1 |  |
|  |  |  |  | Evidence |  | 1 | 1 |  |  |  | 2 |  |
|  |  |  |  | Focus |  | 4 | 4 |  |  |  | 4 |  |
|  |  |  |  | Polarity |  | 7 | 7 |  |  |  | 7 |  |
| 272 | 272 | 11163179\_405 | Many of these experiments were performed using the Ap-GAL4 line, |I:\*\*1MP3E3| which drives expression in three Apterous neurons in each abdominal hemisegment ( Figure 7). |I:\*\*2SP3E3| |L:\*\*1MSP3E3| |T:\*\*1MP3E3| |
|  |  |  |  | Annotation |  | 1MP3E3 2SP3E3 | 1MSP3E3 1MSP3E3 |  |  |  | 1MP3E3 1MP3E3 |  |
|  |  |  |  | Evidence |  | 4 4 | 4 4 |  |  |  | 4 4 |  |
|  |  |  |  | Focus |  | 2 4 | 6 6 |  |  |  | 2 2 |  |
|  |  |  |  | Polarity |  | 7 7 | 7 7 |  |  |  | 7 7 |  |
| 273 | 273 | 12098002\_7 | The extent of inhibition was dependent on both time and drug concentration. |I:\*\*1SP3E0| |L:\*\*1SP3E0| |T:\*\*1SP3E1| |
|  |  |  |  | Annotation |  | 1SP3E0 | 1SP3E0 |  |  |  | 1SP3E1 |  |
|  |  |  |  | Evidence |  | 1 | 1 |  |  |  | 2 |  |
|  |  |  |  | Focus |  | 4 | 4 |  |  |  | 4 |  |
|  |  |  |  | Polarity |  | 7 | 7 |  |  |  | 7 |  |
| 274 | 274 | 10548581\_2 | Bone marrow granulocytes do not constitutively express CD14 |I:\*\*1SN3E0| |L:\*\*1SN3E0| |T:\*\*1SN3E3| but can be stimulated by low doses of LPS in the absence of serum and then express an inducible form of LPS receptor (iLpsR). |I:\*\*2SP3E0| |L:\*\*2SP3E0+| |T:\*\*2SP3E0+| |
|  |  |  |  | Annotation |  | 1SN3E0 2SP3E0 | 1SN3E0 2SP3E0+ |  |  |  | 1SN3E3 2SP3E0+ |  |
|  |  |  |  | Evidence |  | 1 1 | 1 1 |  |  |  | 4 1 |  |
|  |  |  |  | Focus |  | 4 4 | 4 4 |  |  |  | 4 4 |  |
|  |  |  |  | Polarity |  | 1 7 | 1 7 |  |  |  | 1 7 |  |
| 275 | 275 | 9463492\_193 | Fig. 7 shows the percentage of decrease of NPo in the presence of 10 muM almitrine at two intracellular Ca2+ concentrations: 1 and 10 muM. |I:\*\*1SGP3E3-| |L:\*\*1GSP3E3| |T:\*\*1SP3E3-| |
|  |  |  |  | Annotation |  | 1SGP3E3- | 1GSP3E3 |  |  |  | 1SP3E3- |  |
|  |  |  |  | Evidence |  | 4 | 4 |  |  |  | 4 |  |
|  |  |  |  | Focus |  | 5 | 5 |  |  |  | 4 |  |
|  |  |  |  | Polarity |  | 7 | 7 |  |  |  | 7 |  |
| 276 | 276 | 12086862\_145 | We also tested the ability of another potential downstream effector, endothelial nitric oxide synthase (eNOS), |I:\*\*1SGP0E3| which is preferentially expressed in endothelial cells and known to participate in VEGF receptor signaling in cultured cells. |I:\*\*2SP3E3| |L:\*\*1GSP3E13| |T:\*\*1SP3E3| |
|  |  |  |  | Annotation |  | 1SGP0E3 2SP3E3 | 1GSP3E13 1GSP3E13 |  |  |  | 1SP3E3 1SP3E3 |  |
|  |  |  |  | Evidence |  | 4 4 | 2 2 |  |  |  | 4 4 |  |
|  |  |  |  | Focus |  | 5 4 | 5 5 |  |  |  | 4 4 |  |
|  |  |  |  | Polarity |  | 4 7 | 7 7 |  |  |  | 7 7 |  |
| 277 | 277 | 9390512\_137 | Suggestive evidence for linkage at the 5q21-31 locus was provided independently by two groups ( Straub et al. 1997 ; Schwab et al. 1997 ). |I:\*\*1SP2E2| |L:\*\*1SP1E2| |T:\*\*1GP3E2| |
|  |  |  |  | Annotation |  | 1SP2E2 | 1SP1E2 |  |  |  | 1GP3E2 |  |
|  |  |  |  | Evidence |  | 3 | 3 |  |  |  | 3 |  |
|  |  |  |  | Focus |  | 4 | 4 |  |  |  | 1 |  |
|  |  |  |  | Polarity |  | 6 | 5 |  |  |  | 7 |  |
| 278 | 278 | 11976332\_58 | All these peptides were found to serve as substrates, their K m values ranging from 10 to 40 muM (Table III). |I:\*\*1SP3E0| |L:\*\*1SP3E3| |T:\*\*1SP3E3| |
|  |  |  |  | Annotation |  | 1SP3E0 | 1SP3E3 |  |  |  | 1SP3E3 |  |
|  |  |  |  | Evidence |  | 1 | 4 |  |  |  | 4 |  |
|  |  |  |  | Focus |  | 4 | 4 |  |  |  | 4 |  |
|  |  |  |  | Polarity |  | 7 | 7 |  |  |  | 7 |  |
| 279 | 279 | 12177184\_27 | This suggests that the direct cell shape reorganization induced by Wnt signaling |L:\*\*1SP1E3+| could be mediated by GSK-3beta inhibition and its effects on MAPs phosphorylation and MT rearrangement. |L:\*\*2SP1E3-| |I:\*\*1SP1E0| |T:\*\*1SP3E3| |
|  |  |  |  | Annotation |  | 1SP1E0 1SP1E0 | 1SP1E3+ 2SP1E3- |  |  |  | 1SP3E3 1SP3E3 |  |
|  |  |  |  | Evidence |  | 1 1 | 4 4 |  |  |  | 4 4 |  |
|  |  |  |  | Focus |  | 4 4 | 4 4 |  |  |  | 4 4 |  |
|  |  |  |  | Polarity |  | 5 5 | 5 5 |  |  |  | 7 7 |  |
| 280 | 280 | 9390512\_8 | Long-standing impairment is observed in patients with schizophrenia. |I:\*\*1SP3E0| |L:\*\*1SP3E0| |T:\*\*1SP3E0| |
|  |  |  |  | Annotation |  | 1SP3E0 | 1SP3E0 |  |  |  | 1SP3E0 |  |
|  |  |  |  | Evidence |  | 1 | 1 |  |  |  | 1 |  |
|  |  |  |  | Focus |  | 4 | 4 |  |  |  | 4 |  |
|  |  |  |  | Polarity |  | 7 | 7 |  |  |  | 7 |  |
| 281 | 281 | 11792723\_172 | Excluding unincorporated [1,1-3H]hexadecanol, |I:\*\*1SN3E0| it is evident that the myelin fraction contained less than 10% of net lipid incorporated radioactivity between 30 and 240 min, and less than 3% at 240 min. |I:\*\*2SP3E0| |L:\*\*1SP3E3| |T:\*\*1SP3E3| |
|  |  |  |  | Annotation |  | 1SN3E0 2SP3E0 | 1SP3E3 1SP3E3 |  |  |  | 1SP3E3 1SP3E3 |  |
|  |  |  |  | Evidence |  | 1 1 | 4 4 |  |  |  | 4 4 |  |
|  |  |  |  | Focus |  | 4 4 | 4 4 |  |  |  | 4 4 |  |
|  |  |  |  | Polarity |  | 1 7 | 7 7 |  |  |  | 7 7 |  |
| 282 | 282 | 10454355\_263 | Further studies using multiple TRbeta mutations in inbred strains are needed |I:\*\*1SGP3E2| to better understand the contribution of the PV RTH transgene to body weight and exploratory activity, and possible interactions with background genes (Banbury Conference on Genetic Background in Mice 1997 ; Crawley et al. 1997 ). |I:\*\*2SGP0E2| |L:\*\*1GSP0E2| |T:\*\*1SP3E2| |
|  |  |  |  | Annotation |  | 1SGP3E2 2SGP0E2 | 1GSP0E2 1GSP0E2 |  |  |  | 1SP3E2 1SP3E2 |  |
|  |  |  |  | Evidence |  | 3 3 | 3 3 |  |  |  | 3 3 |  |
|  |  |  |  | Focus |  | 5 5 | 5 5 |  |  |  | 4 4 |  |
|  |  |  |  | Polarity |  | 7 4 | 4 4 |  |  |  | 7 7 |  |
| 283 | 283 | 11035751\_152 | It is therefore necessary to screen a variety of candidate genes, such as those for cytokines and members of the TLR signaling pathway, |I:\*\*1SGP3E0| to characterize the genetics of septic shock susceptibility. |I:\*\*2SGP0E0| |L:\*\*1GSP0E0| |T:\*\*1SP3E0| |
|  |  |  |  | Annotation |  | 1SGP3E0 2SGP0E0 | 1GSP0E0 1GSP0E0 |  |  |  | 1SP3E0 1SP3E0 |  |
|  |  |  |  | Evidence |  | 1 1 | 1 1 |  |  |  | 1 1 |  |
|  |  |  |  | Focus |  | 5 5 | 5 5 |  |  |  | 4 4 |  |
|  |  |  |  | Polarity |  | 7 4 | 4 4 |  |  |  | 7 7 |  |
| 284 | 284 | 11740944\_182 | Since high levels of Cut are found only in the absence of Vg |I:\*\*1SP3E3| and Vg-expressing cells show depressed levels of Cut, |I:\*\*2SP3E3-| |L:\*\*1SP3E0-| |T:\*\*1SP3E0| we analyzed the relationship between Vg and Cut expression |I:\*\*3SGP3E3| by inducing overexpression of each gene in all the notum myoblasts. |I:\*\*4MP3E3+| |L:\*\*2SP0E3+| |T:\*\*2MP3E3| |
|  |  |  |  | Annotation |  | 1SP3E3 2SP3E3- 3SGP3E3 4MP3E3+ | 1SP3E0- 1SP3E0- 2SP0E3+ 2SP0E3+ |  |  |  | 1SP3E0 1SP3E0 2MP3E3 2MP3E3 |  |
|  |  |  |  | Evidence |  | 4 4 4 4 | 1 1 4 4 |  |  |  | 1 1 4 4 |  |
|  |  |  |  | Focus |  | 4 4 5 2 | 4 4 4 4 |  |  |  | 4 4 2 2 |  |
|  |  |  |  | Polarity |  | 7 7 7 7 | 7 7 4 4 |  |  |  | 7 7 7 7 |  |
| 285 | 285 | 10402472\_346 | In addition, it is interesting to note that the published sequence of mouse E-cadherin contains several signal sequences in its cytoplasmic domain which are known to specify clathrin-coated endocytosis. |I:\*\*1SP3E3| |L:\*\*1SP3E1| |T:\*\*1SP3E1| |
|  |  |  |  | Annotation |  | 1SP3E3 | 1SP3E1 |  |  |  | 1SP3E1 |  |
|  |  |  |  | Evidence |  | 4 | 2 |  |  |  | 2 |  |
|  |  |  |  | Focus |  | 4 | 4 |  |  |  | 4 |  |
|  |  |  |  | Polarity |  | 7 | 7 |  |  |  | 7 |  |
| 286 | 286 | 9067302\_212 | These data show that the blocking action of nifedipine on the L-type current is more than 30 times strongerR than its action on other ionic currents. |I:\*\*1SP3E0| |L:\*\*1SP3E0| |T:\*\*1SP3E1| |
|  |  |  |  | Annotation |  | 1SP3E0 | 1SP3E0 |  |  |  | 1SP3E1 |  |
|  |  |  |  | Evidence |  | 1 | 1 |  |  |  | 2 |  |
|  |  |  |  | Focus |  | 4 | 4 |  |  |  | 4 |  |
|  |  |  |  | Polarity |  | 7 | 7 |  |  |  | 7 |  |
| 287 | 287 | 11928825\_3 | t-PA, u-PA, PAI-1 and PAI-2 have all been detected in GCF. |I:\*\*1SP3E0| |L:\*\*1SP3E1| |T:\*\*1SP3E1| |
|  |  |  |  | Annotation |  | 1SP3E0 | 1SP3E1 |  |  |  | 1SP3E1 |  |
|  |  |  |  | Evidence |  | 1 | 2 |  |  |  | 2 |  |
|  |  |  |  | Focus |  | 4 | 4 |  |  |  | 4 |  |
|  |  |  |  | Polarity |  | 7 | 7 |  |  |  | 7 |  |
| 288 | 288 | 8758897\_10 | First, all tumors are characterized by high level constitutive expression of the myc oncogene. |I:\*\*1SP3E0| |L:\*\*1MSP3E0+| |T:\*\*1SP3E1| |
|  |  |  |  | Annotation |  | 1SP3E0 | 1MSP3E0+ |  |  |  | 1SP3E1 |  |
|  |  |  |  | Evidence |  | 1 | 1 |  |  |  | 2 |  |
|  |  |  |  | Focus |  | 4 | 6 |  |  |  | 4 |  |
|  |  |  |  | Polarity |  | 7 | 7 |  |  |  | 7 |  |
| 289 | 289 | 11965113\_4 | RESULTS: Elevated intraocular pressure in thyroid-associated orbitopathy |L:\*\*1SP3E3+| observed in the three cases may involve different physiopathological abnormalities such as disturbances of venous circulation, compression by infiltrative muscles, and long corticosteroid use. |L:\*\*2SP1E3-| |I:\*\*1SP3E3| |T:\*\*1SP3E3| |
|  |  |  |  | Annotation |  | 1SP3E3 1SP3E3 | 1SP3E3+ 2SP1E3- |  |  |  | 1SP3E3 1SP3E3 |  |
|  |  |  |  | Evidence |  | 4 4 | 4 4 |  |  |  | 4 4 |  |
|  |  |  |  | Focus |  | 4 4 | 4 4 |  |  |  | 4 4 |  |
|  |  |  |  | Polarity |  | 7 7 | 7 5 |  |  |  | 7 7 |  |
| 290 | 290 | 10835414\_318 | The first generation hybrid line will be heterozygous for all these loci. |I:\*\*1SP3E0| |L:\*\*1SP3E0| |T:\*\*1MP3E3| |
|  |  |  |  | Annotation |  | 1SP3E0 | 1SP3E0 |  |  |  | 1MP3E3 |  |
|  |  |  |  | Evidence |  | 1 | 1 |  |  |  | 4 |  |
|  |  |  |  | Focus |  | 4 | 4 |  |  |  | 2 |  |
|  |  |  |  | Polarity |  | 7 | 7 |  |  |  | 7 |  |
| 291 | 291 | 9889190\_23 | In vitro, wild-type crmA is cleaved by caspase 1 |L:\*\*1SP3E2| |T:\*\*1SP3E2| (Komiyama et al., 1994 ; Xue and Horvitz, 1995 ) |I:\*\*1SP3E2| but not by the C. eleganscaspase Ced-3 (Xue and Horvitz, 1995 ). |I:\*\*2SN3E2| |L:\*\*2SN3E2| |T:\*\*2SN3E2| |
|  |  |  |  | Annotation |  | 1SP3E2 1SP3E2 2SN3E2 | 1SP3E2 2SN3E2 2SN3E2 |  |  |  | 1SP3E2 2SN3E2 2SN3E2 |  |
|  |  |  |  | Evidence |  | 3 3 3 | 3 3 3 |  |  |  | 3 3 3 |  |
|  |  |  |  | Focus |  | 4 4 4 | 4 4 4 |  |  |  | 4 4 4 |  |
|  |  |  |  | Polarity |  | 7 7 1 | 7 1 1 |  |  |  | 7 1 1 |  |
| 292 | 292 | 10625659\_4 | Following agonist-stimulated transmembrane signaling that increases the cytoplasmic Ca2+ level |T:\*\*1MP3E3| |I:\*\*1SP3E2+| cPLA2alpha undergoes translocation from the cytosol to the perinuclear envelope and endoplasmic reticulum ( , ), where many of the downstream eicosanoid-biosynthetic enzymes, including COX-1, COX-2, 5-LO, 5-LO-activating protein, and several terminal PG and leukotriene synthases, are located ( ). |T:\*\*2SP3E2| |I:\*\*2SP3E2| |L:\*\*1SP3E2| |
|  |  |  |  | Annotation |  | 1SP3E2+ 2SP3E2 | 1SP3E2 1SP3E2 |  |  |  | 1MP3E3 2SP3E2 |  |
|  |  |  |  | Evidence |  | 3 3 | 3 3 |  |  |  | 4 3 |  |
|  |  |  |  | Focus |  | 4 4 | 4 4 |  |  |  | 2 4 |  |
|  |  |  |  | Polarity |  | 7 7 | 7 7 |  |  |  | 7 7 |  |
| 293 | 293 | 10549630\_305 | The total numbers of OT-I and tumor cells at each location were determined |I:\*\*1SGP0E0| by multiplying the percent of cells in the population by the total number of cells recovered from each site. |I:\*\*2MP3E0| |L:\*\*1MP3E3| |T:\*\*1MP3E3| |
|  |  |  |  | Annotation |  | 1SGP0E0 2MP3E0 | 1MP3E3 1MP3E3 |  |  |  | 1MP3E3 1MP3E3 |  |
|  |  |  |  | Evidence |  | 1 1 | 4 4 |  |  |  | 4 4 |  |
|  |  |  |  | Focus |  | 5 2 | 2 2 |  |  |  | 2 2 |  |
|  |  |  |  | Polarity |  | 4 7 | 7 7 |  |  |  | 7 7 |  |
| 294 | 294 | 9067302\_25 | The aim of our experiments was to identify and clarify any inhibitory action of cilnidipine on the N-type VDCC. |I:\*\*1SGP0E3| |L:\*\*1GSP0E3| |T:\*\*1GP3E0| |
|  |  |  |  | Annotation |  | 1SGP0E3 | 1GSP0E3 |  |  |  | 1GP3E0 |  |
|  |  |  |  | Evidence |  | 4 | 4 |  |  |  | 1 |  |
|  |  |  |  | Focus |  | 5 | 5 |  |  |  | 1 |  |
|  |  |  |  | Polarity |  | 4 | 4 |  |  |  | 7 |  |
| 295 | 295 | 12201525\_9 | Measures of reproductive and ovarian function did not differ between Propionibacteria-treated and control cows. |I:\*\*1SN3E0| |L:\*\*1SN3E3| |T:\*\*1SN3E1| |
|  |  |  |  | Annotation |  | 1SN3E0 | 1SN3E3 |  |  |  | 1SN3E1 |  |
|  |  |  |  | Evidence |  | 1 | 4 |  |  |  | 2 |  |
|  |  |  |  | Focus |  | 4 | 4 |  |  |  | 4 |  |
|  |  |  |  | Polarity |  | 1 | 1 |  |  |  | 1 |  |
| 296 | 296 | 11296301\_13 | Efforts to identify molecular aberrations associated with the disease may be confounded by the subtle structural and cellular changes that occur and the polygenic nature of schizophrenia. |I:\*\*1SP1E3| |L:\*\*1SP1E1| |T:\*\*1GP3E1| |
|  |  |  |  | Annotation |  | 1SP1E3 | 1SP1E1 |  |  |  | 1GP3E1 |  |
|  |  |  |  | Evidence |  | 4 | 2 |  |  |  | 2 |  |
|  |  |  |  | Focus |  | 4 | 4 |  |  |  | 1 |  |
|  |  |  |  | Polarity |  | 5 | 5 |  |  |  | 7 |  |
| 297 | 297 | 10722617\_124 | E. coli intestinal colonization was determined twice a week by dilution of fecal samples on MacConkey agar. |I:\*\*1MP3E0| |L:\*\*1MP3E3| |T:\*\*1MP3E3| |
|  |  |  |  | Annotation |  | 1MP3E0 | 1MP3E3 |  |  |  | 1MP3E3 |  |
|  |  |  |  | Evidence |  | 1 | 4 |  |  |  | 4 |  |
|  |  |  |  | Focus |  | 2 | 2 |  |  |  | 2 |  |
|  |  |  |  | Polarity |  | 7 | 7 |  |  |  | 7 |  |
| 298 | 298 | 9712786\_79 | The plates were incubated for 5 days at 37 degrees C in 90% N2-5% O2-5% CO2, pinhead-sized colonies were enumerated, and bacterial density was expressed as the total number of CFU for the five biopsy samples (20 mg of tissue). |I:\*\*1MP3E0| |L:\*\*1MP3E3| |T:\*\*1MP3E3| |
|  |  |  |  | Annotation |  | 1MP3E0 | 1MP3E3 |  |  |  | 1MP3E3 |  |
|  |  |  |  | Evidence |  | 1 | 4 |  |  |  | 4 |  |
|  |  |  |  | Focus |  | 2 | 2 |  |  |  | 2 |  |
|  |  |  |  | Polarity |  | 7 | 7 |  |  |  | 7 |  |
| 299 | 299 | 10097073\_12 | Current evidence indicates that the Frizzled family of integral membrane proteins act as Wnt receptors. |I:\*\*1SP3E0| |L:\*\*1SP1E1| |T:\*\*1SP2E1| |
|  |  |  |  | Annotation |  | 1SP3E0 | 1SP1E1 |  |  |  | 1SP2E1 |  |
|  |  |  |  | Evidence |  | 1 | 2 |  |  |  | 2 |  |
|  |  |  |  | Focus |  | 4 | 4 |  |  |  | 4 |  |
|  |  |  |  | Polarity |  | 7 | 5 |  |  |  | 6 |  |
| 300 | 300 | 9717719\_125 | In principle, part of the 24S-hydroxycholesterol in the circulation may be transported in erythrocytes. |I:\*\*1SP3E0| |L:\*\*1SP1E0| |T:\*\*1SP1E0| |
|  |  |  |  | Annotation |  | 1SP3E0 | 1SP1E0 |  |  |  | 1SP1E0 |  |
|  |  |  |  | Evidence |  | 1 | 1 |  |  |  | 1 |  |
|  |  |  |  | Focus |  | 4 | 4 |  |  |  | 4 |  |
|  |  |  |  | Polarity |  | 7 | 5 |  |  |  | 5 |  |
| 301 | 301 | 12551955\_168 | These effects were blocked not only by pharmacologic inhibition of PI3Ks but also by a toxin ( C. difficile toxin B) that inhibits Rho GTPases. |I:\*\*1SP3E0| |L:\*\*1SP3E0-| |T:\*\*1SN3E1| |
|  |  |  |  | Annotation |  | 1SP3E0 | 1SP3E0- |  |  |  | 1SN3E1 |  |
|  |  |  |  | Evidence |  | 1 | 1 |  |  |  | 2 |  |
|  |  |  |  | Focus |  | 4 | 4 |  |  |  | 4 |  |
|  |  |  |  | Polarity |  | 7 | 7 |  |  |  | 1 |  |
| 302 | 302 | 9988738\_156 | The differences in signaling mediated between polycystin and EGF are demonstrated in Fig. 4 c. |I:\*\*1SGP3E3| EGF is a GSK-3 inhibitor |I:\*\*2SP3E3| that does not activate Wnt signaling. |I:\*\*3SN3E3| |L:\*\*1GSN3E3+| |T:\*\*1SP3E3| |
|  |  |  |  | Annotation |  | 1SGP3E3 2SP3E3 3SN3E3 | 1GSN3E3+ 1GSN3E3+ 1GSN3E3+ |  |  |  | 1SP3E3 1SP3E3 1SP3E3 |  |
|  |  |  |  | Evidence |  | 4 4 4 | 4 4 4 |  |  |  | 4 4 4 |  |
|  |  |  |  | Focus |  | 5 4 4 | 5 5 5 |  |  |  | 4 4 4 |  |
|  |  |  |  | Polarity |  | 7 7 1 | 1 1 1 |  |  |  | 7 7 7 |  |
| 303 | 303 | 10593912\_115 | To investigate possible differences between CHO and K562 cells, |I:\*\*1SGP0E3| we performed CHO cell adhesion studies on HvWf |I:\*\*2SGP3E3| using cells transfected with GPIbalpha, Ibbeta, and IX (CHOalphabetaIX). |I:\*\*3MP3E3| |L:\*\*1GMP3E3| |T:\*\*1MP3E3| |
|  |  |  |  | Annotation |  | 1SGP0E3 2SGP3E3 3MP3E3 | 1GMP3E3 1GMP3E3 1GMP3E3 |  |  |  | 1MP3E3 1MP3E3 1MP3E3 |  |
|  |  |  |  | Evidence |  | 4 4 4 | 4 4 4 |  |  |  | 4 4 4 |  |
|  |  |  |  | Focus |  | 5 5 2 | 3 3 3 |  |  |  | 2 2 2 |  |
|  |  |  |  | Polarity |  | 4 7 7 | 7 7 7 |  |  |  | 7 7 7 |  |
| 304 | 304 | 12058019\_37 | In highly confluent cultured cells, APC has been described in two major locations: in clusters near the basal surface and near the lateral plasma membrane ( Nathke et al., 1996; Reinacher-Schick and Gumbiner, 2001; Rosin-Arbesfeld et al., 2001). |I:\*\*1SP3E2| |L:\*\*1SP3E2| |T:\*\*1SP3E2| |
|  |  |  |  | Annotation |  | 1SP3E2 | 1SP3E2 |  |  |  | 1SP3E2 |  |
|  |  |  |  | Evidence |  | 3 | 3 |  |  |  | 3 |  |
|  |  |  |  | Focus |  | 4 | 4 |  |  |  | 4 |  |
|  |  |  |  | Polarity |  | 7 | 7 |  |  |  | 7 |  |
| 305 | 305 | 12072658\_12 | The transvaginal uterosacral ligament vaginal vault suspension is increasingly our procedure of choice for management of the apical defect |I:\*\*1MP3E3| due to its versatility, reduced postoperative morbidity and excellent short-term results. |I:\*\*2MGP3E3| |L:\*\*1GP3E0| |T:\*\*1MP3E3| |
|  |  |  |  | Annotation |  | 1MP3E3 2MGP3E3 | 1GP3E0 1GP3E0 |  |  |  | 1MP3E3 1MP3E3 |  |
|  |  |  |  | Evidence |  | 4 4 | 1 1 |  |  |  | 4 4 |  |
|  |  |  |  | Focus |  | 2 3 | 1 1 |  |  |  | 2 2 |  |
|  |  |  |  | Polarity |  | 7 7 | 7 7 |  |  |  | 7 7 |  |
| 306 | 306 | 9802902\_68 | For transient transfections, HeLa cells grown on glass coverslips (5 x 10^4^ cells/well) were transfected using calcium-phosphate precipitation. |I:\*\*1MP3E0| |L:\*\*1MP3E3| |T:\*\*1MP3E3| |
|  |  |  |  | Annotation |  | 1MP3E0 | 1MP3E3 |  |  |  | 1MP3E3 |  |
|  |  |  |  | Evidence |  | 1 | 4 |  |  |  | 4 |  |
|  |  |  |  | Focus |  | 2 | 2 |  |  |  | 2 |  |
|  |  |  |  | Polarity |  | 7 | 7 |  |  |  | 7 |  |
| 307 | 307 | 9870975\_138 | Comparisons between groups were done with one-way ANOVAs. |I:\*\*1MP3E0| |L:\*\*1MP3E3| |T:\*\*1MP3E3| |
|  |  |  |  | Annotation |  | 1MP3E0 | 1MP3E3 |  |  |  | 1MP3E3 |  |
|  |  |  |  | Evidence |  | 1 | 4 |  |  |  | 4 |  |
|  |  |  |  | Focus |  | 2 | 2 |  |  |  | 2 |  |
|  |  |  |  | Polarity |  | 7 | 7 |  |  |  | 7 |  |
| 308 | 308 | 10049358\_23 | These results raise the possibility that PGC precursors are induced by extracellular factors and/or cell interactions present locally at the junction between the extraembryonic ectoderm and epiblast. |I:\*\*1SP1E0| |L:\*\*1SP1E1+| |T:\*\*1SP1E3+| |
|  |  |  |  | Annotation |  | 1SP1E0 | 1SP1E1+ |  |  |  | 1SP1E3+ |  |
|  |  |  |  | Evidence |  | 1 | 2 |  |  |  | 4 |  |
|  |  |  |  | Focus |  | 4 | 4 |  |  |  | 4 |  |
|  |  |  |  | Polarity |  | 5 | 5 |  |  |  | 5 |  |
| 309 | 309 | 11149951\_33 | In the current study, we demonstrate a correlation between the D3 dopamine receptor on lymphocytes and schizophrenia |I:\*\*1SP3E3| |T:\*\*1SP3E3| and show a significant elevation (2- to 7-fold) in the mRNA level of D3, |I:\*\*2SP3E3+| |L:\*\*1SP3E3+| |T:\*\*2SP3E3+| but not of D4, |I:\*\*3SN3E3+| in schizophrenic patients. |I:\*\*4SP3E3| |L:\*\*2SN3E3| |T:\*\*3SN3E3| |
|  |  |  |  | Annotation |  | 1SP3E3 2SP3E3+ 3SN3E3+ 4SP3E3 | 1SP3E3+ 1SP3E3+ 2SN3E3 2SN3E3 |  |  |  | 1SP3E3 2SP3E3+ 3SN3E3 3SN3E3 |  |
|  |  |  |  | Evidence |  | 4 4 4 4 | 4 4 4 4 |  |  |  | 4 4 4 4 |  |
|  |  |  |  | Focus |  | 4 4 4 4 | 4 4 4 4 |  |  |  | 4 4 4 4 |  |
|  |  |  |  | Polarity |  | 7 7 1 7 | 7 7 1 1 |  |  |  | 7 7 1 1 |  |
| 310 | 310 | 10653712\_90 | For identification and isotopic composition GC-MS and IR Monitoring GC-MS (IRMGC-MS) |I:\*\*1SGP0E0| measurements were performed. |I:\*\*2SGP3E0| |L:\*\*1MP3E3| |T:\*\*1MP3E3| |
|  |  |  |  | Annotation |  | 1SGP0E0 2SGP3E0 | 1MP3E3 1MP3E3 |  |  |  | 1MP3E3 1MP3E3 |  |
|  |  |  |  | Evidence |  | 1 1 | 4 4 |  |  |  | 4 4 |  |
|  |  |  |  | Focus |  | 5 5 | 2 2 |  |  |  | 2 2 |  |
|  |  |  |  | Polarity |  | 4 7 | 7 7 |  |  |  | 7 7 |  |
| 311 | 311 | 12049706\_3 | PATIENTS AND METHOD: Seventy two males with hypercholesterolemia were administered three different 4-weeks duration diets. |I:\*\*1MP3E3| |L:\*\*1MP3E3| |T:\*\*1MP3E3| |
|  |  |  |  | Annotation |  | 1MP3E3 | 1MP3E3 |  |  |  | 1MP3E3 |  |
|  |  |  |  | Evidence |  | 4 | 4 |  |  |  | 4 |  |
|  |  |  |  | Focus |  | 2 | 2 |  |  |  | 2 |  |
|  |  |  |  | Polarity |  | 7 | 7 |  |  |  | 7 |  |
| 312 | 312 | 11562489\_11 | Administration of CGS21680, a selective A2A receptor agonist, into the subarachnoid space induced sleep |T:\*\*1SP3E2| ( 4), |I:\*\*1SP3E2| |L:\*\*1SP3E2| suggesting that PGD2-induced sleep is mediated by adenosine through the adenosine A2A receptor system. |I:\*\*2SP1E3| |L:\*\*2SP1E0| |T:\*\*2SP1E0| |
|  |  |  |  | Annotation |  | 1SP3E2 1SP3E2 2SP1E3 | 1SP3E2 1SP3E2 2SP1E0 |  |  |  | 1SP3E2 2SP1E0 2SP1E0 |  |
|  |  |  |  | Evidence |  | 3 3 4 | 3 3 1 |  |  |  | 3 1 1 |  |
|  |  |  |  | Focus |  | 4 4 4 | 4 4 4 |  |  |  | 4 4 4 |  |
|  |  |  |  | Polarity |  | 7 7 5 | 7 7 5 |  |  |  | 7 5 5 |  |
| 313 | 313 | 10373410\_15 | However, the presence of PKP-1 would overcome this limitation by providing additional desmoplakin-binding sites at the membrane. |I:\*\*1SP3E0| |L:\*\*1SP1E0| |T:\*\*1SP3E0| |
|  |  |  |  | Annotation |  | 1SP3E0 | 1SP1E0 |  |  |  | 1SP3E0 |  |
|  |  |  |  | Evidence |  | 1 | 1 |  |  |  | 1 |  |
|  |  |  |  | Focus |  | 4 | 4 |  |  |  | 4 |  |
|  |  |  |  | Polarity |  | 7 | 5 |  |  |  | 7 |  |
| 314 | 314 | 12359338\_2 | Amino acid sequence alignment revealed the putative Oct-binding sequence, RPYQGVRVKEPVKELL(K/R)RKRG, which is conserved among chicken, mouse and human. |I:\*\*1SP3E0| |L:\*\*1SP3E0| |T:\*\*1SP3E0| |
|  |  |  |  | Annotation |  | 1SP3E0 | 1SP3E0 |  |  |  | 1SP3E0 |  |
|  |  |  |  | Evidence |  | 1 | 1 |  |  |  | 1 |  |
|  |  |  |  | Focus |  | 4 | 4 |  |  |  | 4 |  |
|  |  |  |  | Polarity |  | 7 | 7 |  |  |  | 7 |  |
| 315 | 315 | 11333250\_583 | TRAUT, T. W., 1994 |L:\*\*1GP3E3| The functions and consensus motifs of nine types of peptide segments that form different types of nucleotide-binding sites. |L:\*\*2GSP0E3| |I:\*\*1SGP3E2| |T:\*\*1GP3E0| |
|  |  |  |  | Annotation |  | 1SGP3E2 1SGP3E2 | 1GP3E3 2GSP0E3 |  |  |  | 1GP3E0 1GP3E0 |  |
|  |  |  |  | Evidence |  | 3 3 | 4 4 |  |  |  | 1 1 |  |
|  |  |  |  | Focus |  | 5 5 | 1 5 |  |  |  | 1 1 |  |
|  |  |  |  | Polarity |  | 7 7 | 7 4 |  |  |  | 7 7 |  |
| 316 | 316 | 12220828\_3 | This work tested the extraction, recovery, and biostability of diethylenetriaminepentaacetate (DTPA) as a remediation agent for soils contaminated with metals. |I:\*\*1SGP0E3| |L:\*\*1GSP3E3| |T:\*\*1SP3E0| |
|  |  |  |  | Annotation |  | 1SGP0E3 | 1GSP3E3 |  |  |  | 1SP3E0 |  |
|  |  |  |  | Evidence |  | 4 | 4 |  |  |  | 1 |  |
|  |  |  |  | Focus |  | 5 | 5 |  |  |  | 4 |  |
|  |  |  |  | Polarity |  | 4 | 7 |  |  |  | 7 |  |
| 317 | 317 | 9067330\_90 | All images were recorded on Fuji color ASA400 negative film. |I:\*\*1MP3E0| |L:\*\*1MP3E3| |T:\*\*1MP3E3| |
|  |  |  |  | Annotation |  | 1MP3E0 | 1MP3E3 |  |  |  | 1MP3E3 |  |
|  |  |  |  | Evidence |  | 1 | 4 |  |  |  | 4 |  |
|  |  |  |  | Focus |  | 2 | 2 |  |  |  | 2 |  |
|  |  |  |  | Polarity |  | 7 | 7 |  |  |  | 7 |  |
| 318 | 318 | 10383463\_26 | In contrast, FRP, also designated as Frzb ( 18, 19), inhibits Wnt-induced axis duplication. |I:\*\*1SP3E0| |L:\*\*1SP2E3-| |T:\*\*1SN3E1| |
|  |  |  |  | Annotation |  | 1SP3E0 | 1SP2E3- |  |  |  | 1SN3E1 |  |
|  |  |  |  | Evidence |  | 1 | 4 |  |  |  | 2 |  |
|  |  |  |  | Focus |  | 4 | 4 |  |  |  | 4 |  |
|  |  |  |  | Polarity |  | 7 | 6 |  |  |  | 1 |  |
| 319 | 319 | 9596738\_197 | In C. albicans, the nature and intensity of stimuli combined with the differential activities of these signal transduction pathways may help determine |I:\*\*1SGP1E0| whether cells undergo pseudohyphal or true hyphal differentiation. |I:\*\*2SP0E0| |L:\*\*1SP0E0| |T:\*\*1SP2E0| |
|  |  |  |  | Annotation |  | 1SGP1E0 2SP0E0 | 1SP0E0 1SP0E0 |  |  |  | 1SP2E0 1SP2E0 |  |
|  |  |  |  | Evidence |  | 1 1 | 1 1 |  |  |  | 1 1 |  |
|  |  |  |  | Focus |  | 5 4 | 4 4 |  |  |  | 4 4 |  |
|  |  |  |  | Polarity |  | 5 4 | 4 4 |  |  |  | 6 6 |  |
| 320 | 320 | 9679057\_17 | Although basic aspects of the G1 regulatory events are conserved in yeast and higher eukaryotes, |L:\*\*1SP3E0| it is clear that the requirements of cell growth control that couple proliferation with cell differentiation have added additional complexity. |L:\*\*2SP3E2+| |I:\*\*1SP3E0| |T:\*\*1SP3E1| |
|  |  |  |  | Annotation |  | 1SP3E0 1SP3E0 | 1SP3E0 2SP3E2+ |  |  |  | 1SP3E1 1SP3E1 |  |
|  |  |  |  | Evidence |  | 1 1 | 1 3 |  |  |  | 2 2 |  |
|  |  |  |  | Focus |  | 4 4 | 4 4 |  |  |  | 4 4 |  |
|  |  |  |  | Polarity |  | 7 7 | 7 7 |  |  |  | 7 7 |  |
| 321 | 321 | 9837930\_186 | Noteworthy is that the majority of beta-cells oscillate at the control glucose concentration of 11 mM. |I:\*\*1SP3E0| |L:\*\*1SP3E0| |T:\*\*1SP3E1| |
|  |  |  |  | Annotation |  | 1SP3E0 | 1SP3E0 |  |  |  | 1SP3E1 |  |
|  |  |  |  | Evidence |  | 1 | 1 |  |  |  | 2 |  |
|  |  |  |  | Focus |  | 4 | 4 |  |  |  | 4 |  |
|  |  |  |  | Polarity |  | 7 | 7 |  |  |  | 7 |  |
| 322 | 322 | 11781319\_68 | Numbers represent the corresponding constructs in B. , untransfected cells; lane GFP, cells transfected with empty GFP vector. |I:\*\*1SGP3E3| |L:\*\*1GP3E3| |T:\*\*1MP3E3| |
|  |  |  |  | Annotation |  | 1SGP3E3 | 1GP3E3 |  |  |  | 1MP3E3 |  |
|  |  |  |  | Evidence |  | 4 | 4 |  |  |  | 4 |  |
|  |  |  |  | Focus |  | 5 | 1 |  |  |  | 2 |  |
|  |  |  |  | Polarity |  | 7 | 7 |  |  |  | 7 |  |
| 323 | 323 | 12181332\_3 | Associated with these events, reduction in oxygen consumption and in mitochondrial membrane potential was found. |I:\*\*1SP3E0-| |L:\*\*1SP3E3-| |T:\*\*1SP3E3| |
|  |  |  |  | Annotation |  | 1SP3E0- | 1SP3E3- |  |  |  | 1SP3E3 |  |
|  |  |  |  | Evidence |  | 1 | 4 |  |  |  | 4 |  |
|  |  |  |  | Focus |  | 4 | 4 |  |  |  | 4 |  |
|  |  |  |  | Polarity |  | 7 | 7 |  |  |  | 7 |  |
| 324 | 324 | 11545071\_22 | It was suggested that S180A (amino acid replacement S A at site 180), H197Y, Y277F, T285A, and A308S shift the lambdamax of the LWS/MWS pigments toward green by 7, 28, 7, 15, and 16 nm, respectively, and the reverse changes toward red by the same amounts ( Y OKOYAMA and RADLWIMMER 1999 ). |I:\*\*1SP1E2| |L:\*\*1SP1E2| |T:\*\*1SP1E1| |
|  |  |  |  | Annotation |  | 1SP1E2 | 1SP1E2 |  |  |  | 1SP1E1 |  |
|  |  |  |  | Evidence |  | 3 | 3 |  |  |  | 2 |  |
|  |  |  |  | Focus |  | 4 | 4 |  |  |  | 4 |  |
|  |  |  |  | Polarity |  | 5 | 5 |  |  |  | 5 |  |
| 325 | 325 | 10473520\_134 | In Japan, the high prevalence (29.8%) of BDV p24 RNA in PBMC from 57 healthy horses was demonstrated, and about 60% of the BDV RNA-positive animals showed seropositivity by WB with GST-BDV p24 fusion protein ( 14). |I:\*\*1SP3E2| |L:\*\*1SP3E2| |T:\*\*1SP3E2| |
|  |  |  |  | Annotation |  | 1SP3E2 | 1SP3E2 |  |  |  | 1SP3E2 |  |
|  |  |  |  | Evidence |  | 3 | 3 |  |  |  | 3 |  |
|  |  |  |  | Focus |  | 4 | 4 |  |  |  | 4 |  |
|  |  |  |  | Polarity |  | 7 | 7 |  |  |  | 7 |  |
| 326 | 326 | 12191897\_2 | The study involved an abattoir survey of 1316 pigs at a slaughter slab in Lusaka and two field surveys in villages in Southern and Eastern provinces. |I:\*\*1SGP3E0| |L:\*\*1MP3E3| |T:\*\*1MP3E1| |
|  |  |  |  | Annotation |  | 1SGP3E0 | 1MP3E3 |  |  |  | 1MP3E1 |  |
|  |  |  |  | Evidence |  | 1 | 4 |  |  |  | 2 |  |
|  |  |  |  | Focus |  | 5 | 2 |  |  |  | 2 |  |
|  |  |  |  | Polarity |  | 7 | 7 |  |  |  | 7 |  |
| 327 | 327 | 9657144\_166 | These 9 Ang resolution phases should make it possible to find the heavy atom sites in derivatives containing large numbers of single heavy atoms by difference electron density maps. |I:\*\*1SGP2E0| |L:\*\*1GSP0E0| |T:\*\*1MP2E0| |
|  |  |  |  | Annotation |  | 1SGP2E0 | 1GSP0E0 |  |  |  | 1MP2E0 |  |
|  |  |  |  | Evidence |  | 1 | 1 |  |  |  | 1 |  |
|  |  |  |  | Focus |  | 5 | 5 |  |  |  | 2 |  |
|  |  |  |  | Polarity |  | 6 | 4 |  |  |  | 6 |  |
| 328 | 328 | 9822747\_143 | Scale bar (shown in B for A, B): 150 mum. |I:\*\*1SGP3E3| |L:\*\*1GP3E3| |T:\*\*1GP3E3| |
|  |  |  |  | Annotation |  | 1SGP3E3 | 1GP3E3 |  |  |  | 1GP3E3 |  |
|  |  |  |  | Evidence |  | 4 | 4 |  |  |  | 4 |  |
|  |  |  |  | Focus |  | 5 | 1 |  |  |  | 1 |  |
|  |  |  |  | Polarity |  | 7 | 7 |  |  |  | 7 |  |
| 329 | 329 | 9230313\_139 | Siamois is a homeobox gene whose expression is specifically activated by Wnt signaling |I:\*\*1SP3E2| and which appears to mediate the effects of the Wnt pathway on axis formation (Lemaire et al., 1995 ; Carnac et al., 1996 ; Fagotto et al., 1997 ). |I:\*\*2SP1E2| |L:\*\*1SP3E2+| |T:\*\*1SP3E2+| |
|  |  |  |  | Annotation |  | 1SP3E2 2SP1E2 | 1SP3E2+ 1SP3E2+ |  |  |  | 1SP3E2+ 1SP3E2+ |  |
|  |  |  |  | Evidence |  | 3 3 | 3 3 |  |  |  | 3 3 |  |
|  |  |  |  | Focus |  | 4 4 | 4 4 |  |  |  | 4 4 |  |
|  |  |  |  | Polarity |  | 7 5 | 7 7 |  |  |  | 7 7 |  |
| 330 | 330 | 11566983\_221 | Infection with this clone was allowed to persist for 7 months, and then postinfection clones were recovered. |I:\*\*1MP3E0| |L:\*\*1MP3E3| |T:\*\*1MP3E3| |
|  |  |  |  | Annotation |  | 1MP3E0 | 1MP3E3 |  |  |  | 1MP3E3 |  |
|  |  |  |  | Evidence |  | 1 | 4 |  |  |  | 4 |  |
|  |  |  |  | Focus |  | 2 | 2 |  |  |  | 2 |  |
|  |  |  |  | Polarity |  | 7 | 7 |  |  |  | 7 |  |
| 331 | 331 | 9744873\_289 | Regulatory subunit composition may also modulate PP2A by targeting the phosphatase to select compartments. |I:\*\*1SP1E0| |L:\*\*1SP1E0| |T:\*\*1SP1E0| |
|  |  |  |  | Annotation |  | 1SP1E0 | 1SP1E0 |  |  |  | 1SP1E0 |  |
|  |  |  |  | Evidence |  | 1 | 1 |  |  |  | 1 |  |
|  |  |  |  | Focus |  | 4 | 4 |  |  |  | 4 |  |
|  |  |  |  | Polarity |  | 5 | 5 |  |  |  | 5 |  |
| 332 | 332 | 12163406\_141 | Group1: genes requiring both WntP/beta-catenin and VegT pathways for expression |I:\*\*ERROR| |L:\*\*1GP3E3| |T:\*\*1GP3E3| Fig. 1A shows those genes that were not expressed if either beta-catenin or VegT was depleted. |I:\*\*1SGP3E3| |L:\*\*1SP3E3-| |T:\*\*1SN3E3| |
|  |  |  |  | Annotation |  | ERROR 1SGP3E3 | 1GP3E3 1SP3E3- |  |  |  | 1GP3E3 1SN3E3 |  |
|  |  |  |  | Evidence |  | -1000 4 | 4 4 |  |  |  | 4 4 |  |
|  |  |  |  | Focus |  | -1000 5 | 1 4 |  |  |  | 1 4 |  |
|  |  |  |  | Polarity |  | -1000 7 | 7 7 |  |  |  | 7 1 |  |
| 333 | 333 | 9521135\_197 | VacA binding can be inhibited by antibodies reacting exclusively with the 58-kDa fragment ( 11). |I:\*\*1SP3E2| |L:\*\*1SN3E2-| |T:\*\*1SP3E2| |
|  |  |  |  | Annotation |  | 1SP3E2 | 1SN3E2- |  |  |  | 1SP3E2 |  |
|  |  |  |  | Evidence |  | 3 | 3 |  |  |  | 3 |  |
|  |  |  |  | Focus |  | 4 | 4 |  |  |  | 4 |  |
|  |  |  |  | Polarity |  | 7 | 1 |  |  |  | 7 |  |
| 334 | 334 | 9570815\_130 | The analysis in Figure 1 B suggests that in five cases the side cuts were largely successful. |I:\*\*1MSP3E3| |L:\*\*1SP1E3| |T:\*\*1SP3E3| |
|  |  |  |  | Annotation |  | 1MSP3E3 | 1SP1E3 |  |  |  | 1SP3E3 |  |
|  |  |  |  | Evidence |  | 4 | 4 |  |  |  | 4 |  |
|  |  |  |  | Focus |  | 6 | 4 |  |  |  | 4 |  |
|  |  |  |  | Polarity |  | 7 | 5 |  |  |  | 7 |  |
| 335 | 335 | 9660865\_84 | Intensities of signals were measured by NIH Image 1.58 software. |I:\*\*1MP3E0| |L:\*\*1MP3E3| |T:\*\*1MP3E3| |
|  |  |  |  | Annotation |  | 1MP3E0 | 1MP3E3 |  |  |  | 1MP3E3 |  |
|  |  |  |  | Evidence |  | 1 | 4 |  |  |  | 4 |  |
|  |  |  |  | Focus |  | 2 | 2 |  |  |  | 2 |  |
|  |  |  |  | Polarity |  | 7 | 7 |  |  |  | 7 |  |
| 336 | 336 | 11412995\_275 | The SV40-based DNA replication assay and the micrococcal nuclease digestion of products were performed as described [5 . |I:\*\*1MP3E2| |L:\*\*1SP3E23| |T:\*\*1MP3E3| |
|  |  |  |  | Annotation |  | 1MP3E2 | 1SP3E23 |  |  |  | 1MP3E3 |  |
|  |  |  |  | Evidence |  | 3 | 3 |  |  |  | 4 |  |
|  |  |  |  | Focus |  | 2 | 4 |  |  |  | 2 |  |
|  |  |  |  | Polarity |  | 7 | 7 |  |  |  | 7 |  |
| 337 | 337 | 12191480\_266 | Strikingly, the residues that interact with the C-terminal tail appear to be highly conserved in all three yeast proteins. |I:\*\*1SP3E0| |L:\*\*1SP2E0| |T:\*\*1SP2E1| |
|  |  |  |  | Annotation |  | 1SP3E0 | 1SP2E0 |  |  |  | 1SP2E1 |  |
|  |  |  |  | Evidence |  | 1 | 1 |  |  |  | 2 |  |
|  |  |  |  | Focus |  | 4 | 4 |  |  |  | 4 |  |
|  |  |  |  | Polarity |  | 7 | 6 |  |  |  | 6 |  |
| 338 | 338 | 9390512\_159 | For example, if an allele that occurs naturally in 10% of the population doubles an individual's risk to disease, |I:\*\*1SP0E3| then one would need more than 5000 affected sib-pairs to detect linkage at lod > 3. |I:\*\*2SP3E3| |L:\*\*1SP3E0| |T:\*\*1SP3E0| |
|  |  |  |  | Annotation |  | 1SP0E3 2SP3E3 | 1SP3E0 1SP3E0 |  |  |  | 1SP3E0 1SP3E0 |  |
|  |  |  |  | Evidence |  | 4 4 | 1 1 |  |  |  | 1 1 |  |
|  |  |  |  | Focus |  | 4 4 | 4 4 |  |  |  | 4 4 |  |
|  |  |  |  | Polarity |  | 4 7 | 7 7 |  |  |  | 7 7 |  |
| 339 | 339 | 9789058\_56 | The Expression of CAR Is Sufficient to Confer Full Susceptibility of Lymphocytes to Adenovirus Transduction. |I:\*\*1SP3E0| |L:\*\*1SP3E0| |T:\*\*1SP3E0| |
|  |  |  |  | Annotation |  | 1SP3E0 | 1SP3E0 |  |  |  | 1SP3E0 |  |
|  |  |  |  | Evidence |  | 1 | 1 |  |  |  | 1 |  |
|  |  |  |  | Focus |  | 4 | 4 |  |  |  | 4 |  |
|  |  |  |  | Polarity |  | 7 | 7 |  |  |  | 7 |  |
| 340 | 340 | 9560277\_95 | However, these laminar differences did not achieve statistical significance. |I:\*\*1SN3E0| |L:\*\*1SN3E0| |T:\*\*1SP3E0| |
|  |  |  |  | Annotation |  | 1SN3E0 | 1SN3E0 |  |  |  | 1SP3E0 |  |
|  |  |  |  | Evidence |  | 1 | 1 |  |  |  | 1 |  |
|  |  |  |  | Focus |  | 4 | 4 |  |  |  | 4 |  |
|  |  |  |  | Polarity |  | 1 | 1 |  |  |  | 7 |  |
| 341 | 341 | 9430686\_122 | Base changes in the wobble base position that do not change amino acids will not be detected. |I:\*\*1SN3E0| |L:\*\*1MN3E0| |T:\*\*1MN3E3| |
|  |  |  |  | Annotation |  | 1SN3E0 | 1MN3E0 |  |  |  | 1MN3E3 |  |
|  |  |  |  | Evidence |  | 1 | 1 |  |  |  | 4 |  |
|  |  |  |  | Focus |  | 4 | 2 |  |  |  | 2 |  |
|  |  |  |  | Polarity |  | 1 | 1 |  |  |  | 1 |  |
| 342 | 342 | 11526109\_86 | Additionally, the R28C mutation reduced the plasma membrane association of IRS-1 PH domain-GFP by 67% in both the unstimulated |L:\*\*1SP3E0-| and insulin-stimulated states. |L:\*\*2SP3E0+| |I:\*\*1SP3E0-| |T:\*\*1SP3E3-| |
|  |  |  |  | Annotation |  | 1SP3E0- 1SP3E0- | 1SP3E0- 2SP3E0+ |  |  |  | 1SP3E3- 1SP3E3- |  |
|  |  |  |  | Evidence |  | 1 1 | 1 1 |  |  |  | 4 4 |  |
|  |  |  |  | Focus |  | 4 4 | 4 4 |  |  |  | 4 4 |  |
|  |  |  |  | Polarity |  | 7 7 | 7 7 |  |  |  | 7 7 |  |
| 343 | 343 | 9512517\_57 | These relatively mild changes are consistent with the temperature-sensitive and hypomorphic natures of the mutant phenotypes (see Discussion). |I:\*\*1SP3E3| |L:\*\*1GSP3E3| |T:\*\*1SP1E0-| |
|  |  |  |  | Annotation |  | 1SP3E3 | 1GSP3E3 |  |  |  | 1SP1E0- |  |
|  |  |  |  | Evidence |  | 4 | 4 |  |  |  | 1 |  |
|  |  |  |  | Focus |  | 4 | 5 |  |  |  | 4 |  |
|  |  |  |  | Polarity |  | 7 | 7 |  |  |  | 5 |  |
| 344 | 344 | 11381085\_140 | Inhibition of caspase or cathepsin B activities does not affect TNF-induced NF-kappaB activation |L:\*\*1SN3E0+| or binding, internalization, |L:\*\*2SN3E0| or degradation of TNF. |L:\*\*3SN3E0-| |I:\*\*1SN3E0| |T:\*\*1SP2E0| |
|  |  |  |  | Annotation |  | 1SN3E0 1SN3E0 1SN3E0 | 1SN3E0+ 2SN3E0 3SN3E0- |  |  |  | 1SP2E0 1SP2E0 1SP2E0 |  |
|  |  |  |  | Evidence |  | 1 1 1 | 1 1 1 |  |  |  | 1 1 1 |  |
|  |  |  |  | Focus |  | 4 4 4 | 4 4 4 |  |  |  | 4 4 4 |  |
|  |  |  |  | Polarity |  | 1 1 1 | 1 1 1 |  |  |  | 6 6 6 |  |
| 345 | 345 | 11156972\_124 | This combination of extremely closely and comparatively distantly related alleles |I:\*\*1SGP3E3| yielded a distinctly bimodal frequency distribution of pairwise sequence divergences ( Fig 2A). |I:\*\*2SP3E3| |L:\*\*1SP3E3| |T:\*\*1SP3E3| |
|  |  |  |  | Annotation |  | 1SGP3E3 2SP3E3 | 1SP3E3 1SP3E3 |  |  |  | 1SP3E3 1SP3E3 |  |
|  |  |  |  | Evidence |  | 4 4 | 4 4 |  |  |  | 4 4 |  |
|  |  |  |  | Focus |  | 5 4 | 4 4 |  |  |  | 4 4 |  |
|  |  |  |  | Polarity |  | 7 7 | 7 7 |  |  |  | 7 7 |  |
| 346 | 346 | 11779503\_2 | The crystal structure of a phosphorylated Smad2, at 1.8 A resolution, reveals the formation of a homotrimer mediated by the C-terminal phosphoserine (pSer) residues. |I:\*\*1SP3E0| |L:\*\*1SP3E0| |T:\*\*1SP3E0| |
|  |  |  |  | Annotation |  | 1SP3E0 | 1SP3E0 |  |  |  | 1SP3E0 |  |
|  |  |  |  | Evidence |  | 1 | 1 |  |  |  | 1 |  |
|  |  |  |  | Focus |  | 4 | 4 |  |  |  | 4 |  |
|  |  |  |  | Polarity |  | 7 | 7 |  |  |  | 7 |  |
| 347 | 347 | 9529101\_49 | Fixed sections were washed in 0.1% Triton X-100 in phosphate-buffered saline (PBS) and then incubated with 10 mM glucose-1 mM NaN3-0.4 U of glucose oxidase per ml (Sigma) in PBS for 15 min at 37 degrees C to block endogenous peroxidase activity. |I:\*\*1MP3E0| |L:\*\*1MP3E3| |T:\*\*1MP3E3| |
|  |  |  |  | Annotation |  | 1MP3E0 | 1MP3E3 |  |  |  | 1MP3E3 |  |
|  |  |  |  | Evidence |  | 1 | 4 |  |  |  | 4 |  |
|  |  |  |  | Focus |  | 2 | 2 |  |  |  | 2 |  |
|  |  |  |  | Polarity |  | 7 | 7 |  |  |  | 7 |  |
| 348 | 348 | 11092858\_57 | Bacteria were cultured for assay at 37 degrees C with shaking. |I:\*\*1MP3E0| |L:\*\*1MP3E3| |T:\*\*1MP3E3| |
|  |  |  |  | Annotation |  | 1MP3E0 | 1MP3E3 |  |  |  | 1MP3E3 |  |
|  |  |  |  | Evidence |  | 1 | 4 |  |  |  | 4 |  |
|  |  |  |  | Focus |  | 2 | 2 |  |  |  | 2 |  |
|  |  |  |  | Polarity |  | 7 | 7 |  |  |  | 7 |  |
| 349 | 349 | 12361601\_115 | NC cells began emigrating after 8-9 hr (Figures 3E and 3H). |I:\*\*1SP3E3| |L:\*\*1SP3E3| |T:\*\*1SP3E3| |
|  |  |  |  | Annotation |  | 1SP3E3 | 1SP3E3 |  |  |  | 1SP3E3 |  |
|  |  |  |  | Evidence |  | 4 | 4 |  |  |  | 4 |  |
|  |  |  |  | Focus |  | 4 | 4 |  |  |  | 4 |  |
|  |  |  |  | Polarity |  | 7 | 7 |  |  |  | 7 |  |
| 350 | 350 | 9710604\_92 | After five washes of 5 min each in PBS and two washes in water, the stained cells were mounted on glass slides with a PBS-glycerol solution (1:9, vol/vol) and observed under a fluorescence microscope. |I:\*\*1MP3E0| |L:\*\*1MP3E3| |T:\*\*1MP3E3| |
|  |  |  |  | Annotation |  | 1MP3E0 | 1MP3E3 |  |  |  | 1MP3E3 |  |
|  |  |  |  | Evidence |  | 1 | 4 |  |  |  | 4 |  |
|  |  |  |  | Focus |  | 2 | 2 |  |  |  | 2 |  |
|  |  |  |  | Polarity |  | 7 | 7 |  |  |  | 7 |  |
| 351 | 351 | 7836373\_36 | However, a slow onset inhibition is apparent |T:\*\*1SN2E3| when CaCl is added along with TG ( Fig. 3 ). |T:\*\*2MP3E3| |I:\*\*1SP3E3| |L:\*\*1SP3E3| |
|  |  |  |  | Annotation |  | 1SP3E3 1SP3E3 | 1SP3E3 1SP3E3 |  |  |  | 1SN2E3 2MP3E3 |  |
|  |  |  |  | Evidence |  | 4 4 | 4 4 |  |  |  | 4 4 |  |
|  |  |  |  | Focus |  | 4 4 | 4 4 |  |  |  | 4 2 |  |
|  |  |  |  | Polarity |  | 7 7 | 7 7 |  |  |  | 2 7 |  |
| 352 | 352 | 11861647\_11 | Conversely, Wnt signaling blocks GSK-3s activity toward non-primed substrates |L:\*\*1SP3E0-| without affecting its activity toward primed substrates. |L:\*\*2SN3E0| |I:\*\*1SP3E0| |T:\*\*1SN3E0| |
|  |  |  |  | Annotation |  | 1SP3E0 1SP3E0 | 1SP3E0- 2SN3E0 |  |  |  | 1SN3E0 1SN3E0 |  |
|  |  |  |  | Evidence |  | 1 1 | 1 1 |  |  |  | 1 1 |  |
|  |  |  |  | Focus |  | 4 4 | 4 4 |  |  |  | 4 4 |  |
|  |  |  |  | Polarity |  | 7 7 | 7 1 |  |  |  | 1 1 |  |
| 353 | 353 | 9744886\_294 | However, this death was induced by treatment with 2-deoxyadenosine |T:\*\*1MP3E1| a precursor of dATP, |I:\*\*1SP3E0| |L:\*\*1SP3E0+| so the increase in its concentration may not be physiologically relevant to apoptosis. |T:\*\*1SN1E1| |I:\*\*1SN1E0| |L:\*\*1SN3E3-| |
|  |  |  |  | Annotation |  | 1SP3E0 1SP3E0 1SN1E0 | 1SP3E0+ 1SP3E0+ 1SN3E3- |  |  |  | 1MP3E1 1SN1E1 1SN1E1 |  |
|  |  |  |  | Evidence |  | 1 1 1 | 1 1 4 |  |  |  | 2 2 2 |  |
|  |  |  |  | Focus |  | 4 4 4 | 4 4 4 |  |  |  | 2 4 4 |  |
|  |  |  |  | Polarity |  | 7 7 3 | 7 7 1 |  |  |  | 7 3 3 |  |
| 354 | 354 | 12364586\_14 | Schizophrenia affects almost 1% of the world's population, with a similar prevalence throughout diverse ethnic groups ( 1). |I:\*\*1SP3E2| |L:\*\*1SP3E2| |T:\*\*1SP3E2| |
|  |  |  |  | Annotation |  | 1SP3E2 | 1SP3E2 |  |  |  | 1SP3E2 |  |
|  |  |  |  | Evidence |  | 3 | 3 |  |  |  | 3 |  |
|  |  |  |  | Focus |  | 4 | 4 |  |  |  | 4 |  |
|  |  |  |  | Polarity |  | 7 | 7 |  |  |  | 7 |  |
| 355 | 355 | 10618244\_81 | Thermal-cycling conditions were 94 degrees C for 10 min, 1 cycle; 94 degrees C for 30 s, 40 degrees C for 1 min, and 65 degrees C for 4 min, 35 cycles; 65 degrees C for 7 min, 1 cycle; 4 degrees C, hold. |I:\*\*1MP3E0| |L:\*\*1MP3E3| |T:\*\*1MP3E3| |
|  |  |  |  | Annotation |  | 1MP3E0 | 1MP3E3 |  |  |  | 1MP3E3 |  |
|  |  |  |  | Evidence |  | 1 | 4 |  |  |  | 4 |  |
|  |  |  |  | Focus |  | 2 | 2 |  |  |  | 2 |  |
|  |  |  |  | Polarity |  | 7 | 7 |  |  |  | 7 |  |
| 356 | 356 | 12878679\_3 | Two have been identified: an anterior telencephalic source of fibroblast growth factors and the cortical hem, a medial structure expressing winglessint (WNT) and bone morphogenetic proteins. |I:\*\*1SP3E0| |L:\*\*1SP3E0| |T:\*\*1GP3E0| |
|  |  |  |  | Annotation |  | 1SP3E0 | 1SP3E0 |  |  |  | 1GP3E0 |  |
|  |  |  |  | Evidence |  | 1 | 1 |  |  |  | 1 |  |
|  |  |  |  | Focus |  | 4 | 4 |  |  |  | 1 |  |
|  |  |  |  | Polarity |  | 7 | 7 |  |  |  | 7 |  |
| 357 | 357 | 11934051\_4 | All preoperative signs and symptoms of TMJ disorders (4/13 patients in the USSRO+IVRO group and 2/10 patients in the BSSRO group) disappeared after surgery. |I:\*\*1SP3E0| |L:\*\*1SP3E3| |T:\*\*1SP3E1| |
|  |  |  |  | Annotation |  | 1SP3E0 | 1SP3E3 |  |  |  | 1SP3E1 |  |
|  |  |  |  | Evidence |  | 1 | 4 |  |  |  | 2 |  |
|  |  |  |  | Focus |  | 4 | 4 |  |  |  | 4 |  |
|  |  |  |  | Polarity |  | 7 | 7 |  |  |  | 7 |  |
| 358 | 358 | 11713249\_23 | In contrast, our findings demonstrated that sensitive V-H4 cells undergo greater levels of apoptosis |I:\*\*1SP3E3| than resistant parental cells following both equimolar and equitoxic MMC treatment ( 12). |I:\*\*2SP3E2| |L:\*\*1SP3E23| |T:\*\*1SN3E3+| |
|  |  |  |  | Annotation |  | 1SP3E3 2SP3E2 | 1SP3E23 1SP3E23 |  |  |  | 1SN3E3+ 1SN3E3+ |  |
|  |  |  |  | Evidence |  | 4 3 | 3 3 |  |  |  | 4 4 |  |
|  |  |  |  | Focus |  | 4 4 | 4 4 |  |  |  | 4 4 |  |
|  |  |  |  | Polarity |  | 7 7 | 7 7 |  |  |  | 1 1 |  |
| 359 | 359 | 9462510\_126 | The center panels show representative Wright-stained cells after 4 days in culture; the same two clones, LXSN-10 and FLN 2.4, are depicted in each set of panels. |I:\*\*1SGP3E3| |L:\*\*1GP3E3| |T:\*\*1MP3E3| |
|  |  |  |  | Annotation |  | 1SGP3E3 | 1GP3E3 |  |  |  | 1MP3E3 |  |
|  |  |  |  | Evidence |  | 4 | 4 |  |  |  | 4 |  |
|  |  |  |  | Focus |  | 5 | 1 |  |  |  | 2 |  |
|  |  |  |  | Polarity |  | 7 | 7 |  |  |  | 7 |  |
| 360 | 360 | 11463378\_232 | An attractive idea would be that following gene activation the acetylation label is actively removed by a histone deacetylase (HDAC). |I:\*\*1SGP3E3| |L:\*\*1SP0E0| |T:\*\*1SP3E0| |
|  |  |  |  | Annotation |  | 1SGP3E3 | 1SP0E0 |  |  |  | 1SP3E0 |  |
|  |  |  |  | Evidence |  | 4 | 1 |  |  |  | 1 |  |
|  |  |  |  | Focus |  | 5 | 4 |  |  |  | 4 |  |
|  |  |  |  | Polarity |  | 7 | 4 |  |  |  | 7 |  |
| 361 | 361 | 10660608\_272 | Moreover, the spacer arm of the cross-linking agent was only 11.4 Ang long, |I:\*\*1SP3E0| reinforcing the conclusion that sFRP-1 binds directly to Wg |I:\*\*2SP2E0| |T:\*\*1SP3E1| and presumably other Wnt proteins. |I:\*\*3SP1E0| |L:\*\*1SP2E0| |T:\*\*2SP1E0| |
|  |  |  |  | Annotation |  | 1SP3E0 2SP2E0 3SP1E0 | 1SP2E0 1SP2E0 1SP2E0 |  |  |  | 1SP3E1 1SP3E1 2SP1E0 |  |
|  |  |  |  | Evidence |  | 1 1 1 | 1 1 1 |  |  |  | 2 2 1 |  |
|  |  |  |  | Focus |  | 4 4 4 | 4 4 4 |  |  |  | 4 4 4 |  |
|  |  |  |  | Polarity |  | 7 6 5 | 6 6 6 |  |  |  | 7 7 5 |  |
| 362 | 362 | 8674111\_180 | Most of the characteristics intrinsic to the gelsolin family, such as G - actin and F - actin binding activities and phosphoinositide binding, are retained in members of the cofilin family, which are composed of a single domain. |I:\*\*1SP3E0| |L:\*\*1SP3E0| |T:\*\*1SP3E0| |
|  |  |  |  | Annotation |  | 1SP3E0 | 1SP3E0 |  |  |  | 1SP3E0 |  |
|  |  |  |  | Evidence |  | 1 | 1 |  |  |  | 1 |  |
|  |  |  |  | Focus |  | 4 | 4 |  |  |  | 4 |  |
|  |  |  |  | Polarity |  | 7 | 7 |  |  |  | 7 |  |
| 363 | 363 | 10200328\_22 | By applying immunosuppressive drugs as probes |T:\*\*1MP3E0| |I:\*\*1MP3E3| we have shown that a calcineurin-like activity is critical for the modulation of a plasma membrane ion channel activity in guard cells ( 16). |T:\*\*2SP3E2| |I:\*\*2SP3E3| |L:\*\*1SP3E2| |
|  |  |  |  | Annotation |  | 1MP3E3 2SP3E3 | 1SP3E2 1SP3E2 |  |  |  | 1MP3E0 2SP3E2 |  |
|  |  |  |  | Evidence |  | 4 4 | 3 3 |  |  |  | 1 3 |  |
|  |  |  |  | Focus |  | 2 4 | 4 4 |  |  |  | 2 4 |  |
|  |  |  |  | Polarity |  | 7 7 | 7 7 |  |  |  | 7 7 |  |
| 364 | 364 | 10509706\_38 | Whereas most of the previous studies |I:\*\*1SGP3E3| investigating lesion effects on performance in auditory learning tasks |I:\*\*2SGP0E3| had utilized either classical conditioning paradigms or detection tasks often requiring the detection of only a change in stimulus conditions, |I:\*\*3MP3E3| more recent studies suggested different mechanisms of auditory cortex for mediating CR+ and CR , respectively (e.g., Jarrell et al. 1987 ; Teich et al. 1988 ), |I:\*\*4SGP3E2| making separate record of both behaviors desirable. |I:\*\*5SGP3E3| |L:\*\*1GSP3E2| |T:\*\*1MP3E2| |
|  |  |  |  | Annotation |  | 1SGP3E3 2SGP0E3 3MP3E3 4SGP3E2 5SGP3E3 | 1GSP3E2 1GSP3E2 1GSP3E2 1GSP3E2 1GSP3E2 |  |  |  | 1MP3E2 1MP3E2 1MP3E2 1MP3E2 1MP3E2 |  |
|  |  |  |  | Evidence |  | 4 4 4 3 4 | 3 3 3 3 3 |  |  |  | 3 3 3 3 3 |  |
|  |  |  |  | Focus |  | 5 5 2 5 5 | 5 5 5 5 5 |  |  |  | 2 2 2 2 2 |  |
|  |  |  |  | Polarity |  | 7 4 7 7 7 | 7 7 7 7 7 |  |  |  | 7 7 7 7 7 |  |
| 365 | 365 | 11086983\_176 | As an additional strategy to address the influence of chronic exposure to antipsychotic medications ( Nakahara et al. 1998 ; Johnson et al. 1999 ; Selemon et al. 1999 ), |I:\*\*1SGP0E3| we compared gene expression in the frontal cortex of haloperidol-treated and control monkeys |I:\*\*2SGP3E3| by microarray and in situ hybridization analyses. |I:\*\*3MP3E3| |L:\*\*1GSP3E23| |T:\*\*1MP3E3| |
|  |  |  |  | Annotation |  | 1SGP0E3 2SGP3E3 3MP3E3 | 1GSP3E23 1GSP3E23 1GSP3E23 |  |  |  | 1MP3E3 1MP3E3 1MP3E3 |  |
|  |  |  |  | Evidence |  | 4 4 4 | 3 3 3 |  |  |  | 4 4 4 |  |
|  |  |  |  | Focus |  | 5 5 2 | 5 5 5 |  |  |  | 2 2 2 |  |
|  |  |  |  | Polarity |  | 4 7 7 | 7 7 7 |  |  |  | 7 7 7 |  |
| 366 | 366 | 11463849\_206 | Since brainiac acts cell nonautonomously in Drosophila, |I:\*\*1SP3E0| the putative glycosyltransferase activity suggests that Brainiac might regulate signaling events and/or cell adhesion processes through specific glycosylation of cell-surface proteins. |I:\*\*2SP1E0| |L:\*\*1SP1E0| |T:\*\*1SP1E0| |
|  |  |  |  | Annotation |  | 1SP3E0 2SP1E0 | 1SP1E0 1SP1E0 |  |  |  | 1SP1E0 1SP1E0 |  |
|  |  |  |  | Evidence |  | 1 1 | 1 1 |  |  |  | 1 1 |  |
|  |  |  |  | Focus |  | 4 4 | 4 4 |  |  |  | 4 4 |  |
|  |  |  |  | Polarity |  | 7 5 | 5 5 |  |  |  | 5 5 |  |
| 367 | 367 | 9827803\_194 | Transverse sections of E11.5 v-null embryos compared to wild-type littermates showed no gross abnormalities ( Figure 6A, Figure 6B, Figure 6E, and Figure 6F) |I:\*\*1SN3E3| |L:\*\*1SN3E3| except slightly distended vessels in the forebrain, specifically in the subventricular zones of the ganglionic eminence ( Figure 6F) and, less frequently, at E11.5 in the diencephalic subventricular zones of v-null brains. |I:\*\*2SP3E3+| |L:\*\*2SP3E3| |T:\*\*1SP3E3| |
|  |  |  |  | Annotation |  | 1SN3E3 2SP3E3+ | 1SN3E3 2SP3E3 |  |  |  | 1SP3E3 1SP3E3 |  |
|  |  |  |  | Evidence |  | 4 4 | 4 4 |  |  |  | 4 4 |  |
|  |  |  |  | Focus |  | 4 4 | 4 4 |  |  |  | 4 4 |  |
|  |  |  |  | Polarity |  | 1 7 | 1 7 |  |  |  | 7 7 |  |
| 368 | 368 | 11114901\_235 | Strain RF122 is one of a group of clonally related strains, |I:\*\*1SP3E2| as identified by random amplified polymorphic DNA typing, multilocus enzyme electrophoretic typing, and ribotyping ( 11), |I:\*\*2MP3E2| which characteristically harbor SaPIbov and which produce TSST-1 and SEC ( 12). |I:\*\*3SP3E2| |L:\*\*1GSP3E2| |T:\*\*1MP3E2| |
|  |  |  |  | Annotation |  | 1SP3E2 2MP3E2 3SP3E2 | 1GSP3E2 1GSP3E2 1GSP3E2 |  |  |  | 1MP3E2 1MP3E2 1MP3E2 |  |
|  |  |  |  | Evidence |  | 3 3 3 | 3 3 3 |  |  |  | 3 3 3 |  |
|  |  |  |  | Focus |  | 4 2 4 | 5 5 5 |  |  |  | 2 2 2 |  |
|  |  |  |  | Polarity |  | 7 7 7 | 7 7 7 |  |  |  | 7 7 7 |  |
| 369 | 369 | 12022862\_6 | Oligonucleotides that directly paired with the attenuator gave up to 1760-fold activation. |I:\*\*1SP3E0| |L:\*\*1SP3E0+| |T:\*\*1SP3E1| |
|  |  |  |  | Annotation |  | 1SP3E0 | 1SP3E0+ |  |  |  | 1SP3E1 |  |
|  |  |  |  | Evidence |  | 1 | 1 |  |  |  | 2 |  |
|  |  |  |  | Focus |  | 4 | 4 |  |  |  | 4 |  |
|  |  |  |  | Polarity |  | 7 | 7 |  |  |  | 7 |  |
| 370 | 370 | 10704443\_144 | We could also detect this cross-linked product in vesicles derived from emp24- E178A mutant (data not shown), confirming that vesicle budding occurred using these membranes. |I:\*\*1SP3E3| |L:\*\*1SP3E1| |T:\*\*1SP3E1| |
|  |  |  |  | Annotation |  | 1SP3E3 | 1SP3E1 |  |  |  | 1SP3E1 |  |
|  |  |  |  | Evidence |  | 4 | 2 |  |  |  | 2 |  |
|  |  |  |  | Focus |  | 4 | 4 |  |  |  | 4 |  |
|  |  |  |  | Polarity |  | 7 | 7 |  |  |  | 7 |  |
| 371 | 371 | 9843955\_120 | The von Willebrand factor type C module (VWC), also found in certain collagens and mucins, covers the next 10 cysteine residues, |I:\*\*1SP3E2| and is thought to participate in protein complex formation and oligomerization ( 24). |I:\*\*2SP1E2| |L:\*\*1SP3E2| |T:\*\*1SP2E2| |
|  |  |  |  | Annotation |  | 1SP3E2 2SP1E2 | 1SP3E2 1SP3E2 |  |  |  | 1SP2E2 1SP2E2 |  |
|  |  |  |  | Evidence |  | 3 3 | 3 3 |  |  |  | 3 3 |  |
|  |  |  |  | Focus |  | 4 4 | 4 4 |  |  |  | 4 4 |  |
|  |  |  |  | Polarity |  | 7 5 | 7 7 |  |  |  | 6 6 |  |
| 372 | 372 | 12456651\_5 | The cit1 mutant strains exhibit no visible vegetative defects. |I:\*\*1SN3E0| |L:\*\*1SN3E0| |T:\*\*1SN3E0| |
|  |  |  |  | Annotation |  | 1SN3E0 | 1SN3E0 |  |  |  | 1SN3E0 |  |
|  |  |  |  | Evidence |  | 1 | 1 |  |  |  | 1 |  |
|  |  |  |  | Focus |  | 4 | 4 |  |  |  | 4 |  |
|  |  |  |  | Polarity |  | 1 | 1 |  |  |  | 1 |  |
| 373 | 373 | 9463386\_198 | The phosphorylated IRF-3 associates strongly with CBP/p300 |T:\*\*1SP3E1| then its export from the nucleus is prevented by an unknown mechanism |T:\*\*2SN3E1| |I:\*\*1SP3E0| |L:\*\*1SP3E0| |
|  |  |  |  | Annotation |  | 1SP3E0 1SP3E0 | 1SP3E0 1SP3E0 |  |  |  | 1SP3E1 2SN3E1 |  |
|  |  |  |  | Evidence |  | 1 1 | 1 1 |  |  |  | 2 2 |  |
|  |  |  |  | Focus |  | 4 4 | 4 4 |  |  |  | 4 4 |  |
|  |  |  |  | Polarity |  | 7 7 | 7 7 |  |  |  | 7 1 |  |
| 374 | 374 | 10523653\_163 | GST-Mpk1 bound to resin was resuspended in 2x phosphatase buffer and aliquoted into four 20-mul fractions, each containing ~100 ng of GST-Mpk1. |I:\*\*1MP3E0| |L:\*\*1MP3E3| |T:\*\*1MP3E3| |
|  |  |  |  | Annotation |  | 1MP3E0 | 1MP3E3 |  |  |  | 1MP3E3 |  |
|  |  |  |  | Evidence |  | 1 | 4 |  |  |  | 4 |  |
|  |  |  |  | Focus |  | 2 | 2 |  |  |  | 2 |  |
|  |  |  |  | Polarity |  | 7 | 7 |  |  |  | 7 |  |
| 375 | 375 | 12359610\_11 | standard deviation [SD] 15.0), 1.1 ng/ml (range, 0.0-1.6; |I:\*\*ERROR| |L:\*\*1SP3E0| |T:\*\*1GP3E3| |
|  |  |  |  | Annotation |  | ERROR | 1SP3E0 |  |  |  | 1GP3E3 |  |
|  |  |  |  | Evidence |  | -1000 | 1 |  |  |  | 4 |  |
|  |  |  |  | Focus |  | -1000 | 4 |  |  |  | 1 |  |
|  |  |  |  | Polarity |  | -1000 | 7 |  |  |  | 7 |  |
| 376 | 376 | 9671490\_93 | As anticipated from previous experiments ( 68) |T:\*\*1SP2E2| |I:\*\*1SGP2E3| expression of LEF-1 alone did not augment the activity of the reporter gene. |T:\*\*2SP3E1| |I:\*\*2SN3E3| |L:\*\*1SN3E23+| |
|  |  |  |  | Annotation |  | 1SGP2E3 2SN3E3 | 1SN3E23+ 1SN3E23+ |  |  |  | 1SP2E2 2SP3E1 |  |
|  |  |  |  | Evidence |  | 4 4 | 3 3 |  |  |  | 3 2 |  |
|  |  |  |  | Focus |  | 5 4 | 4 4 |  |  |  | 4 4 |  |
|  |  |  |  | Polarity |  | 6 1 | 1 1 |  |  |  | 6 7 |  |
| 377 | 377 | 12702691\_268 | At this approximate steady state, the accumulation of revertants, as given by the first equality in Equation 14, is formally independent of the mutation rate m. |I:\*\*1SP3E0| |L:\*\*1SN3E3| |T:\*\*1SP3E3| |
|  |  |  |  | Annotation |  | 1SP3E0 | 1SN3E3 |  |  |  | 1SP3E3 |  |
|  |  |  |  | Evidence |  | 1 | 4 |  |  |  | 4 |  |
|  |  |  |  | Focus |  | 4 | 4 |  |  |  | 4 |  |
|  |  |  |  | Polarity |  | 7 | 1 |  |  |  | 7 |  |
| 378 | 378 | 11502253\_38 | Consistent with this idea, |I:\*\*1SGP3E3| Ngn2 expression and enhancer activities are disrupted in mice carrying a mutation in Pax6, which present DV patterning defects in the spinal cord (Ericson et al., 1997 ). |I:\*\*2SP3E2| |L:\*\*1SP3E2-| |T:\*\*1SN3E2| |
|  |  |  |  | Annotation |  | 1SGP3E3 2SP3E2 | 1SP3E2- 1SP3E2- |  |  |  | 1SN3E2 1SN3E2 |  |
|  |  |  |  | Evidence |  | 4 3 | 3 3 |  |  |  | 3 3 |  |
|  |  |  |  | Focus |  | 5 4 | 4 4 |  |  |  | 4 4 |  |
|  |  |  |  | Polarity |  | 7 7 | 7 7 |  |  |  | 1 1 |  |
| 379 | 379 | 9596740\_118 | Supernatants were harvested after 72 h of incubation for detection of IFN-gamma by enzyme-linked immunosorbent assay. |I:\*\*1MP3E0| |L:\*\*1MP3E3| |T:\*\*1SP3E3| |
|  |  |  |  | Annotation |  | 1MP3E0 | 1MP3E3 |  |  |  | 1SP3E3 |  |
|  |  |  |  | Evidence |  | 1 | 4 |  |  |  | 4 |  |
|  |  |  |  | Focus |  | 2 | 2 |  |  |  | 4 |  |
|  |  |  |  | Polarity |  | 7 | 7 |  |  |  | 7 |  |
| 380 | 380 | 12408808\_140 | (C) In wild-type muscle-tendon cell junctions, actin filaments terminate at the submembranous density of the muscle hemiadherens junctions (arrowhead), |L:\*\*1SP3E3| while, in the tendon cells, microtubules (arrow) run from tonofibrils embedded in the cuticle (c) to the basal hemiadherens junctions. |L:\*\*2SP3E3| |I:\*\*1SP3E3| |T:\*\*1SP3E3| |
|  |  |  |  | Annotation |  | 1SP3E3 1SP3E3 | 1SP3E3 2SP3E3 |  |  |  | 1SP3E3 1SP3E3 |  |
|  |  |  |  | Evidence |  | 4 4 | 4 4 |  |  |  | 4 4 |  |
|  |  |  |  | Focus |  | 4 4 | 4 4 |  |  |  | 4 4 |  |
|  |  |  |  | Polarity |  | 7 7 | 7 7 |  |  |  | 7 7 |  |
| 381 | 381 | 10454369\_173 | At which of them synaptic plasticity is essential for odor memory to occur, and whether synaptic plasticity at other brain sites is required in addition remains to be determined (see Hammer and Menzel 1998 ). |I:\*\*1SP0E2| |L:\*\*1SP0E2| |T:\*\*1SP3E2| |
|  |  |  |  | Annotation |  | 1SP0E2 | 1SP0E2 |  |  |  | 1SP3E2 |  |
|  |  |  |  | Evidence |  | 3 | 3 |  |  |  | 3 |  |
|  |  |  |  | Focus |  | 4 | 4 |  |  |  | 4 |  |
|  |  |  |  | Polarity |  | 4 | 4 |  |  |  | 7 |  |
| 382 | 382 | 12034774\_35 | In PC12, SKNMC, and Neuro2A cells, however, serum stimulation resulted in robust reporter gene activation; |L:\*\*1SP3E3+| this was dependent on RhoA, as it was abolished by coexpression of C3 transferase, which ADP ribosylates and thereby inactivates RhoA ( Fig. 1 A). |L:\*\*2SP3E3-| |I:\*\*1SP3E3| |T:\*\*1SN3E3| |
|  |  |  |  | Annotation |  | 1SP3E3 1SP3E3 | 1SP3E3+ 2SP3E3- |  |  |  | 1SN3E3 1SN3E3 |  |
|  |  |  |  | Evidence |  | 4 4 | 4 4 |  |  |  | 4 4 |  |
|  |  |  |  | Focus |  | 4 4 | 4 4 |  |  |  | 4 4 |  |
|  |  |  |  | Polarity |  | 7 7 | 7 7 |  |  |  | 1 1 |  |
| 383 | 383 | 10357840\_172 | Simvastatin treatment significantly reduced the secretion of CE labeled with either [14]oleic acid (54% decrease; P < 0.02) or [14] acetic acid (50% decrease; P < 0.01) |T:\*\*1SP3E3-| |I:\*\*1SP3E0| |L:\*\*1SP3E3-| but had no effect on the secretion of 14C-labeled TG or PL. |T:\*\*2SP3E3| |I:\*\*2SN3E0| |L:\*\*2SN3E3| |
|  |  |  |  | Annotation |  | 1SP3E0 2SN3E0 | 1SP3E3- 2SN3E3 |  |  |  | 1SP3E3- 2SP3E3 |  |
|  |  |  |  | Evidence |  | 1 1 | 4 4 |  |  |  | 4 4 |  |
|  |  |  |  | Focus |  | 4 4 | 4 4 |  |  |  | 4 4 |  |
|  |  |  |  | Polarity |  | 7 1 | 7 1 |  |  |  | 7 7 |  |
| 384 | 384 | 11875077\_117 | The basal cytoplasm of the acinar cells was generally more strongly stained than the apical region which also contained scattered punctate HA staining in some cells. |I:\*\*1SP3E0| |L:\*\*1SP3E0| |T:\*\*1SP3E1+| |
|  |  |  |  | Annotation |  | 1SP3E0 | 1SP3E0 |  |  |  | 1SP3E1+ |  |
|  |  |  |  | Evidence |  | 1 | 1 |  |  |  | 2 |  |
|  |  |  |  | Focus |  | 4 | 4 |  |  |  | 4 |  |
|  |  |  |  | Polarity |  | 7 | 7 |  |  |  | 7 |  |
| 385 | 385 | 9443911\_13 | Cell-cycle regulatory pathways in early embryos differ significantly from those in differentiated somatic cells. |I:\*\*1SP3E0| |L:\*\*1SP3E0| |T:\*\*1SP3E0| |
|  |  |  |  | Annotation |  | 1SP3E0 | 1SP3E0 |  |  |  | 1SP3E0 |  |
|  |  |  |  | Evidence |  | 1 | 1 |  |  |  | 1 |  |
|  |  |  |  | Focus |  | 4 | 4 |  |  |  | 4 |  |
|  |  |  |  | Polarity |  | 7 | 7 |  |  |  | 7 |  |
| 386 | 386 | 9889192\_271 | It is quite possible that this gene participates in some other highly conserved functions common to worms and mammals, and that the involvement of DAP3 in mammalian cell apoptosis is an acquired function occurring as a later evolutionary event. |I:\*\*1SP1E3| |L:\*\*1SP2E0| |T:\*\*1SP2E0| |
|  |  |  |  | Annotation |  | 1SP1E3 | 1SP2E0 |  |  |  | 1SP2E0 |  |
|  |  |  |  | Evidence |  | 4 | 1 |  |  |  | 1 |  |
|  |  |  |  | Focus |  | 4 | 4 |  |  |  | 4 |  |
|  |  |  |  | Polarity |  | 5 | 6 |  |  |  | 6 |  |
| 387 | 387 | 9462510\_37 | The sequence of this fragment predicts 96% amino acid identity with the corresponding region of rJagged1 (amino acids [aa] 205-312), 84% with C-Serrate-1 (aa 178-286), and 52% with C-Delta-1 (aa 203-311). |I:\*\*1SP3E0| |L:\*\*1SP3E0| |T:\*\*1SP3E0| |
|  |  |  |  | Annotation |  | 1SP3E0 | 1SP3E0 |  |  |  | 1SP3E0 |  |
|  |  |  |  | Evidence |  | 1 | 1 |  |  |  | 1 |  |
|  |  |  |  | Focus |  | 4 | 4 |  |  |  | 4 |  |
|  |  |  |  | Polarity |  | 7 | 7 |  |  |  | 7 |  |
| 388 | 388 | 11598064\_149 | Transport of CPS to cell surface increases virulence of V. vulnificus in mice. |I:\*\*1SP3E0+| |L:\*\*1SP3E0+| |T:\*\*1SP3E0+| |
|  |  |  |  | Annotation |  | 1SP3E0+ | 1SP3E0+ |  |  |  | 1SP3E0+ |  |
|  |  |  |  | Evidence |  | 1 | 1 |  |  |  | 1 |  |
|  |  |  |  | Focus |  | 4 | 4 |  |  |  | 4 |  |
|  |  |  |  | Polarity |  | 7 | 7 |  |  |  | 7 |  |
| 389 | 389 | 10958668\_265 | This is reminiscent of the activation of SNF1 in yeast following removal of glucose from the medium ( 50, 52). |I:\*\*1SP3E2| |L:\*\*1SP3E2-| |T:\*\*1SP3E2| |
|  |  |  |  | Annotation |  | 1SP3E2 | 1SP3E2- |  |  |  | 1SP3E2 |  |
|  |  |  |  | Evidence |  | 3 | 3 |  |  |  | 3 |  |
|  |  |  |  | Focus |  | 4 | 4 |  |  |  | 4 |  |
|  |  |  |  | Polarity |  | 7 | 7 |  |  |  | 7 |  |
| 390 | 390 | 11163269\_276 | However, among sensory neurons of the L4 DRG, ShcB / mice exhibit a significant reduction in the number of TrkA+ and IB4+ neurons and small-caliber axon fibers. |I:\*\*1SP3E0-| |L:\*\*1SP3E0-| |T:\*\*1SP3E0-| |
|  |  |  |  | Annotation |  | 1SP3E0- | 1SP3E0- |  |  |  | 1SP3E0- |  |
|  |  |  |  | Evidence |  | 1 | 1 |  |  |  | 1 |  |
|  |  |  |  | Focus |  | 4 | 4 |  |  |  | 4 |  |
|  |  |  |  | Polarity |  | 7 | 7 |  |  |  | 7 |  |
| 391 | 391 | 9696764\_154 | Nevertheless, the significantly higher rates of efflux compared to uptake clearly favor exchange as the usual transport mode of CitT. |I:\*\*1SP3E0| |L:\*\*1SP1E0| |T:\*\*1SP3E0+| |
|  |  |  |  | Annotation |  | 1SP3E0 | 1SP1E0 |  |  |  | 1SP3E0+ |  |
|  |  |  |  | Evidence |  | 1 | 1 |  |  |  | 1 |  |
|  |  |  |  | Focus |  | 4 | 4 |  |  |  | 4 |  |
|  |  |  |  | Polarity |  | 7 | 5 |  |  |  | 7 |  |
| 392 | 392 | 12146969\_7 | A weakly associated S-Met and aqua are likely axial ligands. |I:\*\*1SP3E0| |L:\*\*1GP1E0| |T:\*\*1SP1E0| |
|  |  |  |  | Annotation |  | 1SP3E0 | 1GP1E0 |  |  |  | 1SP1E0 |  |
|  |  |  |  | Evidence |  | 1 | 1 |  |  |  | 1 |  |
|  |  |  |  | Focus |  | 4 | 1 |  |  |  | 4 |  |
|  |  |  |  | Polarity |  | 7 | 5 |  |  |  | 5 |  |
| 393 | 393 | 10873834\_184 | There is apparently much more CDK5 than p35/p25, in other words, there is a dominant level of monomeric CDK5 (data not shown). |I:\*\*1SP3E3| |L:\*\*1SP3E1| |T:\*\*1SP3E1| |
|  |  |  |  | Annotation |  | 1SP3E3 | 1SP3E1 |  |  |  | 1SP3E1 |  |
|  |  |  |  | Evidence |  | 4 | 2 |  |  |  | 2 |  |
|  |  |  |  | Focus |  | 4 | 4 |  |  |  | 4 |  |
|  |  |  |  | Polarity |  | 7 | 7 |  |  |  | 7 |  |
| 394 | 394 | 9334318\_316 | We suggest that in dlg mutant egg chambers, molecules bound by PDZ domains are freed prematurely, in the absence of specific signals, resulting in patterns of cell transformation and intercellular interaction resembling BC development. |I:\*\*1SP1E3| |L:\*\*1SP1E0| |T:\*\*1SP1E0| |
|  |  |  |  | Annotation |  | 1SP1E3 | 1SP1E0 |  |  |  | 1SP1E0 |  |
|  |  |  |  | Evidence |  | 4 | 1 |  |  |  | 1 |  |
|  |  |  |  | Focus |  | 4 | 4 |  |  |  | 4 |  |
|  |  |  |  | Polarity |  | 5 | 5 |  |  |  | 5 |  |
| 395 | 395 | 11230147\_14 | Here, pRB is thought to recruit HDAC and SWI-SNF to the promoter, whereupon these enzymes alter the chromatin structure to that of a repressed state. |I:\*\*1SP3E0| |L:\*\*1SP3E0| |T:\*\*1SP1E0| |
|  |  |  |  | Annotation |  | 1SP3E0 | 1SP3E0 |  |  |  | 1SP1E0 |  |
|  |  |  |  | Evidence |  | 1 | 1 |  |  |  | 1 |  |
|  |  |  |  | Focus |  | 4 | 4 |  |  |  | 4 |  |
|  |  |  |  | Polarity |  | 7 | 7 |  |  |  | 5 |  |
| 396 | 396 | 9545242\_234 | Whole cell extracts were made on ice by homogenization with 30 mul/embryo lysis buffer {2 mM CDTA (1,2-cyclohexanediaminetetraacetic acid), 2 mM DTT, 10% glycerol, 1% Triton-X100, 25 mM Tricine [ N-tris(hydroxymethyl)methyl-glycine], pH 7.8}. |I:\*\*1MP3E0| |L:\*\*1MP3E3| |T:\*\*1MP3E3| |
|  |  |  |  | Annotation |  | 1MP3E0 | 1MP3E3 |  |  |  | 1MP3E3 |  |
|  |  |  |  | Evidence |  | 1 | 4 |  |  |  | 4 |  |
|  |  |  |  | Focus |  | 2 | 2 |  |  |  | 2 |  |
|  |  |  |  | Polarity |  | 7 | 7 |  |  |  | 7 |  |
| 397 | 397 | 9861036\_27 | This sequence shows 25% identity with F-spondin and is followed by a series of eight epidermal growth factor-like repeats similar to those of the tenascin C and X and the beta-subunit of integrins ( 24). |I:\*\*1SP3E2| |L:\*\*1SP3E2| |T:\*\*1SP3E2| |
|  |  |  |  | Annotation |  | 1SP3E2 | 1SP3E2 |  |  |  | 1SP3E2 |  |
|  |  |  |  | Evidence |  | 3 | 3 |  |  |  | 3 |  |
|  |  |  |  | Focus |  | 4 | 4 |  |  |  | 4 |  |
|  |  |  |  | Polarity |  | 7 | 7 |  |  |  | 7 |  |
| 398 | 398 | 11953324\_5 | Several acidic and basic residues turned out to be required for nuclease activity |T:\*\*1SP3E1| |I:\*\*1SP3E0| |L:\*\*1SP3E0| but not DNA binding |T:\*\*1SN3E1| |I:\*\*1SN3E0| |L:\*\*1SN3E0| |
|  |  |  |  | Annotation |  | 1SP3E0 1SN3E0 | 1SP3E0 1SN3E0 |  |  |  | 1SP3E1 1SN3E1 |  |
|  |  |  |  | Evidence |  | 1 1 | 1 1 |  |  |  | 2 2 |  |
|  |  |  |  | Focus |  | 4 4 | 4 4 |  |  |  | 4 4 |  |
|  |  |  |  | Polarity |  | 7 1 | 7 1 |  |  |  | 7 1 |  |
| 399 | 399 | 12186850\_163 | Alkaline extraction was controlled by detection of the marker proteins COXIV and mtHsp70. |I:\*\*1MP3E0| |L:\*\*1SP3E0| |T:\*\*1MP3E3| |
|  |  |  |  | Annotation |  | 1MP3E0 | 1SP3E0 |  |  |  | 1MP3E3 |  |
|  |  |  |  | Evidence |  | 1 | 1 |  |  |  | 4 |  |
|  |  |  |  | Focus |  | 2 | 4 |  |  |  | 2 |  |
|  |  |  |  | Polarity |  | 7 | 7 |  |  |  | 7 |  |
| 400 | 400 | 10347042\_62 | Electrophoresis was performed with an 8% polyacrylamide gel containing 0.1% SDS and 0.01% glycol chitin. |I:\*\*1MP3E0| |L:\*\*1MP3E3| |T:\*\*1MP3E3| |
|  |  |  |  | Annotation |  | 1MP3E0 | 1MP3E3 |  |  |  | 1MP3E3 |  |
|  |  |  |  | Evidence |  | 1 | 4 |  |  |  | 4 |  |
|  |  |  |  | Focus |  | 2 | 2 |  |  |  | 2 |  |
|  |  |  |  | Polarity |  | 7 | 7 |  |  |  | 7 |  |
| 401 | 401 | 12068020\_27 | The thrombin-thrombomodulin interaction can therefore be used as an instructive model system for illustrating the role of hot spots in protein-protein interactions and to dissect the structural origin of residue-residue coupling in epitopes involved in protein recognition. |I:\*\*1SP3E3| |L:\*\*1GP1E0| |T:\*\*1GP3E0| |
|  |  |  |  | Annotation |  | 1SP3E3 | 1GP1E0 |  |  |  | 1GP3E0 |  |
|  |  |  |  | Evidence |  | 4 | 1 |  |  |  | 1 |  |
|  |  |  |  | Focus |  | 4 | 1 |  |  |  | 1 |  |
|  |  |  |  | Polarity |  | 7 | 5 |  |  |  | 7 |  |
| 402 | 402 | 10629227\_168 | Although the majority of beta-catenin was also found in the pellet fraction of epithelial and four-day estradiol-treated cells, |I:\*\*1SP3E3| a minute amount of soluble beta-catenin found in polarized cells was strongly increased upon estradiol-treatment of the cells for four days ( Figure 4 A). |I:\*\*2SP3E3+| |L:\*\*1SP3E3+| |T:\*\*1SP3E3+| |
|  |  |  |  | Annotation |  | 1SP3E3 2SP3E3+ | 1SP3E3+ 1SP3E3+ |  |  |  | 1SP3E3+ 1SP3E3+ |  |
|  |  |  |  | Evidence |  | 4 4 | 4 4 |  |  |  | 4 4 |  |
|  |  |  |  | Focus |  | 4 4 | 4 4 |  |  |  | 4 4 |  |
|  |  |  |  | Polarity |  | 7 7 | 7 7 |  |  |  | 7 7 |  |
| 403 | 403 | 9346957\_65 | The 66- and 52-kDa Shc Isoforms Compete for a Limited Pool of Grb2 Molecules |I:\*\*ERROR| |L:\*\*ERROR| |T:\*\*ERROR| Having isolated the cDNA encoding the 66-kDa Shc isoform, |I:\*\*1SGP3E3| we next examined the effect of increased Shc protein expression on Grb2 association (Fig. 5). |I:\*\*2SGP0E3+| |L:\*\*1SP0E3| |T:\*\*1SP3E3+| |
|  |  |  |  | Annotation |  | ERROR 1SGP3E3 2SGP0E3+ | ERROR 1SP0E3 1SP0E3 |  |  |  | ERROR 1SP3E3+ 1SP3E3+ |  |
|  |  |  |  | Evidence |  | -1000 4 4 | -1000 4 4 |  |  |  | -1000 4 4 |  |
|  |  |  |  | Focus |  | -1000 5 5 | -1000 4 4 |  |  |  | -1000 4 4 |  |
|  |  |  |  | Polarity |  | -1000 7 4 | -1000 4 4 |  |  |  | -1000 7 7 |  |
| 404 | 404 | 11713291\_56 | Given the existence of multiple hsp70 family members (including the stress-induced hsp70.1 and hsp70.3) with close sequence homology, |I:\*\*1SP3E0| it has been hitherto impossible to determine the precise functional contribution of each gene in cellular protection from stress in vivo and how the individual hsp70s functionally relate to each other. |I:\*\*2SGN3E0| |L:\*\*1SN3E3| |T:\*\*1GP3E0| |
|  |  |  |  | Annotation |  | 1SP3E0 2SGN3E0 | 1SN3E3 1SN3E3 |  |  |  | 1GP3E0 1GP3E0 |  |
|  |  |  |  | Evidence |  | 1 1 | 4 4 |  |  |  | 1 1 |  |
|  |  |  |  | Focus |  | 4 5 | 4 4 |  |  |  | 1 1 |  |
|  |  |  |  | Polarity |  | 7 1 | 1 1 |  |  |  | 7 7 |  |
| 405 | 405 | 12359197\_3 | Thirty-six patients undergoing mastectomy and/or axillary clearance who formed seroma post operatively, were randomised into two groups either having daily aspiration of seroma or aspiration as required by patient symptoms. |I:\*\*1MP3E0| |L:\*\*1MP3E3| |T:\*\*1MP3E3| RESULTS: |I:\*\*ERROR| |L:\*\*ERROR| |T:\*\*ERROR| |
|  |  |  |  | Annotation |  | 1MP3E0 ERROR | 1MP3E3 ERROR |  |  |  | 1MP3E3 ERROR |  |
|  |  |  |  | Evidence |  | 1 -1000 | 4 -1000 |  |  |  | 4 -1000 |  |
|  |  |  |  | Focus |  | 2 -1000 | 2 -1000 |  |  |  | 2 -1000 |  |
|  |  |  |  | Polarity |  | 7 -1000 | 7 -1000 |  |  |  | 7 -1000 |  |
| 406 | 406 | 9852164\_332 | The LPA-dependent increase observed in FEPE1L8 cells |I:\*\*1SP3E3+| could be reduced by Toxin B treatment (Fig. 7 B). |I:\*\*2SP3E3-| |L:\*\*1SP1E3-| |T:\*\*1SP3E3-| |
|  |  |  |  | Annotation |  | 1SP3E3+ 2SP3E3- | 1SP1E3- 1SP1E3- |  |  |  | 1SP3E3- 1SP3E3- |  |
|  |  |  |  | Evidence |  | 4 4 | 4 4 |  |  |  | 4 4 |  |
|  |  |  |  | Focus |  | 4 4 | 4 4 |  |  |  | 4 4 |  |
|  |  |  |  | Polarity |  | 7 7 | 5 5 |  |  |  | 7 7 |  |
| 407 | 407 | 11825912\_24 | The ligand-free proteins are more open, as a result of largely rigid body rotations of the domains, using their three connections as a hinge. |I:\*\*1SP3E0| |L:\*\*1SP3E0| |T:\*\*1SP3E0| |
|  |  |  |  | Annotation |  | 1SP3E0 | 1SP3E0 |  |  |  | 1SP3E0 |  |
|  |  |  |  | Evidence |  | 1 | 1 |  |  |  | 1 |  |
|  |  |  |  | Focus |  | 4 | 4 |  |  |  | 4 |  |
|  |  |  |  | Polarity |  | 7 | 7 |  |  |  | 7 |  |
| 408 | 408 | 10662692\_79 | As such, chain elongation during stuttering is truly iterative. |I:\*\*1SP3E0| |L:\*\*1GP3E0| |T:\*\*1SP3E0| |
|  |  |  |  | Annotation |  | 1SP3E0 | 1GP3E0 |  |  |  | 1SP3E0 |  |
|  |  |  |  | Evidence |  | 1 | 1 |  |  |  | 1 |  |
|  |  |  |  | Focus |  | 4 | 1 |  |  |  | 4 |  |
|  |  |  |  | Polarity |  | 7 | 7 |  |  |  | 7 |  |
| 409 | 409 | 9971740\_91 | In this study, we used a segment length of 4.1 mum for all analyses. |I:\*\*1MP3E3| |L:\*\*1MP3E3| |T:\*\*1MP3E3| |
|  |  |  |  | Annotation |  | 1MP3E3 | 1MP3E3 |  |  |  | 1MP3E3 |  |
|  |  |  |  | Evidence |  | 4 | 4 |  |  |  | 4 |  |
|  |  |  |  | Focus |  | 2 | 2 |  |  |  | 2 |  |
|  |  |  |  | Polarity |  | 7 | 7 |  |  |  | 7 |  |
| 410 | 410 | 11069916\_13 | Mutant I614K was shown to misincorporate nucleotides 10-fold more efficiently relative to WT enzyme; in addition, kinetic experiments showed that I614K mutant is also efficient at forming transversion errors by misextending pyrimidine-pyrimidine base pairs at higher rates relative to WT. |I:\*\*1SP3E0| |L:\*\*1SP3E3| |T:\*\*1SP3E1| |
|  |  |  |  | Annotation |  | 1SP3E0 | 1SP3E3 |  |  |  | 1SP3E1 |  |
|  |  |  |  | Evidence |  | 1 | 4 |  |  |  | 2 |  |
|  |  |  |  | Focus |  | 4 | 4 |  |  |  | 4 |  |
|  |  |  |  | Polarity |  | 7 | 7 |  |  |  | 7 |  |
| 411 | 411 | 9039265\_29 | Here, we report that the expression of Cchb4 is abnormal in lh/lh mice |L:\*\*1SP3E3| and show that this is due to a mutation within the Cchb4 gene that is consistent with a complete loss of protein function. |L:\*\*2SP3E3-| |I:\*\*1SP3E3| |T:\*\*1SP3E3| |
|  |  |  |  | Annotation |  | 1SP3E3 1SP3E3 | 1SP3E3 2SP3E3- |  |  |  | 1SP3E3 1SP3E3 |  |
|  |  |  |  | Evidence |  | 4 4 | 4 4 |  |  |  | 4 4 |  |
|  |  |  |  | Focus |  | 4 4 | 4 4 |  |  |  | 4 4 |  |
|  |  |  |  | Polarity |  | 7 7 | 7 7 |  |  |  | 7 7 |  |
| 412 | 412 | 8663454\_64 | 269, 22391-22396 [Abstract] Esko, J. D. (1991) Curr. |I:\*\*ERROR| |L:\*\*1GP3E3| |T:\*\*1GP3E3| |
|  |  |  |  | Annotation |  | ERROR | 1GP3E3 |  |  |  | 1GP3E3 |  |
|  |  |  |  | Evidence |  | -1000 | 4 |  |  |  | 4 |  |
|  |  |  |  | Focus |  | -1000 | 1 |  |  |  | 1 |  |
|  |  |  |  | Polarity |  | -1000 | 7 |  |  |  | 7 |  |
| 413 | 413 | 9390688\_2 | A polymorphism was found at a locus within the H2 complex, producing two distinct but overlapping sets of B27-presented HY peptides. |I:\*\*1SP3E0| |L:\*\*1SP3E3| |T:\*\*1SP3E0| |
|  |  |  |  | Annotation |  | 1SP3E0 | 1SP3E3 |  |  |  | 1SP3E0 |  |
|  |  |  |  | Evidence |  | 1 | 4 |  |  |  | 1 |  |
|  |  |  |  | Focus |  | 4 | 4 |  |  |  | 4 |  |
|  |  |  |  | Polarity |  | 7 | 7 |  |  |  | 7 |  |
| 414 | 414 | 12473690\_71 | Insertion corresponds to 5 - 8% of added [35]atToc159 in the presence of GTP. |I:\*\*1MP3E0| |L:\*\*1SP3E0| |T:\*\*1SP3E0| |
|  |  |  |  | Annotation |  | 1MP3E0 | 1SP3E0 |  |  |  | 1SP3E0 |  |
|  |  |  |  | Evidence |  | 1 | 1 |  |  |  | 1 |  |
|  |  |  |  | Focus |  | 2 | 4 |  |  |  | 4 |  |
|  |  |  |  | Polarity |  | 7 | 7 |  |  |  | 7 |  |
| 415 | 415 | 9649566\_79 | Both whole plasma and the d < 1.006 g/ml density fraction of plasma from 2/2 mice show this broad beta-migration pattern (Fig. 1 B) |T:\*\*1SP3E3| ; |I:\*\*1SP3E3| |L:\*\*1SP3E3| in contrast, 3/3 plasma shows virtually no lipid staining at the beta-position. |T:\*\*1SN3E3| |I:\*\*1SN3E3| |L:\*\*1SN3E3| |
|  |  |  |  | Annotation |  | 1SP3E3 1SN3E3 | 1SP3E3 1SN3E3 |  |  |  | 1SP3E3 1SN3E3 |  |
|  |  |  |  | Evidence |  | 4 4 | 4 4 |  |  |  | 4 4 |  |
|  |  |  |  | Focus |  | 4 4 | 4 4 |  |  |  | 4 4 |  |
|  |  |  |  | Polarity |  | 7 1 | 7 1 |  |  |  | 7 1 |  |
| 416 | 416 | 9520490\_101 | The strain RGY250 ( gef1 ccc2) was transformed with plasmids pRG151; GEF1-GFP and pDY219; CCC2-(HA)3. |I:\*\*1MP3E0| |L:\*\*1MP3E3| |T:\*\*1MP3E3| |
|  |  |  |  | Annotation |  | 1MP3E0 | 1MP3E3 |  |  |  | 1MP3E3 |  |
|  |  |  |  | Evidence |  | 1 | 4 |  |  |  | 4 |  |
|  |  |  |  | Focus |  | 2 | 2 |  |  |  | 2 |  |
|  |  |  |  | Polarity |  | 7 | 7 |  |  |  | 7 |  |
| 417 | 417 | 12065290\_7 | CM also reversed cAMP-mediated NTCP dephosphorylation and translocation to 80 and 15% of the basal level, respectively. |I:\*\*1SP3E0| |L:\*\*1SP3E3| |T:\*\*1SP3E1| |
|  |  |  |  | Annotation |  | 1SP3E0 | 1SP3E3 |  |  |  | 1SP3E1 |  |
|  |  |  |  | Evidence |  | 1 | 4 |  |  |  | 2 |  |
|  |  |  |  | Focus |  | 4 | 4 |  |  |  | 4 |  |
|  |  |  |  | Polarity |  | 7 | 7 |  |  |  | 7 |  |
| 418 | 418 | 12354677\_4 | A mouse hypothalamic cell line, GT1-7, was established as a suitable cell model for the study of leptin signaling. |I:\*\*1SP3E0| |L:\*\*1MP3E3| |T:\*\*1MP3E3| |
|  |  |  |  | Annotation |  | 1SP3E0 | 1MP3E3 |  |  |  | 1MP3E3 |  |
|  |  |  |  | Evidence |  | 1 | 4 |  |  |  | 4 |  |
|  |  |  |  | Focus |  | 4 | 2 |  |  |  | 2 |  |
|  |  |  |  | Polarity |  | 7 | 7 |  |  |  | 7 |  |
| 419 | 419 | 9618427\_34 | The purpose of the present study was |I:\*\*1SGP3E3| to determine the effect of therapeutically relevant concentrations of antithyroid drugs on the neutrophil-mediated inactivation of A1PI and on the formation of reactive oxygen species (superoxide, hypochlorous acid and hydroxyl radicals) in rat neutrophils. |I:\*\*2SGP0E3| |L:\*\*1GSP0E3| |T:\*\*1GP3E3| |
|  |  |  |  | Annotation |  | 1SGP3E3 2SGP0E3 | 1GSP0E3 1GSP0E3 |  |  |  | 1GP3E3 1GP3E3 |  |
|  |  |  |  | Evidence |  | 4 4 | 4 4 |  |  |  | 4 4 |  |
|  |  |  |  | Focus |  | 5 5 | 5 5 |  |  |  | 1 1 |  |
|  |  |  |  | Polarity |  | 7 4 | 4 4 |  |  |  | 7 7 |  |
| 420 | 420 | 11560892\_540 | SEVER, S., H. DAMKE, and S. L. SCHMID, 2000 |L:\*\*1GP3E3| Dynamin: GTP controls the formation of constricted coated pits, the rate limiting step in clathrin-mediated endocytosis. |L:\*\*2SP3E3| |I:\*\*1SP3E2| |T:\*\*1SP3E3| |
|  |  |  |  | Annotation |  | 1SP3E2 1SP3E2 | 1GP3E3 2SP3E3 |  |  |  | 1SP3E3 1SP3E3 |  |
|  |  |  |  | Evidence |  | 3 3 | 4 4 |  |  |  | 4 4 |  |
|  |  |  |  | Focus |  | 4 4 | 1 4 |  |  |  | 4 4 |  |
|  |  |  |  | Polarity |  | 7 7 | 7 7 |  |  |  | 7 7 |  |
| 421 | 421 | 11222586\_192 | The present results confirm the latter observation and further show that the ccmKLMNO genes form an operon (Fig. 6). |I:\*\*1SP3E3| |L:\*\*1SP3E3| |T:\*\*1SP3E3| |
|  |  |  |  | Annotation |  | 1SP3E3 | 1SP3E3 |  |  |  | 1SP3E3 |  |
|  |  |  |  | Evidence |  | 4 | 4 |  |  |  | 4 |  |
|  |  |  |  | Focus |  | 4 | 4 |  |  |  | 4 |  |
|  |  |  |  | Polarity |  | 7 | 7 |  |  |  | 7 |  |
| 422 | 422 | 10369681\_295 | We cannot exclude the possibility |I:\*\*1SGN3E3| that phosphorylated Aiolos increases its affinity for other partners |I:\*\*2SP1E3+| that would allow its translocation to the nucleus, |I:\*\*3SP1E3| |L:\*\*1SN0E3| |T:\*\*1SN2E0| and that Ras might also increase its affinity for other effectors. |I:\*\*4SP1E3| |L:\*\*2SP1E3+| |T:\*\*2SP1E0+| |
|  |  |  |  | Annotation |  | 1SGN3E3 2SP1E3+ 3SP1E3 4SP1E3 | 1SN0E3 1SN0E3 1SN0E3 2SP1E3+ |  |  |  | 1SN2E0 1SN2E0 1SN2E0 2SP1E0+ |  |
|  |  |  |  | Evidence |  | 4 4 4 4 | 4 4 4 4 |  |  |  | 1 1 1 1 |  |
|  |  |  |  | Focus |  | 5 4 4 4 | 4 4 4 4 |  |  |  | 4 4 4 4 |  |
|  |  |  |  | Polarity |  | 1 5 5 5 | 4 4 4 5 |  |  |  | 2 2 2 5 |  |
| 423 | 423 | 9726886\_53 | Membranes were hybridized at 65 degrees C and washed at high stringency (68 degrees C) in 0.1x SSC (1x SSC is 0.15 M NaCl plus 0.015 M sodium citrate) according to the manufacturer's recommendations. |I:\*\*1MP3E0| |L:\*\*1MP3E3| |T:\*\*1MP3E3| |
|  |  |  |  | Annotation |  | 1MP3E0 | 1MP3E3 |  |  |  | 1MP3E3 |  |
|  |  |  |  | Evidence |  | 1 | 4 |  |  |  | 4 |  |
|  |  |  |  | Focus |  | 2 | 2 |  |  |  | 2 |  |
|  |  |  |  | Polarity |  | 7 | 7 |  |  |  | 7 |  |
| 424 | 424 | 9642201\_268 | It is interesting that proteins encoded by neutrally evolving genes can diverge with more than 70% amino acid substitutions and still have a conserved function and three-dimensional structure ( 24). |I:\*\*1SP3E2| |L:\*\*1SP3E2| |T:\*\*1SP3E2| |
|  |  |  |  | Annotation |  | 1SP3E2 | 1SP3E2 |  |  |  | 1SP3E2 |  |
|  |  |  |  | Evidence |  | 3 | 3 |  |  |  | 3 |  |
|  |  |  |  | Focus |  | 4 | 4 |  |  |  | 4 |  |
|  |  |  |  | Polarity |  | 7 | 7 |  |  |  | 7 |  |
| 425 | 425 | 11571181\_263 | One study found that G. candidum is rarely found on industrial St. Nectaire |T:\*\*1SP3E1| |L:\*\*1SN2E2| while it was routinely isolated from farmstead St. Nectaire made with raw1 milk throughout all stages of ripening ( 9). |T:\*\*2MP3E2| |I:\*\*1SP3E2| |L:\*\*2SP3E2| |
|  |  |  |  | Annotation |  | 1SP3E2 1SP3E2 | 1SN2E2 2SP3E2 |  |  |  | 1SP3E1 2MP3E2 |  |
|  |  |  |  | Evidence |  | 3 3 | 3 3 |  |  |  | 2 3 |  |
|  |  |  |  | Focus |  | 4 4 | 4 4 |  |  |  | 4 2 |  |
|  |  |  |  | Polarity |  | 7 7 | 2 7 |  |  |  | 7 7 |  |
| 426 | 426 | 9799795\_42 | The corners in the cube represent the +/ levels in the design, the central point is in the middle of the cube, and the alpha/+alpha levels should be at the same distance from the central point. |I:\*\*1MGP3E3| |L:\*\*1SP3E0| |T:\*\*1GP3E3| |
|  |  |  |  | Annotation |  | 1MGP3E3 | 1SP3E0 |  |  |  | 1GP3E3 |  |
|  |  |  |  | Evidence |  | 4 | 1 |  |  |  | 4 |  |
|  |  |  |  | Focus |  | 3 | 4 |  |  |  | 1 |  |
|  |  |  |  | Polarity |  | 7 | 7 |  |  |  | 7 |  |
| 427 | 427 | 11535587\_1 | Cytosolic CaMK-II activity is involved in the differentiation of adipocytes, myocytes, and pre-neuronal cells ( , ). |I:\*\*1SP3E2| |L:\*\*1SP3E2| |T:\*\*1SP3E2| |
|  |  |  |  | Annotation |  | 1SP3E2 | 1SP3E2 |  |  |  | 1SP3E2 |  |
|  |  |  |  | Evidence |  | 3 | 3 |  |  |  | 3 |  |
|  |  |  |  | Focus |  | 4 | 4 |  |  |  | 4 |  |
|  |  |  |  | Polarity |  | 7 | 7 |  |  |  | 7 |  |
| 428 | 428 | 9660977\_116 | Islet cells and HIT-T15 cells were transfected with either the wild-type prIns1GFP or with constructs carrying mutations of one of the following cis elements: A1, E1, CAAT, CRE, E2, or A3/4. |I:\*\*1MP3E0| |L:\*\*1MP3E3| |T:\*\*1MP3E3| |
|  |  |  |  | Annotation |  | 1MP3E0 | 1MP3E3 |  |  |  | 1MP3E3 |  |
|  |  |  |  | Evidence |  | 1 | 4 |  |  |  | 4 |  |
|  |  |  |  | Focus |  | 2 | 2 |  |  |  | 2 |  |
|  |  |  |  | Polarity |  | 7 | 7 |  |  |  | 7 |  |
| 429 | 429 | 11925399\_6 | In successful attempts, LH and GH levels were higher in those follicles from which oocytes giving rise to transferred embryos (i.e. embryos with best morphology and fastest cleavage rate) originated, |I:\*\*1SP3E0+| as compared with other follicles from which a mature oocyte was recovered but was cryopreserved for later use. |I:\*\*2SP3E0| |L:\*\*1SP3E3+| |T:\*\*1MP3E3| |
|  |  |  |  | Annotation |  | 1SP3E0+ 2SP3E0 | 1SP3E3+ 1SP3E3+ |  |  |  | 1MP3E3 1MP3E3 |  |
|  |  |  |  | Evidence |  | 1 1 | 4 4 |  |  |  | 4 4 |  |
|  |  |  |  | Focus |  | 4 4 | 4 4 |  |  |  | 2 2 |  |
|  |  |  |  | Polarity |  | 7 7 | 7 7 |  |  |  | 7 7 |  |
| 430 | 430 | 12095448\_2 | WNV was detected in all three specimen types from 20 dead crows and jays with an average of >10(5) WNV PFU in each. |I:\*\*1SP3E0| |L:\*\*1SP3E3| |T:\*\*1MP3E3| |
|  |  |  |  | Annotation |  | 1SP3E0 | 1SP3E3 |  |  |  | 1MP3E3 |  |
|  |  |  |  | Evidence |  | 1 | 4 |  |  |  | 4 |  |
|  |  |  |  | Focus |  | 4 | 4 |  |  |  | 2 |  |
|  |  |  |  | Polarity |  | 7 | 7 |  |  |  | 7 |  |
| 431 | 431 | 11086989\_238 | Both of these steps are crucial, as evidenced by the fact that rdgC mutants and arr2(S366A) mutants both undergo rapid light-dependent retinal degeneration. |I:\*\*1SP3E0| |L:\*\*1SP3E1| |T:\*\*1SP3E0| |
|  |  |  |  | Annotation |  | 1SP3E0 | 1SP3E1 |  |  |  | 1SP3E0 |  |
|  |  |  |  | Evidence |  | 1 | 2 |  |  |  | 1 |  |
|  |  |  |  | Focus |  | 4 | 4 |  |  |  | 4 |  |
|  |  |  |  | Polarity |  | 7 | 7 |  |  |  | 7 |  |
| 432 | 432 | 9298899\_230 | Bead pellets were washed four times with 1 ml of binding buffer and boiled in reducing SDS - PAGE loading buffer, and the proteins in the supernatant were separated by SDS - PAGE (data not shown). |I:\*\*1MP3E3| |L:\*\*1MP3E1| |T:\*\*1MP3E2| |
|  |  |  |  | Annotation |  | 1MP3E3 | 1MP3E1 |  |  |  | 1MP3E2 |  |
|  |  |  |  | Evidence |  | 4 | 2 |  |  |  | 3 |  |
|  |  |  |  | Focus |  | 2 | 2 |  |  |  | 2 |  |
|  |  |  |  | Polarity |  | 7 | 7 |  |  |  | 7 |  |
| 433 | 433 | 10406800\_171 | Gentamicin survival assays were performed as previously described (Lecuit et al., 1997 ) with L.innocua transformed with pRB474 without insert, and L.innocua transformed with pRB474 harboring the inlA gene. |I:\*\*1MP3E0| |L:\*\*1MP3E23| |T:\*\*1MP3E3| |
|  |  |  |  | Annotation |  | 1MP3E0 | 1MP3E23 |  |  |  | 1MP3E3 |  |
|  |  |  |  | Evidence |  | 1 | 3 |  |  |  | 4 |  |
|  |  |  |  | Focus |  | 2 | 2 |  |  |  | 2 |  |
|  |  |  |  | Polarity |  | 7 | 7 |  |  |  | 7 |  |
| 434 | 434 | 10495268\_168 | If the engrailed-2 gene were a direct target of Wnt signaling, which worked through -catenin binding to LEF/TCF homologs on the promoter, |I:\*\*1SP0E3| then one would predict that induction of the engrailed-2 gene by Wnt and -catenin could be blocked by a dominant negative TCF. |I:\*\*2SP1E3| |L:\*\*1SP0E0-| |T:\*\*1GP3E0| |
|  |  |  |  | Annotation |  | 1SP0E3 2SP1E3 | 1SP0E0- 1SP0E0- |  |  |  | 1GP3E0 1GP3E0 |  |
|  |  |  |  | Evidence |  | 4 4 | 1 1 |  |  |  | 1 1 |  |
|  |  |  |  | Focus |  | 4 4 | 4 4 |  |  |  | 1 1 |  |
|  |  |  |  | Polarity |  | 4 5 | 4 4 |  |  |  | 7 7 |  |
| 435 | 435 | 12086612\_1 | Summary Main Text References |I:\*\*ERROR| |L:\*\*ERROR| |T:\*\*ERROR| In a novel genetic screen, |I:\*\*1MP3E0| the nuclear-cytoplasmic transport system was found to reposition DNA to the nuclear pore and establish a barrier to the spread of heterochromatin. |I:\*\*2SP3E0| |L:\*\*1SP3E3| |T:\*\*1SP3E1| |
|  |  |  |  | Annotation |  | ERROR 1MP3E0 2SP3E0 | ERROR 1SP3E3 1SP3E3 |  |  |  | ERROR 1SP3E1 1SP3E1 |  |
|  |  |  |  | Evidence |  | -1000 1 1 | -1000 4 4 |  |  |  | -1000 2 2 |  |
|  |  |  |  | Focus |  | -1000 2 4 | -1000 4 4 |  |  |  | -1000 4 4 |  |
|  |  |  |  | Polarity |  | -1000 7 7 | -1000 7 7 |  |  |  | -1000 7 7 |  |
| 436 | 436 | 11136978\_98 | This is consistent with our observation that the K255A mutant is activated by H-Ras G12V to half of the extent of the wild-type p110 in COS-7 cells (data not shown) despite having an affinity for soluble N-Ras. |I:\*\*1SP3E3| |L:\*\*1SP3E1+| |T:\*\*1SP1E1| |
|  |  |  |  | Annotation |  | 1SP3E3 | 1SP3E1+ |  |  |  | 1SP1E1 |  |
|  |  |  |  | Evidence |  | 4 | 2 |  |  |  | 2 |  |
|  |  |  |  | Focus |  | 4 | 4 |  |  |  | 4 |  |
|  |  |  |  | Polarity |  | 7 | 7 |  |  |  | 5 |  |
| 437 | 437 | 11687464\_101 | Appropriate isotype control antibodies were used for both the nonstimulated and PPD-stimulated wells as a control for nonspecific binding of lymphocyte subset antibodies to activated cells. |I:\*\*1MP3E0| |L:\*\*1MP3E3| |T:\*\*1MP3E3| |
|  |  |  |  | Annotation |  | 1MP3E0 | 1MP3E3 |  |  |  | 1MP3E3 |  |
|  |  |  |  | Evidence |  | 1 | 4 |  |  |  | 4 |  |
|  |  |  |  | Focus |  | 2 | 2 |  |  |  | 2 |  |
|  |  |  |  | Polarity |  | 7 | 7 |  |  |  | 7 |  |
| 438 | 438 | 10496894\_15 | In the present study, we set out to clarify the mechanisms underlying the rapid accumulation of platelets and the ensuing anaphylactoid shock that are both induced by LPS while paying particular attention to the possible role of complement. |I:\*\*1SGP0E3| |L:\*\*1GSP0E3| |T:\*\*1GP3E3| |
|  |  |  |  | Annotation |  | 1SGP0E3 | 1GSP0E3 |  |  |  | 1GP3E3 |  |
|  |  |  |  | Evidence |  | 4 | 4 |  |  |  | 4 |  |
|  |  |  |  | Focus |  | 5 | 5 |  |  |  | 1 |  |
|  |  |  |  | Polarity |  | 4 | 4 |  |  |  | 7 |  |
| 439 | 439 | 10948161\_227 | Third, short-term exposure of AGS gastric epithelial cells to H. pylori also results in rapid downregulation of p27kip1 ( 39). |I:\*\*1SP3E2-| |L:\*\*1SP3E2-| |T:\*\*1SP3E2-| |
|  |  |  |  | Annotation |  | 1SP3E2- | 1SP3E2- |  |  |  | 1SP3E2- |  |
|  |  |  |  | Evidence |  | 3 | 3 |  |  |  | 3 |  |
|  |  |  |  | Focus |  | 4 | 4 |  |  |  | 4 |  |
|  |  |  |  | Polarity |  | 7 | 7 |  |  |  | 7 |  |
| 440 | 440 | 10753894\_353 | Furthermore, E-cadherin complexes with beta-catenin, a protein involved in transduction of wnt signaling to the nuclear TCF/LEF transcription factors ( 76). |I:\*\*1SP3E2| |L:\*\*1SP3E2| |T:\*\*1SP3E2| |
|  |  |  |  | Annotation |  | 1SP3E2 | 1SP3E2 |  |  |  | 1SP3E2 |  |
|  |  |  |  | Evidence |  | 3 | 3 |  |  |  | 3 |  |
|  |  |  |  | Focus |  | 4 | 4 |  |  |  | 4 |  |
|  |  |  |  | Polarity |  | 7 | 7 |  |  |  | 7 |  |
| 441 | 441 | 9790533\_144 | Unlike the PH domains contained in other proteins whose location is highly variable, |I:\*\*1SN3E0| the PH domain is always located C-terminal to the DH domain in DH-containing proteins. |I:\*\*2SP3E0| |L:\*\*1SP3E0| |T:\*\*1SP3E0| |
|  |  |  |  | Annotation |  | 1SN3E0 2SP3E0 | 1SP3E0 1SP3E0 |  |  |  | 1SP3E0 1SP3E0 |  |
|  |  |  |  | Evidence |  | 1 1 | 1 1 |  |  |  | 1 1 |  |
|  |  |  |  | Focus |  | 4 4 | 4 4 |  |  |  | 4 4 |  |
|  |  |  |  | Polarity |  | 1 7 | 7 7 |  |  |  | 7 7 |  |
| 442 | 442 | 10197540\_21 | During this scan, the patients were again asked to indicate periods of hallucinations. |I:\*\*1MP3E0| |L:\*\*1MP3E3| |T:\*\*1MP3E3| |
|  |  |  |  | Annotation |  | 1MP3E0 | 1MP3E3 |  |  |  | 1MP3E3 |  |
|  |  |  |  | Evidence |  | 1 | 4 |  |  |  | 4 |  |
|  |  |  |  | Focus |  | 2 | 2 |  |  |  | 2 |  |
|  |  |  |  | Polarity |  | 7 | 7 |  |  |  | 7 |  |
| 443 | 443 | 12547703\_2 | However, the specific complement pathways involved in I/R injury are unknown. |I:\*\*1SN3E0| |L:\*\*1SP0E0| |T:\*\*1GP3E0| |
|  |  |  |  | Annotation |  | 1SN3E0 | 1SP0E0 |  |  |  | 1GP3E0 |  |
|  |  |  |  | Evidence |  | 1 | 1 |  |  |  | 1 |  |
|  |  |  |  | Focus |  | 4 | 4 |  |  |  | 1 |  |
|  |  |  |  | Polarity |  | 1 | 4 |  |  |  | 7 |  |
| 444 | 444 | 7673207\_32 | Testing these structural hypotheses |I:\*\*1SGP0E3| will require further dissection of cytoplasmic domain topology |I:\*\*2SGP3E3| using competition studies with antibody Fab fragments, physical probes such as fluorescence energy transfer, and direct imaging techniques such as electron diffraction. |I:\*\*3MP3E3| |L:\*\*1GSP0E0| |T:\*\*1GP3E0| |
|  |  |  |  | Annotation |  | 1SGP0E3 2SGP3E3 3MP3E3 | 1GSP0E0 1GSP0E0 1GSP0E0 |  |  |  | 1GP3E0 1GP3E0 1GP3E0 |  |
|  |  |  |  | Evidence |  | 4 4 4 | 1 1 1 |  |  |  | 1 1 1 |  |
|  |  |  |  | Focus |  | 5 5 2 | 5 5 5 |  |  |  | 1 1 1 |  |
|  |  |  |  | Polarity |  | 4 7 7 | 4 4 4 |  |  |  | 7 7 7 |  |
| 445 | 445 | 11751886\_205 | Because beta-catenin bears homology to p120 ctn (both contain central Armadillo repeats) and beta-catenin is capable of relieving LEF/TCF-mediated repression of Wnt pathway gene targets, |I:\*\*1SP3E0| |L:\*\*1SP3E0| we were curious to test if Xp120 ctn might analogously relieve XKaiso mediated repression. |I:\*\*2SGP0E3| |L:\*\*2SP0E3| |T:\*\*1GP3E0| |
|  |  |  |  | Annotation |  | 1SP3E0 2SGP0E3 | 1SP3E0 2SP0E3 |  |  |  | 1GP3E0 1GP3E0 |  |
|  |  |  |  | Evidence |  | 1 4 | 1 4 |  |  |  | 1 1 |  |
|  |  |  |  | Focus |  | 4 5 | 4 4 |  |  |  | 1 1 |  |
|  |  |  |  | Polarity |  | 7 4 | 7 4 |  |  |  | 7 7 |  |
| 446 | 446 | 12042012\_7 | Syncope recurred in three patients |T:\*\*1SP3E3| |I:\*\*1SP3E0| |L:\*\*1SP3E3| but in none of them was it caused by an arrhythmic event |T:\*\*2SN3E3| |I:\*\*2SN3E0| |L:\*\*2SN3E3| |
|  |  |  |  | Annotation |  | 1SP3E0 2SN3E0 | 1SP3E3 2SN3E3 |  |  |  | 1SP3E3 2SN3E3 |  |
|  |  |  |  | Evidence |  | 1 1 | 4 4 |  |  |  | 4 4 |  |
|  |  |  |  | Focus |  | 4 4 | 4 4 |  |  |  | 4 4 |  |
|  |  |  |  | Polarity |  | 7 1 | 7 1 |  |  |  | 7 1 |  |
| 447 | 447 | 12172289\_3 | The tissue was obtained from standard temporal lobe specimens removed because of epilepsy. |I:\*\*1MP3E0| |L:\*\*1MP3E3| |T:\*\*1MP3E3| |
|  |  |  |  | Annotation |  | 1MP3E0 | 1MP3E3 |  |  |  | 1MP3E3 |  |
|  |  |  |  | Evidence |  | 1 | 4 |  |  |  | 4 |  |
|  |  |  |  | Focus |  | 2 | 2 |  |  |  | 2 |  |
|  |  |  |  | Polarity |  | 7 | 7 |  |  |  | 7 |  |
| 448 | 448 | 11457839\_143 | Lanes 1, 4, and 7, GST alone; |I:\*\*1GP3E3| |L:\*\*1GP3E3| |T:\*\*1SP3E3| GST-p46 formed a DNA-protein complex with the G1-51 probe ( lane 3), |I:\*\*2SP3E3| |L:\*\*2GSP3E3| |T:\*\*2SN3E3| no complex with the mutated G1-51M10 |L:\*\*3GSN3E3| ( lane 5) |I:\*\*3SN3E3| |T:\*\*3SP3E3+| but efficiently recognized the MafA mutated probe G1-51M3 ( lane 8). |I:\*\*4SP3E3| |L:\*\*4GSP3E3| |T:\*\*4SP3E3| GST-MafA(1-286) increased formation of the p46-DNA complex on G1-51 ( lane 3), |I:\*\*1SP3E3+| |T:\*\*1SP3E3| induced a faint p46-DNA complex on G1-51M10 ( lane 6), |I:\*\*2SP3E3| |T:\*\*2SP3E3| and increased formation of the p46-DNA complex on G1-51M3 ( lane 9). |I:\*\*3SP3E3+| |L:\*\*1GSP3E3+| |T:\*\*3SP3E3| |
|  |  |  |  | Annotation |  | 1GP3E3 2SP3E3 3SN3E3 3SN3E3 4SP3E3 1SP3E3+ 2SP3E3 3SP3E3+ | 1GP3E3 2GSP3E3 3GSN3E3 4GSP3E3 4GSP3E3 1GSP3E3+ 1GSP3E3+ 1GSP3E3+ |  |  |  | 1SP3E3 2SN3E3 3SP3E3+ 3SP3E3+ 4SP3E3 1SP3E3 2SP3E3 3SP3E3 |  |
|  |  |  |  | Evidence |  | 4 4 4 4 4 4 4 4 | 4 4 4 4 4 4 4 4 |  |  |  | 4 4 4 4 4 4 4 4 |  |
|  |  |  |  | Focus |  | 1 4 4 4 4 4 4 4 | 1 5 5 5 5 5 5 5 |  |  |  | 4 4 4 4 4 4 4 4 |  |
|  |  |  |  | Polarity |  | 7 7 1 1 7 7 7 7 | 7 7 1 7 7 7 7 7 |  |  |  | 7 1 7 7 7 7 7 7 |  |
| 449 | 449 | 11481319\_15 | The crystal structure of P-450cam with d-camphor reveals that the substrate is tightly bound in the hydrophobic heme pocket through hydrogen bonding interaction with the hydroxyl group of Tyr-96 and the carbonyl oxygen of d-camphor (Fig. 1 A) ( 9). |I:\*\*1SP3E2| |L:\*\*1SP3E23| |T:\*\*1SP3E3| |
|  |  |  |  | Annotation |  | 1SP3E2 | 1SP3E23 |  |  |  | 1SP3E3 |  |
|  |  |  |  | Evidence |  | 3 | 3 |  |  |  | 4 |  |
|  |  |  |  | Focus |  | 4 | 4 |  |  |  | 4 |  |
|  |  |  |  | Polarity |  | 7 | 7 |  |  |  | 7 |  |
| 450 | 450 | 10877795\_37 | Malic enzyme catalyzes the reversible carboxylation of pyruvate to malate coupled with NADPH oxidation. |I:\*\*1SP3E0| |L:\*\*1SP3E0| |T:\*\*1SP3E0| |
|  |  |  |  | Annotation |  | 1SP3E0 | 1SP3E0 |  |  |  | 1SP3E0 |  |
|  |  |  |  | Evidence |  | 1 | 1 |  |  |  | 1 |  |
|  |  |  |  | Focus |  | 4 | 4 |  |  |  | 4 |  |
|  |  |  |  | Polarity |  | 7 | 7 |  |  |  | 7 |  |
| 451 | 451 | 11313474\_184 | An hnRNPA1 RNA binding domain is required for interaction with IkappaBalpha. |I:\*\*1SP3E0| |L:\*\*1SP3E0| |T:\*\*1SP3E0| |
|  |  |  |  | Annotation |  | 1SP3E0 | 1SP3E0 |  |  |  | 1SP3E0 |  |
|  |  |  |  | Evidence |  | 1 | 1 |  |  |  | 1 |  |
|  |  |  |  | Focus |  | 4 | 4 |  |  |  | 4 |  |
|  |  |  |  | Polarity |  | 7 | 7 |  |  |  | 7 |  |
| 452 | 452 | 9501077\_32 | Significant local structural changes may occur when Gag is cleaved, particularly given the dramatic global changes that accompany viral maturation. |I:\*\*1SP3E0| |L:\*\*1SP1E0| |T:\*\*1SP1E0| |
|  |  |  |  | Annotation |  | 1SP3E0 | 1SP1E0 |  |  |  | 1SP1E0 |  |
|  |  |  |  | Evidence |  | 1 | 1 |  |  |  | 1 |  |
|  |  |  |  | Focus |  | 4 | 4 |  |  |  | 4 |  |
|  |  |  |  | Polarity |  | 7 | 5 |  |  |  | 5 |  |
| 453 | 453 | 10958697\_328 | At 24 h after transfection, the confluent cultures were either incubated in GM for 1 further day (GM) or shifted to DM for 1 day (DM1) or 2 days (DM2). |I:\*\*1MP3E0| |L:\*\*1MP3E3| |T:\*\*1MP3E3| |
|  |  |  |  | Annotation |  | 1MP3E0 | 1MP3E3 |  |  |  | 1MP3E3 |  |
|  |  |  |  | Evidence |  | 1 | 4 |  |  |  | 4 |  |
|  |  |  |  | Focus |  | 2 | 2 |  |  |  | 2 |  |
|  |  |  |  | Polarity |  | 7 | 7 |  |  |  | 7 |  |
| 454 | 454 | 10583997\_82 | The hybridization signals were quantified by phosphorimaging (for details, see below). |I:\*\*1MP3E3| |L:\*\*1MP3E3| |T:\*\*1MP3E3| |
|  |  |  |  | Annotation |  | 1MP3E3 | 1MP3E3 |  |  |  | 1MP3E3 |  |
|  |  |  |  | Evidence |  | 4 | 4 |  |  |  | 4 |  |
|  |  |  |  | Focus |  | 2 | 2 |  |  |  | 2 |  |
|  |  |  |  | Polarity |  | 7 | 7 |  |  |  | 7 |  |
| 455 | 455 | 11254631\_197 | Apoptosis was measured with an ELISA method as described above. |I:\*\*1MP3E3| |L:\*\*1MP3E3| |T:\*\*1MP3E3| |
|  |  |  |  | Annotation |  | 1MP3E3 | 1MP3E3 |  |  |  | 1MP3E3 |  |
|  |  |  |  | Evidence |  | 4 | 4 |  |  |  | 4 |  |
|  |  |  |  | Focus |  | 2 | 2 |  |  |  | 2 |  |
|  |  |  |  | Polarity |  | 7 | 7 |  |  |  | 7 |  |
| 456 | 456 | 11498057\_233 | Finally, a recent study by Mu ller (2000 ) has demonstrated that multiple olfactory conditioning trials produce a prolonged activation of PKA in the antennal lobes of the honeybee, and prolonging the normally transient activation of PKA in this region |I:\*\*1SP3E2+| after a single conditioning trial enhances the induction of LTM. |I:\*\*2SP3E2| |L:\*\*1SP3E2+| |T:\*\*1SP3E2+| |
|  |  |  |  | Annotation |  | 1SP3E2+ 2SP3E2 | 1SP3E2+ 1SP3E2+ |  |  |  | 1SP3E2+ 1SP3E2+ |  |
|  |  |  |  | Evidence |  | 3 3 | 3 3 |  |  |  | 3 3 |  |
|  |  |  |  | Focus |  | 4 4 | 4 4 |  |  |  | 4 4 |  |
|  |  |  |  | Polarity |  | 7 7 | 7 7 |  |  |  | 7 7 |  |
| 457 | 457 | 9187275\_48 | At a holding potential of 100 mV, 10 muM CBZ has negligible effect on the Na+ currents, and even 100 muM CBZ produces no more than ~10% inhibition of the Na+ currents. |I:\*\*1SP3E0| |L:\*\*1SN3E3| |T:\*\*1SP3E1| |
|  |  |  |  | Annotation |  | 1SP3E0 | 1SN3E3 |  |  |  | 1SP3E1 |  |
|  |  |  |  | Evidence |  | 1 | 4 |  |  |  | 2 |  |
|  |  |  |  | Focus |  | 4 | 4 |  |  |  | 4 |  |
|  |  |  |  | Polarity |  | 7 | 1 |  |  |  | 7 |  |
| 458 | 458 | 10511568\_168 | The tapetal layer resembles a normal tapetum |L:\*\*1SP3E0| but |I:\*\*1SP3E0| lacks its typical density. |I:\*\*2SN3E0| |L:\*\*2SN3E0| |T:\*\*1GP3E0| |
|  |  |  |  | Annotation |  | 1SP3E0 1SP3E0 2SN3E0 | 1SP3E0 2SN3E0 2SN3E0 |  |  |  | 1GP3E0 1GP3E0 1GP3E0 |  |
|  |  |  |  | Evidence |  | 1 1 1 | 1 1 1 |  |  |  | 1 1 1 |  |
|  |  |  |  | Focus |  | 4 4 4 | 4 4 4 |  |  |  | 1 1 1 |  |
|  |  |  |  | Polarity |  | 7 7 1 | 7 1 1 |  |  |  | 7 7 7 |  |
| 459 | 459 | 9736749\_13 | Inconsistencies in the in vivo neuroimaging literature may exist |I:\*\*1SGP3E3| because hippocampal volume decreases in schizophrenia are small relative to the normal variability of hippocampal volumes and the error associated with manual techniques for outlining small neuroanatomical structures ( 16-18). |I:\*\*2SP3E2-| |L:\*\*1GSP1E2| |T:\*\*1SP1E2-| |
|  |  |  |  | Annotation |  | 1SGP3E3 2SP3E2- | 1GSP1E2 1GSP1E2 |  |  |  | 1SP1E2- 1SP1E2- |  |
|  |  |  |  | Evidence |  | 4 3 | 3 3 |  |  |  | 3 3 |  |
|  |  |  |  | Focus |  | 5 4 | 5 5 |  |  |  | 4 4 |  |
|  |  |  |  | Polarity |  | 7 7 | 5 5 |  |  |  | 5 5 |  |
| 460 | 460 | 11389849\_113 | [In new window] To measure infectivity in a more quantitative fashion, |I:\*\*1SGP0E3| we then turned to single-cycle challenges of reporter cell lines (Table 1). |I:\*\*2MP3E3| |L:\*\*1GMP3E3| |T:\*\*1MP3E3| |
|  |  |  |  | Annotation |  | 1SGP0E3 2MP3E3 | 1GMP3E3 1GMP3E3 |  |  |  | 1MP3E3 1MP3E3 |  |
|  |  |  |  | Evidence |  | 4 4 | 4 4 |  |  |  | 4 4 |  |
|  |  |  |  | Focus |  | 5 2 | 3 3 |  |  |  | 2 2 |  |
|  |  |  |  | Polarity |  | 4 7 | 7 7 |  |  |  | 7 7 |  |
| 461 | 461 | 10405392\_135 | The gC DNA sequences of two different BHV-5 strains, strains N569 (accession no. Z49224) and TX-89 (accession no. U35883), displayed homologies with strain LA (pMUH-2) of 90.1 and 90.3%, respectively. |I:\*\*1SP3E0| |L:\*\*1SP3E0| |T:\*\*1SP3E1| |
|  |  |  |  | Annotation |  | 1SP3E0 | 1SP3E0 |  |  |  | 1SP3E1 |  |
|  |  |  |  | Evidence |  | 1 | 1 |  |  |  | 2 |  |
|  |  |  |  | Focus |  | 4 | 4 |  |  |  | 4 |  |
|  |  |  |  | Polarity |  | 7 | 7 |  |  |  | 7 |  |
| 462 | 462 | 12012156\_4 | In two experiments either valid or invalid advance information about direction (experiment 1) and about direction and response hand (experiment 2) was provided. |I:\*\*1MGP3E0| |L:\*\*1GP3E3| |T:\*\*1MP3E3| |
|  |  |  |  | Annotation |  | 1MGP3E0 | 1GP3E3 |  |  |  | 1MP3E3 |  |
|  |  |  |  | Evidence |  | 1 | 4 |  |  |  | 4 |  |
|  |  |  |  | Focus |  | 3 | 1 |  |  |  | 2 |  |
|  |  |  |  | Polarity |  | 7 | 7 |  |  |  | 7 |  |
| 463 | 463 | 10197532\_65 | At this stage in development, when axons are contacting most of the dendrite surface, |I:\*\*1SP3E3| we found that the dendrite marker MAP2 was most useful for identifying axonal versus dendritic domains; |I:\*\*2MSP3E3| |L:\*\*1SP3E3| |T:\*\*1MP3E3| labeled axons were identified as myc-positive, MAP2-negative, long, fine caliber processes that typically extended far beyond the somatodendritic domain. |I:\*\*3SP3E3| |L:\*\*2SP3E3| |T:\*\*2SP3E3| |
|  |  |  |  | Annotation |  | 1SP3E3 2MSP3E3 3SP3E3 | 1SP3E3 1SP3E3 2SP3E3 |  |  |  | 1MP3E3 1MP3E3 2SP3E3 |  |
|  |  |  |  | Evidence |  | 4 4 4 | 4 4 4 |  |  |  | 4 4 4 |  |
|  |  |  |  | Focus |  | 4 6 4 | 4 4 4 |  |  |  | 2 2 4 |  |
|  |  |  |  | Polarity |  | 7 7 7 | 7 7 7 |  |  |  | 7 7 7 |  |
| 464 | 464 | 11728332\_105 | As time progressed, the phosphorylation levels of moesin continued to rise |I:\*\*1SP3E0+| and included the bulk of the molecules outside the synapse. |I:\*\*2SP3E0| |L:\*\*1SP3E3+| |T:\*\*1SP3E1+| |
|  |  |  |  | Annotation |  | 1SP3E0+ 2SP3E0 | 1SP3E3+ 1SP3E3+ |  |  |  | 1SP3E1+ 1SP3E1+ |  |
|  |  |  |  | Evidence |  | 1 1 | 4 4 |  |  |  | 2 2 |  |
|  |  |  |  | Focus |  | 4 4 | 4 4 |  |  |  | 4 4 |  |
|  |  |  |  | Polarity |  | 7 7 | 7 7 |  |  |  | 7 7 |  |
| 465 | 465 | 9655854\_185 | This observation strengthens the notion |I:\*\*1SGP3E3| that selective peripherally acting mu opioid agonists can be developed by targeting compounds to act at peripheral sites, |L:\*\*1SP1E0| |T:\*\*1SP3E3| while avoiding centrally mediated undesirable effects. |I:\*\*1SP3E0| |L:\*\*1SN1E0| |T:\*\*1SN3E0| |
|  |  |  |  | Annotation |  | 1SGP3E3 1SP3E0 1SP3E0 | 1SP1E0 1SP1E0 1SN1E0 |  |  |  | 1SP3E3 1SP3E3 1SN3E0 |  |
|  |  |  |  | Evidence |  | 4 1 1 | 1 1 1 |  |  |  | 4 4 1 |  |
|  |  |  |  | Focus |  | 5 4 4 | 4 4 4 |  |  |  | 4 4 4 |  |
|  |  |  |  | Polarity |  | 7 7 7 | 5 5 3 |  |  |  | 7 7 1 |  |
| 466 | 466 | 10393190\_30 | Male fertility can be restored by the introduction in a cross of nuclear genes, usually termed Rf genes for restorer of fertility (Schnable and Wise, 1998 ). |I:\*\*1SP3E2| |L:\*\*1SP3E2| |T:\*\*1SP3E2| |
|  |  |  |  | Annotation |  | 1SP3E2 | 1SP3E2 |  |  |  | 1SP3E2 |  |
|  |  |  |  | Evidence |  | 3 | 3 |  |  |  | 3 |  |
|  |  |  |  | Focus |  | 4 | 4 |  |  |  | 4 |  |
|  |  |  |  | Polarity |  | 7 | 7 |  |  |  | 7 |  |
| 467 | 467 | 8755480\_263 | In newborn mice, axotomized motoneurons die by an apoptotic process within 5 days, with a peak that is observed very rapidly, 28 h after the lesion ( De Bilbao and Dubois-Dauphin 1996 ). |I:\*\*1SP3E2| |L:\*\*1SP3E2| |T:\*\*1SP3E2| |
|  |  |  |  | Annotation |  | 1SP3E2 | 1SP3E2 |  |  |  | 1SP3E2 |  |
|  |  |  |  | Evidence |  | 3 | 3 |  |  |  | 3 |  |
|  |  |  |  | Focus |  | 4 | 4 |  |  |  | 4 |  |
|  |  |  |  | Polarity |  | 7 | 7 |  |  |  | 7 |  |
| 468 | 468 | 12077113\_36 | Specifically, we tested the effects of stable overexpression of Wnt-3A, wild-type GSK-3beta, or kinase-dead GSK-3beta on the level of chondrogenesis, N-cadherin expression, and the subcellular distribution of beta-catenin and LEF-1. |I:\*\*1SGP0E3| |L:\*\*1GSP3E3+| |T:\*\*1MP3E3| |
|  |  |  |  | Annotation |  | 1SGP0E3 | 1GSP3E3+ |  |  |  | 1MP3E3 |  |
|  |  |  |  | Evidence |  | 4 | 4 |  |  |  | 4 |  |
|  |  |  |  | Focus |  | 5 | 5 |  |  |  | 2 |  |
|  |  |  |  | Polarity |  | 4 | 7 |  |  |  | 7 |  |
| 469 | 469 | 12187298\_6 | METHODS: Forty-nine stool samples for steatocrit processing were obtained from 72-hour fecal fat collections performed on twenty-seven persons with cystic fibrosis. |I:\*\*1MP3E3| |L:\*\*1MP3E3| |T:\*\*1MP3E3| |
|  |  |  |  | Annotation |  | 1MP3E3 | 1MP3E3 |  |  |  | 1MP3E3 |  |
|  |  |  |  | Evidence |  | 4 | 4 |  |  |  | 4 |  |
|  |  |  |  | Focus |  | 2 | 2 |  |  |  | 2 |  |
|  |  |  |  | Polarity |  | 7 | 7 |  |  |  | 7 |  |
| 470 | 470 | 9199932\_251 | M13 templates were prepared by the triton method (Mardis 1994 ) and sequenced using Thermo Sequenase (Amersham). |I:\*\*1MP3E0| |L:\*\*1MP3E23| |T:\*\*1MP3E3| |
|  |  |  |  | Annotation |  | 1MP3E0 | 1MP3E23 |  |  |  | 1MP3E3 |  |
|  |  |  |  | Evidence |  | 1 | 3 |  |  |  | 4 |  |
|  |  |  |  | Focus |  | 2 | 2 |  |  |  | 2 |  |
|  |  |  |  | Polarity |  | 7 | 7 |  |  |  | 7 |  |
| 471 | 471 | 9548720\_11 | These proteins consist of several domains, the "`head"` or motor domain (containing the microtubule and ATP binding sites), a "`stalk,"` which is largely alpha-helical and causes two kinesin chains to dimerize via a coiled-coil interaction, and a "`tail"` connecting the stalk to the cargo (for reviews see Goldstein, 1993 ; Brady, 1995 ; Cole and Scholey, 1995 ). |I:\*\*1SP3E2| |L:\*\*1SP3E2| |T:\*\*1GP3E2| |
|  |  |  |  | Annotation |  | 1SP3E2 | 1SP3E2 |  |  |  | 1GP3E2 |  |
|  |  |  |  | Evidence |  | 3 | 3 |  |  |  | 3 |  |
|  |  |  |  | Focus |  | 4 | 4 |  |  |  | 1 |  |
|  |  |  |  | Polarity |  | 7 | 7 |  |  |  | 7 |  |
| 472 | 472 | 12702691\_224 | With high mutagenesis, only 2 of the Lac+ revertants escape associated null mutations. |I:\*\*1SP3E0| |L:\*\*1SP3E3| |T:\*\*1SP3E0| |
|  |  |  |  | Annotation |  | 1SP3E0 | 1SP3E3 |  |  |  | 1SP3E0 |  |
|  |  |  |  | Evidence |  | 1 | 4 |  |  |  | 1 |  |
|  |  |  |  | Focus |  | 4 | 4 |  |  |  | 4 |  |
|  |  |  |  | Polarity |  | 7 | 7 |  |  |  | 7 |  |
| 473 | 473 | 11333250\_405 | This is consistent with the predicted functions of different domains of R genes. |I:\*\*1SP3E0| |L:\*\*1GP3E0| |T:\*\*1GP3E0| |
|  |  |  |  | Annotation |  | 1SP3E0 | 1GP3E0 |  |  |  | 1GP3E0 |  |
|  |  |  |  | Evidence |  | 1 | 1 |  |  |  | 1 |  |
|  |  |  |  | Focus |  | 4 | 1 |  |  |  | 1 |  |
|  |  |  |  | Polarity |  | 7 | 7 |  |  |  | 7 |  |
| 474 | 474 | 12388614\_10 | These include exaggerated responses to stress, dopamine (DA) agonists, and NMDA antagonists, |I:\*\*1SP3E2+| which become apparent only after the animals reach adulthood (Lipska et al., 1993 ; Al-Amin et al., 2000 ). |I:\*\*2SP3E2| |L:\*\*1SP3E2+| |T:\*\*1SP3E2| |
|  |  |  |  | Annotation |  | 1SP3E2+ 2SP3E2 | 1SP3E2+ 1SP3E2+ |  |  |  | 1SP3E2 1SP3E2 |  |
|  |  |  |  | Evidence |  | 3 3 | 3 3 |  |  |  | 3 3 |  |
|  |  |  |  | Focus |  | 4 4 | 4 4 |  |  |  | 4 4 |  |
|  |  |  |  | Polarity |  | 7 7 | 7 7 |  |  |  | 7 7 |  |
| 475 | 475 | 11585798\_6 | Around the proximal module, there are two putative T protein-binding motifs (TTCACACTT). |I:\*\*1SP3E0| |L:\*\*1SP3E0| |T:\*\*1SP3E3| |
|  |  |  |  | Annotation |  | 1SP3E0 | 1SP3E0 |  |  |  | 1SP3E3 |  |
|  |  |  |  | Evidence |  | 1 | 1 |  |  |  | 4 |  |
|  |  |  |  | Focus |  | 4 | 4 |  |  |  | 4 |  |
|  |  |  |  | Polarity |  | 7 | 7 |  |  |  | 7 |  |
| 476 | 476 | 12235125\_186 | pRC/MV-MITF(wt) have been described earlier ( Hemesath et al., 1994) and the pcDNA3.1-HA/MITF(dn) was made by PCR amplification of the basic region (beginning ARALAKER) and to the last codon of mouse MITF(del) and ligated into pcDNA3.1 with an HA-tag in frame with the MITF coding region between the BamHI and EcoRI sites. |I:\*\*1MP3E3| |L:\*\*1SP3E2| |T:\*\*1MP3E3| |
|  |  |  |  | Annotation |  | 1MP3E3 | 1SP3E2 |  |  |  | 1MP3E3 |  |
|  |  |  |  | Evidence |  | 4 | 3 |  |  |  | 4 |  |
|  |  |  |  | Focus |  | 2 | 4 |  |  |  | 2 |  |
|  |  |  |  | Polarity |  | 7 | 7 |  |  |  | 7 |  |
| 477 | 477 | 9463377\_248 | Immunoblot analysis of fractions |I:\*\*1SGP3E3| obtained from Superose 6 fast-performance liquid chromatography (FPLC) gel filtration experiments (see Materials and methods) |I:\*\*2MP3E3| showed that coatomer from wild-type cells eluted in a peak corresponding to an apparent molecular mass of 700-800 000 Da (fractions 17 and 18 in Figure 9), as previously described by Hosobuchi et al. (1992) . |I:\*\*3SP3E3| |L:\*\*1SP3E23| |T:\*\*1MP3E3| |
|  |  |  |  | Annotation |  | 1SGP3E3 2MP3E3 3SP3E3 | 1SP3E23 1SP3E23 1SP3E23 |  |  |  | 1MP3E3 1MP3E3 1MP3E3 |  |
|  |  |  |  | Evidence |  | 4 4 4 | 3 3 3 |  |  |  | 4 4 4 |  |
|  |  |  |  | Focus |  | 5 2 4 | 4 4 4 |  |  |  | 2 2 2 |  |
|  |  |  |  | Polarity |  | 7 7 7 | 7 7 7 |  |  |  | 7 7 7 |  |
| 478 | 478 | 9287306\_21 | Receptor binding kinetics of multivalent ligands, such as antibodies ( , ), epidermal growth factor ( ), and growth hormone ( ), have been analyzed previously in great detail. |I:\*\*1SGP3E2| |L:\*\*1SP3E1| |T:\*\*1SP3E2| |
|  |  |  |  | Annotation |  | 1SGP3E2 | 1SP3E1 |  |  |  | 1SP3E2 |  |
|  |  |  |  | Evidence |  | 3 | 2 |  |  |  | 3 |  |
|  |  |  |  | Focus |  | 5 | 4 |  |  |  | 4 |  |
|  |  |  |  | Polarity |  | 7 | 7 |  |  |  | 7 |  |
| 479 | 479 | 9006934\_68 | To test whether the two genes are closely linked, |I:\*\*1SP0E3| we analyzed two DPC4-containing YACs, Y747A6 and Y945B11, for the presence of Smad2 sequences |I:\*\*2SGP0E3| by PCR. |I:\*\*3MP3E3| |L:\*\*1GMSP0E3| |T:\*\*1MP3E3| |
|  |  |  |  | Annotation |  | 1SP0E3 2SGP0E3 3MP3E3 | 1GMSP0E3 1GMSP0E3 1GMSP0E3 |  |  |  | 1MP3E3 1MP3E3 1MP3E3 |  |
|  |  |  |  | Evidence |  | 4 4 4 | 4 4 4 |  |  |  | 4 4 4 |  |
|  |  |  |  | Focus |  | 4 5 2 | 7 7 7 |  |  |  | 2 2 2 |  |
|  |  |  |  | Polarity |  | 4 4 7 | 4 4 4 |  |  |  | 7 7 7 |  |
| 480 | 480 | 12240900\_3 | Basal serum GH, GH-binding protein (GHBP), IGF-I, IGF-binding protein-3 (IGFBP-3) levels were determined as well as GH levels during GHRH stimulation. |I:\*\*1SP3E0| |L:\*\*1GMP3E0| |T:\*\*1MP3E3| |
|  |  |  |  | Annotation |  | 1SP3E0 | 1GMP3E0 |  |  |  | 1MP3E3 |  |
|  |  |  |  | Evidence |  | 1 | 1 |  |  |  | 4 |  |
|  |  |  |  | Focus |  | 4 | 3 |  |  |  | 2 |  |
|  |  |  |  | Polarity |  | 7 | 7 |  |  |  | 7 |  |
| 481 | 481 | 9724643\_75 | Total protein of 106 cells was size-fractionated by SDS-PAGE and Western blots were probed with Gbeta antibodies. |I:\*\*1MP3E0| |L:\*\*1MP3E3| |T:\*\*1MP3E3| |
|  |  |  |  | Annotation |  | 1MP3E0 | 1MP3E3 |  |  |  | 1MP3E3 |  |
|  |  |  |  | Evidence |  | 1 | 4 |  |  |  | 4 |  |
|  |  |  |  | Focus |  | 2 | 2 |  |  |  | 2 |  |
|  |  |  |  | Polarity |  | 7 | 7 |  |  |  | 7 |  |
| 482 | 482 | 11526011\_120 | Therefore, two specific primers were designed |I:\*\*1MP3E2| to screen an S. xylosus gene library stored as pools of plasmids ( 11). |I:\*\*2SGP3E2| |L:\*\*1SP3E2| |T:\*\*1MP3E3| |
|  |  |  |  | Annotation |  | 1MP3E2 2SGP3E2 | 1SP3E2 1SP3E2 |  |  |  | 1MP3E3 1MP3E3 |  |
|  |  |  |  | Evidence |  | 3 3 | 3 3 |  |  |  | 4 4 |  |
|  |  |  |  | Focus |  | 2 5 | 4 4 |  |  |  | 2 2 |  |
|  |  |  |  | Polarity |  | 7 7 | 7 7 |  |  |  | 7 7 |  |
| 483 | 483 | 11713265\_276 | The cells were incubated for an additional 1 h to allow all conjugated dextran to be taken up into lysosomes ( 21). |I:\*\*1MP3E2| |L:\*\*1MSP3E2| |T:\*\*1MP3E3| |
|  |  |  |  | Annotation |  | 1MP3E2 | 1MSP3E2 |  |  |  | 1MP3E3 |  |
|  |  |  |  | Evidence |  | 3 | 3 |  |  |  | 4 |  |
|  |  |  |  | Focus |  | 2 | 6 |  |  |  | 2 |  |
|  |  |  |  | Polarity |  | 7 | 7 |  |  |  | 7 |  |
| 484 | 484 | 9797273\_83 | Microplates were coated overnight at 4 degrees C with 25, 50, 100, and 200 ng of phage Q38. |I:\*\*1MP3E0| |L:\*\*1MP3E3| |T:\*\*1MP3E3| |
|  |  |  |  | Annotation |  | 1MP3E0 | 1MP3E3 |  |  |  | 1MP3E3 |  |
|  |  |  |  | Evidence |  | 1 | 4 |  |  |  | 4 |  |
|  |  |  |  | Focus |  | 2 | 2 |  |  |  | 2 |  |
|  |  |  |  | Polarity |  | 7 | 7 |  |  |  | 7 |  |
| 485 | 485 | 11864604\_99 | However, assuming a different rotamer for the side chain of F435 and an antagonist position of H12 to avoid steric contacts with F435, |I:\*\*1SP0E3| |L:\*\*1SP0E0| DES and 4-OHT could be fitted into the ERR3 cavity (Figures 3B and 3C) with conformations observed in the crystal structures of their respective complexes with the hER LBD (Shiau et al., 1998 ). |I:\*\*2SP3E3| |L:\*\*2SP1E23| |T:\*\*1SP3E2| |
|  |  |  |  | Annotation |  | 1SP0E3 2SP3E3 | 1SP0E0 2SP1E23 |  |  |  | 1SP3E2 1SP3E2 |  |
|  |  |  |  | Evidence |  | 4 4 | 1 3 |  |  |  | 3 3 |  |
|  |  |  |  | Focus |  | 4 4 | 4 4 |  |  |  | 4 4 |  |
|  |  |  |  | Polarity |  | 4 7 | 4 5 |  |  |  | 7 7 |  |
| 486 | 486 | 12559174\_359 | Diaminobenzidine-nickel peroxidase substrate solution was prepared according to manufacturer's instructions (Vector Laboratories Inc.) and dripped onto the surface of slides. |I:\*\*1MP3E0| |L:\*\*1MP3E3| |T:\*\*1MP3E3| |
|  |  |  |  | Annotation |  | 1MP3E0 | 1MP3E3 |  |  |  | 1MP3E3 |  |
|  |  |  |  | Evidence |  | 1 | 4 |  |  |  | 4 |  |
|  |  |  |  | Focus |  | 2 | 2 |  |  |  | 2 |  |
|  |  |  |  | Polarity |  | 7 | 7 |  |  |  | 7 |  |
| 487 | 487 | 12028628\_8 | At sacrifice, all upper-pole renal segments functioned, as shown by urography, |I:\*\*1SP3E0| |L:\*\*1SP3E3| and no urinomas or abscesses |I:\*\*2SN3E0| were found. |I:\*\*3SP3E0| |L:\*\*2SN3E3| |T:\*\*1MP3E3| |
|  |  |  |  | Annotation |  | 1SP3E0 2SN3E0 3SP3E0 | 1SP3E3 2SN3E3 2SN3E3 |  |  |  | 1MP3E3 1MP3E3 1MP3E3 |  |
|  |  |  |  | Evidence |  | 1 1 1 | 4 4 4 |  |  |  | 4 4 4 |  |
|  |  |  |  | Focus |  | 4 4 4 | 4 4 4 |  |  |  | 2 2 2 |  |
|  |  |  |  | Polarity |  | 7 1 7 | 7 1 1 |  |  |  | 7 7 7 |  |
| 488 | 488 | 10948161\_122 | The H. pylori-resistant AGS derivatives were completely resistant to the moderate apoptosis induced by C. jejuni (1.5 times the control values) and partially resistant to apoptosis induced by S. boydii (control cells, 42% apoptotic; HS3, 28.4% apoptotic; HS4, 28.3% apoptotic). |I:\*\*1SP3E0| |L:\*\*1SP3E3| |T:\*\*1SP3E3+| |
|  |  |  |  | Annotation |  | 1SP3E0 | 1SP3E3 |  |  |  | 1SP3E3+ |  |
|  |  |  |  | Evidence |  | 1 | 4 |  |  |  | 4 |  |
|  |  |  |  | Focus |  | 4 | 4 |  |  |  | 4 |  |
|  |  |  |  | Polarity |  | 7 | 7 |  |  |  | 7 |  |
| 489 | 489 | 10520993\_8 | We conclude that Tsix regulates Xist in cis and determines X chromosome choice |I:\*\*1SP3E3| without affecting silencing. |I:\*\*2SN3E3| |L:\*\*1SP3E3| |T:\*\*1SP3E0| |
|  |  |  |  | Annotation |  | 1SP3E3 2SN3E3 | 1SP3E3 1SP3E3 |  |  |  | 1SP3E0 1SP3E0 |  |
|  |  |  |  | Evidence |  | 4 4 | 4 4 |  |  |  | 1 1 |  |
|  |  |  |  | Focus |  | 4 4 | 4 4 |  |  |  | 4 4 |  |
|  |  |  |  | Polarity |  | 7 1 | 7 7 |  |  |  | 7 7 |  |
| 490 | 490 | 11070169\_103 | The figure shows average plus-or-minus standard deviation of four experiments. |I:\*\*1MGP3E3| |L:\*\*1GP3E3| |T:\*\*1SP3E0| |
|  |  |  |  | Annotation |  | 1MGP3E3 | 1GP3E3 |  |  |  | 1SP3E0 |  |
|  |  |  |  | Evidence |  | 4 | 4 |  |  |  | 1 |  |
|  |  |  |  | Focus |  | 3 | 1 |  |  |  | 4 |  |
|  |  |  |  | Polarity |  | 7 | 7 |  |  |  | 7 |  |
| 491 | 491 | 10893270\_19 | This type of Wnt-1 signal transduction is required for Wnt-induced cell transformation, although alternative Wnt and frizzled signaling pathways also have been observed recently ( Rocheleau et al. 1997 ; Slusarski et al. 1997 ; Strutt et al. 1997 ; Sheldahl et al. 1999 ). |I:\*\*1SP3E2| |L:\*\*1SP3E2| |T:\*\*1SP3E2| |
|  |  |  |  | Annotation |  | 1SP3E2 | 1SP3E2 |  |  |  | 1SP3E2 |  |
|  |  |  |  | Evidence |  | 3 | 3 |  |  |  | 3 |  |
|  |  |  |  | Focus |  | 4 | 4 |  |  |  | 4 |  |
|  |  |  |  | Polarity |  | 7 | 7 |  |  |  | 7 |  |
| 492 | 492 | 10629215\_288 | We also found that caveolins with alanine substitutions in 91tftvtkywfY100 were Triton X-100 soluble. |I:\*\*1SP3E3| |L:\*\*1SP3E3| |T:\*\*1SP3E3| |
|  |  |  |  | Annotation |  | 1SP3E3 | 1SP3E3 |  |  |  | 1SP3E3 |  |
|  |  |  |  | Evidence |  | 4 | 4 |  |  |  | 4 |  |
|  |  |  |  | Focus |  | 4 | 4 |  |  |  | 4 |  |
|  |  |  |  | Polarity |  | 7 | 7 |  |  |  | 7 |  |
| 493 | 493 | 0009835588\_64 | The samples of the other three plants were treated as described under |I:\*\*ERROR| |L:\*\*ERROR| |T:\*\*ERROR| In addition, samples of Ede loamy sand bulk soil were collected and stored at 20 degrees C |I:\*\*1MP3E0| in order to assess the putative rhizosphere effects |I:\*\*2SP0E0| by comparing the patterns obtained from bulk and rhizosphere soils. |I:\*\*3MP3E0| |L:\*\*1MP3E3| |T:\*\*1MP3E3| |
|  |  |  |  | Annotation |  | ERROR 1MP3E0 2SP0E0 3MP3E0 | ERROR 1MP3E3 1MP3E3 1MP3E3 |  |  |  | ERROR 1MP3E3 1MP3E3 1MP3E3 |  |
|  |  |  |  | Evidence |  | -1000 1 1 1 | -1000 4 4 4 |  |  |  | -1000 4 4 4 |  |
|  |  |  |  | Focus |  | -1000 2 4 2 | -1000 2 2 2 |  |  |  | -1000 2 2 2 |  |
|  |  |  |  | Polarity |  | -1000 7 4 7 | -1000 7 7 7 |  |  |  | -1000 7 7 7 |  |
| 494 | 494 | 12221698\_3 | After each period of incubation, the surface electrical resistivity of the sample fabrics was measured |I:\*\*1MP3E0| to monitor the changes caused by the incubation. |I:\*\*2SGP0E0| |L:\*\*1GMP3E3| |T:\*\*1MP3E3| |
|  |  |  |  | Annotation |  | 1MP3E0 2SGP0E0 | 1GMP3E3 1GMP3E3 |  |  |  | 1MP3E3 1MP3E3 |  |
|  |  |  |  | Evidence |  | 1 1 | 4 4 |  |  |  | 4 4 |  |
|  |  |  |  | Focus |  | 2 5 | 3 3 |  |  |  | 2 2 |  |
|  |  |  |  | Polarity |  | 7 4 | 7 7 |  |  |  | 7 7 |  |
| 495 | 495 | 11014804\_33 | The general transcription factors (TF) IIF and TFIIH, which are essential for PIC assembly and initiation, also regulate elongation. |I:\*\*1SP3E0| |L:\*\*1SP3E0| |T:\*\*1SP3E0| |
|  |  |  |  | Annotation |  | 1SP3E0 | 1SP3E0 |  |  |  | 1SP3E0 |  |
|  |  |  |  | Evidence |  | 1 | 1 |  |  |  | 1 |  |
|  |  |  |  | Focus |  | 4 | 4 |  |  |  | 4 |  |
|  |  |  |  | Polarity |  | 7 | 7 |  |  |  | 7 |  |
| 496 | 496 | 10618267\_139 | Probed against these negative sera, |I:\*\*1SGP0E0| the diagnostic immunoblot presented here demonstrated high specificity. |I:\*\*2SP3E0| |L:\*\*1SP3E3| |T:\*\*1GP3E0| |
|  |  |  |  | Annotation |  | 1SGP0E0 2SP3E0 | 1SP3E3 1SP3E3 |  |  |  | 1GP3E0 1GP3E0 |  |
|  |  |  |  | Evidence |  | 1 1 | 4 4 |  |  |  | 1 1 |  |
|  |  |  |  | Focus |  | 5 4 | 4 4 |  |  |  | 1 1 |  |
|  |  |  |  | Polarity |  | 4 7 | 7 7 |  |  |  | 7 7 |  |
| 497 | 497 | 9694885\_142 | The involvement of the granzyme pathway in hCTL-induced killing was confirmed |I:\*\*1SP3E0| by sensitivity to calcium chelation. |I:\*\*2MP3E0| |L:\*\*1SP3E0| |T:\*\*1MP3E3| |
|  |  |  |  | Annotation |  | 1SP3E0 2MP3E0 | 1SP3E0 1SP3E0 |  |  |  | 1MP3E3 1MP3E3 |  |
|  |  |  |  | Evidence |  | 1 1 | 1 1 |  |  |  | 4 4 |  |
|  |  |  |  | Focus |  | 4 2 | 4 4 |  |  |  | 2 2 |  |
|  |  |  |  | Polarity |  | 7 7 | 7 7 |  |  |  | 7 7 |  |
| 498 | 498 | 11971135\_2 | We used a rice whole-cell extract in vitro transcription system |I:\*\*1MP3E3| to characterize the functional interactions of recombinant plant TBP and TFIIB. |I:\*\*2SGP0E3| |L:\*\*1GMP3E3| |T:\*\*1MP3E3| |
|  |  |  |  | Annotation |  | 1MP3E3 2SGP0E3 | 1GMP3E3 1GMP3E3 |  |  |  | 1MP3E3 1MP3E3 |  |
|  |  |  |  | Evidence |  | 4 4 | 4 4 |  |  |  | 4 4 |  |
|  |  |  |  | Focus |  | 2 5 | 3 3 |  |  |  | 2 2 |  |
|  |  |  |  | Polarity |  | 7 4 | 7 7 |  |  |  | 7 7 |  |
| 499 | 499 | 9539126\_169 | Spike pairs that occurred synchronously within 1 ms of each other are drawn in bold. |I:\*\*1MGP3E3| |L:\*\*1MP3E3| |T:\*\*1MP3E3| |
|  |  |  |  | Annotation |  | 1MGP3E3 | 1MP3E3 |  |  |  | 1MP3E3 |  |
|  |  |  |  | Evidence |  | 4 | 4 |  |  |  | 4 |  |
|  |  |  |  | Focus |  | 3 | 2 |  |  |  | 2 |  |
|  |  |  |  | Polarity |  | 7 | 7 |  |  |  | 7 |  |
| 500 | 500 | 9684755\_181 | The areas of the 9-oxononanoyl cholesterol peak obtained from the PBS or LDL matrix are compared in Figure 6. |I:\*\*1SGP3E3| |L:\*\*1GSP3E3| |T:\*\*1MP3E3| |
|  |  |  |  | Annotation |  | 1SGP3E3 | 1GSP3E3 |  |  |  | 1MP3E3 |  |
|  |  |  |  | Evidence |  | 4 | 4 |  |  |  | 4 |  |
|  |  |  |  | Focus |  | 5 | 5 |  |  |  | 2 |  |
|  |  |  |  | Polarity |  | 7 | 7 |  |  |  | 7 |  |
| 501 | 501 | 11707441\_145 | Cells with neurites longer than cell body length were scored as neurite bearing cells, and at least 150 cells expressing EGFP, excluding floating or dead cells, were counted in each experiment. |I:\*\*1MP3E0| |L:\*\*1MP3E3| |T:\*\*1MP3E3| |
|  |  |  |  | Annotation |  | 1MP3E0 | 1MP3E3 |  |  |  | 1MP3E3 |  |
|  |  |  |  | Evidence |  | 1 | 4 |  |  |  | 4 |  |
|  |  |  |  | Focus |  | 2 | 2 |  |  |  | 2 |  |
|  |  |  |  | Polarity |  | 7 | 7 |  |  |  | 7 |  |
| 502 | 502 | 9390512\_49 | It should be noted, however, that a mode of transmission consistent with multiple, common, interacting, and weakly penetrant mutations does not necessarily preclude the possibility of a single locus with a very large effect (a Mendelian-type gene) operating in a rare subset of cases. |I:\*\*1SN3E0| |L:\*\*1GSN3E0| |T:\*\*1GN3E0| |
|  |  |  |  | Annotation |  | 1SN3E0 | 1GSN3E0 |  |  |  | 1GN3E0 |  |
|  |  |  |  | Evidence |  | 1 | 1 |  |  |  | 1 |  |
|  |  |  |  | Focus |  | 4 | 5 |  |  |  | 1 |  |
|  |  |  |  | Polarity |  | 1 | 1 |  |  |  | 1 |  |
| 503 | 503 | 9390556\_69 | We found that 3 out of the 192 ES cell colonies contained the appropriately modified Msh6 locus. |I:\*\*1SGP3E3| |L:\*\*1SP3E3| |T:\*\*1SP3E3| |
|  |  |  |  | Annotation |  | 1SGP3E3 | 1SP3E3 |  |  |  | 1SP3E3 |  |
|  |  |  |  | Evidence |  | 4 | 4 |  |  |  | 4 |  |
|  |  |  |  | Focus |  | 5 | 4 |  |  |  | 4 |  |
|  |  |  |  | Polarity |  | 7 | 7 |  |  |  | 7 |  |
| 504 | 504 | 11466282\_153 | The deduced amino acid sequence of MrpA was subjected to a BLAST search ( 1). |I:\*\*1MP3E2| |L:\*\*1GP3E2| |T:\*\*1SP3E3| |
|  |  |  |  | Annotation |  | 1MP3E2 | 1GP3E2 |  |  |  | 1SP3E3 |  |
|  |  |  |  | Evidence |  | 3 | 3 |  |  |  | 4 |  |
|  |  |  |  | Focus |  | 2 | 1 |  |  |  | 4 |  |
|  |  |  |  | Polarity |  | 7 | 7 |  |  |  | 7 |  |
| 505 | 505 | 9628898\_29 | Ac-YVAD-cmk, also known as ICE inhibitor II, was from both Calbiochem (La Jolla, CA) and Bachem California (Torrance, CA). |I:\*\*1MP3E0| |L:\*\*1GMP3E3| |T:\*\*1MP3E3| |
|  |  |  |  | Annotation |  | 1MP3E0 | 1GMP3E3 |  |  |  | 1MP3E3 |  |
|  |  |  |  | Evidence |  | 1 | 4 |  |  |  | 4 |  |
|  |  |  |  | Focus |  | 2 | 3 |  |  |  | 2 |  |
|  |  |  |  | Polarity |  | 7 | 7 |  |  |  | 7 |  |
| 506 | 506 | 9341199\_33 | The origin of the 7.0-kb transcript remains to be determined. |I:\*\*1SGP0E0| |L:\*\*1SP0E0| |T:\*\*1SN3E3| |
|  |  |  |  | Annotation |  | 1SGP0E0 | 1SP0E0 |  |  |  | 1SN3E3 |  |
|  |  |  |  | Evidence |  | 1 | 1 |  |  |  | 4 |  |
|  |  |  |  | Focus |  | 5 | 4 |  |  |  | 4 |  |
|  |  |  |  | Polarity |  | 4 | 4 |  |  |  | 1 |  |
| 507 | 507 | 9804770\_359 | One possibility is that NE also activates inhibitory signals, such as a protein phosphatase(s), |L:\*\*1SP3E0+| that could limit the activation of Erk. |L:\*\*2SP3E0-| |I:\*\*1SP1E3| |T:\*\*1SP2E3| |
|  |  |  |  | Annotation |  | 1SP1E3 1SP1E3 | 1SP3E0+ 2SP3E0- |  |  |  | 1SP2E3 1SP2E3 |  |
|  |  |  |  | Evidence |  | 4 4 | 1 1 |  |  |  | 4 4 |  |
|  |  |  |  | Focus |  | 4 4 | 4 4 |  |  |  | 4 4 |  |
|  |  |  |  | Polarity |  | 5 5 | 7 7 |  |  |  | 6 6 |  |
| 508 | 508 | 10473520\_100 | Following the identification of BDV RNA in peripheral blood mononuclear cells (PBMC) of experimentally infected rats ( 23), Bode et al. ( 5) reported finding BDV protein in CD14+ PBMC and RNA in PBMC from psychiatric patients. |I:\*\*1SP3E2| |L:\*\*1SP3E2| |T:\*\*1SP3E2| |
|  |  |  |  | Annotation |  | 1SP3E2 | 1SP3E2 |  |  |  | 1SP3E2 |  |
|  |  |  |  | Evidence |  | 3 | 3 |  |  |  | 3 |  |
|  |  |  |  | Focus |  | 4 | 4 |  |  |  | 4 |  |
|  |  |  |  | Polarity |  | 7 | 7 |  |  |  | 7 |  |
| 509 | 509 | 9341128\_34 | This indicated that the two peptides of Mr 29,000 (peptide A) and Mr 25,000 (peptide B) stemmed from the DHOase subunit and that they resulted from the removal of C-terminal fragments of approximately 8,000 and 13,000, respectively |I:\*\*1SP3E3| (according to the mechanism of the cleavage reaction shown on Scheme 1, |I:\*\*2SGP3E3| these two small fragments should have blocked N termini; |I:\*\*3SP3E3| this is confirmed |I:\*\*4SGP3E3| by unsuccessful attempts to sequence the N terminus of the fragment 13,000. |I:\*\*5SN3E3| |L:\*\*1SP3E3| |T:\*\*1SP3E0| |
|  |  |  |  | Annotation |  | 1SP3E3 2SGP3E3 3SP3E3 4SGP3E3 5SN3E3 | 1SP3E3 1SP3E3 1SP3E3 1SP3E3 1SP3E3 |  |  |  | 1SP3E0 1SP3E0 1SP3E0 1SP3E0 1SP3E0 |  |
|  |  |  |  | Evidence |  | 4 4 4 4 4 | 4 4 4 4 4 |  |  |  | 1 1 1 1 1 |  |
|  |  |  |  | Focus |  | 4 5 4 5 4 | 4 4 4 4 4 |  |  |  | 4 4 4 4 4 |  |
|  |  |  |  | Polarity |  | 7 7 7 7 1 | 7 7 7 7 7 |  |  |  | 7 7 7 7 7 |  |
| 510 | 510 | 12711618\_8 | The impaired anti-mitogenic activity of GCS in cells maintained on collagen |I:\*\*1SP3E0| may be due to a lack of efficacy against the collagen-amplified mitogenesis, |I:\*\*2SP1E3| |L:\*\*1SP1E0| |T:\*\*1SP1E1| rather than any defect in responsiveness that is specific to glucocorticoid receptor mechanisms. |I:\*\*3SN1E3| |L:\*\*2SN3E0| |T:\*\*2SN3E1| |
|  |  |  |  | Annotation |  | 1SP3E0 2SP1E3 3SN1E3 | 1SP1E0 1SP1E0 2SN3E0 |  |  |  | 1SP1E1 1SP1E1 2SN3E1 |  |
|  |  |  |  | Evidence |  | 1 4 4 | 1 1 1 |  |  |  | 2 2 2 |  |
|  |  |  |  | Focus |  | 4 4 4 | 4 4 4 |  |  |  | 4 4 4 |  |
|  |  |  |  | Polarity |  | 7 5 3 | 5 5 1 |  |  |  | 5 5 1 |  |
| 511 | 511 | 12530969\_353 | We thank Tom Januszewski for the EM work. |I:\*\*1GP3E3| |L:\*\*1GP3E3| |T:\*\*1GP3E3| |
|  |  |  |  | Annotation |  | 1GP3E3 | 1GP3E3 |  |  |  | 1GP3E3 |  |
|  |  |  |  | Evidence |  | 4 | 4 |  |  |  | 4 |  |
|  |  |  |  | Focus |  | 1 | 1 |  |  |  | 1 |  |
|  |  |  |  | Polarity |  | 7 | 7 |  |  |  | 7 |  |
| 512 | 512 | 9741687\_148 | Visceral adiposity is closely related to impaired lipid metabolism ( 28), insulin resistance ( 29), and high blood pressure ( 30). |I:\*\*1SP3E2| |L:\*\*1GSP2E2| |T:\*\*1SP3E2| |
|  |  |  |  | Annotation |  | 1SP3E2 | 1GSP2E2 |  |  |  | 1SP3E2 |  |
|  |  |  |  | Evidence |  | 3 | 3 |  |  |  | 3 |  |
|  |  |  |  | Focus |  | 4 | 5 |  |  |  | 4 |  |
|  |  |  |  | Polarity |  | 7 | 6 |  |  |  | 7 |  |
| 513 | 513 | 9651509\_146 | Differences in the ability of Xwnt8 and Xfz8 to induce organizer markers |I:\*\*1SP3E3| |L:\*\*1SP3E0| led us to examine if the effect of Xfz8 requires functional Wnt signal transduction pathway. |I:\*\*2SP0E3| |L:\*\*2SP0E3| |T:\*\*1SP3E1| |
|  |  |  |  | Annotation |  | 1SP3E3 2SP0E3 | 1SP3E0 2SP0E3 |  |  |  | 1SP3E1 1SP3E1 |  |
|  |  |  |  | Evidence |  | 4 4 | 1 4 |  |  |  | 2 2 |  |
|  |  |  |  | Focus |  | 4 4 | 4 4 |  |  |  | 4 4 |  |
|  |  |  |  | Polarity |  | 7 4 | 7 4 |  |  |  | 7 7 |  |
| 514 | 514 | 11472937\_133 | By genetic complementation using plasmid pEH20, which carries the native pmi gene |T:\*\*1MP3E3| the ability of the mutant to produce PTT was restored (detected by a biological assay [ 1), |T:\*\*2SP3E1| |I:\*\*1SP3E2| |L:\*\*1SP3E2| indicating that the insertion of the resistance cassette did not prevent the transcription of the PTT biosynthetic genes located downstream. |T:\*\*3SN3E1| |I:\*\*2SN3E3| |L:\*\*2SN1E0| |
|  |  |  |  | Annotation |  | 1SP3E2 1SP3E2 2SN3E3 | 1SP3E2 1SP3E2 2SN1E0 |  |  |  | 1MP3E3 2SP3E1 3SN3E1 |  |
|  |  |  |  | Evidence |  | 3 3 4 | 3 3 1 |  |  |  | 4 2 2 |  |
|  |  |  |  | Focus |  | 4 4 4 | 4 4 4 |  |  |  | 2 4 4 |  |
|  |  |  |  | Polarity |  | 7 7 1 | 7 7 3 |  |  |  | 7 7 1 |  |
| 515 | 515 | 11254617\_78 | Macrophages were obtained from CBA/Ca mice by peritoneal lavage with 3 ml of ice-cold RPMI 1640 medium containing 5 U of heparin ml 1. |I:\*\*1MP3E0| |L:\*\*1MP3E3| |T:\*\*1MP3E3| |
|  |  |  |  | Annotation |  | 1MP3E0 | 1MP3E3 |  |  |  | 1MP3E3 |  |
|  |  |  |  | Evidence |  | 1 | 4 |  |  |  | 4 |  |
|  |  |  |  | Focus |  | 2 | 2 |  |  |  | 2 |  |
|  |  |  |  | Polarity |  | 7 | 7 |  |  |  | 7 |  |
| 516 | 516 | 9844016\_43 | Total RNA was isolated and reverse transcribed from monkey cerebral cortex, amplified by PCR, gel-fractionated, and subcloned into the pBS transcription vector (Stratagene). |I:\*\*1MP3E0| |L:\*\*1MP3E3| |T:\*\*1MP3E3| |
|  |  |  |  | Annotation |  | 1MP3E0 | 1MP3E3 |  |  |  | 1MP3E3 |  |
|  |  |  |  | Evidence |  | 1 | 4 |  |  |  | 4 |  |
|  |  |  |  | Focus |  | 2 | 2 |  |  |  | 2 |  |
|  |  |  |  | Polarity |  | 7 | 7 |  |  |  | 7 |  |
| 517 | 517 | 12175183\_5 | These features enabled CE-EC users to polish the working electrode and reassemble the EC cell as in HPLC-EC. |I:\*\*1MGP3E0| |L:\*\*1GP3E0| |T:\*\*1MP3E3| |
|  |  |  |  | Annotation |  | 1MGP3E0 | 1GP3E0 |  |  |  | 1MP3E3 |  |
|  |  |  |  | Evidence |  | 1 | 1 |  |  |  | 4 |  |
|  |  |  |  | Focus |  | 3 | 1 |  |  |  | 2 |  |
|  |  |  |  | Polarity |  | 7 | 7 |  |  |  | 7 |  |
| 518 | 518 | 12127416\_7 | The most resistant piglets had a higher number of circulating neutrophils |T:\*\*1SP3E1-| and better polymorphonuclear neutrophils (PMNs) function |T:\*\*2SP3E1| |I:\*\*1SP3E0+| |L:\*\*1SP3E3+| but a lower mitogenic response of lymphocytes both pre- and post-infection and a lower antibody response. |T:\*\*3SP3E1-| |I:\*\*2SP3E0-| |L:\*\*2SP3E0-| |
|  |  |  |  | Annotation |  | 1SP3E0+ 1SP3E0+ 2SP3E0- | 1SP3E3+ 1SP3E3+ 2SP3E0- |  |  |  | 1SP3E1- 2SP3E1 3SP3E1- |  |
|  |  |  |  | Evidence |  | 1 1 1 | 4 4 1 |  |  |  | 2 2 2 |  |
|  |  |  |  | Focus |  | 4 4 4 | 4 4 4 |  |  |  | 4 4 4 |  |
|  |  |  |  | Polarity |  | 7 7 7 | 7 7 7 |  |  |  | 7 7 7 |  |
| 519 | 519 | 12033758\_2 | It is seen in patients with healthy joints as well as those with somatic dysfunction. |I:\*\*1SP3E0| |L:\*\*1GP3E3| |T:\*\*1GP3E0| |
|  |  |  |  | Annotation |  | 1SP3E0 | 1GP3E3 |  |  |  | 1GP3E0 |  |
|  |  |  |  | Evidence |  | 1 | 4 |  |  |  | 1 |  |
|  |  |  |  | Focus |  | 4 | 1 |  |  |  | 1 |  |
|  |  |  |  | Polarity |  | 7 | 7 |  |  |  | 7 |  |
| 520 | 520 | 9548586\_39 | Reverse transcription (RT) PCR, cloning and sequencing |I:\*\*ERROR| |L:\*\*ERROR| |T:\*\*ERROR| Total cellular RNA was extracted with RNAzol solution (Biogenesis, Bournemouth, UK) and reverse transcribed using Moloney murine leukemia virus reverse transcriptase and an oligo-dT primer (Life Sciences). |I:\*\*1MP3E0| |L:\*\*1MP3E3| |T:\*\*1MP3E3| |
|  |  |  |  | Annotation |  | ERROR 1MP3E0 | ERROR 1MP3E3 |  |  |  | ERROR 1MP3E3 |  |
|  |  |  |  | Evidence |  | -1000 1 | -1000 4 |  |  |  | -1000 4 |  |
|  |  |  |  | Focus |  | -1000 2 | -1000 2 |  |  |  | -1000 2 |  |
|  |  |  |  | Polarity |  | -1000 7 | -1000 7 |  |  |  | -1000 7 |  |
| 521 | 521 | 12818156\_6 | Thus, intracellular neutralization by dIgA limits the acute local inflammation |L:\*\*1SP3E0-| induced by proinflammatory pathogen-associated molecular patterns such as LPS. |L:\*\*2SP3E0+| |I:\*\*1SP3E0| |T:\*\*1SP3E3| |
|  |  |  |  | Annotation |  | 1SP3E0 1SP3E0 | 1SP3E0- 2SP3E0+ |  |  |  | 1SP3E3 1SP3E3 |  |
|  |  |  |  | Evidence |  | 1 1 | 1 1 |  |  |  | 4 4 |  |
|  |  |  |  | Focus |  | 4 4 | 4 4 |  |  |  | 4 4 |  |
|  |  |  |  | Polarity |  | 7 7 | 7 7 |  |  |  | 7 7 |  |
| 522 | 522 | 10799456\_136 | Similar results were obtained |I:\*\*1SGP3E0| IgA anti-PT and IgA anti-FHA were ranked according to the results of ELISA 0. |I:\*\*2MP3E0| |L:\*\*1SP3E0| |T:\*\*1SP3E1| |
|  |  |  |  | Annotation |  | 1SGP3E0 2MP3E0 | 1SP3E0 1SP3E0 |  |  |  | 1SP3E1 1SP3E1 |  |
|  |  |  |  | Evidence |  | 1 1 | 1 1 |  |  |  | 2 2 |  |
|  |  |  |  | Focus |  | 5 2 | 4 4 |  |  |  | 4 4 |  |
|  |  |  |  | Polarity |  | 7 7 | 7 7 |  |  |  | 7 7 |  |
| 523 | 523 | 10978273\_116 | The set of three plates with between 10 and 100 colonies was counted and used to estimate the total number of colony-forming units present in each infected cornea. |I:\*\*1MP3E0| |L:\*\*1MP3E3| |T:\*\*1MP3E3| |
|  |  |  |  | Annotation |  | 1MP3E0 | 1MP3E3 |  |  |  | 1MP3E3 |  |
|  |  |  |  | Evidence |  | 1 | 4 |  |  |  | 4 |  |
|  |  |  |  | Focus |  | 2 | 2 |  |  |  | 2 |  |
|  |  |  |  | Polarity |  | 7 | 7 |  |  |  | 7 |  |
| 524 | 524 | 10619846\_7 | Furthermore, SAPAP1/GKAP, which binds specifically to the GK domain of membrane-associated guanylate kinases, prevented the SAP97-induced sensitization. |I:\*\*1SP3E0| |L:\*\*1SP3E0-| |T:\*\*1SN3E1| |
|  |  |  |  | Annotation |  | 1SP3E0 | 1SP3E0- |  |  |  | 1SN3E1 |  |
|  |  |  |  | Evidence |  | 1 | 1 |  |  |  | 2 |  |
|  |  |  |  | Focus |  | 4 | 4 |  |  |  | 4 |  |
|  |  |  |  | Polarity |  | 7 | 7 |  |  |  | 1 |  |
| 525 | 525 | 9890971\_188 | Significantly, expression of crmA inhibited apoptosis |L:\*\*1SP3E0-| induced by Abeta1-42. |L:\*\*2SP3E3+| |I:\*\*1SP3E0| |T:\*\*1SP3E1+| |
|  |  |  |  | Annotation |  | 1SP3E0 1SP3E0 | 1SP3E0- 2SP3E3+ |  |  |  | 1SP3E1+ 1SP3E1+ |  |
|  |  |  |  | Evidence |  | 1 1 | 1 4 |  |  |  | 2 2 |  |
|  |  |  |  | Focus |  | 4 4 | 4 4 |  |  |  | 4 4 |  |
|  |  |  |  | Polarity |  | 7 7 | 7 7 |  |  |  | 7 7 |  |
| 526 | 526 | 9813087\_309 | First, the CFTR mutant delta259-M265V demonstrated near wild-type rates of CFTR-modulated ATP release |I:\*\*1SP3E0| |T:\*\*1SP3E1| despite its inability to conduct Cl. |I:\*\*2SN3E0| |L:\*\*1SP3E0| |T:\*\*2SN3E1| |
|  |  |  |  | Annotation |  | 1SP3E0 2SN3E0 | 1SP3E0 1SP3E0 |  |  |  | 1SP3E1 2SN3E1 |  |
|  |  |  |  | Evidence |  | 1 1 | 1 1 |  |  |  | 2 2 |  |
|  |  |  |  | Focus |  | 4 4 | 4 4 |  |  |  | 4 4 |  |
|  |  |  |  | Polarity |  | 7 1 | 7 7 |  |  |  | 7 1 |  |
| 527 | 527 | 12213835\_272 | Spindle positioning has been speculated to involve a balancing of forces generated either by growing astral microtubules pushing against the cell cortex or by cortically bound motor complexes containing dynein and Lis1 pulling on astral microtubules ( Faulkner et al., 2000; Segal and Bloom, 2001; Dujardin and Vallee, 2002). |I:\*\*1SP1E2| |L:\*\*1SP0E2| |T:\*\*1SP3E2| |
|  |  |  |  | Annotation |  | 1SP1E2 | 1SP0E2 |  |  |  | 1SP3E2 |  |
|  |  |  |  | Evidence |  | 3 | 3 |  |  |  | 3 |  |
|  |  |  |  | Focus |  | 4 | 4 |  |  |  | 4 |  |
|  |  |  |  | Polarity |  | 5 | 4 |  |  |  | 7 |  |
| 528 | 528 | 11906793\_4 | GDNF immunoreactivity was localized to RGCs, photoreceptors, and retinal pigment epithelial cells. |I:\*\*1SP3E0| |L:\*\*1SP3E0| |T:\*\*1SP3E1| |
|  |  |  |  | Annotation |  | 1SP3E0 | 1SP3E0 |  |  |  | 1SP3E1 |  |
|  |  |  |  | Evidence |  | 1 | 1 |  |  |  | 2 |  |
|  |  |  |  | Focus |  | 4 | 4 |  |  |  | 4 |  |
|  |  |  |  | Polarity |  | 7 | 7 |  |  |  | 7 |  |
| 529 | 529 | 12351398\_6 | Accordingly, the expression of CD158 by TILs may favor tumor cell escape to the immune response. |I:\*\*1SP1E0| |L:\*\*1SP1E0| |T:\*\*1SP1E1| |
|  |  |  |  | Annotation |  | 1SP1E0 | 1SP1E0 |  |  |  | 1SP1E1 |  |
|  |  |  |  | Evidence |  | 1 | 1 |  |  |  | 2 |  |
|  |  |  |  | Focus |  | 4 | 4 |  |  |  | 4 |  |
|  |  |  |  | Polarity |  | 5 | 5 |  |  |  | 5 |  |
| 530 | 530 | 11266442\_57 | The mitochondria were lysed and immunoprecipitated with an anti-Bax antibody (N20) and the amount of coimmunoprecipitated VDAC was estimated by Western blot analysis. |I:\*\*1MP3E0| |L:\*\*1MP3E3| |T:\*\*1MP3E3| |
|  |  |  |  | Annotation |  | 1MP3E0 | 1MP3E3 |  |  |  | 1MP3E3 |  |
|  |  |  |  | Evidence |  | 1 | 4 |  |  |  | 4 |  |
|  |  |  |  | Focus |  | 2 | 2 |  |  |  | 2 |  |
|  |  |  |  | Polarity |  | 7 | 7 |  |  |  | 7 |  |
| 531 | 531 | 9103521\_232 | The specificity of sotalol's blocking action has been less clear. |I:\*\*1SP3E0| |L:\*\*1SN1E0| |T:\*\*1MP3E1| |
|  |  |  |  | Annotation |  | 1SP3E0 | 1SN1E0 |  |  |  | 1MP3E1 |  |
|  |  |  |  | Evidence |  | 1 | 1 |  |  |  | 2 |  |
|  |  |  |  | Focus |  | 4 | 4 |  |  |  | 2 |  |
|  |  |  |  | Polarity |  | 7 | 3 |  |  |  | 7 |  |
| 532 | 532 | 11545071\_421 | SHOSHANI, J. and M. C. MCKENNA, 1998 |L:\*\*1GP3E3| Higher taxonomic relationships among extant mammals based on morphology, with selected comparisons of results from molecular data. |L:\*\*2GP3E3| |I:\*\*1SGP3E2| |T:\*\*1SP3E0| |
|  |  |  |  | Annotation |  | 1SGP3E2 1SGP3E2 | 1GP3E3 2GP3E3 |  |  |  | 1SP3E0 1SP3E0 |  |
|  |  |  |  | Evidence |  | 3 3 | 4 4 |  |  |  | 1 1 |  |
|  |  |  |  | Focus |  | 5 5 | 1 1 |  |  |  | 4 4 |  |
|  |  |  |  | Polarity |  | 7 7 | 7 7 |  |  |  | 7 7 |  |
| 533 | 533 | 12115865\_3 | The stem cells of the gastrointestinal tract are as yet undefined, |I:\*\*1SN3E0| |L:\*\*1SP0E0| |T:\*\*1SN3E1| although it is generally agreed that they are located within a 'niche' in the intestinal crypts and gastric glands. |I:\*\*2SP2E1| |L:\*\*2SP1E0| |T:\*\*2SP3E1| |
|  |  |  |  | Annotation |  | 1SN3E0 2SP2E1 | 1SP0E0 2SP1E0 |  |  |  | 1SN3E1 2SP3E1 |  |
|  |  |  |  | Evidence |  | 1 2 | 1 1 |  |  |  | 2 2 |  |
|  |  |  |  | Focus |  | 4 4 | 4 4 |  |  |  | 4 4 |  |
|  |  |  |  | Polarity |  | 1 6 | 4 5 |  |  |  | 1 7 |  |
| 534 | 534 | 8805362\_43 | Some CHO mutant cell lines generated by Krieger and co-workers [19,20] are defective in both N- and O-linked glycosylation. |I:\*\*1SGP3E3| |L:\*\*1SP3E2| |T:\*\*1SP3E2| |
|  |  |  |  | Annotation |  | 1SGP3E3 | 1SP3E2 |  |  |  | 1SP3E2 |  |
|  |  |  |  | Evidence |  | 4 | 3 |  |  |  | 3 |  |
|  |  |  |  | Focus |  | 5 | 4 |  |  |  | 4 |  |
|  |  |  |  | Polarity |  | 7 | 7 |  |  |  | 7 |  |
| 535 | 535 | 9507986\_217 | Recently, many studies have focussed on the use of fibric acid derivatives to treat diet-resistant hyperlipidemic patients. |I:\*\*1SGP3E0| |L:\*\*1GP3E1| |T:\*\*1GP3E0| |
|  |  |  |  | Annotation |  | 1SGP3E0 | 1GP3E1 |  |  |  | 1GP3E0 |  |
|  |  |  |  | Evidence |  | 1 | 2 |  |  |  | 1 |  |
|  |  |  |  | Focus |  | 5 | 1 |  |  |  | 1 |  |
|  |  |  |  | Polarity |  | 7 | 7 |  |  |  | 7 |  |
| 536 | 536 | 10768941\_63 | B-cell epitope specificities were assessed by |I:\*\*1SGP0E0| the presence of immunoglobulin G (IgG) serum antibodies to individual peptides. |I:\*\*2MP3E0| |L:\*\*1MP3E3| |T:\*\*1MP3E3| |
|  |  |  |  | Annotation |  | 1SGP0E0 2MP3E0 | 1MP3E3 1MP3E3 |  |  |  | 1MP3E3 1MP3E3 |  |
|  |  |  |  | Evidence |  | 1 1 | 4 4 |  |  |  | 4 4 |  |
|  |  |  |  | Focus |  | 5 2 | 2 2 |  |  |  | 2 2 |  |
|  |  |  |  | Polarity |  | 4 7 | 7 7 |  |  |  | 7 7 |  |
| 537 | 537 | 10454379\_58 | Assessment of the distribution of LY was carried out without knowledge of the experimental history of animals. |I:\*\*1MP3E0| |L:\*\*1GMP3E3| |T:\*\*1MP3E3| |
|  |  |  |  | Annotation |  | 1MP3E0 | 1GMP3E3 |  |  |  | 1MP3E3 |  |
|  |  |  |  | Evidence |  | 1 | 4 |  |  |  | 4 |  |
|  |  |  |  | Focus |  | 2 | 3 |  |  |  | 2 |  |
|  |  |  |  | Polarity |  | 7 | 7 |  |  |  | 7 |  |
| 538 | 538 | 10594025\_47 | One of the alleles described here, rsp5-25, contains two changes in its open reading frame (ORF). |I:\*\*1SP3E0| |L:\*\*1SP3E3| |T:\*\*1MP3E3| |
|  |  |  |  | Annotation |  | 1SP3E0 | 1SP3E3 |  |  |  | 1MP3E3 |  |
|  |  |  |  | Evidence |  | 1 | 4 |  |  |  | 4 |  |
|  |  |  |  | Focus |  | 4 | 4 |  |  |  | 2 |  |
|  |  |  |  | Polarity |  | 7 | 7 |  |  |  | 7 |  |
| 539 | 539 | 9852562\_93 | Similar results were obtained using antisera to Drosophila n-synaptobrevin. |I:\*\*1SP3E0| |L:\*\*1SP2E0| |T:\*\*1SP3E1| |
|  |  |  |  | Annotation |  | 1SP3E0 | 1SP2E0 |  |  |  | 1SP3E1 |  |
|  |  |  |  | Evidence |  | 1 | 1 |  |  |  | 2 |  |
|  |  |  |  | Focus |  | 4 | 4 |  |  |  | 4 |  |
|  |  |  |  | Polarity |  | 7 | 6 |  |  |  | 7 |  |
| 540 | 540 | 12135986\_345 | The final outcome of epithelial skin cell fate then appears to be dictated not only by the levels and location of beta-catenin in a cell, but also the relative levels of other beta-catenin interacting factors in the cell. |I:\*\*1SP2E0| |L:\*\*1SP2E0| |T:\*\*1SP3E1| |
|  |  |  |  | Annotation |  | 1SP2E0 | 1SP2E0 |  |  |  | 1SP3E1 |  |
|  |  |  |  | Evidence |  | 1 | 1 |  |  |  | 2 |  |
|  |  |  |  | Focus |  | 4 | 4 |  |  |  | 4 |  |
|  |  |  |  | Polarity |  | 6 | 6 |  |  |  | 7 |  |
| 541 | 541 | 11390647\_83 | The percentages of cells in the G0/G1, S, and G2/M phases were determined. |I:\*\*1SGP3E0| |L:\*\*1GMP3E3| |T:\*\*1MP3E3| |
|  |  |  |  | Annotation |  | 1SGP3E0 | 1GMP3E3 |  |  |  | 1MP3E3 |  |
|  |  |  |  | Evidence |  | 1 | 4 |  |  |  | 4 |  |
|  |  |  |  | Focus |  | 5 | 3 |  |  |  | 2 |  |
|  |  |  |  | Polarity |  | 7 | 7 |  |  |  | 7 |  |
| 542 | 542 | 12086603\_7 | Biallelic disruption of FANCD2 results in both MMC and IR hypersensitivity. |I:\*\*1SP3E0| |L:\*\*1SP3E0| |T:\*\*1SP3E1| |
|  |  |  |  | Annotation |  | 1SP3E0 | 1SP3E0 |  |  |  | 1SP3E1 |  |
|  |  |  |  | Evidence |  | 1 | 1 |  |  |  | 2 |  |
|  |  |  |  | Focus |  | 4 | 4 |  |  |  | 4 |  |
|  |  |  |  | Polarity |  | 7 | 7 |  |  |  | 7 |  |
| 543 | 543 | 11157218\_87 | N-terminal amino acid sequencing was performed on a Perkin-Elmer ABI 476A automated sequencer using Edman chemistry by the Nucleic Acids/Protein Service Unit, University of British Columbia, Vancouver, British Columbia, Canada. |I:\*\*1MP3E0| |L:\*\*1MP3E3| |T:\*\*1MP3E3| |
|  |  |  |  | Annotation |  | 1MP3E0 | 1MP3E3 |  |  |  | 1MP3E3 |  |
|  |  |  |  | Evidence |  | 1 | 4 |  |  |  | 4 |  |
|  |  |  |  | Focus |  | 2 | 2 |  |  |  | 2 |  |
|  |  |  |  | Polarity |  | 7 | 7 |  |  |  | 7 |  |
| 544 | 544 | 10790410\_43 | Thus, uncertainty obscures aspects of the mutational process once widely accepted, and this impedes understanding of the actual evolutionary consequences of mutation. |I:\*\*1SP3E3| |L:\*\*1GSP0E0| |T:\*\*1SP3E0| |
|  |  |  |  | Annotation |  | 1SP3E3 | 1GSP0E0 |  |  |  | 1SP3E0 |  |
|  |  |  |  | Evidence |  | 4 | 1 |  |  |  | 1 |  |
|  |  |  |  | Focus |  | 4 | 5 |  |  |  | 4 |  |
|  |  |  |  | Polarity |  | 7 | 4 |  |  |  | 7 |  |
| 545 | 545 | 11395449\_57 | Incubation was carried out at 80 degrees C for 15 min in the presence of a solution containing 100 mM KCl, 20 mM Tris-HCl (pH 7), and 20 mM Mg acetate, in a final volume of 15 mul. |I:\*\*1MP3E0| |L:\*\*1MP3E3| |T:\*\*1MP3E3| |
|  |  |  |  | Annotation |  | 1MP3E0 | 1MP3E3 |  |  |  | 1MP3E3 |  |
|  |  |  |  | Evidence |  | 1 | 4 |  |  |  | 4 |  |
|  |  |  |  | Focus |  | 2 | 2 |  |  |  | 2 |  |
|  |  |  |  | Polarity |  | 7 | 7 |  |  |  | 7 |  |
| 546 | 546 | 12527808\_13 | DNR is the first anthracycline developed and has been found to be effective against acute leukemia, whereas DOX was found to be effective also against solid tumors. |I:\*\*1SP3E0| |L:\*\*1SP3E0| |T:\*\*1SP3E1| |
|  |  |  |  | Annotation |  | 1SP3E0 | 1SP3E0 |  |  |  | 1SP3E1 |  |
|  |  |  |  | Evidence |  | 1 | 1 |  |  |  | 2 |  |
|  |  |  |  | Focus |  | 4 | 4 |  |  |  | 4 |  |
|  |  |  |  | Polarity |  | 7 | 7 |  |  |  | 7 |  |
| 547 | 547 | 10455160\_1 | Based on hydropathy analysis ( ), the profile-fed neural network system ( ), and the recently published revised topological models based on scanning cysteine accessibility method ( , ), |I:\*\*1MP3E2| |T:\*\*1MP3E3| the 31 C-terminal amino acids (Table I) of the NCX1 gene are modeled to contain the last transmembrane alpha helix ( ) and an extramembraneous tail. |I:\*\*2SP3E2| |L:\*\*1GMP3E3| |T:\*\*2SP3E1| |
|  |  |  |  | Annotation |  | 1MP3E2 2SP3E2 | 1GMP3E3 1GMP3E3 |  |  |  | 1MP3E3 2SP3E1 |  |
|  |  |  |  | Evidence |  | 3 3 | 4 4 |  |  |  | 4 2 |  |
|  |  |  |  | Focus |  | 2 4 | 3 3 |  |  |  | 2 4 |  |
|  |  |  |  | Polarity |  | 7 7 | 7 7 |  |  |  | 7 7 |  |
| 548 | 548 | 11904424\_30 | We now show that in some melanoma, sarcoma, and fibroblastic cell types that survive in suspension, detachment from the ECM unexpectedly decreases cell death after DNA damage. |I:\*\*1SP3E3-| |L:\*\*1SP3E3-| |T:\*\*1SP3E1-| |
|  |  |  |  | Annotation |  | 1SP3E3- | 1SP3E3- |  |  |  | 1SP3E1- |  |
|  |  |  |  | Evidence |  | 4 | 4 |  |  |  | 2 |  |
|  |  |  |  | Focus |  | 4 | 4 |  |  |  | 4 |  |
|  |  |  |  | Polarity |  | 7 | 7 |  |  |  | 7 |  |
| 549 | 549 | 8980227\_126 | This process was not significantly facilitated by prolongation of the stimulus to 3 min |T:\*\*1MN3E1+| |I:\*\*1SN3E0| |L:\*\*1SN3E0| suggesting that it is not the extent of the initial rise in pCREB that determines its persistence. |T:\*\*1SN1E1+| |I:\*\*1SN1E0| |L:\*\*1SN1E0| |
|  |  |  |  | Annotation |  | 1SN3E0 1SN1E0 | 1SN3E0 1SN1E0 |  |  |  | 1MN3E1+ 1SN1E1+ |  |
|  |  |  |  | Evidence |  | 1 1 | 1 1 |  |  |  | 2 2 |  |
|  |  |  |  | Focus |  | 4 4 | 4 4 |  |  |  | 2 4 |  |
|  |  |  |  | Polarity |  | 1 3 | 1 3 |  |  |  | 1 3 |  |
| 550 | 550 | 10049922\_228 | An additional mechanism is therefore required to entirely explain the increase in sigma2. |I:\*\*1SP3E3| |L:\*\*1GP3E0+| |T:\*\*1SP3E0+| |
|  |  |  |  | Annotation |  | 1SP3E3 | 1GP3E0+ |  |  |  | 1SP3E0+ |  |
|  |  |  |  | Evidence |  | 4 | 1 |  |  |  | 1 |  |
|  |  |  |  | Focus |  | 4 | 1 |  |  |  | 4 |  |
|  |  |  |  | Polarity |  | 7 | 7 |  |  |  | 7 |  |
| 551 | 551 | 10464322\_18 | Our present results suggest an alternative pathway to destabilize the kinase structure for activation through compelling individual Src molecules to interact with each other by S-S bond. |I:\*\*1SP1E3| |L:\*\*1SP1E1| |T:\*\*1SP3E3| |
|  |  |  |  | Annotation |  | 1SP1E3 | 1SP1E1 |  |  |  | 1SP3E3 |  |
|  |  |  |  | Evidence |  | 4 | 2 |  |  |  | 4 |  |
|  |  |  |  | Focus |  | 4 | 4 |  |  |  | 4 |  |
|  |  |  |  | Polarity |  | 5 | 5 |  |  |  | 7 |  |
| 552 | 552 | 9736671\_238 | Thus, in normal rats, a widespread neuronal ensemble may be engaged both during encoding and again during retrieval of the stored information. |I:\*\*1SP3E0| |L:\*\*1SP1E0| |T:\*\*1SP3E1| |
|  |  |  |  | Annotation |  | 1SP3E0 | 1SP1E0 |  |  |  | 1SP3E1 |  |
|  |  |  |  | Evidence |  | 1 | 1 |  |  |  | 2 |  |
|  |  |  |  | Focus |  | 4 | 4 |  |  |  | 4 |  |
|  |  |  |  | Polarity |  | 7 | 5 |  |  |  | 7 |  |
| 553 | 553 | 8752214\_140 | Detection and quantitation of endogenous noggin and BMP proteins in the embryo remains problematic. |I:\*\*1SP3E0| |L:\*\*1GSP0E0| |T:\*\*1GP3E3| |
|  |  |  |  | Annotation |  | 1SP3E0 | 1GSP0E0 |  |  |  | 1GP3E3 |  |
|  |  |  |  | Evidence |  | 1 | 1 |  |  |  | 4 |  |
|  |  |  |  | Focus |  | 4 | 5 |  |  |  | 1 |  |
|  |  |  |  | Polarity |  | 7 | 4 |  |  |  | 7 |  |
| 554 | 554 | 10490606\_43 | Following transfection, cells were harvested at 15 h for Western blot analysis or 24 h for viability assays. |I:\*\*1MP3E0| |L:\*\*1MP3E3| |T:\*\*1MP3E3| |
|  |  |  |  | Annotation |  | 1MP3E0 | 1MP3E3 |  |  |  | 1MP3E3 |  |
|  |  |  |  | Evidence |  | 1 | 4 |  |  |  | 4 |  |
|  |  |  |  | Focus |  | 2 | 2 |  |  |  | 2 |  |
|  |  |  |  | Polarity |  | 7 | 7 |  |  |  | 7 |  |
| 555 | 555 | 11956230\_282 | Data presented are the average of at least four independent experiments. |I:\*\*1MGP3E3| |L:\*\*1GMP3E3| |T:\*\*1MP3E3| |
|  |  |  |  | Annotation |  | 1MGP3E3 | 1GMP3E3 |  |  |  | 1MP3E3 |  |
|  |  |  |  | Evidence |  | 4 | 4 |  |  |  | 4 |  |
|  |  |  |  | Focus |  | 3 | 3 |  |  |  | 2 |  |
|  |  |  |  | Polarity |  | 7 | 7 |  |  |  | 7 |  |
| 556 | 556 | 12150977\_8 | Thus, our data show that sumoylation status of Daxx does not affect its presence in PODs. |I:\*\*1SN3E3| |L:\*\*1SN3E3| |T:\*\*1SP3E1| |
|  |  |  |  | Annotation |  | 1SN3E3 | 1SN3E3 |  |  |  | 1SP3E1 |  |
|  |  |  |  | Evidence |  | 4 | 4 |  |  |  | 2 |  |
|  |  |  |  | Focus |  | 4 | 4 |  |  |  | 4 |  |
|  |  |  |  | Polarity |  | 1 | 1 |  |  |  | 7 |  |
| 557 | 557 | 11894887\_1 | American psychology by the 1920s contained a greater capacity for viewing some homosexual experiences as normal than most current historical literature suggests. |I:\*\*1SP3E0| |L:\*\*1GSP1E1| |T:\*\*1GP3E0| |
|  |  |  |  | Annotation |  | 1SP3E0 | 1GSP1E1 |  |  |  | 1GP3E0 |  |
|  |  |  |  | Evidence |  | 1 | 2 |  |  |  | 1 |  |
|  |  |  |  | Focus |  | 4 | 5 |  |  |  | 1 |  |
|  |  |  |  | Polarity |  | 7 | 5 |  |  |  | 7 |  |
| 558 | 558 | 12839992\_388 | Itoh,K. and Sokol,S.Y. (1999) |L:\*\*1GP3E3| Axis determination by inhibition of Wnt signaling in Xenopus. |L:\*\*2GSP3E3-| |I:\*\*1SGP0E2| |T:\*\*1GP3E3| |
|  |  |  |  | Annotation |  | 1SGP0E2 1SGP0E2 | 1GP3E3 2GSP3E3- |  |  |  | 1GP3E3 1GP3E3 |  |
|  |  |  |  | Evidence |  | 3 3 | 4 4 |  |  |  | 4 4 |  |
|  |  |  |  | Focus |  | 5 5 | 1 5 |  |  |  | 1 1 |  |
|  |  |  |  | Polarity |  | 4 4 | 7 7 |  |  |  | 7 7 |  |
| 559 | 559 | 9508770\_135 | No detectable changes in cell capacitance and fusion pore conductance and thus no fusion pore opening was observed |I:\*\*1SN3E0| when low pH was applied to a HAb2 cell with two bound RBC at 4 degrees C. |L:\*\*1SN3E3| Arrow at pH pulse. |I:\*\*2SGP3E0| |L:\*\*2GP3E3| |T:\*\*1MP3E3| |
|  |  |  |  | Annotation |  | 1SN3E0 2SGP3E0 2SGP3E0 | 1SN3E3 1SN3E3 2GP3E3 |  |  |  | 1MP3E3 1MP3E3 1MP3E3 |  |
|  |  |  |  | Evidence |  | 1 1 1 | 4 4 4 |  |  |  | 4 4 4 |  |
|  |  |  |  | Focus |  | 4 5 5 | 4 4 1 |  |  |  | 2 2 2 |  |
|  |  |  |  | Polarity |  | 1 7 7 | 1 1 7 |  |  |  | 7 7 7 |  |
| 560 | 560 | 9502820\_97 | The beginning was defined as the minimum between 200 msec before the cue onset and the peak for this response, and the end of the responses was defined as the minimum between the peak and 1000 msec after the peak. |I:\*\*1MP3E0| |L:\*\*1MP3E3| |T:\*\*1MP3E3| |
|  |  |  |  | Annotation |  | 1MP3E0 | 1MP3E3 |  |  |  | 1MP3E3 |  |
|  |  |  |  | Evidence |  | 1 | 4 |  |  |  | 4 |  |
|  |  |  |  | Focus |  | 2 | 2 |  |  |  | 2 |  |
|  |  |  |  | Polarity |  | 7 | 7 |  |  |  | 7 |  |
| 561 | 561 | 9390512\_216 | Ideally, observations of a chromosomal abnormality and schizophrenia cosegregating in families would greatly increase the likelihood of a true association. |I:\*\*1SP3E3| |L:\*\*1GP1E0| |T:\*\*1GP3E0| |
|  |  |  |  | Annotation |  | 1SP3E3 | 1GP1E0 |  |  |  | 1GP3E0 |  |
|  |  |  |  | Evidence |  | 4 | 1 |  |  |  | 1 |  |
|  |  |  |  | Focus |  | 4 | 1 |  |  |  | 1 |  |
|  |  |  |  | Polarity |  | 7 | 5 |  |  |  | 7 |  |
| 562 | 562 | 12743106\_326 | Rotarod test Testing began at 15 d of age and animals were studied every 4 d, then once a week after 1 mo of age. |I:\*\*1MP3E0| |L:\*\*1MP3E3| |T:\*\*1MP3E3| |
|  |  |  |  | Annotation |  | 1MP3E0 | 1MP3E3 |  |  |  | 1MP3E3 |  |
|  |  |  |  | Evidence |  | 1 | 4 |  |  |  | 4 |  |
|  |  |  |  | Focus |  | 2 | 2 |  |  |  | 2 |  |
|  |  |  |  | Polarity |  | 7 | 7 |  |  |  | 7 |  |
| 563 | 563 | 11923437\_19 | In the present study, we wished to determine whether transcript levels in more than 70 different gene groups involved in cellular metabolism, which could impact the quality of neuronal communication, were altered in a larger sample of subjects with schizophrenia and whether the effects on these gene groups were interrelated. |I:\*\*1SP0E3| |L:\*\*1GSP0E3| |T:\*\*1SP3E0| |
|  |  |  |  | Annotation |  | 1SP0E3 | 1GSP0E3 |  |  |  | 1SP3E0 |  |
|  |  |  |  | Evidence |  | 4 | 4 |  |  |  | 1 |  |
|  |  |  |  | Focus |  | 4 | 5 |  |  |  | 4 |  |
|  |  |  |  | Polarity |  | 4 | 4 |  |  |  | 7 |  |
| 564 | 564 | 11679314\_118 | The concentration of acrylic acid and 4-pentenoic acid that remained in media was analyzed in a rather different way. |I:\*\*1MGP3E0| |L:\*\*1GMP3E0| |T:\*\*1MP3E3| |
|  |  |  |  | Annotation |  | 1MGP3E0 | 1GMP3E0 |  |  |  | 1MP3E3 |  |
|  |  |  |  | Evidence |  | 1 | 1 |  |  |  | 4 |  |
|  |  |  |  | Focus |  | 3 | 3 |  |  |  | 2 |  |
|  |  |  |  | Polarity |  | 7 | 7 |  |  |  | 7 |  |
| 565 | 565 | 11086983\_22 | Meador-Woodruff et al. 1997 ; Volk et al. 2000 ) or second messenger systems ( Dean et al. 1997 ; Shimon et al. 1998 ; Hudson et al. 1999 ) have been observed. |I:\*\*ERROR| |L:\*\*1SP3E2| |T:\*\*1GP3E3| |
|  |  |  |  | Annotation |  | ERROR | 1SP3E2 |  |  |  | 1GP3E3 |  |
|  |  |  |  | Evidence |  | -1000 | 3 |  |  |  | 4 |  |
|  |  |  |  | Focus |  | -1000 | 4 |  |  |  | 1 |  |
|  |  |  |  | Polarity |  | -1000 | 7 |  |  |  | 7 |  |
| 566 | 566 | 8626772\_32 | Analogous sedimentation velocity experiments (data not shown) of the DnaB hexamer in the presence of the saturating concentration of ADP (5 10 M) show |I:\*\*1MP3E3| that the sedimentation coefficient of the helicase is s = 11.4 plus-or-minus 0.2, a value significantly higher than the sedimentation coefficient of the free enzyme. |I:\*\*2SP3E3| |L:\*\*1MSP3E13+| |T:\*\*1SP3E1| |
|  |  |  |  | Annotation |  | 1MP3E3 2SP3E3 | 1MSP3E13+ 1MSP3E13+ |  |  |  | 1SP3E1 1SP3E1 |  |
|  |  |  |  | Evidence |  | 4 4 | 2 2 |  |  |  | 2 2 |  |
|  |  |  |  | Focus |  | 2 4 | 6 6 |  |  |  | 4 4 |  |
|  |  |  |  | Polarity |  | 7 7 | 7 7 |  |  |  | 7 7 |  |
| 567 | 567 | 11390664\_206 | However, the same mutant still causes significant suppression of deltaMEKK1-induced apoptosis (Fig. 4B). |I:\*\*1SP3E3-| |L:\*\*1SP3E3-| |T:\*\*1SP3E3| |
|  |  |  |  | Annotation |  | 1SP3E3- | 1SP3E3- |  |  |  | 1SP3E3 |  |
|  |  |  |  | Evidence |  | 4 | 4 |  |  |  | 4 |  |
|  |  |  |  | Focus |  | 4 | 4 |  |  |  | 4 |  |
|  |  |  |  | Polarity |  | 7 | 7 |  |  |  | 7 |  |
| 568 | 568 | 9224822\_154 | The VIP radioligand bound with high affinity to both wild-type receptors and all chimeric receptors. |I:\*\*1SP3E0| |L:\*\*1SP3E0| |T:\*\*1SP3E1| |
|  |  |  |  | Annotation |  | 1SP3E0 | 1SP3E0 |  |  |  | 1SP3E1 |  |
|  |  |  |  | Evidence |  | 1 | 1 |  |  |  | 2 |  |
|  |  |  |  | Focus |  | 4 | 4 |  |  |  | 4 |  |
|  |  |  |  | Polarity |  | 7 | 7 |  |  |  | 7 |  |
| 569 | 569 | 12135806\_5 | This article reviews the features of FAOD, critically evaluates methods of investigation including metabolite analyses in body fluids, in vitro oxidation rates and acylcarnitine profiling studies, enzymatic and mutational tests, and discusses genotype-phenotype correlation, treatment and monitoring options. |I:\*\*1SGP3E3| |L:\*\*1GP3E3| |T:\*\*1GP3E1| |
|  |  |  |  | Annotation |  | 1SGP3E3 | 1GP3E3 |  |  |  | 1GP3E1 |  |
|  |  |  |  | Evidence |  | 4 | 4 |  |  |  | 2 |  |
|  |  |  |  | Focus |  | 5 | 1 |  |  |  | 1 |  |
|  |  |  |  | Polarity |  | 7 | 7 |  |  |  | 7 |  |
| 570 | 570 | 11890447\_9 | RESULTS: The overall stone-free rate for stones <10 mm was 62% at 1 month and 76% at 3 months. |I:\*\*1SP3E3| |L:\*\*1SP3E3| |T:\*\*1SP3E3| |
|  |  |  |  | Annotation |  | 1SP3E3 | 1SP3E3 |  |  |  | 1SP3E3 |  |
|  |  |  |  | Evidence |  | 4 | 4 |  |  |  | 4 |  |
|  |  |  |  | Focus |  | 4 | 4 |  |  |  | 4 |  |
|  |  |  |  | Polarity |  | 7 | 7 |  |  |  | 7 |  |
| 571 | 571 | 10535740\_138 | To do this, we imaged carboxyfluorescein-filled sensory cells, treated them according to the synapse-specific capture or cell-wide protocol, and reimaged them at 72 hr. |I:\*\*1MP3E3| |L:\*\*1MP3E3| |T:\*\*1MP3E3| |
|  |  |  |  | Annotation |  | 1MP3E3 | 1MP3E3 |  |  |  | 1MP3E3 |  |
|  |  |  |  | Evidence |  | 4 | 4 |  |  |  | 4 |  |
|  |  |  |  | Focus |  | 2 | 2 |  |  |  | 2 |  |
|  |  |  |  | Polarity |  | 7 | 7 |  |  |  | 7 |  |
| 572 | 572 | 11832230\_154 | A large F,Ca2 in the spine and a small or no F,Ca2 in the dendrite are evidence of a diffusion barrier posed by the spine neck; |I:\*\*1SP3E0| |L:\*\*1SP3E3| without such a barrier, |I:\*\*2SN3E0| Ca2+ would equilibrate quickly across the spine neck and hence trial-to-trial variability should be equal in both comparetments. |I:\*\*3SP3E0| |L:\*\*2GSP1E3| |T:\*\*1GP3E0| |
|  |  |  |  | Annotation |  | 1SP3E0 2SN3E0 3SP3E0 | 1SP3E3 2GSP1E3 2GSP1E3 |  |  |  | 1GP3E0 1GP3E0 1GP3E0 |  |
|  |  |  |  | Evidence |  | 1 1 1 | 4 4 4 |  |  |  | 1 1 1 |  |
|  |  |  |  | Focus |  | 4 4 4 | 4 5 5 |  |  |  | 1 1 1 |  |
|  |  |  |  | Polarity |  | 7 1 7 | 7 5 5 |  |  |  | 7 7 7 |  |
| 573 | 573 | 10049922\_139 | The method was checked using simulated data with known constant Ne values and census sizes corresponding to those observed in our experiments (results not shown). |I:\*\*1MP3E3| |L:\*\*1SP3E1| |T:\*\*1MP3E3| |
|  |  |  |  | Annotation |  | 1MP3E3 | 1SP3E1 |  |  |  | 1MP3E3 |  |
|  |  |  |  | Evidence |  | 4 | 2 |  |  |  | 4 |  |
|  |  |  |  | Focus |  | 2 | 4 |  |  |  | 2 |  |
|  |  |  |  | Polarity |  | 7 | 7 |  |  |  | 7 |  |
| 574 | 574 | 12086850\_188 | No contamination of cytoplasmic fractions by nuclear proteins was detected (see Figure 6A and data not shown). |I:\*\*1SN3E3| |L:\*\*1SN3E3| |T:\*\*1SP3E3| |
|  |  |  |  | Annotation |  | 1SN3E3 | 1SN3E3 |  |  |  | 1SP3E3 |  |
|  |  |  |  | Evidence |  | 4 | 4 |  |  |  | 4 |  |
|  |  |  |  | Focus |  | 4 | 4 |  |  |  | 4 |  |
|  |  |  |  | Polarity |  | 1 | 1 |  |  |  | 7 |  |
| 575 | 575 | 9336330\_5 | Continuous infusion of ABT-089 to aged rats enhanced spatial learning in a standard Morris water maze, as indexed by spatial bias exhibited during a probe trial conducted after 4 days of training, |I:\*\*1SP3E0| |L:\*\*1SP3E3| but not when they were subsequently trained in a two-platform spatial discrimination water maze. |I:\*\*2SN3E0| |L:\*\*2SN3E3| |T:\*\*1MP3E3| |
|  |  |  |  | Annotation |  | 1SP3E0 2SN3E0 | 1SP3E3 2SN3E3 |  |  |  | 1MP3E3 1MP3E3 |  |
|  |  |  |  | Evidence |  | 1 1 | 4 4 |  |  |  | 4 4 |  |
|  |  |  |  | Focus |  | 4 4 | 4 4 |  |  |  | 2 2 |  |
|  |  |  |  | Polarity |  | 7 1 | 7 1 |  |  |  | 7 7 |  |
| 576 | 576 | 12194849\_276 | M.M.T. acknowledges support from grants from the Ministry of Education, Science, Sports, and Culture, and from the Organization for Pharmaceutical Safety and Research, Japan. |I:\*\*1GP3E3| |L:\*\*1GP3E3| |T:\*\*1SP3E3| |
|  |  |  |  | Annotation |  | 1GP3E3 | 1GP3E3 |  |  |  | 1SP3E3 |  |
|  |  |  |  | Evidence |  | 4 | 4 |  |  |  | 4 |  |
|  |  |  |  | Focus |  | 1 | 1 |  |  |  | 4 |  |
|  |  |  |  | Polarity |  | 7 | 7 |  |  |  | 7 |  |
| 577 | 577 | 9389652\_185 | One of these Galpha subunits, Gpa3, is required for both virulence and mating (Regenfelder et al. 1996 ). |I:\*\*1SP3E2| |L:\*\*1SP3E2| |T:\*\*1SP3E2| |
|  |  |  |  | Annotation |  | 1SP3E2 | 1SP3E2 |  |  |  | 1SP3E2 |  |
|  |  |  |  | Evidence |  | 3 | 3 |  |  |  | 3 |  |
|  |  |  |  | Focus |  | 4 | 4 |  |  |  | 4 |  |
|  |  |  |  | Polarity |  | 7 | 7 |  |  |  | 7 |  |
| 578 | 578 | 11805099\_7 | We show that paxillin binds directly to syndesmos, |I:\*\*1SP3E3| a cytoplasmic protein that binds specifically to the cytoplasmic domain of syndecan-4 ( ). |I:\*\*2SP3E2| |L:\*\*1SP3E3| |T:\*\*1SP3E2| |
|  |  |  |  | Annotation |  | 1SP3E3 2SP3E2 | 1SP3E3 1SP3E3 |  |  |  | 1SP3E2 1SP3E2 |  |
|  |  |  |  | Evidence |  | 4 3 | 4 4 |  |  |  | 3 3 |  |
|  |  |  |  | Focus |  | 4 4 | 4 4 |  |  |  | 4 4 |  |
|  |  |  |  | Polarity |  | 7 7 | 7 7 |  |  |  | 7 7 |  |
| 579 | 579 | 10588958\_261 | Although lipoprotein lipase only hydrolyzes the 1- and 3-positions of TAG ( 47) |I:\*\*1SP3E2| hydrolysis of fatty acids from TAG in plasma appears to be complete, |I:\*\*2SP2E2| |L:\*\*1SP3E2| so that no 2-monoacylglycerol is taken up ( 48). |I:\*\*3SN3E2| |L:\*\*2SN3E2| |T:\*\*1SP3E2| |
|  |  |  |  | Annotation |  | 1SP3E2 2SP2E2 3SN3E2 | 1SP3E2 1SP3E2 2SN3E2 |  |  |  | 1SP3E2 1SP3E2 1SP3E2 |  |
|  |  |  |  | Evidence |  | 3 3 3 | 3 3 3 |  |  |  | 3 3 3 |  |
|  |  |  |  | Focus |  | 4 4 4 | 4 4 4 |  |  |  | 4 4 4 |  |
|  |  |  |  | Polarity |  | 7 6 1 | 7 7 1 |  |  |  | 7 7 7 |  |
| 580 | 580 | 10562277\_363 | This finding suggests a close functional interaction of the Sec34 and Sec35 proteins. |I:\*\*1SP1E0| |L:\*\*1SP1E3| |T:\*\*1SP1E1| |
|  |  |  |  | Annotation |  | 1SP1E0 | 1SP1E3 |  |  |  | 1SP1E1 |  |
|  |  |  |  | Evidence |  | 1 | 4 |  |  |  | 2 |  |
|  |  |  |  | Focus |  | 4 | 4 |  |  |  | 4 |  |
|  |  |  |  | Polarity |  | 5 | 5 |  |  |  | 5 |  |
| 581 | 581 | 11416145\_292 | Two previous studies have detailed the gene expression changes during the S. cerevisiae cell cycle ( 1, 23). |I:\*\*1SGP3E2| |L:\*\*1SP3E2| |T:\*\*1SP3E2| |
|  |  |  |  | Annotation |  | 1SGP3E2 | 1SP3E2 |  |  |  | 1SP3E2 |  |
|  |  |  |  | Evidence |  | 3 | 3 |  |  |  | 3 |  |
|  |  |  |  | Focus |  | 5 | 4 |  |  |  | 4 |  |
|  |  |  |  | Polarity |  | 7 | 7 |  |  |  | 7 |  |
| 582 | 582 | 11705923\_69 | The samples and a positive serum reference (a serum pool from Swedish volunteers vaccinated orally with the whole-cell-CTB cholera vaccine) were added in duplicates and serially diluted. |I:\*\*1MP3E0| |L:\*\*1MP3E3| |T:\*\*1MP3E3| |
|  |  |  |  | Annotation |  | 1MP3E0 | 1MP3E3 |  |  |  | 1MP3E3 |  |
|  |  |  |  | Evidence |  | 1 | 4 |  |  |  | 4 |  |
|  |  |  |  | Focus |  | 2 | 2 |  |  |  | 2 |  |
|  |  |  |  | Polarity |  | 7 | 7 |  |  |  | 7 |  |
| 583 | 583 | 12150331\_3 | Twenty-three children, whose mothers were hyperthyroid during pregnancy and treated with MMI 5-20 mg were studied from age 3-11 yr. |I:\*\*1MP3E0| |L:\*\*1MP3E3| |T:\*\*1MP3E3| |
|  |  |  |  | Annotation |  | 1MP3E0 | 1MP3E3 |  |  |  | 1MP3E3 |  |
|  |  |  |  | Evidence |  | 1 | 4 |  |  |  | 4 |  |
|  |  |  |  | Focus |  | 2 | 2 |  |  |  | 2 |  |
|  |  |  |  | Polarity |  | 7 | 7 |  |  |  | 7 |  |
| 584 | 584 | 11973317\_86 | For the TFL1 promoter, Tajima's D was estimated |I:\*\*1SGP0E0| using nucleotide and indel polymorphism data, with the latter coded as single characters. |I:\*\*2MP3E0| |L:\*\*1SP1E0| |T:\*\*1MP3E3| |
|  |  |  |  | Annotation |  | 1SGP0E0 2MP3E0 | 1SP1E0 1SP1E0 |  |  |  | 1MP3E3 1MP3E3 |  |
|  |  |  |  | Evidence |  | 1 1 | 1 1 |  |  |  | 4 4 |  |
|  |  |  |  | Focus |  | 5 2 | 4 4 |  |  |  | 2 2 |  |
|  |  |  |  | Polarity |  | 4 7 | 5 5 |  |  |  | 7 7 |  |
| 585 | 585 | 12551965\_26 | This latter study was limited, however, by small sample sizes (4 young dogs, 7 middle-aged dogs, and 4 old dogs), constraints on length of testing (50 trials), and the absence of a size reversal task to assess possible differences in inhibitory control between young and old beagle dogs. |I:\*\*1MSP3E0| |L:\*\*1MP3E1| |T:\*\*1SN3E0| |
|  |  |  |  | Annotation |  | 1MSP3E0 | 1MP3E1 |  |  |  | 1SN3E0 |  |
|  |  |  |  | Evidence |  | 1 | 2 |  |  |  | 1 |  |
|  |  |  |  | Focus |  | 6 | 2 |  |  |  | 4 |  |
|  |  |  |  | Polarity |  | 7 | 7 |  |  |  | 1 |  |
| 586 | 586 | 11144365\_12 | This is the case for NMDA receptors, in which Ca2+ induces binding of calmodulin to the channel ( Ehlers et al. 1996 ), |L:\*\*1SP3E2+| causing its rapid inactivation ( Legendre et al. 1993 ; Medina et al. 1994, Medina et al. 1996 ; Kyrozis et al. 1996 ). |L:\*\*2SP3E2-| |I:\*\*1SP3E2| |T:\*\*1SP3E2-| |
|  |  |  |  | Annotation |  | 1SP3E2 1SP3E2 | 1SP3E2+ 2SP3E2- |  |  |  | 1SP3E2- 1SP3E2- |  |
|  |  |  |  | Evidence |  | 3 3 | 3 3 |  |  |  | 3 3 |  |
|  |  |  |  | Focus |  | 4 4 | 4 4 |  |  |  | 4 4 |  |
|  |  |  |  | Polarity |  | 7 7 | 7 7 |  |  |  | 7 7 |  |
| 587 | 587 | 7493953\_35 | ( ) The abbreviations used are: IL, interleukin; IL-5alphaR, IL-5 receptor alpha-subunit; IL-5betaR, IL-5 receptor beta-subunit; alphaRED, extracellular domain of the IL-5 receptor alpha-subunit; PCR, polymerase chain reaction; FLAG(TM), N-Asp-Tyr-Lys-Asp-Asp-Asp-Asp-Lys- C octapeptide; BS , bis(sulfosuccinimidyl) suberate; PAGE, polyacrylamide gel electrophoresis. |I:\*\*1SGP3E3| |L:\*\*1GSP3E3| |T:\*\*1MP3E3| |
|  |  |  |  | Annotation |  | 1SGP3E3 | 1GSP3E3 |  |  |  | 1MP3E3 |  |
|  |  |  |  | Evidence |  | 4 | 4 |  |  |  | 4 |  |
|  |  |  |  | Focus |  | 5 | 5 |  |  |  | 2 |  |
|  |  |  |  | Polarity |  | 7 | 7 |  |  |  | 7 |  |
| 588 | 588 | 10347042\_144 | Thus, the percentage of chitin-degrading bacteria in the original water sample can be estimated |I:\*\*1SGP0E0| by dividing the observed frequency of MUF-diNAG positive clones by the expected frequency. |I:\*\*2MP3E0| |L:\*\*1GMP3E0| |T:\*\*1MP3E0| |
|  |  |  |  | Annotation |  | 1SGP0E0 2MP3E0 | 1GMP3E0 1GMP3E0 |  |  |  | 1MP3E0 1MP3E0 |  |
|  |  |  |  | Evidence |  | 1 1 | 1 1 |  |  |  | 1 1 |  |
|  |  |  |  | Focus |  | 5 2 | 3 3 |  |  |  | 2 2 |  |
|  |  |  |  | Polarity |  | 4 7 | 7 7 |  |  |  | 7 7 |  |
| 589 | 589 | 9614213\_125 | Fig. 2A shows the double-reciprocal plot of 1/ k versus 1/[puromycin] for anisomycin concentrations ranging from 1 x 10 6 to 20 x 10 6M. |I:\*\*1SGP3E3| |L:\*\*1GSP3E3| |T:\*\*1MP3E3| |
|  |  |  |  | Annotation |  | 1SGP3E3 | 1GSP3E3 |  |  |  | 1MP3E3 |  |
|  |  |  |  | Evidence |  | 4 | 4 |  |  |  | 4 |  |
|  |  |  |  | Focus |  | 5 | 5 |  |  |  | 2 |  |
|  |  |  |  | Polarity |  | 7 | 7 |  |  |  | 7 |  |
| 590 | 590 | 10400654\_70 | View larger version (15K): [in this window] [in a new window] |I:\*\*ERROR| |L:\*\*ERROR| |T:\*\*ERROR| Fig. 4. Accumulation of dexamethasone in W7TB, MS23, and MSPP-1 cells. |I:\*\*1SGP3E3| |L:\*\*1GSP3E3| |T:\*\*1GP3E3| |
|  |  |  |  | Annotation |  | ERROR 1SGP3E3 | ERROR 1GSP3E3 |  |  |  | ERROR 1GP3E3 |  |
|  |  |  |  | Evidence |  | -1000 4 | -1000 4 |  |  |  | -1000 4 |  |
|  |  |  |  | Focus |  | -1000 5 | -1000 5 |  |  |  | -1000 1 |  |
|  |  |  |  | Polarity |  | -1000 7 | -1000 7 |  |  |  | -1000 7 |  |
| 591 | 591 | 10978289\_247 | The mRNA can be detected in the germ cells throughout their migration through the midgut and into the gonadal mesoderm (data not shown) and is present in the embryonic gonad of stage 15 embryos ( Fig 6G). |I:\*\*1SP3E3| |L:\*\*1SP3E13| |T:\*\*1SP3E3| |
|  |  |  |  | Annotation |  | 1SP3E3 | 1SP3E13 |  |  |  | 1SP3E3 |  |
|  |  |  |  | Evidence |  | 4 | 2 |  |  |  | 4 |  |
|  |  |  |  | Focus |  | 4 | 4 |  |  |  | 4 |  |
|  |  |  |  | Polarity |  | 7 | 7 |  |  |  | 7 |  |
| 592 | 592 | 10488083\_29 | These antibodies yielded an excellent signal with NRP (Fig. 2). |I:\*\*1MSP3E3| |L:\*\*1SP3E3| |T:\*\*1SP3E3| |
|  |  |  |  | Annotation |  | 1MSP3E3 | 1SP3E3 |  |  |  | 1SP3E3 |  |
|  |  |  |  | Evidence |  | 4 | 4 |  |  |  | 4 |  |
|  |  |  |  | Focus |  | 6 | 4 |  |  |  | 4 |  |
|  |  |  |  | Polarity |  | 7 | 7 |  |  |  | 7 |  |
| 593 | 593 | 9797283\_40 | The clinical strains Enterobacter cloacae RYC70770 and Escherichia hermannii RYC78330 were provided by the Hospital Ramon y Cajal, Madrid, Spain. |I:\*\*1MGP3E0| |L:\*\*1GMP3E3| |T:\*\*1GP3E3| |
|  |  |  |  | Annotation |  | 1MGP3E0 | 1GMP3E3 |  |  |  | 1GP3E3 |  |
|  |  |  |  | Evidence |  | 1 | 4 |  |  |  | 4 |  |
|  |  |  |  | Focus |  | 3 | 3 |  |  |  | 1 |  |
|  |  |  |  | Polarity |  | 7 | 7 |  |  |  | 7 |  |
| 594 | 594 | 11861563\_9 | This novel numb allele, as well as previously described ones, was shown to affect the fly's rhythm of locomotor activity. |I:\*\*1SP3E0| |L:\*\*1SP3E13| |T:\*\*1SP3E1| |
|  |  |  |  | Annotation |  | 1SP3E0 | 1SP3E13 |  |  |  | 1SP3E1 |  |
|  |  |  |  | Evidence |  | 1 | 2 |  |  |  | 2 |  |
|  |  |  |  | Focus |  | 4 | 4 |  |  |  | 4 |  |
|  |  |  |  | Polarity |  | 7 | 7 |  |  |  | 7 |  |
| 595 | 595 | 7499415\_48 | Thus the decrease of the biosynthesis rate of apoB-100 could not explain the almost complete absence of apoB-100 lipoproteins in the secretory pathway. |I:\*\*1SN3E0-| |L:\*\*1GSN3E0-| |T:\*\*1SN3E1-| |
|  |  |  |  | Annotation |  | 1SN3E0- | 1GSN3E0- |  |  |  | 1SN3E1- |  |
|  |  |  |  | Evidence |  | 1 | 1 |  |  |  | 2 |  |
|  |  |  |  | Focus |  | 4 | 5 |  |  |  | 4 |  |
|  |  |  |  | Polarity |  | 1 | 1 |  |  |  | 1 |  |
| 596 | 596 | 12620407\_325 | Binding reactions contained 200 cpm of 32P-labeled probe, 75 ng of wild-type p53, or 10 ng of p53 C ( 363), in the presence of 50 or 100 ng of GST-PTEN, GST-PTEN-C, GST-PTEN-CT, GST, or GST-c-Abl, in a total volume of 12.5 l of reaction buffer (60 mM KCl, 12% glycerol, 5 mM MgCl2, 1 mM EDTA, 1 g BSA, 0.1 g poly [d(GC)]). |I:\*\*1MP3E0| |L:\*\*1MP3E3| |T:\*\*1MP3E3| |
|  |  |  |  | Annotation |  | 1MP3E0 | 1MP3E3 |  |  |  | 1MP3E3 |  |
|  |  |  |  | Evidence |  | 1 | 4 |  |  |  | 4 |  |
|  |  |  |  | Focus |  | 2 | 2 |  |  |  | 2 |  |
|  |  |  |  | Polarity |  | 7 | 7 |  |  |  | 7 |  |
| 597 | 597 | 11927033\_3 | This report is the first of enzootic A. cantonensis infection in Jamaica, |I:\*\*1SGP3E0| |L:\*\*1GP3E3| providing evidence that this parasite is likely to cause human cases of eosinophilic meningitis. |I:\*\*2SP2E0| |L:\*\*2SP1E3| |T:\*\*1SP3E1| |
|  |  |  |  | Annotation |  | 1SGP3E0 2SP2E0 | 1GP3E3 2SP1E3 |  |  |  | 1SP3E1 1SP3E1 |  |
|  |  |  |  | Evidence |  | 1 1 | 4 4 |  |  |  | 2 2 |  |
|  |  |  |  | Focus |  | 5 4 | 1 4 |  |  |  | 4 4 |  |
|  |  |  |  | Polarity |  | 7 6 | 7 5 |  |  |  | 7 7 |  |
| 598 | 598 | 9671487\_130 | These sequences are the G10 stretch between positions 239 and 230 and two GC boxes consisting of the G10 stretch with one internal C residue insertion between positions 105 and 95 and between 54 and 44 (Fig. 1A). |I:\*\*1SP3E3| |L:\*\*1SP3E3| |T:\*\*1SP3E3| |
|  |  |  |  | Annotation |  | 1SP3E3 | 1SP3E3 |  |  |  | 1SP3E3 |  |
|  |  |  |  | Evidence |  | 4 | 4 |  |  |  | 4 |  |
|  |  |  |  | Focus |  | 4 | 4 |  |  |  | 4 |  |
|  |  |  |  | Polarity |  | 7 | 7 |  |  |  | 7 |  |
| 599 | 599 | 9442058\_89 | We did not observe any differences in HeLa cell morphology or growth rate of the CCT.12 cells grown in the presence or absence of doxycycline. |I:\*\*1SN3E3| |L:\*\*1SN3E3| |T:\*\*1SN3E3| |
|  |  |  |  | Annotation |  | 1SN3E3 | 1SN3E3 |  |  |  | 1SN3E3 |  |
|  |  |  |  | Evidence |  | 4 | 4 |  |  |  | 4 |  |
|  |  |  |  | Focus |  | 4 | 4 |  |  |  | 4 |  |
|  |  |  |  | Polarity |  | 1 | 1 |  |  |  | 1 |  |
| 600 | 600 | 11021528\_19 | CTLA-4 engagement can transduce an off signal, disengaging T cells from further activation and proliferation ( Walunas et al. 1994 ; Krummel and Allison 1995 ; Saito 1998 ). |I:\*\*1SP3E2| |L:\*\*1SP3E2-| |T:\*\*1SP3E2| |
|  |  |  |  | Annotation |  | 1SP3E2 | 1SP3E2- |  |  |  | 1SP3E2 |  |
|  |  |  |  | Evidence |  | 3 | 3 |  |  |  | 3 |  |
|  |  |  |  | Focus |  | 4 | 4 |  |  |  | 4 |  |
|  |  |  |  | Polarity |  | 7 | 7 |  |  |  | 7 |  |
| 601 | 601 | 11996672\_3 | Individual, structurally defined oligosaccharides derived from S. mansoni egg glycolipids were tested for their binding to this monoclonal antibody |I:\*\*1SP0E0| by immunoaffinity chromatography. |I:\*\*2MP3E0| |L:\*\*1MP3E3| |T:\*\*1MN3E3| |
|  |  |  |  | Annotation |  | 1SP0E0 2MP3E0 | 1MP3E3 1MP3E3 |  |  |  | 1MN3E3 1MN3E3 |  |
|  |  |  |  | Evidence |  | 1 1 | 4 4 |  |  |  | 4 4 |  |
|  |  |  |  | Focus |  | 4 2 | 2 2 |  |  |  | 2 2 |  |
|  |  |  |  | Polarity |  | 4 7 | 7 7 |  |  |  | 1 1 |  |
| 602 | 602 | 12875988\_171 | Synovial sarcomas were characterized by genes expressed in early developmental pathways involving WNT and notch signaling, including TLE1, FZD1, WNT5A, and JAG2. |I:\*\*1SP3E0| |L:\*\*1SP3E0| |T:\*\*1MN3E3| |
|  |  |  |  | Annotation |  | 1SP3E0 | 1SP3E0 |  |  |  | 1MN3E3 |  |
|  |  |  |  | Evidence |  | 1 | 1 |  |  |  | 4 |  |
|  |  |  |  | Focus |  | 4 | 4 |  |  |  | 2 |  |
|  |  |  |  | Polarity |  | 7 | 7 |  |  |  | 1 |  |
| 603 | 603 | 9388247\_48 | [View Larger Version of this Image (83K GIF file)] |I:\*\*ERROR| |L:\*\*1GP3E3| |T:\*\*1GP3E3| |
|  |  |  |  | Annotation |  | ERROR | 1GP3E3 |  |  |  | 1GP3E3 |  |
|  |  |  |  | Evidence |  | -1000 | 4 |  |  |  | 4 |  |
|  |  |  |  | Focus |  | -1000 | 1 |  |  |  | 1 |  |
|  |  |  |  | Polarity |  | -1000 | 7 |  |  |  | 7 |  |
| 604 | 604 | 9473028\_203 | Previously, we have found that recovery from transient acidification is dependent upon K+ transport, |I:\*\*1SP3E3| |L:\*\*1SP3E2| |T:\*\*1SP3E1| though no net K+ accumulation was detected ( 5). |I:\*\*2SN3E3| |L:\*\*2SN3E2| |T:\*\*2SN3E2| |
|  |  |  |  | Annotation |  | 1SP3E3 2SN3E3 | 1SP3E2 2SN3E2 |  |  |  | 1SP3E1 2SN3E2 |  |
|  |  |  |  | Evidence |  | 4 4 | 3 3 |  |  |  | 2 3 |  |
|  |  |  |  | Focus |  | 4 4 | 4 4 |  |  |  | 4 4 |  |
|  |  |  |  | Polarity |  | 7 1 | 7 1 |  |  |  | 7 1 |  |
| 605 | 605 | 11869678\_189 | However, it now seems clear that both arms can have an important function in Ab/IC-induced inflammation (Kohl and Gessner, 1999 ; Ravetch and Bolland, 2001 ), their relative contributions varying with the particular tissue(s) involved and the genetic background. |I:\*\*1SP3E2| |L:\*\*1SP1E2| |T:\*\*1SP3E3| |
|  |  |  |  | Annotation |  | 1SP3E2 | 1SP1E2 |  |  |  | 1SP3E3 |  |
|  |  |  |  | Evidence |  | 3 | 3 |  |  |  | 4 |  |
|  |  |  |  | Focus |  | 4 | 4 |  |  |  | 4 |  |
|  |  |  |  | Polarity |  | 7 | 5 |  |  |  | 7 |  |
| 606 | 606 | 11754812\_84 | Similar results were obtained in the A20 B cell line transfected with CD1-WT (data not shown). |I:\*\*1SGP3E3| |L:\*\*1SP3E1| |T:\*\*1SP3E1| |
|  |  |  |  | Annotation |  | 1SGP3E3 | 1SP3E1 |  |  |  | 1SP3E1 |  |
|  |  |  |  | Evidence |  | 4 | 2 |  |  |  | 2 |  |
|  |  |  |  | Focus |  | 5 | 4 |  |  |  | 4 |  |
|  |  |  |  | Polarity |  | 7 | 7 |  |  |  | 7 |  |
| 607 | 607 | 10197540\_5 | Our results provide direct evidence of the involvement of primary auditory areas in auditory verbal hallucinations and establish novel constraints for psychopathological models. |I:\*\*1SP3E3| |L:\*\*1GSP3E3| |T:\*\*1SP3E3| |
|  |  |  |  | Annotation |  | 1SP3E3 | 1GSP3E3 |  |  |  | 1SP3E3 |  |
|  |  |  |  | Evidence |  | 4 | 4 |  |  |  | 4 |  |
|  |  |  |  | Focus |  | 4 | 5 |  |  |  | 4 |  |
|  |  |  |  | Polarity |  | 7 | 7 |  |  |  | 7 |  |
| 608 | 608 | 9182756\_174 | There is one monomer in the asymmetric unit that corresponds to a solvent content of approximately 66%. |I:\*\*1SP3E0| |L:\*\*1SP3E0| |T:\*\*1MP3E3| |
|  |  |  |  | Annotation |  | 1SP3E0 | 1SP3E0 |  |  |  | 1MP3E3 |  |
|  |  |  |  | Evidence |  | 1 | 1 |  |  |  | 4 |  |
|  |  |  |  | Focus |  | 4 | 4 |  |  |  | 2 |  |
|  |  |  |  | Polarity |  | 7 | 7 |  |  |  | 7 |  |
| 609 | 609 | 9390512\_176 | Wei et al. 1995 ; Karayiorgou et al. 1996 ; Karayiorgou et al. 1997a ; Li et al. 1996 ; Shaikh et al. 1996 ), models of serotonergic dysfunction ( Arranz et al. 1995 ; Inayama et al. 1996 Williams ; et al. 1997 ), viral/autoimmune theories ( Nimgaonkar et al. 1993b ; Nimgaonkar et al. 1995 ; Wright et al. 1996 ), and neurodevelopmental disturbances ( Nanko et al. 1994 ; Dawson et al. 1995 ). |I:\*\*ERROR| |L:\*\*1GSP3E2| |T:\*\*1GP3E3| |
|  |  |  |  | Annotation |  | ERROR | 1GSP3E2 |  |  |  | 1GP3E3 |  |
|  |  |  |  | Evidence |  | -1000 | 3 |  |  |  | 4 |  |
|  |  |  |  | Focus |  | -1000 | 5 |  |  |  | 1 |  |
|  |  |  |  | Polarity |  | -1000 | 7 |  |  |  | 7 |  |
| 610 | 610 | 9316830\_55 | Only hair specimens that could be clearly distinguished as black and brown or as blond were included. |I:\*\*1MP3E0| |L:\*\*1MP3E3| |T:\*\*1MP3E3| |
|  |  |  |  | Annotation |  | 1MP3E0 | 1MP3E3 |  |  |  | 1MP3E3 |  |
|  |  |  |  | Evidence |  | 1 | 4 |  |  |  | 4 |  |
|  |  |  |  | Focus |  | 2 | 2 |  |  |  | 2 |  |
|  |  |  |  | Polarity |  | 7 | 7 |  |  |  | 7 |  |
| 611 | 611 | 12509439\_3 | To test the postulated relationship between PtdSer externalization and its biosynthesis, |I:\*\*1SGP0E3| we studied the involvement of PLSCR isoforms responsible for active translocation of PtdSer to the cell surface in regulating PtdSer biosynthetic pathways. |I:\*\*2SGP3E3| |L:\*\*1GSP3E3| |T:\*\*1GP3E3| |
|  |  |  |  | Annotation |  | 1SGP0E3 2SGP3E3 | 1GSP3E3 1GSP3E3 |  |  |  | 1GP3E3 1GP3E3 |  |
|  |  |  |  | Evidence |  | 4 4 | 4 4 |  |  |  | 4 4 |  |
|  |  |  |  | Focus |  | 5 5 | 5 5 |  |  |  | 1 1 |  |
|  |  |  |  | Polarity |  | 4 7 | 7 7 |  |  |  | 7 7 |  |
| 612 | 612 | 11086983\_40 | Second, to assess interexperimental variability, |I:\*\*1SGP0E0| pair 685c/622s was hybridized to three different UniGEM-V microarrays. |I:\*\*2MP3E0| |L:\*\*1MP3E3| |T:\*\*1MP3E3| |
|  |  |  |  | Annotation |  | 1SGP0E0 2MP3E0 | 1MP3E3 1MP3E3 |  |  |  | 1MP3E3 1MP3E3 |  |
|  |  |  |  | Evidence |  | 1 1 | 4 4 |  |  |  | 4 4 |  |
|  |  |  |  | Focus |  | 5 2 | 2 2 |  |  |  | 2 2 |  |
|  |  |  |  | Polarity |  | 4 7 | 7 7 |  |  |  | 7 7 |  |
| 613 | 613 | 9813103\_228 | Indeed, in LMU cells, paxillin shows reduced levels of phosphotyrosine when compared with LP and LWT cells (Fig. 8 B, top). |I:\*\*1SP3E3-| |L:\*\*1SP3E3-| |T:\*\*1SP3E2-| |
|  |  |  |  | Annotation |  | 1SP3E3- | 1SP3E3- |  |  |  | 1SP3E2- |  |
|  |  |  |  | Evidence |  | 4 | 4 |  |  |  | 3 |  |
|  |  |  |  | Focus |  | 4 | 4 |  |  |  | 4 |  |
|  |  |  |  | Polarity |  | 7 | 7 |  |  |  | 7 |  |
| 614 | 614 | 11030628\_28 | Moreover, we provide evidence that Ser-46 of p53 is phosphorylated in response to DNA damage in vivo, and it plays a pivotal role for apoptotic signaling by p53 through regulating the transcriptional activation of an apoptosis-inducing gene, p53AIP1. |I:\*\*1SP3E3| |L:\*\*1SP3E3| |T:\*\*1SP3E1| |
|  |  |  |  | Annotation |  | 1SP3E3 | 1SP3E3 |  |  |  | 1SP3E1 |  |
|  |  |  |  | Evidence |  | 4 | 4 |  |  |  | 2 |  |
|  |  |  |  | Focus |  | 4 | 4 |  |  |  | 4 |  |
|  |  |  |  | Polarity |  | 7 | 7 |  |  |  | 7 |  |
| 615 | 615 | 9655899\_91 | The 32P-labeled probes were added to the prehybridization solution in a concentration of 0.5 to 1 x 10^6^ dpm/ml. |I:\*\*1MP3E0| Hybridization of the membrane was performed at 42 degrees C for 16 to 20 hr. |I:\*\*2MP3E0| |L:\*\*1MP3E3| |T:\*\*1MP3E3| |
|  |  |  |  | Annotation |  | 1MP3E0 2MP3E0 | 1MP3E3 1MP3E3 |  |  |  | 1MP3E3 1MP3E3 |  |
|  |  |  |  | Evidence |  | 1 1 | 4 4 |  |  |  | 4 4 |  |
|  |  |  |  | Focus |  | 2 2 | 2 2 |  |  |  | 2 2 |  |
|  |  |  |  | Polarity |  | 7 7 | 7 7 |  |  |  | 7 7 |  |
| 616 | 616 | 8530383\_34 | Shown is the relationship of full-length clone W5(h15R), the form most closely homologous to the murine IL-15Ralpha, to clone A212 (h15RdeltaE3), |I:\*\*1SP3E3| which lacks exon 3, |I:\*\*2SN3E3| and clone A133(h15RaltC), which contains a 120-bp insertion at the position of intron 6 ( panel A). |I:\*\*3SP3E3| |L:\*\*1GSP3E3| |T:\*\*1SP3E3| |
|  |  |  |  | Annotation |  | 1SP3E3 2SN3E3 3SP3E3 | 1GSP3E3 1GSP3E3 1GSP3E3 |  |  |  | 1SP3E3 1SP3E3 1SP3E3 |  |
|  |  |  |  | Evidence |  | 4 4 4 | 4 4 4 |  |  |  | 4 4 4 |  |
|  |  |  |  | Focus |  | 4 4 4 | 5 5 5 |  |  |  | 4 4 4 |  |
|  |  |  |  | Polarity |  | 7 1 7 | 7 7 7 |  |  |  | 7 7 7 |  |
| 617 | 617 | 8681378\_269 | From the larval phenotype, CUL-1 is required for growth regulation in all tissues from the L2 stage onward. |I:\*\*1SP3E0| |L:\*\*1SP3E0| |T:\*\*1SP3E1| |
|  |  |  |  | Annotation |  | 1SP3E0 | 1SP3E0 |  |  |  | 1SP3E1 |  |
|  |  |  |  | Evidence |  | 1 | 1 |  |  |  | 2 |  |
|  |  |  |  | Focus |  | 4 | 4 |  |  |  | 4 |  |
|  |  |  |  | Polarity |  | 7 | 7 |  |  |  | 7 |  |
| 618 | 618 | 9592085\_69 | Both samples were incubated with protein A-Sepharose preconjugated with rabbit anti-Ras antibody (Zymed, San Francisco, CA). |I:\*\*1MP3E0| |L:\*\*1MP3E3| |T:\*\*1MP3E3| |
|  |  |  |  | Annotation |  | 1MP3E0 | 1MP3E3 |  |  |  | 1MP3E3 |  |
|  |  |  |  | Evidence |  | 1 | 4 |  |  |  | 4 |  |
|  |  |  |  | Focus |  | 2 | 2 |  |  |  | 2 |  |
|  |  |  |  | Polarity |  | 7 | 7 |  |  |  | 7 |  |
| 619 | 619 | 11290820\_157 | Hepatocytes and Kupffer cells are proliferating in liver of lal-/- mice. |I:\*\*1SP3E0| |L:\*\*1SP3E0+| |T:\*\*1MP3E3| |
|  |  |  |  | Annotation |  | 1SP3E0 | 1SP3E0+ |  |  |  | 1MP3E3 |  |
|  |  |  |  | Evidence |  | 1 | 1 |  |  |  | 4 |  |
|  |  |  |  | Focus |  | 4 | 4 |  |  |  | 2 |  |
|  |  |  |  | Polarity |  | 7 | 7 |  |  |  | 7 |  |
| 620 | 620 | 10514529\_32 | After 60 min at 37 degrees C, non-internalized E were lysed and the phagocytic index (the number of internalized erythrocytes per 100 P388D1 cells) was determined by light microscopy. |I:\*\*1MP3E0| |L:\*\*1MP3E3| |T:\*\*1MP3E3| |
|  |  |  |  | Annotation |  | 1MP3E0 | 1MP3E3 |  |  |  | 1MP3E3 |  |
|  |  |  |  | Evidence |  | 1 | 4 |  |  |  | 4 |  |
|  |  |  |  | Focus |  | 2 | 2 |  |  |  | 2 |  |
|  |  |  |  | Polarity |  | 7 | 7 |  |  |  | 7 |  |
| 621 | 621 | 11087000\_385 | Sound was delivered to the tympanic membrane by a closed acoustic system comprised of two Bruel & Kjaer 4133 1/2-inch microphones for delivering tones and a single Bruel & Kjaer 3135 1/4-inch microphone for monitoring sound pressure at the tympanum. |I:\*\*1MP3E0| |L:\*\*1MP3E3| |T:\*\*1MP3E3| |
|  |  |  |  | Annotation |  | 1MP3E0 | 1MP3E3 |  |  |  | 1MP3E3 |  |
|  |  |  |  | Evidence |  | 1 | 4 |  |  |  | 4 |  |
|  |  |  |  | Focus |  | 2 | 2 |  |  |  | 2 |  |
|  |  |  |  | Polarity |  | 7 | 7 |  |  |  | 7 |  |
| 622 | 622 | 10230402\_316 | To prepare trapped TEC at the terminator, the same TB was used but KCl concentration was 5 mM. |I:\*\*1MP3E0| |L:\*\*1MP3E3| |T:\*\*1MP3E3| |
|  |  |  |  | Annotation |  | 1MP3E0 | 1MP3E3 |  |  |  | 1MP3E3 |  |
|  |  |  |  | Evidence |  | 1 | 4 |  |  |  | 4 |  |
|  |  |  |  | Focus |  | 2 | 2 |  |  |  | 2 |  |
|  |  |  |  | Polarity |  | 7 | 7 |  |  |  | 7 |  |
| 623 | 623 | 11925572\_1 | Psychiatric nursing, while vibrant and applicable once, is predicated on an approach to care and an understanding of mental illness that reflects an older time. |I:\*\*1SP3E0| |L:\*\*1MP3E3| |T:\*\*1GP3E0| |
|  |  |  |  | Annotation |  | 1SP3E0 | 1MP3E3 |  |  |  | 1GP3E0 |  |
|  |  |  |  | Evidence |  | 1 | 4 |  |  |  | 1 |  |
|  |  |  |  | Focus |  | 4 | 2 |  |  |  | 1 |  |
|  |  |  |  | Polarity |  | 7 | 7 |  |  |  | 7 |  |
| 624 | 624 | 9323138\_53 | Following addition of purified full-length RHA protein, however, GST-CH3 resin ( Figure 3) |I:\*\*1SP3E3| |L:\*\*1SP3E3| |T:\*\*1SMP3E3| but not GST resin alone (not shown) |I:\*\*2SN3E3| was found to associate with pol II, |I:\*\*3SP3E3| both by Western blot ( Figure 3, top) and in vitro transcription ( Figure 3, bottom) assay. |I:\*\*4MP3E3| |L:\*\*2SN3E3| |T:\*\*2SN3E3| |
|  |  |  |  | Annotation |  | 1SP3E3 2SN3E3 3SP3E3 4MP3E3 | 1SP3E3 2SN3E3 2SN3E3 2SN3E3 |  |  |  | 1SMP3E3 2SN3E3 2SN3E3 2SN3E3 |  |
|  |  |  |  | Evidence |  | 4 4 4 4 | 4 4 4 4 |  |  |  | 4 4 4 4 |  |
|  |  |  |  | Focus |  | 4 4 4 2 | 4 4 4 4 |  |  |  | 6 4 4 4 |  |
|  |  |  |  | Polarity |  | 7 1 7 7 | 7 1 1 1 |  |  |  | 7 1 1 1 |  |
| 625 | 625 | 9083007\_2 | Both enzymes exhibit a curvilinear dependence of k2,obs on peroxide concentration |I:\*\*1SP3E0| that has led us to consider two alternative mechanisms, represented by Equations 3 and 4 for Fe-PGHS and Equations 6 and 7 for Mn-PGHS. |I:\*\*2SP0E3| |L:\*\*1GSP3E0| |T:\*\*1SP3E0| |
|  |  |  |  | Annotation |  | 1SP3E0 2SP0E3 | 1GSP3E0 1GSP3E0 |  |  |  | 1SP3E0 1SP3E0 |  |
|  |  |  |  | Evidence |  | 1 4 | 1 1 |  |  |  | 1 1 |  |
|  |  |  |  | Focus |  | 4 4 | 5 5 |  |  |  | 4 4 |  |
|  |  |  |  | Polarity |  | 7 4 | 7 7 |  |  |  | 7 7 |  |
| 626 | 626 | 9700168\_30 | Laminin was purified from Engelbreth-Holm Swarm sarcoma as published (Timpl et al., 1979 ). |I:\*\*1SP3E2| |L:\*\*1MP3E2| |T:\*\*1MP3E2| |
|  |  |  |  | Annotation |  | 1SP3E2 | 1MP3E2 |  |  |  | 1MP3E2 |  |
|  |  |  |  | Evidence |  | 3 | 3 |  |  |  | 3 |  |
|  |  |  |  | Focus |  | 4 | 2 |  |  |  | 2 |  |
|  |  |  |  | Polarity |  | 7 | 7 |  |  |  | 7 |  |
| 627 | 627 | 10548594\_100 | Figure 3 shows the typical outer membrane protein profiles of E. coli and K. pneumoniae isolates separated by SDS-PAGE. |I:\*\*1SGP3E3| |L:\*\*1SP3E3| |T:\*\*1MP3E3| |
|  |  |  |  | Annotation |  | 1SGP3E3 | 1SP3E3 |  |  |  | 1MP3E3 |  |
|  |  |  |  | Evidence |  | 4 | 4 |  |  |  | 4 |  |
|  |  |  |  | Focus |  | 5 | 4 |  |  |  | 2 |  |
|  |  |  |  | Polarity |  | 7 | 7 |  |  |  | 7 |  |
| 628 | 628 | 9684747\_154 | When 18:1 c was present at sn -2, |I:\*\*1SGP3E0| both enzymes derived >95% of acyl groups from sn -2. |I:\*\*2SP3E0| |L:\*\*1SP3E0| |T:\*\*1GP3E0| |
|  |  |  |  | Annotation |  | 1SGP3E0 2SP3E0 | 1SP3E0 1SP3E0 |  |  |  | 1GP3E0 1GP3E0 |  |
|  |  |  |  | Evidence |  | 1 1 | 1 1 |  |  |  | 1 1 |  |
|  |  |  |  | Focus |  | 5 4 | 4 4 |  |  |  | 1 1 |  |
|  |  |  |  | Polarity |  | 7 7 | 7 7 |  |  |  | 7 7 |  |
| 629 | 629 | 9482726\_136 | To determine the sequence changes responsible for the reversion, |I:\*\*1SGP0E3| we sequenced the G protein mRNAs from the VSV-CT1 mutant and the revertant after RT-PCR of mRNA from infected cells. |I:\*\*2SGP3E3| |L:\*\*1GMP3E3| |T:\*\*1MP3E0| |
|  |  |  |  | Annotation |  | 1SGP0E3 2SGP3E3 | 1GMP3E3 1GMP3E3 |  |  |  | 1MP3E0 1MP3E0 |  |
|  |  |  |  | Evidence |  | 4 4 | 4 4 |  |  |  | 1 1 |  |
|  |  |  |  | Focus |  | 5 5 | 3 3 |  |  |  | 2 2 |  |
|  |  |  |  | Polarity |  | 4 7 | 7 7 |  |  |  | 7 7 |  |
| 630 | 630 | 9323141\_220 | The following antibodies were used: MAb against the lumenal domain of VSV-G (anti-VG; from Kai Simons, EMBL, Heidelberg, Germany); MAb P5D4 and anti-P4 against the C terminus of VSV-G (Kreis and Lodish, 1986 ); MAb CM1A10 (anti-beta''-COP; Palmer et al., 1993 ); anti-mammalian Sec13p (Tang et al., 1997 ); anti-calnexin (Hammond and Helenius, 1994 ), MAb G1/93 (anti-ERGIC-53; Schweizer et al., 1988 ); MAb 1D3 (anti-PDI; from Stephen Fuller, EMBL, Heidelberg, Germany); anti-KDEL-receptor (Tang, 1993); MAb against galactosyltransferase (Kawano et al., 1994 ); MAb 1A2 against tubulin (Kreis, 1987 ); and MAb AD7 against p200 (Narula et al., 1992 ). |I:\*\*1MP3E0| |L:\*\*1MP3E23| |T:\*\*1MP3E2| |
|  |  |  |  | Annotation |  | 1MP3E0 | 1MP3E23 |  |  |  | 1MP3E2 |  |
|  |  |  |  | Evidence |  | 1 | 3 |  |  |  | 3 |  |
|  |  |  |  | Focus |  | 2 | 2 |  |  |  | 2 |  |
|  |  |  |  | Polarity |  | 7 | 7 |  |  |  | 7 |  |
| 631 | 631 | 12031766\_2 | This anomaly has been explained with the persistence of the left superior cardinal vein. |I:\*\*1SP3E0| |L:\*\*1SP3E0| |T:\*\*1GP3E0| |
|  |  |  |  | Annotation |  | 1SP3E0 | 1SP3E0 |  |  |  | 1GP3E0 |  |
|  |  |  |  | Evidence |  | 1 | 1 |  |  |  | 1 |  |
|  |  |  |  | Focus |  | 4 | 4 |  |  |  | 1 |  |
|  |  |  |  | Polarity |  | 7 | 7 |  |  |  | 7 |  |
| 632 | 632 | 11416155\_321 | The expression of CDC25A also could affect the responsiveness of cancer cells to oxidative and/or genotoxic stresses caused by cancer therapies. |I:\*\*1SP2E0| |L:\*\*1SP1E0| |T:\*\*1SP3E0| |
|  |  |  |  | Annotation |  | 1SP2E0 | 1SP1E0 |  |  |  | 1SP3E0 |  |
|  |  |  |  | Evidence |  | 1 | 1 |  |  |  | 1 |  |
|  |  |  |  | Focus |  | 4 | 4 |  |  |  | 4 |  |
|  |  |  |  | Polarity |  | 6 | 5 |  |  |  | 7 |  |
| 633 | 633 | 9067330\_226 | This interpretation is supported by histological studies |I:\*\*1SGP3E3| |L:\*\*1SP3E1| which demonstrate that tumor DOX fluorescence did not correlate with TAM location |I:\*\*2SN3E1| |L:\*\*2SN3E0| and often was completely absent in CD IIb positive cells after injection of liposomal DOX. |I:\*\*3SP3E1| |L:\*\*3SP3E0-| |T:\*\*1GN2E0| |
|  |  |  |  | Annotation |  | 1SGP3E3 2SN3E1 3SP3E1 | 1SP3E1 2SN3E0 3SP3E0- |  |  |  | 1GN2E0 1GN2E0 1GN2E0 |  |
|  |  |  |  | Evidence |  | 4 2 2 | 2 1 1 |  |  |  | 1 1 1 |  |
|  |  |  |  | Focus |  | 5 4 4 | 4 4 4 |  |  |  | 1 1 1 |  |
|  |  |  |  | Polarity |  | 7 1 7 | 7 1 7 |  |  |  | 2 2 2 |  |
| 634 | 634 | 9590173\_144 | As illustrated in Figure 6A, cells from both Jak2-deficient and wild-type embryos responded to IL-6 |I:\*\*1SP3E3| |T:\*\*1SP3E3| as assessed by tyrosine phosphorylation of Jak1 and Tyk2, and gp130 and Stat3. |I:\*\*2MP3E3| |L:\*\*1SP3E3| |T:\*\*2GP3E3| |
|  |  |  |  | Annotation |  | 1SP3E3 2MP3E3 | 1SP3E3 1SP3E3 |  |  |  | 1SP3E3 2GP3E3 |  |
|  |  |  |  | Evidence |  | 4 4 | 4 4 |  |  |  | 4 4 |  |
|  |  |  |  | Focus |  | 4 2 | 4 4 |  |  |  | 4 1 |  |
|  |  |  |  | Polarity |  | 7 7 | 7 7 |  |  |  | 7 7 |  |
| 635 | 635 | 12002669\_2 | Cell injury was assessed |I:\*\*1SGP0E0| by lactate dehydrogenase (LDH) leakage and by reduction of the tetrazolium salt WST-1 to formazan by mitochondrial metabolic activity. |I:\*\*2MP3E0| |L:\*\*1MP3E3| |T:\*\*1MP3E3| |
|  |  |  |  | Annotation |  | 1SGP0E0 2MP3E0 | 1MP3E3 1MP3E3 |  |  |  | 1MP3E3 1MP3E3 |  |
|  |  |  |  | Evidence |  | 1 1 | 4 4 |  |  |  | 4 4 |  |
|  |  |  |  | Focus |  | 5 2 | 2 2 |  |  |  | 2 2 |  |
|  |  |  |  | Polarity |  | 4 7 | 7 7 |  |  |  | 7 7 |  |
| 636 | 636 | 11463813\_62 | Total RNA was isolated with the RNA INSTAPURE kit (Eurogentech, Seraing, Belgium) in accordance with the manufacturer's recommendations. |I:\*\*1MP3E3| |L:\*\*1MP3E23| |T:\*\*1MP3E3| |
|  |  |  |  | Annotation |  | 1MP3E3 | 1MP3E23 |  |  |  | 1MP3E3 |  |
|  |  |  |  | Evidence |  | 4 | 3 |  |  |  | 4 |  |
|  |  |  |  | Focus |  | 2 | 2 |  |  |  | 2 |  |
|  |  |  |  | Polarity |  | 7 | 7 |  |  |  | 7 |  |
| 637 | 637 | 12044748\_2 | In the present study, observers were required to judge symmetry in arrays composed of elements varying not only in color, but also in size, spatial frequency and orientation. |I:\*\*1MP3E3| |L:\*\*1MP3E3| |T:\*\*1MP3E3| |
|  |  |  |  | Annotation |  | 1MP3E3 | 1MP3E3 |  |  |  | 1MP3E3 |  |
|  |  |  |  | Evidence |  | 4 | 4 |  |  |  | 4 |  |
|  |  |  |  | Focus |  | 2 | 2 |  |  |  | 2 |  |
|  |  |  |  | Polarity |  | 7 | 7 |  |  |  | 7 |  |
| 638 | 638 | 12044748\_4 | It is proposed that this increase reflects a sequential strategy whereby coarse "binary maps" are created by attentional filtering, |I:\*\*1SP3E0| and the symmetry of each map is then checked. |I:\*\*2MP3E0| |L:\*\*1GSP3E0| |T:\*\*1GP2E0+| |
|  |  |  |  | Annotation |  | 1SP3E0 2MP3E0 | 1GSP3E0 1GSP3E0 |  |  |  | 1GP2E0+ 1GP2E0+ |  |
|  |  |  |  | Evidence |  | 1 1 | 1 1 |  |  |  | 1 1 |  |
|  |  |  |  | Focus |  | 4 2 | 5 5 |  |  |  | 1 1 |  |
|  |  |  |  | Polarity |  | 7 7 | 7 7 |  |  |  | 6 6 |  |
| 639 | 639 | 10982831\_106 | A one-tailed t test was performed to test for differences between the means of each of these values relative to those for the BFP-expressing cell line. |I:\*\*1MP3E0| |L:\*\*1MP3E3| |T:\*\*1MP3E3| |
|  |  |  |  | Annotation |  | 1MP3E0 | 1MP3E3 |  |  |  | 1MP3E3 |  |
|  |  |  |  | Evidence |  | 1 | 4 |  |  |  | 4 |  |
|  |  |  |  | Focus |  | 2 | 2 |  |  |  | 2 |  |
|  |  |  |  | Polarity |  | 7 | 7 |  |  |  | 7 |  |
| 640 | 640 | 9609084\_9 | Abciximab treatment reduced death or myocardial infarction among diabetic and nondiabetic patients (hazard ratios, 0.28 [95% confidence interval (CI), 0.13 to 0.57] and 0.47 [95% CI, 0.33 to 0.70] at 30 days for diabetics and nondiabetics, respectively, and 0.36 [95% CI, 0.21 to 0.61] and 0.60 [95% CI, 0.44 to 0.82] at 6 months for diabetics and nondiabetics, respectively). |I:\*\*1SP3E0-| |L:\*\*1MP3E3| |T:\*\*1SN3E3| |
|  |  |  |  | Annotation |  | 1SP3E0- | 1MP3E3 |  |  |  | 1SN3E3 |  |
|  |  |  |  | Evidence |  | 1 | 4 |  |  |  | 4 |  |
|  |  |  |  | Focus |  | 4 | 2 |  |  |  | 4 |  |
|  |  |  |  | Polarity |  | 7 | 7 |  |  |  | 1 |  |
| 641 | 641 | 12676581\_1 | Tumor angiogenesis is postulated to be regulated by the balance between pro- and anti-angiogenic factors. |I:\*\*1SP3E0| |L:\*\*1GSP3E0| |T:\*\*1GP2E0| |
|  |  |  |  | Annotation |  | 1SP3E0 | 1GSP3E0 |  |  |  | 1GP2E0 |  |
|  |  |  |  | Evidence |  | 1 | 1 |  |  |  | 1 |  |
|  |  |  |  | Focus |  | 4 | 5 |  |  |  | 1 |  |
|  |  |  |  | Polarity |  | 7 | 7 |  |  |  | 6 |  |
| 642 | 642 | 10893236\_5 | Bip, CalP, and CalM are calcium-binding proteins and implicated in calcium homeostasis, |L:\*\*1SP3E0| |T:\*\*1GP3E0| whereas SFRP-2 is a negative regulator of Wnt signaling. |L:\*\*2SP3E0-| |I:\*\*1SP3E0| |T:\*\*2GN3E0| |
|  |  |  |  | Annotation |  | 1SP3E0 1SP3E0 | 1SP3E0 2SP3E0- |  |  |  | 1GP3E0 2GN3E0 |  |
|  |  |  |  | Evidence |  | 1 1 | 1 1 |  |  |  | 1 1 |  |
|  |  |  |  | Focus |  | 4 4 | 4 4 |  |  |  | 1 1 |  |
|  |  |  |  | Polarity |  | 7 7 | 7 7 |  |  |  | 7 1 |  |
| 643 | 643 | 12033244\_3 | The catalytic reduction of carbon dioxide was performed using thin mercury film and glassy carbon electrodes modified with cubic phases containing the catalyst. |I:\*\*1MP3E0| |L:\*\*1MP3E3| |T:\*\*1MP3E0| |
|  |  |  |  | Annotation |  | 1MP3E0 | 1MP3E3 |  |  |  | 1MP3E0 |  |
|  |  |  |  | Evidence |  | 1 | 4 |  |  |  | 1 |  |
|  |  |  |  | Focus |  | 2 | 2 |  |  |  | 2 |  |
|  |  |  |  | Polarity |  | 7 | 7 |  |  |  | 7 |  |
| 644 | 644 | 11502253\_334 | Further analysis of the function and regulation of Ngn2 and other bHLH genes will shed more light |I:\*\*1SGP0E0| on how these genes may act to synchronize genetic pathways that specify neuronal identity with the progression of neural lineage development. |I:\*\*2SP0E0| |L:\*\*1GSP0E0| |T:\*\*1GP3E0| |
|  |  |  |  | Annotation |  | 1SGP0E0 2SP0E0 | 1GSP0E0 1GSP0E0 |  |  |  | 1GP3E0 1GP3E0 |  |
|  |  |  |  | Evidence |  | 1 1 | 1 1 |  |  |  | 1 1 |  |
|  |  |  |  | Focus |  | 5 4 | 5 5 |  |  |  | 1 1 |  |
|  |  |  |  | Polarity |  | 4 4 | 4 4 |  |  |  | 7 7 |  |
| 645 | 645 | 11859073\_12 | Of the A. aeolicus proteins studied in this report, these two subunits display the least percent identity to the corresponding E. coli subunit sequences (see Fig. 1 B). |L:\*\*1SN3E3| |I:\*\*1SP3E3| |T:\*\*1SP3E3| Despite the rather wide sequence variations in A. aeolicusdelta and delta' compared with their E. coli counterparts, |L:\*\*1GSP3E3| they are remarkably similar to the behavior of E. coli proteins in ability to form deltadelta' and taudeltadelta' complexes which remain associated during gel filtration analysis ( ). |L:\*\*2SP3E3| |I:\*\*1SP3E2| |T:\*\*1SN3E3| |
|  |  |  |  | Annotation |  | 1SP3E3 1SP3E2 1SP3E2 | 1SN3E3 1GSP3E3 2SP3E3 |  |  |  | 1SP3E3 1SN3E3 1SN3E3 |  |
|  |  |  |  | Evidence |  | 4 3 3 | 4 4 4 |  |  |  | 4 4 4 |  |
|  |  |  |  | Focus |  | 4 4 4 | 4 5 4 |  |  |  | 4 4 4 |  |
|  |  |  |  | Polarity |  | 7 7 7 | 1 7 7 |  |  |  | 7 1 1 |  |
| 646 | 646 | 9528791\_211 | It has previously been demonstrated that dissociation of U4 is highly sensitive to ATP concentration ( 53). |I:\*\*1SP3E2| |L:\*\*1SP3E2| |T:\*\*1SP3E2| |
|  |  |  |  | Annotation |  | 1SP3E2 | 1SP3E2 |  |  |  | 1SP3E2 |  |
|  |  |  |  | Evidence |  | 3 | 3 |  |  |  | 3 |  |
|  |  |  |  | Focus |  | 4 | 4 |  |  |  | 4 |  |
|  |  |  |  | Polarity |  | 7 | 7 |  |  |  | 7 |  |
| 647 | 647 | 10722578\_66 | After being washed, the grids were negatively stained with freshly prepared 4% uranyl acetate (pH 4.5) and studied by transmission electron microscopy with a Phillips Model 300 operating at 60 or 80 kV. |I:\*\*1MP3E0| |L:\*\*1MP3E13| |T:\*\*1MP3E3| |
|  |  |  |  | Annotation |  | 1MP3E0 | 1MP3E13 |  |  |  | 1MP3E3 |  |
|  |  |  |  | Evidence |  | 1 | 2 |  |  |  | 4 |  |
|  |  |  |  | Focus |  | 2 | 2 |  |  |  | 2 |  |
|  |  |  |  | Polarity |  | 7 | 7 |  |  |  | 7 |  |
| 648 | 648 | 11254742\_82 | Pigeon LDL was oxidized by dialysis at 4 degrees C for 18 h against 0.9% NaCl containing 20 mumol/l of CuSO4. |I:\*\*1MP3E0| |L:\*\*1MP3E3| |T:\*\*1MP3E3| |
|  |  |  |  | Annotation |  | 1MP3E0 | 1MP3E3 |  |  |  | 1MP3E3 |  |
|  |  |  |  | Evidence |  | 1 | 4 |  |  |  | 4 |  |
|  |  |  |  | Focus |  | 2 | 2 |  |  |  | 2 |  |
|  |  |  |  | Polarity |  | 7 | 7 |  |  |  | 7 |  |
| 649 | 649 | 10428966\_289 | The reaction products were run on a 12% SDS-polyacrylamide gel. |I:\*\*1MP3E0| |L:\*\*1MP3E3| |T:\*\*1MP3E3| |
|  |  |  |  | Annotation |  | 1MP3E0 | 1MP3E3 |  |  |  | 1MP3E3 |  |
|  |  |  |  | Evidence |  | 1 | 4 |  |  |  | 4 |  |
|  |  |  |  | Focus |  | 2 | 2 |  |  |  | 2 |  |
|  |  |  |  | Polarity |  | 7 | 7 |  |  |  | 7 |  |
| 650 | 650 | 10525091\_4 | The higher mitochondrial function was also seen in YM934-treated hearts at the end of ischemia. |I:\*\*1SP3E0| |L:\*\*1SP3E0+| |T:\*\*1GP3E0| |
|  |  |  |  | Annotation |  | 1SP3E0 | 1SP3E0+ |  |  |  | 1GP3E0 |  |
|  |  |  |  | Evidence |  | 1 | 1 |  |  |  | 1 |  |
|  |  |  |  | Focus |  | 4 | 4 |  |  |  | 1 |  |
|  |  |  |  | Polarity |  | 7 | 7 |  |  |  | 7 |  |
| 651 | 651 | 12163503\_34 | alpha4 Integrin presentation and clustering on lymphocyte microvilli have been suggested to regulate lymphocyte capture on ligand under shear flow ( , ). |I:\*\*1SP1E2| |L:\*\*1SP1E0| |T:\*\*1GP2E2| |
|  |  |  |  | Annotation |  | 1SP1E2 | 1SP1E0 |  |  |  | 1GP2E2 |  |
|  |  |  |  | Evidence |  | 3 | 1 |  |  |  | 3 |  |
|  |  |  |  | Focus |  | 4 | 4 |  |  |  | 1 |  |
|  |  |  |  | Polarity |  | 5 | 5 |  |  |  | 6 |  |
| 652 | 652 | 12003609\_8 | Only 3 (18%) of 17 controls had SAS (P =.005). |I:\*\*1SP3E0| |L:\*\*1SP3E3| |T:\*\*1SP3E3| |
|  |  |  |  | Annotation |  | 1SP3E0 | 1SP3E3 |  |  |  | 1SP3E3 |  |
|  |  |  |  | Evidence |  | 1 | 4 |  |  |  | 4 |  |
|  |  |  |  | Focus |  | 4 | 4 |  |  |  | 4 |  |
|  |  |  |  | Polarity |  | 7 | 7 |  |  |  | 7 |  |
| 653 | 653 | 11682507\_146 | Few studies have addressed |I:\*\*1SGP3E1| the possible correlation between specific serovars or genotypes and disease manifestations and severity of disease. |I:\*\*2SP1E0| |L:\*\*1GSN3E0| |T:\*\*1GP3E0| |
|  |  |  |  | Annotation |  | 1SGP3E1 2SP1E0 | 1GSN3E0 1GSN3E0 |  |  |  | 1GP3E0 1GP3E0 |  |
|  |  |  |  | Evidence |  | 2 1 | 1 1 |  |  |  | 1 1 |  |
|  |  |  |  | Focus |  | 5 4 | 5 5 |  |  |  | 1 1 |  |
|  |  |  |  | Polarity |  | 7 5 | 1 1 |  |  |  | 7 7 |  |
| 654 | 654 | 12072480\_205 | The effect of heterogeneity in the recombination rate on delta was investigated ( Fig 2) |I:\*\*1SGP0E3| because the effect of R on delta is relatively large. |I:\*\*2SP3E3| |L:\*\*1GMP3E3| |T:\*\*1SP3E3| |
|  |  |  |  | Annotation |  | 1SGP0E3 2SP3E3 | 1GMP3E3 1GMP3E3 |  |  |  | 1SP3E3 1SP3E3 |  |
|  |  |  |  | Evidence |  | 4 4 | 4 4 |  |  |  | 4 4 |  |
|  |  |  |  | Focus |  | 5 4 | 3 3 |  |  |  | 4 4 |  |
|  |  |  |  | Polarity |  | 4 7 | 7 7 |  |  |  | 7 7 |  |
| 655 | 655 | 12113551\_2 | To date, the role of antigen presenting cells (APCs), |I:\*\*1SGP3E3| which are known to be critical in the regulation of T cell response, |I:\*\*2SP3E0| |T:\*\*1GP3E0| has been poorly investigated in periodontitis. |I:\*\*3SP0E3| |L:\*\*1GSP3E0| |T:\*\*2GN3E0| |
|  |  |  |  | Annotation |  | 1SGP3E3 2SP3E0 3SP0E3 | 1GSP3E0 1GSP3E0 1GSP3E0 |  |  |  | 1GP3E0 1GP3E0 2GN3E0 |  |
|  |  |  |  | Evidence |  | 4 1 4 | 1 1 1 |  |  |  | 1 1 1 |  |
|  |  |  |  | Focus |  | 5 4 4 | 5 5 5 |  |  |  | 1 1 1 |  |
|  |  |  |  | Polarity |  | 7 7 4 | 7 7 7 |  |  |  | 7 7 1 |  |
| 656 | 656 | 10791958\_1 | Ubiquitin-independent Degradation of Native CaM in Vitro -- |L:\*\*ERROR| |I:\*\*ERROR| |T:\*\*ERROR| Although our initial goal was to identify the mechanism for the selective degradation of age-damaged proteins using CaM as a model substrate, |L:\*\*1GSP3E3| |I:\*\*1SGP0E3| |T:\*\*1GP3E0| these studies have uncovered unexpected findings regarding the pathway for the degradation of the native molecule. |L:\*\*2SP3E1| |I:\*\*2SGP3E3| |T:\*\*2GN3E0| |
|  |  |  |  | Annotation |  | ERROR 1SGP0E3 2SGP3E3 | ERROR 1GSP3E3 2SP3E1 |  |  |  | ERROR 1GP3E0 2GN3E0 |  |
|  |  |  |  | Evidence |  | -1000 4 4 | -1000 4 2 |  |  |  | -1000 1 1 |  |
|  |  |  |  | Focus |  | -1000 5 5 | -1000 5 4 |  |  |  | -1000 1 1 |  |
|  |  |  |  | Polarity |  | -1000 4 7 | -1000 7 7 |  |  |  | -1000 7 1 |  |
| 657 | 657 | 10523671\_162 | To establish that the band intensities produced for Gal80p and GSTG4ADp were within the linear response range for the chemiluminescence method, |I:\*\*1SP0E3| we performed pilot experiments |I:\*\*2SGP3E3| in which we loaded different amounts of the GT-Sepharose eluate per lane and used several different film exposure times. |I:\*\*3MP3E3| |L:\*\*1MSP3E3| |T:\*\*1MP3E3| |
|  |  |  |  | Annotation |  | 1SP0E3 2SGP3E3 3MP3E3 | 1MSP3E3 1MSP3E3 1MSP3E3 |  |  |  | 1MP3E3 1MP3E3 1MP3E3 |  |
|  |  |  |  | Evidence |  | 4 4 4 | 4 4 4 |  |  |  | 4 4 4 |  |
|  |  |  |  | Focus |  | 4 5 2 | 6 6 6 |  |  |  | 2 2 2 |  |
|  |  |  |  | Polarity |  | 4 7 7 | 7 7 7 |  |  |  | 7 7 7 |  |
| 658 | 658 | 12204687\_25 | HRP-2 has been shown to bind multiple molecules of heme and to mediate heme polymerization in vitro [10, 11] . |I:\*\*1SP3E2| |L:\*\*1SP3E2| |T:\*\*1GP3E2| |
|  |  |  |  | Annotation |  | 1SP3E2 | 1SP3E2 |  |  |  | 1GP3E2 |  |
|  |  |  |  | Evidence |  | 3 | 3 |  |  |  | 3 |  |
|  |  |  |  | Focus |  | 4 | 4 |  |  |  | 1 |  |
|  |  |  |  | Polarity |  | 7 | 7 |  |  |  | 7 |  |
| 659 | 659 | 8621388\_5 | The present study is the first to carry out a systematic survey |I:\*\*1SGP3E3| of a possible difference in the recognition mechanisms of Ras by its distinct effectors. |I:\*\*2SP0E3| |L:\*\*1GSP3E3| |T:\*\*1GP3E3| |
|  |  |  |  | Annotation |  | 1SGP3E3 2SP0E3 | 1GSP3E3 1GSP3E3 |  |  |  | 1GP3E3 1GP3E3 |  |
|  |  |  |  | Evidence |  | 4 4 | 4 4 |  |  |  | 4 4 |  |
|  |  |  |  | Focus |  | 5 4 | 5 5 |  |  |  | 1 1 |  |
|  |  |  |  | Polarity |  | 7 4 | 7 7 |  |  |  | 7 7 |  |
| 660 | 660 | 9425166\_8 | We suggest that the signaling complex, |I:\*\*1SP1E3| which has been shown by others to comprise armadillo and a member of the lymphocyte enhancer binding factor-1/T cell factor-family, |I:\*\*2SP3E1| |T:\*\*1GP3E1| may contain an additional factor that normally binds to the COOH-terminal region of armadillo. |I:\*\*3SP1E3| |L:\*\*1SP1E3| |T:\*\*2GP3E3| |
|  |  |  |  | Annotation |  | 1SP1E3 2SP3E1 3SP1E3 | 1SP1E3 1SP1E3 1SP1E3 |  |  |  | 1GP3E1 1GP3E1 2GP3E3 |  |
|  |  |  |  | Evidence |  | 4 2 4 | 4 4 4 |  |  |  | 2 2 4 |  |
|  |  |  |  | Focus |  | 4 4 4 | 4 4 4 |  |  |  | 1 1 1 |  |
|  |  |  |  | Polarity |  | 5 7 5 | 5 5 5 |  |  |  | 7 7 7 |  |
| 661 | 661 | 9390512\_106 | Thus, no region of 22q could be excluded, |I:\*\*1SN3E0| |L:\*\*1SN3E3| and the most likely region included q11-q12. |I:\*\*2SP3E0| |L:\*\*2SP2E3| |T:\*\*1MP3E0| |
|  |  |  |  | Annotation |  | 1SN3E0 2SP3E0 | 1SN3E3 2SP2E3 |  |  |  | 1MP3E0 1MP3E0 |  |
|  |  |  |  | Evidence |  | 1 1 | 4 4 |  |  |  | 1 1 |  |
|  |  |  |  | Focus |  | 4 4 | 4 4 |  |  |  | 2 2 |  |
|  |  |  |  | Polarity |  | 1 7 | 1 6 |  |  |  | 7 7 |  |
| 662 | 662 | 8663495\_69 | tPA inhibits the binding of defensin to fibrin and to HUVECs. |I:\*\*1SP3E0| |L:\*\*1SP3E0-| |T:\*\*1SN3E0-| |
|  |  |  |  | Annotation |  | 1SP3E0 | 1SP3E0- |  |  |  | 1SN3E0- |  |
|  |  |  |  | Evidence |  | 1 | 1 |  |  |  | 1 |  |
|  |  |  |  | Focus |  | 4 | 4 |  |  |  | 4 |  |
|  |  |  |  | Polarity |  | 7 | 7 |  |  |  | 1 |  |
| 663 | 663 | 12045153\_23 | ACEDB was adopted by the C. elegans sequencing project at the Sanger Centre and Washington University (Consortium 1998 ). |I:\*\*1MGP3E2| |L:\*\*1GP3E2| |T:\*\*1MP3E2| |
|  |  |  |  | Annotation |  | 1MGP3E2 | 1GP3E2 |  |  |  | 1MP3E2 |  |
|  |  |  |  | Evidence |  | 3 | 3 |  |  |  | 3 |  |
|  |  |  |  | Focus |  | 3 | 1 |  |  |  | 2 |  |
|  |  |  |  | Polarity |  | 7 | 7 |  |  |  | 7 |  |
| 664 | 664 | 11583949\_3 | We show that in germ cells RNF4 expression is strongly modulated during progression of spermatogonia to spermatids, with a peak in spermatocytes. |I:\*\*1SP3E3| |L:\*\*1SP3E3| |T:\*\*1SP3E1| |
|  |  |  |  | Annotation |  | 1SP3E3 | 1SP3E3 |  |  |  | 1SP3E1 |  |
|  |  |  |  | Evidence |  | 4 | 4 |  |  |  | 2 |  |
|  |  |  |  | Focus |  | 4 | 4 |  |  |  | 4 |  |
|  |  |  |  | Polarity |  | 7 | 7 |  |  |  | 7 |  |
| 665 | 665 | 12037699\_5 | A percentage of 44.6% of smokers in the control group vs 46.0% of smokers in the hypertensive groups were found. |I:\*\*1SP3E0| |L:\*\*1SP3E3| |T:\*\*1SP3E3| |
|  |  |  |  | Annotation |  | 1SP3E0 | 1SP3E3 |  |  |  | 1SP3E3 |  |
|  |  |  |  | Evidence |  | 1 | 4 |  |  |  | 4 |  |
|  |  |  |  | Focus |  | 4 | 4 |  |  |  | 4 |  |
|  |  |  |  | Polarity |  | 7 | 7 |  |  |  | 7 |  |
| 666 | 666 | 11713294\_284 | Likewise, inhibition of PtdIns 3-kinase |L:\*\*1SP3E0-| or expression of dominant-negative Rac prevented F(ab'')2 anti-Ig-triggered BCR-F-actin association and receptor internalization, respectively. |L:\*\*2SP3E0+| |I:\*\*1SP3E0| |T:\*\*1SN3E3| |
|  |  |  |  | Annotation |  | 1SP3E0 1SP3E0 | 1SP3E0- 2SP3E0+ |  |  |  | 1SN3E3 1SN3E3 |  |
|  |  |  |  | Evidence |  | 1 1 | 1 1 |  |  |  | 4 4 |  |
|  |  |  |  | Focus |  | 4 4 | 4 4 |  |  |  | 4 4 |  |
|  |  |  |  | Polarity |  | 7 7 | 7 7 |  |  |  | 1 1 |  |
| 667 | 667 | 8996174\_85 | In the rabbit aorta and the prostate phenylephrine elicited contractions with potencies (EC50) of approximately 0.3 and 4 muM, respectively. |I:\*\*1SP3E0| |L:\*\*1SP3E0| |T:\*\*1SP3E3| |
|  |  |  |  | Annotation |  | 1SP3E0 | 1SP3E0 |  |  |  | 1SP3E3 |  |
|  |  |  |  | Evidence |  | 1 | 1 |  |  |  | 4 |  |
|  |  |  |  | Focus |  | 4 | 4 |  |  |  | 4 |  |
|  |  |  |  | Polarity |  | 7 | 7 |  |  |  | 7 |  |
| 668 | 668 | 9827803\_219 | Organs were well vascularized, |I:\*\*1SP3E3| |L:\*\*1SP3E3| and no obvious hemorrhages were observed |I:\*\*2SN3E3| |L:\*\*2SN3E3| with the exception of the brain (see above) |T:\*\*1SP3E3| and the intestines of newborns and mild hemorrhage in the developing spinal cord (data not shown) and, less frequently, in the lamina propria of the urinary bladder (data not shown). |I:\*\*3SP3E3| |L:\*\*3SP3E3| |T:\*\*2SP3E1| |
|  |  |  |  | Annotation |  | 1SP3E3 2SN3E3 3SP3E3 3SP3E3 | 1SP3E3 2SN3E3 3SP3E3 3SP3E3 |  |  |  | 1SP3E3 1SP3E3 1SP3E3 2SP3E1 |  |
|  |  |  |  | Evidence |  | 4 4 4 4 | 4 4 4 4 |  |  |  | 4 4 4 2 |  |
|  |  |  |  | Focus |  | 4 4 4 4 | 4 4 4 4 |  |  |  | 4 4 4 4 |  |
|  |  |  |  | Polarity |  | 7 1 7 7 | 7 1 7 7 |  |  |  | 7 7 7 7 |  |
| 669 | 669 | 10454563\_112 | The ability of AR to activate expression of a luciferase reporter gene (MMTV-LUC) with an androgen responsive mouse mammary tumor virus promoter was enhanced six- to ninefold by each of these coactivators, compared with the activity of AR alone. |I:\*\*1SP3E0+| |L:\*\*1SP3E0+| |T:\*\*1SP3E3+| |
|  |  |  |  | Annotation |  | 1SP3E0+ | 1SP3E0+ |  |  |  | 1SP3E3+ |  |
|  |  |  |  | Evidence |  | 1 | 1 |  |  |  | 4 |  |
|  |  |  |  | Focus |  | 4 | 4 |  |  |  | 4 |  |
|  |  |  |  | Polarity |  | 7 | 7 |  |  |  | 7 |  |
| 670 | 670 | 11325993\_34 | Samples (200 mul) of laboratory-cultured strains or clinical specimens (see details below) were extracted using QIAamp DNA Mini-Kits (Qiagen, Mississauga, Ontario, Canada) following a tissue or blood extraction protocol and eluted in 100 mul of buffer. |I:\*\*1MP3E0| |L:\*\*1MP3E23| |T:\*\*1MP3E3| |
|  |  |  |  | Annotation |  | 1MP3E0 | 1MP3E23 |  |  |  | 1MP3E3 |  |
|  |  |  |  | Evidence |  | 1 | 3 |  |  |  | 4 |  |
|  |  |  |  | Focus |  | 2 | 2 |  |  |  | 2 |  |
|  |  |  |  | Polarity |  | 7 | 7 |  |  |  | 7 |  |
| 671 | 671 | 11731474\_45 | We find that PIASy antagonizes Wnt-independent and Wnt-induced transcriptional activation by LEF1. |I:\*\*1SP3E3| |L:\*\*1SP3E3| |T:\*\*1SN3E1| |
|  |  |  |  | Annotation |  | 1SP3E3 | 1SP3E3 |  |  |  | 1SN3E1 |  |
|  |  |  |  | Evidence |  | 4 | 4 |  |  |  | 2 |  |
|  |  |  |  | Focus |  | 4 | 4 |  |  |  | 4 |  |
|  |  |  |  | Polarity |  | 7 | 7 |  |  |  | 1 |  |
| 672 | 672 | 12108212\_3 | The chosen quality parameters permitted the level of the durability influence of croissants depending on external (sorbic acid) as well as internal (recipe, aw) conditions to be estimated. |I:\*\*1SGP0E0| |L:\*\*1GMP3E3| |T:\*\*1GP3E0| |
|  |  |  |  | Annotation |  | 1SGP0E0 | 1GMP3E3 |  |  |  | 1GP3E0 |  |
|  |  |  |  | Evidence |  | 1 | 4 |  |  |  | 1 |  |
|  |  |  |  | Focus |  | 5 | 3 |  |  |  | 1 |  |
|  |  |  |  | Polarity |  | 4 | 7 |  |  |  | 7 |  |
| 673 | 673 | 11395478\_16 | Three genes ( SKCa1-3) ( 6) with a conserved genomic organization ( 7) encode SKCa channels, |I:\*\*1SP3E2| their gene products bearing 70-80% amino acid sequence identity. |I:\*\*2SP3E0| |L:\*\*1SP3E2| |T:\*\*1SP3E2| |
|  |  |  |  | Annotation |  | 1SP3E2 2SP3E0 | 1SP3E2 1SP3E2 |  |  |  | 1SP3E2 1SP3E2 |  |
|  |  |  |  | Evidence |  | 3 1 | 3 3 |  |  |  | 3 3 |  |
|  |  |  |  | Focus |  | 4 4 | 4 4 |  |  |  | 4 4 |  |
|  |  |  |  | Polarity |  | 7 7 | 7 7 |  |  |  | 7 7 |  |
| 674 | 674 | 11925450\_191 | Studies of in vivo functions of GalNAc-transferase isoforms are missing due to the inherent difficulty of assessing the finite number of isoforms and specificity in a given cell, |I:\*\*1SGP3E0| |L:\*\*1GSP3E0-| and |T:\*\*1GP3E0| it is presently not possible to address the first option. |I:\*\*2SN3E0| |L:\*\*2GN3E0| |T:\*\*2GN3E0| |
|  |  |  |  | Annotation |  | 1SGP3E0 2SN3E0 2SN3E0 | 1GSP3E0- 2GN3E0 2GN3E0 |  |  |  | 1GP3E0 1GP3E0 2GN3E0 |  |
|  |  |  |  | Evidence |  | 1 1 1 | 1 1 1 |  |  |  | 1 1 1 |  |
|  |  |  |  | Focus |  | 5 4 4 | 5 1 1 |  |  |  | 1 1 1 |  |
|  |  |  |  | Polarity |  | 7 1 1 | 7 1 1 |  |  |  | 7 7 1 |  |
| 675 | 675 | 9722603\_71 | Images were captured using a Axioscop microscope ( Carl Zeiss Inc., Thornwood, NY) equipped with a CCD camera (Photometrics Ltd., Tucson, AZ), or on an MRC 1000 inverted confocal microscope (Bio-Rad Laboratories). |I:\*\*1MP3E0| |L:\*\*1MP3E23| |T:\*\*1MP3E3| |
|  |  |  |  | Annotation |  | 1MP3E0 | 1MP3E23 |  |  |  | 1MP3E3 |  |
|  |  |  |  | Evidence |  | 1 | 3 |  |  |  | 4 |  |
|  |  |  |  | Focus |  | 2 | 2 |  |  |  | 2 |  |
|  |  |  |  | Polarity |  | 7 | 7 |  |  |  | 7 |  |
| 676 | 676 | 11038179\_97 | All samples contained 1.5 muM FITC - RanGDP and 2 mg/ml BSA in TB, and the import reaction was incubated for 15 min at room temperature before washing and fixation. |I:\*\*1MP3E0| |L:\*\*1MP3E3| |T:\*\*1MP3E3| |
|  |  |  |  | Annotation |  | 1MP3E0 | 1MP3E3 |  |  |  | 1MP3E3 |  |
|  |  |  |  | Evidence |  | 1 | 4 |  |  |  | 4 |  |
|  |  |  |  | Focus |  | 2 | 2 |  |  |  | 2 |  |
|  |  |  |  | Polarity |  | 7 | 7 |  |  |  | 7 |  |
| 677 | 677 | 12181333\_4 | The scd5-Delta338 mutant contains larger and depolarized cortical actin patches and a prevalence of G-actin bars. |I:\*\*1SP3E0| |L:\*\*1SP3E0| |T:\*\*1SP3E3+| |
|  |  |  |  | Annotation |  | 1SP3E0 | 1SP3E0 |  |  |  | 1SP3E3+ |  |
|  |  |  |  | Evidence |  | 1 | 1 |  |  |  | 4 |  |
|  |  |  |  | Focus |  | 4 | 4 |  |  |  | 4 |  |
|  |  |  |  | Polarity |  | 7 | 7 |  |  |  | 7 |  |
| 678 | 678 | 12079534\_1 | PURPOSE: To characterize the role of various cellular damagesensing, processing and survival genes in the in-vitro radiosensitivity of haemopoietic colony-forming cells. |I:\*\*1SGP0E3| |L:\*\*1GSP3E3| |T:\*\*1GP3E0| |
|  |  |  |  | Annotation |  | 1SGP0E3 | 1GSP3E3 |  |  |  | 1GP3E0 |  |
|  |  |  |  | Evidence |  | 4 | 4 |  |  |  | 1 |  |
|  |  |  |  | Focus |  | 5 | 5 |  |  |  | 1 |  |
|  |  |  |  | Polarity |  | 4 | 7 |  |  |  | 7 |  |
| 679 | 679 | 11239469\_135 | Subsequently, the fluorescent AMC production was continuously monitored. |I:\*\*1MP3E0| |L:\*\*1MP3E3| |T:\*\*1MP3E3| |
|  |  |  |  | Annotation |  | 1MP3E0 | 1MP3E3 |  |  |  | 1MP3E3 |  |
|  |  |  |  | Evidence |  | 1 | 4 |  |  |  | 4 |  |
|  |  |  |  | Focus |  | 2 | 2 |  |  |  | 2 |  |
|  |  |  |  | Polarity |  | 7 | 7 |  |  |  | 7 |  |
| 680 | 680 | 10473520\_76 | Although p24 antibody was more frequent than p40 antibody in most cases, |L:\*\*1SP3E0| |T:\*\*1MP3E3| in some psychosis patients antibody profiles showed only p40 antibody (Table 2). |L:\*\*2SP3E3| |I:\*\*1SP3E3| |T:\*\*2SP3E3| |
|  |  |  |  | Annotation |  | 1SP3E3 1SP3E3 | 1SP3E0 2SP3E3 |  |  |  | 1MP3E3 2SP3E3 |  |
|  |  |  |  | Evidence |  | 4 4 | 1 4 |  |  |  | 4 4 |  |
|  |  |  |  | Focus |  | 4 4 | 4 4 |  |  |  | 2 4 |  |
|  |  |  |  | Polarity |  | 7 7 | 7 7 |  |  |  | 7 7 |  |
| 681 | 681 | 8910595\_127 | 8, 2237-2241 [Medline] Gorlach, M., Burd, C. G., and Dreyfuss, G. (1994) J. Biol. |I:\*\*ERROR| |L:\*\*1GP3E3| |T:\*\*1GP3E3| |
|  |  |  |  | Annotation |  | ERROR | 1GP3E3 |  |  |  | 1GP3E3 |  |
|  |  |  |  | Evidence |  | -1000 | 4 |  |  |  | 4 |  |
|  |  |  |  | Focus |  | -1000 | 1 |  |  |  | 1 |  |
|  |  |  |  | Polarity |  | -1000 | 7 |  |  |  | 7 |  |
| 682 | 682 | 11181762\_18 | Vesicles containing anionic phospholipids also bind to SR-BI, as do the apolipoproteins A-I, A-II, and C-III, either as lipoprotein-bound or as lipid-free proteins ( 7). |I:\*\*1SP3E2| |L:\*\*1SP3E2| |T:\*\*1SP3E2| |
|  |  |  |  | Annotation |  | 1SP3E2 | 1SP3E2 |  |  |  | 1SP3E2 |  |
|  |  |  |  | Evidence |  | 3 | 3 |  |  |  | 3 |  |
|  |  |  |  | Focus |  | 4 | 4 |  |  |  | 4 |  |
|  |  |  |  | Polarity |  | 7 | 7 |  |  |  | 7 |  |
| 683 | 683 | 10948110\_23 | Recently, our group identified a novel LPS-binding protein, serum amyloid P component (SAP) ( 4). |I:\*\*1SP3E3| |L:\*\*1SP3E2| |T:\*\*1GP3E2| |
|  |  |  |  | Annotation |  | 1SP3E3 | 1SP3E2 |  |  |  | 1GP3E2 |  |
|  |  |  |  | Evidence |  | 4 | 3 |  |  |  | 3 |  |
|  |  |  |  | Focus |  | 4 | 4 |  |  |  | 1 |  |
|  |  |  |  | Polarity |  | 7 | 7 |  |  |  | 7 |  |
| 684 | 684 | 10873836\_110 | The physiological substrates of plasmin, however, are folded proteins rather than peptides. |I:\*\*1SP3E0| |L:\*\*1SP3E0| |T:\*\*1GP3E0| |
|  |  |  |  | Annotation |  | 1SP3E0 | 1SP3E0 |  |  |  | 1GP3E0 |  |
|  |  |  |  | Evidence |  | 1 | 1 |  |  |  | 1 |  |
|  |  |  |  | Focus |  | 4 | 4 |  |  |  | 1 |  |
|  |  |  |  | Polarity |  | 7 | 7 |  |  |  | 7 |  |
| 685 | 685 | 8978603\_332 | We are grateful to all ADPKD family members for their invaluable participation and the Polycystic Kidney Research Foundation for assistance in the collection of tissue samples used in the study. |I:\*\*1GP3E3| |L:\*\*1GP3E3| |T:\*\*1GP3E3| |
|  |  |  |  | Annotation |  | 1GP3E3 | 1GP3E3 |  |  |  | 1GP3E3 |  |
|  |  |  |  | Evidence |  | 4 | 4 |  |  |  | 4 |  |
|  |  |  |  | Focus |  | 1 | 1 |  |  |  | 1 |  |
|  |  |  |  | Polarity |  | 7 | 7 |  |  |  | 7 |  |
| 686 | 686 | 12102594\_9 | They are, however, soluble in a range of organic solvents (e.g. ethyl acetate, acetone, acetone/water, chloroform/methylene chloride, dimethylsulfoxide, dimethylformamide, and/or chloroform/methanol). |I:\*\*1SP3E0| |L:\*\*1SP3E0| |T:\*\*1SGP3E3| |
|  |  |  |  | Annotation |  | 1SP3E0 | 1SP3E0 |  |  |  | 1SGP3E3 |  |
|  |  |  |  | Evidence |  | 1 | 1 |  |  |  | 4 |  |
|  |  |  |  | Focus |  | 4 | 4 |  |  |  | 5 |  |
|  |  |  |  | Polarity |  | 7 | 7 |  |  |  | 7 |  |
| 687 | 687 | 11950881\_2 | The cadherin family of cell adhesion receptors, located in the adherens junction, interact homophilically to mediate strong cell-cell adhesion. |I:\*\*1SP3E0| |L:\*\*1SP3E0| |T:\*\*1GP3E0| |
|  |  |  |  | Annotation |  | 1SP3E0 | 1SP3E0 |  |  |  | 1GP3E0 |  |
|  |  |  |  | Evidence |  | 1 | 1 |  |  |  | 1 |  |
|  |  |  |  | Focus |  | 4 | 4 |  |  |  | 1 |  |
|  |  |  |  | Polarity |  | 7 | 7 |  |  |  | 7 |  |
| 688 | 688 | 12750340\_125 | We conclude that significant evidence for abundant ancestral shared polymorphism |T:\*\*1GP3E0| contradicts the inference from genealogical analysis. |T:\*\*2GN3E0| |I:\*\*1SP2E3| |L:\*\*1SP2E3| |
|  |  |  |  | Annotation |  | 1SP2E3 1SP2E3 | 1SP2E3 1SP2E3 |  |  |  | 1GP3E0 2GN3E0 |  |
|  |  |  |  | Evidence |  | 4 4 | 4 4 |  |  |  | 1 1 |  |
|  |  |  |  | Focus |  | 4 4 | 4 4 |  |  |  | 1 1 |  |
|  |  |  |  | Polarity |  | 6 6 | 6 6 |  |  |  | 7 1 |  |
| 689 | 689 | 11114901\_98 | Vectorette units were ligated onto the ends of the cleaved target DNA. |I:\*\*1MP3E0| |L:\*\*1SP3E0| |T:\*\*1MP3E3| |
|  |  |  |  | Annotation |  | 1MP3E0 | 1SP3E0 |  |  |  | 1MP3E3 |  |
|  |  |  |  | Evidence |  | 1 | 1 |  |  |  | 4 |  |
|  |  |  |  | Focus |  | 2 | 4 |  |  |  | 2 |  |
|  |  |  |  | Polarity |  | 7 | 7 |  |  |  | 7 |  |
| 690 | 690 | 9367987\_302 | Reactions were stopped by addition of SDS (0.1% final concentration) and treated with proteinase K (100 mug per sample for 1 hr at 37 degrees C). |I:\*\*1MP3E0| |L:\*\*1MP3E3| |T:\*\*1MP3E3| |
|  |  |  |  | Annotation |  | 1MP3E0 | 1MP3E3 |  |  |  | 1MP3E3 |  |
|  |  |  |  | Evidence |  | 1 | 4 |  |  |  | 4 |  |
|  |  |  |  | Focus |  | 2 | 2 |  |  |  | 2 |  |
|  |  |  |  | Polarity |  | 7 | 7 |  |  |  | 7 |  |
| 691 | 691 | 10633111\_213 | The present study shows that VirB9 interacts with two other VirB proteins, VirB8 and VirB10. |I:\*\*1SP3E0| |L:\*\*1SP3E3| |T:\*\*1SP3E1| |
|  |  |  |  | Annotation |  | 1SP3E0 | 1SP3E3 |  |  |  | 1SP3E1 |  |
|  |  |  |  | Evidence |  | 1 | 4 |  |  |  | 2 |  |
|  |  |  |  | Focus |  | 4 | 4 |  |  |  | 4 |  |
|  |  |  |  | Polarity |  | 7 | 7 |  |  |  | 7 |  |
| 692 | 692 | 9512515\_314 | Colonies positive by PCR were genotyped by Southern blotting of HindIII-digested DNA and hybridized with random hexamer [32]dCTP-labeled DNA probes (Amersham). |I:\*\*1MP3E0| |L:\*\*1MP3E23| |T:\*\*1MP3E3| |
|  |  |  |  | Annotation |  | 1MP3E0 | 1MP3E23 |  |  |  | 1MP3E3 |  |
|  |  |  |  | Evidence |  | 1 | 3 |  |  |  | 4 |  |
|  |  |  |  | Focus |  | 2 | 2 |  |  |  | 2 |  |
|  |  |  |  | Polarity |  | 7 | 7 |  |  |  | 7 |  |
| 693 | 693 | 10383970\_174 | The turn angle distribution of a cell population is a measure of its directional persistence. |I:\*\*1SP3E0| |L:\*\*1GMP3E0| |T:\*\*1GP3E0| |
|  |  |  |  | Annotation |  | 1SP3E0 | 1GMP3E0 |  |  |  | 1GP3E0 |  |
|  |  |  |  | Evidence |  | 1 | 1 |  |  |  | 1 |  |
|  |  |  |  | Focus |  | 4 | 3 |  |  |  | 1 |  |
|  |  |  |  | Polarity |  | 7 | 7 |  |  |  | 7 |  |
| 694 | 694 | 7622494\_31 | Thus the B ring is primarily a kinetic barrier to binding. |I:\*\*1SP3E0| |L:\*\*1SP3E0| |T:\*\*1GP3E0| |
|  |  |  |  | Annotation |  | 1SP3E0 | 1SP3E0 |  |  |  | 1GP3E0 |  |
|  |  |  |  | Evidence |  | 1 | 1 |  |  |  | 1 |  |
|  |  |  |  | Focus |  | 4 | 4 |  |  |  | 1 |  |
|  |  |  |  | Polarity |  | 7 | 7 |  |  |  | 7 |  |
| 695 | 695 | 10884290\_57 | The culture was grown at 37 degrees C with constant shaking (series 25 incubator/shaker; New Brunswick Scientific, Edison, NJ) until its absorbance reached 0.5 OD at 600 nm, and expression was induced by adding IPTG to a final concentration of 0.4 mM. |I:\*\*1MP3E0| |L:\*\*1MP3E23| |T:\*\*1MP3E3| |
|  |  |  |  | Annotation |  | 1MP3E0 | 1MP3E23 |  |  |  | 1MP3E3 |  |
|  |  |  |  | Evidence |  | 1 | 3 |  |  |  | 4 |  |
|  |  |  |  | Focus |  | 2 | 2 |  |  |  | 2 |  |
|  |  |  |  | Polarity |  | 7 | 7 |  |  |  | 7 |  |
| 696 | 696 | 12433363\_153 | TN thymocytes from wt, Bax / , or Bcl-2tg mice were isolated |I:\*\*1SGP3E3| by negative selection with magnetic beads as described in Experimental Procedures. |I:\*\*2MP3E3| |L:\*\*1GMP3E3| |T:\*\*1MP3E3| |
|  |  |  |  | Annotation |  | 1SGP3E3 2MP3E3 | 1GMP3E3 1GMP3E3 |  |  |  | 1MP3E3 1MP3E3 |  |
|  |  |  |  | Evidence |  | 4 4 | 4 4 |  |  |  | 4 4 |  |
|  |  |  |  | Focus |  | 5 2 | 3 3 |  |  |  | 2 2 |  |
|  |  |  |  | Polarity |  | 7 7 | 7 7 |  |  |  | 7 7 |  |
| 697 | 697 | 12364586\_11 | Four SNP markers from DAAO were found to be associated with schizophrenia in the Canadian samples. |I:\*\*1SP3E0| |L:\*\*1SP3E3| |T:\*\*1SP3E0| |
|  |  |  |  | Annotation |  | 1SP3E0 | 1SP3E3 |  |  |  | 1SP3E0 |  |
|  |  |  |  | Evidence |  | 1 | 4 |  |  |  | 1 |  |
|  |  |  |  | Focus |  | 4 | 4 |  |  |  | 4 |  |
|  |  |  |  | Polarity |  | 7 | 7 |  |  |  | 7 |  |
| 698 | 698 | 12163503\_39 | However, spontaneous VLA-4 and LFA-1 adhesiveness developed by T cells at rapid adhesive contacts are insensitive to PI3K inhibition. |I:\*\*1SN3E0| |L:\*\*1SN3E3| |T:\*\*1SN3E0| 4 Raft clustering alone is also insufficient to stimulate VLA-4 integrin adhesiveness at these short-lived contacts (Fig. 6). |I:\*\*1SN3E3| |L:\*\*1SN3E3| |T:\*\*1SN3E2| |
|  |  |  |  | Annotation |  | 1SN3E0 1SN3E3 | 1SN3E3 1SN3E3 |  |  |  | 1SN3E0 1SN3E2 |  |
|  |  |  |  | Evidence |  | 1 4 | 4 4 |  |  |  | 1 3 |  |
|  |  |  |  | Focus |  | 4 4 | 4 4 |  |  |  | 4 4 |  |
|  |  |  |  | Polarity |  | 1 1 | 1 1 |  |  |  | 1 1 |  |
| 699 | 699 | 10692408\_8 | One proposed role for the cyclin E/CDK2 complex is the phosphorylation of MCM proteins, |I:\*\*1SP3E2| |L:\*\*1SP1E1| |T:\*\*1GP3E0| but precisely how it regulates MCMs and perhaps additional targets during initiation of replication is unknown ( ). |I:\*\*2SN3E2| |L:\*\*2SP0E0| |T:\*\*2GP3E2| |
|  |  |  |  | Annotation |  | 1SP3E2 2SN3E2 | 1SP1E1 2SP0E0 |  |  |  | 1GP3E0 2GP3E2 |  |
|  |  |  |  | Evidence |  | 3 3 | 2 1 |  |  |  | 1 3 |  |
|  |  |  |  | Focus |  | 4 4 | 4 4 |  |  |  | 1 1 |  |
|  |  |  |  | Polarity |  | 7 1 | 5 4 |  |  |  | 7 7 |  |
| 700 | 700 | 9462517\_7 | Nuclear factor of activated T cells (NF-AT) was first described |I:\*\*1SGP3E2| as a transcriptional regulatory complex critical for the expression of the T cell cytokine interleukin-2 (IL-2) ( Shaw et al. 1988 ). |I:\*\*2SP3E2| |L:\*\*1SP3E2| |T:\*\*1GP3E2| |
|  |  |  |  | Annotation |  | 1SGP3E2 2SP3E2 | 1SP3E2 1SP3E2 |  |  |  | 1GP3E2 1GP3E2 |  |
|  |  |  |  | Evidence |  | 3 3 | 3 3 |  |  |  | 3 3 |  |
|  |  |  |  | Focus |  | 5 4 | 4 4 |  |  |  | 1 1 |  |
|  |  |  |  | Polarity |  | 7 7 | 7 7 |  |  |  | 7 7 |  |
| 701 | 701 | 10966427\_115 | Since there was very little or no acid formed in the mutant cultures, |I:\*\*1SP3E0| |T:\*\*1MP3E3| the lower growth rate of the mutant cannot be attributed to the inhibitory role of acidic by-products, such as acetate. |I:\*\*2SN3E0| |L:\*\*1SN3E3| |T:\*\*2SN3E3| |
|  |  |  |  | Annotation |  | 1SP3E0 2SN3E0 | 1SN3E3 1SN3E3 |  |  |  | 1MP3E3 2SN3E3 |  |
|  |  |  |  | Evidence |  | 1 1 | 4 4 |  |  |  | 4 4 |  |
|  |  |  |  | Focus |  | 4 4 | 4 4 |  |  |  | 2 4 |  |
|  |  |  |  | Polarity |  | 7 1 | 1 1 |  |  |  | 7 1 |  |
| 702 | 702 | 12444082\_49 | View larger version (24K): [in this window] [in a new window] |I:\*\*ERROR| |L:\*\*ERROR| |T:\*\*ERROR| Fig. 2. Influence of the ethylene glycol concentration on the hydrolysis of the synthetic substrates MOC-D-Nle-Gly-Arg-pNA ( A) and MS-D-Phe-Gly-Arg-pNA ( B) by several rf9a ( a) and rf10a ( b) variants. |I:\*\*1SGP3E3| |L:\*\*1GSP3E3| |T:\*\*1SP3E0| |
|  |  |  |  | Annotation |  | ERROR 1SGP3E3 | ERROR 1GSP3E3 |  |  |  | ERROR 1SP3E0 |  |
|  |  |  |  | Evidence |  | -1000 4 | -1000 4 |  |  |  | -1000 1 |  |
|  |  |  |  | Focus |  | -1000 5 | -1000 5 |  |  |  | -1000 4 |  |
|  |  |  |  | Polarity |  | -1000 7 | -1000 7 |  |  |  | -1000 7 |  |
| 703 | 703 | 9774654\_284 | Alternatively, other granzymes |I:\*\*1SP3E0| not inhibited by PI-9 |I:\*\*2SN3E0| may have contributed to target cell death in these systems. |I:\*\*3SP1E0| |L:\*\*1SP1E0| |T:\*\*1SP3E0| |
|  |  |  |  | Annotation |  | 1SP3E0 2SN3E0 3SP1E0 | 1SP1E0 1SP1E0 1SP1E0 |  |  |  | 1SP3E0 1SP3E0 1SP3E0 |  |
|  |  |  |  | Evidence |  | 1 1 1 | 1 1 1 |  |  |  | 1 1 1 |  |
|  |  |  |  | Focus |  | 4 4 4 | 4 4 4 |  |  |  | 4 4 4 |  |
|  |  |  |  | Polarity |  | 7 1 5 | 5 5 5 |  |  |  | 7 7 7 |  |
| 704 | 704 | 7673207\_27 | No competition was observed between labeled F/C11 and unlabeled B/D6. |I:\*\*1SN3E0| |L:\*\*1SN3E3| |T:\*\*1SP3E3| |
|  |  |  |  | Annotation |  | 1SN3E0 | 1SN3E3 |  |  |  | 1SP3E3 |  |
|  |  |  |  | Evidence |  | 1 | 4 |  |  |  | 4 |  |
|  |  |  |  | Focus |  | 4 | 4 |  |  |  | 4 |  |
|  |  |  |  | Polarity |  | 1 | 1 |  |  |  | 7 |  |
| 705 | 705 | 12059934\_10 | CONCLUSIONS: Post removal with the Eggler device is a relatively safe procedure |T:\*\*1GP3E0| but care must be taken when there is a possibility of pulling the post out in a nonaxial direction or when less than 1 mm of dentine surrounds the apical end of the post. |T:\*\*2GP2E0| |I:\*\*1SP3E3| |L:\*\*1GSP3E3| |
|  |  |  |  | Annotation |  | 1SP3E3 1SP3E3 | 1GSP3E3 1GSP3E3 |  |  |  | 1GP3E0 2GP2E0 |  |
|  |  |  |  | Evidence |  | 4 4 | 4 4 |  |  |  | 1 1 |  |
|  |  |  |  | Focus |  | 4 4 | 5 5 |  |  |  | 1 1 |  |
|  |  |  |  | Polarity |  | 7 7 | 7 7 |  |  |  | 7 6 |  |
| 706 | 706 | 11713294\_323 | However, we found essentially identical BCR-Dok colocalization |I:\*\*1SP3E3| when B cells were stimulated with intact or F(ab'')2 fragments of anti-Ig. |I:\*\*2SGP3E3| |L:\*\*1SP3E3| |T:\*\*1SP3E3| |
|  |  |  |  | Annotation |  | 1SP3E3 2SGP3E3 | 1SP3E3 1SP3E3 |  |  |  | 1SP3E3 1SP3E3 |  |
|  |  |  |  | Evidence |  | 4 4 | 4 4 |  |  |  | 4 4 |  |
|  |  |  |  | Focus |  | 4 5 | 4 4 |  |  |  | 4 4 |  |
|  |  |  |  | Polarity |  | 7 7 | 7 7 |  |  |  | 7 7 |  |
| 707 | 707 | 9808625\_222 | The developmental stages in which small size and developmental delay first occur correlate with the increase in apoptosis (cf. Figs. 5, C-H, and 6A). |I:\*\*1SP3E3| |L:\*\*1SP3E3| |T:\*\*1SP3E3+| |
|  |  |  |  | Annotation |  | 1SP3E3 | 1SP3E3 |  |  |  | 1SP3E3+ |  |
|  |  |  |  | Evidence |  | 4 | 4 |  |  |  | 4 |  |
|  |  |  |  | Focus |  | 4 | 4 |  |  |  | 4 |  |
|  |  |  |  | Polarity |  | 7 | 7 |  |  |  | 7 |  |
| 708 | 708 | 9802902\_96 | Transiently transfected cells were double-labeled with the monoclonal antibody 9E10 to detect myc-ARNO and with rhodamine-phalloidin to label F-actin. |I:\*\*1MP3E0| |L:\*\*1MP3E3| |T:\*\*1MP3E3| |
|  |  |  |  | Annotation |  | 1MP3E0 | 1MP3E3 |  |  |  | 1MP3E3 |  |
|  |  |  |  | Evidence |  | 1 | 4 |  |  |  | 4 |  |
|  |  |  |  | Focus |  | 2 | 2 |  |  |  | 2 |  |
|  |  |  |  | Polarity |  | 7 | 7 |  |  |  | 7 |  |
| 709 | 709 | 9390512\_238 | As noted above, schizophrenia and other psychiatric disorders have been reported in adults with velo-cardio-facial syndrome (VCFS). |I:\*\*1SP3E0| |L:\*\*1SP3E1| |T:\*\*1GP3E0| |
|  |  |  |  | Annotation |  | 1SP3E0 | 1SP3E1 |  |  |  | 1GP3E0 |  |
|  |  |  |  | Evidence |  | 1 | 2 |  |  |  | 1 |  |
|  |  |  |  | Focus |  | 4 | 4 |  |  |  | 1 |  |
|  |  |  |  | Polarity |  | 7 | 7 |  |  |  | 7 |  |
| 710 | 710 | 10224161\_237 | When cholesterol was added onto the DPPC film in the presence of SP-Ahyp,deltaN1-A7, |I:\*\*1SGP3E3| |T:\*\*1MP3E3| this protein could not prevent the detrimental effect of cholesterol on the surface activity of DPPC ( Figure 10 and Table 1, row 14). |I:\*\*2SN3E3| |L:\*\*1SN3E3| |T:\*\*2SN3E3| |
|  |  |  |  | Annotation |  | 1SGP3E3 2SN3E3 | 1SN3E3 1SN3E3 |  |  |  | 1MP3E3 2SN3E3 |  |
|  |  |  |  | Evidence |  | 4 4 | 4 4 |  |  |  | 4 4 |  |
|  |  |  |  | Focus |  | 5 4 | 4 4 |  |  |  | 2 4 |  |
|  |  |  |  | Polarity |  | 7 1 | 1 1 |  |  |  | 7 1 |  |
| 711 | 711 | 10383463\_24 | Wnt interacts with frizzled to induce axis duplication following coinjection of early Xenopus embryos ( 17). |I:\*\*1SP3E2| |L:\*\*1SP3E2| |T:\*\*1SP3E3+| |
|  |  |  |  | Annotation |  | 1SP3E2 | 1SP3E2 |  |  |  | 1SP3E3+ |  |
|  |  |  |  | Evidence |  | 3 | 3 |  |  |  | 4 |  |
|  |  |  |  | Focus |  | 4 | 4 |  |  |  | 4 |  |
|  |  |  |  | Polarity |  | 7 | 7 |  |  |  | 7 |  |
| 712 | 712 | 11777924\_20 | In the case of the P426L-ASA, the degradation results in a rapid loss of catalytic activity and immunoreactive peptides, |I:\*\*1SP3E0-| |L:\*\*1SP3E0-| |T:\*\*1SN3E0| whereas in the case of the 464R-ASA a 54-kDa intermediate is generated, which is catalytically active. |I:\*\*2SP3E0+| |L:\*\*2SP3E0+| |T:\*\*2SP3E0| |
|  |  |  |  | Annotation |  | 1SP3E0- 2SP3E0+ | 1SP3E0- 2SP3E0+ |  |  |  | 1SN3E0 2SP3E0 |  |
|  |  |  |  | Evidence |  | 1 1 | 1 1 |  |  |  | 1 1 |  |
|  |  |  |  | Focus |  | 4 4 | 4 4 |  |  |  | 4 4 |  |
|  |  |  |  | Polarity |  | 7 7 | 7 7 |  |  |  | 1 7 |  |
| 713 | 713 | 11483581\_17 | In the present study, we examined the conditions for identifying the genome of origin for a specific genomic sequence, |I:\*\*1SGP0E3| using the genomic signature concept. |I:\*\*2MP3E3| |L:\*\*1GMP3E3| |T:\*\*1GMP3E3| |
|  |  |  |  | Annotation |  | 1SGP0E3 2MP3E3 | 1GMP3E3 1GMP3E3 |  |  |  | 1GMP3E3 1GMP3E3 |  |
|  |  |  |  | Evidence |  | 4 4 | 4 4 |  |  |  | 4 4 |  |
|  |  |  |  | Focus |  | 5 2 | 3 3 |  |  |  | 3 3 |  |
|  |  |  |  | Polarity |  | 4 7 | 7 7 |  |  |  | 7 7 |  |
| 714 | 714 | 9145910\_81 | Internalization was performed using 125I-[Tyr4]bombesin as described previously ( 7). |I:\*\*1MP3E3| |L:\*\*1MP3E23| |T:\*\*1MP3E2| |
|  |  |  |  | Annotation |  | 1MP3E3 | 1MP3E23 |  |  |  | 1MP3E2 |  |
|  |  |  |  | Evidence |  | 4 | 3 |  |  |  | 3 |  |
|  |  |  |  | Focus |  | 2 | 2 |  |  |  | 2 |  |
|  |  |  |  | Polarity |  | 7 | 7 |  |  |  | 7 |  |
| 715 | 715 | 7721780\_36 | NHP6A/B Form More Stable Complexes with DNA Fragments than HU or HMG1 |I:\*\*ERROR| |L:\*\*1SP3E0| |T:\*\*1SP3E0| To investigate the relative stability of NHP6A/B DNA complexes in comparison to those containing HU or HMG1, |I:\*\*1SGP0E0| each of these proteins was added to a 46-bp P-labeled double-stranded DNA and run in a nondenaturing polyacrylamide gel to separate the protein-bound DNA from the free DNA fragments. |I:\*\*2MP3E0| |L:\*\*1MSP3E3| |T:\*\*1MP3E3| |
|  |  |  |  | Annotation |  | ERROR 1SGP0E0 2MP3E0 | 1SP3E0 1MSP3E3 1MSP3E3 |  |  |  | 1SP3E0 1MP3E3 1MP3E3 |  |
|  |  |  |  | Evidence |  | -1000 1 1 | 1 4 4 |  |  |  | 1 4 4 |  |
|  |  |  |  | Focus |  | -1000 5 2 | 4 6 6 |  |  |  | 4 2 2 |  |
|  |  |  |  | Polarity |  | -1000 4 7 | 7 7 7 |  |  |  | 7 7 7 |  |
| 716 | 716 | 9687574\_74 | Ligand binding was measured as described previously (Houghtling et al., 1995 ) with minor modifications. |I:\*\*1MP3E3| |L:\*\*1MP3E23| |T:\*\*1MP3E2| |
|  |  |  |  | Annotation |  | 1MP3E3 | 1MP3E23 |  |  |  | 1MP3E2 |  |
|  |  |  |  | Evidence |  | 4 | 3 |  |  |  | 3 |  |
|  |  |  |  | Focus |  | 2 | 2 |  |  |  | 2 |  |
|  |  |  |  | Polarity |  | 7 | 7 |  |  |  | 7 |  |
| 717 | 717 | 8810344\_3 | Intracellular FVIII WT was detected in its single chain form and migrated at approximately 280 kDa (Fig. 1, lane 2). |I:\*\*1SP3E3| |L:\*\*1SP3E3| |T:\*\*1SP3E2| |
|  |  |  |  | Annotation |  | 1SP3E3 | 1SP3E3 |  |  |  | 1SP3E2 |  |
|  |  |  |  | Evidence |  | 4 | 4 |  |  |  | 3 |  |
|  |  |  |  | Focus |  | 4 | 4 |  |  |  | 4 |  |
|  |  |  |  | Polarity |  | 7 | 7 |  |  |  | 7 |  |
| 718 | 718 | 9052839\_193 | Interestingly, a short transcript that possibly corresponds to the soluble receptor TRAMP is highly up-regulated in mice that lack a functional Fas. |I:\*\*1SP3E0+| |L:\*\*1SP3E3+| |T:\*\*1SP3E0+| |
|  |  |  |  | Annotation |  | 1SP3E0+ | 1SP3E3+ |  |  |  | 1SP3E0+ |  |
|  |  |  |  | Evidence |  | 1 | 4 |  |  |  | 1 |  |
|  |  |  |  | Focus |  | 4 | 4 |  |  |  | 4 |  |
|  |  |  |  | Polarity |  | 7 | 7 |  |  |  | 7 |  |
| 719 | 719 | 10934279\_20 | Although it is well known that striatal NMDA and dopamine receptors play a key role in motor control (Hauber, 1996 , 1998 ), |I:\*\*1SP3E2| |L:\*\*1SP3E2| |T:\*\*1GP3E2| the NAc does not control motor aspects of RT performance per se (Amalric and Koob, 1987 ; Brown and Robbins, 1989 ; Carli et al., 1989 ). |I:\*\*2SN3E2| |L:\*\*2SN3E2| |T:\*\*2GN3E2| |
|  |  |  |  | Annotation |  | 1SP3E2 2SN3E2 | 1SP3E2 2SN3E2 |  |  |  | 1GP3E2 2GN3E2 |  |
|  |  |  |  | Evidence |  | 3 3 | 3 3 |  |  |  | 3 3 |  |
|  |  |  |  | Focus |  | 4 4 | 4 4 |  |  |  | 1 1 |  |
|  |  |  |  | Polarity |  | 7 1 | 7 1 |  |  |  | 7 1 |  |
| 720 | 720 | 10764813\_31 | In addition, mice with IRS-2 gene knockouts show defects in islet development |T:\*\*1SN3E2| ( ) |I:\*\*1SP3E2| and IRS-2 in beta-cells may mediate IGF-1 receptor effects on beta-cell development and peripheral insulin signaling ( ). |I:\*\*2SP1E2| |L:\*\*1SP3E0| |T:\*\*2SP3E2| |
|  |  |  |  | Annotation |  | 1SP3E2 1SP3E2 2SP1E2 | 1SP3E0 1SP3E0 1SP3E0 |  |  |  | 1SN3E2 2SP3E2 2SP3E2 |  |
|  |  |  |  | Evidence |  | 3 3 3 | 1 1 1 |  |  |  | 3 3 3 |  |
|  |  |  |  | Focus |  | 4 4 4 | 4 4 4 |  |  |  | 4 4 4 |  |
|  |  |  |  | Polarity |  | 7 7 5 | 7 7 7 |  |  |  | 1 7 7 |  |
| 721 | 721 | 10473520\_3 | We developed a new electrochemiluminescence immunoassay (ECLIA) for the antibody to BDV |I:\*\*1SGP3E3| that uses two recombinant proteins of BDV, p40 and p24 (full length). |I:\*\*2MP3E3| |L:\*\*1MP3E3| |T:\*\*1MP3E3| |
|  |  |  |  | Annotation |  | 1SGP3E3 2MP3E3 | 1MP3E3 1MP3E3 |  |  |  | 1MP3E3 1MP3E3 |  |
|  |  |  |  | Evidence |  | 4 4 | 4 4 |  |  |  | 4 4 |  |
|  |  |  |  | Focus |  | 5 2 | 2 2 |  |  |  | 2 2 |  |
|  |  |  |  | Polarity |  | 7 7 | 7 7 |  |  |  | 7 7 |  |
| 722 | 722 | 10207091\_166 | Putative RAG-induced structural perturbations in substrate DNA, |T:\*\*1SP3E0| as defined by overrepresentation of KMnO4 modification, are indicated by diamonds. |T:\*\*2MP3E0| |I:\*\*1SP3E0| |L:\*\*1GP3E3| |
|  |  |  |  | Annotation |  | 1SP3E0 1SP3E0 | 1GP3E3 1GP3E3 |  |  |  | 1SP3E0 2MP3E0 |  |
|  |  |  |  | Evidence |  | 1 1 | 4 4 |  |  |  | 1 1 |  |
|  |  |  |  | Focus |  | 4 4 | 1 1 |  |  |  | 4 2 |  |
|  |  |  |  | Polarity |  | 7 7 | 7 7 |  |  |  | 7 7 |  |
| 723 | 723 | 9150141\_362 | We would also like to thank Drs. J. A. Steitz, A. Horwich, M. Solomon, D. G. Schatz, M. Schlissel, D. Sengupta, and members of the laboratory for suggestions and for carefully reading the manuscript. |I:\*\*1GP3E3| |L:\*\*1GP3E3| |T:\*\*1GP3E3| |
|  |  |  |  | Annotation |  | 1GP3E3 | 1GP3E3 |  |  |  | 1GP3E3 |  |
|  |  |  |  | Evidence |  | 4 | 4 |  |  |  | 4 |  |
|  |  |  |  | Focus |  | 1 | 1 |  |  |  | 1 |  |
|  |  |  |  | Polarity |  | 7 | 7 |  |  |  | 7 |  |
| 724 | 724 | 10684251\_273 | This is consistent with a model in which overexpressed kinase-deficient Xgsk-3 acts as a dominant- negative either by displacing endogenous Xgsk-3 already bound to Axin or by preventing endogenous Xgsk-3 from binding Axin as new complexes form. |I:\*\*1SP3E0| |L:\*\*1SP2E0| |T:\*\*1SN3E0-| |
|  |  |  |  | Annotation |  | 1SP3E0 | 1SP2E0 |  |  |  | 1SN3E0- |  |
|  |  |  |  | Evidence |  | 1 | 1 |  |  |  | 1 |  |
|  |  |  |  | Focus |  | 4 | 4 |  |  |  | 4 |  |
|  |  |  |  | Polarity |  | 7 | 6 |  |  |  | 1 |  |
| 725 | 725 | 9430635\_85 | When [Ca2+]Lu had risen to a level close to the pre-stimulation state, thapsigargin was added. |I:\*\*1MP3E0| |L:\*\*1MP3E3| |T:\*\*1MP3E3| |
|  |  |  |  | Annotation |  | 1MP3E0 | 1MP3E3 |  |  |  | 1MP3E3 |  |
|  |  |  |  | Evidence |  | 1 | 4 |  |  |  | 4 |  |
|  |  |  |  | Focus |  | 2 | 2 |  |  |  | 2 |  |
|  |  |  |  | Polarity |  | 7 | 7 |  |  |  | 7 |  |
| 726 | 726 | 11404325\_249 | To map the 2.2-kb RNA fragment |I:\*\*1SGP3E3| we performed different hybridizations using as DNA probes the 366- and 244-bp DraI fragments of HIS3 and the 222-bp HindIII- SspI, 561-bp PstI- SspI, 1166-bp DraI- PvuII, and 641-bp AvaI- PvuII fragments of pBR322 (data not shown). |I:\*\*2MP3E3| |L:\*\*1MP3E13| |T:\*\*1MP3E1| |
|  |  |  |  | Annotation |  | 1SGP3E3 2MP3E3 | 1MP3E13 1MP3E13 |  |  |  | 1MP3E1 1MP3E1 |  |
|  |  |  |  | Evidence |  | 4 4 | 2 2 |  |  |  | 2 2 |  |
|  |  |  |  | Focus |  | 5 2 | 2 2 |  |  |  | 2 2 |  |
|  |  |  |  | Polarity |  | 7 7 | 7 7 |  |  |  | 7 7 |  |
| 727 | 727 | 12086620\_221 | Mutation of two C helix residues, Val 194 and Val 198 (Ile 85 and Leu 89 of PKA), reduced PIFtide activation to only 25% of wild-type, |L:\*\*1SP3E3| |T:\*\*1SP3E0-| whereas a Leu 225 mutant of the -5 strand (Leu 116 of PKA) caused almost a complete loss of responsiveness to PIFtide ( Figures 2, 5 , and 7B). |L:\*\*2SP3E3-| |I:\*\*1SP3E3-| |T:\*\*2SN3E3| |
|  |  |  |  | Annotation |  | 1SP3E3- 1SP3E3- | 1SP3E3 2SP3E3- |  |  |  | 1SP3E0- 2SN3E3 |  |
|  |  |  |  | Evidence |  | 4 4 | 4 4 |  |  |  | 1 4 |  |
|  |  |  |  | Focus |  | 4 4 | 4 4 |  |  |  | 4 4 |  |
|  |  |  |  | Polarity |  | 7 7 | 7 7 |  |  |  | 7 1 |  |
| 728 | 728 | 10713180\_75 | If required, membranes were stripped as described ( 40). |I:\*\*1MP3E2| |L:\*\*1MP3E2| |T:\*\*1MP3E2| |
|  |  |  |  | Annotation |  | 1MP3E2 | 1MP3E2 |  |  |  | 1MP3E2 |  |
|  |  |  |  | Evidence |  | 3 | 3 |  |  |  | 3 |  |
|  |  |  |  | Focus |  | 2 | 2 |  |  |  | 2 |  |
|  |  |  |  | Polarity |  | 7 | 7 |  |  |  | 7 |  |
| 729 | 729 | 9729526\_61 | The plates were then washed, and 100 mul of test serum diluted 1:100 in PBS-BSA was added to each well and incubated for 2 h at 37 degrees C, after which a fixed amount of IgA was captured per well. |I:\*\*1MP3E0| |L:\*\*1MP3E3| |T:\*\*1MP3E3| |
|  |  |  |  | Annotation |  | 1MP3E0 | 1MP3E3 |  |  |  | 1MP3E3 |  |
|  |  |  |  | Evidence |  | 1 | 4 |  |  |  | 4 |  |
|  |  |  |  | Focus |  | 2 | 2 |  |  |  | 2 |  |
|  |  |  |  | Polarity |  | 7 | 7 |  |  |  | 7 |  |
| 730 | 730 | 8797824\_125 | Collectively, these results indicate that Par-4 binds to PKC in vitro and in vivo and |T:\*\*1SP3E0| that this association leads to a dramatic inhibition of the kinase activity. |T:\*\*2SN3E0| |I:\*\*1SP3E0-| |L:\*\*1SP1E0| |
|  |  |  |  | Annotation |  | 1SP3E0- 1SP3E0- | 1SP1E0 1SP1E0 |  |  |  | 1SP3E0 2SN3E0 |  |
|  |  |  |  | Evidence |  | 1 1 | 1 1 |  |  |  | 1 1 |  |
|  |  |  |  | Focus |  | 4 4 | 4 4 |  |  |  | 4 4 |  |
|  |  |  |  | Polarity |  | 7 7 | 5 5 |  |  |  | 7 1 |  |
| 731 | 731 | 12718890\_73 | An analysis of the domains in SPT5 that interact with either PRMT5 or PRMT1 was performed |I:\*\*1SGP3E3| by transfecting expression vectors encoding either Myc-tagged PRMT5 or Myc-tagged PRMT1 and the indicated Flag-tagged SPT5 constructs into 293 cells (left panel). |I:\*\*2MP3E3| |L:\*\*1MP3E3| |T:\*\*1MP3E3| |
|  |  |  |  | Annotation |  | 1SGP3E3 2MP3E3 | 1MP3E3 1MP3E3 |  |  |  | 1MP3E3 1MP3E3 |  |
|  |  |  |  | Evidence |  | 4 4 | 4 4 |  |  |  | 4 4 |  |
|  |  |  |  | Focus |  | 5 2 | 2 2 |  |  |  | 2 2 |  |
|  |  |  |  | Polarity |  | 7 7 | 7 7 |  |  |  | 7 7 |  |
| 732 | 732 | 10721990\_24 | Binding of WNT to its seven pass transmembrane receptor protein, Frizzled (Fz), leads to activation of Disheveled, |T:\*\*1SP3E0+| |L:\*\*1SP3E0+| resulting in the inhibition of a serine/threonine kinase, GSK-3beta, |T:\*\*2SN3E0| |I:\*\*1SP3E0| and in the subsequent accumulation and nuclear translocation of beta-catenin. |T:\*\*3SP3E0| |I:\*\*2SP3E0+| |L:\*\*2SP3E0-| |
|  |  |  |  | Annotation |  | 1SP3E0 1SP3E0 2SP3E0+ | 1SP3E0+ 2SP3E0- 2SP3E0- |  |  |  | 1SP3E0+ 2SN3E0 3SP3E0 |  |
|  |  |  |  | Evidence |  | 1 1 1 | 1 1 1 |  |  |  | 1 1 1 |  |
|  |  |  |  | Focus |  | 4 4 4 | 4 4 4 |  |  |  | 4 4 4 |  |
|  |  |  |  | Polarity |  | 7 7 7 | 7 7 7 |  |  |  | 7 1 7 |  |
| 733 | 733 | 12354395\_204 | The WNT Antagonist sFRP1 Blocks the Effect of WNT-3 on Axonal Remodeling |I:\*\*ERROR| |L:\*\*1SP3E0| |T:\*\*1SP3E0| |
|  |  |  |  | Annotation |  | ERROR | 1SP3E0 |  |  |  | 1SP3E0 |  |
|  |  |  |  | Evidence |  | -1000 | 1 |  |  |  | 1 |  |
|  |  |  |  | Focus |  | -1000 | 4 |  |  |  | 4 |  |
|  |  |  |  | Polarity |  | -1000 | 7 |  |  |  | 7 |  |
| 734 | 734 | 10790380\_209 | We thank colleagues who generously contributed strains. |I:\*\*1GP3E3| |L:\*\*1GP3E3| |T:\*\*1GP3E0| |
|  |  |  |  | Annotation |  | 1GP3E3 | 1GP3E3 |  |  |  | 1GP3E0 |  |
|  |  |  |  | Evidence |  | 4 | 4 |  |  |  | 1 |  |
|  |  |  |  | Focus |  | 1 | 1 |  |  |  | 1 |  |
|  |  |  |  | Polarity |  | 7 | 7 |  |  |  | 7 |  |
| 735 | 735 | 12628171\_106 | The expression of GRID2Lc in PCs causes a massive degeneration of these neurons accompanied by target-related death of PC afferents during the first postnatal month in Grid2Lc/Grid2+ mice (Caddy and Biscoe, 1979 ; Dumesnil-Bousez and Sotelo, 1992 ). |I:\*\*1SP3E2| |L:\*\*1SP3E2-| |T:\*\*1SN3E2| |
|  |  |  |  | Annotation |  | 1SP3E2 | 1SP3E2- |  |  |  | 1SN3E2 |  |
|  |  |  |  | Evidence |  | 3 | 3 |  |  |  | 3 |  |
|  |  |  |  | Focus |  | 4 | 4 |  |  |  | 4 |  |
|  |  |  |  | Polarity |  | 7 | 7 |  |  |  | 1 |  |
| 736 | 736 | 12011094\_56 | B, S1 nuclease mapping experiment carried out |I:\*\*1SGP3E0| to discriminate between these two hypotheses. |I:\*\*2SP0E0| |L:\*\*1GP3E3| |T:\*\*1MP3E0| |
|  |  |  |  | Annotation |  | 1SGP3E0 2SP0E0 | 1GP3E3 1GP3E3 |  |  |  | 1MP3E0 1MP3E0 |  |
|  |  |  |  | Evidence |  | 1 1 | 4 4 |  |  |  | 1 1 |  |
|  |  |  |  | Focus |  | 5 4 | 1 1 |  |  |  | 2 2 |  |
|  |  |  |  | Polarity |  | 7 4 | 7 7 |  |  |  | 7 7 |  |
| 737 | 737 | 9755191\_309 | It is not known if ARF provides a binding site for coat assembly on a membrane or |T:\*\*1SP0E0| if ARF alters the membrane in a manner that allows efficient coat assembly. |T:\*\*2SN0E0| |I:\*\*1SN3E0| |L:\*\*1SP0E0| |
|  |  |  |  | Annotation |  | 1SN3E0 1SN3E0 | 1SP0E0 1SP0E0 |  |  |  | 1SP0E0 2SN0E0 |  |
|  |  |  |  | Evidence |  | 1 1 | 1 1 |  |  |  | 1 1 |  |
|  |  |  |  | Focus |  | 4 4 | 4 4 |  |  |  | 4 4 |  |
|  |  |  |  | Polarity |  | 1 1 | 4 4 |  |  |  | 4 4 |  |
| 738 | 738 | 9750191\_176 | There is still a deviation from the binomial distribution, only with a lesser degree. |I:\*\*1SP3E0| |L:\*\*1SP3E3| |T:\*\*1SP3E0-| |
|  |  |  |  | Annotation |  | 1SP3E0 | 1SP3E3 |  |  |  | 1SP3E0- |  |
|  |  |  |  | Evidence |  | 1 | 4 |  |  |  | 1 |  |
|  |  |  |  | Focus |  | 4 | 4 |  |  |  | 4 |  |
|  |  |  |  | Polarity |  | 7 | 7 |  |  |  | 7 |  |
| 739 | 739 | 12052268\_5 | Several studies suggest that the positive effects of statins on plaque stabilization, inflammation, thrombosis, and endothelial function may be independent of lipid levels. |I:\*\*1SP1E1| |L:\*\*1SP1E1| |T:\*\*1SP2E1+| |
|  |  |  |  | Annotation |  | 1SP1E1 | 1SP1E1 |  |  |  | 1SP2E1+ |  |
|  |  |  |  | Evidence |  | 2 | 2 |  |  |  | 2 |  |
|  |  |  |  | Focus |  | 4 | 4 |  |  |  | 4 |  |
|  |  |  |  | Polarity |  | 5 | 5 |  |  |  | 6 |  |
| 740 | 740 | 9722623\_319 | Alternatively, they may have a mechanism for sequestering the zymogen in a subcellular compartment in which it is protected from activation. |I:\*\*1SP1E0| |L:\*\*1GSP1E0| |T:\*\*1SP2E0| |
|  |  |  |  | Annotation |  | 1SP1E0 | 1GSP1E0 |  |  |  | 1SP2E0 |  |
|  |  |  |  | Evidence |  | 1 | 1 |  |  |  | 1 |  |
|  |  |  |  | Focus |  | 4 | 5 |  |  |  | 4 |  |
|  |  |  |  | Polarity |  | 5 | 5 |  |  |  | 6 |  |
| 741 | 741 | 8626459\_25 | First, thiophosphorylated precursor proteins were used for import. |I:\*\*1MP3E0| |L:\*\*1SP3E0| |T:\*\*1MP3E0| |
|  |  |  |  | Annotation |  | 1MP3E0 | 1SP3E0 |  |  |  | 1MP3E0 |  |
|  |  |  |  | Evidence |  | 1 | 1 |  |  |  | 1 |  |
|  |  |  |  | Focus |  | 2 | 4 |  |  |  | 2 |  |
|  |  |  |  | Polarity |  | 7 | 7 |  |  |  | 7 |  |
| 742 | 742 | 11160081\_59 | The resulting PCR products were individually cloned into pGEM7 |I:\*\*1SGP3E0| using XhoI and NsiI |I:\*\*2MP3E0| to yield plasmids pEA4 and pVALGOX, respectively. |I:\*\*3SGP3E0| |L:\*\*1MP3E3| |T:\*\*1MP3E3| |
|  |  |  |  | Annotation |  | 1SGP3E0 2MP3E0 3SGP3E0 | 1MP3E3 1MP3E3 1MP3E3 |  |  |  | 1MP3E3 1MP3E3 1MP3E3 |  |
|  |  |  |  | Evidence |  | 1 1 1 | 4 4 4 |  |  |  | 4 4 4 |  |
|  |  |  |  | Focus |  | 5 2 5 | 2 2 2 |  |  |  | 2 2 2 |  |
|  |  |  |  | Polarity |  | 7 7 7 | 7 7 7 |  |  |  | 7 7 7 |  |
| 743 | 743 | 12140158\_2 | We report such a case |I:\*\*1SGP3E3| in which both upper gastrointestinal series and abdominal computerized tomography (CT) |I:\*\*2MP3E3| demonstrated an antral mass; |I:\*\*3SP3E3| surgical and histological results are also reported. |I:\*\*4SGP3E3| |L:\*\*1GSP3E3| |T:\*\*1GP3E0| |
|  |  |  |  | Annotation |  | 1SGP3E3 2MP3E3 3SP3E3 4SGP3E3 | 1GSP3E3 1GSP3E3 1GSP3E3 1GSP3E3 |  |  |  | 1GP3E0 1GP3E0 1GP3E0 1GP3E0 |  |
|  |  |  |  | Evidence |  | 4 4 4 4 | 4 4 4 4 |  |  |  | 1 1 1 1 |  |
|  |  |  |  | Focus |  | 5 2 4 5 | 5 5 5 5 |  |  |  | 1 1 1 1 |  |
|  |  |  |  | Polarity |  | 7 7 7 7 | 7 7 7 7 |  |  |  | 7 7 7 7 |  |
| 744 | 744 | 11408566\_27 | However, the drug did not induce catalepsy nor did it influence the apomorphine- or the amphetamine-induced stereotypy (Bruhwyler et al., 1997 ). |I:\*\*1SN3E2| |L:\*\*1SN3E2| |T:\*\*1SN3E2| |
|  |  |  |  | Annotation |  | 1SN3E2 | 1SN3E2 |  |  |  | 1SN3E2 |  |
|  |  |  |  | Evidence |  | 3 | 3 |  |  |  | 3 |  |
|  |  |  |  | Focus |  | 4 | 4 |  |  |  | 4 |  |
|  |  |  |  | Polarity |  | 1 | 1 |  |  |  | 1 |  |
| 745 | 745 | 11159738\_7 | Taken together, our results suggest that DCA can stimulate pro-apoptotic and anti-apoptotic signaling pathways and that sensitivity to DCA-induced apoptosis can be modulated by the ERK MAP kinase. |I:\*\*1SP1E3| |L:\*\*1SP1E3| |T:\*\*1SP3E0| |
|  |  |  |  | Annotation |  | 1SP1E3 | 1SP1E3 |  |  |  | 1SP3E0 |  |
|  |  |  |  | Evidence |  | 4 | 4 |  |  |  | 1 |  |
|  |  |  |  | Focus |  | 4 | 4 |  |  |  | 4 |  |
|  |  |  |  | Polarity |  | 5 | 5 |  |  |  | 7 |  |
| 746 | 746 | 12175867\_6 | Surprisingly, mlc/NT-3 mice also exhibited significant hyperactivity, |I:\*\*1SP3E0| |L:\*\*1SP3E3| |T:\*\*1SP3E0| suggesting that NT-3 overexpression in the periphery may have caused abnormalities in the CNS |I:\*\*2SP1E0+| that are related to the cortical processing of proprioceptive afferent information. |I:\*\*3SP1E0| |L:\*\*2SP1E3| |T:\*\*2SP2E0| |
|  |  |  |  | Annotation |  | 1SP3E0 2SP1E0+ 3SP1E0 | 1SP3E3 2SP1E3 2SP1E3 |  |  |  | 1SP3E0 2SP2E0 2SP2E0 |  |
|  |  |  |  | Evidence |  | 1 1 1 | 4 4 4 |  |  |  | 1 1 1 |  |
|  |  |  |  | Focus |  | 4 4 4 | 4 4 4 |  |  |  | 4 4 4 |  |
|  |  |  |  | Polarity |  | 7 5 5 | 7 5 5 |  |  |  | 7 6 6 |  |
| 747 | 747 | 10722605\_121 | The resistance cassette contains the omega fragment ( 23), |T:\*\*1GP3E2| |I:\*\*1SP3E2| which encodes resistance to spectinomycin due to aadA (aminoglycoside adenyltransferase) and |T:\*\*2GP3E0| interrupts both translation and transcription, flanked on each side in inverted orientation by the neisserial uptake sequence GCCGTCTGAA. |T:\*\*3GN3E0| |I:\*\*2SP3E0| |L:\*\*1SP3E2| |
|  |  |  |  | Annotation |  | 1SP3E2 2SP3E0 2SP3E0 | 1SP3E2 1SP3E2 1SP3E2 |  |  |  | 1GP3E2 2GP3E0 3GN3E0 |  |
|  |  |  |  | Evidence |  | 3 1 1 | 3 3 3 |  |  |  | 3 1 1 |  |
|  |  |  |  | Focus |  | 4 4 4 | 4 4 4 |  |  |  | 1 1 1 |  |
|  |  |  |  | Polarity |  | 7 7 7 | 7 7 7 |  |  |  | 7 7 1 |  |
| 748 | 748 | 11381111\_9 | These data suggest that the COMT Val allele, |I:\*\*1SP1E0| |L:\*\*1SP1E3| because it increases prefrontal dopamine catabolism, |I:\*\*2SP3E0+| |L:\*\*2SP3E3+| |T:\*\*1SP3E0| impairs prefrontal cognition and physiology, |I:\*\*3SP1E0| |L:\*\*3SP3E3-| and by this mechanism slightly increases risk for schizophrenia. |I:\*\*4SP1E0+| |L:\*\*4SP3E3+| |T:\*\*2SN3E0| |
|  |  |  |  | Annotation |  | 1SP1E0 2SP3E0+ 3SP1E0 4SP1E0+ | 1SP1E3 2SP3E3+ 3SP3E3- 4SP3E3+ |  |  |  | 1SP3E0 1SP3E0 2SN3E0 2SN3E0 |  |
|  |  |  |  | Evidence |  | 1 1 1 1 | 4 4 4 4 |  |  |  | 1 1 1 1 |  |
|  |  |  |  | Focus |  | 4 4 4 4 | 4 4 4 4 |  |  |  | 4 4 4 4 |  |
|  |  |  |  | Polarity |  | 5 7 5 5 | 5 7 7 7 |  |  |  | 7 7 1 1 |  |
| 749 | 749 | 9671482\_89 | Chloramphenicol acetyltransferase (CAT) activity was normalized for transfection efficiency to the corresponding beta-Gal activity. |I:\*\*1MP3E0| |L:\*\*1MP3E3| |T:\*\*1MP3E3| |
|  |  |  |  | Annotation |  | 1MP3E0 | 1MP3E3 |  |  |  | 1MP3E3 |  |
|  |  |  |  | Evidence |  | 1 | 4 |  |  |  | 4 |  |
|  |  |  |  | Focus |  | 2 | 2 |  |  |  | 2 |  |
|  |  |  |  | Polarity |  | 7 | 7 |  |  |  | 7 |  |
| 750 | 750 | 11879640\_222 | C. elegans strain N2 served as the wild-type strain (Brenner, 1974 ). |I:\*\*1MP3E2| |L:\*\*1MP3E23| |T:\*\*1GP3E2| |
|  |  |  |  | Annotation |  | 1MP3E2 | 1MP3E23 |  |  |  | 1GP3E2 |  |
|  |  |  |  | Evidence |  | 3 | 3 |  |  |  | 3 |  |
|  |  |  |  | Focus |  | 2 | 2 |  |  |  | 1 |  |
|  |  |  |  | Polarity |  | 7 | 7 |  |  |  | 7 |  |
| 751 | 751 | 9528762\_60 | Two carefully marked replicate filters were prepared |I:\*\*1SGP3E0| by direct transfer of bacterial colonies onto a second prewetted nitrocellulose filter. |I:\*\*2MP3E0| |L:\*\*1MP3E3| |T:\*\*1MP3E3| |
|  |  |  |  | Annotation |  | 1SGP3E0 2MP3E0 | 1MP3E3 1MP3E3 |  |  |  | 1MP3E3 1MP3E3 |  |
|  |  |  |  | Evidence |  | 1 1 | 4 4 |  |  |  | 4 4 |  |
|  |  |  |  | Focus |  | 5 2 | 2 2 |  |  |  | 2 2 |  |
|  |  |  |  | Polarity |  | 7 7 | 7 7 |  |  |  | 7 7 |  |
| 752 | 752 | 11926550\_2 | Despite of some structural differences both Fe(III) complexes are taken up by either strain with a high rate. |I:\*\*1SP3E0| |L:\*\*1SP3E3| |T:\*\*1MP3E3| |
|  |  |  |  | Annotation |  | 1SP3E0 | 1SP3E3 |  |  |  | 1MP3E3 |  |
|  |  |  |  | Evidence |  | 1 | 4 |  |  |  | 4 |  |
|  |  |  |  | Focus |  | 4 | 4 |  |  |  | 2 |  |
|  |  |  |  | Polarity |  | 7 | 7 |  |  |  | 7 |  |
| 753 | 753 | 11015381\_42 | B. burgdorferi isolate 50772 organisms lack ospA and ospB |L:\*\*1SP3E2| and consequently do not produce OspA or OspB ( 1). |L:\*\*2SN3E2| |I:\*\*1SN3E2| |T:\*\*1GP3E2| |
|  |  |  |  | Annotation |  | 1SN3E2 1SN3E2 | 1SP3E2 2SN3E2 |  |  |  | 1GP3E2 1GP3E2 |  |
|  |  |  |  | Evidence |  | 3 3 | 3 3 |  |  |  | 3 3 |  |
|  |  |  |  | Focus |  | 4 4 | 4 4 |  |  |  | 1 1 |  |
|  |  |  |  | Polarity |  | 1 1 | 7 1 |  |  |  | 7 7 |  |
| 754 | 754 | 12511568\_24 | In this work, we therefore examined whether catalase function was inhibited by ceramide at mRNA or protein synthesis levels in a caspase-3-dependent manner. |I:\*\*1SP0E3| |L:\*\*1GSP0E3| |T:\*\*1SN3E0| |
|  |  |  |  | Annotation |  | 1SP0E3 | 1GSP0E3 |  |  |  | 1SN3E0 |  |
|  |  |  |  | Evidence |  | 4 | 4 |  |  |  | 1 |  |
|  |  |  |  | Focus |  | 4 | 5 |  |  |  | 4 |  |
|  |  |  |  | Polarity |  | 4 | 4 |  |  |  | 1 |  |
| 755 | 755 | 12021257\_285 | In the experiments shown in Fig. 6 A, the maximum possible effect was 89.6%, the proportion of cells treated with BAF plus CsA that could be rescued after 10 d of NGF deprivation. |I:\*\*1SP3E3| |L:\*\*1SP3E3| |T:\*\*1SP3E3| |
|  |  |  |  | Annotation |  | 1SP3E3 | 1SP3E3 |  |  |  | 1SP3E3 |  |
|  |  |  |  | Evidence |  | 4 | 4 |  |  |  | 4 |  |
|  |  |  |  | Focus |  | 4 | 4 |  |  |  | 4 |  |
|  |  |  |  | Polarity |  | 7 | 7 |  |  |  | 7 |  |
| 756 | 756 | 11248093\_29 | We also show that DCC recruits caspase-3 and caspase-9, resulting in the activation of caspase-3 via caspase-9. |I:\*\*1SP3E3| |L:\*\*1SP3E3+| |T:\*\*1SP3E1| |
|  |  |  |  | Annotation |  | 1SP3E3 | 1SP3E3+ |  |  |  | 1SP3E1 |  |
|  |  |  |  | Evidence |  | 4 | 4 |  |  |  | 2 |  |
|  |  |  |  | Focus |  | 4 | 4 |  |  |  | 4 |  |
|  |  |  |  | Polarity |  | 7 | 7 |  |  |  | 7 |  |
| 757 | 757 | 9435194\_104 | The mGluR antagonist, ( RS)-MCPG, blocks the locomotor-activating effects of NAcc apomorphine (APO). |I:\*\*1SP3E0| |L:\*\*1SP3E0-| |T:\*\*1SP3E0| |
|  |  |  |  | Annotation |  | 1SP3E0 | 1SP3E0- |  |  |  | 1SP3E0 |  |
|  |  |  |  | Evidence |  | 1 | 1 |  |  |  | 1 |  |
|  |  |  |  | Focus |  | 4 | 4 |  |  |  | 4 |  |
|  |  |  |  | Polarity |  | 7 | 7 |  |  |  | 7 |  |
| 758 | 758 | 11156982\_139 | Prior to the emergence of the first hyphal tube, cortical patches were found to cluster at the incipient branch site ( Fig 5A, Fig 4). |I:\*\*1SP3E3| |L:\*\*1SP3E3| |T:\*\*1SP3E2| |
|  |  |  |  | Annotation |  | 1SP3E3 | 1SP3E3 |  |  |  | 1SP3E2 |  |
|  |  |  |  | Evidence |  | 4 | 4 |  |  |  | 3 |  |
|  |  |  |  | Focus |  | 4 | 4 |  |  |  | 4 |  |
|  |  |  |  | Polarity |  | 7 | 7 |  |  |  | 7 |  |
| 759 | 759 | 9736749\_104 | As shown in Fig. 4, the pattern of hippocampal shape variability in the control subjects was not related to the distribution of disease-related deformations. |I:\*\*1SN3E3| |L:\*\*1SN3E3| |T:\*\*1SN3E3| |
|  |  |  |  | Annotation |  | 1SN3E3 | 1SN3E3 |  |  |  | 1SN3E3 |  |
|  |  |  |  | Evidence |  | 4 | 4 |  |  |  | 4 |  |
|  |  |  |  | Focus |  | 4 | 4 |  |  |  | 4 |  |
|  |  |  |  | Polarity |  | 1 | 1 |  |  |  | 1 |  |
| 760 | 760 | 12538357\_9 | There was an absence of a mutually exclusive relationship between disruption of p53 and p19Arf, since the concordance was 62%. |I:\*\*1SN3E0| |L:\*\*1SP3E0| |T:\*\*1SP3E0| |
|  |  |  |  | Annotation |  | 1SN3E0 | 1SP3E0 |  |  |  | 1SP3E0 |  |
|  |  |  |  | Evidence |  | 1 | 1 |  |  |  | 1 |  |
|  |  |  |  | Focus |  | 4 | 4 |  |  |  | 4 |  |
|  |  |  |  | Polarity |  | 1 | 7 |  |  |  | 7 |  |
| 761 | 761 | 9844016\_76 | Reductions in schizophrenics (open bars) in comparison with matched controls (shaded bars) do not reach statistical significance, |I:\*\*1SGN3E3| |L:\*\*1SN3E3| |T:\*\*1SN3E3-| except marginally in layers II and III. |I:\*\*2SGP3E3| |L:\*\*2SP3E3| |T:\*\*2SP3E3| |
|  |  |  |  | Annotation |  | 1SGN3E3 2SGP3E3 | 1SN3E3 2SP3E3 |  |  |  | 1SN3E3- 2SP3E3 |  |
|  |  |  |  | Evidence |  | 4 4 | 4 4 |  |  |  | 4 4 |  |
|  |  |  |  | Focus |  | 5 5 | 4 4 |  |  |  | 4 4 |  |
|  |  |  |  | Polarity |  | 1 7 | 1 7 |  |  |  | 1 7 |  |
| 762 | 762 | 10591180\_243 | Lysates were subjected to immunoprecipitation with agarose-conjugated anti-phosphotyrosine antibodies. |I:\*\*1MP3E0| |L:\*\*1GSP3E0| |T:\*\*1MP3E3| |
|  |  |  |  | Annotation |  | 1MP3E0 | 1GSP3E0 |  |  |  | 1MP3E3 |  |
|  |  |  |  | Evidence |  | 1 | 1 |  |  |  | 4 |  |
|  |  |  |  | Focus |  | 2 | 5 |  |  |  | 2 |  |
|  |  |  |  | Polarity |  | 7 | 7 |  |  |  | 7 |  |
| 763 | 763 | 10809708\_162 | From a functional point of view, ClpC seems to combine properties of ClpA as well as ClpB ATPase in the direction of proteolysis while also protecting the cell from stress by resolubilization of protein aggregates (for a review, see references 9, 10, 11, and 43). |I:\*\*1SP3E2| |L:\*\*1GSP3E2| |T:\*\*1SP3E2| |
|  |  |  |  | Annotation |  | 1SP3E2 | 1GSP3E2 |  |  |  | 1SP3E2 |  |
|  |  |  |  | Evidence |  | 3 | 3 |  |  |  | 3 |  |
|  |  |  |  | Focus |  | 4 | 5 |  |  |  | 4 |  |
|  |  |  |  | Polarity |  | 7 | 7 |  |  |  | 7 |  |
| 764 | 764 | 12244472\_3 | pretherapeutic diagnosis of colorectal carcinoma, CTC can be combined with a staging CT of the abdomen. |I:\*\*ERROR| |L:\*\*1GSP3E0| |T:\*\*1GP3E0| |
|  |  |  |  | Annotation |  | ERROR | 1GSP3E0 |  |  |  | 1GP3E0 |  |
|  |  |  |  | Evidence |  | -1000 | 1 |  |  |  | 1 |  |
|  |  |  |  | Focus |  | -1000 | 5 |  |  |  | 1 |  |
|  |  |  |  | Polarity |  | -1000 | 7 |  |  |  | 7 |  |
| 765 | 765 | 9362059\_80 | Telomerase-containing extracts were prepared from mated Euplotes cells as described by Bednenko et al. (1997) . |I:\*\*1MP3E3| |L:\*\*1MP3E23| |T:\*\*1MP3E2| |
|  |  |  |  | Annotation |  | 1MP3E3 | 1MP3E23 |  |  |  | 1MP3E2 |  |
|  |  |  |  | Evidence |  | 4 | 3 |  |  |  | 3 |  |
|  |  |  |  | Focus |  | 2 | 2 |  |  |  | 2 |  |
|  |  |  |  | Polarity |  | 7 | 7 |  |  |  | 7 |  |
| 766 | 766 | 7874498\_81 | The line above the nucleotide sequence indicates the site homologous to the oligonucleotide probe (primer 5). |I:\*\*1MSP3E3| |L:\*\*1SP1E3| |T:\*\*1MP3E3| |
|  |  |  |  | Annotation |  | 1MSP3E3 | 1SP1E3 |  |  |  | 1MP3E3 |  |
|  |  |  |  | Evidence |  | 4 | 4 |  |  |  | 4 |  |
|  |  |  |  | Focus |  | 6 | 4 |  |  |  | 2 |  |
|  |  |  |  | Polarity |  | 7 | 5 |  |  |  | 7 |  |
| 767 | 767 | 11604494\_18 | In addition, at least four outer membrane proteins, Tom20p-Tom22p and Tom70p-Tom37p, make up two receptor systems with partially overlapping specificity for precursor binding ( 2, 4, 18, 23, 26, 33, 38, 39). |I:\*\*1SP3E2| |L:\*\*1SP3E2| |T:\*\*1SP3E2| |
|  |  |  |  | Annotation |  | 1SP3E2 | 1SP3E2 |  |  |  | 1SP3E2 |  |
|  |  |  |  | Evidence |  | 3 | 3 |  |  |  | 3 |  |
|  |  |  |  | Focus |  | 4 | 4 |  |  |  | 4 |  |
|  |  |  |  | Polarity |  | 7 | 7 |  |  |  | 7 |  |
| 768 | 768 | 12454064\_299 | In addition, no sperm nullisomic for an autosome were found in a direct search |I:\*\*1SN3E0| that would have allowed such offspring to survive. |I:\*\*2SP3E0| |L:\*\*1SN3E3| |T:\*\*1SP3E0| |
|  |  |  |  | Annotation |  | 1SN3E0 2SP3E0 | 1SN3E3 1SN3E3 |  |  |  | 1SP3E0 1SP3E0 |  |
|  |  |  |  | Evidence |  | 1 1 | 4 4 |  |  |  | 1 1 |  |
|  |  |  |  | Focus |  | 4 4 | 4 4 |  |  |  | 4 4 |  |
|  |  |  |  | Polarity |  | 1 7 | 1 1 |  |  |  | 7 7 |  |
| 769 | 769 | 10913072\_59 | The Pi protein is required for replication of pIVET1 derivatives. |I:\*\*1SP3E0| |L:\*\*1SP3E0| |T:\*\*1GP3E0| |
|  |  |  |  | Annotation |  | 1SP3E0 | 1SP3E0 |  |  |  | 1GP3E0 |  |
|  |  |  |  | Evidence |  | 1 | 1 |  |  |  | 1 |  |
|  |  |  |  | Focus |  | 4 | 4 |  |  |  | 1 |  |
|  |  |  |  | Polarity |  | 7 | 7 |  |  |  | 7 |  |
| 770 | 770 | 8616896\_244 | Clearly, the next step is to isolate and characterize the putative factor(s) |I:\*\*1SGP3E3| postulated to mediate specific inhibition of splicing induced by nonsense mutations. |I:\*\*2SP0E0| |L:\*\*1GSP3E0| |T:\*\*1GP3E3| |
|  |  |  |  | Annotation |  | 1SGP3E3 2SP0E0 | 1GSP3E0 1GSP3E0 |  |  |  | 1GP3E3 1GP3E3 |  |
|  |  |  |  | Evidence |  | 4 1 | 1 1 |  |  |  | 4 4 |  |
|  |  |  |  | Focus |  | 5 4 | 5 5 |  |  |  | 1 1 |  |
|  |  |  |  | Polarity |  | 7 4 | 7 7 |  |  |  | 7 7 |  |
| 771 | 771 | 9835589\_47 | Aliquots of culture (5 to 50 ml, depending on the biomass) were centrifuged at 14,000 x g for 8 min at 4 degrees C. |I:\*\*1MP3E0| Aliquots from the supernatant fluid were frozen for pyruvate analyses. |I:\*\*2MP3E0| |L:\*\*1MP3E3| |T:\*\*1MP3E3| |
|  |  |  |  | Annotation |  | 1MP3E0 2MP3E0 | 1MP3E3 1MP3E3 |  |  |  | 1MP3E3 1MP3E3 |  |
|  |  |  |  | Evidence |  | 1 1 | 4 4 |  |  |  | 4 4 |  |
|  |  |  |  | Focus |  | 2 2 | 2 2 |  |  |  | 2 2 |  |
|  |  |  |  | Polarity |  | 7 7 | 7 7 |  |  |  | 7 7 |  |
| 772 | 772 | 10553008\_239 | To test this hypothesis, |I:\*\*1SGP3E3| the expression of pancreatic lipase (PL) was examined in the mutant cells, |I:\*\*2SGP0E3| an enzyme structurally related to LPL but capable of renaturation to an enzymatically active state in vitro (see Experimental Procedures). |I:\*\*3SP3E3| |L:\*\*1GMSP3E3| |T:\*\*1MP3E3| |
|  |  |  |  | Annotation |  | 1SGP3E3 2SGP0E3 3SP3E3 | 1GMSP3E3 1GMSP3E3 1GMSP3E3 |  |  |  | 1MP3E3 1MP3E3 1MP3E3 |  |
|  |  |  |  | Evidence |  | 4 4 4 | 4 4 4 |  |  |  | 4 4 4 |  |
|  |  |  |  | Focus |  | 5 5 4 | 7 7 7 |  |  |  | 2 2 2 |  |
|  |  |  |  | Polarity |  | 7 4 7 | 7 7 7 |  |  |  | 7 7 7 |  |
| 773 | 773 | 9065447\_75 | A rapid displacement of labeled 123I-uPA by unlabeled uPA was observed, |T:\*\*1SP3E0| with a calculated dissociation rate constant of 1.8 x 10 3 s 1. |T:\*\*2SP3E3| |I:\*\*1SP3E0| |L:\*\*1SP3E3| |
|  |  |  |  | Annotation |  | 1SP3E0 1SP3E0 | 1SP3E3 1SP3E3 |  |  |  | 1SP3E0 2SP3E3 |  |
|  |  |  |  | Evidence |  | 1 1 | 4 4 |  |  |  | 1 4 |  |
|  |  |  |  | Focus |  | 4 4 | 4 4 |  |  |  | 4 4 |  |
|  |  |  |  | Polarity |  | 7 7 | 7 7 |  |  |  | 7 7 |  |
| 774 | 774 | 10090727\_119 | Although the area of expression is expanded in the compound mutant embryos as compared with wild-type embryos, |I:\*\*1SP3E0+| |L:\*\*1SP3E3| we favor the view that the expanded expression is due to the generation of excess neural ectoderm at the expense of mesoderm, |I:\*\*2SP1E3| |L:\*\*2SP1E3| |T:\*\*1SP3E0| rather than a loss of negative regulation, |I:\*\*3SN1E3| which has been shown to operate in the absence of Wnt signals (Cavallo et al. 1998 ; Waltzer and Bienz 1998 ). |I:\*\*4SP3E2| |L:\*\*3SN3E2| |T:\*\*2SN3E2| |
|  |  |  |  | Annotation |  | 1SP3E0+ 2SP1E3 3SN1E3 4SP3E2 | 1SP3E3 2SP1E3 3SN3E2 3SN3E2 |  |  |  | 1SP3E0 1SP3E0 2SN3E2 2SN3E2 |  |
|  |  |  |  | Evidence |  | 1 4 4 3 | 4 4 3 3 |  |  |  | 1 1 3 3 |  |
|  |  |  |  | Focus |  | 4 4 4 4 | 4 4 4 4 |  |  |  | 4 4 4 4 |  |
|  |  |  |  | Polarity |  | 7 5 3 7 | 7 5 1 1 |  |  |  | 7 7 1 1 |  |
| 775 | 775 | 12069872\_4 | RESULTS: CFS sufferers did not demonstrate any impairment in objective cognitive functioning compared to the control group, and objective performance was not related to their higher levels of depression or their level of fatigue. |I:\*\*1SN3E3| |L:\*\*1SN3E3| |T:\*\*1SN3E0| |
|  |  |  |  | Annotation |  | 1SN3E3 | 1SN3E3 |  |  |  | 1SN3E0 |  |
|  |  |  |  | Evidence |  | 4 | 4 |  |  |  | 1 |  |
|  |  |  |  | Focus |  | 4 | 4 |  |  |  | 4 |  |
|  |  |  |  | Polarity |  | 1 | 1 |  |  |  | 1 |  |
| 776 | 776 | 11294848\_52 | The similarity of CD curves verified that the structure of CP10A in the presence of DPC is comparable with that seen in the presence of POPC and mixed liposomes. |I:\*\*1SP3E0| |L:\*\*1SP2E0| |T:\*\*1SP3E0| |
|  |  |  |  | Annotation |  | 1SP3E0 | 1SP2E0 |  |  |  | 1SP3E0 |  |
|  |  |  |  | Evidence |  | 1 | 1 |  |  |  | 1 |  |
|  |  |  |  | Focus |  | 4 | 4 |  |  |  | 4 |  |
|  |  |  |  | Polarity |  | 7 | 6 |  |  |  | 7 |  |
| 777 | 777 | 11063707\_298 | W ALKER et al. 1999 chose to model a TTG1 structure based on the heterotrimeric G-protein beta-subunit, |I:\*\*1SGP3E2| which possesses a sevenfold symmetry ( S ONDEK et al. 1996 ), |I:\*\*2SP3E2| and infer that like the G-protein, TTG1 may be a signal transduction component. |I:\*\*3SP1E2| |L:\*\*1SP1E2| |T:\*\*1MGP3E2| |
|  |  |  |  | Annotation |  | 1SGP3E2 2SP3E2 3SP1E2 | 1SP1E2 1SP1E2 1SP1E2 |  |  |  | 1MGP3E2 1MGP3E2 1MGP3E2 |  |
|  |  |  |  | Evidence |  | 3 3 3 | 3 3 3 |  |  |  | 3 3 3 |  |
|  |  |  |  | Focus |  | 5 4 4 | 4 4 4 |  |  |  | 3 3 3 |  |
|  |  |  |  | Polarity |  | 7 7 5 | 5 5 5 |  |  |  | 7 7 7 |  |
| 778 | 778 | 11160370\_140 | In addition, it appears evident from the same data that the 18:1/18:0 labeling ratio was lower in CMPH than in healthy controls, at any time after isotope administration. |I:\*\*1SP2E0| |L:\*\*1SP3E0-| |T:\*\*1SP3E0| |
|  |  |  |  | Annotation |  | 1SP2E0 | 1SP3E0- |  |  |  | 1SP3E0 |  |
|  |  |  |  | Evidence |  | 1 | 1 |  |  |  | 1 |  |
|  |  |  |  | Focus |  | 4 | 4 |  |  |  | 4 |  |
|  |  |  |  | Polarity |  | 6 | 7 |  |  |  | 7 |  |
| 779 | 779 | 10924475\_194 | This work was funded by a Medical Research Council program grant to Michael Ashburner and David Gubb, a National Institutes of Health grant to Paul Adler, a Hong Kong Croucher Foundation Scholarship to Edwin Chan, and an Imperial Council Research Fund grant to Takashi Toda. |I:\*\*1GP3E3| |L:\*\*1GP3E3| |T:\*\*1GP3E0| |
|  |  |  |  | Annotation |  | 1GP3E3 | 1GP3E3 |  |  |  | 1GP3E0 |  |
|  |  |  |  | Evidence |  | 4 | 4 |  |  |  | 1 |  |
|  |  |  |  | Focus |  | 1 | 1 |  |  |  | 1 |  |
|  |  |  |  | Polarity |  | 7 | 7 |  |  |  | 7 |  |
| 780 | 780 | 9700171\_86 | The cells were left undisturbed in a CO2 incubator at 37 degrees C for 1 d, and the morphology of cell aggregates formed were observed under a Diaphot microscope ( Nikon, Inc., Melville, NY). |I:\*\*1MP3E0| |L:\*\*1MP3E3| |T:\*\*1MP3E2| |
|  |  |  |  | Annotation |  | 1MP3E0 | 1MP3E3 |  |  |  | 1MP3E2 |  |
|  |  |  |  | Evidence |  | 1 | 4 |  |  |  | 3 |  |
|  |  |  |  | Focus |  | 2 | 2 |  |  |  | 2 |  |
|  |  |  |  | Polarity |  | 7 | 7 |  |  |  | 7 |  |
| 781 | 781 | 12445773\_244 | It is also feasible for RNA to expand its functional group diversity by recruiting amino acids through thioesterification. |I:\*\*1SP1E0| |L:\*\*1SP3E0| |T:\*\*1GP3E0| |
|  |  |  |  | Annotation |  | 1SP1E0 | 1SP3E0 |  |  |  | 1GP3E0 |  |
|  |  |  |  | Evidence |  | 1 | 1 |  |  |  | 1 |  |
|  |  |  |  | Focus |  | 4 | 4 |  |  |  | 1 |  |
|  |  |  |  | Polarity |  | 5 | 7 |  |  |  | 7 |  |
| 782 | 782 | 11114897\_245 | However, for the T124I and A126T mutants, which form putative tighter dimers, progress past this step is blocked. |I:\*\*1SP3E0| |L:\*\*1SP3E0-| |T:\*\*1SN3E0| |
|  |  |  |  | Annotation |  | 1SP3E0 | 1SP3E0- |  |  |  | 1SN3E0 |  |
|  |  |  |  | Evidence |  | 1 | 1 |  |  |  | 1 |  |
|  |  |  |  | Focus |  | 4 | 4 |  |  |  | 4 |  |
|  |  |  |  | Polarity |  | 7 | 7 |  |  |  | 1 |  |
| 783 | 783 | 9360990\_24 | However, the simple induction of these four genes is unlikely to be enough |I:\*\*1SN2E0| to explain all of the in vivo effects of PDX-1. |I:\*\*2SGP0E0| |L:\*\*1SN1E0+| |T:\*\*1SN2E0| |
|  |  |  |  | Annotation |  | 1SN2E0 2SGP0E0 | 1SN1E0+ 1SN1E0+ |  |  |  | 1SN2E0 1SN2E0 |  |
|  |  |  |  | Evidence |  | 1 1 | 1 1 |  |  |  | 1 1 |  |
|  |  |  |  | Focus |  | 4 5 | 4 4 |  |  |  | 4 4 |  |
|  |  |  |  | Polarity |  | 2 4 | 3 3 |  |  |  | 2 2 |  |
| 784 | 784 | 10747890\_60 | Termination probability was calculated as outlined under "Experimental Procedures." |I:\*\*1MGP3E3| |L:\*\*1MP3E3| |T:\*\*1MP3E3| |
|  |  |  |  | Annotation |  | 1MGP3E3 | 1MP3E3 |  |  |  | 1MP3E3 |  |
|  |  |  |  | Evidence |  | 4 | 4 |  |  |  | 4 |  |
|  |  |  |  | Focus |  | 3 | 2 |  |  |  | 2 |  |
|  |  |  |  | Polarity |  | 7 | 7 |  |  |  | 7 |  |
| 785 | 785 | 10542235\_33 | Surprisingly, also the single deletion in PBP1B showed a similar change. |I:\*\*1SP3E0| |L:\*\*1SP3E3| |T:\*\*1SP3E0| |
|  |  |  |  | Annotation |  | 1SP3E0 | 1SP3E3 |  |  |  | 1SP3E0 |  |
|  |  |  |  | Evidence |  | 1 | 4 |  |  |  | 1 |  |
|  |  |  |  | Focus |  | 4 | 4 |  |  |  | 4 |  |
|  |  |  |  | Polarity |  | 7 | 7 |  |  |  | 7 |  |
| 786 | 786 | 10669745\_38 | Anti-green fluorescent protein antibodies were obtained from Clontech. |I:\*\*1MP3E0| |L:\*\*1MP3E3| |T:\*\*1MP3E3| |
|  |  |  |  | Annotation |  | 1MP3E0 | 1MP3E3 |  |  |  | 1MP3E3 |  |
|  |  |  |  | Evidence |  | 1 | 4 |  |  |  | 4 |  |
|  |  |  |  | Focus |  | 2 | 2 |  |  |  | 2 |  |
|  |  |  |  | Polarity |  | 7 | 7 |  |  |  | 7 |  |
| 787 | 787 | 11375170\_33 | The RNA concentration was determined |I:\*\*1SGP0E0| by measuring absorbance at optical densities of 260 and 280 nm (OD260/280). |I:\*\*2MP3E0| |L:\*\*1MP3E3| |T:\*\*1MP3E3| |
|  |  |  |  | Annotation |  | 1SGP0E0 2MP3E0 | 1MP3E3 1MP3E3 |  |  |  | 1MP3E3 1MP3E3 |  |
|  |  |  |  | Evidence |  | 1 1 | 4 4 |  |  |  | 4 4 |  |
|  |  |  |  | Focus |  | 5 2 | 2 2 |  |  |  | 2 2 |  |
|  |  |  |  | Polarity |  | 4 7 | 7 7 |  |  |  | 7 7 |  |
| 788 | 788 | 10790380\_113 | Rv3135, a member of the PPE family of proteins, was uniquely variable. |I:\*\*1SP3E0| |L:\*\*1SP3E0| |T:\*\*1SP3E0| |
|  |  |  |  | Annotation |  | 1SP3E0 | 1SP3E0 |  |  |  | 1SP3E0 |  |
|  |  |  |  | Evidence |  | 1 | 1 |  |  |  | 1 |  |
|  |  |  |  | Focus |  | 4 | 4 |  |  |  | 4 |  |
|  |  |  |  | Polarity |  | 7 | 7 |  |  |  | 7 |  |
| 789 | 789 | 9380037\_175 | The substitution of IIIS6 of the alpha1C channel with that of the alpha1E channel (EC2) resulted in a 4.5-fold decrease in the isradipine sensitivity (Fig. 1), |I:\*\*1SP3E3-| |T:\*\*1SP3E0-| which indicates that the amino acid residues in this transmembrane segment are necessary for the DHP interaction. |I:\*\*2SP2E3| |L:\*\*1SP3E3| |T:\*\*2SP2E0| |
|  |  |  |  | Annotation |  | 1SP3E3- 2SP2E3 | 1SP3E3 1SP3E3 |  |  |  | 1SP3E0- 2SP2E0 |  |
|  |  |  |  | Evidence |  | 4 4 | 4 4 |  |  |  | 1 1 |  |
|  |  |  |  | Focus |  | 4 4 | 4 4 |  |  |  | 4 4 |  |
|  |  |  |  | Polarity |  | 7 6 | 7 7 |  |  |  | 7 6 |  |
| 790 | 790 | 12034770\_251 | A TFF1 function as a differentiation factor is also consistent with in vivo observations. |I:\*\*1SP3E0| |L:\*\*1SP3E0| |T:\*\*1GP1E0| |
|  |  |  |  | Annotation |  | 1SP3E0 | 1SP3E0 |  |  |  | 1GP1E0 |  |
|  |  |  |  | Evidence |  | 1 | 1 |  |  |  | 1 |  |
|  |  |  |  | Focus |  | 4 | 4 |  |  |  | 1 |  |
|  |  |  |  | Polarity |  | 7 | 7 |  |  |  | 5 |  |
| 791 | 791 | 12164523\_6 | RESULTS: 29 cases were diagnosed in the dissolution stage, 2 in coalascence, and 5 in the resolution stage. |I:\*\*1SP3E3| |L:\*\*1SP3E3| |T:\*\*1MP3E3| |
|  |  |  |  | Annotation |  | 1SP3E3 | 1SP3E3 |  |  |  | 1MP3E3 |  |
|  |  |  |  | Evidence |  | 4 | 4 |  |  |  | 4 |  |
|  |  |  |  | Focus |  | 4 | 4 |  |  |  | 2 |  |
|  |  |  |  | Polarity |  | 7 | 7 |  |  |  | 7 |  |
| 792 | 792 | 7499334\_10 | Figure 1:Effect of G -Gly on [Ca ] mobilization and cAMP generation in AR4-2J cells. |I:\*\*1SGP3E3| |L:\*\*1GSP3E3| |T:\*\*1MP3E3| |
|  |  |  |  | Annotation |  | 1SGP3E3 | 1GSP3E3 |  |  |  | 1MP3E3 |  |
|  |  |  |  | Evidence |  | 4 | 4 |  |  |  | 4 |  |
|  |  |  |  | Focus |  | 5 | 5 |  |  |  | 2 |  |
|  |  |  |  | Polarity |  | 7 | 7 |  |  |  | 7 |  |
| 793 | 793 | 12137688\_3 | OBJECTIVES: To examine the evidence for the relative effectiveness of the main methods of anaesthesia (haematoma block, intravenous regional anaesthesia (IVRA), regional nerve blocks, sedation and general anaesthesia) as well as associated physical techniques and drug adjuncts used during the management of distal radial fractures in adults. |I:\*\*1SGP0E3| |L:\*\*1GSP3E3| |T:\*\*1MGP3E3| |
|  |  |  |  | Annotation |  | 1SGP0E3 | 1GSP3E3 |  |  |  | 1MGP3E3 |  |
|  |  |  |  | Evidence |  | 4 | 4 |  |  |  | 4 |  |
|  |  |  |  | Focus |  | 5 | 5 |  |  |  | 3 |  |
|  |  |  |  | Polarity |  | 4 | 7 |  |  |  | 7 |  |
| 794 | 794 | 11062251\_223 | After a 15-min incubation with cytochrome c and dATP, only a small fraction of intact PARP remained and, by 30 min, all intact PARP disappeared ( Fig 7 A). |I:\*\*1MP3E3| |L:\*\*1SP3E3-| |T:\*\*1MP3E3| |
|  |  |  |  | Annotation |  | 1MP3E3 | 1SP3E3- |  |  |  | 1MP3E3 |  |
|  |  |  |  | Evidence |  | 4 | 4 |  |  |  | 4 |  |
|  |  |  |  | Focus |  | 2 | 4 |  |  |  | 2 |  |
|  |  |  |  | Polarity |  | 7 | 7 |  |  |  | 7 |  |
| 795 | 795 | 11013259\_59 | We have used colchicine and vinblastine |I:\*\*1MP3E3| to characterize the residues in TMs 6, 11, and 12 |I:\*\*2SGP0E3| that were sensitive to inhibition by dBBn ( , ). |I:\*\*3SP3E2| |T:\*\*1MP3E3| Six mutants that were inhibited by dBBn, Y118C (TM2), V125C (TM2), S222 (TM4), S766C (TM8), I868C (TM10), and G872C (TM10), were tested for their ability to be protected from inhibition by dBBn in the presence of substrate. |I:\*\*4SGP0E0| |L:\*\*1GSP3E3| |T:\*\*2SN3E3| |
|  |  |  |  | Annotation |  | 1MP3E3 2SGP0E3 3SP3E2 4SGP0E0 | 1GSP3E3 1GSP3E3 1GSP3E3 1GSP3E3 |  |  |  | 1MP3E3 1MP3E3 1MP3E3 2SN3E3 |  |
|  |  |  |  | Evidence |  | 4 4 3 1 | 4 4 4 4 |  |  |  | 4 4 4 4 |  |
|  |  |  |  | Focus |  | 2 5 4 5 | 5 5 5 5 |  |  |  | 2 2 2 4 |  |
|  |  |  |  | Polarity |  | 7 4 7 4 | 7 7 7 7 |  |  |  | 7 7 7 1 |  |
| 796 | 796 | 10200329\_25 | Both issues can be effectively examined |I:\*\*1SGP3E0| by testing first-degree relatives of schizophrenic patients. |I:\*\*2MP3E0| |L:\*\*1GMP3E0| |T:\*\*1GP3E0| |
|  |  |  |  | Annotation |  | 1SGP3E0 2MP3E0 | 1GMP3E0 1GMP3E0 |  |  |  | 1GP3E0 1GP3E0 |  |
|  |  |  |  | Evidence |  | 1 1 | 1 1 |  |  |  | 1 1 |  |
|  |  |  |  | Focus |  | 5 2 | 3 3 |  |  |  | 1 1 |  |
|  |  |  |  | Polarity |  | 7 7 | 7 7 |  |  |  | 7 7 |  |
| 797 | 797 | 10858193\_127 | Unp-Ia was treated under the same conditions and then combined with Pr-Ib. |I:\*\*1MP3E0| |L:\*\*1MP3E3| |T:\*\*1MP3E3| |
|  |  |  |  | Annotation |  | 1MP3E0 | 1MP3E3 |  |  |  | 1MP3E3 |  |
|  |  |  |  | Evidence |  | 1 | 4 |  |  |  | 4 |  |
|  |  |  |  | Focus |  | 2 | 2 |  |  |  | 2 |  |
|  |  |  |  | Polarity |  | 7 | 7 |  |  |  | 7 |  |
| 798 | 798 | 9736749\_21 | An analysis of hippocampal shape as well as volume was carried out. |I:\*\*1SGP0E0| |L:\*\*1GMP3E3| |T:\*\*1MP3E3| |
|  |  |  |  | Annotation |  | 1SGP0E0 | 1GMP3E3 |  |  |  | 1MP3E3 |  |
|  |  |  |  | Evidence |  | 1 | 4 |  |  |  | 4 |  |
|  |  |  |  | Focus |  | 5 | 3 |  |  |  | 2 |  |
|  |  |  |  | Polarity |  | 4 | 7 |  |  |  | 7 |  |
| 799 | 799 | 11092858\_41 | The desired base substitutions were made directly in a pKK223-3-0A clone using mutagenic primers S210G/A, E213G/A, and E214G/A and the selection primer STOM (Table 2). |I:\*\*1MP3E3| |L:\*\*1MP3E3| |T:\*\*1MP3E3| |
|  |  |  |  | Annotation |  | 1MP3E3 | 1MP3E3 |  |  |  | 1MP3E3 |  |
|  |  |  |  | Evidence |  | 4 | 4 |  |  |  | 4 |  |
|  |  |  |  | Focus |  | 2 | 2 |  |  |  | 2 |  |
|  |  |  |  | Polarity |  | 7 | 7 |  |  |  | 7 |  |
| 800 | 800 | 11230167\_13 | When a fluorescent molecule is excited by plane-polarized light at the correct wavelength, the fluorescence emitted is also polarized. |I:\*\*1SP3E0| |L:\*\*1SP3E0| |T:\*\*1GP3E0| |
|  |  |  |  | Annotation |  | 1SP3E0 | 1SP3E0 |  |  |  | 1GP3E0 |  |
|  |  |  |  | Evidence |  | 1 | 1 |  |  |  | 1 |  |
|  |  |  |  | Focus |  | 4 | 4 |  |  |  | 1 |  |
|  |  |  |  | Polarity |  | 7 | 7 |  |  |  | 7 |  |
| 801 | 801 | 10523653\_105 | The resulting plasmid, YIp-PTP3Z, was linearized |I:\*\*1SGP3E2| by digestion with SnaBI and transformed into the wild-type haploid, BBY48 ( 1). |I:\*\*2MP3E2| |L:\*\*1MP3E23| |T:\*\*1MP3E3| |
|  |  |  |  | Annotation |  | 1SGP3E2 2MP3E2 | 1MP3E23 1MP3E23 |  |  |  | 1MP3E3 1MP3E3 |  |
|  |  |  |  | Evidence |  | 3 3 | 3 3 |  |  |  | 4 4 |  |
|  |  |  |  | Focus |  | 5 2 | 2 2 |  |  |  | 2 2 |  |
|  |  |  |  | Polarity |  | 7 7 | 7 7 |  |  |  | 7 7 |  |
| 802 | 802 | 11149951\_25 | Although the significance of dopamine receptors, as well as of other neurotransmitter receptors, in lymphocytes is still not clear, |I:\*\*1SN3E0| |L:\*\*1SN0E0| |T:\*\*1GN3E0| it has been suggested that they may reflect corresponding brain receptors. |I:\*\*2SP1E0| |L:\*\*2SP1E0| |T:\*\*2GP1E1| |
[truncated: 4,612,598 more chars]
