# Supplementary material for: How to Get the Most out of Your Curation Effort
Source: PLoS Comput Biol. 2009 May 22;5(5):e1000391. doi: 10.1371/journal.pcbi.1000391 (PMC2678295; doi:10.1371/journal.pcbi.1000391)
Supplement: Dataset S1 — Full dataset produced by the two-round annotation effort described in this study. (1.79 MB ZIP) [file pcbi.1000391.s002.zip › annotation-data/read-me.doc]

**Data:**

“original8.html” – an HTML table with the original 8-evaluator annotations. The dataset comprises 8 collections of sentences (“chunks”); each sentence within the same collection was analyzed by the same trio of annotators, with 8 distinct triplets of annotators overall. Annotators are encoded with letters *L*, *T*, *I*, *M*, *O*, *Y*, *A*, and *N*.

“additional5.html” – an analogous HTML table with additional set of 5-evaluator annotations per sentence; 1,000 sentences sampled from the set of nearly 10,000 original sentences. Annotators are encoded as A1, A2, A3, A4, and A5.

Note that the original alphanumeric annotation codes are converted into in integer codes differently in the two tables.

To understand rules and admissible codes of annotation please see

Wilbur WJ, Rzhetsky A, Shatkay H (2006) New directions in biomedical text annotation: definitions, guidelines and corpus construction. BMC Bioinformatics 7: 356.
