# Supplementary material for: How to Get the Most out of Your Curation Effort
Source: PLoS Comput Biol. 2009 May 22;5(5):e1000391. doi: 10.1371/journal.pcbi.1000391 (PMC2678295; doi:10.1371/journal.pcbi.1000391)
Supplement: Dataset S2 — Complete set of MatLab programs required to estimate model parameters (under both models described here) and to compute the posterior distribution of correct annotation values. (0.62 MB ZIP) [file pcbi.1000391.s003.zip › annotation-scripts/read-me.doc]

This is a set of MatLab scripts (MatLab 7.6.0.324, R2008a) implementing simulation of data and parameter estimation under models A and B described in the manuscript by A. Rzhetsky, H. Shatkay, and W.J. Wilbur “How to stop worrying and get most out of your curation effort.”

**We want to make certain that there is no warranty for this free software. You are more than welcome to modify and redistribute the source files at your own risk -- the appropriate attribution of credit would be most appreciated.**

**EstimateA** → estimates parameters under the model A (one can choose to estimate α-parameters with maximum likelihood, or use their definition in terms of ω*_i_*’s)

For example, **EstimateA(1,10,1,0)** estimates model parameters for dimension 1 (number of fragments), performs 10 independent estimates with random starting optimization values, uses informative prior distribution, and does not estimate alpha-parameters simultaneously with theta-parameters.

**EstimateB** → estimates parameters under the *full* model B

For example, **EstimateB(1,100,1)** estimates model parameters for dimension 1 (number of fragments), performs 100 independent estimates with random starting optimization values, and uses an informative prior distribution.

**EstimateBt** → estimates parameters under the model B-with-thetas

For example, **EstimateBt(1,5,0)** estimates model parameters for dimension 1 (number of fragments), performs 5 independent estimates with random starting optimization values, and uses an *un*informative prior distribution.

**simulateEstimateA** → simulates data under the model A, estimates parameters under models A and B

For example, **simulateEstimateA(3,10,2,'tstA.mat')** generates annotations with 3 admissible values, performs 10 cycles of simulation—estimation with 2 independent runs of optimization with random starting values, and writes the results to file ‘tstA.mat.’

**simulateEstimateB** → simulates data under the model B, estimates parameters under models A and B

For example, **simulateEstimateB(5,10,3,'tstB.mat')** generates annotations with 5 admissible values, performs 10 cycles of simulation—estimation with 3 independent runs of optimization with random starting values, and writes the results to file ‘tstB.mat.’

**testModels**  → benchmarks models A and B-with-thetas using real data set described in the manuscript. Specifically, using estimates of model parameters and 3-way disagreement annotations, each model predicts the correct annotation. The gold standard for judging the accuracy of the predictions is provided by additional set of 5-expert annotations for the same data. The function does not require input arguments.
